# Characterization of the Immune Microenvironmental Landscape of Lung Squamous Cell Carcinoma with Immune Cell Infiltration

**DOI:** 10.1155/2022/2361507

**Published:** 2022-11-11

**Authors:** Chunji Chen, Dongfang Tang, Chang Gu, Bin Wang, Yuanshan Yao, Rui Wang, Huibiao Zhang, Wen Gao

**Affiliations:** ^1^Shanghai Key Laboratory of Clinical Geriatric Medicine, Huadong Hospital Affiliated to Fudan University, No. 221 West Yanan Road, Shanghai, China; ^2^Department of Thoracic Surgery, Huadong Hospital Affiliated to Fudan University, No. 221 West Yanan Road, Shanghai 200040, China; ^3^Department of Thoracic Surgery, Shanghai Chest Hospital, Shanghai Jiao Tong University, No. 241 West Huaihai Road, Shanghai 200030, China; ^4^Department of Cardiothoracic Surgery, Xinhua Hospital, Shanghai Jiao Tong University School of Medicine, 1665 Kongjiang Road, Shanghai 200092, China

## Abstract

**Background:**

Increasing evidence supports that immune cell infiltration (ICI) patterns play a key role in the tumor progression of lung squamous cell carcinoma (LUSC). However, to date, the immune infiltration picture of LUSC has not been elucidated.

**Method:**

TCGA was used to download multiomics data from LUSC samples. At the same time, we included two datasets on lung squamous cell carcinoma, GSE17710 and GSE157010. To reveal the landscape of tumor immune microenvironment (TIME), the ESTIMATE algorithm, ssGSEA approach, and CIBERSORT analysis are used. To quantify the ICI pattern in a single tumor, consistent clustering is used to determine the LUSC subtype based on the ICI pattern, and principal component analysis (PCA) is used to obtain the ICI score. The prognostic value of the Kaplan-Meier curves is confirmed. GSEA (Gene Set Enrichment Analysis) was used to perform functional annotation. To investigate the immunotherapeutic effects of the ICI score, the immunophenotyping score (IPS) is used. Finally, analyze the mutation data with the “maftools” R package.

**Results:**

We identified four different immune infiltration patterns with different prognosis and biological characteristics in 792 LUSC samples. The identification of ICI patterns in individual tumors developed under ICI-related characteristic genes based on the ICI score helps to analyze the biological process, clinical results, immune cell infiltration, immunotherapy effects, and genetic variation. Immune failure is indicated by a high ICI score subtype marked by immunosuppression. Patients with low ICI scores have an abundance of efficient immune cells, which corresponds to the immunological activation phenotype and may have therapeutic benefits. The immunophenotypic score was used as a surrogate indicator of immunotherapy results, and samples with low ICI scores obtained significantly higher immunophenotypic scores. Finally, the relationship between the ICI score and tumor mutation burden (TMB) was proven.

**Conclusion:**

This study fully clarified the indispensable role of the ICI model in the complexity and diversity of TIME. The quantitative identification of ICI patterns in a single tumor will help draw the picture of TIME and further optimize precision immunotherapy.

## 1. Introduction

Non-small-cell lung cancer (NSCLC) is the world's most deadly and fatal cancer [1, 2], with lung squamous cell carcinoma (LUSC) accounting for roughly 20-25 percent of NSCLC cases [3]. Viable genomic mutations have completely changed the treatment paradigm for lung adenocarcinoma (LUAD). Due to the presence of operable oncogenes in 20%-60% of patients with LUAD, many patients choose targeted therapy as an option, resulting in improved clinical outcomes. In contrast, fewer manipulable oncogenes are defined in LUSC, which pose a greater challenge for the treatment of LUSC [4, 5].

Tumor immunotherapy has revolutionized the way LUSC is treated. The employment of antitumor immune responses to locate and destroy tumor cells by activating the host's immune system is known as immunotherapy. In recent years, a series of studies of immunotherapy for advanced squamous cell carcinoma of the lung have changed clinical practice guidelines. Based on studies such as KEYNOTE-024, KEYNOTE-042, and KEYNOTE-407, the 2020 CSCO guidelines recommend pembrolumab as a single agent (PD-L1 TPS ≥ 50% only) and pembrolumab in combination with paclitaxel and platinum for the first-line treatment of advanced squamous cell carcinoma of the lung. The RATIONALE 307 study, the CameL-sq study, the orientation -12 study, and IMpower110, all conducted by domestic scholars, have shown that immunotherapy has better safety and prognosis compared with conventional radiotherapy [6–9]. Furthermore, there is mounting evidence that immune infiltration in the TIME is a factor in LUSC prognosis prediction [10–12]. As a result, the autoimmune cell infiltration (ICI) profiling approach divides LUSC samples into molecular-specific subgroups according to ICI patterns, allowing for more personalized treatment and better therapeutic effects. However, there have been no studies that look at the full context of ICI pattern-mediated LUSC.

The GSE17710 and GSE157010 datasets, as well as genomic and transcriptome data from 792 LUSC samples from the TCGA-LUSC project, were employed to synthesize the possible interaction of the ICI pattern with the TIME context. The CIBERSORT algorithm, ssGSEA technique, and ESTIMATE algorithm were utilized to map the TIME landscape using the LUSC genomic data. Four separate subtypes of ICI patterns were identified using a consensus clustering approach. In addition, to identify ICI patterns in individual samples and evaluate immunotherapy response, an ICI-based scoring scheme was developed. Predicting immunotherapy response in various ICI scored samples will also provide promising insights for improved precision immunotherapy. Finally, the intrinsic relationship and synergistic effects between ICI scores and tumor mutation load (TMB) were demonstrated. In conclusion, our findings suggest that the ICI models play an inseparable role in shaping the diversity and complexity of TIME and help to tailor immunotherapy strategies for LUSC.

## 2. Materials and Methods

### 2.1. LUSC Datasets and Samples

A total of 792 LUSC sample datasets were procured from publicly available datasets (TCGA-LUSC from TCGA database, GSE17710 and GSE157010 from Array Express database), patient's information is shown in Table S1. The TCGA-LUSC gene-expression profiles were received in the Fragments Per Kilobase per Million (FPKM) format from the TCGA site (http://cancergenome.nih.gov) and then converted into TPMs (transcripts per kilobase million). GSE17710 is a GEO dataset of 56 patients with lung squamous cell carcinoma confirmed by postoperative pathology. The overall design was RNA from tumors and a common reference were hybridized to Agilent two-color microarrays, see https://www.ncbi.nlm.nih.gov/bioproject/PRJNA118343 for more information. And the GSE157010 is a GEO dataset of 235 patients with lung squamous cell carcinoma confirmed by postoperative pathology. The overall design was total RNA from squamous cell carcinoma specimens were extracted for mRNA profiling with microarray analysis, see https://www.ncbi.nlm.nih.gov/bioproject/PRJNA659803 for more information. To limit the possibility of batch effects induced by non-biotechnology differences across various data sets, the “ComBat” algorithm [13] is utilized.

### 2.2. Clustering of Tumor-Infiltrating Immune Cells by Consensus

Gene expression data from the TCGA and GEO cohorts was processed with the CIBERSORT program (http://cibersort.stanford.edu/) to generate a fraction matrix for ICI, which evaluated the abundance of 22 different leukocyte subpopulations [14]. The expression data (ESTIMATE) method [15] uses unique aspects of transcription profiles to infer tumor cell shape and tumor purity in stromal and immune cells in malignant tumors. ESTIMATE, immune, and stromal scores were generated using the ESTIMATE approach to predict amounts of infiltrating immune and stromal cells, which are used to infer tumor purity. In addition, based on the expression levels of 29 immune-related features, single-sample gene set enrichment analysis (ssGSEA) was done using the R package “GSEABase.” In this study, the unsupervised clustering “PAM” approach based on Euclidean and Ward's linkage was applied, with the “ConsensusClusterPlus” R package [16] being used to assure classification stability.

### 2.3. DEGs and Enrichment Analysis in Inter-ICI Clusters

The data are classified into ICI subgroups using the prior consensus clustering approach, and the genes associated with the ICI pattern are identified. Then, in these ICI patterns, use the “limma” R package to find the differentially expressed genes (DEGs) linked with ICI. DEGs with an adjusted *P* value less than 0.05 and an absolute multiple change more than 1.5 were considered to be significant and used in further research. Gene Ontology (GO) annotations were used to further understand the biological role of DEG.

### 2.4. Dimension Reduction and ICI Score Generation

The DEG value is utilized to classify TCGA patients using the unsupervised clustering approach, and the DEG values that are positively and negatively correlated with the cluster signature are referred to as ICI gene signatures A and B, respectively. The ICI gene signatures A and B are reduced in dimensionality using the Boruta technique, and the principal component 1 is extracted as the signature score using PCA. To determine each patient's ICI score, we employ an approach comparable to the gene expression grading index: ICI score = *Σ*PC1A − *Σ*PC1B.

## 3. Results

### 3.1. The Immuno-Cell Infiltration Landscape in LUSC's TIME

To measure the activity or enrichment levels of immune cells in LUSC tumor tissues, we employed the CIBERSORT and ESTIMATE algorithms (Tables S2 and S3) [14, 15]. Based on 792 immune cell infiltration (ICI) signature-matched tumor samples from the metacohort (array expression databases: GSE17710 and GSE157010; TCGA-LUSC (Cancer Genome Atlas)). To classify LUSC patients into distinct subgroups, unsupervised clustering was performed using the R program ConsensusClusterPlus.

**Figure 1:**
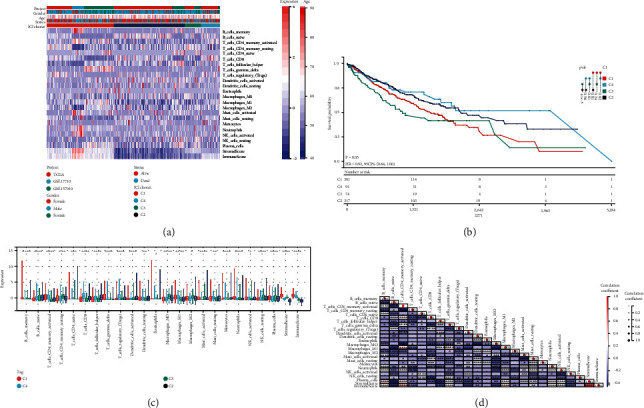
The immune cell infiltration landscape in the TIME context. (a) In LUSC patients, unsupervised clustering of tumor-infiltrating immune cells. (b) Kaplan-Meier curves for all LUSC patients in separate ICI clusters for overall survival (OS). (c) The infiltrating immune cell subpopulation, immune score, and stromal score in four ICI clusters. (d) The intrinsic link between infiltrating immune cells and immune scores. (∗*P* < 0.05; ∗∗*P* < 0.01; ∗∗∗*P* < 0.001; ∗∗∗∗*P* < 0.0001).

We discovered that *k* = 4 had the optimum clustering stability based on the ICI profiles' similarity. Significant clustering was identified by the increasing trend of the cumulative distribution function (CDF) values (Figures S1A–S1G). Model C1 (304 samples), model C2 (323 samples), model C3 (74 samples), and model C4 (91 samples) were the four ICI models identified using unsupervised clustering. The integrated heat map investigated and documented the link between the ICI models and clinical traits (Figure 1(a)). The Kaplan-Meier survival study of the four ICI models revealed that ICI clusters C2 and C4 had a considerable advantage in median survival time, whereas ICI cluster C1 had the worst prognosis (see Figure 1(b) for *P* values between the clusters). We provide a connection to depict the entire picture of TIME to further reveal the probable association between immunological scores and invading immune cells (Figure 1(c)). We analyzed the immune cell composition of TIME to learn more about the intrinsic biological distinctions that rise to diverse clinical presentations. ICI clusters C2 and C4 were shown to be related with a favorable prognosis among the four major immunological subtypes. They were characterized by a large infiltration of memory-activated CD4 T cells, CD8 T cells, follicular helper T cells, resting dendritic cells (DCs), M1 macrophages, activated mast cells, and activated NK cells. Patients with ICI cluster C3 were characterized by a significant density of naive B cells, memory-dormant CD4 T cells, M0 and M2 macrophages, dormant NK cells, and plasma cells. ICI cluster C1 patients were characterized by a significant increase in memory B cells, *γ*-T cells, and neutrophils. Stromal score were significantly higher in the C3 group than in the C2 and C4 groups. In addition, heat maps of correlation coefficients were generated to visualize the general picture of immune cell interactions in TIME (Figure 1(d)).

**Figure 2:**
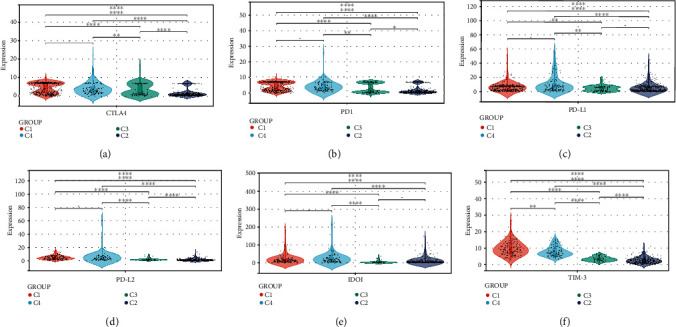
A comparison of ICB-relevant critical genes from different ICI clusters. CTLA4 (a), PD1 (b), PD-L1 (c), PD-L2 (d), IDO1 (e), and TIM-3 (f) expression levels in patients from different ICI clusters. The asterisks represented the statistical *P* value. (∗*P* < 0.05; ∗∗*P* < 0.01; ∗∗∗*P* < 0.001; ∗∗∗∗*P* < 0.0001).

We also evaluated the expression levels of six critical immune checkpoint blockade- (ICB-) related genes in the four ICI clusters, including CTLA4, IDO1, PD1, TIM3, PD-L1, and PD-L2. These results suggest that there may be differences in the selection of optimal ICB targets by different ICI clusters. For C2 and C4, CTLA-4, PD-L1, IDO1, and TIM3 seem to be more suitable, and for C3, CTLA4 may be more appropriate. As for C1, they seem to benefit from all these targets. (Figure 2).

### 3.2. Identified Immune Gene Subtype

**Figure 3:**
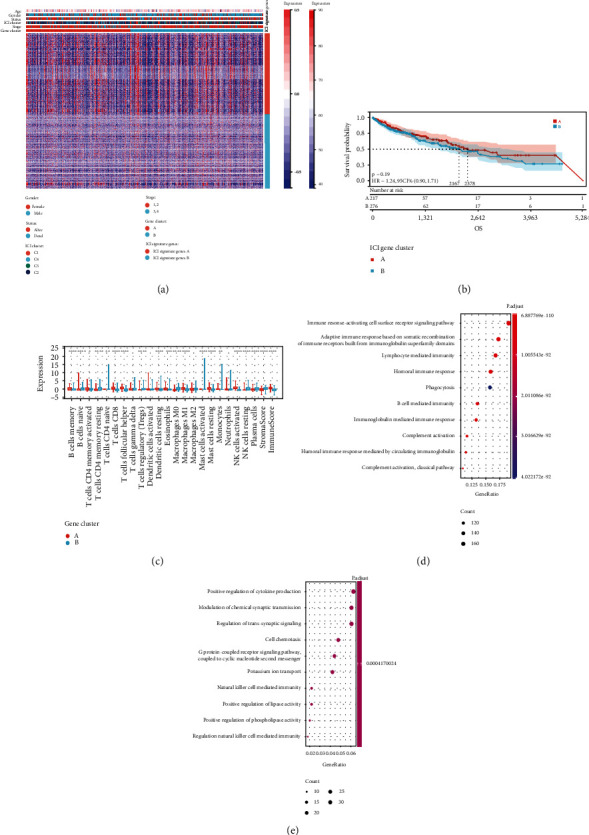
Subtypes of immunogenic genes are created. (a) Unsupervised clustering of common DEGs across three ICI cluster groups to divide patients into two subgroups: gene cluster A and B. (b) Analysis of overall survival in patients with two ICI-relevant signature genes. (c) The invading immune cell subpopulation, immune score, and stromal score in two ICI-relevant signature genes. (d, e) Analysis of Gene Ontology (GO) enrichment of the two ICI-relevant signature genes A (d) and B (e).

We did not combine the TCGA database and the GEO database due to the different data contexts of these two databases. In the subsequent analysis, the main focus was on the TCGA-LUSC cohort, which had the largest number of patients and the most detailed clinical information in this study. Using the limma package, we identified 2168 DEGs, which are regarded as critical indicators to identify distinct ICI symptoms, to investigate potential transcriptional expression alterations linked with ICI in diverse ICI patterns (Table S4). Unsupervised cluster analysis was used to classify the data into different transcriptome phenotypes (gene clusters A and B; Figures S2A–S2F) based on the 2168 most typical ICI phenotype-associated genes found, in order to better understand the underlying molecular mechanisms. There were 1131 DEGs with ICI-A gene signature positively associated with this gene cluster, and 1037 DEGs were introduced into the ICI-B gene signature (Table S5). The genetic differences between these genotypes were visualized using a heat map (Figure 3(a)). Kaplan-Meier curves were utilized for survival analysis to evaluate the prognostic value of ICI gene clustering. Although there was no statistically significant difference between the two gene clusters (*P* = 0.19; Figure 3(b)), the A gene cluster‘s mean survival time was longer than the B gene cluster's. The ESTIMATE algorithm and the CIBERSORT approach were utilized to estimate the relative subpopulation of infiltrating immune cells to elucidate the probable role of distinct gene clusters in TIME. Gene cluster A was strongly associated with memory B cells, CD4 memory-activated T cells, CD8 T cells, follicular helper T cells, DCs activation, eosinophils, M1 macrophages, mast cell dormancy, and NK cell activation, corresponding to the active immune phenotype [17, 18]. In contrast, gene cluster B shows increased infiltration of naive B cells, CD4 memory dormant T cells, Treg, M0 and M2 macrophages, and NK cell dormancy, termed the immunosuppressive phenotype [19, 20]. Gene cluster A, for example, had a higher immune score, implying an immunologically “hot” phenotype (Figure 3(c)). We discovered that the ICI signature gene A is enriched in the immune response-activating surface receptor signaling pathway, which appears to correspond with the “hot” immunophenotype, by exploring and showing the biologically meaningful enrichment using GSEA analysis (Figures 3(d) and 3(e)).

**Figure 4:**
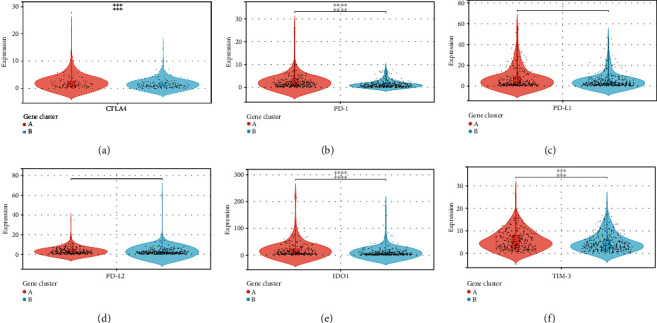
A comparison of ICB-relevant important genes from different ICI gene clusters. CTLA4 (a), PD-1 (b), PD-L1 (c), PD-L2 (d), IDO1 (e), and TIM-3 (f) expression levels in patients from different ICI gene clusters. (∗*P* < 0.05; ∗∗*P* < 0.01; ∗∗∗*P* < 0.001; ∗∗∗∗*P* < 0.0001).

Furthermore, in addition to PD-1 and PD-L1, the expression levels of ICB-related genes were significantly different across the two gene clusters (Figures 4(a)–4(f)). When compared to the ICI-B gene cluster, the ICI-A gene cluster dramatically boosted the expression levels of ICB-related genes, suggesting that the ICI-A gene cluster might benefit from immunotherapy.

### 3.3. Validation of the ICI Score in Lung Squamous Cell Carcinoma

**Figure 5:**
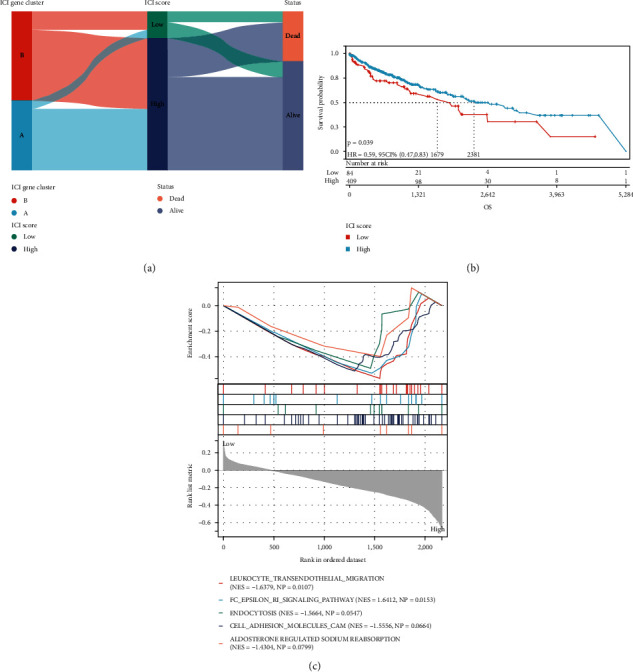
The ICI scores are being developed. (a) Sankey diagram illustrating the distribution of ICI gene clusters in subgroups with different ICI clusters, ICI scores, and survival status. (b) Kaplan-Meier curves for the TCGA-LUSC cohort's high and low ICI score groups. (c) Enrichment plots demonstrating that in patients with high ICI scores, the leukocyte transendothelial migratory signaling pathway was considerably activated.

Although the potential role of the ICI models in prognostic prediction and TIME information was found, the above analysis was performed only for the sample population and could not be performed accurately in individuals. To form quantitative indicators of ICI and use them for individual evaluation, we developed a scoring system called ICI score based on these characteristic genes associated with the ICI phenotype. Two composite scores were calculated using principal component analysis (PCA): [1] ICI score A (ISA) from signature gene A and [2] ICI score B (ISB) from signature gene B. The total and individual correlation of the individual accomplishment scale were used to determine the ICI scores and characteristics for each patient in this investigation (Table S6). Finally, we obtained prognostic characteristic scores, defined as ICI scores. Patients in the TCGA cohort were separated into two groups based on the best cut-off values achieved using the X-tile software: a high and a low ICI score group. The distribution of patients in the two gene clusters is represented by Sankey plots (Figure 5(a)).

The predictive significance of the ICI score in predicting overall survival was established by categorizing patients as having a high or low ICI score. As expected, survival was significantly poorer in the low-ICI score group than in the high-ICI score group (*P* = 0.039, Figure 5(b)); in patients with high ICI scores, the leukocyte transendothelial migratory signaling pathway was significantly active (Figure 5(c)).

### 3.4. Correlation of ICI Score and TIME Context in the Prognosis of Squamous Lung Cancer

**Figure 6:**
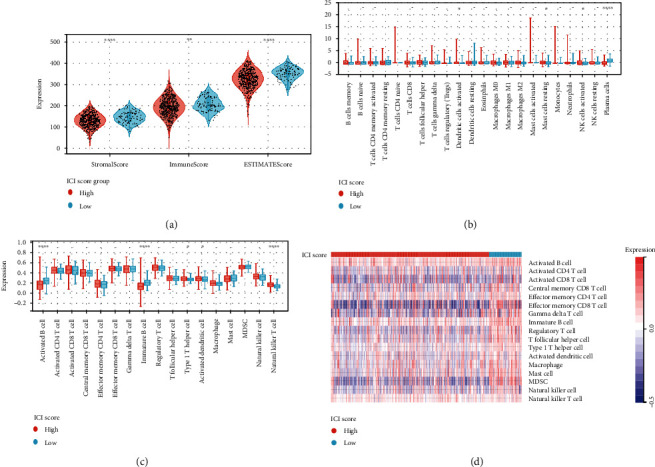
Correlation between ICI score and TIME characterization. (a) The ESTIMATE algorithm (estimate score, stromal score, and immune score) was compared among patients with varied ICI scores. (b) Infiltrating immune cell subgroups and levels differ across groups with low and high ICI scores. Violin plot (c) and heat map (d) showing the difference in immune-related signature enrichment between the ICI low and ICI high groups. CI gene clusters. (∗*P* < 0.05; ∗∗*P* < 0.01; ∗∗∗*P* < 0.001; ∗∗∗∗*P* < 0.0001).

We looked into how ICI scores might contribute to the complexities of the TIME. First, patients with low ICI scores had considerably higher stromal, immunological, and projected scores than those with high ICI ratings (Figure 6(a)). The results showed that patients with high ICI scores were significantly stronger in dendritic cell activation, mast cell resting, and NK cell activation than those with low ICI scores. In contrast, plasma cell expression was higher in patients with low ICI scores (Figure 6(b)). ssGSEA data revealed that immature B cells and B cell activation were considerably higher in patients with a low ICI score than in patients with a high ICI score, although natural killer T cells were the reverse (Figure 6(c)).

The heat map depicts the immunological enrichment for each patient in the low-/high-ICI score subgroup (Figure 6(d)). Based on these findings, we discovered substantial disparities in ICI phenotypes among the various ICI score samples. Subjects with low ICI scores expressed more immune cells than patients with high ICI scores, indicating an immune activation profile that may have an immunotherapeutic advantage.

### 3.5. Correlation between ICI Score and Immunotherapy and Construction of Prognosis Nomogram

**Figure 7:**
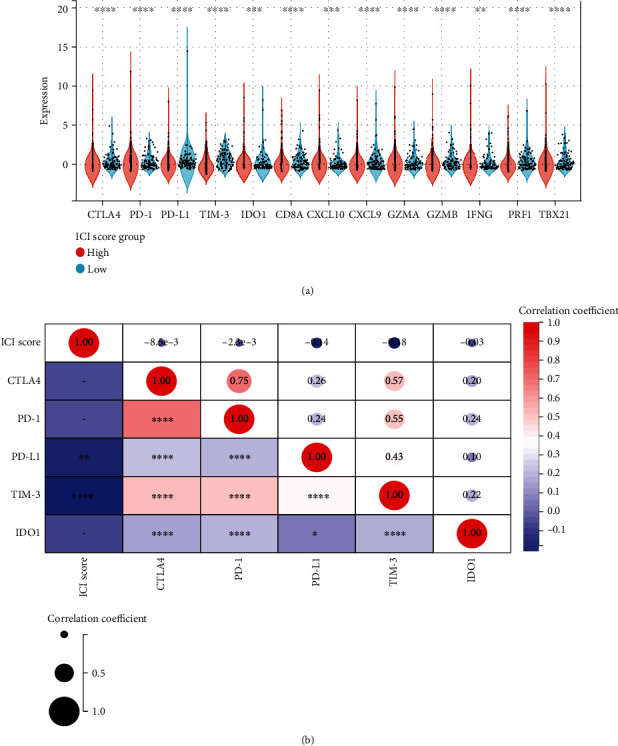
The immunotherapeutic importance of ICI scores. (a) Expression of ICB-related genes (CTLA4, PD-1, PD-L1, TIM-3, and IDO1) and inflammatory-related genes (CD8A, CXCL10, CXCL9, GZMA, GZMB, IFNG, PRF1, and TBX21) in high and low ICI score subgroups. There is a link between the ICI score and key immune checkpoint blockage genes. (b) Correlation analysis of immune checkpoint inhibitors with the ICI score (CTLA4, PD-1, PD-L1, TIM-3, and IDO1). (∗*P* < 0.05; ∗∗*P* < 0.01; ∗∗∗*P* < 0.001; ∗∗∗∗*P* < 0.0001).

We assessed the tolerance status and immune activity of the low-/high-ICI score subgroup. Analysis of the expression levels of ICB-related genes and inflammatory genes [21, 22] was shown in Figure 7(a). In patients with low ICI score, the expression levels of 13 genes related to immune activity and tolerance conditions were significantly upregulated (*P* < 0.001).

In addition, six ICB key target genes (PD-1, PD-L1, PD-L2, CTLA-4, TIM-3, and IDO1) were associated with ICI scores to reveal their potential role in ICB therapy for LUSC (Figure 7(b)). We found a significant negative correlation between ICI score and these six targets, suggesting that ICI score may play a significant role in predicting the response to ICB therapy in LUSC patients.

**Figure 8:**
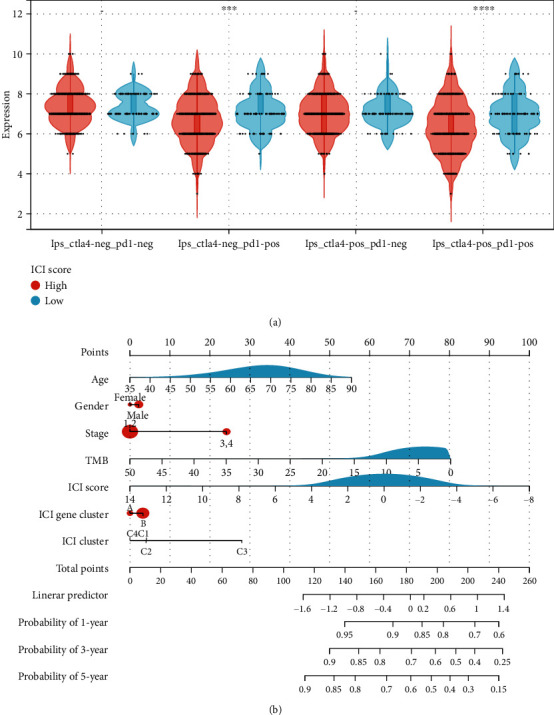
The estimation of the ICI score in immunotherapy response. (a) IPS-CTLA4_negative-PD-1_negative, IPS-CTLA4_negative-PD-1_positive, IPS-CTLA4_positive-PD-1_negative, and IPS-CTLA4_positive-PD-1_positive score distribution plot. (b) Nomogram was assembled by age and risk signature for predicting survival of LUSC patients. (∗∗∗*P* < 0.001; ∗∗∗∗*P* < 0.0001).

To predict the outcome of immunotherapy under ICI score, we used two subtypes of IPS values as substitutes for the immunotherapy response of LUSC patients (Table S7). In our prediction protocol, samples with low ICI scores had higher IPS-CTLA4_negative-PD-1_positive and IPS-CTLA4_positive-PD-1_positive scores (all *P* < 0.001; Figure 8(a)), indicating that LUSC samples with low ICI values may be suited for immunotherapy.

Meanwhile, using the stepwise Cox regression model, we created prognostic nomograms for OS prediction at 1, 3, and 5 years. We created a predictive nomogram comprising of ICI score to quantitatively determine the OS rate of individual patients (Figure 8(b)).

### 3.6. The Correlation between the ICI Scores and Tumor Mutation Burden

**Figure 9:**
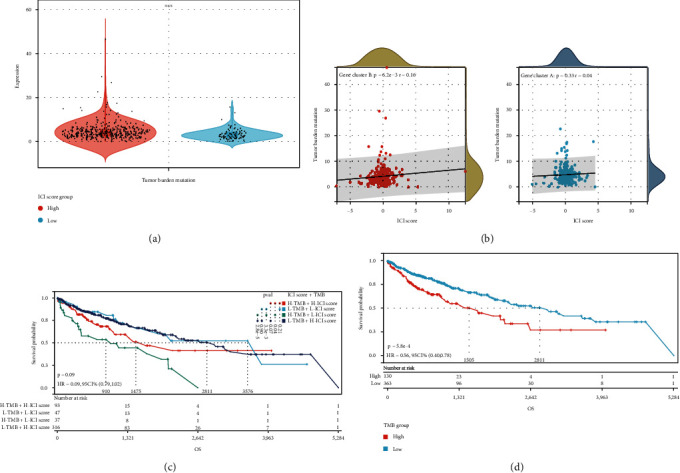
The ICI score and TMB have a correlation. (a) TMB differences between patients in low- and high-ICI score groupings. (b) Scatterplots illustrating the favorable relationship between ICI and TMB. (c) Kaplan-Meier curves for patients with TMB and ICI scores stratified. (d) Kaplan-Meier curves for the TMB groups with high and low TMB. (∗∗∗*P* < 0.001).

**Figure 10:**
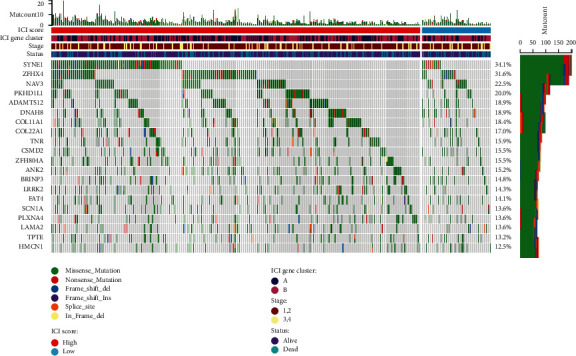
The oncoPrint was created by combining the high and low ICI values.

Previous research has found a link between high tumor burden mutations (TMB) and an increase in infiltrating CD8+ T lymphocytes that detect tumor neoantigens, resulting in a potent tumor-killing action that eliminates tumor cells [23–25]. As a result, we hypothesized that TMB may be used as a predictive factor for anticancer immunotherapeutic response and set out to look into the possible interaction between ICI score and TMB in order to uncover genetic diversity in ICI score subgroups. First, TMB levels in subgroups with low and high ICI scores were investigated. We found that the subgroup with the lowest ICI score had the highest TMB (*P* < 0.001, Figure 9(a)). TMB was found to be significantly positively linked with ICI score (*R* = 0.16, *P* < 0.001; Figure 9(b)) after further correlation analysis. The patients were then separated into subtypes based on their TMB immune setpoint, and we used the Kaplan-Meier analysis to show that having a low TMB signifies having a better chance of survival (*P* < 0.001,Figure 10(d)). We verified the synergistic effect of the two indicators in the prognosis prediction of LUSC to further explore the validity of the ICI score with the consistent prognostic importance of TMB. As shown by the stratified survival curve, the H-TMB+L-ICI score group had the worst outcomes, the L-TMB+H-ICI score group and the L-TMB+L-ICI score group had better prognosis, and the H-TMB+H-ICI group was in between (see Figure 9(c) for *P* values between the combined groups). Taken together, our findings imply that the ICI score could be used to assess the clinical success of antitumor immunotherapy as a significant prognostic predictor.

We also examined how somatic variations in LUSC driver genes differed between low and high ICI subsets. Using maftools [26] to access LUSC driver genes, the top 20 most often changed driver genes were further investigated (Figure 10). The altered frequencies of NFE2L2, TRIM51, and GOLGB1 were most significantly different between the low- and high-ICI score groups, according to mutation annotation data. These findings could lead to new insights into the mechanism of tumor ICI composition and gene mutation in the context of immune checkpoint blockade therapy.

## 4. Discussion

Immunotherapy is widely used to treat patients with non-small-cell lung cancer (NSCLC), and multiple standard of care (SOC) regimens have been approved for locally advanced and metastatic disease [27]. Early immunotherapy studies in non-small-cell lung cancer evaluated the efficacy of PD-L1 single-agent blockade therapy in previously treated advanced NSCLC and showed greater effectiveness than standard docetaxel chemotherapy. Thus, based on the results of a series of clinical studies, nivolumab, pembrolizumab, and atezolizumab were conditionally approved for the treatment of non-small-cell lung cancer [28–31], ushering in an era of immunotherapy for lung cancer and the attendant management challenges associated with immune-mediated toxicity [32, 33]. The traditional treatment for lung squamous cell carcinoma is chemoradiotherapy. The four commonly used chemotherapy drugs, gemcitabine, paclitaxel, docetaxel, and vinorelbine, are selected in combination with platinum, which is commonly used cisplatin and carboplatin. But overall, chemotherapy is relatively insensitive to lung squamous cell carcinoma. Nowadays, doctors also recommend sensitive gene testing for targeted drugs for patients with lung squamous cell carcinoma. Although the mutation rate of EGFR, ALK, and ROS genes in lung squamous cell carcinoma is low, less than 10%, there are still a few patients with mutant targets who can benefit from targeted drugs. It is an option for patients who cannot tolerate chemotherapy. In addition, immunotherapy plays a great role in lung squamous cell carcinoma. Among the lung cancer treated by nivolumab and pembrolizumab, lung squamous cell carcinoma has the best effect. Multiple clinical trials have confirmed that the effect of nivolumab and pembrolizumab in the treatment of lung squamous cell carcinoma is significantly better than chemotherapy, and the short-term death risk of patients is greatly reduced and the survival time is prolonged. The US FDA has approved pembrolizumab as a single-agent first-line treatment for patients with stage III or metastatic non-small-cell lung cancer who have PD-L1 expression ≥1% and no EGFR or ALK mutations. Multiple studies have shown that immunotherapy combined with chemotherapy has the opportunity to become the treatment of choice for patients with advanced lung squamous cell carcinoma, regardless of PD-L1 expression or TMB level. However, a notable limitation of immunotherapy is that only a minority of patients benefit from it. Even the Cancer Immunotherapy Association emphasizes that patients should be identified as candidates for immunotherapy. We developed a method to evaluate the integrated TIME of LUSC in this work. The ICI score appears to be a reliable predictive biomarker and predictor of immunotherapy response, according to our findings.

Here, our findings show that increased infiltration of activated CD4 T cells, CD8 T cells, follicular helper T cells, resting dendritic cells (DCs), M1 macrophages, activated mast cells, and activated NK cells is significantly associated with better overall survival. This shows that immune activity levels influence the clinical outcome of immunological therapies in the opposite direction of tumor growth. However, due to the heterogeneity of LUSC, a series of clinical investigations of immunotherapy for LUSC found disparities in objective response rates (ORR) and long-term survival [27], implying that immunophenotypes cannot correctly determine the outcome of immunotherapy.

Furthermore, we evaluated the ICI profiles of 792 LUSC patients from the combined TCGA-LUSC, GSE17710, and GSE157010 cohorts, and then used consensus clustering to divide these samples into four separate ICI subgroups. Varied immunophenotypes with different anticancer immune profiles are linked to the four different ICI patterns. The immunological rejection phenotype of ICI-C3 is characterized by a substantial invasion of quiescent immune cells rich in matrix components [34].

The ability of the host to participate in an antitumor immune response is determined by a variety of cytokines, chemokines, and other TIME components, according to LUSC's molecular research. We believe that the ICI profile and immune-related gene expression profile of patients with a combination of phenotypes would be a novel approach to specific therapeutic strategies, as these molecules in the altered process may interfere with aggressive cell signaling between immune cells, thus altering the balance among host defense and immune activity, and we believe that the ICI profile and immune-related gene expression profile of patients with a combination of phenotypes would be a novel approach to specific therapeutic strategies. Our main goal is to characterize the LUSC-TIME-regulated immune system at the molecular level; thus, we started by extracting immune-related genes from prior and novel ICI gene clusters. Among these gene clusters, ICI gene cluster B was found to have the lowest immune score and a higher matrix score, suggesting the presence of an immune hypothermic phenotype. In contrast, ICI gene cluster A had a higher immune score and inflammatory cell density. In addition, high stromal scores were observed to be associated with increased macrophage M0 and NK cell infiltration located in ICI gene cluster B, suggesting the presence of a humoral immune response in the gene cluster [18]. In addition, ICI gene cluster A had a favorable immune activation phenotype with the highest density of CD8+ T cells and activated CD4+ T cells [35]. We expected that individuals with ICI gene cluster A would benefit from immunotherapy since their antitumor immune response was associated with a positive prognosis. Our findings are consistent with earlier research, implying that the gene clusters identified in this work could lead to the creation of more specific immunotherapy.

Given the diversity of the individual immunological milieu, quantifying the ICI models for individual malignancies is very relevant. In breast cancer, esophageal cancer, and the head and neck squamous cell carcinoma, the individual-based models based on tumor subtype-specific biomarkers have been well established to improve prognostic prediction [36–38]. In this study, we established an ICI score to quantify the ICI pattern. By GSEA, we found that genes of immune activation pathways such as the leukocyte transendothelial migration signaling pathway were significantly enriched in the group with high ICI scores. In addition, we found that TMB was significantly lower in patients with lower ICI scores who were more sensitive to immunotherapy. The correlation between ICI score and TMB was 0.16.

We used the CIBERSORT, ESTIMATE, and ssGSEA algorithms to examine ICI patterns among subgroups with low and high ICI scores to further investigate the impact of ICI scores in TIME diversity and complexity. ICI scores were strongly positively linked with practically all immune cell infiltration, immunological scores, and immune-related markers, implying that ICI scores may be an indicator of immune activity. In addition, we verified that ICI scores were significantly positively correlated with key targets of ICB treatment (CTLA-4 and PD-1), suggesting that high ICI scores may be more sensitive to immunotherapy, while the opposite is true for PD-L1. Similarly, immunophenotype scores were upregulated, with Ips-CTLA4_negative-PD-1_positive, and Ips-ctla4_positive-pd-1_positive being higher in samples with lower ICI scores, considering that LUSC samples with lower ICI scores may be suitable for immunotherapy. These findings suggest that the ICI models may provide new insights into predicting ICB treatment outcomes in LUSC patients. We were unable to investigate the link between ICI scores and ICB immunotherapy response in the LUSC cohort due to the lack of an ICB treatment dataset. However, these findings will need to be confirmed in a broader cohort and at multiple centers.

Currently, some clinical data suggest an association between genetic alterations and immunotherapeutic response [39, 40]. The TMB (a predictor of immunotherapy sensitivity) was calculated and determined, and it rose considerably with the ICI score. In this study, we found that NFE2L2 and GOLGB1 had significantly higher mutation rates in subtypes with low ICI scores, while TRIM51 had increased mutation rates in patients with high ICI scores, which may provide a new target for ICB therapy. Differences in the distribution driven by mutations associated with ICI scores were significantly associated with antitumor immunity, highlighting the complex interaction between ICI patterns and somatic mutations in regulating the tumor immune genome complex interactions between ICI patterns and somatic mutations in regulating the tumor immune genome. Following that, stratified survival curves revealed that ICI scores have predictive power independent of TMB, implying that TMB and ICI scores are different components of immunology. Combined ICI score and TMB subgroup analysis showed that the H-TMB+L-ICI score group had the worst prognosis and the L-TMB+H-ICI score and L-TMB+L-ICI score groups had a better prognosis. Furthermore, at the genomic level, the ICI score paired with mutation data revealed significant variations in gene variation rates between low- and high-ICI score groups.

In summary, the complexity and heterogeneity of the tumor immune microenvironment is an important basis for regulating antitumor immunity and is analyzed comprehensively using a unique ICI model. A comprehensive assessment of ICI patterns in individual tumors will provide new insights to describe the TIME picture and guide precise immunotherapy strategies.

## Data Availability

The simulation experiment data used to support the findings of this study are available from the corresponding authors upon request.

## Supplementary Materials

Supplementary 1 Supplementary table 1: basic clinical data of 792 LUSC patients. Supplementary table 2: relative fractions of tumor-infiltrating immune cells of 792 LUSC patients. Supplementary table 3: relative immune scores of 792 LUSC patients. Supplementary table 4: differentially expressed genes of ICI-clusters in the TCGA cohort. Supplementary table 5: ICI signature genes A and B. Supplementary table 6: ICI score for each individual. Supplementary table 7: IPS score for each individual.

Supplementary 2 Supplementary Figure 1: consensus clustering based on the ICI patterns. (A–D) Consensus matrixes of BRCA samples for each *k* (*k* = 2–5), displaying the clustering stability using 1000 iterations of hierarchical clustering. (E) The relative change in area under the CDF curve for *k* = 2–9. (F) Area under cumulative distribution function (CDF) curve when index *k* ranges from 2 to 10. (G) Bar graph of consensus values when index *k* ranges from 2 to 10. Supplementary Figure 2: consensus clustering based on DEGs of different ICI clusters. (A) Consensus matrixes of BRCA cohorts for *k* = 2 displaying the clustering stability using 1000 iterations of hierarchical clustering. (B) Area under cumulative distribution function (CDF) curve when index *k* ranges from 2 to 10. (C) The relative change in area under the CDF curve for *k* = 2–10. (D) Bar graph of consensus values when index *k* ranges from 2 to 10.
