# Supplementary material for: Characterization of the Immune Microenvironmental Landscape of Lung Squamous Cell Carcinoma with Immune Cell Infiltration
Source: Dis Markers. 2022 Nov 11;2022:2361507. doi: 10.1155/2022/2361507 (PMC9674995; doi:10.1155/2022/2361507)
Supplement: Supplementary 1 — Supplementary table 1: basic clinical data of 792 LUSC patients. Supplementary table 2: relative fractions of tumor-infiltrating immune cells of 792 LUSC patients. Supplementary table 3: relative immune scores of 792 LUSC patients. Supplementary table 4: differentially expressed genes of ICI-clusters in the TCGA cohort. Supplementary table 5: ICI signature genes A and B. Supplementary table 6: ICI score for each individual. Supplementary table 7: IPS score for each individual. [file 2361507.f1.pdf]

Table S1- Patients information

ID	ICI score g	TMB grou	ICI score+TMB	ICI score	ICI gene cluster	ICI cluster	Stage	Age	Gender	Data source	ll survival (day)	Status
TCGA-34-	High	Low	L-TMB+H-ICI score	1.54404	A	C1	1,2	71	Femal	TCGA	2271	Alive
TCGA-96-	Low	High	H-TMB+L-ICI score	-0.9316	B	C1	1,2	83	Male	TCGA	2160	Alive
TCGA-85-	Low	Low	L-TMB+L-ICI score	-1.1379	A	C1	1,2	60	Femal	TCGA	910	Alive
TCGA-85-	High	High	H-TMB+H-ICI score	1.31615	A	C4	1,2	71	Male	TCGA	383	Alive
TCGA-85-	High	Low	L-TMB+H-ICI score	-0.0794	B	C1	1,2	62	Male	TCGA	455	Alive
TCGA-66-	High	High	H-TMB+H-ICI score	0.48593	B	C3	3,4	73	Male	TCGA	123	Dead
TCGA-22-	High	High	H-TMB+H-ICI score	0.48451	A	C1	1,2	71	Male	TCGA	669	Dead
TCGA-96-	High	Low	L-TMB+H-ICI score	1.62746	A	C4	1,2	78	Femal	TCGA	842	Alive
TCGA-77-	High	High	H-TMB+H-ICI score	-0.4646	A	C1	1,2	74	Femal	TCGA	1189	Dead
TCGA-63-	High	High	H-TMB+H-ICI score	0.85194	A	C2	1,2	67	Male	TCGA	0	Dead
TCGA-90-	High	High	H-TMB+H-ICI score	-0.0512	A	C2	1,2	70	Male	TCGA	428	Alive
TCGA-85-	High	High	H-TMB+H-ICI score	0.04297	B	C2	1,2	58	Male	TCGA	111	Alive
TCGA-37-	High	High	H-TMB+H-ICI score	0.46568	A	C4	1,2	65	Male	TCGA	13	Alive
TCGA-O2	High	High	H-TMB+H-ICI score	1.64748	B	C2	3,4	71	Femal	TCGA	340	Dead
TCGA-58-	High	High	H-TMB+H-ICI score	0.74257	A	C3	1,2	70	Male	TCGA	911	Alive
TCGA-66-	Low	Low	L-TMB+L-ICI score	-0.819	B	C4	1,2	71	Male	TCGA	61	Alive
TCGA-77-	High	High	H-TMB+H-ICI score	0.33032	B	C2	1,2	66	Femal	TCGA	351	Dead
TCGA-98-	High	High	H-TMB+H-ICI score	-0.7235	B	C2	1,2	67	Male	TCGA	826	Alive
TCGA-66-	High	High	H-TMB+H-ICI score	-0.1619	A	C4	1,2	65	Male	TCGA	60	Alive
TCGA-43-	High	Low	L-TMB+H-ICI score	0.54584	B	C2	1,2	70	Male	TCGA	849	Alive
TCGA-22-	High	High	H-TMB+H-ICI score	0.35531	A	C2	1,2	67	Male	TCGA	1975	Dead
TCGA-60-	Low	Low	L-TMB+L-ICI score	-1.5816	B	C1	1,2	73	Male	TCGA	1154	Dead
TCGA-77-	High	High	H-TMB+H-ICI score	0.24714	B	C2	1,2	74	Male	TCGA	2224	Dead
TCGA-63-	Low	Low	L-TMB+L-ICI score	-2.1814	B	C2	1,2	68	Male	TCGA	2148	Alive
TCGA-66-	High	High	H-TMB+H-ICI score	-0.3319	B	C1	1,2	62	Femal	TCGA	1311	Alive
TCGA-43-	Low	High	H-TMB+L-ICI score	-2.2177	B	C1	3,4	47	Femal	TCGA	1176	Alive
TCGA-52-	High	Low	L-TMB+H-ICI score	-0.4461	B	C2	1,2	67	Male	TCGA	266	Dead
TCGA-85-	Low	High	H-TMB+L-ICI score	-1.8357	B	C1	1,2	72	Male	TCGA	23	Alive
TCGA-85-	High	Low	L-TMB+H-ICI score	0.99308	B	C4	1,2	63	Male	TCGA	61	Alive
TCGA-94-	High	Low	L-TMB+H-ICI score	0.60285	A	C2	1,2	73	Male	TCGA	640	Alive
TCGA-60-	High	Low	L-TMB+H-ICI score	-0.7151	B	C1	1,2	41	Male	TCGA	372	Dead
TCGA-77-	High	Low	L-TMB+H-ICI score	0.01001	B	C3	3,4	73	Male	TCGA	212	Dead
TCGA-85-	High	High	H-TMB+H-ICI score	0.03992	B	C3	1,2	71	Male	TCGA	911	Alive
TCGA-39-	High	High	H-TMB+H-ICI score	1.95209	B	C2	3,4	59	Male	TCGA	519	Dead
TCGA-66-	Low	High	H-TMB+L-ICI score	-0.8528	A	C1	3,4	68	Male	TCGA	122	Alive
TCGA-33-	High	High	H-TMB+H-ICI score	-0.4233	B	C2	3,4	57	Male	TCGA	428	Dead
TCGA-NC	High	High	H-TMB+H-ICI score	1.39553	A	C2	1,2	53	Male	TCGA	37	Alive
TCGA-22-	High	High	H-TMB+H-ICI score	1.39343	B	C1	1,2	69	Male	TCGA	131	Dead
TCGA-60-	High	High	H-TMB+H-ICI score	0.63113	A	C2	1,2	74	Femal	TCGA	642	Alive
TCGA-77-	High	High	H-TMB+H-ICI score	-0.6417	A	C2	1,2	70	Male	TCGA	1884	Alive
TCGA-63-	High	High	H-TMB+H-ICI score	-0.163	B	C4	1,2	56	Male	TCGA	769	Alive
TCGA-22-	High	Low	L-TMB+H-ICI score	-0.1717	B	C2	1,2	61	Male	TCGA	223	Dead
TCGA-21-	High	High	H-TMB+H-ICI score	0.18106	B	C2	1,2	69	Male	TCGA	284	Dead
TCGA-O2	High	High	H-TMB+H-ICI score	-0.1563	B	C2	1,2	75	Femal	TCGA	1335	Dead
TCGA-33-	High	High	H-TMB+H-ICI score	0.80011	B	C2	1,2	66	Male	TCGA	211	Dead
TCGA-63-	High	High	H-TMB+H-ICI score	-0.4769	B	C2	1,2	67	Male	TCGA	0	Dead
TCGA-96-	High	High	H-TMB+H-ICI score	0.34545	B	C2	1,2	75	Femal	TCGA	531	Alive
TCGA-63-	High	High	H-TMB+H-ICI score	2.86557	B	C3	1,2	76	Male	TCGA	1639	Alive
TCGA-37-	High	High	H-TMB+H-ICI score	-0.5751	A	C1	1,2	69	Male	TCGA	12	Alive
TCGA-56-	High	Low	L-TMB+H-ICI score	-0.6436	B	C2	1,2	70	Male	TCGA	405	Alive
TCGA-56-	High	High	H-TMB+H-ICI score	3.77414	B	C2	1,2	56	Male	TCGA	150	Alive
TCGA-98-	High	High	H-TMB+H-ICI score	2.84831	B	C1	1,2	76	Femal	TCGA	618	Alive
TCGA-94-	High	High	H-TMB+H-ICI score	0.39569	A	C2	1,2	71	Femal	TCGA	430	Alive
TCGA-43-	High	High	H-TMB+H-ICI score	2.49428	A	C2	1,2	75	Femal	TCGA	2378	Alive
TCGA-NC	High	High	H-TMB+H-ICI score	-0.7493	A	C2	1,2	77	Male	TCGA	1499	Alive
TCGA-63-	Low	High	H-TMB+L-ICI score	-1.1087	B	C4	1,2	67	Male	TCGA	2142	Alive
TCGA-22-	High	High	H-TMB+H-ICI score	0.51192	A	C4	1,2	75	Male	TCGA	1845	Alive
TCGA-77-	High	High	H-TMB+H-ICI score	0.41257	B	C4	1,2	74	Male	TCGA	1640	Dead
TCGA-37-	High	High	H-TMB+H-ICI score	0.48811	A	C1	1,2	49	Male	TCGA	867	Alive
TCGA-85-	High	High	H-TMB+H-ICI score	0.89373	A	C2	1,2	52	Male	TCGA	815	Alive
TCGA-NC	High	High	H-TMB+H-ICI score	0.81118	B	C3	3,4	69	Male	TCGA	770	Alive
TCGA-85-	High	Low	L-TMB+H-ICI score	4.53431	A	C4	1,2	61	Male	TCGA	741	Alive
TCGA-33-	High	High	H-TMB+H-ICI score	0.27969	A	C2	1,2	55	Male	TCGA	3149	Dead
TCGA-43-	High	High	H-TMB+H-ICI score	0.26494	A	C2	1,2	59	Femal	TCGA	550	Alive
TCGA-34-	High	High	H-TMB+H-ICI score	0.51767	A	C2	1,2	80	Male	TCGA	1031	Alive
TCGA-56-	High	High	H-TMB+H-ICI score	-0.0617	A	C2	1,2	74	Male	TCGA	510	Alive
TCGA-18-	High	High	H-TMB+H-ICI score	-0.0013	B	C2	1,2	50	Male	TCGA	827	Dead
TCGA-21-	Low	Low	L-TMB+L-ICI score	-1.0015	A	C4	1,2	80	Femal	TCGA	1268	Alive
TCGA-90-	High	High	H-TMB+H-ICI score	-0.6047	B	C2	1,2	69	Male	TCGA	615	Alive
TCGA-MF	High	High	H-TMB+H-ICI score	-0.2956	B	C2	1,2	54	Male	TCGA	360	Alive
TCGA-46-	High	High	H-TMB+H-ICI score	-0.6162	B	C2	3,4	58	Male	TCGA	299	Dead
TCGA-77-	Low	High	H-TMB+L-ICI score	-1.5691	A	C1	3,4	62	Femal	TCGA	2133	Dead
TCGA-56-	High	Low	L-TMB+H-ICI score	0.51221	B	C2	1,2	66	Male	TCGA	543	Alive
TCGA-85-	High	High	H-TMB+H-ICI score	-0.526	B	C2	1,2	71	Male	TCGA	960	Alive
TCGA-56-	High	High	H-TMB+H-ICI score	-0.5422	B	C2	3,4	61	Male	TCGA	951	Alive
TCGA-NC	High	High	H-TMB+H-ICI score	0.18439	A	C2	3,4	59	Male	TCGA	1963	Alive
TCGA-56-	Low	High	H-TMB+L-ICI score	-0.9517	B	C2	1,2	72	Male	TCGA	105	Alive
TCGA-60-	High	High	H-TMB+H-ICI score	0.32033	B	C3	1,2	73	Male	TCGA	2945	Dead
TCGA-39-	High	High	H-TMB+H-ICI score	-0.5007	A	C2	1,2	73	Male	TCGA	2165	Alive
TCGA-33-	High	High	H-TMB+H-ICI score	0.20045	A	C2	3,4	71	Male	TCGA	601	Dead
TCGA-34-	Low	Low	L-TMB+L-ICI score	-1.759	B	C1	1,2	84	Male	TCGA	1000	Dead
TCGA-39-	High	High	H-TMB+H-ICI score	0.35741	A	C2	3,4	65	Femal	TCGA	2510	Alive
TCGA-77-	High	High	H-TMB+H-ICI score	0.80456	A	C2	1,2	59	Femal	TCGA	2227	Alive
TCGA-85-	High	High	H-TMB+H-ICI score	1.12182	A	C2	1,2	61	Male	TCGA	683	Alive
TCGA-O2	High	High	H-TMB+H-ICI score	-0.2057	B	C4	3,4	44	Femal	TCGA	113	Dead
TCGA-68-	High	High	H-TMB+H-ICI score	-0.2026	A	C2	1,2	66	Male	TCGA	244	Alive
TCGA-70-	High	Low	L-TMB+H-ICI score	0.19814	A	C2	1,2	65	Male	TCGA	375	Alive
TCGA-68-	High	High	H-TMB+H-ICI score	1.05175	B	C1	1,2	78	Male	TCGA	406	Alive
TCGA-85-	High	High	H-TMB+H-ICI score	-0.2714	A	C2	1,2	49	Male	TCGA	1063	Alive
TCGA-66-	High	High	H-TMB+H-ICI score	1.45638	A	C2	3,4	68	Male	TCGA	30	Alive
TCGA-85-	Low	High	H-TMB+L-ICI score	-3.134	A	C1	1,2	66	Male	TCGA	1224	Alive
TCGA-94-	High	High	H-TMB+H-ICI score	0.88289	B	C2	1,2	80	Male	TCGA	559	Alive
TCGA-22-	Low	Low	L-TMB+L-ICI score	-3.4492	B	C4	1,2	73	Male	TCGA	53	Dead
TCGA-85-	High	High	H-TMB+H-ICI score	0.69447	A	C2	1,2	73	Male	TCGA	550	Alive
TCGA-63-	High	High	H-TMB+H-ICI score	1.5471	B	C2	1,2	68	Male	TCGA	2026	Alive
TCGA-94-	High	High	H-TMB+H-ICI score	-0.3459	B	C2	1,2	70	Male	TCGA	153	Alive

TCGA-85-	High	High	H-TMB+H-ICI score	-0.2802	B	C3	1,2	70	Male	TCGA	236	Alive
TCGA-60-	High	Low	L-TMB+H-ICI score	0.53458	B	C2	1,2	39	Male	TCGA	1475	Alive
TCGA-O2	High	High	H-TMB+H-ICI score	-0.4238	B	C2	3,4	57	Femal	TCGA	387	Dead
TCGA-51-	High	High	H-TMB+H-ICI score	-0.2236	B	C2	3,4	65	Male	TCGA	12	Dead
TCGA-22-	Low	High	H-TMB+L-ICI score	-0.8782	B	C2	1,2	58	Femal	TCGA	916	Dead
TCGA-85-	High	Low	L-TMB+H-ICI score	-0.3134	B	C1	1,2	63	Femal	TCGA	294	Alive
TCGA-85-	Low	High	H-TMB+L-ICI score	-2.208	B	C1	1,2	62	Male	TCGA	1050	Alive
TCGA-77-	Low	Low	L-TMB+L-ICI score	-1.1801	B	C1	1,2	68	Male	TCGA	2639	Dead
TCGA-39-	Low	Low	L-TMB+L-ICI score	-0.871	B	C1	3,4	75	Male	TCGA	52	Dead
TCGA-34-	High	High	H-TMB+H-ICI score	-0.1492	B	C3	1,2	83	Femal	TCGA	1196	Alive
TCGA-43-	Low	Low	L-TMB+L-ICI score	-7.0614	B	C1	1,2	76	Male	TCGA	116	Dead
TCGA-63-	High	High	H-TMB+H-ICI score	0.59978	B	C2	3,4	65	Male	TCGA	1784	Alive
TCGA-63-	High	High	H-TMB+H-ICI score	0.20279	A	C2	1,2	68	Male	TCGA	1386	Alive
TCGA-39-	Low	Low	L-TMB+L-ICI score	-4.4515	A	C1	3,4	81	Femal	TCGA	59	Dead
TCGA-51-	Low	High	H-TMB+L-ICI score	-1.7076	B	C2	1,2	73	Femal	TCGA	12	Dead
TCGA-22-	High	High	H-TMB+H-ICI score	-0.0574	B	C2	1,2	74	Male	TCGA	1713	Dead
TCGA-66-	Low	High	H-TMB+L-ICI score	-0.953	B	C1	1,2	71	Male	TCGA	30	Alive
TCGA-33-	High	High	H-TMB+H-ICI score	0.06955	B	C2	1,2	68	Male	TCGA	2419	Alive
TCGA-52-	High	High	H-TMB+H-ICI score	0.16592	B	C2	1,2	60	Femal	TCGA	923	Alive
TCGA-77-	High	High	H-TMB+H-ICI score	-0.7439	B	C2	1,2	56	Male	TCGA	4261	Alive
TCGA-66-	Low	High	H-TMB+L-ICI score	-1.1422	B	C4	3,4	70	Male	TCGA	1492	Alive
TCGA-33-	High	High	H-TMB+H-ICI score	-0.7569	A	C4	1,2	40	Male	TCGA	5287	Dead
TCGA-90-	Low	Low	L-TMB+L-ICI score	-1.2005	B	C4	1,2	66	Femal	TCGA	289	Alive
TCGA-60-	High	Low	L-TMB+H-ICI score	-0.4652	B	C3	1,2	70	Male	TCGA	667	Dead
TCGA-56-	High	High	H-TMB+H-ICI score	0.89301	B	C1	1,2	78	Male	TCGA	616	Alive
TCGA-85-	High	High	H-TMB+H-ICI score	-0.4537	B	C3	1,2	65	Male	TCGA	687	Alive
TCGA-60-	High	Low	L-TMB+H-ICI score	0.50836	B	C1	1,2	73	Male	TCGA	983	Alive
TCGA-22-	High	Low	L-TMB+H-ICI score	0.44485	A	C1	1,2	69	Femal	TCGA	17	Dead
TCGA-33-	High	High	H-TMB+H-ICI score	-0.0448	B	C2	1,2	67	Male	TCGA	9	Alive
TCGA-60-	High	Low	L-TMB+H-ICI score	2.09585	B	C1	1,2	51	Male	TCGA	1075	Dead
TCGA-85-	High	Low	L-TMB+H-ICI score	0.59103	A	C2	1,2	72	Male	TCGA	510	Alive
TCGA-58-	High	High	H-TMB+H-ICI score	-0.363	A	C2	1,2	61	Male	TCGA	1072	Alive
TCGA-37-	High	Low	L-TMB+H-ICI score	5.22516	B	C2	1,2	52	Femal	TCGA	242	Alive
TCGA-21-	High	High	H-TMB+H-ICI score	-0.4789	B	C2	1,2	57	Male	TCGA	2134	Alive
TCGA-43-	High	High	H-TMB+H-ICI score	0.71101	A	C4	1,2	71	Male	TCGA	596	Alive
TCGA-58-	High	High	H-TMB+H-ICI score	2.22083	A	C1	1,2	60	Male	TCGA	412	Alive
TCGA-98-	High	Low	L-TMB+H-ICI score	0.0951	A	C2	1,2	77	Male	TCGA	630	Alive
TCGA-85-	High	High	H-TMB+H-ICI score	0.58443	B	C2	1,2	64	Male	TCGA	1111	Alive
TCGA-34-	High	High	H-TMB+H-ICI score	0.29343	B	C2	1,2	72	Male	TCGA	1984	Alive
TCGA-34-	High	High	H-TMB+H-ICI score	0.25373	B	C2	1,2	78	Femal	TCGA	151	Dead
TCGA-22-	Low	High	H-TMB+L-ICI score	-0.8965	A	C4	3,4	73	Femal	TCGA	506	Dead
TCGA-98-	High	Low	L-TMB+H-ICI score	3.8209	B	C1	1,2	77	Femal	TCGA	822	Alive
TCGA-94-	High	High	H-TMB+H-ICI score	0.69166	B	C4	1,2	73	Male	TCGA	810	Alive
TCGA-90-	High	Low	L-TMB+H-ICI score	1.34877	A	C1	1,2	56	Male	TCGA	89	Alive
TCGA-96-	High	Low	L-TMB+H-ICI score	-0.5609	B	C2	1,2	67	Femal	TCGA	557	Alive
TCGA-34-	High	High	H-TMB+H-ICI score	0.16876	B	C2	1,2	70	Male	TCGA	80	Dead
TCGA-56-	High	High	H-TMB+H-ICI score	-0.1239	A	C2	1,2	78	Male	TCGA	440	Alive
TCGA-21-	High	Low	L-TMB+H-ICI score	0.146	B	C2	1,2	67	Male	TCGA	1426	Dead
TCGA-22-	High	Low	L-TMB+H-ICI score	1.07179	A	C4	1,2	66	Femal	TCGA	2170	Dead
TCGA-77-	High	High	H-TMB+H-ICI score	0.49125	A	C1	1,2	81	Male	TCGA	1182	Alive
TCGA-L3-	High	Low	L-TMB+H-ICI score	0.54416	A	C2	1,2	71	Male	TCGA	392	Alive
TCGA-60-	Low	High	H-TMB+L-ICI score	-1.3734	A	C1	1,2	74	Femal	TCGA	1092	Alive
TCGA-37-	High	High	H-TMB+H-ICI score	0.84418	A	C1	3,4	51	Male	TCGA	122	Alive
TCGA-66-	High	High	H-TMB+H-ICI score	-0.2064	A	C2	1,2	63	Femal	TCGA	30	Alive
TCGA-63-	High	High	H-TMB+H-ICI score	-0.6304	A	C1	1,2	67	Male	TCGA	1602	Alive
TCGA-L3-	High	High	H-TMB+H-ICI score	-0.3222	B	C2	1,2	45	Femal	TCGA	490	Alive
TCGA-98-	High	High	H-TMB+H-ICI score	0.41348	B	C2	1,2	75	Femal	TCGA	937	Alive
TCGA-77-	Low	Low	L-TMB+L-ICI score	-0.809	B	C2	1,2	70	Male	TCGA	840	Alive
TCGA-66-	High	High	H-TMB+H-ICI score	-0.2432	A	C4	1,2	57	Male	TCGA	1217	Alive
TCGA-77-	High	Low	L-TMB+H-ICI score	-0.4665	B	C3	1,2	74	Male	TCGA	539	Dead
TCGA-63-	High	High	H-TMB+H-ICI score	0.0357	B	C2	1,2	70	Femal	TCGA	2716	Alive
TCGA-66-	High	High	H-TMB+H-ICI score	0.33627	A	C4	1,2	57	Male	TCGA	61	Alive
TCGA-77-	High	Low	L-TMB+H-ICI score	-0.185	B	C2	1,2	72	Male	TCGA	3189	Alive
TCGA-43-	High	High	H-TMB+H-ICI score	-0.0982	A	C2	3,4	61	Male	TCGA	366	Alive
TCGA-22-	Low	Low	L-TMB+L-ICI score	-5.0149	A	C1	1,2	63	Male	TCGA	1953	Dead
TCGA-66-	High	Low	L-TMB+H-ICI score	-0.5958	B	C3	1,2	65	Male	TCGA	366	Dead
TCGA-77-	Low	Low	L-TMB+L-ICI score	-0.8767	B	C1	1,2	60	Male	TCGA	1106	Alive
TCGA-66-	High	High	H-TMB+H-ICI score	1.3936	A	C3	1,2	72	Male	TCGA	699	Alive
TCGA-98-	High	High	H-TMB+H-ICI score	-0.1636	B	C4	1,2	61	Male	TCGA	933	Alive
TCGA-58-	High	High	H-TMB+H-ICI score	1.79169	B	C2	3,4	73	Male	TCGA	1723	Alive
TCGA-O2	High	High	H-TMB+H-ICI score	0.98593	A	C2	1,2	78	Male	TCGA	1006	Dead
TCGA-63-	Low	Low	L-TMB+L-ICI score	-5.0562	B	C1	1,2	67	Femal	TCGA	2073	Alive
TCGA-37-	High	High	H-TMB+H-ICI score	1.45936	A	C2	1,2	68	Male	TCGA	207	Alive
TCGA-66-	High	High	H-TMB+H-ICI score	-0.5728	B	C2	1,2	55	Femal	TCGA	516	Dead
TCGA-34-	Low	High	H-TMB+L-ICI score	-1.3585	B	C3	1,2	70	Male	TCGA	34	Dead
TCGA-NC	High	High	H-TMB+H-ICI score	0.12148	A	C2	1,2	79	Male	TCGA	2	Dead
TCGA-56-	High	High	H-TMB+H-ICI score	-0.2007	B	C2	1,2	83	Male	TCGA	601	Alive
TCGA-18-	High	High	H-TMB+H-ICI score	-0.6334	A	C1	1,2	77	Male	TCGA	2803	Alive
TCGA-56-	High	Low	L-TMB+H-ICI score	0.07517	A	C2	1,2	84	Male	TCGA	925	Alive
TCGA-33-	High	High	H-TMB+H-ICI score	0.58016	A	C2	1,2	57	Femal	TCGA	826	Dead
TCGA-43-	Low	High	H-TMB+L-ICI score	-1.4639	A	C1	3,4	62	Femal	TCGA	1223	Alive
TCGA-77-	Low	High	H-TMB+L-ICI score	-1.1533	B	C1	3,4	64	Male	TCGA	3838	Dead
TCGA-33-	High	High	H-TMB+H-ICI score	-0.0129	A	C2	1,2	73	Male	TCGA	4601	Alive
TCGA-46-	High	High	H-TMB+H-ICI score	0.6554	B	C2	1,2	81	Male	TCGA	423	Alive
TCGA-18-	High	Low	L-TMB+H-ICI score	0.77372	A	C2	3,4	73	Male	TCGA	716	Dead
TCGA-63-	High	High	H-TMB+H-ICI score	-0.0238	A	C2	1,2	63	Male	TCGA	1052	Alive
TCGA-56-	High	High	H-TMB+H-ICI score	0.38042	B	C2	1,2	80	Femal	TCGA	455	Alive
TCGA-60-	Low	Low	L-TMB+L-ICI score	-3.2891	A	C1	1,2	79	Femal	TCGA	274	Dead
TCGA-60-	High	High	H-TMB+H-ICI score	-0.0055	A	C4	1,2	62	Male	TCGA	311	Dead
TCGA-52-	High	High	H-TMB+H-ICI score	1.65902	A	C2	1,2	74	Male	TCGA	166	Dead
TCGA-34-	High	High	H-TMB+H-ICI score	0.47385	B	C4	1,2	75	Femal	TCGA	2471	Alive
TCGA-18-	High	High	H-TMB+H-ICI score	-0.3588	B	C2	1,2	63	Male	TCGA	188	Dead
TCGA-37-	High	High	H-TMB+H-ICI score	0.17877	A	C2	3,4	59	Male	TCGA	660	Alive
TCGA-85-	High	Low	L-TMB+H-ICI score	3.00445	A	C2	1,2	74	Femal	TCGA	482	Alive
TCGA-60-	High	High	H-TMB+H-ICI score	-0.7517	B	C4	1,2	60	Femal	TCGA	97	Alive
TCGA-60-	High	High	H-TMB+H-ICI score	-0.6386	B	C1	1,2	67	Femal	TCGA	2024	Alive
TCGA-94-	High	High	H-TMB+H-ICI score	-0.0612	B	C2	1,2	64	Male	TCGA	122	Alive
TCGA-22-	High	High	H-TMB+H-ICI score	0.38432	B	C3	1,2	77	Male	TCGA	1067	Dead

TCGA-18-	High	High	H-TMB+H-ICI score	-0.5268	A	C2	1,2	73	Male	TCGA	2811	Alive
TCGA-66-	High	High	H-TMB+H-ICI score	0.94749	B	C2	3,4	68	Male	TCGA	306	Dead
TCGA-21-	High	High	H-TMB+H-ICI score	0.3664	B	C3	3,4	71	Male	TCGA	965	Dead
TCGA-92-	Low	High	H-TMB+L-ICI score	-1.1749	A	C1	1,2	58	Male	TCGA	160	Alive
TCGA-63-	High	Low	L-TMB+H-ICI score	0.38335	A	C2	1,2	67	Male	TCGA	1682	Alive
TCGA-60-	High	Low	L-TMB+H-ICI score	-0.0719	A	C4	1,2	58	Male	TCGA	2820	Alive
TCGA-98-	High	Low	L-TMB+H-ICI score	-0.7812	B	C1	1,2	73	Male	TCGA	652	Alive
TCGA-37-	High	High	H-TMB+H-ICI score	2.49249	B	C2	1,2	65	Femal	TCGA	12	Alive
TCGA-22-	High	Low	L-TMB+H-ICI score	-0.1685	A	C2	1,2	65	Male	TCGA	1346	Dead
TCGA-33-	High	High	H-TMB+H-ICI score	2.90422	B	C2	1,2	76	Male	TCGA	4068	Alive
TCGA-77-	High	High	H-TMB+H-ICI score	-0.508	B	C2	3,4	60	Male	TCGA	1150	Dead
TCGA-56-	High	Low	L-TMB+H-ICI score	-0.1239	B	C1	1,2	66	Male	TCGA	428	Alive
TCGA-56-	High	High	H-TMB+H-ICI score	0.30199	A	C2	1,2	81	Male	TCGA	4	Alive
TCGA-37-	High	High	H-TMB+H-ICI score	4.2751	A	C4	1,2	56	Male	TCGA	247	Alive
TCGA-58-	High	Low	L-TMB+H-ICI score	0.32016	A	C2	1,2	64	Male	TCGA	2589	Alive
TCGA-43-	High	High	H-TMB+H-ICI score	0.52838	B	C2	1,2	70	Male	TCGA	699	Alive
TCGA-77-	High	High	H-TMB+H-ICI score	0.353	A	C2	3,4	66	Male	TCGA	678	Dead
TCGA-39-	High	High	H-TMB+H-ICI score	-0.7682	B	C4	1,2	76	Femal	TCGA	1841	Alive
TCGA-90-	High	High	H-TMB+H-ICI score	0.29603	A	C4	1,2	61	Femal	TCGA	322	Alive
TCGA-68-	High	High	H-TMB+H-ICI score	-0.5567	A	C2	1,2	60	Femal	TCGA	83	Alive
TCGA-66-	High	High	H-TMB+H-ICI score	0.52135	A	C1	3,4	62	Male	TCGA	61	Alive
TCGA-66-	High	High	H-TMB+H-ICI score	-0.1088	B	C4	1,2	60	Male	TCGA	578	Alive
TCGA-85-	High	High	H-TMB+H-ICI score	0.11718	B	C1	1,2	59	Male	TCGA	1259	Alive
TCGA-56-	High	High	H-TMB+H-ICI score	-0.6863	B	C2	1,2	73	Femal	TCGA	106	Alive
TCGA-56-	High	High	H-TMB+H-ICI score	0.94873	B	C2	1,2	79	Male	TCGA	608	Alive
TCGA-18-	High	High	H-TMB+H-ICI score	0.64346	A	C4	1,2	65	Male	TCGA	2645	Alive
TCGA-51-	High	High	H-TMB+H-ICI score	0.34998	A	C2	1,2	72	Femal	TCGA	1856	Dead
TCGA-NC	High	High	H-TMB+H-ICI score	0.98637	A	C4	1,2	73	Male	TCGA	88	Dead
TCGA-77-	High	Low	L-TMB+H-ICI score	0.18847	B	C4	3,4	75	Male	TCGA	4026	Alive
TCGA-96-	High	Low	L-TMB+H-ICI score	0.11201	A	C1	1,2	73	Male	TCGA	1736	Alive
TCGA-NC	High	High	H-TMB+H-ICI score	0.47549	A	C2	1,2	76	Male	TCGA	1212	Alive
TCGA-33-	Low	Low	L-TMB+L-ICI score	-0.8497	A	C4	1,2	62	Femal	TCGA	47	Dead
TCGA-85-	High	High	H-TMB+H-ICI score	0.03306	B	C2	1,2	67	Male	TCGA	600	Alive
TCGA-60-	High	Low	L-TMB+H-ICI score	-0.4365	A	C2	1,2	64	Femal	TCGA	1260	Alive
TCGA-33-	High	High	H-TMB+H-ICI score	-0.2343	A	C2	1,2	68	Male	TCGA	3924	Alive
TCGA-NC	High	High	H-TMB+H-ICI score	3.22818	A	C4	1,2	59	Male	TCGA	418	Dead
TCGA-21-	High	High	H-TMB+H-ICI score	-0.0814	A	C4	3,4	65	Male	TCGA	329	Dead
TCGA-85-	High	Low	L-TMB+H-ICI score	0.43025	B	C2	1,2	66	Male	TCGA	468	Alive
TCGA-34-	High	High	H-TMB+H-ICI score	-0.3012	B	C3	1,2	73	Femal	TCGA	1541	Alive
TCGA-77-	Low	High	H-TMB+L-ICI score	-0.8082	A	C2	3,4	68	Male	TCGA	2023	Alive
TCGA-56-	High	High	H-TMB+H-ICI score	1.41832	B	C2	1,2	59	Male	TCGA	302	Alive
TCGA-33-	High	High	H-TMB+H-ICI score	-0.6904	B	C2	1,2	60	Male	TCGA	143	Dead
TCGA-39-	High	High	H-TMB+H-ICI score	-0.0711	A	C2	1,2	73	Male	TCGA	3108	Alive
TCGA-56-	High	Low	L-TMB+H-ICI score	-0.5568	B	C1	1,2	76	Femal	TCGA	41	Alive
TCGA-56-	High	High	H-TMB+H-ICI score	1.27486	A	C1	1,2	71	Male	TCGA	692	Alive
TCGA-77-	High	High	H-TMB+H-ICI score	-0.1594	B	C2	1,2	69	Male	TCGA	708	Dead
TCGA-85-	High	High	H-TMB+H-ICI score	0.83906	A	C2	1,2	57	Male	TCGA	493	Alive
TCGA-77-	High	High	H-TMB+H-ICI score	0.15204	B	C2	1,2	70	Male	TCGA	833	Alive
TCGA-85-	High	High	H-TMB+H-ICI score	0.35037	B	C2	1,2	63	Male	TCGA	377	Alive
TCGA-XC	High	Low	L-TMB+H-ICI score	-0.0355	B	C4	1,2	77	Femal	TCGA	6	Dead
TCGA-66-	High	High	H-TMB+H-ICI score	0.4994	A	C1	1,2	71	Male	TCGA	365	Dead
TCGA-68-	High	High	H-TMB+H-ICI score	0.721	B	C1	3,4	73	Femal	TCGA	492	Alive
TCGA-77-	High	Low	L-TMB+H-ICI score	0.91597	B	C2	1,2	63	Male	TCGA	180	Dead
TCGA-90-	High	High	H-TMB+H-ICI score	2.12303	A	C1	1,2	53	Male	TCGA	688	Alive
TCGA-98-	High	High	H-TMB+H-ICI score	0.16393	A	C1	3,4	70	Male	TCGA	649	Alive
TCGA-34-	High	Low	L-TMB+H-ICI score	-0.0202	B	C2	1,2	71	Femal	TCGA	804	Alive
TCGA-56-	Low	Low	L-TMB+L-ICI score	-2.3735	B	C1	1,2	74	Male	TCGA	378	Alive
TCGA-66-	High	High	H-TMB+H-ICI score	0.24773	B	C4	1,2	68	Femal	TCGA	790	Alive
TCGA-39-	High	High	H-TMB+H-ICI score	-0.3773	B	C2	1,2	65	Male	TCGA	1690	Alive
TCGA-56-	Low	High	H-TMB+L-ICI score	-0.9813	B	C1	1,2	77	Femal	TCGA	666	Alive
TCGA-NK	High	High	H-TMB+H-ICI score	1.45232	A	C2	1,2	70	Male	TCGA	1997	Alive
TCGA-22-	High	High	H-TMB+H-ICI score	1.41622	B	C1	1,2	65	Male	TCGA	822	Dead
TCGA-39-	High	High	H-TMB+H-ICI score	-0.0427	A	C4	1,2	44	Male	TCGA	3850	Alive
TCGA-63-	High	High	H-TMB+H-ICI score	0.24391	A	C1	1,2	69	Femal	TCGA	456	Dead
TCGA-33-	High	High	H-TMB+H-ICI score	-0.2853	A	C2	3,4	66	Male	TCGA	2979	Dead
TCGA-63-	High	High	H-TMB+H-ICI score	0.40281	B	C1	1,2	74	Male	TCGA	498	Alive
TCGA-18-	High	High	H-TMB+H-ICI score	1.32099	A	C4	1,2	83	Male	TCGA	973	Alive
TCGA-66-	High	High	H-TMB+H-ICI score	-0.4252	B	C2	1,2	69	Male	TCGA	31	Alive
TCGA-63-	High	High	H-TMB+H-ICI score	0.38364	A	C4	1,2	67	Male	TCGA	2133	Alive
TCGA-56-	Low	High	H-TMB+L-ICI score	-1.2661	B	C2	1,2	79	Male	TCGA	517	Alive
TCGA-18-	High	Low	L-TMB+H-ICI score	-0.489	A	C2	1,2	77	Femal	TCGA	2304	Alive
TCGA-NK	High	Low	L-TMB+H-ICI score	1.66043	B	C2	1,2	57	Male	TCGA	511	Alive
TCGA-85-	High	High	H-TMB+H-ICI score	-0.5015	B	C2	3,4	57	Male	TCGA	195	Alive
TCGA-22-	Low	High	H-TMB+L-ICI score	-1.6324	A	C4	1,2	73	Femal	TCGA	1161	Dead
TCGA-56-	High	High	H-TMB+H-ICI score	-0.4899	A	C4	1,2	76	Femal	TCGA	570	Alive
TCGA-56-	Low	High	H-TMB+L-ICI score	-1.4834	B	C1	1,2	66	Femal	TCGA	3	Alive
TCGA-92-	Low	Low	L-TMB+L-ICI score	-0.822	B	C3	1,2	71	Male	TCGA	106	Alive
TCGA-85-	Low	High	H-TMB+L-ICI score	-1.556	B	C1	3,4	72	Male	TCGA	94	Alive
TCGA-85-	High	High	H-TMB+H-ICI score	0.53781	A	C2	1,2	68	Male	TCGA	927	Alive
TCGA-77-	High	High	H-TMB+H-ICI score	0.43614	A	C4	1,2	79	Male	TCGA	899	Dead
TCGA-22-	High	High	H-TMB+H-ICI score	-0.5772	A	C1	3,4	57	Male	TCGA	734	Dead
TCGA-33-	High	High	H-TMB+H-ICI score	1.631	B	C2	1,2	63	Femal	TCGA	1656	Dead
TCGA-56-	High	High	H-TMB+H-ICI score	1.28795	A	C2	1,2	55	Male	TCGA	585	Alive
TCGA-21-	High	High	H-TMB+H-ICI score	0.11865	B	C1	1,2	54	Femal	TCGA	1852	Alive
TCGA-6A-	High	High	H-TMB+H-ICI score	0.79843	A	C2	1,2	73	Femal	TCGA	0	Dead
TCGA-63-	High	Low	L-TMB+H-ICI score	0.40744	B	C2	1,2	67	Femal	TCGA	0	Alive
TCGA-39-	Low	Low	L-TMB+L-ICI score	-3.3198	B	C1	1,2	76	Male	TCGA	1679	Dead
TCGA-43-	High	High	H-TMB+H-ICI score	-0.3362	A	C3	1,2	85	Male	TCGA	166	Dead
TCGA-98-	High	Low	L-TMB+H-ICI score	-0.3541	B	C3	3,4	56	Femal	TCGA	84	Dead
TCGA-77-	High	High	H-TMB+H-ICI score	0.17912	B	C2	1,2	64	Male	TCGA	15	Alive
TCGA-92-	High	High	H-TMB+H-ICI score	-0.2529	B	C1	1,2	45	Femal	TCGA	82	Alive
TCGA-56-	Low	High	H-TMB+L-ICI score	-0.917	B	C3	1,2	68	Male	TCGA	55	Alive
TCGA-70-	Low	High	H-TMB+L-ICI score	-0.8535	B	C1	3,4	47	Male	TCGA	367	Alive
TCGA-43-	Low	Low	L-TMB+L-ICI score	-3.7196	B	C4	1,2	76	Femal	TCGA	432	Alive
TCGA-66-	High	High	H-TMB+H-ICI score	-0.3396	A	C1	1,2	79	Male	TCGA	700	Alive
TCGA-85-	High	High	H-TMB+H-ICI score	0.72172	A	C2	1,2	59	Femal	TCGA	42	Alive
TCGA-58-	Low	Low	L-TMB+L-ICI score	-1.9507	B	C4	1,2	60	Male	TCGA	403	Dead
TCGA-85-	High	High	H-TMB+H-ICI score	1.30518	A	C2	3,4	67	Male	TCGA	161	Alive

TCGA-21-	Low	High	H-TMB+L-ICI score	-0.9383	A	C2	1,2	64	Male	TCGA	1032	Alive
TCGA-33-	High	High	H-TMB+H-ICI score	0.00757	A	C2	1,2	65	Femal	TCGA	1344	Dead
TCGA-56-	High	High	H-TMB+H-ICI score	0.33879	B	C1	3,4	66	Femal	TCGA	315	Alive
TCGA-34-	High	High	H-TMB+H-ICI score	0.45895	A	C2	1,2	76	Femal	TCGA	1874	Dead
TCGA-60-	High	High	H-TMB+H-ICI score	1.69595	A	C2	1,2	56	Male	TCGA	358	Dead
TCGA-77-	High	High	H-TMB+H-ICI score	-0.51	B	C2	1,2	77	Femal	TCGA	1992	Alive
TCGA-66-	High	High	H-TMB+H-ICI score	-0.7683	B	C1	3,4	66	Male	TCGA	153	Dead
TCGA-68-	High	Low	L-TMB+H-ICI score	-0.5574	B	C2	3,4	84	Male	TCGA	202	Alive
TCGA-94-	High	Low	L-TMB+H-ICI score	0.02345	B	C2	1,2	73	Male	TCGA	5	Dead
TCGA-56-	High	High	H-TMB+H-ICI score	0.78742	B	C2	1,2	72	Femal	TCGA	8	Alive
TCGA-22-	Low	High	H-TMB+L-ICI score	-1.4481	A	C4	1,2	73	Femal	TCGA	358	Dead
TCGA-77-	High	High	H-TMB+H-ICI score	-0.0033	B	C3	1,2	69	Male	TCGA	4765	Alive
TCGA-21-	High	High	H-TMB+H-ICI score	0.18013	A	C2	1,2	66	Male	TCGA	3724	Alive
TCGA-98-	High	High	H-TMB+H-ICI score	-0.0848	A	C4	1,2	64	Male	TCGA	565	Alive
TCGA-34-	Low	Low	L-TMB+L-ICI score	-1.0752	B	C3	1,2	60	Male	TCGA	276	Dead
TCGA-21-	High	High	H-TMB+H-ICI score	12.5014	B	C2	1,2	68	Femal	TCGA	962	Dead
TCGA-60-	High	High	H-TMB+H-ICI score	0.19911	B	C3	1,2	64	Femal	TCGA	2447	Alive
TCGA-96-	Low	High	H-TMB+L-ICI score	-0.8015	A	C2	1,2	65	Male	TCGA	589	Alive
TCGA-66-	Low	High	H-TMB+L-ICI score	-1.5136	A	C1	1,2	56	Male	TCGA	699	Alive
TCGA-56-	High	Low	L-TMB+H-ICI score	1.43424	A	C2	1,2	84	Male	TCGA	420	Alive
TCGA-22-	High	High	H-TMB+H-ICI score	-0.5409	B	C2	1,2	78	Male	TCGA	1933	Dead
TCGA-21-	High	Low	L-TMB+H-ICI score	0.68245	B	C2	1,2	77	Male	TCGA	979	Alive
TCGA-85-	Low	Low	L-TMB+L-ICI score	-1.9472	B	C1	1,2	57	Male	TCGA	579	Alive
TCGA-21-	High	High	H-TMB+H-ICI score	0.95576	B	C2	1,2	76	Male	TCGA	2680	Dead
TCGA-46-	Low	High	H-TMB+L-ICI score	-1.526	B	C2	1,2	59	Femal	TCGA	405	Alive
TCGA-39-	Low	Low	L-TMB+L-ICI score	-1.311	B	C4	1,2	70	Male	TCGA	2086	Dead
TCGA-90-	Low	High	H-TMB+L-ICI score	-1.0205	B	C1	1,2	64	Male	TCGA	758	Alive
TCGA-39-	High	High	H-TMB+H-ICI score	-0.7657	B	C4	1,2	70	Femal	TCGA	4053	Alive
TCGA-39-	High	Low	L-TMB+H-ICI score	-0.3183	B	C2	1,2	70	Male	TCGA	3387	Alive
TCGA-98-	High	High	H-TMB+H-ICI score	-0.1028	B	C2	1,2	63	Male	TCGA	173	Alive
TCGA-66-	Low	Low	L-TMB+L-ICI score	-0.8836	A	C2	1,2	67	Male	TCGA	121	Alive
TCGA-37-	High	High	H-TMB+H-ICI score	0.21442	A	C2	3,4	64	Male	TCGA	103	Alive
TCGA-56-	Low	Low	L-TMB+L-ICI score	-1.6131	B	C1	1,2	74	Male	TCGA	397	Alive
TCGA-63-	High	High	H-TMB+H-ICI score	0.60759	B	C2	1,2	62	Male	TCGA	3123	Alive
TCGA-60-	High	High	H-TMB+H-ICI score	-0.1688	A	C2	1,2	66	Male	TCGA	908	Alive
TCGA-85-	High	Low	L-TMB+H-ICI score	0.70518	B	C1	1,2	62	Male	TCGA	765	Alive
TCGA-56-	High	High	H-TMB+H-ICI score	1.34681	B	C2	3,4	66	Male	TCGA	442	Alive
TCGA-43-	High	High	H-TMB+H-ICI score	-0.0926	B	C1	1,2	72	Femal	TCGA	407	Alive
TCGA-18-	High	High	H-TMB+H-ICI score	-0.6872	B	C2	1,2	57	Male	TCGA	1519	Alive
TCGA-77-	High	High	H-TMB+H-ICI score	-0.1891	B	C3	1,2	65	Male	TCGA	3253	Dead
TCGA-60-	High	High	H-TMB+H-ICI score	0.02262	A	C2	1,2	83	Femal	TCGA	1297	Alive
TCGA-22-	High	High	H-TMB+H-ICI score	0.16148	B	C3	1,2	79	Male	TCGA	24	Dead
TCGA-66-	High	High	H-TMB+H-ICI score	0.3684	A	C2	1,2	65	Femal	TCGA	1338	Alive
TCGA-18-	High	High	H-TMB+H-ICI score	-0.3917	A	C1	1,2	67	Male	TCGA	371	Dead
TCGA-77-	High	Low	L-TMB+H-ICI score	-0.6167	B	C1	1,2	90	Male	TCGA	0	Dead
TCGA-21-	High	High	H-TMB+H-ICI score	-0.1952	A	C4	3,4	60	Femal	TCGA	3636	Alive
TCGA-43-	High	High	H-TMB+H-ICI score	2.11877	A	C4	1,2	67	Femal	TCGA	296	Alive
TCGA-85-	High	Low	L-TMB+H-ICI score	-0.1316	B	C3	1,2	48	Male	TCGA	952	Alive
TCGA-85-	Low	High	H-TMB+L-ICI score	-0.8902	B	C3	1,2	50	Male	TCGA	35	Alive
TCGA-NC	High	High	H-TMB+H-ICI score	0.23407	B	C2	3,4	69	Male	TCGA	804	Alive
TCGA-58-	High	High	H-TMB+H-ICI score	1.08689	A	C4	1,2	68	Femal	TCGA	1058	Alive
TCGA-66-	High	High	H-TMB+H-ICI score	-0.0418	B	C1	1,2	72	Male	TCGA	61	Alive
TCGA-LA-	High	Low	L-TMB+H-ICI score	1.10944	B	C2	3,4	71	Male	TCGA	408	Alive
TCGA-77-	High	Low	L-TMB+H-ICI score	-0.2522	B	C1	1,2	68	Male	TCGA	198	Dead
TCGA-85-	High	High	H-TMB+H-ICI score	-0.3251	A	C2	1,2	53	Male	TCGA	995	Alive
TCGA-22-	Low	Low	L-TMB+L-ICI score	-2.0872	A	C4	1,2	74	Male	TCGA	445	Dead
TCGA-77-	High	High	H-TMB+H-ICI score	-0.2587	A	C2	1,2	69	Femal	TCGA	632	Dead
TCGA-22-	High	High	H-TMB+H-ICI score	0.01761	B	C2	3,4	73	Femal	TCGA	1057	Dead
TCGA-66-	High	High	H-TMB+H-ICI score	-0.2994	A	C4	3,4	64	Male	TCGA	1645	Alive
TCGA-22-	High	High	H-TMB+H-ICI score	0.63158	A	C2	1,2	62	Male	TCGA	1485	Dead
TCGA-77-	High	Low	L-TMB+H-ICI score	-0.1276	B	C2	1,2	76	Male	TCGA	1280	Alive
TCGA-56-	High	High	H-TMB+H-ICI score	0.30311	B	C2	1,2	60	Male	TCGA	562	Alive
TCGA-85-	High	High	H-TMB+H-ICI score	-0.6905	A	C1	3,4	73	Male	TCGA	1001	Alive
TCGA-66-	High	High	H-TMB+H-ICI score	-0.6977	A	C4	1,2	71	Male	TCGA	639	Alive
TCGA-85-	High	High	H-TMB+H-ICI score	3.38559	A	C1	1,2	46	Male	TCGA	576	Alive
TCGA-39-	High	High	H-TMB+H-ICI score	-0.7515	B	C3	1,2	73	Femal	TCGA	1107	Dead
TCGA-85-	High	Low	L-TMB+H-ICI score	-0.6457	B	C2	1,2	53	Male	TCGA	734	Alive
TCGA-43-	High	Low	L-TMB+H-ICI score	0.06661	B	C2	1,2	52	Male	TCGA	1190	Alive
TCGA-NK	High	Low	L-TMB+H-ICI score	-0.2506	A	C2	1,2	73	Male	TCGA	111	Alive
TCGA-39-	Low	Low	L-TMB+L-ICI score	-0.9224	A	C4	1,2	72	Femal	TCGA	2080	Alive
TCGA-43-	High	Low	L-TMB+H-ICI score	-0.5703	B	C1	1,2	69	Femal	TCGA	757	Alive
TCGA-18-	Low	Low	L-TMB+L-ICI score	-1.5888	B	C3	1,2	64	Male	TCGA	85	Dead
TCGA-NC	High	High	H-TMB+H-ICI score	0.2996	A	C2	3,4	74	Male	TCGA	138	Dead
TCGA-39-	Low	Low	L-TMB+L-ICI score	-0.8604	A	C4	3,4	67	Male	TCGA	740	Dead
TCGA-43-	High	High	H-TMB+H-ICI score	-0.5911	A	C4	1,2	78	Male	TCGA	559	Alive
TCGA-22-	High	High	H-TMB+H-ICI score	-0.3795	B	C2	1,2	80	Femal	TCGA	429	Dead
TCGA-34-	High	High	H-TMB+H-ICI score	-0.1319	A	C1	3,4	62	Femal	TCGA	1180	Alive
TCGA-60-	Low	Low	L-TMB+L-ICI score	-3.2629	B	C1	1,2	69	Male	TCGA	1505	Alive
TCGA-85-	High	High	H-TMB+H-ICI score	0.50802	B	C2	1,2	46	Male	TCGA	465	Alive
TCGA-NC	High	High	H-TMB+H-ICI score	0.632	A	C2	1,2	68	Femal	TCGA	1743	Alive
TCGA-18-	High	High	H-TMB+H-ICI score	0.54158	B	C1	1,2	74	Male	TCGA	3747	Alive
TCGA-56-	High	High	H-TMB+H-ICI score	1.10083	A	C4	1,2	55	Femal	TCGA	818	Alive
TCGA-85-	High	High	H-TMB+H-ICI score	0.7273	B	C3	1,2	70	Male	TCGA	402	Alive
TCGA-18-	High	High	H-TMB+H-ICI score	2.17495	A	C4	1,2	81	Male	TCGA	146	Dead
TCGA-22-	Low	Low	L-TMB+L-ICI score	-2.3024	B	C1	1,2	76	Male	TCGA	454	Dead
TCGA-66-	High	Low	L-TMB+H-ICI score	-0.2786	A	C4	3,4	68	Femal	TCGA	578	Alive
TCGA-34-	High	High	H-TMB+H-ICI score	0.62373	A	C1	3,4	75	Male	TCGA	1834	Alive
TCGA-34-	High	High	H-TMB+H-ICI score	-0.0619	B	C2	3,4	67	Male	TCGA	123	Dead
TCGA-66-	High	Low	L-TMB+H-ICI score	-0.523	A	C4	1,2	64	Male	TCGA	61	Alive
TCGA-85-	High	High	H-TMB+H-ICI score	-0.6967	A	C2	3,4	70	Male	TCGA	307	Alive
TCGA-92-	High	High	H-TMB+H-ICI score	-0.4992	A	C4	1,2	74	Femal	TCGA	70	Alive
TCGA-46-	Low	High	H-TMB+L-ICI score	-2.2234	B	C1	1,2	62	Femal	TCGA	370	Alive
TCGA-77-	Low	Low	L-TMB+L-ICI score	-2.4316	A	C1	1,2	72	Male	TCGA	383	Dead
TCGA-94-	High	High	H-TMB+H-ICI score	-0.2255	B	C2	1,2	61	Male	TCGA	491	Alive
TCGA-18-	Low	Low	L-TMB+L-ICI score	-1.4283	B	C3	1,2	72	Male	TCGA	136	Dead
TCGA-77-	Low	High	H-TMB+L-ICI score	-1.6228	B	C2	1,2	72	Male	TCGA	3166	Alive
TCGA-21-	High	Low	L-TMB+H-ICI score	1.06673	A	C2	1,2	77	Male	TCGA	474	Dead
TCGA-18-	Low	High	H-TMB+L-ICI score	-1.6212	A	C2	3,4	63	Femal	TCGA	3576	Alive

TCGA-66- High	High	H-TMB+H-ICI score	-0.1397	B	C2	3,4	70	Male	TCGA	641	Alive
TCGA-56- High	High	H-TMB+H-ICI score	0.34449	B	C2	1,2	58	Male	TCGA	881	Dead
TCGA-77- High	High	H-TMB+H-ICI score	0.24346	A	C2	1,2	63	Male	TCGA	4570	Alive
TCGA-22- High	Low	L-TMB+H-ICI score	-0.5119	B	C2	1,2	81	Male	TCGA	357	Dead
TCGA-NK High	Low	L-TMB+H-ICI score	0.27315	A	C2	3,4	66	Male	TCGA	13	Alive
TCGA-22- Low	High	H-TMB+L-ICI score	-2.5396	B	C1	1,2	64	Male	TCGA	1912	Dead
TCGA-O2 High	Low	L-TMB+H-ICI score	0.56683	A	C2	1,2	63	Male	TCGA	261	Dead
TCGA-52- High	High	H-TMB+H-ICI score	0.04172	A	C4	1,2	68	Male	TCGA	835	Dead
TCGA-46- High	High	H-TMB+H-ICI score	-0.184	B	C2	1,2	71	Male	TCGA	324	Alive
TCGA-58- High	High	H-TMB+H-ICI score	-0.1013	B	C2	3,4	57	Femal	TCGA	2167	Alive
TCGA-85- High	Low	L-TMB+H-ICI score	0.20795	B	C2	1,2	47	Male	TCGA	826	Alive
TCGA-18- High	High	H-TMB+H-ICI score	-0.7378	B	C3	3,4	65	Male	TCGA	1097	Alive
TCGA-60- High	High	H-TMB+H-ICI score	0.06185	B	C3	3,4	47	Male	TCGA	717	Alive
TCGA-94- High	High	H-TMB+H-ICI score	-0.1052	B	C2	1,2	62	Male	TCGA	538	Alive
TCGA-43- High	High	H-TMB+H-ICI score	-0.1273	B	C2	1,2	73	Male	TCGA	358	Alive
TCGA-34- High	Low	L-TMB+H-ICI score	0.63726	A	C1	1,2	79	Male	TCGA	515	Alive
TCGA-77- High	Low	L-TMB+H-ICI score	-0.7455	B	C3	1,2	64	Male	TCGA	5	Dead
TCGA-56- High	High	H-TMB+H-ICI score	0.25474	B	C2	1,2	75	Male	TCGA	532	Alive
TCGA-85- High	High	H-TMB+H-ICI score	-0.2184	B	C3	1,2	84	Male	TCGA	604	Alive
TCGA-66- High	Low	L-TMB+H-ICI score	-0.2521	B	C2	1,2	58	Male	TCGA	913	Alive
TCGA-98- High	High	H-TMB+H-ICI score	0.3531	A	C2	1,2	70	Male	TCGA	552	Alive
TCGA-22- Low	High	H-TMB+L-ICI score	-3.4668	B	C1	3,4	60	Femal	TCGA	1470	Dead
TCGA-58- High	High	H-TMB+H-ICI score	-0.0557	A	C1	3,4	75	Male	TCGA	1	Dead
TCGA-22- High	High	H-TMB+H-ICI score	-0.5907	A	C2	1,2	72	Femal	TCGA	2409	Dead
TCGA-66- High	High	H-TMB+H-ICI score	2.05383	A	C2	3,4	67	Male	TCGA	61	Alive
TCGA-21- High	High	H-TMB+H-ICI score	0.01745	A	C4	1,2	61	Male	TCGA	3644	Alive
TCGA-58- High	Low	L-TMB+H-ICI score	-0.3007	B	C2	1,2	70	Male	TCGA	501	Dead
TCGA-60- High	High	H-TMB+H-ICI score	-0.4783	B	C1	1,2	64	Male	TCGA	1731	Alive
TCGA-22- Low	High	H-TMB+L-ICI score	-1.0845	B	C2	1,2	64	Male	TCGA	2625	Alive
TCGA-37- High	High	H-TMB+H-ICI score	-0.3602	A	C2	3,4	63	Male	TCGA	238	Alive
TCGA-63- High	High	H-TMB+H-ICI score	-0.4483	A	C2	1,2	48	Male	TCGA	0	Dead
TCGA-66- High	High	H-TMB+H-ICI score	-0.7608	A	C1	3,4	75	Male	TCGA	215	Dead
TCGA-LA- High	High	H-TMB+H-ICI score	-0.1563	A	C2	1,2	68	Male	TCGA	401	Alive
TCGA-66- High	High	H-TMB+H-ICI score	-0.623	A	C4	3,4	66	Male	TCGA	762	Alive
TCGA-22- Low	High	H-TMB+L-ICI score	-2.0586	B	C1	1,2	81	Male	TCGA	291	Dead
TCGA-66- High	High	H-TMB+H-ICI score	-0.113	A	C4	1,2	69	Male	TCGA	92	Dead
TCGA-66- High	High	H-TMB+H-ICI score	-0.2162	B	C1	1,2	63	Male	TCGA	28	Alive
TCGA-77- High	Low	L-TMB+H-ICI score	-0.2808	A	C2	3,4	64	Male	TCGA	1655	Dead
TCGA-37- High	High	H-TMB+H-ICI score	0.6784	A	C2	1,2	53	Male	TCGA	1143	Alive
TCGA-77- High	Low	L-TMB+H-ICI score	-0.1033	B	C2	1,2	68	Male	TCGA	1695	Alive
TCGA-33- High	High	H-TMB+H-ICI score	-0.0967	B	C2	1,2	59	Femal	TCGA	1114	Dead
TCGA-58- High	High	H-TMB+H-ICI score	-0.2024	A	C2	1,2	52	Male	TCGA	910	Alive
TCGA-33- High	High	H-TMB+H-ICI score	-0.0358	B	C2	1,2	83	Male	TCGA	2249	Dead
TCGA-63- High	High	H-TMB+H-ICI score	0.48889	B	C1	1,2	78	Male	TCGA	2381	Alive
TCGA-43- High	High	H-TMB+H-ICI score	-0.3283	B	C2	1,2	66	Male	TCGA	353	Alive
TCGA-43- High	High	H-TMB+H-ICI score	-0.3221	B	C2	1,2	59	Femal	TCGA	653	Alive
TCGA-22- High	Low	L-TMB+H-ICI score	0.04231	B	C2	1,2	75	Male	TCGA	587	Dead
TCGA-58- High	Low	L-TMB+H-ICI score	0.04899	A	C2	3,4	59	Male	TCGA	1045	Dead
TCGA-77- High	High	H-TMB+H-ICI score	1.03213	A	C4	1,2	58	Male	TCGA	990	Alive
TCGA-18- Low	High	H-TMB+L-ICI score	-1.8989	B	C1	1,2	74	Male	TCGA	4694	Alive
TCGA-85- High	High	H-TMB+H-ICI score	0.07552	A	C4	1,2	65	Male	TCGA	921	Alive
TCGA-22- Low	High	H-TMB+L-ICI score	-1.2318	B	C1	1,2	73	Male	TCGA	399	Dead
TCGA-56- High	High	H-TMB+H-ICI score	-0.3689	B	C2	1,2	73	Male	TCGA	198	Alive
TCGA-39- High	Low	L-TMB+H-ICI score	-0.2239	A	C3	1,2	76	Male	TCGA	544	Dead
TCGA-J1- Low	High	H-TMB+L-ICI score	-1.5999	B	C2	1,2	70	Male	TCGA	581	Alive
TCGA-92- High	High	H-TMB+H-ICI score	-0.2234	A	C2	3,4	52	Male	TCGA	122	Alive
TCGA-46- High	High	H-TMB+H-ICI score	-0.5806	A	C1	1,2	57	Male	TCGA	135	Alive
TCGA-43- High	High	H-TMB+H-ICI score	0.0668	B	C2	1,2	55	Femal	TCGA	89	Dead
TCGA-NC High	High	H-TMB+H-ICI score	0.91726	A	C2	3,4	70	Femal	TCGA	1336	Alive
TCGA-22- High	Low	L-TMB+H-ICI score	-0.7634	B	C1	1,2	78	Femal	TCGA	974	Dead
TCGA-60- Low	High	H-TMB+L-ICI score	-1.245	A	C4	1,2	66	Femal	TCGA	1531	Alive
TCGA-33- High	High	H-TMB+H-ICI score	0.16256	B	C2	3,4	80	Male	TCGA	1927	Alive
TCGA-63- High	Low	L-TMB+H-ICI score	1.58834	B	C1	1,2	69	Male	TCGA	1100	Alive
TCGA-85- High	High	H-TMB+H-ICI score	-0.6947	B	C3	1,2	56	Femal	TCGA	1038	Alive
TCGA-NC High	High	H-TMB+H-ICI score	0.97354	A	C2	1,2	58	Femal	TCGA	128	Alive
TCGA-43- High	High	H-TMB+H-ICI score	2.04207	B	C1	1,2	71	Male	TCGA	1007	Alive
TCGA-21- High	High	H-TMB+H-ICI score	-0.2388	A	C2	1,2	75	Male	TCGA	3016	Alive
TCGA-98- High	High	H-TMB+H-ICI score	1.64198	B	C2	1,2	69	Male	TCGA	61	Dead
TCGA-85- High	Low	L-TMB+H-ICI score	-0.1122	A	C2	1,2	60	Male	TCGA	932	Alive
TCGA-68- High	High	H-TMB+H-ICI score	1.03185	A	C2	1,2	74	Femal	TCGA	448	Alive
TCGA-66- High	High	H-TMB+H-ICI score	-0.278	B	C2	3,4	54	Male	TCGA	31	Alive
TCGA-NC High	High	H-TMB+H-ICI score	0.83484	A	C2	1,2	75	Femal	TCGA	1244	Alive
TCGA-NC High	High	H-TMB+H-ICI score	2.082	A	C4	1,2	60	Male	TCGA	2336	Alive
TCGA-98- High	High	H-TMB+H-ICI score	2.7239	B	C1	1,2	68	Male	TCGA	645	Alive
TCGA-66- Low	High	H-TMB+L-ICI score	-1.6518	B	C2	3,4	67	Male	TCGA	759	Alive
TCGA-77- High	Low	L-TMB+H-ICI score	0.24802	B	C2	1,2	67	Male	TCGA	340	Dead
TCGA-56- High	High	H-TMB+H-ICI score	0.36051	B	C2	1,2	63	Male	TCGA	481	Alive
TCGA-79- High	High	H-TMB+H-ICI score	-0.4087	B	C2	3,4	67	Male	TCGA	0	Alive
TCGA-63- High	High	H-TMB+H-ICI score	-0.1611	B	C3	1,2	54	Male	TCGA	1824	Alive
TCGA-56- Low	High	H-TMB+L-ICI score	-1.9323	B	C1	1,2	67	Male	TCGA	0	Alive
TCGA-51- High	High	H-TMB+H-ICI score	0.21641	B	C2	1,2	55	Male	TCGA	911	Alive
TCGA-34- High	High	H-TMB+H-ICI score	1.51635	A	C2	1,2	70	Femal	TCGA	1361	Alive
TCGA-85- High	High	H-TMB+H-ICI score	0.15708	B	C3	1,2	49	Male	TCGA	1160	Alive
TCGA-77- High	High	H-TMB+H-ICI score	0.26803	B	C2	1,2	67	Male	TCGA	1841	Alive
TCGA-77- High	High	H-TMB+H-ICI score	0.76426	A	C2	3,4	74	Male	TCGA	2284	Dead
TCGA-85- High	High	H-TMB+H-ICI score	-0.0731	A	C2	1,2	52	Femal	TCGA	1113	Alive
TCGA-56- High	Low	L-TMB+H-ICI score	0.90845	A	C1	1,2	75	Femal	TCGA	640	Alive
TCGA-21- High	High	H-TMB+H-ICI score	1.05567	B	C2	1,2	75	Male	TCGA	1315	Dead
TCGA-46- High	Low	L-TMB+H-ICI score	0.01736	A	C1	1,2	76	Male	TCGA	396	Alive
TCGA-56- High	Low	L-TMB+H-ICI score	-0.4151	A	C2	1,2	69	Male	TCGA	555	Alive
TCGA-68- High	High	H-TMB+H-ICI score	0.04515	B	C3	1,2	74	Male	TCGA	211	Alive
TCGA-56- High	High	H-TMB+H-ICI score	1.50135	B	C4	3,4	67	Male	TCGA	637	Alive
TCGA-18- High	Low	L-TMB+H-ICI score	-0.3029	A	C2	1,2	52	Male	TCGA	345	Dead
TCGA-37- High	High	H-TMB+H-ICI score	-0.6077	A	C4	3,4	61	Femal	TCGA	227	Alive
TCGA-22- High	High	H-TMB+H-ICI score	0.03492	B	C3	1,2	74	Male	TCGA	573	Dead
TCGA-85- High	High	H-TMB+H-ICI score	0.2221	B	C2	1,2	74	Male	TCGA	942	Alive
TCGA-60- High	Low	L-TMB+H-ICI score	-0.0763	B	C1	1,2	74	Male	TCGA	816	Alive
TCGA-77- High	High	H-TMB+H-ICI score	-0.0658	A	C2	1,2	75	Male	TCGA	1423	Dead

TCGA-33-	High	High	H-TMB+H-ICI score	0.09179	B	C2	1,2		60	Male	TCGA	3600	Dead
TCGA-43-	High	Low	L-TMB+H-ICI score	-0.3925	B	C2	1,2		68	Femal	TCGA	236	Alive
TCGA-22-	High	High	H-TMB+H-ICI score	1.19022	B	C2	1,2		70	Male	TCGA	671	Alive
TCGA-90-	High	High	H-TMB+H-ICI score	-0.3304	B	C2	1,2		55	Male	TCGA	358	Alive
TCGA-21-	Low	High	H-TMB+L-ICI score	-1.4828	B	C2	1,2		64	Male	TCGA	1058	Dead
TCGA-NC	High	High	H-TMB+H-ICI score	1.5306	A	C2	3,4		70	Male	TCGA	448	Dead
TCGA-60-	High	High	H-TMB+H-ICI score	-0.6876	A	C4	1,2		76	Femal	TCGA	109	Dead
TCGA-22-	High	High	H-TMB+H-ICI score	0.03327	A	C2	3,4		80	Male	TCGA	623	Dead
TCGA-77-	High	Low	L-TMB+H-ICI score	0.28225	A	C2	3,4		76	Male	TCGA	803	Dead
TCGA-NK	High	High	H-TMB+H-ICI score	-0.5511	B	C2	1,2		77	Male	TCGA	2542	Alive
TCGA-56-	High	High	H-TMB+H-ICI score	0.24435	B	C2	1,2		58	Femal	TCGA	1011	Alive
TCGA-63-	Low	Low	L-TMB+L-ICI score	-2.4732	A	C1	1,2		78	Femal	TCGA	345	Dead
TCGA-52-	High	High	H-TMB+H-ICI score	-0.6236	B	C4	1,2		62	Femal	TCGA	862	Alive
GSM442070						C1	1,2		64	Femal	GSE17710	150.8008215	
GSM442071						C1	1,2		61	Male	GSE17710	172.4845995	
GSM442072						C1	1,2		61	Femal	GSE17710	666.2833677	
GSM442073						C1	1,2		80	Femal	GSE17710	119.2607802	
GSM442074						C1	1,2		68	Femal	GSE17710	151.7864475	
GSM442075						C1	1,2		61	Male	GSE17710	41.396304	
GSM442076						C1	1,2		59	Femal	GSE17710	371.5811088	
GSM442077						C1	1,2		57	Femal	GSE17710	1.9712526	
GSM442078						C1	1,2		73	Femal	GSE17710	1609.527721	
GSM442079						C1	1,2		74	Male	GSE17710	2527.145791	
GSM442080						C1	1,2		53	Male	GSE17710	112.3613964	
GSM442081						C1	1,2		68	Femal	GSE17710	1114.743326	
GSM442082						C1	1,2		69	Femal	GSE17710	1321.724846	
GSM442083						C1	1,2		76	Femal	GSE17710	594.332649	
GSM442084						C1	1,2		63	Male	GSE17710	488.8706367	
GSM442085						C1	1,2		55	Male	GSE17710	3429.979466	
GSM442086						C1	1,2		49	Femal	GSE17710	532.238193	
GSM442087						C1	1,2		81	Male	GSE17710	1277.371663	
GSM442088						C1	3,4		82	Male	GSE17710	825.9548256	
GSM442089						C1	1,2		61	Femal	GSE17710	1008.295688	
GSM442090						C1	1,2		73	Male	GSE17710	12.8131416	
GSM442091						C1	1,2		65	Femal	GSE17710	931.4168379	
GSM442092						C1	1,2		61	Femal	GSE17710	447.4743327	
GSM442093						C1	1,2		53	Male	GSE17710	653.4702258	
GSM442094						C1	1,2		76	Male	GSE17710	94.6201233	
GSM442095						C1	1,2		83	Femal	GSE17710	702.7515399	
GSM442096						C1	1,2		85	Male	GSE17710	2441.396304	
GSM442097						C1	1,2		42	Femal	GSE17710	231.6221766	
GSM442098						C3	1,2		62	Male	GSE17710	1162.053388	
GSM442099						C1	1,2		78	Male	GSE17710	18.7268994	
GSM442100						C1	1,2		58	Femal	GSE17710	647.5564683	
GSM442101						C1	1,2		81	Male	GSE17710	764.8459959	
GSM442102						C1	1,2		79	Male	GSE17710	9.8562627	
GSM442103						C1	1,2		73	Femal	GSE17710	1829.322382	
GSM442104						C1	1,2		50	Femal	GSE17710	1766.2423	
GSM442105						C1	1,2		65	Male	GSE17710	803.285421	
GSM442106						C1	1,2		67	Male	GSE17710	411.9917865	
GSM442107						C1	1,2		81	Femal	GSE17710	1477.453799	
GSM442108						C1	1,2		73	Male	GSE17710	1543.49076	
GSM442109						C1	1,2		80	Male	GSE17710	729.3634497	
GSM442110						C1	1,2		49	Male	GSE17710	882.1355235	
GSM442111						C1	1,2		75	Femal	GSE17710	834.825462	
GSM442112						C1	1,2		59	Male	GSE17710	405.0924024	
GSM442113						C1	1,2		73	Male	GSE17710	1049.691992	
GSM442114						C1	1,2		67	Male	GSE17710	509.5687884	
GSM442115						C1	1,2		67	Femal	GSE17710	24.6406572	
GSM442116						C1	1,2		52	Male	GSE17710	799.3429158	
GSM442117						C1	1,2		41	Femal	GSE17710	481.9712526	
GSM442118						C1	1,2		70	Male	GSE17710	466.2012321	
GSM442119						C1	3,4		68	Male	GSE17710	899.8767966	
GSM442120						C1	1,2		78	Femal	GSE17710	822.0123204	
GSM442121						C1	1,2		70	Male	GSE17710	487.8850104	
GSM442122						C1	1,2		70	Male	GSE17710	4.9281315	
GSM442123						C1	1,2		66	Male	GSE17710	373.5523614	
GSM442124						C3	3,4		67	Male	GSE17710	691.9096509	
GSM442125						C1	1,2		63	Male	GSE17710	630.8008215	
GSM4750621						C1		1	63	Male	GSE157010	1259.506849	
GSM4750622						C1		1	78	Male	GSE157010	993.2054795	
GSM4750623						C3		0	68	Male	GSE157010	2071.232877	
GSM4750624						C3		0	71	Female	GSE157010	2836.60274	
GSM4750625						C3		1	83	Male	GSE157010	452.7123288	
GSM4750626						C4		1	63	Female	GSE157010	835.3972603	
GSM4750627						C3		0	73	Male	GSE157010	1945.972603	
GSM4750628						C2		1	71	Male	GSE157010	234.739726	
GSM4750629						C3		1	60	Male	GSE157010	690.4109589	
GSM4750630						C3		0	76	Female	GSE157010	1045.479452	
GSM4750631						C2		1	72	Male	GSE157010	581.9178082	
GSM4750632						C1		0	78	Male	GSE157010	1919.342466	
GSM4750633						C2		1	72	Male	GSE157010	982.3561644	
GSM4750634						C1		1	71	Female	GSE157010	1091.835616	
GSM4750635						C3		1	68	Female	GSE157010	671.6712329	
GSM4750636						C2		0	59	Male	GSE157010	1880.876712	
GSM4750637						C2		0	83	Female	GSE157010	1959.780822	
GSM4750638						C1		1	57	Female	GSE157010	323.5068493	
GSM4750639						C4		1	52	Male	GSE157010	1534.684932	
GSM4750640						C1		1	66	Male	GSE157010	226.8493151	
GSM4750641						C2		0	55	Female	GSE157010	238.6849315	
GSM4750642						C1		0	79	Male	GSE157010	2388.821918	
GSM4750643						C1		0	62	Female	GSE157010	1802.958904	
GSM4750644						C2		0	51	Female	GSE157010	1843.39726	
GSM4750645						C3		1	81	Female	GSE157010	967.5616438	
GSM4750646						C1		0	75	Male	GSE157010	1820.712329	
GSM4750647						C2		1	59	Male	GSE157010	1609.643836	
GSM4750648						C2		0	62	Male	GSE157010	1868.054795	
GSM4750649						C1		0	74	Male	GSE157010	2327.671233	

GSM4750650	C1	1	79	Male	GSE157010	966.5753425
GSM4750651	C3	0	64	Female	GSE157010	1815.780822
GSM4750652	C3	1	55	Female	GSE157010	258.4109589
GSM4750653	C2	0	72	Female	GSE157010	2216.219178
GSM4750654	C1	1	72	Male	GSE157010	176.5479452
GSM4750655	C2	1	70	Male	GSE157010	962.630137
GSM4750656	C1	1	84	Female	GSE157010	836.3835616
GSM4750657	C1	1	81	Female	GSE157010	691.3972603
GSM4750658	C3	0	66	Male	GSE157010	2113.643836
GSM4750659	C4	0	68	Female	GSE157010	1935.123288
GSM4750660	C4	0	63	Male	GSE157010	1830.575342
GSM4750661	C2	0	73	Male	GSE157010	1835.506849
GSM4750662	C1	0	79	Male	GSE157010	1808.876712
GSM4750663	C1	0	61	Male	GSE157010	1151.013699
GSM4750664	C1	1	54	Male	GSE157010	1389.69863
GSM4750665	C1	1	66	Female	GSE157010	1596.821918
GSM4750666	C1	1	74	Female	GSE157010	1670.794521
GSM4750667	C1	0	81	Male	GSE157010	1996.273973
GSM4750668	C1	1	61	Male	GSE157010	1670.794521
GSM4750669	C2	1	75	Male	GSE157010	280.109589
GSM4750670	C2	1	64	Female	GSE157010	160.7671233
GSM4750671	C1	1	83	Male	GSE157010	788.0547945
GSM4750672	C2	1	60	Male	GSE157010	308.7123288
GSM4750673	C1	0	58	Male	GSE157010	1010.958904
GSM4750674	C1	0	66	Female	GSE157010	1743.780822
GSM4750675	C1	0	64	Female	GSE157010	1805.917808
GSM4750676	C1	0	59	Male	GSE157010	1639.232877
GSM4750677	C1	1	57	Female	GSE157010	1216.109589
GSM4750678	C2	0	74	Male	GSE157010	1513.972603
GSM4750679	C2	1	64	Male	GSE157010	839.3424658
GSM4750680	C4	1	62	Female	GSE157010	1746.739726
GSM4750681	C2	0	66	Male	GSE157010	4.931506849
GSM4750682	C1	0	71	Male	GSE157010	1999.232877
GSM4750683	C2	1	66	Male	GSE157010	751.5616438
GSM4750684	C1	1	79	Male	GSE157010	775.2328767
GSM4750685	C3	0	57	Female	GSE157010	1668.821918
GSM4750686	C4	0	64	Male	GSE157010	964.6027397
GSM4750687	C2	1	88	Male	GSE157010	1030.684932
GSM4750688	C1	0	70	Female	GSE157010	1852.273973
GSM4750689	C2	1	63	Male	GSE157010	237.6986301
GSM4750690	C2	0	79	Female	GSE157010	1886.794521
GSM4750691	C4	0	71	Male	GSE157010	2344.438356
GSM4750692	C1	1	72	Female	GSE157010	1387.726027
GSM4750693	C3	1	62	Male	GSE157010	843.2876712
GSM4750694	C2	1	55	Male	GSE157010	228.8219178
GSM4750695	C1	1	68	Male	GSE157010	1424.219178
GSM4750696	C2	0	72	Male	GSE157010	203.1780822
GSM4750697	C1	1	76	Male	GSE157010	119.3424658
GSM4750698	C1	1	63	Male	GSE157010	295.890411
GSM4750699	C1	1	81	Male	GSE157010	947.8356164
GSM4750700	C1	0	72	Male	GSE157010	2070.246575
GSM4750701	C1	1	78	Female	GSE157010	1325.589041
GSM4750702	C2	1	67	Male	GSE157010	1370.958904
GSM4750703	C1	0	63	Male	GSE157010	1855.232877
GSM4750704	C2	0	67	Male	GSE157010	1944
GSM4750705	C1	1	64	Female	GSE157010	2016
GSM4750706	C2	0	61	Female	GSE157010	1857.205479
GSM4750707	C2	0	79	Male	GSE157010	2037.69863
GSM4750708	C1	1	82	Male	GSE157010	1902.575342
GSM4750709	C1	1	65	Male	GSE157010	498.0821918
GSM4750710	C3	1	64	Male	GSE157010	382.6849315
GSM4750711	C1	0	69	Male	GSE157010	2079.123288
GSM4750712	C1	1	64	Female	GSE157010	1368.986301
GSM4750713	C1	1	82	Female	GSE157010	975.4520548
GSM4750714	C3	1	69	Male	GSE157010	1477.479452
GSM4750715	C1	1	52	Female	GSE157010	2160.986301
GSM4750716	C2	0	73	Male	GSE157010	1886.794521
GSM4750717	C1	0	89	Female	GSE157010	2106.739726
GSM4750718	C2	1	80	Male	GSE157010	1424.219178
GSM4750719	C1	1	70	Male	GSE157010	635.1780822
GSM4750720	C4	0	79	Male	GSE157010	2084.054795
GSM4750721	C1	1	77	Female	GSE157010	2714.30137
GSM4750722	C1	0	69	Male	GSE157010	1823.671233
GSM4750723	C1	0	65	Male	GSE157010	2164.931507
GSM4750724	C1	0	73	Male	GSE157010	2040.657534
GSM4750725	C1	0	54	Male	GSE157010	3062.465753
GSM4750726	C1	1	61	Male	GSE157010	1739.835616
GSM4750727	C1	1	61	Female	GSE157010	532.6027397
GSM4750728	C1	1	71	Male	GSE157010	1423.232877
GSM4750729	C2	0	48	Female	GSE157010	2160.986301
GSM4750730	C1	1	78	Female	GSE157010	976.4383562
GSM4750731	C3	0	80	Male	GSE157010	2385.863014
GSM4750732	C2	0	60	Female	GSE157010	2002.191781
GSM4750733	C1	0	79	Female	GSE157010	1871.013699
GSM4750734	C2	0	59	Male	GSE157010	1851.287671
GSM4750735	C1	1	82	Female	GSE157010	485.260274
GSM4750736	C1	1	84	Male	GSE157010	1430.136986
GSM4750737	C1	0	68	Female	GSE157010	2539.726027
GSM4750738	C1	0	63	Male	GSE157010	1853.260274
GSM4750739	C2	0	69	Male	GSE157010	1832.547945
GSM4750740	C1	0	65	Male	GSE157010	1995.287671
GSM4750741	C1	0	63	Male	GSE157010	2003.178082
GSM4750742	C1	0	51	Male	GSE157010	2065.315068
GSM4750743	C1	0	64	Male	GSE157010	2029.808219
GSM4750744	C1	1	72	Male	GSE157010	1339.39726
GSM4750745	C2	1	73	Male	GSE157010	169.6438356
GSM4750746	C1	1	60	Male	GSE157010	1552.438356
GSM4750747	C3	0	72	Female	GSE157010	1832.547945

GSM4750748	C1	1	76	Male	GSE157010	734.7945205
GSM4750749	C4	0	63	Female	GSE157010	2130.410959
GSM4750750	C1	1	84	Male	GSE157010	137.0958904
GSM4750751	C1	0	63	Female	GSE157010	1996.273973
GSM4750752	C2	1	65	Male	GSE157010	878.7945205
GSM4750753	C1	0	75	Male	GSE157010	1924.273973
GSM4750754	C1	1	61	Male	GSE157010	622.3561644
GSM4750755	C2	0	67	Male	GSE157010	2069.260274
GSM4750756	C1	0	74	Male	GSE157010	1140.164384
GSM4750757	C1	0	71	Female	GSE157010	1967.671233
GSM4750758	C1	1	65	Male	GSE157010	1812.821918
GSM4750759	C1	0	79	Female	GSE157010	1863.123288
GSM4750760	C1	1	80	Male	GSE157010	1949.917808
GSM4750761	C3	1	78	Male	GSE157010	292.9315068
GSM4750762	C2	0	66	Male	GSE157010	1823.671233
GSM4750763	C1	1	82	Female	GSE157010	595.7260274
GSM4750764	C2	0	65	Female	GSE157010	1921.315068
GSM4750765	C1	0	78	Male	GSE157010	1671.780822
GSM4750766	C4	1	70	Male	GSE157010	804.8219178
GSM4750767	C2	1	59	Male	GSE157010	911.3424658
GSM4750768	C1	0	85	Female	GSE157010	2611.726027
GSM4750769	C3	0	65	Female	GSE157010	1178.630137
GSM4750770	C2	1	74	Female	GSE157010	1533.69863
GSM4750771	C4	1	76	Female	GSE157010	1876.931507
GSM4750772	C1	1	74	Male	GSE157010	1570.191781
GSM4750773	C2	1	61	Female	GSE157010	466.5205479
GSM4750774	C1	1	64	Female	GSE157010	254.4657534
GSM4750775	C3	1	64	Male	GSE157010	221.9178082
GSM4750776	C4	0	56	Male	GSE157010	1820.712329
GSM4750777	C1	0	70	Male	GSE157010	1895.671233
GSM4750778	C3	1	59	Female	GSE157010	413.260274
GSM4750779	C2	0	73	Female	GSE157010	1998.246575
GSM4750780	C1	0	69	Male	GSE157010	353.0958904
GSM4750781	C1	0	71	Female	GSE157010	1825.643836
GSM4750782	C2	0	68	Male	GSE157010	1915.39726
GSM4750783	C1	0	61	Male	GSE157010	1810.849315
GSM4750784	C1	0	71	Male	GSE157010	1859.178082
GSM4750785	C1	1	78	Male	GSE157010	143.0136986
GSM4750786	C3	0	73	Male	GSE157010	1924.273973
GSM4750787	C1	1	59	Male	GSE157010	1530.739726
GSM4750788	C1	1	65	Male	GSE157010	306.739726
GSM4750789	C3	1	62	Female	GSE157010	780.1643836
GSM4750790	C1	1	76	Male	GSE157010	541.4794521
GSM4750791	C1	1	68	Male	GSE157010	1288.109589
GSM4750792	C1	0	69	Female	GSE157010	1483.39726
GSM4750793	C1	1	73	Male	GSE157010	360
GSM4750794	C3	1	79	Male	GSE157010	864
GSM4750795	C1	0	68	Female	GSE157010	2577.205479
GSM4750796	C1	0	72	Female	GSE157010	2730.082192
GSM4750797	C2	1	59	Male	GSE157010	663.7808219
GSM4750798	C1	0	73	Female	GSE157010	1835.506849
GSM4750799	C2	1	53	Male	GSE157010	1182.575342
GSM4750800	C1	1	60	Male	GSE157010	1396.60274
GSM4750801	C1	1	81	Male	GSE157010	518.7945205
GSM4750802	C3	1	61	Male	GSE157010	106.5205479
GSM4750803	C2	0	65	Male	GSE157010	2007.123288
GSM4750804	C1	0	68	Female	GSE157010	1670.794521
GSM4750805	C1	1	68	Male	GSE157010	245.5890411
GSM4750806	C1	0	71	Male	GSE157010	1940.054795
GSM4750807	C2	1	55	Female	GSE157010	256.4383562
GSM4750808	C2	1	63	Male	GSE157010	441.8630137
GSM4750809	C1	1	65	Male	GSE157010	383.6712329
GSM4750810	C1	1	66	Male	GSE157010	564.1643836
GSM4750811	C4	0	59	Male	GSE157010	1864.109589
GSM4750812	C1	1	65	Male	GSE157010	1246.684932
GSM4750813	C1	0	65	Female	GSE157010	1870.027397
GSM4750814	C1	0	55	Male	GSE157010	393.5342466
GSM4750815	C1	1	64	Male	GSE157010	811.7260274
GSM4750816	C2	1	70	Female	GSE157010	932.0547945
GSM4750817	C2	0	46	Male	GSE157010	2138.30137
GSM4750818	C1	1	67	Male	GSE157010	841.3150685
GSM4750819	C2	0	73	Male	GSE157010	1831.561644
GSM4750820	C1	0	71	Male	GSE157010	2071.232877
GSM4750821	C2	0	55	Male	GSE157010	1907.506849
GSM4750822	C1	1	86	Female	GSE157010	209.0958904
GSM4750823	C1	0	70	Female	GSE157010	2120.547945
GSM4750824	C3	1	75	Male	GSE157010	707.1780822
GSM4750825	C3	1	68	Female	GSE157010	1615.561644
GSM4750826	C3	0	73	Male	GSE157010	1836.493151
GSM4750827	C2	0	48	Male	GSE157010	1840.438356
GSM4750828	C1	1	66	Female	GSE157010	139.0684932
GSM4750829	C1	0	73	Male	GSE157010	1831.561644
GSM4750830	C2	0	71	Female	GSE157010	1923.287671
GSM4750831	C1	0	61	Male	GSE157010	1820.712329
GSM4750832	C1	1	81	Female	GSE157010	423.1232877
GSM4750833	C2	0	70	Male	GSE157010	2036.712329
GSM4750834	C1	0	70	Female	GSE157010	2057.424658
GSM4750835	C1	1	71	Male	GSE157010	1449.863014
GSM4750836	C1	0	82	Male	GSE157010	1850.30137
GSM4750837	C1	0	66	Male	GSE157010	1035.616438
GSM4750838	C1	0	60	Female	GSE157010	1934.136986
GSM4750839	C1	0	77	Male	GSE157010	2071.232877
GSM4750840	C1	0	65	Male	GSE157010	2156.054795
GSM4750841	C1	1	67	Male	GSE157010	1994.30137
GSM4750842	C1	0	69	Male	GSE157010	1808.876712
GSM4750843	C1	1	52	Male	GSE157010	452.7123288
GSM4750844	C1	0	65	Male	GSE157010	1851.287671
GSM4750845	C1	0	75	Female	GSE157010	2061.369863

GSM4750846	C1	0	82	Female	GSE157010	1731.945205
GSM4750847	C2	0	59	Male	GSE157010	1926.246575
GSM4750848	C1	0	82	Female	GSE157010	1728
GSM4750849	C1	1	57	Female	GSE157010	589.8082192
GSM4750850	C1	1	53	Female	GSE157010	1164.821918
GSM4750851	C1	0	70	Male	GSE157010	1632.328767
GSM4750852	C3	1	63	Male	GSE157010	639.1232877
GSM4750853	C1	1	77	Male	GSE157010	360
GSM4750854	C1	0	69	Male	GSE157010	1911.452055
GSM4750855	C1	0	72	Female	GSE157010	1805.917808

Table-S2: Relative Fractions of tumor-infiltrating immune cells of 792 LUSC patients

Tag	B_cells_m	B_cells_na	T_cells_CD	T_cells_CD	T_cells_CD	T_cells_CD	T_cells_fol	T_cells_ga	T_cells_re	Dendritic_	Dendritic_	Eosinophi	Macropha	Macropha	Macropha	Mast_cells	Mast_cells	Monocyte	Neutroph	NK_cells_a	NK_cells_r	Plasma_cells
TCGA-34	-0.3625	-0.1294	-0.0194	-0.1703	-0.2832	1.04749	-0.205	1.31043	-0.5526	0.6713	2.19334	-0.2692	-1.2504	-0.3269	1.06954	-0.5036	0.95602	0.6476	-0.2573	-0.5924	-0.669	-0.6726
TCGA-96	-0.3625	0.20662	0.0812	1.23276	-0.2832	-0.4462	-1.1257	-0.3373	-0.5526	-0.5416	1.81631	-0.2286	-0.5014	0.47018	-0.4913	-0.5036	0.22248	4.42947	-0.3744	-0.5924	0.53081	-0.0575
TCGA-85	1.1382	-0.7909	-0.4249	0.84481	-0.2832	0.60992	1.09238	-0.4735	1.13564	-0.5416	-0.7162	-0.2692	-0.0145	-0.1896	0.55368	-0.5036	1.15648	-0.3974	-0.6123	-0.5924	-0.669	-0.4056
TCGA-85	-0.3625	1.17146	0.17016	0.08432	-0.2832	1.69572	0.52018	-0.4735	1.17763	-0.5416	-0.6862	-0.2692	-0.8874	0.05245	-1.369	-0.5036	0.81704	-0.3974	-0.4228	0.8146	-0.669	0.18778
TCGA-85	-0.3625	1.43099	-0.0152	1.0825	-0.2832	-0.5073	-0.3048	-0.4735	0.51918	-0.5416	-0.8413	-0.2692	0.52792	-0.227	-0.6441	-0.5036	-0.4274	-0.3974	-0.3521	-0.5924	-0.1616	0.22957
TCGA-66	0.01399	-0.6404	-0.6097	-0.8101	-0.2832	0.20562	-0.0211	-0.4735	-0.4986	3.32852	-0.8413	-0.2692	0.11196	-0.8774	1.11232	-0.5036	1.80783	-0.3974	-0.2366	-0.3849	0.44009	-0.0986
TCGA-22	-0.3625	1.15565	1.02595	-0.5693	-0.2832	1.32968	0.20093	0.1699	-0.5526	-0.4633	0.32737	-0.2692	-0.9219	-0.1944	-0.6924	-0.5036	0.45148	0.04421	-0.4719	-0.0425	0.29045	0.0049
TCGA-96	-0.3625	0.68607	0.41878	0.85146	-0.2832	1.81399	0.08817	-0.4735	1.10419	-0.5416	-0.5401	-0.2692	-0.4889	0.71106	-0.8596	-0.5036	0.5125	-0.3974	-0.4369	0.14041	-0.669	-1.2705
TCGA-77	-0.3625	0.09071	0.27566	1.6697	-0.2832	0.53014	0.07051	-0.4735	-0.5526	0.26948	-0.8413	-0.2692	0.00259	0.18316	-0.4358	-0.5036	-0.3248	-0.3974	-0.3399	-0.5924	2.17283	-1.0053
TCGA-63	-0.3625	-0.0579	-0.9056	0.74007	-0.2832	0.20738	1.38897	-0.4735	-0.5526	5.78087	-0.8413	-0.2692	-1.046	-1.6769	-0.5714	-0.5036	0.87451	0.51076	4.38196	0.63199	-0.669	-1.0458
TCGA-90	-0.0211	-0.8378	-1.0809	0.88783	-0.2832	-0.6686	-0.2172	-0.4735	-0.5526	0.61653	0.44643	8.97853	-0.5634	-1.2898	1.78162	-0.5036	1.64134	7.85145	0.16684	0.8773	-0.4313	-1.1586
TCGA-85	-0.3625	1.62254	-0.5119	0.38434	-0.2832	-0.2275	-1.1257	-0.4735	-0.5526	-0.5416	-0.693	-0.2692	0.32968	1.72668	0.31016	-0.5036	0.17783	-0.3974	-0.6123	-0.3708	-0.5826	-0.7533
TCGA-37	-0.3625	-0.5625	1.60058	-0.9847	-0.2832	1.04143	2.78884	-0.4735	-0.5526	0.52467	0.25097	1.63783	-0.8159	1.14735	-0.2354	0.1171	-0.9589	-0.0459	0.26792	1.33499	-0.669	-1.1874
TCGA-O2	-0.3625	2.37039	-0.3137	-0.4285	-0.2832	0.34197	0.32995	-0.4735	-0.3256	0.97588	-0.8413	-0.2692	-0.4232	-0.038	-0.1375	-0.5036	-0.0139	-0.0562	-0.6123	-0.1119	-0.669	0.06459
TCGA-58	-0.3625	0.58554	-1.0809	0.75108	-0.2832	-0.2801	0.37076	-0.4735	-0.4111	0.14916	0.3326	-0.18	1.19589	-0.254	-1.0498	0.47714	-0.9589	0.45047	-0.2708	1.46883	-0.669	-0.912
TCGA-66	-0.3625	0.30193	-0.0077	-0.7118	-0.2832	-0.5729	-0.3039	-0.4735	-0.3587	0.12255	-0.1218	-0.2692	2.57933	-1.6562	0.42804	-0.5036	-0.3953	-0.3974	-0.1864	-0.5924	-0.018	-0.7081
TCGA-77	-0.3625	2.64385	-0.9052	-1.2082	-0.2832	0.93586	0.08544	0.18878	-0.5526	0.27338	-0.8413	-0.2692	-0.6148	-1.2741	-1.332	0.52227	0.22576	0.70035	-0.0476	-0.5924	-0.669	1.75259
TCGA-98	-0.3625	1.68122	-0.4991	-0.0276	-0.2832	0.09759	-0.1178	-0.4735	-0.5526	-0.5416	-0.0029	-0.2692	-0.5926	-0.0597	-0.0457	-0.5036	-0.5575	-0.2828	0.05187	0.07841	-0.669	1.29851
TCGA-66	-0.3625	-0.4931	0.67865	1.20325	-0.2832	1.61	1.06976	-0.4735	-0.5526	-0.4758	-0.3605	-0.2692	-1.1723	0.36964	-0.255	-0.5036	1.46446	0.71278	-0.0989	1.34324	-0.396	-1.1526
TCGA-43	-0.3625	-0.7212	-1.0809	-0.9199	-0.2832	-0.7692	1.64011	-0.4735	-0.4973	5.14775	-0.8413	-0.2692	1.77888	-1.7519	-2.1135	5.35238	-0.9589	-0.3974	0.32074	-0.5924	-0.669	-1.1843
TCGA-22	-0.3625	-0.7015	-0.7363	0.44943	-0.2832	0.36789	-0.5746	-0.4735	-0.5526	0.27718	0.47253	-0.2692	0.01477	-1.2421	2.3769	-0.5036	1.70942	-0.3974	1.52616	0.00189	-0.0746	-1.2705
TCGA-60	-0.3625	0.85084	-0.248	0.56867	-0.2832	-0.5136	-1.1257	-0.4735	-0.5526	-0.5416	-0.3802	-0.2692	0.24804	-0.4229	1.01141	-0.5036	-0.1861	-0.3974	0.19514	-0.5924	-0.5646	0.65205
TCGA-77	-0.3625	-0.1721	-1.0809	0.08305	-0.2832	-0.2221	-0.9183	-0.4735	-0.5526	1.62347	2.73113	4.39396	-0.2496	-1.4787	2.86739	-0.5036	1.12739	-0.3974	-0.6123	-0.1868	-0.669	-1.2705
TCGA-63	-0.3625	1.21493	-0.6667	0.23231	-0.2832	-0.2792	-0.4111	-0.4735	-0.4938	0.65947	-0.4199	-0.2692	-0.6493	-1.5847	-0.8595	-0.2212	-0.0485	0.71	-0.1734	-0.5924	-0.2352	3.12799
TCGA-66	-0.3625	-0.0617	-0.8023	0.32411	-0.2832	-1.1028	-0.9068	-0.4735	-0.5526	0.25921	0.50807	-0.2692	-0.2905	-0.928	1.25226	-0.5036	-0.1893	-0.3974	9.19681	-0.5924	0.3707	-0.9974
TCGA-43	-0.3625	0.76524	-0.59	0.67531	-0.2832	-1.0755	-1.1257	-0.3705	1.24052	-0.5416	0.32336	-0.2692	0.82417	-0.7916	0.00449	-0.5036	0.26367	0.52255	0.10954	-0.5924	0.91371	0.1282
TCGA-52	-0.3625	0.41905	-1.0809	0.90295	-0.2832	-1.0759	0.45783	-0.4735	0.33814	-0.5416	-0.8413	-0.2692	2.16575	-0.2222	-0.0618	-0.5036	-0.0332	-0.3974	-0.4847	-0.2315	-0.669	-0.6907
TCGA-85	0.20047	-0.056	-0.4408	1.96329	-0.2832	1.5296	-0.8281	-0.4735	0.60268	-0.2191	-0.8413	-0.2692	-0.2491	-0.001	-0.4598	-0.5036	0.28994	-0.3974	-0.6123	-0.5924	-0.669	-0.3076
TCGA-85	-0.0142	0.40992	0.32331	0.34355	-0.2832	0.55281	-0.5117	-0.4735	0.97598	-0.3144	-0.7404	-0.2692	0.08011	-0.7779	0.78165	-0.5036	-0.0799	-0.3974	-0.6123	-0.5924	0.87784	-0.2639
TCGA-94	-0.3625	0.82386	-0.7318	0.03496	-0.2832	1.01347	0.88209	-0.4735	-0.5526	2.97238	-0.8413	-0.2692	-0.8004	-0.1194	-1.5686	-0.5036	0.71188	0.79316	-0.6123	2.21369	-0.669	0.06777
TCGA-60	1.58158	0.34855	-0.1116	0.88209	-0.2832	0.79481	-0.4816	-0.4735	1.30548	-0.3179	-0.0322	-0.2692	-0.9941	-0.8324	-1.3685	-0.5036	0.35543	0.61468	-0.3342	-0.19	-0.669	1.43799
TCGA-77	-0.3625	3.05896	-0.8675	0.2072	-0.2832	-1.0193	-1.1257	-0.3628	-0.5526	-0.1215	-0.8413	-0.2692	1.06849	-1.119	-1.1638	0.84589	-0.9589	-0.3974	-0.5167	-0.5924	1.07922	0.2536
TCGA-85	-0.3625	0.72612	-0.3388	1.20388	-0.2832	-0.7714	-1.1257	-0.4735	-0.5526	-0.5416	-0.7943	-0.2692	0.567	-0.7122	-0.1442	-0.5036	1.12479	-0.3974	-0.4351	-0.5924	1.21824	0.46947
TCGA-39	-0.3625	1.44901	-0.2099	0.90047	-0.2832	0.36827	-0.1981	-0.4735	-0.5526	-0.5416	-0.1497	-0.2692	-0.6571	0.06631	-0.5223	-0.5036	0.05303	-0.0893	-0.6123	-0.5198	-0.2206	0.77215
TCGA-66	-0.3625	0.02092	0.3467	0.14544	-0.2832	0.01845	-0.5137	-0.4735	-0.5526	-0.5416	1.9909	1.45659	-0.9199	0.62198	1.32117	-0.5036	0.44832	-0.1685	-0.1013	-0.5924	-0.1681	-0.317
TCGA-33	-0.3625	2.44061	-0.9386	0.25908	-0.2832	-1.1028	-1.1257	0.1458	0.07334	-0.5416	-0.8413	-0.2692	1.26593	-1.2644	0.85082	0.37181	-0.9589	-0.0929	-0.6123	-0.5924	0.19172	-0.4774
TCGA-NC	-0.3625	-0.1208	-0.2103	-0.3721	-0.2832	1.89941	3.08568	-0.3107	-0.5526	-0.5416	-0.1248	-0.2692	-0.1921	1.26404	-0.1246	-0.5036	-0.8336	-0.3974	-0.6123	0.21768	-0.669	-0.9672
TCGA-22	-0.3625	1.15218	-1.0809	1.82675	-0.2832	0.44577	-1.1257	-0.4735	-0.5526	1.6493	-0.6458	-0.2692	-1.3295	-1.1023	-0.4387	-0.5036	0.44545	3.90414	1.29056	1.08349	-0.5225	0.16543
TCGA-60	-0.3625	0.45526	0.10177	0.219	-0.2832	2.03179	0.37368	-0.4735	0.31856	-0.5366	-0.3029	-0.2692	-0.9653	0.13674	0.18426	-0.5036	-0.0443	0.36187	-0.2358	-0.5924	1.29421	-0.5891
TCGA-77	-0.3625	-0.6204	0.19274	-0.7787	-0.2832	0.93539	-0.6537	-0.4735	2.68049	-0.5416	2.68589	-0.2692	0.59261	-0.1703	0.50955	-0.5036	-0.5763	-0.3974	-0.6105	0.14888	-0.4746	-0.9754
TCGA-63	-0.3625	1.11403	-0.1327	0.21792	-0.2832	0.33864	-1.0134	-0.1736	-0.5394	-0.2541	-0.8413	-0.2692	0.69342	-0.2289	-0.3739	-0.5036	-0.4098	-0.3974	-0.6123	-0.5924	-0.0165	0.32801
TCGA-22	-0.3625	1.40292	-0.4235	-0.8772	-0.2832	-0.4287	-0.5636	-0.4735	-0.5526	-0.3478	-0.8413	-0.2692	1.14422	-0.9719	-0.5998	0.75445	-0.9589	-0.3974	-0.6123	-0.5924	-0.669	1.65815
TCGA-21	-0.3625	0.36383	-1.0809	0.1033	-0.2832	-0.2091	-1.1257	-0.4735	-0.3008	-0.2854	-0.8413	-0.2692	0.16452	-1.6561	0.43001	3.95462	-0.9589	0.13183	1.28115	-0.5924	3.02518	-0.5391
TCGA-O2	-0.3625	0.47941	-1.0809	2.27379	-0.2832	-0.6584	-1.1257	-0.4735	-0.5526	0.68572	0.35098	-0.2692	0.9605	-0.9354	0.04491	0.20903	-0.9589	-0.3974	-0.4311	-0.5924	1.74243	-1.1537
TCGA-33	-0.3625	-0.2422	-1.0809	-0.783	-0.2832	-0.9172	1.13518	-0.4735	2.97168	-0.0916	-0.8413	-0.2692	3.70037	-1.4053	-0.0961	-0.5036	-0.6667	-0.3974	-0.4212	0.39868	-0.669	-1.1573
TCGA-63	-0.3625	-0.3131	-1.0809	0.91463	-0.2832	-0.6213	-0.9437	-0.4735	-0.5526	0.72047	0.50688	7.767	-0.0856	-0.8163	0.78035	1.08003	-0.9589	0.58437	0.27689	-0.5924	1.44328	-0.2997
TCGA-96	-0.3625	1.45437	-1.0809	0.44761	-0.2832	-0.2863	0.01652	-0.4735	-0.5526	0.46242	-0.3652	-0.2692	-0.0437	-1.546	0.57387	-0.5036	-0.4663	1.53441	1.17691	-0.5924	1.04322	0.25857
TCGA-63	-0.3625	-0.8623	0.06928	-0.0753	-0.2832	1.51163	-0.2569	-0.4735	-0.5526	-0.5416	1.27635	-0.2692	0.3194	-0.6274	0.96711	-0.5036	1.32634	-0.3974	-0.6123	0.60451	-0.669	-1.2705
TCGA-37	-0.3625	-0.5908	-0.1272	-0.9049	-0.2832	-0.3412	-0.4038	-0.0112	-0.5526	1.22497	-0.8413	-0.2692	0.25806	-0.7906	4.25964	-0.5036	-0.8859	-0.3974	1.23895	1.60599	-0.669	-1.2287
TCGA-56	-0.3625	0.05093	-1.0809	-0.3818	-0.2832	1.39362	-0.6408	-0.4735	-0.2253	0.4956	3.36865	6.58742	-0.5365	-1.7519	1.48099	-0.5036	0.03951	1.67509	-0.5772	-0.5924	-0.669	-0.75
TCGA-56	-0.3625	3.02989	-1.0809	0.50564	-0.2832	-0.4139	-0.5755	-0.4735	2.04864	-0.2934	-0.8413	-0.2692	0.60495	-1.621	-0.427	-0.5036	1.05709	-0.0834	-0.6123	1.83349	-0.669	-0.9933
TCGA-98	-0.3625	1.80603	0.71277	1.16765	-0.2832	-0.1132	-1.1257	-0.4735	0.92945	0.03783	-0.8413	-0.2692	-0.0967	-0.8816	-0.7374	-0.5036	1.26585	0.73407	-0.5262	-0.5924	-0.669	-0.335
TCGA-94	-0.3625	0.55239	-0.8516	-0.103	-0.2832	-0.4009	1.12296	-0.4735	2.23232	-0.0273	1.46067	-0.2692	0.86013	-0.8071	-0.0874	-0.5036	0.1362	-0.3974	-0.5999	0.91112	-0.669	-0.8174
TCGA-43	-0.3625	-0.6114	0.04697	-1.0794	-0.2832	4.55986	2.01505	0.29125	0.27827	-0.3993	-0.5318	-0.2692	-1.0418	0.45062	0.12156	-0.5036	-0.6463	-0.3974	-0.6123	1.09592	-0.669	-1.2563
TCGA-NC	-0.3625	-0.7577	0.90811	-1.5382	-0.2832	1.63496	0.67557	-0.4067	-0.5526	-0.5416	-0.8413	-0.2692	-0.2253	0.79487	-0.1939	-0.484	-0.8825	-0.3974	-0.6123	-0.5924	1.63012	1.34655
TCGA-63	-0.3625	0.62815	-0.6454	1.34901	-0.2832	-0.09	-1.1257	-0.4735	0.51227	-0.5416	-0.8413	-0.2692	0.73007	-0.6192	-0.6887	-0.5036	-0.5081	-0.3974	-0.4686	-0.5924	0.37127	0.95458
TCGA-22	-0.3625	0.40206	1.24078	-1.5382	-0.2832	3.41308	0.232	-0.4735	2.26947	-0.3623	-0.1306	-0.2692	-0.6381	0.24294	-0.5355	-0.5036	-0.5664	-0.3974	-0.6123	-0.2604	-0.669	-0.6291
TCGA-77	1.51511	2.36797	-0.9287	0.44823	-0.2832	-0.2902	1.58269	-0.4735	0.98238	0.79559	-0.3493	-0.2692	0.21912	-1.6401	-1.2422	-0.5036	0.01357	-0.3974	-0.6123	-0.0832	-0.669	-0.3482
TCGA-37	-0.3625	-0.1674	0.57419	-0.4031	-0.2832	0.42796	0.07857	-0.4735	0.88918	1.408	0.78414	-0.2692	0.44504	-0.3522	-0.0473	-0.5036	0.16728	0.92546	-0.5957	-0.5924	-0.2848	-0.8715
TCGA-85	-0.3625	-0.335	0.30373	-0.7535	-0.2832	1.73893	2.38582	-0.4735	0.38768	0.34589	0.80304	-0.2692	-1.3432	-0.9224	-1.1877	-0.5036	1.26979	-0.3974	-0.6123	0.48933	6.23844	-0.5123
TCGA-NC	-0.3625	-0.6725	-1.0809	0.10764	-0.2832	-0.2871	0.12647	-0.4735	0.59304	-0.5416	-0.7887	-0.2692	2.89226	-0.6458	-0.0505	0.82525	-0.9589	-0.3974	-0.6123	-0.5924	0.87005	-1.1839
TCGA-85	2.3029	4.53709	-0.5883	-1.5382	-0.2832	0.89885	1.69381	-0.4735	5.66463	0.23816	-0.8413	-0.2692	-0.8973	-1.7267	-1.5033	-0.5036	-0.5685	0.06232	-0.6123	0.15728	-0.669	-0.6418
TCGA-33	-0.3625	1.5478	0.57669	-1.5382	-0.2832	0.89855	0.63225	-0.4735	-0.4393	-0.1311	1.22535	-0.2692	-0.2602	0.58133	-0.9117	-0.5036	-0.3547	-0.3974	-0.6123	-0.5054	-0.5724	0.2384
TCGA-43	-0.3625	0.3551	-0.3261	-1.013	-0.2832	1.44567	0.60125	-0.4735	-0.5526	-0.3144	0.23985	-0.2692	-1.1207	1.09289	0.29209	-0.5036	1.85664	0.831	-0.6123	0.12353	0.05269	-0.0694
TCGA-34	-0.3625	0.02747	-0.4846	-0.7995	-0.2832	0.06479	0.18265	-0.4735	-0.0943	-0.5416	0.74804	-0.2692	1.64687	-0.364	-0.9776	2.29117	-0.9589	-0.3974	-0.2086	0.36282	-0.669	-0.9145
TCGA-56	-0.3625	0.27901	0.18859	0.44857	-0.2832	0.51089	-1.1257	-0.4735	0.86432	-0.5416	-0.8413	-0.2692	1.16553	-0.0606	0.08655	-0.5036	0.70614	-0.3974	-0.6123	-0.5673	-0.0812	-1.1601
TCGA-18	-0.3625	1.55517	0.02074	-1.3507	-0.2832	0.1074	0.51792	-0.4735	0.05484	-0.5416	1.21734	0.95121	-0.1485	-0.0449	-0.8798	-0.5036	1.21644	0.26715	-0.6123	-0.1407	1.36123	-0.0768
TCGA-21	0.43914	-0.8836	-0.6066	-0.5611	-0.2832	1.21519	0.28709	0.38345	0.46454	-0.0387	1.10946	-0.2692	-1.1064	-0.2314	-0.1236	-0.5036	-0.6692	4.19818	0.44573	0.60495	-0.669	1.25333
TCGA-90	-0.3625	1.89204	-0.1795	-1.4243	-0.2832	-0.011	-0.8083	-0.4735	-0.3926	-0.5416	-0.8413	-0.2692	0.08982	-0.9261	-0.4572	-0.5036	0.20537	-0.3974	-0.6123	0.62496	-0.669	2.45315
TCGA-MF	-0.3625	-0.8483	1.76133	-1.5382	-0.2832	3.20582	1.31847	-0.4735	-0.5526	2.15041	-0.4802	0.69767	-0.692	-1.703	-0.009	0.50623	-0.9589	0.66922	-0.4411	-0.5924	2.83416	-1.2037
TCGA-46	-0.3625	0.73643	-0.9845	0.49391	-0.2832	-0.2377	-0.9226	0.10658	-0.1305	-0.5416	0.27849	-0.2692	0.18302	-0.3765	2.09882	-0.5036	1.75603	0.09489	-0.2804	-0.5924	-0.669	-1.1557
TCGA-77	-0.3625	-0.1599	0.71328	-0.3047	-0.2832	1.94192	-0.2304	-0.4735	0.31575	-0.5416	-0.6299	-0.2692	0.09793	1.01343	-0.1408	-0.5036	-0.067	-0.3974	-0.6123	-0.586	-0.512	-0.661
TCGA-56	-0.3625	2.58575	0.22694	-0.3828	-0.2832	0.24589	-0.7267	-0.4735	0.10512	-0.4994	-0.2174	-0.2692	-0.7309	-0.0407	-1.04	-0.5036	0.96711	0.08861	-0.6123	-0.5924	0.67903	0.68271
TCGA-85	-0.3625	2.49824	-1.0395	-0.0827	-0.2832	0.39328	-0.7426	-0.4735	-0.4057	0.7138	-0.8413	0.56977	-0.658	-1.5419	-1.4742	-0.5036	-0.1554	1.11645	-0.6123	-0.5924	-0.669	3.01971
TCGA-56	-0.3625	0.0544	-0.7452	-0.8685	-0.2832	-1.0776	-0.8593	-0.4735	0.07261	-0.5416	1.38784	-0.2692	2.56319	-1.2907	1.50685	-0.5036	-0.0284	-0.3974	0.03672	-0.5924	-0.5928	-0.8608
TCGA-NC	-0.2282	-0.7664	0.71223	-1.2745	-0.2832	0.9411	-0.3143	-0.4735	1.895	1.0165	-0.3597	-0.2692	-0.454	1.72004	1.48482	-0.5036	1.42407	-0.3974	-0.1539	-0.5924	-0.669	-1.2705
TCGA-56	-0.3625	-0.2752	-0.6554	0.76622	-0.2832	-0.956	-1.1257	-0.4735	-0.5526	-0.5416	-0.8413	-0.2692	3.16956	-0.6757	-0.3386	-0.5036	-0.6378	-0.3974	-0.5956	-0.5924	-0.147	-0.3022
TCGA-60	-0.3625	0.0977	-1.0713	2.72949	-0.2832	-0.3327	-1.1257	-0.4735	-0.5526	1.05102	-0.8413	-0.2692	1.02941	-1.5089	-1.822	0.00454	-0.9589	-0.3974	-0.2059	-0.5924	2.08865	0.58297
TCGA-39	-0.3625	0.65326	-0.4267	-0.4989	-0.2832	1.22734	0.02132	-0.4735	0.80613	-0.5416	-0.2895	-0.233	0.72529	-0.2817	-0.5825	-0.5036	-0.9337	-0.3974	-0.5806	-0.5924	-0.669	0.61651
TCGA-33	-0.3625	-0.3555	-0.3624	1.87112	-0.2832	0.22103	0.67511	-0.4574	-0.5199	-0.5416	-0.0812	0.80856	0.43607	-0.0782	-0.1092	0.09485	-0.9589	1.1536	-0.0917	0.7547	-0.669	-1.1168
TCGA-34	-0.3287	-0.6002	-0.243	0.98135	-0.2832	-0.124	-0.4069	-0.456	0.04656	-0.5416	0.59972	-0.2692	1.07756	-0.5262	0.28543	-0.5036	-0.0987	-0.3974	-0.3027	-0.5924	-0.0228	-0.282
TCGA-39	-0.3625	-0.0691	-1.0809	0.58153	-0.2832	0.58934	0.75406	-0.4735	2.85169	0.84842	-0.0468	-0.2692	-0.3506	1.98159	-1.5555	-0.5036	0.60594	-0.1339	-0.5598	2.70201	-0.669	-1.1212
TCGA-77	-0.3625	0.05559	-0.1204	-0.0707	-0.2832	0.2485	-0.5453	-0.4735	-0.5454	-0.055	0.72996	0.3573	0.57376	-1.0147	1.14994	-0.5036	2.4665	-0.3974	-0.5392	-0.5924	-0.0069	-1.2393
TCGA-85	-0.3625	0.79287	-1.0809	-0.2568	-0.2832	0.5058	-0.3994	-0.0467	-0.0253	-0.5416	3.86958	-0.2692	0.38777	-0.1828	-0.9145	-0.5036	0.33893	0.7254	-0.6123	1.23918	-0.669	-1.1783
TCGA-O2	-0.3625	-0.7555	0.04085	0.37011	-0.2832	-0.2389	-0.8418	-0.4657	2.29835	-0.5416	-0.7846	-0.2692	2.30663	0.32975	0.17368	-0.5036	-0.2379	-0.3974	-0.4627	-0.5924	-0.669	-1.2433
TCGA-68	-0.3625	-0.0438	-0.0188	0.70831	-0.2832	0.50608	0.19017	-0.4735	-0.5526	0.30223	-0.4996	-0.2692	-0.2878	0.66528	0.58465	-0.5036	0.18732	1.17705	0.017	-0.5924	1.31346	-1.0916
TCGA-70	-0.3625	-0.905	1.55473	-0.0843	-0.2832	3.7314	0.51844	-0.4735	-0.5526	-0.5416	-0.223	-0.2692	-1.049	0.06564	-0.2783	-0.5036	-0.0713	0.07829	-0.6123	0.51653	0.59566	-1.119
TCGA-68	-0.3625	0.2532	-0.5153	2.76675	-0.2832	0.2188	-1.1257	-0.4735	-0.5526	-0.5416	-0.5309	-0.2692	0.78997	0.05458	-0.7762	-0.5036	0.34358	-0.3974	-0.4927	-0.4725	-0.669	-0.8342
TCGA-85	0.45374	0.50974	-0.0773	0.52106	-0.2832	0.23865	1.29446	-0.4735	2.39121	0.67493	0.15286	-0.2692	-0.3953	-0.4723	-0.957	-0.5036	-0.0804	-0.3974	-0.5013	0.10347	-0.669	0.07932
TCGA-66	-0.3625	-0.1369	-0.1702	-0.1828	-0.2832	0.48016	1.15749	-0.4735	2.27046	-0.5416	-0.7207	-0.2692	-0.3551	-0.1025	0.6129	-0.5036	1.75743	0.32687	-0.6123	1.1905	-0.669	-0.309
TCGA-85	-0.3625	-0.1235	-0.1146	2.52339	-0.2832	1.68348	-1.1257	-0.4735	-0.5526	-0.0955	0.03311	-0.2692	-0.8961	-0.318	0.04108	-0.4992	0.41904	1.10775	-0.1003	-0.5924	-0.1386	-0.8437
TCGA-94	-0.3625	-0.0973	-1.0809	1.73303	-0.2832	0.29532	0.34888	-0.4735	2.73906	-0.0024	1.6648	-0.2692	-0.0958	-0.3615	-0.5095	-0.5036	0.35994	0.88863	-0.6123	-0.0481	-0.669	-0.7314
TCGA-22	-0.0071	0.09248	0.19144	-0.3045	-0.2832	0.09595	-0.5419	-0.4735	1.0261	-0.0298	-0.8413	-0.2692	-0.3415	-0.6789	-0.9278	-0.5036	-0.3383	-0.1053	-0.6123	-0.5924	-0.4354	3.36119
TCGA-85	-0.3625	0.80678	-1.0809	-0.3527	-0.2832	0.65892	0.423	-0.4735	2.95961	-0.5416	-0.8413	-0.2692	2.65165	-0.7671	-1.9393	-0.3072	-0.9589	-0.3974	-0.6123	1.19815	-0.669	-1.0901
TCGA-63	-0.3625	0.10802	-1.0657	0.55096	-0.2832	-0.2047	0.24949	-0.4735	-0.5526	0.87524	-0.8413	-0.2692	2.04008	-0.3239	-1.7584	2.26487	-0.9589	-0.3974	-0.6123	-0.5924	0.57631	-1.1355
TCGA-94	-0.3625	3.29015	0.01402	-1.1276	-0.2832	2.63979	0.54453	-0.4735	-0.5526	0.19206	0.75245	-0.2692	-0.9947	-0.7163	-1.7064	-0.5036	0.31506	0.83454	-0.6123	-0.4332	0.05739	-0.8313
TCGA-85	-0.3625	1.70091	-0.8335	0.2323	-0.2832	-0.2437	-1.1257	-0.4735	1.07027	-0.5416	-0.8413	-0.2692	0.39157	-0.2079	0.6301	0.78197	-0.9589	-0.3974	-0.1253	-0.5924	-0.417	-0.0972
TCGA-60	-0.3625	0.66582	-1.0373	0.73473	-0.2832	-0.2542	0.78872	-0.4735	1.38045	-0.0211	0.55859	-0.2692	0.27747	-0.6117	0.2374	-0.5036	0.67327	-0.3974	-0.4886	0.13399	-0.5016	-0.5039
TCGA-O2	-0.3625	0.82371	-0.743	0.07935	-0.2832	-0.5828	0.88431	-0.4735	-0.5526	-0.5416	0.21931	-0.2692	0.98194	0.37068	-0.5596	-0.5036	-0.186	-0.3974	-0.6123	0.82741	-0.669	-0.0855
TCGA-51	-0.3625	-0.6366	-0.4738	0.01981	-0.2832	-0.2732	-0.5865	-0.4735	-0.4998	-0.2656	-0.7185	-0.2692	0.78958	0.2516	3.70491	-0.5036	0.22007	-0.3974	-0.5462	-0.5924	0.02461	-1.2705
TCGA-22	-0.3625	-0.8436	-1.0809	-0.1657	-0.2832	-0.6574	-0.7559	-0.4735	-0.5526	-0.5416	3.63249	-0.2692	-0.9213	-0.8542	3.62845	-0.5036	4.1563	2.80876	-0.559	0.5384	-0.669	-1.1984
TCGA-85	-0.3625	-0.6204	-0.8465	0.84852	-0.2832	0.26622	-0.6208	-0.4735	0.29443	-0.5416	5.37554	-0.2692	-0.4866	0.0354	0.44635	-0.5036	1.36599	-0.3974	-0.6123	0.10886	-0.669	-1.2374
TCGA-85	-0.3625	0.41065	-0.1467	0.08824	-0.2832	0.50858	0.0587	-0.4735	0.80229	-0.5416	-0.724	-0.2692	0.59691	-0.0615	-0.4387	-0.5036	-0.383	-0.3974	-0.6123	-0.4698	-0.669	0.66559
TCGA-77	-0.3625	0.76854	-1.0809	2.8369	-0.2832	-0.8068	-1.1257	-0.4735	0.15627	-0.474	-0.8413	-0.2692	0.13769	-1.1829	0.18451	1.42312	-0.9589	-0.3974	-0.2468	-0.5924	1.88425	-0.3021
TCGA-39	-0.3625	0.73688	-0.6343	0.88814	-0.2832	-0.867	-0.8582	-0.3404	0.26149	-0.0775	-0.3776	-0.2692	-0.3784	-0.0799	-0.1617	-0.5036	1.05864	-0.3974	0.01837	0.13007	-0.669	1.45006
TCGA-34	-0.3625	0.27108	-0.8456	0.7949	-0.2832	-0.1007	-1.1047	-0.4735	-0.5526	-0.5416	-0.8413	-0.2692	1.40073	1.68496	-0.3126	-0.5036	0.13857	-0.3974	0.02483	-0.5924	0.02933	-1.1369
TCGA-43	-0.3625	0.35786	-0.3188	0.8185	-0.2832	0.64018	-1.1257	0.51597	0.703	-0.5144	-0.7755	0.11059	-0.4335	-0.9427	-0.4237	-0.5036	-0.3347	0.11459	-0.5662	-0.5924	-0.669	2.16695
TCGA-63	-0.3625	2.196	-0.9689	-0.1234	-0.2832	-0.5356	-1.1257	0.44057	-0.2239	-0.4892	-0.8413	-0.2692	-0.0169	-1.1414	-0.6574	-0.5036	0.68243	-0.3974	1.07659	-0.5924	-0.669	1.88257
TCGA-63	-0.3625	0.1913	-0.6806	-0.1987	-0.2832	2.50449	0.01364	-0.4735	0.7518	-0.5416	-0.2791	-0.2692	-1.2155	0.1275	1.99865	-0.5036	1.25258	-0.3833	-0.6123	0.37943	-0.6235	-1.0381
TCGA-39	-0.3625	-0.3549	0.20418	-0.9118	-0.2832	0.38873	-0.5032	-0.4735	5.12245	-0.5416	-0.7555	-0.2692	0.73509	-0.3646	-0.6084	-0.5036	0.29463	-0.3974	-0.6123	0.78838	-0.669	0.85047
TCGA-51	-0.3625	0.70827	-0.0159	0.43195	-0.2832	-0.1997	-0.3596	-0.4735	-0.5526	-0.5416	-0.2569	-0.2692	-0.1581	0.94033	-0.0968	-0.5036	-0.4638	-0.3974	-0.1421	-0.5924	-0.6455	0.91977
TCGA-22	-0.3625	1.32354	-0.2915	-1.0362	-0.2832	0.7972	0.45783	-0.4735	0.70277	-0.5416	1.6267	-0.2692	-0.7895	-0.1592	0.44862	-0.5036	0.32026	-0.0632	-0.3993	0.02766	-0.669	0.14995
TCGA-66	0.02932	-0.6196	0.93211	0.34656	-0.2832	0.93047	-0.3766	-0.4735	-0.5526	0.14076	-0.8413	-0.2692	0.46017	-0.1303	0.52501	-0.5036	-0.3626	-0.3974	0.01652	-0.5924	0.99049	-1.0164
TCGA-33	-0.3625	2.26133	-0.8679	0.70109	-0.2832	-0.6412	-0.7883	-0.4735	0.54171	0.48963	0.33963	-0.2692	-0.7569	-1.2277	-0.4964	-0.4892	0.24761	1.0168	-0.1539	-0.5924	-0.669	1.67843
TCGA-52	-0.323	-0.905	-1.0809	1.15195	0.11126	-0.6629	-1.1257	-0.4735	-0.5526	0.01019	-0.8413	-0.2692	2.10901	-1.7519	-0.5193	3.52711	-0.9589	-0.3974	-0.6123	-0.5924	3.44694	-1.2245
TCGA-77	-0.3625	-0.08	0.02047	-0.0717	-0.2832	0.25313	-0.0774	-0.4735	1.65419	-0.5416	-0.8413	-0.2692	1.78559	0.1093	-0.3667	-0.5036	-0.0876	-0.3974	-0.6123	-0.5924	0.02084	-0.9098
TCGA-66	-0.3625	0.55292	-0.7155	0.24276	-0.2832	-0.2672	-0.742	-0.4735	-0.5526	-0.5416	1.48365	0.83354	-0.1991	-0.5093	1.00408	-0.4178	-0.7152	4.62881	1.19128	-0.582	-0.6287	-0.0251
TCGA-33	-0.3625	-0.7112	2.43594	0.18855	-0.2832	1.49639	-1.1257	-0.4735	-0.5526	0.4556	-0.7363	1.19629	-0.5813	-0.0314	0.36923	-0.5036	0.176	0.07587	-0.5411	0.81513	0.19207	-1.0327
TCGA-90	-0.3625	0.60032	1.23003	-1.4745	-0.2832	0.3793	-0.1503	-0.4735	1.70319	0.16529	-0.793	-0.2692	-0.2364	-0.2377	-0.5199	-0.5036	-0.2803	-0.3974	0.36936	-0.5924	0.42777	1.34237
TCGA-60	-0.3625	0.79456	-1.0809	0.90928	-0.2832	-1.1028	-0.7626	-0.4735	-0.1918	1.04144	-0.8413	-0.2692	1.5388	-1.7519	-0.1214	0.20315	-0.9589	-0.3974	2.97024	-0.5924	0.78032	-0.7586
TCGA-56	-0.3625	0.30749	-0.7398	0.66507	-0.2832	0.24229	-1.1055	-0.4735	-0.4575	0.04394	-0.2186	-0.2692	1.3949	-0.5697	0.09847	-0.5036	-0.3317	-0.3974	-0.6123	-0.5924	0.02622	-0.3814
TCGA-85	-0.3625	0.20664	-0.1554	-0.2142	-0.2832	-1.1028	-0.3862	-0.4735	-0.5526	0.19313	-0.8413	-0.2692	2.10094	-1.0843	0.20965	1.87712	-0.9589	-0.3974	-0.0899	-0.5924	1.4184	-1.1548
TCGA-60	-0.3625	0.00463	0.66852	0.39124	-0.2832	2.43167	-0.9519	-0.4735	1.78577	-0.4047	-0.3229	-0.2692	-1.0176	-0.4709	-0.4891	-0.5036	0.36602	1.06586	-0.4531	-0.5924	-0.0408	0.27303
TCGA-22	-0.3625	-0.1328	-0.1348	1.41924	-0.2832	-0.2059	-1.1257	-0.4735	1.46066	0.30271	1.50608	-0.2692	-0.4592	-0.8252	0.43382	-0.5036	1.10198	-0.3974	0.08552	-0.5924	0.01789	-0.2266
TCGA-33	-0.3625	-0.6934	-1.0809	-0.7532	-0.2832	-0.3703	0.67326	-0.4735	-0.0509	6.84358	3.28303	2.00689	-0.9009	-1.7519	-0.127	-0.5036	1.12615	7.19736	-0.6123	-0.5924	1.55958	-1.065
TCGA-60	-0.3625	0.58404	-0.237	1.74642	-0.2832	0.05571	-1.1257	-0.4735	-0.5526	0.75616	1.44613	-0.2692	-0.6782	-0.8498	1.00256	-0.5036	0.99168	1.21404	-0.0145	-0.1985	-0.669	-1.2705
TCGA-85	-0.3625	0.31152	-0.7566	0.83354	-0.2832	-0.1892	-1.1257	-0.4735	-0.5526	-0.5416	1.90265	0.32524	-0.334	0.40228	1.0222	-0.5036	0.70307	0.32392	-0.4032	0.81992	0.64389	-1.1162
TCGA-58	-0.3625	0.50924	1.4083	-1.5382	-0.2832	1.95834	0.20852	-0.4735	-0.5526	-0.5416	-0.7107	-0.2692	-0.3361	0.367	0.52864	-0.5036	0.70586	-0.3974	-0.504	1.16904	-0.669	-1.0551
TCGA-37	-0.3625	-0.4685	-1.0809	-1.5382	-0.2832	1.00568	-0.2107	-0.4735	-0.5526	-0.0531	-0.8413	5.16396	-1.5147	-1.5011	6.56536	1.86202	-0.9589	4.62926	-0.6123	1.21926	-0.669	-1.2092
TCGA-21	-0.3625	-0.2039	-1.0809	0.35767	-0.2832	-0.3108	0.26903	-0.4735	-0.5526	2.43865	2.20275	-0.2692	-0.7223	-1.2426	1.43573	0.93251	-0.9589	1.79646	-0.3981	-0.5924	1.22399	-0.4526
TCGA-43	0.03335	0.48236	0.13607	0.4812	-0.2832	1.05923	0.17685	-0.4735	0.45807	-0.5416	-0.6647	-0.2692	-0.3618	1.87876	-1.3971	-0.5036	0.4102	-0.3974	-0.6123	-0.5924	-0.669	-0.0943
TCGA-58	-0.3625	0.92016	-0.6398	1.07621	-0.2832	0.84166	0.72128	-0.4735	1.14445	-0.5416	-0.2305	-0.2692	-0.5678	0.51557	-0.5979	-0.5036	0.66936	1.28958	-0.6123	1.07527	-0.669	-0.9452
TCGA-98	-0.3625	0.30002	-1.0809	-0.894	-0.2832	-0.3093	2.09955	-0.4735	-0.5526	-0.5416	0.44523	-0.2692	0.91218	0.44833	0.05031	-0.5036	-0.6534	-0.3974	-0.3744	2.03376	-0.669	-0.1194
TCGA-85	-0.3625	-0.152	-0.729	0.15097	-0.2832	-0.0515	0.08926	-0.4735	0.42105	-0.5416	0.12172	-0.2692	1.10702	-0.3962	0.73964	-0.5036	0.53087	-0.3974	-0.6123	0.32504	-0.669	-0.4799
TCGA-34	-0.3625	0.69477	-0.3545	-1.5382	-0.2832	1.65939	1.34703	-0.4735	3.81621	-0.5416	0.35629	-0.2692	-0.6099	0.31519	-0.765	-0.5036	-0.4663	-0.3974	-0.6123	0.0526	0.88817	0.45411
TCGA-34	-0.3625	-0.491	-0.1579	0.29288	-0.2832	0.33	0.51582	-0.4735	0.14022	2.28756	-0.8413	-0.2692	-0.352	1.14576	2.11549	0.03897	-0.9495	-0.3974	-0.5604	-0.5924	-0.669	-1.2705
TCGA-22	-0.3625	0.71514	-0.6652	0.72666	-0.2832	0.43488	-0.4063	-0.4735	-0.5526	-0.3355	0.4809	-0.2692	-0.9967	-0.6695	0.84745	-0.5036	2.17812	-0.3974	0.2128	2.19248	-0.669	-0.6611
TCGA-98	-0.3625	1.33989	-0.3787	2.0407	-0.2832	0.97427	-1.1257	-0.4735	-0.4066	0.38661	-0.7898	-0.2692	-1.2456	-1.2253	-0.2562	-0.5036	1.86738	2.47873	-0.3884	-0.2567	-0.6408	-0.0287
TCGA-94	-0.3625	1.00006	-1.0809	1.52913	-0.2832	0.40237	-1.0387	-0.4735	3.74579	-0.488	0.89316	-0.2692	-0.5427	-1.1912	-0.8645	-0.5036	0.05184	-0.3974	-0.2139	0.11549	-0.669	0.90294

TCGA-90	-0.3625	0.12731	-0.4831	1.15024	-0.2832	0.81745	-0.2081	-0.4735	3.21299	-0.5416	-0.0908	-0.2692	0.75737	1.21502	-1.4232	-0.5036	-0.3588	-0.3974	-0.6123	-0.1189	-0.669	-1.1785
TCGA-96	-0.3625	1.14771	-0.71	1.04699	-0.2832	-0.5786	-1.1257	-0.4735	2.26561	-0.2867	0.88767	-0.2692	-0.3852	-0.895	-0.2145	-0.5036	0.71231	0.41344	-0.1638	-0.5744	-0.669	0.95064
TCGA-34	-0.3625	1.45314	-0.2699	1.16119	-0.2832	0.12166	0.87066	-0.4735	-0.393	-0.1054	-0.6506	-0.2692	-0.3267	-0.5517	-0.3283	-0.5036	-0.4168	-0.1641	-0.0823	-0.5924	-0.2831	0.30657
TCGA-56	-0.3625	-0.769	-1.0809	0.45229	-0.2832	-0.8956	-0.0881	-0.4735	-0.2003	-0.2933	2.46573	-0.2692	-0.2725	-0.7761	3.87901	-0.5036	0.23329	-0.3974	-0.6123	1.29697	0.24132	-0.9367
TCGA-21	-0.3625	-0.0595	0.04994	1.0835	-0.2832	-0.2871	-0.9883	-0.4735	-0.5526	-0.5416	-0.2493	-0.2692	0.77039	0.7386	0.13521	-0.5036	-0.0494	-0.3974	0.64674	-0.5924	1.31668	-1.187
TCGA-22	-0.3625	-0.4284	-0.3278	1.6939	-0.2832	-0.5889	1.34052	-0.4735	5.91602	-0.5416	-0.6752	-0.2692	0.44504	2.01149	-1.4656	-0.5036	-0.3841	-0.3974	-0.6123	0.63884	-0.669	-1.0218
TCGA-77	-0.3625	0.9812	0.15826	1.81343	-0.2832	-0.8617	-1.1257	-0.4735	0.08067	-0.5416	0.33659	-0.2692	-0.7209	0.46711	-0.87	-0.5036	1.35786	-0.3471	-0.548	1.48581	-0.669	0.21927
TCGA-L3	-0.3625	-0.1566	-0.5289	-0.279	-0.2832	-0.4071	0.07351	-0.4735	-0.5526	0.52489	-0.8413	-0.2692	0.75816	-0.5033	-0.3747	0.48395	-0.8948	-0.3974	0.68908	-0.5924	1.45484	1.00139
TCGA-60	-0.3625	-0.1785	2.44186	1.08838	-0.2832	1.45149	-1.1257	-0.4735	-0.5526	-0.2998	-0.2972	-0.2692	-0.7723	-0.0466	-0.8432	-0.5036	0.15831	0.22058	-0.6123	-0.5924	2.07166	-0.7497
TCGA-37	-0.3625	-0.0248	1.37304	-1.3487	-0.2832	2.20421	0.69481	0.35463	-0.5526	1.1124	0.14103	0.33982	-1.5147	0.23836	0.48793	-0.1024	-0.9589	-0.3974	2.05787	-0.5924	-0.4564	-0.7021
TCGA-66	-0.3625	2.70996	-0.7223	-0.5702	-0.2832	-0.7585	-1.125	-0.4735	0.46318	-0.5416	-0.8413	-0.2692	0.29459	-0.3454	0.23417	-0.5036	0.77238	-0.3974	-0.0697	0.02925	-0.6485	0.55845
TCGA-63	-0.3625	-0.1364	1.37955	-0.8528	-0.2832	1.91325	-0.4812	-0.4735	1.1231	-0.3056	1.28155	-0.2692	-0.6009	-0.1948	-0.1614	-0.5036	0.69793	2.00595	-0.4105	0.79072	-0.669	-1.0811
TCGA-L3	-0.3625	-0.1444	-0.3903	-0.7942	-0.2832	0.06294	1.19897	-0.4735	1.21921	-0.5416	-0.8413	-0.2692	2.0922	-0.2113	1.0494	-0.5036	-0.5898	-0.3974	-0.6123	-0.5924	-0.5619	-1.1036
TCGA-98	-0.3625	3.88571	-0.8495	0.23559	-0.2832	-0.1455	-1.0569	-0.4735	-0.3545	-0.4517	-0.8413	-0.2692	-0.0591	-1.1208	-0.4101	-0.4813	-0.0183	-0.3196	-0.6123	-0.5924	-0.669	0.76535
TCGA-77	-0.3625	1.25621	-0.252	-1.0696	-0.2832	0.79566	0.34441	-0.4735	2.76615	-0.5416	-0.0806	-0.2692	-0.7453	0.96212	-0.799	-0.5036	0.38235	-0.3713	-0.6123	-0.5924	0.15969	0.93435
TCGA-66	-0.3625	-0.1428	1.20368	-1.1588	-0.2832	2.22643	-0.3602	-0.2488	-0.5526	-0.5416	0.89708	-0.2692	-0.9417	-0.0344	1.01135	-0.5036	0.16574	-0.3974	-0.5505	-0.5924	-0.0887	-0.1076
TCGA-77	-0.3625	1.59043	-1.0809	0.07638	-0.2832	-0.4082	-0.6341	-0.4735	0.47013	-0.5416	-0.8413	-0.2692	1.62188	-1.1743	0.31838	0.28129	-0.9589	-0.3974	-0.5485	-0.5924	-0.669	0.00163
TCGA-63	-0.3625	0.0552	-1.0809	0.16359	-0.2832	-1.0183	0.03703	-0.4735	-0.5526	2.97096	-0.8413	-0.2692	1.49654	-0.5445	-0.8083	0.27726	-0.9589	-0.3974	-0.5429	0.31109	-0.669	0.50923
TCGA-66	-0.3625	-0.8847	-0.5572	-0.7302	-0.2832	0.63882	-0.3817	-0.4735	-0.5526	1.00767	-0.8413	-0.2692	0.58074	0.02488	1.41014	1.76446	-0.9589	-0.3974	1.26487	0.38774	-0.1824	-1.268
TCGA-77	-0.3625	1.62819	-1.0809	-1.0658	-0.2832	-0.178	0.00372	-0.4735	1.5098	-0.5159	-0.8413	-0.2692	1.14941	-1.2942	-0.0069	-0.5036	0.73313	-0.3974	0.17493	0.16321	-0.669	0.46572
TCGA-43	1.16447	-0.8234	-0.8692	0.67952	-0.2832	-0.3522	0.59066	-0.4735	-0.5526	2.28527	-0.6596	-0.2692	0.21453	-0.6891	-0.1672	-0.5036	1.39349	-0.3974	1.62646	0.43584	-0.669	-0.5984
TCGA-22	-0.3625	0.04664	0.06091	-1.5382	-0.2832	1.16508	-0.3512	-0.4735	3.43018	-0.4955	-0.7221	-0.2692	-0.7787	-0.0331	-1.4871	-0.5036	0.33623	-0.3974	-0.471	-0.4736	-0.2314	3.55414
TCGA-66	-0.3625	-0.4397	-0.5045	-0.6464	-0.2832	-0.2545	-0.2493	-0.4735	-0.2082	1.3589	-0.8413	-0.2692	0.86201	-0.0783	0.79881	-0.3994	-0.6005	-0.3974	0.16816	-0.1383	-0.4693	0.70312
TCGA-77	-0.3625	0.3584	-0.0214	0.59639	-0.2832	1.02965	-0.1516	-0.0151	1.26131	-0.5416	-0.5968	-0.2692	0.46223	-0.0601	-0.8164	-0.5036	0.24861	-0.3974	-0.6123	-0.3689	-0.669	-0.3628
TCGA-66	-0.3625	0.75705	0.35046	0.67617	-0.2832	1.27213	-0.8897	-0.4735	-0.4497	-0.5416	-0.6913	-0.2692	-0.5892	1.17809	-0.5703	-0.5036	-0.3704	-0.3974	-0.2562	0.51189	-0.669	-0.1124
TCGA-98	0.6478	-0.4845	-0.7844	1.3434	-0.2832	-0.3781	0.93101	-0.4735	0.81384	-0.5416	-0.6222	-0.2692	1.18716	0.18524	-0.133	-0.5036	0.59671	-0.3974	-0.6123	0.57198	-0.669	-1.2705
TCGA-58	-0.3625	0.38122	-0.0241	1.33829	-0.2832	0.83701	0.54327	-0.3443	-0.5526	-0.4384	1.7436	-0.2692	-0.6016	0.04491	-0.8245	-0.5036	0.43569	-0.3974	-0.6123	0.09433	-0.669	-0.5849
TCGA-O2	-0.3625	0.86236	0.03579	0.79433	-0.2832	-0.2974	0.57336	-0.4735	-0.5526	-0.5416	-0.2869	-0.2692	-0.4178	1.47652	-0.128	-0.5036	0.56065	1.13018	-0.6123	0.81822	0.27862	-1.0137
TCGA-63	-0.3625	0.23945	0.09046	0.90467	-0.2832	0.09821	-1.1257	-0.4735	-0.1812	-0.4146	-0.8413	-0.2692	-0.1289	-0.9132	0.17794	-0.5036	0.67429	-0.2078	-0.4359	-0.5924	0.20147	1.32488
TCGA-37	-0.3625	-0.0653	0.15388	-1.5382	-0.2832	1.3205	0.48873	0.23092	-0.5526	-0.5416	1.93127	-0.2692	-1.1966	1.1764	2.39081	-0.5036	0.00842	-0.2581	-0.4728	-0.5924	-0.669	-0.6165
TCGA-66	-0.3625	0.24383	0.22257	0.31155	-0.2832	0.11135	-0.3537	-0.4735	-0.5526	-0.5416	1.76205	-0.2692	-0.9893	0.1168	0.66726	-0.5036	0.95241	0.37856	-0.3063	-0.3527	-0.6219	0.37168
TCGA-34	-0.3625	1.74966	-0.9496	-0.4543	-0.2832	-0.6952	-0.8017	-0.2929	0.46012	-0.5416	-0.8413	-0.2692	0.39012	-0.1178	1.70195	-0.5036	0.09944	-0.3974	-0.5552	-0.0111	-0.669	0.26476
TCGA-NC	-0.3625	0.06311	-0.6262	-1.3047	-0.2832	-0.4558	1.82012	-0.4735	-0.5526	4.99706	-0.8413	-0.2692	-0.2243	-1.4077	-1.2083	-0.5036	1.21389	-0.3974	-0.2973	1.38834	-0.669	1.76864
TCGA-56	-0.3625	-0.3138	-0.295	-0.7538	-0.2832	0.22988	0.75382	-0.4735	0.79334	-0.5416	0.70025	-0.2692	0.90736	0.35822	0.92533	-0.5036	0.94416	-0.3974	-0.5656	-0.5924	0.23395	-1.2373
TCGA-18	-0.3625	0.25594	0.15009	1.8189	-0.2832	1.41245	-0.9508	-0.3762	-0.5526	-0.5416	0.26491	-0.2692	-1.0984	0.98724	-0.3159	-0.5036	-0.2467	1.89088	-0.3288	-0.5924	1.25492	-0.9574
TCGA-56	-0.3625	0.96067	-0.6932	0.61457	-0.2832	0.10753	-0.2056	-0.4735	-0.5526	-0.5416	-0.8413	-0.2692	0.8161	-1.137	-0.6373	0.32702	-0.9589	-0.3974	0.30716	-0.4239	-0.669	0.87916
TCGA-33	-0.3625	-0.0464	0.53144	1.92241	-0.2832	0.19076	-1.1257	0.60699	-0.5526	3.13433	-0.8413	-0.2692	-0.0516	-0.3261	-0.8394	-0.5036	-0.1101	-0.3974	-0.5504	-0.4089	-0.6647	-0.7054
TCGA-43	0.93699	-0.905	-0.7831	-0.083	-0.2832	0.56498	1.21406	-0.4735	1.9738	-0.5416	-0.8413	-0.2692	1.20148	-0.602	-0.0443	-0.5036	0.61009	-0.2862	-0.6123	0.82015	-0.669	-0.5876
TCGA-77	0.88397	0.57786	-0.016	1.23352	-0.2832	0.98796	-0.828	-0.4735	2.47381	-0.4019	-0.3012	-0.2692	-0.6071	-1.1854	-0.5505	-0.5036	-0.1789	1.97595	0.5516	-0.5902	-0.669	-0.0046
TCGA-33	-0.3625	-0.443	2.96383	-1.0587	-0.2832	3.15619	-1.1257	-0.4735	-0.5526	0.24656	0.17078	-0.2692	-0.6586	0.23644	-0.9432	-0.5036	-0.0853	-0.0053	-0.6123	-0.5924	2.07875	-1.2705
TCGA-46	-0.3625	0.41599	-1.0809	-1.5382	-0.2832	0.63774	-0.6535	-0.4735	2.09846	-0.5416	-0.8413	-0.2692	2.60501	-0.6856	-0.1055	-0.5036	0.38333	-0.3974	-0.354	-0.4158	0.7651	-1.048
TCGA-18	-0.3625	1.46312	-1.0576	0.15682	-0.2832	1.64397	0.57806	-0.4282	-0.5526	-0.0297	0.87069	-0.2692	-1.383	-0.6879	-0.6258	-0.5036	0.64432	-0.3974	4.26265	0.09184	-0.669	-0.7323
TCGA-63	-0.3625	4.74361	-0.1054	-1.5382	-0.2164	-0.3326	-1.1257	-0.4735	2.92706	-0.3233	-0.8413	-0.2692	-0.9997	-1.0371	-1.9717	0.9944	-0.9589	-0.3974	7.13512	-0.5924	0.10864	-0.4435
TCGA-56	-0.3625	0.3052	-1.0809	1.59132	-0.2832	-0.5973	0.95794	-0.4735	-0.5526	1.75073	-0.8413	-0.2692	0.70889	-0.8786	-0.5022	2.55021	-0.9589	-0.3974	-0.0553	-0.5924	-0.669	-1.1141
TCGA-60	-0.3625	0.11526	-0.1224	0.66299	-0.2832	0.37997	-0.4905	-0.4735	0.77455	-0.5416	-0.8413	-0.2692	0.14152	0.43149	-0.7461	-0.5036	-0.2956	-0.3974	-0.6048	-0.5924	0.24881	1.09815
TCGA-60	-0.3625	-0.5891	0.59535	-0.2996	-0.2832	1.63024	-0.22	-0.4735	0.97147	-0.0531	0.13362	0.98268	-0.248	-0.45	0.66771	-0.5036	0.36314	3.30418	0.10333	-0.5924	0.29928	-1.114
TCGA-52	-0.3625	0.76659	-1.0792	0.76129	-0.2832	1.17935	2.08042	-0.4735	-0.5526	-0.5067	-0.3705	2.3729	-1.0509	-0.1739	-0.1633	-0.5036	0.83385	0.60835	-0.6123	1.7829	-0.669	-0.3711
TCGA-34	-0.3625	0.99998	-0.2874	1.05363	-0.2832	0.08533	1.34551	-0.4735	4.00515	1.02768	1.01341	-0.2692	-0.6412	-0.325	-1.4983	-0.5036	0.25944	-0.3974	-0.6123	-0.5924	-0.669	-0.2226
TCGA-18	-0.3625	1.45933	-0.5516	0.66424	-0.2832	1.15375	-0.1342	-0.4735	-0.5526	-0.5416	-0.8413	-0.2692	-0.3398	-0.8832	-0.6878	-0.5036	0.72307	-0.3974	-0.5731	0.87184	-0.669	0.45089
TCGA-37	-0.3625	0.93683	-0.4227	-0.2941	-0.2832	0.60173	1.05382	-0.4735	2.55385	-0.5416	0.68972	-0.2692	-0.1202	-0.0957	-0.8203	-0.5036	0.26785	-0.3974	-0.6123	-0.3349	-0.669	0.28138
TCGA-85	-0.3625	1.48117	1.21377	-0.6305	-0.2832	1.36297	1.59505	-0.4735	-0.2095	-0.5416	0.36036	-0.2692	-1.3014	-0.2373	-0.0105	-0.4818	-0.9287	0.67398	-0.6123	1.08036	-0.669	-0.1744
TCGA-60	-0.3625	1.77677	0.09148	-1.4398	-0.2832	0.22597	-0.3225	-0.3408	2.53315	-0.3808	-0.559	-0.2692	0.46148	-0.3239	-1.0776	-0.5036	0.15187	-0.3974	-0.4289	-0.164	-0.669	0.95294
TCGA-60	-0.3625	0.76447	-0.5067	-0.1271	-0.2832	0.29518	-0.2449	-0.4735	1.41968	0.07436	0.58943	-0.2692	0.8306	-0.5553	-0.9568	-0.5036	0.82394	-0.162	-0.5873	-0.3865	-0.5549	-0.2
TCGA-94	-0.3625	1.04335	-0.4455	-0.2321	-0.2832	-0.6127	-0.9693	-0.4735	-0.2095	-0.5416	0.3768	-0.2692	0.29221	-0.7098	-1.0101	1.14507	-0.9589	-0.2591	1.19769	0.41587	-0.669	1.2918
TCGA-22	-0.3625	0.77487	-1.0809	3.16796	-0.2832	0.86077	-1.1257	-0.4735	-0.5526	-0.5416	-0.8413	-0.2692	0.55696	-1.2687	-1.1759	1.61465	-0.9589	-0.3974	0.4397	-0.5924	-0.669	-1.0727
TCGA-18	-0.3625	-0.4597	1.61885	-0.3749	-0.2832	1.31122	-0.7748	-0.4735	0.07457	0.24097	-0.8244	-0.2692	-0.3353	0.13192	0.19605	-0.5036	-0.789	-0.3974	1.1678	-0.5924	0.87221	-0.1686
TCGA-66	-0.3625	0.62902	-0.9838	-1.5382	-0.2832	1.16096	1.63418	-0.4735	0.13671	-0.5416	-0.8413	-0.2692	0.73669	-0.3658	0.51003	-0.5036	-0.0672	-0.3974	-0.3537	0.36421	-0.669	0.14886
TCGA-21	-0.3625	-0.905	-1.0315	1.30624	-0.2832	-0.621	-1.1257	-0.4735	-0.5526	-0.3138	-0.6211	-0.2692	1.07057	-0.8037	1.97346	0.66147	-0.9589	-0.3974	-0.4839	1.17678	-0.669	-0.1713
TCGA-92	-0.3625	-0.2152	1.93662	-0.2311	-0.2832	2.85286	-0.0457	-0.4735	-0.5526	-0.4683	-0.5992	-0.2692	-1.0022	-0.159	-0.8111	-0.5036	0.36935	0.29549	-0.6123	-0.5924	1.06194	-0.1436
TCGA-63	-0.3625	-0.648	0.45317	0.77826	-0.2832	1.56295	0.29562	-0.4735	0.1326	3.19938	-0.5536	-0.2692	-0.9361	-0.5822	0.09673	-0.5036	0.64343	0.01952	-0.6123	0.70748	-0.669	-0.8888
TCGA-60	-0.3625	-0.1896	0.69755	-0.0124	-0.2832	0.96873	-0.9757	-0.4735	1.60755	-0.5416	-0.0775	-0.2692	-0.2253	0.32893	0.248	-0.5036	0.60157	4.77147	-0.5375	-0.5924	1.52487	-1.2041
TCGA-98	-0.3625	0.06957	1.32528	0.65788	-0.2832	0.15635	-1.1257	-0.4735	-0.5526	-0.3567	0.0141	-0.2692	-0.6836	0.62464	-0.5317	-0.5036	2.18436	0.57581	0.06453	-0.5924	1.02351	-0.4811
TCGA-37	-0.3625	0.50053	0.04119	-0.1109	-0.2832	1.31652	0.35189	-0.4735	-0.1605	-0.5416	0.10418	-0.2692	-0.5651	-0.3452	0.7076	-0.5036	-0.1331	0.39592	-0.5505	-0.5924	-0.669	0.33428
TCGA-22	-0.3625	-0.107	0.74503	1.15203	-0.2832	1.80075	0.89517	-0.4735	-0.5526	-0.2856	0.28616	-0.2692	-0.5419	-0.4574	-0.3989	-0.5036	0.24441	0.98845	-0.298	-0.3204	0.10183	-1.234
TCGA-33	-0.3625	1.13703	-0.3827	0.14826	-0.2832	0.24473	-1.0872	0.29116	-0.5526	0.95415	2.39534	-0.2692	-0.008	-0.6505	-0.8019	-0.5036	0.03744	-0.0858	-0.6123	-0.5924	-0.669	-0.0676
TCGA-77	-0.3625	-0.3493	-1.0809	-0.0771	-0.2832	-0.0769	0.39837	-0.4735	0.53856	-0.5416	-0.6371	-0.2692	1.49796	-0.3414	1.81685	-0.5036	0.84131	-0.3974	-0.6123	-0.3994	-0.1013	-1.1163
TCGA-56	-0.3625	1.1517	-0.3164	1.50495	-0.2832	0.33484	-1.1257	-0.4735	1.35381	-0.5416	0.09401	-0.2692	-0.2734	0.04854	-0.6592	-0.5036	0.4979	-0.3974	-0.4359	0.31816	-0.669	-0.3508
TCGA-56	-0.3625	-0.6014	2.30304	-0.0922	-0.2832	0.80309	-0.1013	-0.4735	-0.5526	0.6072	-0.7313	-0.2692	-0.2913	0.55245	-0.1823	-0.5036	-0.5255	0.69635	-0.477	-0.5924	3.24208	-1.0309
TCGA-37	-0.3625	-0.5847	1.28616	-1.5382	-0.2832	4.10675	0.94915	2.88234	-0.5526	-0.5416	0.11948	-0.2692	-0.9548	0.07352	-0.997	-0.5036	0.36618	-0.3974	-0.4016	0.30798	-0.669	-1.2357
TCGA-58	-0.3625	0.82514	-0.5262	-0.4147	-0.2832	0.48561	0.86668	-0.4735	1.20192	1.19751	0.08654	-0.2692	-0.8631	-0.9129	0.75987	-0.5036	2.06511	0.50068	1.4562	-0.2627	-0.669	-0.8295
TCGA-43	-0.3625	1.14192	-1.0809	0.34303	-0.2832	-0.5376	0.00702	-0.4735	1.44262	-0.5416	-0.7741	-0.2692	1.20983	-0.5963	0.13585	-0.5036	0.41758	-0.3974	-0.6123	-0.1988	0.95763	-0.5811
TCGA-77	-0.3625	1.62782	-1.0325	0.26595	-0.2832	-0.021	-0.4015	-0.4735	0.92015	-0.5416	2.86924	-0.2692	-1.5147	-0.051	-0.5547	-0.5036	1.1026	2.34751	-0.4203	1.32753	-0.669	0.35003
TCGA-39	-0.3625	0.86164	-0.6009	0.75516	-0.2832	-0.4217	0.16381	-0.4735	-0.5526	-0.5416	-0.4448	-0.2692	0.27861	0.27397	-0.4744	-0.5036	0.26834	-0.3974	-0.6079	-0.5924	-0.669	0.9795
TCGA-90	-0.3625	0.05973	0.78747	-0.8782	-0.2832	1.78011	-0.5509	-0.4735	0.22926	-0.5416	-0.0783	-0.2692	-1.3205	-0.4009	-0.9438	-0.5036	0.91762	-0.3974	2.77432	0.86628	-0.669	1.08243
TCGA-68	-0.3625	-0.1177	0.2175	2.19933	-0.2832	0.27444	-0.2616	-0.4735	-0.5526	0.14163	2.77095	0.68968	-0.9834	0.04455	-0.6868	-0.5036	0.94213	-0.0714	-0.6123	-0.4801	0.16988	-0.9256
TCGA-66	0.40302	-0.609	0.68562	-0.4468	-0.2832	1.89619	1.12896	-0.4735	1.35206	-0.5416	0.17497	-0.2692	-0.522	-0.3769	0.55307	-0.5036	0.37717	0.68979	-0.143	-0.5924	-0.1835	-0.8306
TCGA-66	-0.3625	0.11419	0.38179	-0.8797	-0.2832	0.59513	0.16626	-0.1388	-0.5526	-0.5416	1.07676	-0.2692	-1.5147	-0.0011	1.09848	-0.5036	0.49247	-0.087	1.89054	-0.0901	-0.669	0.84527
TCGA-85	-0.3625	0.56836	0.3792	0.02696	-0.2832	1.14916	-0.7358	-0.4735	-0.5452	-0.5416	-0.3022	-0.2692	0.32399	0.00276	-0.3486	-0.5036	0.22202	0.22806	-0.6123	-0.0416	-0.669	-0.2707
TCGA-56	-0.3625	1.87306	0.11364	-1.4222	-0.2832	0.52402	0.5943	-0.4735	-0.5526	0.04543	-0.7536	-0.2692	-0.4942	-0.1358	-0.6767	-0.5036	-0.9589	-0.2161	-0.5153	0.60383	-0.669	1.949
TCGA-56	-0.3625	0.07718	-1.0809	-0.9864	-0.2832	-0.9391	0.04428	-0.4735	0.30812	0.22641	-0.8413	-0.2692	3.86057	-1.4698	-0.202	0.03316	-0.9589	-0.3974	-0.6123	-0.5924	1.19693	-0.9215
TCGA-18	-0.3625	0.14345	0.66994	-1.5382	-0.2832	3.23417	-0.3939	-0.4735	-0.5526	1.00132	0.18129	-0.2692	-0.9079	0.18129	0.95749	-0.5036	0.51565	0.8202	-0.5351	-0.5924	-0.669	-1.1643
TCGA-51	-0.3625	0.15445	0.1589	1.31258	-0.2832	1.51789	0.79215	-0.4735	-0.5526	0.03612	-0.7558	-0.2692	-1.1907	1.112	-0.5636	-0.5036	0.72842	1.61911	-0.6123	1.33874	-0.4119	-1.1273
TCGA-NC	0.00826	-0.4525	0.59327	-0.5648	-0.2832	0.99777	-0.0814	-0.4735	2.95608	-0.5416	-0.792	-0.2692	0.47391	0.17826	1.2656	-0.5036	-0.4336	-0.3974	-0.6123	-0.5924	1.1327	-1.2705
TCGA-77	-0.3625	0.82283	-0.7468	0.59277	-0.2832	-1.1028	0.03924	-0.4735	1.861	-0.5416	-0.8413	-0.2692	2.1648	-0.3535	0.02224	-0.5036	-0.1179	-0.3974	-0.6123	-0.5924	0.65167	-1.2705
TCGA-96	-0.3625	0.45465	-0.6481	1.2281	-0.2832	0.57995	-1.1257	-0.4735	-0.5526	-0.0278	1.64014	1.07263	-0.9425	-0.8372	1.26288	-0.5036	2.326	1.01598	-0.0333	-0.5924	-0.0042	-1.2705
TCGA-NC	-0.3625	1.94128	-1.0253	-0.3257	-0.2832	-0.0715	0.38741	-0.4735	-0.4145	-0.5236	-0.8413	-0.2692	0.19343	0.7624	-1.2597	0.64687	-0.9589	-0.3974	-0.1404	1.81209	-0.669	0.27565
TCGA-33	-0.3625	-0.8827	1.82123	-1.5382	-0.2832	1.94182	-1.0792	-0.4735	-0.5526	-0.5416	-0.0618	-0.2692	-0.479	1.28368	1.16613	-0.5036	0.10429	-0.2726	-0.398	-0.5924	2.30381	-0.9379
TCGA-85	-0.3625	4.07239	-1.0809	-1.2111	-0.2832	1.50296	3.02054	-0.4735	-0.5526	1.59761	-0.8413	0.10588	-1.1474	-1.6092	-1.2086	-0.5036	0.21963	6.32852	-0.6123	-0.5924	-0.669	-0.3815
TCGA-60	-0.3625	0.32824	0.75786	-0.3501	-0.2832	1.64969	1.10622	-0.3954	-0.1581	0.23946	-0.1614	-0.2692	-1.1387	-0.7471	-0.3063	-0.5036	1.12412	-0.3974	-0.6123	0.19401	-0.669	0.55577
TCGA-33	-0.3625	-0.2322	0.09192	-0.0203	-0.2832	1.17486	0.53993	-0.4735	-0.5526	0.01325	0.62628	-0.2692	0.25322	-0.7639	0.97067	-0.4469	0.69172	-0.1119	-0.3454	-0.5924	-0.3581	-1.143
TCGA-NC	-0.3625	0.06687	-0.3652	-0.6698	-0.2832	2.24398	0.20779	-0.4735	0.47104	-0.5416	0.60358	0.84772	-0.8673	0.47191	1.48102	-0.5036	-0.0596	0.7199	-0.5418	-0.5783	0.18065	-0.8986
TCGA-21	-0.3625	0.4687	2.569	-1.4728	-0.2832	3.33603	1.05445	-0.4735	-0.5526	-0.2096	-0.7566	-0.2692	-1.126	-1.166	-1.0913	-0.5036	1.46464	0.37009	-0.4639	1.01577	-0.669	-0.5252
TCGA-85	-0.0849	-0.905	-1.0231	-1.5382	-0.2832	-0.8801	0.87452	0.90153	0.64348	3.3914	-0.8413	0.39599	3.4107	-1.7519	-0.6072	1.13473	-0.9589	-0.3974	-0.3336	-0.5924	-0.669	-1.1974
TCGA-34	-0.3625	-0.4708	-0.141	-0.6864	-0.2832	0.03288	-0.4548	0.45559	-0.5526	-0.5416	-0.4831	-0.2692	1.38777	-0.9469	2.74047	-0.5036	0.63058	-0.3974	-0.3154	-0.5924	-0.669	-1.0732
TCGA-77	-0.3625	-0.4132	-0.8141	-0.6024	-0.2832	0.39132	0.23696	-0.4735	2.98489	-0.5416	-0.829	-0.2692	1.02675	0.29752	1.23267	-0.5036	0.11011	-0.3974	-0.6123	-0.3383	-0.5992	-0.361
TCGA-56	-0.3625	-0.7214	-0.976	1.13066	-0.2832	-0.3146	-0.2519	-0.4735	-0.5526	-0.5416	1.52304	-0.2692	0.73059	-1.0516	0.28864	-0.5036	2.27356	1.64964	-0.4868	-0.5924	0.73879	-0.6498
TCGA-33	-0.3625	1.08076	-1.0809	0.05353	-0.2832	0.08897	0.81958	-0.4735	0.92245	-0.3175	-0.2131	-0.2692	0.11787	-1.4649	-1.0535	0.59886	-0.9589	0.44399	-0.5133	-0.5924	-0.669	2.1444
TCGA-39	-0.3625	0.63842	0.16914	0.0125	-0.2832	0.60438	0.29217	-0.4735	-0.5526	-0.1463	1.47786	-0.2692	-0.3522	0.38449	-0.6864	-0.5036	1.15597	-0.3974	-0.6123	1.67948	-0.669	-1.0501
TCGA-56	-0.3014	-0.747	-0.6247	0.13446	-0.2832	-0.6904	-0.5279	-0.4735	1.0262	-0.5416	-0.4869	-0.2692	2.17056	1.00102	-0.3179	1.02171	-0.9589	-0.3974	-0.0187	-0.5924	0.73006	-1.2334
TCGA-56	-0.3625	-0.4546	-1.0809	0.11653	-0.2832	0.40331	-1.1257	-0.4735	-0.0857	-0.5416	-0.5356	1.32896	1.56006	-1.2303	0.93646	-0.0675	0.16726	1.05839	0.87092	-0.5924	0.70769	-0.866
TCGA-77	-0.3625	2.08922	-1.0809	-0.8031	-0.2832	-0.0663	-0.0775	-0.4735	-0.5526	0.39733	3.98591	3.06204	-0.5397	-1.4261	1.19516	0.08859	-0.5948	0.26414	-0.4418	-0.5924	-0.669	-0.7238
TCGA-85	-0.3625	-0.5793	-1.0236	0.38411	-0.2832	-0.3237	-0.773	0.08604	-0.4035	1.54328	-0.8413	-0.2692	-1.0697	-0.9926	-0.9182	-0.5036	3.51862	-0.3974	7.00562	4.8182	-0.669	-0.9529
TCGA-77	-0.3625	-0.8649	-1.0809	2.06402	-0.2832	-0.3633	-1.1257	-0.4735	-0.5526	-0.5416	0.05372	-0.2692	1.74337	0.19395	-1.0762	2.10201	-0.9589	-0.3974	-0.251	-0.5858	0.43385	-1.1771
TCGA-85	0.52885	-0.2953	-1.0809	1.55158	-0.2832	0.00359	-0.9462	-0.4735	-0.5526	1.9092	-0.8413	-0.2692	1.27109	-1.414	-1.437	2.31299	-0.9589	-0.3974	-0.6123	-0.5924	0.925	-0.782
TCGA-XC	-0.3625	1.6838	-0.566	1.3912	-0.2832	-0.7406	-0.7066	-0.4735	0.21603	0.80901	-0.8413	-0.2692	1.18369	-1.7519	-0.3546	-0.5036	-0.5767	-0.3974	-0.3367	-0.5924	0.3559	-0.32
TCGA-66	-0.3625	0.44899	-0.0351	0.05768	-0.2832	0.02979	-0.0952	-0.4735	0.20242	-0.5416	0.54667	-0.2692	-1.1076	0.16036	1.15818	-0.5036	0.4375	-0.3974	2.69523	-0.5877	-0.1541	-0.0511
TCGA-68	-0.3625	2.34695	-0.5296	0.22644	-0.2832	0.10053	0.18114	-0.3037	-0.2322	-0.3377	-0.8413	-0.2692	0.20374	-1.2077	-0.9893	-0.3371	-0.66	-0.3974	-0.0023	-0.5924	-0.1634	1.03921
TCGA-77	-0.3625	3.8276	-0.7472	-1.1529	-0.2832	1.02768	-0.6243	-0.4735	-0.5526	0.63807	0.23199	-0.2692	-1.159	0.23817	-1.1305	-0.5036	-0.2037	-0.3974	-0.476	-0.4525	-0.669	0.94507
TCGA-90	-0.3625	-0.1309	0.79972	0.32771	-0.2832	2.06191	-0.3092	-0.4735	1.92775	-0.5416	-0.303	-0.2692	-0.0262	0.14293	-0.7569	-0.5036	-0.365	0.26321	-0.6123	-0.5924	0.52809	-0.839
TCGA-98	0.23363	-0.905	0.61096	-0.0428	-0.2832	0.45899	0.22376	-0.4735	3.77369	-0.5416	-0.714	-0.2692	0.79454	0.76002	-0.4076	-0.5036	0.60635	-0.3974	-0.6123	-0.1883	-0.0961	-1.0247
TCGA-34	-0.3625	0.05975	-0.1465	-0.0566	-0.2832	-0.0848	-1.1257	-0.4735	-0.1728	-0.3487	-0.6489	-0.2692	1.6721	-1.1914	-0.318	-0.5036	0.28077	-0.3974	-0.6123	-0.5924	0.13584	0.72623
TCGA-56	-0.3625	0.80408	-0.5228	0.37575	-0.2832	0.06066	-1.0041	-0.4735	-0.5526	-0.5416	-0.2472	-0.2692	0.6799	-0.0135	-0.3951	-0.5036	0.43124	-0.2301	-0.3949	-0.5924	-0.669	0.53847
TCGA-66	-0.3625	1.72844	-0.8984	-0.0558	-0.2832	-0.0044	0.35697	-0.4735	0.40992	-0.3764	0.19072	-0.2692	-0.6604	-0.8388	1.32865	-0.5036	1.60004	-0.2752	-0.4053	-0.1703	-0.669	-0.0355
TCGA-39	-0.3625	0.89822	-0.6969	3.50837	-0.2832	-0.8721	-1.1257	-0.4735	-0.5526	0.6595	4.20366	-0.2692	-0.7216	-1.3143	-1.8388	1.26583	-0.9589	-0.12	-0.6123	-0.5924	0.02101	-0.3822
TCGA-56	-0.3625	-0.5554	-1.0629	2.43599	-0.2832	-0.2776	-1.1257	-0.4735	-0.5526	-0.5416	-0.5778	-0.2692	0.93897	0.3525	-0.2878	-0.5036	0.71268	-0.3974	-0.1317	-0.4104	-0.6344	-0.1298
TCGA-NK	-0.3625	0.64883	-0.9746	0.56906	-0.2832	0.69407	1.17584	-0.4735	0.27051	0.91579	-0.8413	-0.2692	-0.2796	-0.9788	-2.1135	2.438	-0.9589	0.18448	0.95099	-0.5924	2.89114	-0.3655
TCGA-22	-0.3625	0.85901	-0.9159	0.66674	-0.2832	-0.4688	-1.1257	-0.4735	0.05763	2.00749	-0.8413	-0.2692	0.10603	-1.5688	1.53753	-0.5036	0.3081	4.33142	1.9703	-0.3965	-0.669	-1.0428
TCGA-39	-0.3625	0.24525	-0.1122	0.08458	-0.2832	1.46687	0.58188	-0.4735	1.63085	-0.5247	0.16718	-0.2692	-0.8807	1.8687	0.10514	-0.5036	1.06459	-0.3974	-0.6123	-0.4042	-0.669	-1.1404
TCGA-63	-0.3625	-0.57	1.0584	0.83169	-0.2832	0.8729	0.41059	-0.4735	-0.5526	-0.5416	0.46859	1.46182	-0.8448	0.71665	0.93408	-0.5036	-0.9589	-0.3974	-0.1108	2.03261	-0.669	-1.2353
TCGA-33	-0.3625	0.14625	0.8213	-0.3327	-0.2832	0.69001	-0.3593	-0.4735	-0.283	-0.5416	2.75934	2.49616	-1.1943	0.71373	-0.3747	-0.5036	0.65375	2.92183	-0.455	-0.5924	-0.669	-0.1214
TCGA-63	-0.3625	1.42273	-1.0809	0.76391	-0.2832	-1.1028	0.20864	-0.4105	0.01392	-0.5416	-0.8413	-0.2692	1.52365	0.52251	0.0824	-0.5036	-0.2155	-0.3974	-0.1181	-0.5924	-0.3536	-0.9871
TCGA-18	-0.3625	-0.8638	1.05758	-1.5382	-0.2832	5.0866	2.01521	-0.4735	-0.5526	-0.5204	0.43033	-0.2692	-1.5147	-0.2069	0.19104	-0.5036	0.40906	0.57801	-0.4246	0.18555	-0.669	-1.0795
TCGA-66	-0.3625	1.88852	-0.9418	1.29812	-0.2832	-0.1273	-0.7067	-0.4735	-0.5526	-0.5416	-0.2091	-0.2692	-0.7713	-0.8068	0.72049	-0.5036	0.08043	-0.3974	1.62356	-0.3607	-0.669	0.28547
TCGA-63	-0.3625	0.5501	-0.6402	-0.1172	-0.2832	0.82179	-0.1041	-0.4735	-0.3634	0.53779	-0.8413	-0.2692	0.08774	-0.5622	-0.1911	-0.5036	0.4896	-0.3974	-0.2432	0.30264	1.03982	0.17496
TCGA-56	-0.3625	1.02649	-0.6708	0.28026	-0.2832	0.08578	-0.2227	-0.4735	-0.5526	0.31569	-0.8196	-0.2692	1.11674	-1.6528	0.21718	0.00078	-0.4619	-0.3974	-0.5106	-0.5924	-0.669	0.28609
TCGA-18	-0.3625	-0.258	0.56097	1.55357	-0.2832	-0.5033	-1.1257	0.39721	-0.5526	0.58391	3.37242	-0.2692	-1.5147	-0.8182	2.3377	-0.5036	0.82789	-0.3974	-0.397	-0.5924	-0.2534	-0.9916
TCGA-NK	-0.3625	1.86755	-0.9569	-0.9609	-0.2832	0.512	-0.1236	-0.4735	1.33071	-0.5416	-0.8413	-0.2692	1.50168	-0.4818	-1.5722	-0.5036	0.08043	-0.3974	-0.6123	1.34385	-0.669	-0.1431
TCGA-85	-0.3625	0.97795	-1.0809	0.01276	2.27717	-0.57	-1.1257	-0.4735	-0.5526	0.87392	-0.4708	-0.2692	0.4075	-1.7519	1.22806	0.89133	-0.9589	-0.3974	-0.6123	-0.4032	0.20705	0.64525
TCGA-22	-0.3625	-0.375	2.38036	-0.713	-0.2832	1.49518	-1.1257	-0.4735	-0.5005	-0.5416	0.26766	0.68503	-0.692	0.12208	0.07303	-0.5036	0.10769	-0.3974	-0.3574	-0.5924	1.62755	-0.1397
TCGA-56	-0.3625	0.25347	-0.6626	0.29325	-0.2832	-0.5569	-0.4149	-0.16	-0.5526	-0.5416	-0.1518	-0.2692	1.19535	1.08516	0.64589	0.00131	-0.9589	-0.3974	-0.3859	-0.5924	-0.407	-0.6545
TCGA-56	-0.3625	1.04182	-0.6049	0.73277	-0.2832	-0.2237	-1.1257	-0.4735	1.04309	-0.1677	-0.6902	-0.2692	0.09844	-1.1576	-0.2225	-0.5036	0.68258	3.17223	0.06272	-0.5924	0.07339	0.68045
TCGA-92	-0.3625	2.0086	-0.7741	1.67482	-0.2832	-1.0401	-1.1257	-0.4735	0.60283	-0.5351	-0.8413	-0.2692	0.79078	-1.1577	0.44701	-0.4082	0.21464	-0.3974	-0.5462	-0.5924	0.04745	-0.4274
TCGA-85	-0.3625	0.64549	-0.6328	1.9296	-0.2832	0.26593	-1.1257	-0.4735	-0.5526	-0.5416	-0.8413	-0.2692	0.45243	-0.4676	-0.9018	-0.5036	0.33327	-0.3974	-0.6123	-0.5924	0.29529	0.47206
TCGA-85	-0.3625	0.04074	0.04768	-1.5382	-0.2832	0.1962	1.45766	-0.4735	-0.3959	-0.5416	0.49171	-0.2692	2.26767	-0.6967	-0.7641	-0.5036	0.05659	-0.3974	-0.6123	0.07908	-0.669	-0.5163
TCGA-77	0.12071	-0.7248	-1.0809	4.16249	-0.2832	-0.4584	-1.1257	-0.4735	0.60143	-0.5416	-0.6841	-0.2692	0.61088	-0.11	-0.8079	-0.5036	-0.4054	0.66599	-0.2843	2.89204	-0.669	-1.1938
TCGA-22	-0.3625	0.78872	-0.8035	-0.1112	-0.2832	0.28909	-1.1257	-0.4735	0.30739	-0.5416	0.29709	-0.2692	-0.912	-0.5812	0.8371	-0.5036	1.24783	-0.3204	0.6412	1.91699	-0.669	0.67099
TCGA-33	-0.3625	-0.2804	0.04124	1.06628	-0.2832	0.18213	0.72771	-0.4735	-0.4574	-0.0997	-0.7772	-0.2692	0.77845	0.31059	0.44298	-0.5036	0.02562	-0.3974	-0.6123	-0.5924	-0.2799	-1.0334
TCGA-56	-0.3625	0.15102	-0.5196	-0.2406	-0.2832	0.25021	0.97139	-0.4735	1.73286	0.86516	-0.5933	-0.2692	-0.004	-0.4437	-0.7441	-0.5036	1.95066	-0.3974	0.26583	0.49292	-0.669	0.12086
TCGA-21	-0.3625	0.07087	-0.2397	0.04452	-0.2832	-0.4017	-0.073	-0.0596	0.17029	-0.5416	-0.5636	-0.2692	1.48237	0.31665	-0.0484	-0.5036	0.55663	-0.3974	-0.3829	-0.5924	-0.669	-0.4793
TCGA-6A	-0.3625	-0.905	-0.6416	-0.5799	-0.2832	0.95732	0.99964	-0.4735	-0.3551	-0.5416	-0.4479	-0.2692	2.30879	1.74263	-2.1135	0.99793	-0.9589	-0.3974	-0.6123	-0.4502	0.20737	-1.2632
TCGA-63	-0.3625	0.5402	1.07807	0.63879	-0.2832	0.56596	-1.1257	-0.4735	-0.5526	-0.4286	0.15891	-0.2692	-0.3982	0.05088	0.35313	-0.5036	1.39341	0.1393	-0.6123	-0.3539	-0.3336	-0.86
TCGA-39	-0.011	-0.905	0.36597	-0.8946	-0.2832	1.63559	0.02016	1.26402	1.25478	-0.5416	-0.3193	-0.2692	-0.0996	1.27783	-0.5554	-0.5036	0.01947	-0.3974	-0.6123	-0.5924	-0.669	0.2316

TCGA-43	-0.3625	-0.8512	0.31102	1.43339	-0.2832	0.738	-1.1257	-0.4735	-0.5526	0.74106	0.55747	-0.2692	0.06194	-0.4276	1.76365	-0.4077	-0.9589	-0.3974	-0.4684	-0.5924	0.33691	-1.2479
TCGA-98	-0.3625	-0.905	0.90946	0.78435	-0.2832	0.37031	-1.1257	-0.4735	-0.5526	-0.4054	-0.4418	-0.2692	0.91186	0.79661	-0.0628	-0.5036	-0.2837	-0.3974	-0.4363	-0.5924	1.03782	-0.8138
TCGA-77	-0.3625	0.89474	-0.1798	-0.9	-0.2832	0.4537	0.8095	-0.4735	0.3633	-0.5416	3.75724	-0.2692	-1.1874	1.57772	-0.1471	-0.5036	1.02056	-0.3609	-0.6123	-0.5924	0.13303	-0.8114
TCGA-92	-0.3625	0.38481	-0.2585	1.04275	-0.2832	0.41457	-1.1257	-0.4735	-0.5526	0.05641	-0.408	-0.2692	-0.5901	1.17076	-0.2838	-0.5036	0.87525	-0.2742	0.04099	-0.5924	0.54441	-0.0706
TCGA-56	-0.3625	1.30621	-1.0809	-0.5352	-0.2832	-0.7644	-0.0215	-0.4735	1.14175	-0.3556	0.06283	-0.2692	0.42809	-1.5771	-0.1311	-0.5036	0.33128	-0.3974	-0.4223	-0.0372	-0.669	2.21979
TCGA-70	-0.3625	0.41653	-0.7866	0.93711	-0.2832	-1.0332	-1.1257	-0.4735	0.47691	-0.1905	-0.8413	-0.2692	0.91667	0.24918	0.81738	0.08285	-0.9589	-0.3974	-0.3434	-0.5924	1.48051	-0.2405
TCGA-43	-0.1741	1.20395	0.35346	-0.3814	-0.2832	0.48318	-0.3791	-0.3261	0.09004	-0.5416	-0.8413	-0.2692	-0.7444	-0.6905	-1.2852	-0.5036	-0.1453	0.4679	-0.6123	-0.4675	-0.669	3.10003
TCGA-66	-0.3625	-0.3306	0.68647	-0.7959	-0.2832	0.96073	-0.1977	-0.4735	-0.3283	-0.5416	-0.8413	-0.2692	1.22576	-0.015	0.06864	-0.5036	-0.1466	-0.2761	-0.5873	-0.5924	0.91372	-0.4871
TCGA-85	-0.3625	-0.2789	0.56575	-0.245	-0.2832	1.29807	0.36794	-0.4735	3.2884	-0.2378	3.96991	-0.2692	-1.2187	0.63444	-1.3591	0.22494	-0.9589	0.5173	-0.0726	-0.5924	1.49804	-0.8962
TCGA-58	-0.3625	0.37405	0.74208	-1.4877	-0.2832	0.65551	-0.1074	0.74405	-0.165	-0.5416	-0.0474	-0.2692	-0.7061	0.01488	-1.0709	-0.5036	0.44457	-0.3974	0.06282	-0.3818	-0.669	2.44412
TCGA-85	-0.3625	-0.5009	-0.2035	-1.2535	-0.2832	2.31132	1.87494	-0.4735	-0.5526	2.19134	-0.8413	-0.2692	0.72209	-0.3476	-1.0772	-0.5036	0.90253	-0.3974	-0.6123	0.03256	-0.2269	-1.0157
TCGA-21	-0.3625	0.71774	-0.6326	-0.4011	-0.2832	0.20362	-0.0745	-0.4735	-0.148	-0.5416	-0.8413	-0.2692	0.59481	-0.136	-0.8599	-0.5036	-0.0221	-0.3974	-0.3751	-0.1827	-0.669	1.74207
TCGA-33	-0.3625	1.71488	0.43395	-1.5382	-0.2832	1.31861	1.13558	-0.4735	1.73229	0.68315	-0.8413	-0.2692	-0.8626	1.21381	-1.3259	-0.5036	-0.1392	-0.3974	-0.6123	-0.5924	-0.669	0.6144
TCGA-56	-0.3625	0.14771	0.37904	-0.2713	-0.2832	0.4923	0.03337	-0.4735	1.77475	-0.5416	0.6181	-0.2692	-0.4798	0.20226	0.25161	-0.5036	0.4282	1.27277	-0.5886	-0.4715	0.9578	-0.208
TCGA-34	-0.3625	2.05414	-1.0809	-0.1391	-0.2832	0.87191	2.6733	-0.4735	-0.5526	-0.3026	0.46695	-0.2692	-0.4573	-0.2194	-1.1922	-0.1766	-0.1237	0.45292	-0.2958	0.58603	-0.2942	-0.444
TCGA-60	-0.3625	0.28299	1.95183	1.41305	-0.2832	1.13836	-1.1257	0.00568	-0.5526	-0.5416	-0.2199	-0.2692	-0.7706	0.37037	-1.3077	-0.5036	0.55124	-0.3974	-0.6123	-0.5924	-0.3503	-0.1895
TCGA-77	-0.3625	0.04693	-0.3714	0.9392	-0.2832	0.36496	-0.3341	-0.4735	-0.5526	-0.5416	-0.8413	-0.2692	0.338	-0.494	-1.036	0.46799	-0.7247	-0.3974	-0.4167	-0.5924	2.52383	0.7395
TCGA-66	-0.3625	-0.2032	2.20599	-0.9142	-0.2832	1.90367	-0.8909	-0.2687	0.11309	-0.4824	-0.1778	-0.2692	-0.8774	-0.0141	0.41882	-0.5036	-0.0064	-0.0328	-0.4053	-0.5924	1.31155	-0.15
TCGA-68	-0.3625	0.85131	-0.5831	-0.2057	-0.2832	-0.8535	-0.4094	-0.319	-0.24	0.58538	-0.8413	-0.2692	1.35279	-1.0396	-0.5414	-0.5036	0.77032	-0.3974	-0.3323	-0.1105	-0.669	0.84111
TCGA-94	-0.3625	0.11417	-1.0809	-0.6353	-0.2832	-0.6562	0.18108	-0.4735	-0.4225	0.05189	-0.4003	-0.2692	2.9493	-0.6178	-0.276	-0.5036	-0.1155	-0.3974	-0.0751	0.69444	-0.669	-0.9197
TCGA-56	-0.3625	2.05502	-0.9395	0.98557	-0.2832	0.15503	-1.1257	-0.4735	2.79699	-0.0317	-0.3793	-0.2692	-0.997	-1.0966	-1.1232	-0.5036	1.18056	0.52923	1.11075	-0.059	-0.669	0.96075
TCGA-22	-0.3625	0.38246	0.72238	0.18745	-0.2832	0.86983	0.03002	-0.4735	0.17341	-0.5416	0.20802	-0.2692	-0.6052	0.21886	1.13002	-0.5036	0.54511	-0.3974	-0.3914	-0.4902	-0.669	-0.8061
TCGA-77	-0.2498	-0.8061	-0.7322	0.57767	-0.2832	-0.4858	0.45311	-0.4735	1.16535	-0.3134	0.04457	-0.0919	2.17076	-1.3224	0.0674	1.89327	-0.9589	-0.3974	-0.5127	-0.5924	-0.669	-1.0891
TCGA-21	-0.3625	0.42839	1.52694	-0.8563	-0.2832	2.14672	-0.2077	-0.4735	-0.2921	0.78007	-0.6414	-0.2692	-0.6039	-0.5682	-0.5979	-0.5036	0.55006	-0.3052	0.12271	-0.5924	0.30272	-0.2844
TCGA-98	1.4718	1.12547	-0.3967	0.23713	-0.2832	0.39167	1.10959	-0.4735	3.54883	-0.5416	0.44384	-0.2692	-0.2798	-0.4983	-0.5862	-0.5036	0.24126	-0.3731	-0.6123	-0.1875	-0.5117	-0.8382
TCGA-34	-0.3625	1.13653	-0.9904	0.00283	-0.2832	-0.3871	-1.1257	-0.4735	-0.0157	-0.3618	0.90624	-0.2692	2.1997	-1.3218	-0.0686	-0.2987	-0.9589	-0.3974	-0.6049	-0.5924	-0.669	-0.396
TCGA-21	-0.3625	0.72216	0.87993	-0.8681	-0.2832	0.59537	-0.4316	-0.4735	-0.5526	-0.4728	-0.6566	-0.2692	0.64405	-1.2991	-0.6607	-0.5036	1.18572	-0.3974	-0.6123	-0.0511	0.42036	0.55031
TCGA-60	-0.3625	-0.9044	-1.0809	1.22956	-0.2832	-0.807	-1.1257	-0.4735	-0.5526	0.86898	0.68054	-0.2692	2.75726	-1.7519	0.62335	-0.5036	-0.1981	-0.3974	-0.3042	-0.5924	0.11775	-1.2017
TCGA-96	-0.3625	-0.4719	0.90834	0.17511	-0.2832	0.25773	-0.3805	1.44615	-0.5526	-0.5416	-0.7966	-0.2692	0.60709	0.10235	-0.1041	-0.5036	0.79565	-0.3974	-0.6123	-0.2989	-0.669	-0.3679
TCGA-66	-0.3625	0.17458	-0.2111	0.03063	-0.2832	0.59767	-1.1257	-0.4735	-0.4613	-0.3015	-0.8254	-0.2692	-0.0766	0.92862	-0.2658	-0.5036	0.06562	-0.3974	0.95659	-0.5924	-0.0088	0.72183
TCGA-56	-0.3625	4.14372	-1.0809	0.56456	-0.2832	-0.278	-0.3748	0.76066	-0.5526	-0.0549	-0.4587	-0.2692	-0.2968	-1.4884	-0.4082	-0.5036	1.65467	0.04656	-0.6123	-0.0878	-0.669	-0.8641
TCGA-22	-0.3625	1.43367	-0.0751	0.89495	-0.2832	-0.1445	-1.1257	-0.4735	-0.5526	-0.5416	-0.8413	-0.2692	0.49826	-0.5812	0.7642	-0.393	-0.4331	-0.3974	-0.5325	-0.0756	-0.0379	-0.4536
TCGA-21	-0.2642	-0.905	-0.1708	0.34539	-0.2832	0.62051	0.09527	-0.4735	-0.5526	2.41775	-0.8413	-0.2692	1.61112	-1.1721	-1.3652	1.90341	-0.9589	-0.3974	-0.6123	0.30228	0.21889	-1.2584
TCGA-85	-0.3625	1.7749	-0.4829	0.84188	-0.2832	0.41939	-0.6803	-0.4735	1.11216	-0.5416	-0.8276	-0.2692	0.27409	-0.4229	-0.6131	-0.5036	-0.249	-0.3974	-0.3514	-0.5924	-0.0861	-0.0194
TCGA-21	-0.3625	1.41215	-1.0809	1.82423	-0.2832	-1.0915	-1.1257	-0.4735	-0.5526	-0.3689	-0.8413	-0.2692	1.4381	-0.8132	-0.7006	-0.5036	-0.7447	-0.3974	-0.6123	-0.5924	0.15478	0.55317
TCGA-46	-0.3625	0.78705	-0.5577	1.17738	-0.2832	-1.0685	-0.8343	-0.4735	-0.5526	-0.5416	-0.7326	-0.2692	-0.7109	0.08365	0.56135	-0.5036	0.43318	-0.3974	-0.3475	-0.437	-0.3266	2.20188
TCGA-39	-0.3625	-0.245	0.58366	0.88133	-0.2832	1.02986	-1.1257	-0.4735	-0.2505	-0.3225	-0.1133	-0.2692	0.28745	-0.0254	-0.4271	-0.5036	0.05703	-0.2297	-0.6123	-0.5924	-0.669	0.04081
TCGA-90	-0.3625	1.2996	-0.6325	-0.3267	-0.2832	-0.1701	-0.3309	-0.3542	0.88288	-0.5416	-0.8413	-0.2692	1.19676	-0.9381	-0.114	-0.2612	-0.3303	-0.3974	0.13457	-0.5924	-0.669	0.41397
TCGA-39	-0.3625	0.11384	0.54828	-0.6145	-0.2832	0.92856	0.60145	1.1415	-0.4417	-0.5416	-0.212	-0.2692	-0.6985	1.08476	1.14839	-0.5036	-0.4935	1.9279	-0.416	-0.4418	-0.669	-0.6245
TCGA-39	-0.3625	-0.1309	-0.1999	1.5747	-0.2832	0.31744	-1.1257	-0.4735	-0.3878	-0.3189	0.71243	-0.2692	1.49107	-0.7315	-0.5724	-0.5036	-0.1372	-0.3974	-0.6123	-0.5924	-0.669	-0.8968
TCGA-98	0.69677	-0.6716	-0.1119	0.11931	-0.2832	-0.8023	-1.1257	-0.4735	-0.3421	0.98156	1.48146	-0.2692	1.05622	-1.3685	0.58258	2.29251	-0.9589	0.21642	-0.1196	-0.5572	-0.4481	-1.0771
TCGA-66	-0.3625	1.81516	-0.501	-1.0389	-0.2832	0.08179	-0.2257	-0.4735	-0.5526	0.70081	-0.6469	-0.2692	-0.8945	-1.3082	-1.1263	-0.5036	0.96654	-0.3974	3.95842	0.05322	-0.669	1.86928
TCGA-37	-0.3625	-0.4397	0.29874	-0.1848	-0.2832	2.09891	0.68197	-0.4735	3.92107	-0.5416	-0.8413	-0.2692	-0.1823	0.41154	-0.8887	-0.5036	-0.2919	-0.3974	-0.6123	-0.4704	-0.669	0.23234
TCGA-56	-0.3625	-0.6327	-0.396	2.2723	-0.2832	0.69592	-0.7189	-0.4735	-0.5526	-0.5416	-0.0496	-0.2692	0.62685	0.07721	0.32677	-0.4998	-0.8649	-0.3974	-0.2904	-0.5924	1.09749	-1.2255
TCGA-63	-0.3625	1.46112	0.28276	-1.5382	-0.2832	0.91425	2.71398	-0.4735	-0.2313	-0.5416	1.25746	-0.2692	-0.7081	1.02941	-0.3872	-0.5036	-0.7422	-0.2719	-0.5216	-0.4042	0.38053	-0.3783
TCGA-60	-0.3625	0.10235	-0.1693	-1.5382	-0.2832	0.13415	1.17417	-0.4735	0.83613	-0.5416	-0.1799	-0.2692	0.71291	-0.5458	2.57795	-0.5036	0.42499	-0.3974	-0.6123	-0.5924	0.43533	-0.8587
TCGA-85	0.94067	-0.6458	0.67472	0.55248	-0.2832	1.20413	0.89403	-0.4735	-0.1598	0.11334	-0.4641	-0.2692	0.04302	0.13813	-0.9197	-0.5036	0.69262	0.01139	-0.6123	-0.5924	-0.669	-0.6018
TCGA-56	-0.3625	0.04562	-1.0107	0.12064	-0.2832	-0.9067	-1.1257	-0.4735	-0.5526	-0.5416	-0.7982	5.2798	2.30482	-1.5466	2.00014	-0.1416	-0.9589	-0.3974	-0.6123	-0.5924	2.22033	-1.2042
TCGA-43	-0.3625	0.78965	-0.3836	1.12124	-0.2832	0.79631	-0.4969	-0.4268	-0.1742	-0.1762	-0.5483	-0.2692	0.15213	-0.7648	0.17022	-0.5036	-0.1416	-0.3974	-0.2861	-0.5924	-0.669	-0.072
TCGA-18	-0.3625	-0.5675	0.37263	1.11906	-0.2832	2.44862	-0.2081	-0.4735	-0.0252	-0.5416	0.55786	-0.2692	-0.7318	-0.1847	-0.6884	-0.5036	-0.6605	0.3628	-0.6123	-0.5924	4.15253	-1.0799
TCGA-77	-0.3625	1.03205	-0.5652	1.06137	-0.2832	-0.8273	-0.1257	-0.0136	-0.5526	-0.5416	-0.3839	-0.2692	1.31	0.56396	-0.524	-0.5036	-0.5411	-0.3974	-0.0907	-0.5924	-0.1619	-0.8185
TCGA-60	-0.3625	0.13173	-0.3021	0.23497	-0.2832	0.32282	-0.1684	-0.4735	-0.2915	-0.1184	-0.1473	-0.2692	0.45225	-0.4737	0.58311	-0.5036	0.3362	1.30409	-0.2143	-0.5924	0.44508	-0.4315
TCGA-22	-0.3625	0.85901	-0.8898	1.56144	-0.2832	-0.5381	-0.5966	-0.4735	0.42001	-0.5416	-0.8413	-0.2692	2.11908	-0.5826	-0.0284	-0.5036	-0.779	-0.3974	-0.6123	-0.5924	-0.669	-1.1011
TCGA-66	-0.3625	-0.4108	0.21144	-1.5382	-0.2832	2.63481	0.737	-0.4735	0.34414	-0.3765	-0.617	-0.245	-0.2459	-0.0214	1.15226	-0.5036	0.33775	-0.0768	-0.6123	0.04026	-0.669	-0.3533
TCGA-18	-0.3625	0.24331	-0.0771	0.82217	-0.2832	-0.2325	-0.1031	-0.1506	-0.5526	-0.5416	0.98313	2.53056	-1.2806	0.30407	-0.4898	-0.5036	-0.0749	-0.2103	0.55117	-0.5924	-0.669	1.86672
TCGA-77	-0.3625	0.79361	-0.9413	-0.1619	-0.2832	-0.2794	-0.4326	-0.4735	0.66441	-0.5416	-0.4429	-0.2692	1.22179	-0.3376	-0.363	0.90642	-0.9589	-0.3974	-0.6123	-0.5924	-0.1213	0.4269
TCGA-21	-0.3625	0.52551	0.20646	0.74801	-0.2832	0.17918	1.12051	-0.4735	-0.5526	0.70041	-0.773	-0.2692	-0.7597	-0.0482	-0.3753	-0.5036	-0.5427	-0.3974	0.48813	-0.5924	0.64371	0.64617
TCGA-43	-0.3625	-0.6254	2.47615	-0.9424	-0.2832	2.80013	-0.26	-0.4735	-0.5526	-0.5416	0.16419	-0.2692	-0.5591	0.58745	0.20699	-0.5036	-0.0108	-0.3974	-0.5616	-0.5924	0.21254	-1.2202
TCGA-85	-0.3625	1.44712	-0.6301	1.69999	-0.2832	0.2432	-0.8563	-0.4735	-0.5526	-0.4834	-0.8413	-0.2692	0.58377	0.01091	0.19817	-0.5036	-0.9281	-0.3974	-0.6123	-0.5127	-0.669	-0.6781
TCGA-85	-0.3625	1.2773	-0.8915	0.02676	-0.2832	-0.3355	-0.3414	-0.4735	0.71087	-0.5416	-0.8413	-0.2692	1.14658	-0.3762	0.60327	-0.3409	-0.9589	-0.3974	-0.1176	-0.5924	-0.669	0.09811
TCGA-NC	-0.3625	0.29135	-0.7206	-1.5382	-0.2832	0.01248	1.94721	-0.4735	-0.5526	-0.5416	-0.8413	-0.2692	0.82692	0.56623	-1.1347	-0.5036	2.32213	-0.3974	-0.6123	0.56627	-0.669	0.82836
TCGA-58	-0.3625	0.58303	0.73763	0.46263	-0.2832	1.3243	0.44252	-0.4735	0.22511	0.36073	-0.489	-0.2692	-0.6887	-0.3596	0.27675	-0.5036	0.06036	-0.3974	-0.0743	-0.1886	-0.1047	-0.8649
TCGA-66	-0.3625	0.70232	-1.0809	1.60827	-0.2832	0.55494	-1.1257	-0.4735	-0.5526	-0.5416	0.57604	-0.2692	-1.1072	-0.297	1.0254	-0.4423	-0.1287	0.09843	-0.3704	-0.5924	-0.5232	0.9496
TCGA-LA	-0.3625	-0.6507	0.2611	-1.2398	-0.1306	-0.8618	-1.1257	-0.4735	-0.5526	1.77008	-0.8413	-0.2692	2.36198	-1.7519	1.54484	1.09424	-0.9589	-0.3974	1.10588	-0.5924	0.59825	-1.2484
TCGA-77	-0.3625	-0.3679	-0.2561	0.01534	-0.2832	0.53142	-0.5113	-0.4735	1.48158	-0.5416	-0.8413	-0.2692	0.82088	-0.9155	2.84632	-0.4053	-0.8938	0.42426	-0.0303	-0.5924	0.07684	-1.066
TCGA-85	-0.3625	-0.0494	-0.0772	1.13771	-0.2832	0.1306	0.01499	-0.4735	1.08145	-0.5416	-0.8278	-0.2692	0.33638	-0.7217	0.01353	1.59838	-0.9589	-0.3974	-0.6123	0.06525	0.3341	-0.4681
TCGA-22	-0.3625	0.15995	-0.1976	-1.5382	-0.2832	1.09825	0.09469	-0.4735	2.99547	-0.5416	-0.4707	-0.2692	-0.3611	0.97797	-0.3121	-0.5036	0.36642	-0.3974	-0.4315	-0.5924	0.96271	0.94765
TCGA-77	-0.3625	-0.0899	0.34679	-0.17	-0.2832	0.43446	-1.1257	-0.4735	-0.5526	-0.2695	5.61427	-0.2692	-0.7301	-0.4461	0.18781	-0.5036	0.71701	-0.3974	-0.6123	0.09484	0.57126	-0.8947
TCGA-22	-0.3625	1.17883	-0.1977	-0.3534	-0.2832	0.38539	0.9259	-0.4735	0.33567	1.59015	1.03888	-0.2692	-1.0036	-0.8827	0.00513	-0.5036	0.30103	-0.1675	-0.3731	-0.0124	-0.669	0.42423
TCGA-66	-0.3625	-0.2462	0.92205	-1.5382	-0.2832	3.76843	-0.1718	-0.4735	0.64023	-0.5416	0.99733	-0.2692	-0.7237	0.3304	-0.6273	-0.5036	0.24858	-0.3974	-0.6123	-0.5924	2.16104	-1.181
TCGA-22	-0.3625	-0.5606	0.85652	0.39754	-0.2832	0.32526	1.08412	-0.4735	0.05856	1.50473	1.4858	-0.2692	-0.7302	-0.2988	0.30844	-0.5036	1.53079	-0.3974	0.17861	-0.4201	-0.669	-1.1693
TCGA-77	-0.3625	2.46812	-0.8651	0.3541	-0.2832	-0.054	0.55993	-0.4735	0.07833	0.13492	-0.7528	-0.2692	-0.3791	-1.3176	-0.9062	-0.5036	0.23782	-0.3974	-0.3025	0.72837	-0.669	1.15813
TCGA-56	-0.3625	-0.6279	-1.0809	-0.6043	-0.2832	-1.1028	0.73615	-0.4735	-0.5526	2.35878	-0.8413	1.95006	0.228	-1.7172	-0.6779	8.70903	-0.9589	-0.3974	0.17185	-0.4634	-0.669	-0.6525
TCGA-85	-0.3625	-0.5782	1.06201	-1.0539	-0.2832	2.22761	1.03286	-0.4735	2.8231	-0.5416	-0.3735	-0.2692	0.28858	0.57319	-0.4884	-0.5036	0.03972	-0.3974	-0.5472	0.20413	-0.669	-1.23
TCGA-66	-0.3625	-0.7124	2.45943	-0.5234	-0.2832	2.71563	-0.8062	-0.4735	-0.5526	-0.5416	-0.2712	0.05921	-0.9038	0.17847	-0.0441	-0.5036	0.16461	0.11718	-0.398	-0.5924	1.44227	-0.6341
TCGA-85	-0.3625	-0.6746	0.37002	1.83411	-0.2832	0.51451	0.60963	-0.4735	1.12244	1.94181	-0.8413	-0.2692	0.26869	0.73468	-1.307	-0.5036	-0.2078	-0.3974	-0.6123	0.26009	-0.669	-1.2676
TCGA-39	-0.3625	0.96753	-1.0809	2.09961	-0.2832	-0.2625	-0.0446	-0.4735	-0.5526	-0.2228	-0.333	-0.2692	-0.7676	-0.7316	-1.1402	-0.5036	0.38728	0.27435	-0.6123	0.85914	-0.669	1.87515
TCGA-85	0.21018	-0.7968	-0.2244	-0.3628	-0.2832	-0.249	-0.0261	-0.4735	0.59819	-0.4095	-0.2384	-0.1356	2.36543	-1.3863	1.30062	-0.5036	0.33936	-0.3974	-0.6123	-0.2981	-0.669	-1.0955
TCGA-43	-0.3625	1.22929	-0.7174	0.362	-0.2832	-0.3357	-0.2858	-0.4735	-0.5526	0.63099	0.15651	-0.2692	0.70934	-1.3394	0.40174	-0.2514	-0.6953	-0.3974	-0.1231	-0.5382	-0.5633	0.15778
TCGA-NK	-0.3625	0.93362	-0.7215	-0.2163	-0.2832	0.30504	0.10684	-0.4735	-0.3114	-0.5416	-0.7058	-0.2692	-0.3528	0.18186	-0.3009	-0.5036	0.82018	-0.1237	-0.2321	1.56378	-0.669	0.90628
TCGA-39	-0.3625	-0.2172	1.01207	-1.5382	-0.2832	3.61258	0.34287	-0.4735	-0.4628	-0.4243	-0.0679	-0.2692	-1.0636	0.08082	-0.6357	-0.5036	0.0143	-0.1504	-0.5594	-0.4771	-0.4346	0.84272
TCGA-43	-0.2162	-0.8211	-1.0809	1.38885	-0.2832	-0.4919	-0.6321	-0.4735	-0.523	0.19969	-0.0315	-0.2206	2.22404	-0.5882	-0.0587	0.57463	-0.9589	-0.3974	-0.4691	-0.5924	-0.5038	-0.8004
TCGA-18	-0.3625	0.79386	-0.5181	-0.1479	-0.2832	0.37465	-1.1257	-0.3114	-0.5526	-0.5416	0.42381	-0.2692	-0.8228	0.12404	0.60032	-0.5036	1.23465	-0.1366	-0.3258	-0.1564	-0.669	1.11945
TCGA-NC	-0.3625	-0.905	-0.736	-0.5592	-0.2832	-0.4743	2.74769	-0.4735	-0.5526	-0.1154	2.53653	-0.2692	1.22809	-1.2447	-0.8136	-0.5036	2.13053	-0.3974	-0.6123	2.80274	-0.669	-0.9827
TCGA-39	-0.3625	1.87838	-0.3641	-1.5382	-0.2832	0.09152	-0.6366	-0.4735	1.24331	-0.5416	-0.7754	-0.2692	0.19401	0.57243	-0.9545	-0.5036	1.07981	-0.3974	-0.6123	0.7456	-0.669	0.98059
TCGA-43	-0.3625	-0.371	-0.1306	-0.6879	-0.2832	0.97464	0.43179	-0.2594	2.25407	-0.5416	-0.5495	-0.2692	1.36888	-0.2959	0.13929	-0.5036	0.57023	-0.3974	-0.6123	0.05873	-0.669	-1.0582
TCGA-22	-0.3625	0.64974	-0.7234	-0.4725	-0.2832	0.43151	-1.1257	-0.4735	0.64851	-0.5416	-0.8413	-0.2692	1.8273	-1.0786	-0.3214	0.3633	-0.9589	-0.3974	-0.4871	-0.5924	-0.669	0.4628
TCGA-34	-0.3625	0.79478	0.95855	0.85517	-0.2832	0.06938	0.11969	-0.4735	0.35924	-0.3496	-0.735	-0.2692	-0.8874	-0.2364	-1.1032	-0.5036	3.11305	0.34726	0.16525	0.6913	-0.3209	-0.3431
TCGA-60	-0.3625	0.30064	-0.576	-0.7682	-0.2832	0.43579	-0.4634	-0.215	1.41822	-0.5416	-0.6584	-0.2692	1.87646	-0.643	-0.154	-0.5036	0.10533	-0.3974	-0.6123	-0.3339	-0.669	-0.1809
TCGA-85	-0.3625	0.5686	-0.9629	0.09325	-0.2832	-0.5732	0.49844	-0.4735	-0.1171	-0.3254	-0.5026	-0.0184	2.04441	0.45365	-0.4801	0.17045	-0.9589	-0.3974	-0.6123	0.5185	-0.669	-1.0477
TCGA-NC	-0.3625	1.03612	-0.032	-0.533	-0.2832	0.74862	1.65709	-0.1128	0.34991	-0.5416	0.25765	-0.2692	0.44846	0.15247	-0.6437	-0.5036	-0.734	-0.3974	-0.2702	-0.5924	-0.2747	-0.5755
TCGA-18	-0.3625	0.06426	-0.7163	0.94231	-0.2832	-0.5507	-0.5889	-0.4735	-0.3651	-0.5416	2.91676	-0.2692	-0.4247	0.50286	1.92291	-0.5036	0.37532	-0.3974	-0.2186	-0.5924	-0.3969	-0.9987
TCGA-56	-0.3625	-0.1068	0.05539	2.78199	-0.2832	0.29184	-1.1257	-0.4735	2.88048	-0.5416	-0.5199	-0.2692	-0.7803	1.34802	-1.6666	-0.5036	0.78039	3.79716	-0.3039	2.06278	-0.669	-1.2365
TCGA-85	-0.1317	-0.905	0.14456	0.38216	-0.2832	0.68638	1.25299	-0.4735	2.24332	-0.5416	2.78734	-0.2692	-0.6142	1.96525	-1.4429	-0.5036	-0.3549	-0.3974	-0.6123	0.07825	0.79779	-0.8126
TCGA-18	-0.3625	0.30607	0.12791	1.61329	-0.2832	1.08249	-0.5326	-0.4735	-0.2274	-0.5416	-0.6996	-0.2692	0.03591	0.99533	-1.2397	-0.5036	-0.2713	-0.2413	-0.6123	-0.236	-0.669	-0.2882
TCGA-22	-0.3625	0.70262	-0.7501	0.71143	-0.2832	-0.2167	-0.5641	-0.4735	0.6411	-0.5028	1.67169	-0.2692	0.22386	-0.7295	0.07249	-0.5036	0.61647	-0.3974	-0.4465	-0.1248	-0.669	-0.0166
TCGA-66	-0.3625	1.13771	-1.0809	-0.831	-0.2832	-0.213	-0.4564	-0.3755	0.32227	-0.3758	2.5056	-0.2692	-1.0379	-1.0971	-0.1792	-0.3664	1.15258	-0.3974	6.37297	-0.5924	-0.669	0.24849
TCGA-34	-0.3625	0.13706	0.05223	0.82715	-0.2832	1.291	-0.7342	-0.4735	0.82755	-0.5416	0.28951	-0.2692	0.1004	0.14418	-1.059	-0.5036	2.25171	-0.3974	-0.6123	0.30707	-0.669	-1.2014
TCGA-34	-0.3625	0.3066	-0.618	-0.7102	-0.2832	0.0038	-0.2225	-0.4735	-0.2827	-0.2582	-0.8413	-0.2692	-0.3872	0.46119	0.13142	-0.5036	1.25023	-0.3974	0.7767	0.55837	-0.669	1.4809
TCGA-66	-0.3625	0.23123	-0.1289	1.12801	-0.2832	-0.7163	-1.0911	-0.4735	0.70235	-0.124	2.86445	-0.2692	-1.169	-0.2362	-0.0094	-0.5036	2.18094	0.61268	-0.2029	-0.4975	######	0.27494
TCGA-85	0.19579	-0.905	0.20829	-1.5382	-0.2832	1.36167	0.91266	-0.0345	1.17842	-0.5416	-0.2173	-0.2692	0.13898	1.30638	-0.8754	-0.5036	0.03193	-0.3974	-0.6123	1.4019	0.55041	-0.0334
TCGA-92	-0.3625	-0.2278	-0.3747	0.45465	-0.2832	0.35425	0.45821	-0.4735	3.54426	-0.5416	0.12089	-0.2692	-0.2124	-0.5415	0.75666	-0.5036	-0.2534	3.16871	-0.5035	-0.576	-0.4827	0.11787
TCGA-46	-0.3625	0.58096	-0.3178	0.44664	-0.2832	0.17754	-1.1257	0.10184	-0.5526	-0.4597	0.9942	-0.2692	-0.8618	-0.6883	-0.1154	-0.5036	1.95833	-0.3974	-0.2624	-0.3249	-0.669	1.28773
TCGA-77	-0.3625	-0.0482	-1.0202	-0.1433	-0.2832	-0.0352	1.99635	-0.4393	1.16593	-0.5416	-0.4887	-0.0401	1.87897	-0.2698	-0.8334	-0.5036	0.03428	0.6282	-0.4857	-0.232	-0.669	-0.4436
TCGA-94	-0.3625	1.90398	-1.0809	-0.5894	-0.2832	-0.7535	-0.4424	-0.4735	2.21796	-0.5416	-0.8413	-0.2692	1.66914	-1.0926	0.17055	0.26396	-0.9589	-0.3974	-0.3357	-0.5924	-0.5193	0.10185
TCGA-18	-0.3625	-0.2761	-0.6375	0.44803	-0.2832	0.77945	-1.1257	-0.4735	0.58457	1.58057	-0.8413	-0.2692	0.50056	-0.5084	-0.9539	0.0536	0.06901	-0.3974	-0.2341	-0.5924	0.66721	0.42466
TCGA-77	-0.3625	-0.7287	0.00042	0.16753	-0.2832	0.05633	0.52955	-0.4735	2.10682	-0.5416	-0.8413	-0.2692	0.68341	-0.8758	0.51997	-0.5036	-0.3563	-0.3974	-0.3796	-0.5924	1.62225	0.48111
TCGA-21	-0.3625	-0.1215	-1.0809	0.77908	-0.2832	-0.0547	-1.1257	-0.4735	2.32952	0.68173	0.97598	-0.2692	-1.1292	-0.8109	0.44667	-0.5036	2.10846	1.47502	-0.6123	1.28468	2.94872	-0.297
TCGA-18	-0.3625	-0.1685	1.08993	0.0093	-0.2832	-0.4018	0.10504	-0.4623	-0.5249	-0.5416	1.93392	-0.2692	-0.5813	-0.202	1.43666	-0.5036	0.79727	4.53514	-0.6123	-0.5654	0.08587	-0.8741
TCGA-66	-0.3625	1.3172	-0.3441	-0.5448	-0.2832	-0.4324	-0.4122	-0.4735	-0.4269	-0.4148	0.5695	-0.2692	-0.4711	-0.0267	-0.4157	-0.5036	0.24473	-0.3974	3.39498	-0.5924	-0.3601	0.82862
TCGA-56	-0.3625	0.54571	-0.7704	0.34555	-0.2832	-0.6961	-1.1257	-0.4735	0.84928	1.50083	-0.8413	-0.2692	-0.4359	-1.3304	-0.6014	6.19044	-0.9589	-0.3974	0.27922	-0.5924	0.89826	-0.4025
TCGA-77	-0.3625	0.71799	1.04529	0.67593	-0.2832	1.00716	0.26616	-0.4735	-0.5526	-0.5416	-0.0983	-0.2692	-0.2701	-0.4683	0.26349	-0.5036	-0.3005	-0.3974	-0.6123	-0.5924	0.14824	-0.7524
TCGA-22	-0.3625	2.00276	-1.0809	-1.0637	-0.2832	-0.6608	0.2982	-0.3343	0.41316	-0.2434	-0.6699	0.99569	0.82094	-1.4233	0.87224	0.44442	-0.9589	-0.3974	-0.5071	-0.5924	-0.3131	0.80729
TCGA-NK	-0.251	-0.905	1.65594	-1.5382	-0.2832	1.30453	3.40919	-0.4735	-0.5526	3.76021	-0.338	-0.2692	-0.1074	-0.064	-1.483	-0.5036	-0.3071	3.50093	-0.274	0.57756	-0.669	-1.1224
TCGA-22	-0.3625	0.39295	-0.5361	0.12669	-0.2832	0.16917	-0.5377	-0.4735	3.18898	-0.5416	-0.8072	-0.2692	0.28966	0.17761	-0.5137	-0.5036	0.13474	-0.3974	-0.4589	0.00314	-0.669	0.93245
TCGA-O2	-0.3625	0.6867	-1.0809	-0.6873	-0.2832	0.23945	1.19219	-0.4735	0.73368	0.1378	1.65359	-0.2692	0.09956	-0.4366	0.56907	-0.5036	0.32993	4.38519	-0.5795	-0.5924	-0.0186	-0.8417
TCGA-52	-0.3625	-0.5123	2.23678	-1.5382	-0.2832	2.22232	0.428	1.53207	-0.4195	-0.2689	-0.6272	-0.2692	0.27193	1.1492	-1.057	-0.5036	0.14053	-0.3974	-0.6123	-0.5924	-0.669	-1.245
TCGA-46	-0.3625	0.93337	-0.1351	-1.0231	-0.2832	0.275	-0.4178	-0.4735	-0.5526	0.19737	-0.8413	-0.2692	0.97696	0.92883	-0.6246	-0.5036	0.22863	-0.3974	-0.6123	-0.4225	0.0238	-0.0683
TCGA-58	-0.3625	2.09565	-0.0179	-0.4815	-0.2832	0.20212	-0.4514	-0.4735	0.89874	-0.5416	-0.8413	-0.2692	0.76505	-0.7387	-0.5194	-0.5036	-0.4179	-0.3974	0.0497	0.02786	-0.669	-0.0204
TCGA-85	-0.3625	-0.175	-1.0809	1.75878	-0.2832	-0.4073	-1.1257	-0.4735	-0.5526	0.73317	-0.8413	-0.2692	1.57063	-1.6777	-0.774	1.94315	-0.9589	-0.3974	-0.4662	-0.5924	0.77332	-0.2533
TCGA-18	-0.3625	1.29209	-0.5647	0.67653	-0.2832	-1.1028	-1.1257	-0.2916	-0.5526	-0.5416	-0.8413	-0.2692	1.11278	-1.1164	-0.0618	0.88652	-0.9589	-0.3974	-0.334	-0.5924	0.91876	0.44918
TCGA-60	-0.3625	-0.905	-1.0568	1.05357	-0.2832	-0.8472	-1.1257	-0.4735	-0.5526	1.56739	-0.8413	-0.2692	2.03346	-1.7519	1.47903	-0.0252	-0.9589	-0.3974	2.2761	-0.5924	0.55375	-1.225
TCGA-94	-0.3625	0.71247	-1.0051	-0.4429	-0.2832	-0.7107	-0.8696	-0.4735	-0.5526	-0.0586	1.44072	0.90825	2.27725	-0.925	-0.7019	0.79647	-0.9589	-0.3974	-0.6123	-0.0251	-0.669	-0.5718
TCGA-43	1.47923	-0.8672	0.28742	1.24676	-0.2832	8.06E-05	0.25782	-0.4735	-0.1131	0.14611	-0.6374	-0.2692	0.73314	-0.4463	-1.4048	-0.5036	0.2724	-0.3974	-0.6123	-0.5924	1.07732	-0.0828
TCGA-34	-0.3625	-0.0083	0.08199	1.59431	-0.2832	0.38597	-0.165	-0.4735	-0.5526	0.46615	0.87861	-0.2692	-1.0942	0.08372	0.83397	-0.5036	0.2911	2.89479	0.42108	-0.5924	0.0258	-1.1169
TCGA-77	-0.3625	-0.567	-0.6308	0.02785	-0.2832	0.03394	-0.5219	-0.4735	0.80227	-0.5416	-0.3456	-0.2692	1.70424	-0.8454	1.4859	0.60103	-0.9589	-0.3974	-0.4199	-0.5924	-0.0276	-0.838
TCGA-56	-0.3625	0.99104	-1.0809	-0.6968	-0.2832	-0.3	-0.4081	-0.4735	0.08419	-0.5179	-0.0221	-0.2692	0.63745	-1.1908	2.92121	-0.5036	1.12179	-0.3974	-0.1956	0.16265	-0.669	-0.8241
TCGA-85	-0.0726	-0.905	-0.7586	1.2521	-0.2832	-0.3323	0.05018	-0.4735	1.09291	0.25925	-0.8413	-0.2692	1.69749	-1.1629	-1.0437	1.58059	-0.9589	1.56565	1.42756	-0.5924	-0.6605	-0.7748
TCGA-66	-0.3625	-0.2381	-1.0809	1.98658	-0.2832	0.41014	-1.1257	-0.4735	-0.5526	-0.5416	-0.2502	-0.2692	0.06156	0.10662	0.94264	-0.5036	0.88911	-0.3974	-0.0908	1.47254	-0.669	-1.0846
TCGA-98	-0.3625	1.03678	1.24915	0.10958	-0.2832	1.0776	1.89644	-0.4735	0.12831	-0.5416	-0.0224	-0.2692	-1.2002	-0.5493	-0.9949	-0.5036	-0.1161	0.11447	-0.6123	-0.5924	-0.2864	0.88015
TCGA-22	-0.3625	0.22586	0.09515	-0.4565	-0.2832	0.26813	-0.6707	-0.4735	-0.4809	-0.5416	0.65201	-0.2692	-0.7451	-0.517	-0.6005	-0.5036	-0.1811	-0.3974	-0.1382	-0.5924	0.14916	3.09634
TCGA-58	-0.3625	-0.2814	-1.0688	0.4003	-0.2832	-0.0698	-0.4784	-0.4735	-0.5526	-0.5416	-0.4496	-0.072	-0.0686	-0.0536	3.08423	-0.5036	1.23747	0.16165	1.65197	0.20844	-0.669	-1.2242
TCGA-22	-0.3625	-0.2355	-0.2983	0.79553	-0.2832	2.17346	-0.0763	-0.4735	-0.3716	-0.5416	-0.8413	-0.2692	-0.532	-0.0048	-1.0878	-0.5036	-0.7692	-0.3974	-0.6123	-0.3932	-0.669	1.76435
TCGA-66	-0.3625	0.3024	0.18727	1.42332	-0.2832	-0.2844	-0.056	-0.4735	-0.5526	-0.5416	0.07356	0.62428	-0.5285	0.05995	2.08466	-0.5036	0.05179	0.74292	-0.6123	0.15307	-0.669	-1.0349
TCGA-21	-0.3625	0.23683	-0.0846	0.20605	-0.2832	0.54144	0.14218	-0.4735	1.86415	0.41	1.10486	-0.2692	-0.2849	-0.942	-0.8372	-0.5036	-0.0427	0.81215	-0.6123	-0.5924	0.29082	0.7185
TCGA-58	-0.3625	2.82807	-1.0809	-0.3954	-0.2832	-0.1358	-1.1257	-0.4735	-0.4241	-0.5416	0.62001	-0.2692	0.23028	-1.3282	-0.6758	-0.5036	-0.2567	0.11529	-0.4151	-0.3502	-0.669	1.54363
TCGA-60	-0.3625	0.00509	-0.422	0.9148	-0.2832	0.30456	-1.1257	-0.4735	-0.5526	-0.5416	-0.6094	-0.2692	1.55882	-0.9213	0.68056	-0.5036	-0.1536	-0.3974	-0.3589	-0.5924	-0.0906	-0.7194
TCGA-22	-0.3625	0.94035	0.02341	-0.8305	-0.2832	0.4733	-0.3094	-0.4735	-0.5526	-0.5416	0.22155	-0.2692	-0.313	1.05842	-0.9061	-0.5036	0.32448	-0.2269	-0.6123	0.45309	-0.669	1.09079
TCGA-37	-0.3625	-0.7033	0.38669	-0.0595	-0.2832	0.86337	0.74849	-0.4735	-0.3286	0.10008	0.58627	1.62705	-1.5147	0.90653	1.48445	-0.5036	-0.2037	2.92753	-0.5691	-0.5924	1.03756	-0.1981
TCGA-63	-0.3625	-0.1425	-1.0809	0.20283	-0.2832	-0.5051	1.74805	-0.4735	-0.2648	2.0372	0.1216	-0.2692	-1.2411	0.32039	-0.5495	-0.5036	3.5543	-0.3974	-0.2836	0.15632	-0.669	1.0401
TCGA-66	-0.3625	0.26768	-1.0145	0.17828	-0.2832	-0.5104	0.62715	0.03971	-0.4778	-0.5416	0.07431	-0.2692	1.36542	-0.5389	-0.0563	0.9894	-0.9589	-0.3974	-0.2724	-0.5924	-0.669	-0.0801
TCGA-LA	-0.3625	3.18841	-1.0809	-0.8648	-0.2832	0.19557	0.65703	-0.4735	-0.5526	0.54492	-0.5241	-0.2692	0.8838	-1.606	-0.3061	1.91897	-0.9589	0.70362	-0.6123	-0.5924	-0.669	-1.2705

TCGA-66	-0.3625	-0.3242	0.8083	-0.5251	-0.2832	0.27378	-0.6015	-0.4735	0.76761	-0.5416	-0.3983	-0.1595	0.69925	0.32525	1.34164	-0.5036	-0.1223	-0.3974	-0.2065	-0.5924	0.63337	-0.9584
TCGA-22	-0.3625	0.20301	-0.0664	0.76255	-0.2832	-0.0397	-1.1257	-0.4735	0.20385	-0.5416	-0.6626	-0.2692	0.78145	-0.3711	-0.2528	-0.5036	0.79399	-0.3974	-0.4197	-0.5924	-0.1361	0.31405
TCGA-66	-0.3625	-0.4326	-0.142	1.39864	-0.2832	0.47559	0.2762	-0.4735	0.60814	-0.5112	1.27974	1.04888	-0.6751	0.82596	0.24413	-0.5036	-0.3551	4.81235	0.27546	-0.5924	-0.3276	-1.2467
TCGA-66	-0.3625	-0.6674	-1.0809	2.42524	-0.2832	-0.3522	-1.1257	-0.4735	-0.5526	0.42071	-0.1108	-0.2692	1.04999	0.59414	-1.2398	2.15166	-0.9589	-0.3974	-0.0375	-0.5924	0.37043	-1.2304
TCGA-77	-0.3625	0.44851	1.30191	0.41747	-0.2832	1.28534	-0.1385	-0.4735	2.0315	-0.5416	-0.487	-0.2692	-0.1722	-0.2792	-0.2887	-0.5036	0.05226	-0.2565	-0.6062	-0.5924	0.08206	-0.873
TCGA-37	-0.3625	-0.4276	0.23017	-1.5382	-0.2832	2.21604	-0.4953	-0.4735	-0.3227	7.8541	-0.8413	-0.2692	-1.1265	-1.7519	-1.7519	-0.5036	1.48528	0.39347	-0.6123	1.18338	5.84003	-1.2116
TCGA-77	-0.3625	-0.905	0.26065	-0.2281	-0.2832	0.85112	-1.1257	-0.4735	-0.5526	-0.5416	-0.677	-0.2692	1.58429	1.23477	0.24016	-0.5036	-0.2794	-0.3974	-0.6123	-0.4295	0.8266	-1.2367
TCGA-33	-0.3625	2.80521	-0.2864	2.07013	-0.2832	-0.2812	-0.7446	-0.4735	-0.5526	0.64625	1.76936	-0.2692	-0.6593	-1.3085	-1.5135	-0.0153	-0.9589	-0.3974	-0.6123	1.16196	-0.669	-0.5644
TCGA-58	-0.3625	0.41861	-1.0478	0.03346	-0.2832	1.72198	-1.1257	-0.4735	-0.5526	0.64983	1.17119	-0.2692	0.00617	-1.2862	-1.0708	3.50923	-0.9589	0.94633	-0.4167	-0.5924	-0.669	-0.679
TCGA-33	-0.1843	-0.905	0.29865	-0.2985	-0.2832	0.50179	0.2164	-0.4735	-0.5526	0.46251	0.21265	4.97634	-0.0146	0.64304	1.59239	-0.5036	-0.3474	-0.3974	-0.6123	-0.5924	1.30151	-1.2705
TCGA-63	-0.3625	0.67102	-0.7779	0.40288	-0.2832	-0.6648	-0.1433	-0.3168	0.04623	-0.5416	-0.81	-0.2692	1.34288	-0.532	-0.0472	-0.5036	0.26237	-0.3974	-0.5446	-0.5924	-0.669	0.54355
TCGA-43	-0.3625	0.21616	-1.0809	0.91862	-0.2832	-0.7636	-1.1257	-0.4735	-0.5526	-0.5416	-0.8413	-0.2692	2.88973	-1.2274	-0.5145	0.99602	-0.572	-0.3974	-0.6123	-0.5924	1.02302	-1.0637
TCGA-43	-0.3625	-0.6635	-0.2407	0.28757	-0.2832	-0.5988	-1.1257	-0.4735	-0.5526	-0.5416	0.06442	-0.2692	2.46445	-1.3615	1.05442	-0.5036	1.6708	-0.3974	-0.5463	-0.5924	-0.2149	-1.2558
TCGA-22	-0.3625	2.06031	-1.0809	-0.903	-0.2832	-1.1028	-0.4955	-0.4735	0.7705	0.31057	-0.7417	0.99106	0.35244	-0.9346	0.72126	-0.5036	-0.1804	-0.3974	1.16125	-0.5924	0.95714	0.75373
TCGA-58	-0.3625	0.29263	0.92953	0.06942	-0.2832	1.39992	1.71716	-0.4735	-0.5526	-0.4171	-0.4792	-0.2692	-0.9634	1.27819	-0.2503	-0.5036	-0.2079	-0.29	-0.6123	-0.5924	1.33479	-0.7253
TCGA-77	-0.3625	0.16632	1.5814	-0.1661	-0.2832	1.2447	0.05408	-0.4735	1.38561	-0.5416	1.13253	-0.2692	-0.0377	0.96597	-1.5032	-0.5036	-0.4564	-0.3974	-0.4901	-0.5924	-0.2612	-0.818
TCGA-18	-0.3625	-0.4441	0.8519	0.17357	-0.2832	1.12713	-0.0712	-0.4735	-0.1523	-0.5416	-0.0454	-0.2692	-0.1793	0.3085	0.75368	-0.5036	0.19729	-0.3974	-0.6123	-0.5924	1.7885	-1.0418
TCGA-85	-0.3625	0.06703	1.33609	-0.2734	-0.2832	2.48469	0.84792	-0.4735	-0.5526	-0.5416	-0.4394	-0.2692	-0.8376	0.50366	-0.3961	-0.5036	-0.1615	0.757	-0.6123	0.98521	0.49464	-1.196
TCGA-22	-0.3625	0.79591	-0.6939	0.62328	-0.2832	0.07975	0.38075	-0.4735	0.3061	-0.5416	-0.5488	-0.2692	0.25509	0.21861	1.20634	-0.1205	-0.9589	-0.3974	-0.4903	-0.5924	-0.3015	-0.5103
TCGA-56	-0.3625	3.9437	-1.0809	0.73351	-0.2832	-0.2697	-1.1257	-0.4735	-0.5526	0.37435	-0.8413	-0.2692	-0.7695	-0.9568	0.22546	-0.5036	-0.6	1.20492	-0.2061	-0.2399	-0.669	0.4815
TCGA-39	-0.3625	2.66764	-1.0809	1.04175	-0.2832	-0.7193	-1.1257	0.78679	0.03114	-0.1984	-0.7836	-0.2692	-1.1677	-0.345	0.30547	-0.0544	0.26433	-0.3974	-0.515	-0.5924	-0.669	1.31629
TCGA-J1-	-0.3625	0.23292	-0.6312	-1.043	-0.2832	-0.6097	1.15156	-0.2607	0.48509	-0.5416	-0.8413	-0.2692	0.93975	-0.6158	1.27184	-0.5036	0.46504	-0.3974	-0.6123	-0.4589	-0.5671	0.91537
TCGA-92	-0.3625	0.10224	-0.5797	-0.2564	-0.2832	0.31381	2.36466	-0.4735	0.25037	0.61544	1.12632	-0.2692	-0.2343	0.31996	-0.2784	-0.5036	-0.0684	1.23718	0.59113	1.23168	-0.669	-1.0471
TCGA-46	-0.3625	-0.6931	-0.1234	1.84948	-0.2832	1.21823	-1.1257	-0.4735	-0.1796	-0.5179	0.09045	-0.2692	-1.2021	-0.0559	3.24568	-0.5036	0.05257	-0.3956	-0.4201	-0.5924	-0.669	-0.8361
TCGA-43	-0.3625	-0.5584	-0.1201	-0.0822	-0.2832	-0.5716	-1.1257	-0.4735	-0.5526	-0.5416	-0.4717	-0.2692	2.25591	-1.0433	1.60943	-0.5036	0.76433	-0.3974	0.30878	-0.5924	-0.196	-1.135
TCGA-NC	-0.3625	-0.1113	0.82816	-1.0373	-0.2832	1.40709	1.10038	-0.4735	2.45523	-0.5363	0.65428	-0.2692	-0.1917	1.05118	-1.2441	1.14989	-0.9589	-0.3974	-0.0544	-0.5924	1.16066	-1.1679
TCGA-22	-0.3625	-0.2274	-0.4617	-0.3754	-0.2832	-0.8505	-0.9616	-0.4735	-0.5313	-0.5416	-0.6345	-0.2692	2.67266	-1.3252	2.31751	-0.3304	-0.4981	-0.3974	-0.4888	-0.3068	-0.669	-0.8301
TCGA-60	-0.1431	-0.9021	1.60318	-0.0363	-0.2832	1.10543	-0.7627	-0.4735	-0.5526	0.43051	-0.0043	-0.2692	-0.6893	0.04555	0.59959	-0.5036	0.51694	0.0457	-0.2308	-0.5924	2.23751	-0.6266
TCGA-33	-0.3625	2.9918	-0.5907	-0.3804	-0.2832	0.0546	-1.1257	-0.4735	3.52478	-0.2688	-0.8413	-0.2692	0.09013	-1.4284	-0.7385	-0.4579	-0.577	0.43292	-0.5595	-0.5924	-0.4776	1.22908
TCGA-63	-0.3625	0.90632	-0.8571	0.51242	-0.2832	-0.4609	-0.8571	0.36396	-0.5194	-0.0176	-0.2503	0.80322	0.72155	-1.5218	1.20328	-0.5036	-0.2016	-0.3974	-0.6123	-0.5924	-0.669	0.42405
TCGA-85	-0.3625	0.67121	-0.8714	2.32541	-0.2832	1.10045	-1.1257	-0.4735	-0.5526	-0.5416	-0.8413	-0.2692	0.44564	-1.0132	0.34375	-0.2782	0.16497	-0.3974	-0.6123	-0.0235	-0.669	-1.0669
TCGA-NC	-0.3625	1.27173	-1.0809	-0.4742	-0.2832	0.67524	1.60292	-0.4735	-0.5526	6.66892	-0.8413	-0.2692	-0.9829	-1.2583	-1.3905	-0.5036	-0.1622	-0.3974	-0.4995	2.17479	-0.669	0.15796
TCGA-43	-0.3625	0.09775	1.29478	-0.9277	-0.2832	1.3683	-0.7972	0.87959	-0.5526	-0.5416	0.15948	0.05995	-1.2258	-0.258	1.35992	-0.5036	0.00225	-0.3974	0.18709	-0.5924	-0.669	0.56867
TCGA-21	-0.3625	-0.1766	0.05445	1.29416	-0.2832	-0.2511	-1.045	-0.4735	-0.5526	1.22275	1.22846	2.28403	-1.0463	0.61318	0.49944	-0.5036	-0.5199	1.25569	1.01703	-0.5924	0.76635	-0.6036
TCGA-98	-0.3625	0.58646	-0.7968	-0.6492	-0.2832	0.23557	0.90413	-0.4735	-0.1687	-0.5416	-0.8413	-0.2692	1.70515	-0.0951	-0.0697	-0.5036	0.83956	-0.3974	-0.6123	-0.1948	0.29181	-1.0598
TCGA-85	-0.3625	1.52044	-1.0438	-0.5828	-0.2832	0.97212	-0.1775	-0.4735	0.13756	-0.4875	0.44299	-0.2692	-0.1954	-0.9178	-0.4421	-0.5036	1.83038	0.84179	-0.6123	1.02936	-0.669	0.09992
TCGA-68	-0.3625	-0.2567	1.60793	-1.3213	-0.2832	1.19596	1.107	-0.4735	-0.5526	1.61187	0.23242	0.79074	-0.8974	0.77491	-0.2964	-0.5036	0.1614	1.21776	-0.6123	2.0306	0.03078	-1.1303
TCGA-66	-0.3625	2.02391	-0.1705	-0.5956	-0.2832	0.24205	-1.0873	-0.4735	0.42929	-0.5416	0.29707	-0.2692	-1.3091	0.12365	2.04111	-0.5036	-0.2173	-0.3974	-0.2399	-0.5924	0.29345	0.35452
TCGA-NC	-0.3625	0.19901	-0.3888	-0.3083	-0.2832	0.27649	2.88386	-0.4735	-0.5526	-0.5416	0.17376	-0.2692	-0.2099	1.37089	0.56593	-0.5036	-0.0668	-0.3974	-0.5478	0.87818	-0.3597	-0.9581
TCGA-NC	-0.3625	-0.4593	2.24371	-1.3061	-0.2832	2.67899	-0.2026	-0.4735	0.59223	-0.5416	0.40272	-0.2692	-0.2949	0.63178	-0.7705	-0.5036	0.14354	-0.3974	-0.3399	-0.5924	-0.669	-0.6731
TCGA-98	-0.0657	1.02038	-0.22	0.65266	-0.2832	-0.0897	-1.1257	-0.4735	0.4906	1.65561	-0.8413	-0.2692	0.07845	-1.4494	-0.0087	-0.5036	0.95334	-0.1979	1.00899	-0.5924	0.70811	-0.5586
TCGA-66	0.06531	-0.7495	-0.6112	1.24883	-0.2832	0.49429	-1.1257	-0.4735	0.27936	-0.5416	1.11339	-0.2692	-0.4557	-0.3914	-0.1547	-0.5036	0.5396	2.54038	-0.1899	-0.5924	-0.0087	0.85508
TCGA-77	-0.3625	-0.7494	0.16468	0.98654	-0.2832	-0.5935	-1.1257	-0.4735	-0.5526	0.02235	0.10828	-0.2692	2.28446	-0.7823	-0.6314	-0.0656	-0.9589	-0.3974	-0.2403	-0.5924	0.29895	-0.4746
TCGA-56	-0.3625	2.38575	-0.1927	-0.6467	-0.2832	-0.9139	-0.4045	-0.4735	-0.0427	-0.1478	-0.6762	-0.2692	2.66824	-1.3928	-1.3502	0.0819	-0.9589	-0.3974	-0.2523	-0.5924	0.36224	-0.836
TCGA-79	-0.3625	0.97577	-1.0809	0.95685	0.01329	-0.5159	-1.1257	-0.4735	-0.5526	0.91131	-0.8413	-0.2692	2.4955	-1.7519	-0.7586	-0.5036	-0.6312	-0.3974	-0.6123	0.10052	-0.1976	-0.5615
TCGA-63	-0.3625	1.23655	-0.9095	-1.5252	-0.2832	1.19407	-0.1513	-0.4735	-0.5526	-0.4707	-0.8413	1.01402	0.78657	0.84285	0.58931	1.11246	-0.9589	-0.3974	-0.6123	-0.5924	-0.669	-0.908
TCGA-56	-0.3625	0.3031	0.43476	-0.0995	-0.2832	0.80881	0.40221	-0.4735	0.41637	-0.5416	-0.5541	-0.2692	0.63382	0.07586	-0.8575	-0.5036	0.02582	-0.3974	-0.503	-0.4225	0.25259	-0.2325
TCGA-51	-0.3625	0.90095	-0.6206	-0.2229	-0.2832	-0.7086	-1.1257	-0.4735	-0.5526	0.1513	-0.8413	0.27106	3.04855	-1.6104	-0.2282	-0.5036	-0.0824	-0.3974	-0.6123	-0.5924	0.37235	-0.7859
TCGA-34	-0.3625	-0.4731	0.82092	-0.5717	-0.2832	-0.1134	2.31797	-0.4735	-0.5526	-0.5416	-0.4712	-0.2692	0.84559	-0.0316	-0.4486	-0.5036	0.63105	-0.3974	-0.6123	-0.0816	0.60616	-0.1275
TCGA-85	-0.3625	2.19059	-1.0809	1.18704	-0.2832	-0.5243	-1.1257	-0.4735	0.16937	-0.5416	-0.8413	-0.2692	1.67456	-0.5091	-1.2177	-0.2197	-0.9589	-0.3974	-0.6123	-0.5924	-0.669	-0.1316
TCGA-77	-0.3625	0.50642	-0.9223	-0.4811	-0.2832	-1.1028	0.44389	1.38603	0.54709	1.65807	-0.8413	-0.2692	2.15243	-1.5456	-0.4595	0.03743	1.13315	-0.3974	-0.6123	-0.5924	0.81352	-1.2705
TCGA-77	-0.3625	1.80837	-0.791	0.75989	-0.2832	0.45078	0.88046	-0.4735	-0.2043	-0.5416	1.13943	-0.2692	-1.1918	0.60596	-0.6183	-0.5036	1.2152	1.19452	-0.1708	0.1656	-0.669	-0.6689
TCGA-85	2.16136	0.59744	0.22146	-0.4166	-0.2832	0.0297	0.27586	-0.4735	3.10096	0.10443	-0.1153	-0.2692	0.82072	-0.7623	-1.4912	-0.5036	0.37916	0.18009	-0.6123	-0.3828	-0.669	-0.5949
TCGA-56	-0.3625	-0.1596	-1.0267	1.29589	-0.2832	-0.6119	-0.2736	-0.4735	-0.5526	-0.5416	2.0695	-0.2692	-0.2823	0.06436	1.43578	-0.5036	-0.0292	7.7429	-0.0592	0.20267	-0.669	-1.1874
TCGA-21	-0.3625	0.13908	0.22957	1.65064	-0.2832	-0.0962	0.14222	-0.4735	-0.5526	-0.1428	-0.8413	-0.2692	-0.1574	0.46249	0.35461	-0.5036	0.20036	-0.3974	-0.6123	0.40106	-0.2753	-0.5757
TCGA-46	0.28955	-0.3399	0.10619	2.07145	-0.2832	0.51614	-1.1257	-0.4735	-0.523	-0.5416	0.64754	-0.2692	-0.1538	-0.6498	-0.2028	-0.5036	1.25694	-0.3569	0.16558	-0.5924	0.77912	-0.9637
TCGA-56	-0.3625	0.1228	-0.3948	-0.1201	-0.2832	0.18053	0.65435	-0.4735	0.7315	-0.243	1.06143	-0.2692	0.0309	0.04594	1.60068	-0.4823	-0.3603	0.28734	-0.4942	-0.5924	-0.2112	-0.7479
TCGA-68	-0.3625	1.45576	-1.0809	-0.1628	-0.2832	-0.6756	-1.1257	-0.4735	1.91493	0.08975	-0.8413	-0.2692	1.05382	-1.7519	0.22434	0.89222	-0.9589	-0.3974	0.21179	-0.5924	-0.2028	0.71819
TCGA-56	-0.3625	0.38692	0.29966	-0.274	-0.2832	1.16492	0.28666	-0.4735	0.71064	0.94564	-0.0545	-0.2692	-0.8856	0.68369	-0.0925	-0.5036	1.01183	0.73877	-0.5617	-0.5924	1.55534	-1.0206
TCGA-18	-0.3625	-0.5605	-1.0809	1.08593	-0.2832	0.17042	1.29341	-0.4735	-0.5526	-0.5416	0.08175	0.56243	-0.5044	1.03616	0.91274	-0.5036	1.4609	0.21234	0.05631	1.70625	-0.669	-1.1945
TCGA-37	-0.3625	-0.3931	0.35288	-0.7844	-0.2832	1.93146	2.18097	0.64357	-0.5526	-0.5416	-0.0129	0.16965	-0.7397	-0.2631	0.41555	-0.5036	-0.5391	-0.3974	-0.581	-0.4287	-0.669	0.57414
TCGA-22	-0.3625	0.42546	-0.02	-1.1704	-0.2832	0.0393	-0.5094	-0.4735	-0.0161	-0.5416	-0.7794	-0.2692	1.49019	-0.0175	0.99123	-0.5036	0.16345	-0.3974	-0.4875	-0.5596	-0.669	-0.2614
TCGA-85	-0.3625	2.21398	-1.0809	1.18404	-0.2832	-0.9743	0.7503	-0.4735	-0.4073	-0.5416	-0.8413	-0.2692	0.32407	0.40916	-1.8308	0.8233	-0.9589	-0.3974	-0.1232	1.15263	-0.669	0.14028
TCGA-60	-0.3625	0.471	-0.703	1.03693	-0.2832	0.63268	0.11891	-0.4735	-0.5526	-0.5416	-0.2136	-0.2692	0.13614	-0.3874	-0.2089	-0.5036	0.94601	0.39917	0.55169	-0.0299	-0.669	-0.5331
TCGA-77	-0.3625	0.08728	-1.0809	0.16572	-0.2832	-0.9106	0.44675	-0.4735	0.54954	-0.5416	0.94716	-0.2692	1.48144	-0.3614	0.09872	1.21437	-0.6652	-0.3974	-0.4343	-0.5924	-0.1778	-0.6226
TCGA-33	-0.3625	2.86623	0.7748	-1.3407	-0.2832	-0.4367	-0.2403	-0.1264	-0.5526	-0.5416	-0.8413	0.443	-0.1376	-0.9051	-1.2536	-0.0722	-0.9589	-0.3974	-0.5926	-0.5924	0.3533	2.24771
TCGA-43	-0.3625	0.15137	-0.4125	-1.3449	-0.2832	-0.6905	0.16705	-0.4735	-0.5526	-0.5416	0.75695	-0.2692	2.07677	-0.9226	-0.0547	-0.5036	0.49616	-0.3974	-0.6123	-0.1756	-0.669	0.49791
TCGA-22	-0.3625	-0.2108	-0.9278	2.18142	-0.2832	-0.7787	-1.1257	-0.4735	-0.5526	-0.5233	-0.0292	-0.2692	-0.8276	0.5215	1.15968	-0.5036	4.26368	0.6599	-0.0818	1.56636	-0.669	-1.2254
TCGA-90	-0.3625	3.47462	-0.001	-0.1848	-0.2832	-0.2737	-0.0713	-0.4735	0.37004	-0.0552	-0.4087	1.26139	-0.8	-1.6638	-1.1693	2.57897	-0.9589	2.07626	-0.39	-0.5924	-0.1464	0.13379
TCGA-21	-0.3625	0.60459	-0.6572	0.57406	-0.2832	-0.4462	-1.1257	-0.4735	-0.5526	-0.3503	-0.8188	-0.2692	1.17576	-1.4318	-0.2626	0.34878	-0.9589	-0.3974	-0.5586	-0.5924	0.71115	1.28168
TCGA-NC	-0.3625	1.46741	0.67926	-0.4264	-0.2832	1.14728	0.39417	-0.4735	3.64658	1.41012	-0.3671	-0.2692	-0.7033	-0.9091	-1.1589	-0.5036	-0.8178	-0.3974	-0.568	-0.4903	-0.669	0.55189
TCGA-60	-0.3625	-0.3992	0.67721	-0.2906	-0.2832	1.31263	-0.3615	-0.4735	1.75714	-0.1494	0.27201	-0.2692	-0.8795	0.78374	0.21538	-0.5036	-0.1388	-0.023	1.09006	-0.5924	1.7626	-0.7249
TCGA-22	-0.3625	-0.2921	-0.0675	1.85263	-0.2832	0.97692	-0.4057	-0.4735	-0.5526	1.77164	0.47048	-0.2692	-1.0615	-0.8283	0.67391	-0.5036	0.22068	2.85015	-0.537	-0.5924	0.62763	-0.9356
TCGA-77	-0.3625	-0.8205	0.25555	0.09806	-0.2832	0.41245	0.24972	-0.4735	-0.3554	0.47321	-0.8182	-0.2692	1.51798	-0.501	-0.5857	-0.5036	0.74876	-0.3974	-0.6123	-0.5924	0.45077	-0.466
TCGA-NK	-0.3625	2.69786	-0.8998	-1.0374	-0.2832	-0.3519	-0.3225	-0.4735	0.30803	-0.5145	-0.8413	0.0623	0.36145	-1.7519	-0.8515	1.79083	-0.9589	-0.3974	-0.6123	-0.5924	-0.1023	1.82336
TCGA-56	-0.3625	-0.7309	-0.3983	-0.0712	-0.2832	0.036	0.05375	-0.4735	-0.5526	-0.5416	0.00073	-0.2692	1.33104	0.95105	0.36938	-0.5036	0.56073	0.01916	-0.591	1.25772	-0.6097	-1.2188
TCGA-63	-0.3625	-0.5282	0.3716	1.39127	-0.2832	0.92118	-0.252	-0.4735	1.59804	-0.4439	1.4045	-0.2692	-1.0055	0.61277	-1.0202	-0.5036	0.2753	0.05234	-0.3484	-0.5924	-0.5543	0.40228
TCGA-52	-0.3625	0.7957	-0.4863	0.41142	-0.2832	0.17924	-0.3885	-0.4735	-0.5526	-0.3399	-0.1347	-0.2692	-0.0088	0.0152	-0.1253	-0.5036	1.78516	-0.3974	-0.1612	0.28292	0.21538	-0.4634
GSM4420	1.10998	-0.905	2.39307	-1.5382	-0.2832	-0.1048	-0.9317	-0.4735	-0.5526	-0.5416	-0.4366	-0.2692	0.02865	-0.3234	-0.9141	0.18426	2.18985	-0.3974	2.51814	-0.5924	1.0841	0.01139
GSM4420	2.89237	-0.905	2.14299	-0.7252	-0.2832	0.31054	-1.1257	-0.4735	-0.5526	-0.5416	-0.8413	-0.2692	0.12565	-0.9376	-0.9036	1.18136	2.59564	-0.3974	0.01149	-0.3737	-0.669	-0.54
GSM4420	2.97762	-0.905	2.51245	-1.5382	0.85376	-0.0734	-1.1257	-0.4735	-0.5526	-0.5416	-0.7434	-0.2692	-0.3948	2.42634	-0.992	-0.3005	-0.6801	0.32576	0.05247	2.22838	-0.669	-0.1806
GSM4420	2.34582	-0.905	1.876	-0.9298	0.1957	-0.053	-1.1257	-0.4735	-0.5526	-0.5416	-0.4481	-0.2692	-1.308	0.73848	-1.0893	4.26752	-0.9589	-0.3974	1.12843	2.04326	-0.669	-0.2057
GSM4420	1.98792	-0.905	2.1175	-1.5382	0.51768	0.51141	-1.1257	-0.4735	-0.5526	-0.5416	-0.4461	-0.2692	-0.6995	1.72277	-1.1282	0.92497	-0.7013	3.07582	0.49868	3.32596	-0.669	-0.6876
GSM4420	3.58983	-0.905	2.20084	-1.5382	-0.1581	0.19185	-1.1257	-0.4735	-0.5526	-0.4795	-0.8079	-0.2692	-0.6949	-0.2057	-1.1226	3.67173	-0.9589	0.43466	1.5381	2.98759	-0.669	-0.9865
GSM4420	0.24659	-0.905	-0.0934	-1.5382	-0.2832	-0.5915	-1.1257	-0.4735	-0.41	-0.5416	-0.8413	-0.2692	0.45838	1.60362	0.30323	4.20089	-0.9589	-0.3974	1.42726	2.32674	-0.669	-1.0843
GSM4420	-0.3625	0.05822	0.76621	-1.5382	-0.2832	-0.1567	-1.081	-0.3311	2.50744	-0.5416	-0.6596	-0.2692	-0.2351	0.36006	-1.0921	1.45906	-0.8629	1.15898	2.72847	2.64115	-0.669	0.34171
GSM4420	1.44545	-0.905	1.65823	-1.5382	-0.2832	0.37579	-1.1257	-0.4735	-0.5526	-0.5416	-0.3551	-0.2692	-0.5292	0.55708	-0.6001	-0.5036	5.08169	-0.3974	0.0749	2.22632	-0.669	-0.5588
GSM4420	3.09286	-0.905	2.02739	-1.5382	0.86953	-0.1302	-1.1257	-0.4735	-0.5526	-0.5416	-0.6658	-0.2611	-1.315	1.69373	-1.2624	0.8052	0.49183	1.2466	0.68372	3.67617	-0.669	0.12821
GSM4420	5.12665	-0.905	2.31689	-1.5382	0.29994	0.64472	-1.1257	0.69163	1.04011	-0.5416	-0.8413	-0.2692	-0.7034	-0.3105	-1.4223	3.15119	-0.9589	-0.3974	-0.2102	-0.3921	-0.669	-0.3649
GSM4420	-0.0938	-0.905	2.15299	-0.9022	-0.2832	-0.6045	0.4165	-0.4735	-0.5526	-0.5416	-0.2404	-0.2692	-1.5147	0.26152	-0.6147	3.78851	-0.9589	-0.0031	1.92658	5.86032	-0.669	-0.5506
GSM4420	2.35518	-0.905	1.2249	-1.5382	1.04589	0.02903	-1.1257	-0.4735	-0.5526	-0.5416	-0.4341	-0.2692	-0.2671	1.0996	-2.1135	0.62536	2.74923	-0.3974	1.64272	1.92471	-0.669	-0.1803
GSM4420	4.72431	-0.905	2.6607	-1.5382	-0.2832	0.39051	-1.1257	-0.4735	1.79643	-0.5031	-0.4688	-0.2692	-0.6635	-1.2761	-1.8448	3.39286	-0.9589	-0.3974	0.19258	0.66291	-0.669	0.27173
GSM4420	3.4004	-0.905	1.68543	-1.5382	-0.2832	0.38119	-1.1257	-0.4735	-0.5526	-0.5416	-0.8413	-0.2692	-1.3975	1.90853	-0.9772	-0.5036	1.42105	0.34706	0.03436	3.66522	-0.669	0.4747
GSM4420	1.32594	-0.905	3.20491	-1.5382	-0.2832	-0.6675	-0.7722	-0.4735	-0.5526	-0.4724	-0.679	-0.2692	-0.328	0.39156	-1.1058	4.19528	-0.9589	-0.3974	1.11049	3.22437	-0.669	-1.0751
GSM4420	8.59766	-0.905	2.18891	-1.5382	2.25196	1.09906	-1.1257	-0.4735	-0.5526	-0.5416	-0.8413	-0.2692	-0.8937	0.35532	-1.5838	-0.0337	0.27138	-0.3974	0.04963	0.78819	-0.669	-1.1789
GSM4420	-0.1652	-0.905	0.81384	-0.7071	-0.2832	-0.8711	-1.0132	-0.1019	-0.5526	-0.5416	-0.0179	-0.2692	-0.361	1.13813	-0.9973	2.25267	-0.9589	-0.3974	1.66837	4.30269	-0.669	0.34015
GSM4420	3.01289	-0.905	3.52572	-1.2477	-0.2832	-0.5108	-1.1257	-0.4735	-0.5526	-0.5416	-0.4642	-0.2692	-1.0628	1.58035	-1.0417	0.40327	-0.9589	-0.3974	0.95138	3.3345	-0.669	0.01397
GSM4420	4.83862	-0.905	0.96271	-1.5382	1.29647	0.32288	-1.1257	-0.4735	1.35966	-0.5416	-0.8413	-0.2692	-0.7732	1.04464	-1.2641	1.82727	-0.9589	-0.3974	0.02815	2.88901	-0.669	-0.25
GSM4420	-0.3625	-0.5805	1.72254	0.08721	-0.2832	-1.1028	-1.1257	-0.4735	-0.5526	-0.5416	-0.8413	-0.2692	0.61379	0.82107	-0.7762	1.92981	-0.9589	0.08266	1.57475	2.88017	-0.669	-1.0614
GSM4420	-0.3625	0.99239	1.51261	-0.7028	2.13608	-0.5286	-1.1257	-0.4735	-0.5526	-0.5416	-0.391	-0.2692	0.8755	-1.0818	-1.1052	-0.181	-0.7763	0.15476	-0.6123	-0.5924	2.47998	0.59445
GSM4420	0.06997	-0.905	0.91541	-1.1149	8.043	-1.0378	-1.1257	-0.4735	-0.5526	-0.5416	-0.4738	-0.2692	1.56319	0.0626	-0.8331	-0.3469	-0.5988	-0.3974	0.14343	-0.5924	0.56609	0.69851
GSM4420	1.86964	-0.905	1.28847	-1.1106	-0.2832	-0.6736	-1.1257	-0.4735	-0.5526	0.76788	-0.8413	-0.2692	0.17677	0.57222	-1.013	1.53984	1.90381	-0.3974	2.43635	1.37818	-0.669	-1.2705
GSM4420	1.74431	-0.905	1.39491	-1.5382	-0.2832	-0.2947	-1.1257	-0.4735	-0.5526	0.50376	0.50248	-0.2692	-0.7034	1.57303	-1.0392	-0.3951	1.51466	-0.3974	3.7254	2.18526	-0.669	-0.6765
GSM4420	4.38728	-0.905	0.9564	-1.2944	-0.2832	0.21677	-1.1257	-0.4735	-0.5526	-0.5416	-0.2268	-0.2692	-1.1037	0.51608	0.06669	-0.5036	0.49019	-0.3974	2.95222	0.57477	1.26556	-0.197
GSM4420	11.7426	-0.905	1.10173	-1.5382	-0.2832	0.32951	-1.1257	-0.4735	-0.5526	-0.5416	-0.8413	-0.2692	-1.0738	-0.0872	-0.8149	-0.5036	0.35397	-0.3974	0.82425	-0.0079	-0.669	-0.5319
GSM4420	1.30835	-0.905	2.49261	-1.5382	-0.2832	0.36417	-1.1257	-0.4735	-0.5526	-0.5416	-0.5859	-0.2692	-0.7468	0.47731	-0.3313	0.93706	-0.9589	-0.3974	3.85403	3.91113	-0.669	-0.89
GSM4420	-0.3625	-0.8783	-0.3881	-0.7246	-0.2832	0.90451	-1.1257	-0.4735	-0.5526	-0.5416	-0.8413	-0.2692	-1.3628	-0.5791	-0.5723	-0.5036	1.9644	-0.3974	4.32986	3.52388	-0.669	2.00503
GSM4420	1.86717	-0.905	3.05428	-1.5382	-0.2832	-0.4766	-1.1257	-0.4735	-0.5526	-0.5416	-0.3557	-0.2692	-0.4933	0.11685	-0.4273	-0.5036	2.85511	-0.3974	1.72607	1.98476	-0.669	-0.3161
GSM4421	3.48024	-0.905	0.47017	-1.5382	-0.2832	0.05188	0.34518	-0.4735	-0.5526	-0.5416	0.60092	-0.2692	0.34339	0.16876	-1.6068	0.84114	-0.9589	1.00853	1.85838	1.08133	-0.669	-0.1031
GSM4421	1.90958	-0.905	0.8629	-1.5382	0.07555	0.15848	-1.1257	-0.4735	-0.5526	-0.5416	-0.1531	-0.2692	-0.8264	1.14378	-0.9639	0.44566	2.33189	1.17094	2.96314	4.3395	-0.669	-1.209
GSM4421	0.30726	-0.905	-0.1843	-1.5382	-0.2832	0.36608	-1.1257	-0.4735	1.95626	-0.5416	-0.0868	-0.2692	2.37149	-0.4066	-0.8501	1.46563	-0.8468	-0.3974	-0.0069	0.80136	-0.5005	-0.758
GSM4421	-0.1341	-0.905	1.35583	-1.5382	-0.2832	-0.3358	0.1761	-0.4735	-0.5526	-0.5416	0.24226	0.65505	-0.9537	1.52959	-1.3362	0.41914	-0.9589	-0.3974	2.0233	4.27249	-0.669	1.4407
GSM4421	2.31956	-0.905	1.75287	-1.4832	-0.2832	-0.5048	-1.1013	-0.4735	-0.5526	-0.5416	1.10398	2.63208	-0.9277	0.77995	-0.841	0.30128	0.90612	-0.3974	2.13253	3.04478	-0.669	-0.3064
GSM4421	-0.3625	0.37381	2.38174	-1.5382	0.55214	-0.2297	-1.1257	0.42609	-0.5526	-0.5416	-0.8413	-0.2692	0.0888	0.92987	-0.6113	-0.5036	-0.2009	-0.3974	3.29759	1.54676	-0.669	-0.7324
GSM4421	1.54476	-0.905	-0.3913	-1.5382	-0.2832	1.20297	-1.1257	-0.4735	-0.5526	-0.5416	1.43495	-0.2692	0.08913	-0.0298	-1.1809	-0.5036	2.91175	1.49093	2.38785	2.45539	-0.669	-1.1507
GSM4421	2.49336	-0.905	2.46238	-0.8833	-0.2832	-0.6586	-1.1257	-0.4735	-0.5526	-0.5416	-0.5414	-0.2692	-0.7632	0.019	-0.8919	3.02867	-0.9589	-0.3974	4.41556	1.55911	-0.669	-0.8243
GSM4421	2.78138	-0.905	1.62826	-1.2501	-0.2832	-0.3429	-1.1257	-0.4735	-0.5526	-0.0128	-0.6969	-0.2692	-1.4272	-0.0048	-1.154	-0.1934	5.38603	-0.3974	4.08158	1.83164	-0.669	-0.3238
GSM4421	-0.3625	-0.5625	-1.0809	-1.5382	-0.2832	-0.0723	-0.8203	-0.4735	0.97115	-0.2373	-0.8413	-0.2692	0.88734	0.48387	-1.6165	5.5687	-0.9589	-0.3974	1.95016	3.73015	-0.669	-1.2705
GSM4421	2.08156	-0.905	1.49156	-1.5382	-0.2832	0.07125	-0.5707	-0.4735	-0.5526	-0.5416	-0.5411	-0.2692	-0.1295	2.43693	-1.1157	-0.5036	1.35547	-0.3974	1.88421	1.60044	-0.669	-0.7848
GSM4421	2.56388	-0.905	1.04214	-0.0221	-0.2832	-0.6993	-1.1257	-0.4735	-0.5526	-0.5416	-0.4775	-0.2692	-0.3629	-0.1899	-1.2103	1.93899	-0.5052	0.39491	1.77276	3.81013	-0.669	-0.3166
GSM4421	2.29487	-0.905	1.46331	-1.5382	-0.2832	0.0254	-1.1257	-0.0523	-0.5526	-0.5416	-0.3393	-0.2692	-0.8859	-0.3917	-0.9224	0.31045	2.69767	-0.3974	2.49639	4.23996	-0.669	-0.2455
GSM4421	0.75226	-0.905	-0.7506	-0.7367	-0.2832	0.24745	-1.1257	-0.4735	-0.5526	-0.5416	0.65816	-0.2692	-1.456	0.73918	-1.11	0.37614	5.76998	3.76278	1.27312	6.20498	-0.669	-1.2705
GSM4421	3.20243	-0.905	0.60675	-1.5382	0.27377	-0.8099	-1.1257	-0.4735	-0.5526	0.34524	1.30688	-0.2692	-1.5147	-0.1831	-1.2972	0.05235	3.75623	-0.3974	5.81211	4.40057	-0.669	-0.9989
GSM4421	1.38572	-0.905	0.98094	-1.5382	-0.2832	1.28116	-1.1048	-0.4735	-0.5526	-0.5416	0.8418	-0.2692	-0.5783	0.74764	-0.6345	-0.4583	1.55515	-0.3974	2.6946	0.68926	-0.669	-0.4195
GSM4421	5.78685	-0.905	2.99755	-1.5382	-0.138	0.64571	-1.1257	-0.4735	-0.5526	-0.5416	-0.5148	-0.2692	-1.1392	-0.0237	-0.7135	-0.5036	2.10366	-0.3974	0.82408	1.69701	-0.669	-1.0134
GSM4421	0.83354	-0.905	0.87832	-1.5382	-0.2832	0.99785	-1.1257	-0.4735	1.43207	-0.5416	-0.8413	-0.2692	0.73548	-0.6315	-0.5258	1.29499	-0.9589	-0.3974	0.15632	0.67241	-0.669	0.64216
GSM4421	1.30696	-0.905	2.59985	-1.467	-0.2832	-0.1772	-1.1257	-0.4735	-0.5526	-0.5416	-0.2319	-0.2692	-1.1899	-0.4809	-0.8912	0.96956	2.59259	-0.3974	1.79975	0.96099	-0.6336	1.23349
GSM4421	2.00043	-0.905	1.22901	-1.5382	-0.2832	-0.3069	-0.5777	-0.4735	-0.5526	-0.5416	-0.7183	-0.2692	0.39372	-0.0175	-0.3787	-0.5036	3.76147	-0.3974	2.05579	0.04228	-0.669	-0.4015
GSM4421	2.65592	-0.905	1.2576	-1.41	-0.2832	-0.0382	-1.1257	-0.4735	-0.5526	-0.5402	-0.0747	-0.2692	-0.7814	0.5597	-1.3623	-0.1106	2.38668	-0.3974	1.51243	3.23645	-0.669	0.35784
GSM4421	3.84226	-0.905	3.02829	-1.5319	-0.2832	0.08009	-1.1257	-0.4735	-0.5526	-0.5226	-0.5775	-0.2692	-0.4223	0.63304	-2.0316	2.15766	-0.9589	-0.3974	1.79289	-0.5924	1.81641	-0.4832
GSM4421	2.72878	-0.905	2.70294	-1.5382	0.35389	0.31592	-1.1257	-0.4735	-0.216	-0.5416	-0.8413	-0.2692	0.03527	0.61109	-1.5547	1.08702	-0.9589	-0.3974	0.33639	1.96693	-0.669	-0.0378
GSM4421	1.49983	-0.905	1.43118	-1.5382	-0.0905	-0.2131	-1.1257	-0.4735	-0.5526	-0.5416	-0.5793	-0.2692	-0.097	-0.2738	-0.9024	0.71359	2.80866	-0.3974	2.44818	2.12913	-0.669	-0.1589
GSM4421	-0.3625	0.13713	2.30173	-1.5382	-0.2832	0.13624	-1.1257	-0.4735	-0.5526	-0.5416	-0.8033	-0.2692	-1.3589	1.37478	-0.352	-0.5036	2.64701	-0.3974	-0.6123	5.43195	-0.669	0.04909
GSM4421	2.2369	-0.905	1.36963	-1.5382	-0.2832	-0.4024	-1.1257	-0.2868	0.23549	-0.5416	-0.8413	-0.2692	-0.0591	-0.0891	-2.1135	2.39074	2.7005	-0.2553	1.37772	1.76084	-0.669	0.04019
GSM4750	-0.3625	-0.905	2.76833	-1.3966	-0.2832	0.1913	1.51928	-0.4735	-0.5526	-0.3893	0.42207	-0.2692	-0.9186	1.35205	-0.1673	-0.5036	-0.0205	-0.3974	0.06844	0.62679	0.08746	0.48983
GSM4750	0.9881	-0.905	1.09392	0.66653	-0.2832	-1.1028	1.2475	1.54798	-0.5526	0.69828	-0.6129	-0.2692	-1.4119	1.5439	0.86258	-0.5036	1.48328	-0.3974	-0.5449	0.35493	-0.669	-0.491
GSM4750	1.64661	-0.905	-1.0809	1.33113	5.54924	-1.1028	1.01164	-0.4735	-0.5526	-0.0055	1.24064	-0.2692	0.08782	0.09355	-0.963	-0.1532	0.02579	-0.3974	0.20278	-0.2006	-0.3332	-0.1563
GSM4750	-0.3625	-0.905	-0.0951	0.16466	2.23306	-1.1028	2.46164	0.75816	-0.5526	-0.256	0.02279	-0.2692	-0.1093	-0.7032	-0.3235	-0.388	-0.3876	-0.3974	-0.2388	-0.5924	1.24855	1.85529
GSM4750	-0.3625	-0.4024	-1.0809	0.7817	-0.2832	-0.349	0.92223	-0.4735	-0.5526	0.20832	-0.3846	0.0292	-0.8843	-1.123	0.40442	0.93804	-0.9589	1.06649	0.34126	-0.5924	1.843	2.01448
GSM4750	0.08787	-0.905	0.79574	-1.1098	-0.2832	-1.1028	2.43653	3.36746	-0.5526	0.60238	0.45127	-0.2692	-0.9091	1.87062	0.33222	-0.5036	0.25379	-0.3974	-0.6123	1.32347	-0.669	-0.2685
GSM4750	-0.3625	0.43997	-0.1493	0.58563	1.49777	-1.1028	-0.3638	0.93617	-0.5526	1.63933	-0.8413	-0.2692	-0.2191	-0.4518	-1.0326	-0.5036	-0.8297	-0.3974	-0.2939	-0.5924	-0.1903	2.28703
GSM4750	0.59304	-0.905	-0.0093	0.36297	-0.2832	-0.8704	0.69052	0.83408	-0.5526	1.23924	0.65969	-0.2692	-0.9611	1.12335	0.83268	-0.3148	-0.648	-0.3974	0.03212	0.28271	-0.158	0.21944

GSM4750	1.14482	-0.905	-1.0809	1.22289	-0.2832	-1.1028	1.24526	-0.4735	-0.5526	4.76351	-0.8413	-0.2692	-0.535	-0.9819	0.73111	-0.5036	0.09118	-0.3974	-0.6123	-0.0417	0.16898	0.53184
GSM4750	0.4481	-0.905	0.37329	-0.3084	0.11806	-0.6568	-0.0567	1.17743	-0.5526	-0.4427	0.00905	-0.1425	-0.4343	1.22439	0.5522	1.05492	-0.9589	-0.3974	0.36612	-0.5924	0.50786	0.25895
GSM4750	0.70025	-0.905	0.06999	-0.06	-0.2832	-1.0467	0.40474	-0.4735	-0.5526	2.31088	-0.8413	-0.2692	0.59243	-0.5377	0.5544	-0.2099	-0.7407	-0.3974	0.27863	-0.5924	3.34448	-0.3036
GSM4750	-0.3625	-0.6667	-0.2314	0.54566	-0.2832	-0.6915	-0.6094	2.61881	-0.5526	-0.5416	-0.0641	0.56329	-0.4064	0.23554	0.46707	1.35547	-0.9589	-0.3974	-0.1593	-0.5924	-0.669	0.95124
GSM4750	2.51925	-0.905	-0.208	0.25659	1.80215	-0.6119	-0.5269	-0.4735	-0.5526	-0.5416	0.53336	-0.2692	0.52216	-0.609	0.11555	0.02024	-0.9043	-0.3974	-0.6123	-0.5924	3.03461	-0.1692
GSM4750	-0.3625	-0.6945	0.19932	1.07653	-0.2832	-1.1028	-1.0356	1.59731	-0.5526	-0.5416	0.03002	-0.2692	-0.6271	1.45945	0.71444	-0.5036	0.52127	-0.3974	0.04097	-0.5469	-0.6483	0.79662
GSM4750	-0.2563	-0.905	-0.1466	0.56492	-0.2832	-1.1028	-1.1257	-0.4735	-0.5526	-0.5416	-0.8413	-0.2692	1.11089	-0.7172	-0.093	2.5097	-0.9589	-0.3974	0.46797	-0.5924	1.39665	0.56676
GSM4750	-0.3625	-0.0334	0.45526	-0.8691	-0.2832	-0.5513	1.68095	1.18769	-0.5526	-0.5416	-0.5211	-0.1953	-0.7155	0.93306	0.57544	-0.259	-0.4401	-0.3974	-0.4595	-0.5924	-0.6176	1.70319
GSM4750	-0.3625	-0.4025	-1.0302	-1.3985	2.98114	-1.1028	2.2157	-0.4735	2.21378	0.03911	-0.8413	0.04443	0.41376	-0.5972	-0.1979	1.69353	-0.9589	-0.3974	-0.0885	-0.5924	4.38111	0.67219
GSM4750	-0.3625	-0.7169	1.34269	-1.1321	1.92095	-0.4675	0.07899	1.51363	-0.5526	-0.5416	-0.5967	-0.0992	-0.1434	1.13968	0.69036	0.56341	-0.9589	-0.3974	0.15648	-0.2254	-0.669	0.2965
GSM4750	-0.3625	-0.905	1.14843	-0.7423	-0.2832	-0.1152	0.88639	1.00344	-0.5526	-0.5416	-0.1345	-0.2692	-0.5603	0.95724	0.02604	-0.1495	-0.7791	-0.3974	0.1158	0.31667	-0.669	1.39639
GSM4750	0.45669	-0.905	0.30085	-0.6372	0.49154	-0.7765	1.03818	0.22696	-0.5526	-0.5416	1.56511	-0.2692	-0.9837	2.15601	0.43731	-0.3028	-0.5947	-0.3974	0.1037	-0.5924	0.72652	0.78543
GSM4750	-0.0858	-0.905	0.03842	0.90105	1.29819	-1.1028	1.77148	-0.4735	-0.5526	-0.1117	-0.5164	-0.1559	0.03346	0.14009	-0.0819	0.80806	-0.9589	-0.3974	0.32131	-0.5924	3.57881	-0.2407
GSM4750	-0.3625	-0.4915	-0.91	0.93335	-0.2832	-1.1028	1.52652	2.2878	-0.5526	-0.5416	-0.306	-0.2692	0.27523	0.00727	0.63728	-0.1046	-0.9589	-0.3974	1.57592	-0.5081	-0.669	0.06028
GSM4750	-0.3625	-0.4546	-0.0161	-0.2049	0.13479	-1.1028	0.99689	2.69985	-0.5526	-0.5416	-0.4939	-0.2692	-0.3971	0.61706	-0.0453	-0.2078	-0.8419	-0.3974	-0.2949	-0.3354	-0.669	2.19775
GSM4750	0.20688	-0.905	0.68933	-0.3163	3.35718	-1.1028	1.63758	0.07791	-0.5526	0.09936	-0.3031	-0.2692	-1.0349	0.88896	0.70586	-0.0568	-0.4742	-0.0881	0.0394	-0.2301	1.07026	0.93447
GSM4750	0.38669	-0.905	-0.5243	1.64725	0.8371	-1.1028	-1.1257	-0.4735	-0.5526	-0.2988	-0.4431	-0.2692	0.24923	-0.2917	0.0395	-0.5036	0.01259	-0.3974	-0.5194	-0.5924	1.05735	1.74465
GSM4750	0.28728	-0.905	-0.687	0.17914	-0.2832	-0.4679	0.36956	1.0279	-0.5526	0.26117	-0.8413	-0.2692	-0.6101	2.17657	1.74417	-0.5036	-0.4099	-0.3974	-0.1289	0.85806	-0.669	-0.1028
GSM4750	0.82079	-0.7035	0.05681	0.21117	-0.0723	-0.2952	0.79907	-0.4735	-0.5526	-0.5416	1.99477	-0.2692	-0.1304	0.45986	-0.9952	-0.5036	1.5836	-0.3974	-0.6123	0.42727	1.45568	-0.495
GSM4750	3.38743	-0.905	-1.0809	0.84853	1.0991	-1.1028	2.36189	-0.4735	-0.5526	0.30933	3.37668	-0.2692	-0.4153	-0.5298	-1.4656	0.89535	-0.3609	0.51145	-0.6123	-0.5924	-0.2377	-0.05
GSM4750	-0.3625	-0.8464	-0.0839	0.05169	-0.2832	-1.1028	0.04226	2.5887	-0.5526	-0.5416	-0.182	-0.2692	0.01063	0.76573	1.73665	0.19027	-0.9589	-0.3974	0.02549	0.61798	-0.669	-0.0612
GSM4750	-0.3625	-0.7845	0.14226	1.00661	-0.2832	-0.866	-0.3985	0.97971	-0.5526	-0.5416	-0.7845	-0.2692	0.93552	0.85026	-0.2613	-0.0505	-0.9589	-0.3974	-0.1449	-0.5924	-0.669	0.53853
GSM4750	-0.3625	-0.5886	-0.7813	1.20725	-0.2832	-1.1028	-1.0239	-0.2491	-0.5526	-0.5416	-0.8413	-0.2692	-0.5584	0.01148	0.4801	0.0283	0.0814	-0.3974	-0.5609	-0.5924	0.44407	3.23527
GSM4750	0.82482	-0.905	-0.3317	0.76483	-0.2832	-0.9904	-0.2073	-0.4735	-0.5526	-0.5416	-0.8413	-0.2692	1.24813	-0.5117	1.43022	0.2176	-0.9589	-0.3974	-0.6123	-0.5924	2.76281	-0.6354
GSM4750	-0.3625	-0.905	-1.0809	-0.2923	2.32383	-1.1028	0.52848	-0.4735	-0.5526	-0.3484	0.01629	-0.2692	0.17497	-1.1607	0.32427	0.07062	-0.7967	-0.3974	-0.3043	-0.5924	1.44969	3.36271
GSM4750	-0.3625	-0.905	1.13597	-1.0584	-0.162	-0.593	0.52449	3.82267	-0.5526	0.22025	-0.8413	-0.2692	-1.1034	5.55021	-0.7389	-0.5036	0.42441	-0.3974	-0.2642	-0.5924	0.92967	-0.8721
GSM4750	0.87158	-0.905	-0.0671	0.21981	1.64436	-1.1028	-0.6335	-0.4735	-0.5526	0.41071	0.31161	1.92499	-0.2841	-1.3035	0.70783	2.18288	-0.9589	-0.3974	0.44324	-0.4885	0.14784	0.89922
GSM4750	-0.3625	-0.4366	-0.8887	0.57927	-0.2832	-1.1028	0.2798	0.70453	-0.5526	-0.285	-0.7334	-0.2692	0.21959	-0.584	0.01277	2.56553	-0.9589	-0.3974	0.27191	-0.0989	-0.2956	1.09837
GSM4750	-0.3625	-0.9005	0.01975	-0.1584	0.70977	-1.1028	0.51394	1.13334	-0.5526	-0.54	-0.2111	-0.2692	-0.4841	0.74942	0.69723	1.41735	-0.9589	-0.3974	0.3405	0.44753	-0.669	1.09772
GSM4750	0.48959	-0.905	-0.4058	-0.0703	-0.2832	-0.0731	-0.0786	-0.4735	-0.5526	-0.5416	2.88586	-0.2692	-1.5147	0.5739	1.83408	-0.5036	1.98819	0.41165	0.12527	0.49963	0.34311	-0.4346
GSM4750	-0.3625	-0.905	1.35196	0.01899	2.97843	-0.9982	0.20433	0.5731	-0.5526	0.50942	-0.7039	0.08411	0.09952	0.34025	-0.1546	0.45219	-0.9589	-0.3974	-0.6123	-0.5924	0.7545	0.55273
GSM4750	-0.3625	-0.407	0.8987	-0.8173	-0.2832	-0.1585	0.33126	1.3582	-0.5526	-0.5416	0.15583	-0.2692	-0.9938	2.11409	0.67609	-0.1305	-0.9589	-0.3974	-0.0066	0.40328	-0.669	0.59215
GSM4750	1.4506	-0.905	1.05753	-1.5382	-0.2832	0.61117	2.43571	-0.429	-0.5526	-0.5416	0.87766	0.11301	-0.6804	1.05208	1.12755	-0.35	-0.9589	-0.3974	-0.1627	0.50207	-0.669	-0.4597
GSM4750	-0.3625	-0.7299	-0.8941	-0.2183	1.61022	-1.1028	-0.3495	-0.3746	-0.5526	0.26194	-0.8413	-0.2692	1.10119	-0.6975	0.24272	1.32186	-0.9589	-0.3974	0.67968	-0.5924	0.23776	1.32252
GSM4750	-0.3625	-0.5064	-0.4411	0.87738	-0.2832	-0.7756	-0.5989	-0.4735	-0.5526	0.83437	3.13917	-0.2692	-1.4127	-0.0606	0.46109	0.77709	1.05503	2.73053	-0.0839	-0.5508	0.98403	-0.0826
GSM4750	-0.3625	-0.8142	0.53087	-0.6631	-0.2832	-0.901	-0.515	1.58406	-0.5526	-0.3268	-0.7382	-0.2692	-0.306	1.44519	1.56893	-0.5036	-0.1565	-0.3974	-0.3257	-0.5924	0.19508	1.13652
GSM4750	1.30512	-0.905	0.08152	-0.1458	1.1379	-1.1028	0.23646	-0.3233	-0.5526	-0.2503	0.45414	-0.2692	1.2632	-0.8222	0.79852	0.02349	-0.6458	-0.3974	-0.1357	-0.4331	-0.6243	-0.046
GSM4750	-0.3625	-0.6284	-0.3841	-0.3393	-0.2832	-1.1028	-0.3401	3.1146	-0.5526	-0.5416	-0.0858	-0.2692	0.18515	0.40039	0.49745	0.40944	-0.9589	-0.3974	0.28172	-0.5623	-0.669	1.34163
GSM4750	0.44656	-0.905	-0.4868	0.26542	-0.2832	-1.1028	1.38336	2.23877	-0.5526	-0.4963	0.34346	2.04505	-0.8905	-0.3054	1.43198	1.74499	-0.9589	-0.3974	1.1707	-0.0862	-0.669	0.04676
GSM4750	-0.3625	-0.7771	-0.3808	0.42286	0.08209	-1.1028	-0.7587	0.79332	-0.5526	1.69133	-0.0085	0.37416	0.0186	0.36163	0.54319	-0.1665	-0.9589	-0.3974	0.93889	-0.5924	-0.0708	0.80321
GSM4750	0.31039	-0.905	-0.2493	0.43154	0.97332	-1.1028	0.68464	-0.4735	-0.5526	-0.4389	0.33254	-0.2692	-0.0816	0.72501	-0.3035	0.21022	-0.9589	-0.3974	0.05628	-0.0446	-0.3132	1.7261
GSM4750	-0.3625	-0.4893	0.28302	-0.6967	-0.2832	-0.5077	2.85464	-0.4735	-0.5526	-0.0395	-0.4411	-0.2692	-0.925	1.12382	0.22109	-0.5036	0.79273	-0.3974	-0.443	0.02561	-0.0756	1.61221
GSM4750	-0.3625	-0.353	0.17647	0.32828	0.88343	-0.8049	-0.464	2.675	-0.5526	-0.5114	-0.6214	-0.2692	-0.4441	0.63419	0.28179	-0.3198	-0.8002	-0.3974	0.11004	-0.5924	-0.669	1.49288
GSM4750	0.41482	-0.905	-0.4638	1.30224	-0.2832	-0.753	1.22652	-0.4735	-0.5526	1.16652	2.58107	-0.2692	-1.5147	0.06775	0.75484	0.39784	-0.9589	-0.3974	-0.1683	-0.5924	3.01281	-0.1818
GSM4750	-0.2138	-0.905	1.64019	-1.2667	-0.2832	0.2788	0.14874	2.48717	-0.5526	2.40873	0.17453	-0.2692	-1.0637	1.66545	-0.1007	-0.3537	-0.6862	-0.3974	-0.4516	0.17864	-0.669	-0.0032
GSM4750	-0.3625	-0.4925	0.5554	-0.3198	-0.2832	-1.051	0.24699	4.19933	-0.5526	1.30121	0.35381	4.2638	-0.6213	0.56068	-0.2823	-0.2762	-0.8339	-0.3974	-0.133	-0.5924	-0.669	0.5573
GSM4750	-0.3625	-0.905	0.19823	-0.1583	0.01414	-0.8856	-0.6911	1.00605	-0.5526	-0.5416	-0.8413	-0.2692	2.2554	-0.0164	-0.2661	-0.502	-0.865	-0.3974	-0.4731	-0.5924	-0.3269	0.57528
GSM4750	-0.0676	-0.905	1.30451	-0.713	0.71279	-1.0283	-0.8544	1.56644	-0.5526	0.00456	-0.7157	-0.2692	-0.6709	-0.2021	0.68395	-0.1238	-0.9589	-0.3974	0.99997	-0.3706	-0.669	2.68954
GSM4750	-0.2869	-0.9035	-1.0809	1.15249	-0.2832	-1.1028	-0.6834	1.3239	-0.5526	1.51401	-0.3812	1.27369	-1.1549	-0.0776	1.26636	-0.2182	-0.1211	-0.3974	1.86405	-0.3849	-0.669	1.60565
GSM4750	-0.3625	-0.7045	0.24797	0.05066	-0.2832	-0.7941	0.96633	0.24589	-0.5526	0.05072	1.70429	3.98652	-1.1044	0.10445	1.15299	-0.5036	1.04362	-0.3974	0.28724	-0.1626	-0.669	0.39048
GSM4750	0.1738	-0.905	-0.0614	1.99617	-0.2832	-1.1028	0.74655	-0.4735	-0.5526	3.96054	-0.8413	-0.2692	-0.4036	-1.2065	-0.3302	0.78565	-0.9589	-0.3974	-0.3382	-0.5924	2.63844	0.04126
GSM4750	-0.3625	-0.8778	-0.4164	1.12473	-0.2832	-1.1028	0.95177	2.72628	-0.5526	-0.3027	-0.3517	-0.2692	-0.7182	0.82898	-0.497	-0.5036	-0.0635	-0.3974	-0.0786	-0.2003	-0.669	1.97855
GSM4750	0.19364	-0.905	-0.1017	-0.2479	-0.2832	-1.095	0.97865	0.74636	-0.5526	-0.5416	-0.8413	-0.2692	0.5476	1.94192	-0.3892	2.35623	-0.9589	-0.3974	-0.4591	-0.5924	1.10198	-0.413
GSM4750	0.16838	-0.905	0.88449	-1.3422	0.24976	0.06059	2.10534	0.19166	-0.5526	0.02043	0.62757	-0.2692	-1.0485	2.22902	-1.088	-0.5036	0.04647	-0.3974	0.2473	-0.324	-0.2472	1.42859
GSM4750	1.03796	-0.905	0.27356	0.48307	0.59547	-0.2045	0.12327	-0.0064	-0.5526	0.91669	0.81366	-0.2692	-1.056	0.07237	1.02831	-0.2763	-0.4244	-0.3083	0.23783	-0.5924	1.11081	-0.0512
GSM4750	-0.3625	-0.8071	0.94705	-0.5098	-0.2832	-1.04	-0.8119	4.33132	-0.5526	-0.5416	-0.4888	-0.2692	-0.252	3.45933	-0.2731	-0.5036	-0.8903	-0.3974	-0.3772	-0.5924	-0.669	0.52447
GSM4750	-0.3625	-0.842	0.83979	-0.2697	1.75787	-0.9464	-1.1257	-0.3915	-0.5526	-0.5416	0.54126	-0.2692	0.52174	-0.6344	0.23406	-0.4452	-0.4959	-0.3974	-0.4596	-0.5924	0.23051	2.12235
GSM4750	0.99651	-0.905	-0.8852	0.16878	1.88931	-1.1028	1.22248	1.95282	-0.5526	-0.2255	-0.6354	-0.2692	0.46002	2.05447	-0.5758	-0.5036	-0.5648	-0.3974	-0.1324	-0.5924	1.00921	-0.1768
GSM4750	1.38882	-0.905	-0.5391	0.23669	-0.2832	-1.1028	1.48745	-0.4735	-0.5526	-0.5416	0.58165	-0.2692	0.06794	0.03229	0.78646	0.63981	-0.9589	-0.3974	1.32149	-0.5924	2.85418	-0.6386
GSM4750	-0.3625	-0.806	-0.9715	1.20125	0.49513	-1.1028	-0.5405	1.4058	-0.5526	0.70196	-0.8413	1.92194	0.06666	8.14E-05	0.10829	0.57305	-0.9589	-0.3974	0.73639	-0.5924	0.43849	0.89885
GSM4750	0.47406	-0.905	-1.0809	0.34387	-0.2832	-0.977	0.92968	-0.4735	-0.5526	2.33167	1.05729	-0.2692	-0.4701	-0.2937	0.51202	0.01799	-0.4312	-0.3974	0.69081	-0.5924	1.67205	0.53787
GSM4750	2.30862	-0.905	-1.0809	-0.4403	4.16636	-1.1028	3.08584	-0.4735	-0.5526	-0.4687	1.60353	-0.2692	-0.9663	0.65623	0.18356	0.37305	-0.9589	-0.3974	-0.6123	-0.5924	3.15919	0.35868
GSM4750	0.1509	-0.905	1.59666	-0.4721	-0.2832	-1.0955	0.84201	4.41727	-0.5526	-0.4724	0.78215	3.81332	-0.1819	1.05505	-0.0425	-0.5036	-0.2988	-0.3974	0.07798	-0.5793	-0.669	-0.8718
GSM4750	-0.3625	-0.8389	1.32603	-0.0639	-0.2832	-1.0463	0.85398	3.88744	-0.5526	-0.5416	-0.2885	-0.2692	-0.3614	2.88228	-0.7933	0.13789	-0.9589	-0.3974	-0.172	-0.5924	-0.6144	-0.0837
GSM4750	2.22036	-0.905	0.02184	-1.3562	-0.2832	-0.7098	2.04443	1.22454	-0.5526	-0.118	2.40359	-0.2692	-0.6375	0.40599	0.52975	-0.5036	1.1438	-0.3974	-0.3259	0.71358	-0.5831	-0.4603
GSM4750	0.57778	-0.905	-1.0809	0.41141	-0.2832	-1.1028	1.76394	-0.4735	0.44634	0.99097	1.13741	-0.2692	-1.2539	-0.0982	1.72911	0.37406	-0.9589	-0.3974	-0.6123	1.01434	4.90913	-0.3371
GSM4750	-0.3625	-0.905	-0.2217	0.15464	0.41695	-1.1028	0.70543	4.24517	-0.5526	-0.5416	-0.4532	-0.2692	0.43757	1.17625	-0.7486	-0.5036	0.09533	-0.3974	-0.0163	-0.5924	-0.669	0.57237
GSM4750	-0.2601	-0.905	2.4143	-1.1943	-0.2832	-0.1491	0.07411	0.41221	-0.5526	-0.5416	-0.2232	-0.2692	-0.4091	2.19549	0.94261	0.31144	-0.8067	-0.3974	-0.3882	-0.5924	0.632	-0.4469
GSM4750	-0.3625	-0.7263	-0.8867	-0.3675	-0.2299	-1.1028	0.49569	-0.4735	-0.4564	-0.5416	-0.0124	-0.2692	1.47928	0.13026	0.62349	0.78191	-0.9589	-0.3974	-0.1211	-0.3977	-0.1061	0.65019
GSM4750	-0.3625	-0.884	-0.5596	1.23033	-0.2832	-1.1028	0.08528	1.98871	-0.5526	-0.5416	0.62606	-0.2692	0.62334	0.55803	-0.0854	-0.1065	-0.9589	-0.3974	-0.3739	-0.5924	-0.2434	0.39073
GSM4750	-0.3625	-0.5483	0.25207	-0.8956	-0.2832	-1.066	2.80422	2.47977	-0.5526	-0.203	-0.1775	-0.2692	-1.3503	0.62416	-0.185	0.02168	-0.9589	-0.3974	-0.1345	-0.1977	-0.669	3.15407
GSM4750	-0.1811	-0.7031	-1.031	0.66581	-0.2832	-1.1028	0.75761	1.77063	-0.5526	-0.5164	0.11239	-0.2692	-0.1285	0.88423	-0.316	-0.5036	0.53142	-0.3974	-0.3085	0.80662	-0.669	1.25628
GSM4750	-0.3625	-0.6173	1.63066	-0.6216	1.22384	-0.9305	-1.1257	3.12431	-0.5526	0.60936	0.02049	-0.2692	-1.1764	0.26339	0.28097	-0.5036	-0.0567	-0.3974	-0.2858	-0.5924	-0.669	2.09167
GSM4750	-0.3625	-0.7576	-0.0702	0.72456	0.32565	-1.0682	-0.3238	0.59902	-0.5526	-0.5165	-0.8413	-0.2692	0.17703	0.72913	-0.7307	0.81231	-0.9589	-0.3974	-0.2941	-0.4719	-0.669	2.19446
GSM4750	-0.3625	-0.905	3.55186	-1.5382	0.17582	0.84125	0.49879	2.48644	-0.5526	-0.2187	-0.3063	-0.2692	-1.1289	0.68798	-0.9816	-0.5036	0.16553	-0.3974	-0.1688	-0.5924	-0.547	0.97462
GSM4750	-0.3625	-0.6843	-0.8006	0.35707	-0.2832	-1.1028	1.69802	-0.4735	-0.5526	1.10165	-0.3415	0.26353	-0.5738	-0.2871	0.06803	2.35191	-0.9589	0.28347	0.40729	-0.5924	3.49932	0.41824
GSM4750	0.99904	-0.905	-0.6359	-0.4704	-0.2525	-1.0607	0.98383	-0.2033	-0.5526	0.07545	-0.8413	-0.2692	1.26361	1.32056	0.61815	0.4577	-0.9589	-0.3974	-0.1921	-0.5924	0.43848	-0.4248
GSM4750	-0.3625	-0.4899	0.42906	-0.4032	0.39159	-0.5367	-0.5639	-0.4735	-0.5526	-0.3279	-0.8413	-0.2692	-0.7022	-0.7321	-0.765	-0.5036	0.42825	-0.3974	0.07218	0.66469	-0.3888	4.27622
GSM4750	-0.1085	-0.905	-0.7349	-0.7428	-0.2832	-1.1028	2.07922	-0.4735	-0.5526	5.92618	0.54989	-0.2692	-1.3038	-0.8581	0.38756	-0.3611	-0.7832	-0.3974	3.32841	-0.5924	1.63725	0.92664
GSM4750	-0.3625	-0.905	1.19558	-0.6249	0.90414	0.06622	1.35394	0.15344	-0.5526	-0.1194	0.32315	-0.2692	-0.3506	1.55864	0.27687	-0.5036	0.22864	-0.3974	-0.1697	0.33308	-0.4766	-0.4968
GSM4750	-0.3086	-0.8343	3.31181	-0.2933	-0.2832	-1.0436	-0.8921	2.83496	-0.5526	-0.5416	-0.5579	-0.2692	-0.3785	1.92252	0.69219	-0.4029	-0.9411	-0.3974	-0.3898	1.35438	-0.669	-0.8981
GSM4750	1.46256	-0.905	-0.9205	0.14356	2.52825	-1.1028	0.95556	-0.4735	-0.5526	3.61269	-0.6229	-0.2692	-0.8567	0.23578	0.81858	-0.5036	1.26273	-0.3974	0.14848	0.08629	1.86233	-0.5568
GSM4750	-0.3625	-0.897	0.1786	-0.1167	1.13103	-1.1028	-0.1534	1.6758	-0.5526	-0.5416	-0.8413	-0.2692	1.08754	-0.3372	0.38931	-0.5036	-0.6003	-0.3974	-0.6123	-0.5924	0.4101	1.24391
GSM4750	-0.3625	-0.5994	3.64007	-1.5382	-0.2832	0.93299	-1.1257	2.34199	-0.5526	-0.5416	-0.7403	-0.2692	-0.5745	0.54262	-0.2478	-0.4364	-0.9589	-0.3974	-0.6123	-0.5924	0.67868	0.61564
GSM4750	-0.3625	-0.905	-0.4214	1.05742	-0.2832	-1.1028	-1.1257	1.662	-0.5526	-0.4652	-0.8413	-0.2692	-0.5496	-0.5307	0.44876	-0.1564	-0.8808	-0.3974	-0.2348	-0.5924	-0.6222	3.80473
GSM4750	1.3967	-0.905	-0.4915	-0.8041	-0.2832	-0.7064	-0.6065	-0.4735	0.40444	-0.4081	-0.1929	-0.2692	0.89163	2.26457	-0.0059	0.40645	-0.6483	-0.3974	-0.0693	-0.5924	2.92936	-0.8679
GSM4750	-0.2593	-0.4435	-0.3894	1.5265	-0.2832	-1.1028	-0.4923	0.55033	-0.5526	-0.5416	1.75571	-0.2692	-0.0846	-0.3909	0.19294	-0.4208	0.12831	3.2447	0.87494	-0.5924	0.67356	-0.2997
GSM4750	2.03352	-0.905	-1.0809	-1.1401	9.21365	0.03733	0.94576	-0.4735	-0.5526	1.57685	0.55831	-0.2692	-1.4959	-0.6666	1.1703	0.62735	-0.9589	1.01448	0.78107	0.46338	-0.0873	0.16628
GSM4750	-0.3625	-0.6096	0.08898	0.21717	1.06103	-1.1028	0.44949	3.14627	-0.5526	-0.5416	0.49155	-0.2692	-0.7594	0.44457	1.7863	-0.5036	0.59026	-0.3974	-0.2189	-0.3423	-0.669	0.00037
GSM4750	0.92488	-0.905	-1.0809	-0.0313	1.32643	-1.1028	0.43257	-0.4735	-0.5526	5.44694	2.23939	-0.2692	-1.5147	-0.8616	1.38196	0.45512	-0.9589	-0.3974	1.01439	-0.4101	1.37348	-0.1939
GSM4750	-0.3625	-0.2145	-0.8613	-0.2364	-0.2832	-1.1009	1.4331	2.13346	-0.5526	-0.5416	-0.5908	11.8723	-0.3108	-0.1838	0.52792	2.09663	-0.9589	-0.3974	-0.0034	1.35862	-0.669	-0.5606
GSM4750	-0.3625	-0.4047	-0.5857	-0.5552	2.60015	-0.7922	0.89562	0.40119	-0.5526	-0.5416	-0.8413	0.33559	0.14769	0.45974	-0.2086	0.29795	-0.9589	-0.3974	-0.6123	-0.5924	1.26378	1.94656
GSM4750	-0.3625	-0.905	-0.7604	0.9975	-0.2832	-0.9859	-1.0639	-0.4735	-0.5526	-0.3902	-0.1653	-0.2692	0.28998	-0.0961	0.36756	0.22014	-0.9589	-0.3974	-0.1452	-0.5924	0.94293	2.22748
GSM4750	-0.3625	-0.1814	1.54634	-1.404	-0.2832	-0.2705	1.35968	1.08061	-0.5526	-0.0483	-0.1254	0.01037	-0.8362	0.93861	0.19083	-0.5036	0.56723	-0.3974	-0.023	-0.1653	-0.669	0.85883
GSM4750	0.17187	-0.5132	-0.3388	-0.3329	0.5583	-1.0662	0.63473	-0.0177	-0.5526	-0.3343	0.81068	2.22266	-0.2634	-0.3638	0.30054	0.93319	-0.9589	0.02476	0.69624	-0.5924	0.31521	1.31778
GSM4750	-0.3625	-0.7241	1.34535	0.42248	-0.2832	-1.0471	0.24014	0.84344	-0.5526	-0.1557	-0.6305	-0.2692	-1.0576	1.97558	-0.3067	-0.5036	-0.1652	-0.3974	-0.1962	-0.5924	-0.0863	1.84651
GSM4750	-0.3625	-0.5416	0.60007	-0.0645	-0.2832	-1.1028	-0.8637	2.33232	-0.5526	-0.5416	0.71889	-0.2692	-0.2295	0.54017	0.07434	-0.5036	-0.2124	-0.3974	-0.1407	-0.5924	-0.4089	1.56619
GSM4750	-0.0225	-0.905	-0.0097	-1.2893	2.68973	-1.1028	0.2565	-0.4735	-0.5526	1.45394	-0.2842	4.38895	0.2769	-0.2908	0.8131	0.35671	-0.9589	-0.3974	0.01061	-0.5924	1.33231	0.9048
GSM4750	-0.3625	-0.4913	2.51991	-0.7835	-0.2832	-0.6061	0.30553	2.43574	-0.5526	0.49295	0.41243	-0.2692	-1.0371	1.53773	-0.0029	-0.2532	-0.9589	-0.3974	0.1573	-0.5924	-0.1024	0.15056
GSM4750	0.40269	-0.905	-1.057	-0.4205	-0.2832	-0.9657	0.40893	-0.4735	-0.5526	1.12778	-0.6247	-0.2692	-0.4956	2.15793	2.24488	1.64571	-0.9589	-0.3974	1.4966	0.45479	-0.669	-0.8801
GSM4750	-0.3625	-0.8795	0.49035	-0.0933	0.55698	-0.9871	2.03284	-0.4735	-0.5526	0.50856	-0.0383	-0.1793	-0.3025	1.41656	0.61784	0.34678	-0.9589	-0.3974	-0.6123	0.43089	-0.3291	0.31836
GSM4750	-0.087	-0.905	-0.3136	-0.8679	-0.2832	-0.3886	1.36111	-0.3726	-0.5526	-0.5416	1.82611	-0.2692	-1.2612	3.19011	1.3584	-0.5036	1.27248	-0.3974	-0.373	0.23461	-0.6479	-0.0512
GSM4750	-0.0561	-0.905	-1.0809	0.35286	1.51988	-1.1028	-0.0601	-0.4735	-0.5526	-0.5416	1.58819	-0.2692	1.51929	-0.3017	0.63266	0.65173	-0.9589	-0.3974	-0.6123	-0.5691	0.88217	-0.4416
GSM4750	-0.3625	-0.3804	0.12201	-0.6833	1.9834	-0.7092	1.38607	1.70938	-0.5526	0.04338	1.75823	-0.2692	-0.862	-0.0614	-0.6788	0.72035	-0.8465	-0.3974	-0.6123	0.4697	-0.669	1.54517
GSM4750	-0.3625	-0.7429	-0.2298	0.88316	-0.2832	-1.1028	0.77828	0.07735	-0.5526	-0.5416	-0.0751	-0.2692	0.59619	0.06075	-0.1379	0.24693	-0.9589	-0.3974	-0.0173	-0.5924	1.05754	0.8339
GSM4750	-0.3625	-0.905	-0.3878	0.43473	-0.2832	-1.1028	-0.6423	-0.2404	-0.5526	0.95949	-0.0837	-0.2692	0.83396	0.99869	-0.6938	0.8378	-0.9589	-0.3974	0.1534	0.78806	-0.669	0.49817
GSM4750	-0.3625	-0.7417	-1.0809	0.32794	-0.2832	-1.1028	0.38447	0.22282	-0.5526	1.2649	1.9969	-0.2692	-1.1355	0.44422	0.01387	-0.5036	0.67355	-0.3974	0.19841	1.51081	-0.669	1.67123
GSM4750	0.39188	-0.905	-1.0809	1.3898	-0.2832	-1.1028	0.75856	1.35202	-0.5526	-0.0519	1.82562	-0.2692	-0.7019	1.14994	0.72898	-0.5036	0.40088	-0.3974	-0.0965	1.25787	-0.669	-0.6536
GSM4750	-0.3625	-0.6002	0.09934	-0.2181	-0.2832	-0.7824	0.5228	1.13588	-0.5526	-0.5416	1.67512	-0.2692	-0.8681	0.62306	0.90736	0.45779	-0.9589	-0.3974	-0.119	0.04251	-0.669	1.11235
GSM4750	-0.1566	-0.905	0.26675	0.28472	-0.2832	-1.1028	-0.5145	0.3028	-0.5526	-0.312	-0.1057	-0.2692	1.23949	-0.3643	0.59999	-0.3739	-0.2263	-0.3974	0.59727	-0.5924	0.49285	0.0796
GSM4750	-0.3625	-0.6815	-0.9079	-0.5185	-0.2832	-1.1028	-0.0707	-0.0737	0.52086	0.58346	1.89591	1.47571	0.82386	-1.294	1.7067	0.21698	-0.9307	-0.3974	-0.0992	-0.5924	0.33808	0.30763
GSM4750	-0.3352	-0.5574	-0.6374	1.34435	-0.2832	-1.1028	0.86142	1.27159	-0.5526	-0.314	-0.4728	-0.0938	0.06122	0.93623	0.27912	-0.2086	-0.6861	-0.3974	0.36355	-0.1191	-0.669	0.36452
GSM4750	0.54037	-0.905	-1.0809	0.44078	-0.2832	-1.1028	0.98378	-0.4735	-0.5526	3.96896	-0.4216	-0.2692	0.45405	0.01652	0.76069	-0.4425	-0.6195	0.00938	0.82752	0.45052	-0.4074	-0.7399
GSM4750	0.55281	-0.905	-1.0145	1.83619	0.40239	-1.1028	0.09076	2.3599	-0.5526	-0.3148	0.85318	0.52607	-1.5147	0.49354	0.68625	-0.5036	1.78062	3.54163	0.09552	-0.2829	-0.669	0.11696
GSM4750	-0.3625	-0.8852	1.0669	0.42121	-0.2832	-1.1028	-0.3565	3.03877	-0.5526	-0.5416	-0.8413	0.02419	-0.179	0.50313	-0.6162	1.08253	-0.9589	-0.3974	0.08707	-0.5924	0.28311	1.05947
GSM4750	-0.3625	-0.706	1.59951	-1.2058	-0.2832	-1.1028	0.31851	1.75851	-0.5526	-0.5416	-0.5908	0.26987	-0.4145	1.96557	1.16141	0.24497	-0.9589	-0.3974	1.01649	0.32472	-0.669	0.06176
GSM4750	1.25787	-0.905	-1.0809	-0.7508	-0.2832	-0.4242	2.27866	0.46466	-0.5526	-0.5416	-0.8413	-0.2692	0.59969	0.51511	1.22469	-0.3813	-0.7035	-0.3974	-0.6123	0.23141	-0.669	0.57668
GSM4750	-0.3625	-0.5346	0.31357	0.03506	-0.2832	-1.1028	-0.2353	-0.4735	-0.5526	0.17018	4.59068	0.34359	-1.5147	-0.9494	0.30606	-0.5036	0.97314	3.49905	0.30301	-0.2433	-0.669	1.61886
GSM4750	-0.3625	-0.905	-0.376	1.29593	-0.2832	-1.1028	-0.7087	-0.4735	-0.5526	-0.5416	2.13033	-0.2692	0.15194	-0.72	0.09501	0.12849	-0.6016	-0.3974	0.05656	-0.5924	1.23071	1.19153
GSM4750	-0.3625	-0.6277	0.16541	0.81087	0.13935	-0.8916	1.03872	3.99155	-0.5526	-0.35	0.57879	-0.2692	-0.7295	1.35771	0.96491	-0.2021	-0.8259	-0.3974	-0.0858	-0.5924	-0.669	-0.7487
GSM4750	-0.3625	-0.905	1.76907	-1.5382	0.32299	0.37572	1.06988	3.4348	-0.5526	1.6699	-0.4808	-0.2692	-1.1402	2.36131	0.10279	-0.0655	-0.9589	-0.3974	-0.332	0.34177	-0.669	-0.6728
GSM4750	-0.3625	-0.905	0.19639	-0.7963	-0.2832	-0.5884	1.94755	-0.4735	-0.5526	-0.401	-0.1289	-0.2692	-1.1203	1.383	0.36958	-0.5036	-0.0682	-0.3974	0.3412	0.23581	0.06305	2.49886
GSM4750	-0.3625	-0.2145	0.34893	-0.6371	1.79868	-0.9698	-0.1916	0.36298	-0.5526	-0.5416	2.11997	-0.2692	-0.8163	1.37205	0.81888	-0.5036	0.64748	-0.3974	-0.3581	-0.0922	-0.669	0.56022
GSM4750	3.73526	-0.905	-1.0809	-0.3308	4.01708	-1.1028	-0.304	-0.2841	-0.5526	-0.2432	2.61946	-0.1536	-1.3905	-0.1738	0.26396	0.96288	-0.9589	0.43135	0.0773	-0.188	1.8036	0.85516
GSM4750	-0.3625	-0.6476	1.05956	-0.0451	-0.2832	-1.1028	-0.1726	1.27491	-0.5526	0.0845	-0.0613	0.63829	-0.4634	2.22664	0.08386	-0.5036	0.20299	-0.3974	0.15135	-0.5924	-0.2782	0.3396
GSM4750	-0.3625	-0.737	-0.9231	0.6214	-0.2832	-1.1028	-0.1222	-0.3915	-0.5526	0.66638	-0.121	2.33052	0.31201	-0.0135	0.98188	1.10109	-0.9589	-0.3974	-0.1899	-0.5924	0.72743	0.4806
GSM4750	1.8431	-0.905	-0.8194	0.62075	-0.2832	-0.1403	2.64588	-0.4735	-0.5526	-0.0003	-0.6109	-0.2692	-0.8727	-1.4215	0.80087	1.08966	-0.9589	-0.3974	0.48025	-0.5924	4.93148	-0.1736
GSM4750	-0.3625	-0.6074	0.69417	0.55319	1.19609	-1.1028	0.3018	2.1373	-0.5526	0.08879	-0.1752	0.53178	-0.8724	1.25047	-0.3478	-0.137	-0.2638	-0.3974	0.36713	-0.5924	0.43742	0.67002
GSM4750	-0.3625	-0.5475	-0.203	-1.5382	-0.2832	0.17706	1.1154	1.80431	-0.5526	-0.5416	-0.3481	-0.2692	0.70404	1.47297	0.07919	0.8312	-0.9589	-0.3974	0.49245	-0.3978	-0.669	-0.4779
GSM4750	0.18727	-0.905	0.65763	-0.1174	-0.2832	-1.1028	0.42096	1.24116	-0.5526	0.97125	-0.3732	2.3358	-1.0582	1.06768	2.08578	0.67563	-0.9589	-0.3974	0.27549	-0.061	-0.669	-0.1662
GSM4750	-0.3625	-0.1431	0.01664	0.76906	-0.2832	-1.0032	0.05663	1.74937	-0.5526	-0.5409	1.23987	-0.2692	-0.7922	1.44751	-0.0674	-0.5036	0.22119	-0.3974	-0.6083	-0.5924	-0.669	0.81846
GSM4750	-0.3625	-0.3989	4.48763	-1.5382	-0.2832	0.80356	-0.9529	1.8311	-0.5526	-0.5416	0.51919	-0.2692	-1.013	1.41114	-0.0614	-0.5036	-0.9589	-0.3974	-0.2686	0.20909	-0.669	-0.554
GSM4750	0.38259	-0.905	-0.7512	0.27525	-0.2832	-1.1028	-0.12	-0.4735	-0.5526	-0.41	-0.8413	-0.2692	0.51287	0.69058	1.04766	-0.0598	-0.7736	-0.3974	0.10958	-0.5924	1.45777	1.04456
GSM4750	2.17048	-0.905	-1.0809	0.01526	3.79623	-1.1028	4.52748	-0.4735	-0.5526	0.27549	0.9779	-0.2692	-0.1276	-0.8075	-0.8809	-0.2242	-0.7839	1.54526	-0.6123	2.077	-0.669	0.35774
GSM4750	-0.3625	-0.7191	0.83255	-0.5746	-0.2832	-0.6929	0.53271	1.02997	-0.5526	-0.5416	1.88001	0.67653	-1.0771	1.97519	0.86523	-0.5036	-0.2813	0.15191	0.18587	-0.2094	-0.5148	0.29672
GSM4750	-0.3625	-0.555	-0.3162	0.79911	-0.2832	-0.8619	-0.1909	-0.4735	-0.5526	0.12341	-0.8413	-0.2692	0.83155	-0.3377	-0.0844	0.23202	-0.9589	-0.3974	-0.1798	-0.5924	0.82104	1.2314
GSM4750	-0.3625	-0.5466	0.45556	-0.0584	-0.2832	-1.0635	1.16801	1.4959	-0.5526	2.36366	0.89508	-0.2692	-1.3185	1.0741	0.06869	-0.5036	-0.1328	-0.3974	0.31635	-0.2474	-0.1832	0.64764
GSM4750	-0.2057	-0.905	2.37779	-1.5382	-0.2832	0.99365	-0.1765	4.02157	-0.5526	-0.2575	-0.0301	1.1598	-1.0502	2.22623	-0.6158	-0.5036	-0.2554	-0.3974	-0.2784	0.22663	-0.669	-0.4302
GSM4750	0.92044	-0.905	-1.0605	1.08608	0.56024	-0.4328	0.03596	-0.4735	-0.5526	1.70382	0.44135	-0.2692	-0.0617	-1.4232	0.27198	0.69682	-0.9589	-0.3974	-0.3461	-0.5924	1.21807	0.67485
GSM4750	0.36432	-0.905	-1.0809	0.1279	-0.2832	-1.1028	0.41075	0.05893	-0.5526	-0.5416	0.68502	5.15011	0.37505	-0.2985	1.83083	-0.5036	2.21381	0.84496	0.13543	-0.0312	-0.669	-0.8334
GSM4750	-0.3625	-0.6664	0.43411	0.22911	-0.2832	-0.8412	1.11304	0.72521	-0.5526	-0.3196	-0.8413	-0.2692	-0.5547	0.24543	0.31447	-0.3854	-0.4096	-0.3974	0.09094	-0.5924	0.58426	1.97201
GSM4750	0.22304	-0.905	-0.282	-0.7527	1.047	-0.9584	1.42068	-0.4735	-0.1283	-0.2002	0.63356	-0.2692	-0.7377	1.52549	-0.1116	-0.5036	-0.5008	-0.3974	-0.6123	0.03608	0.04716	2.62912

GSM4750	-0.3625	-0.5723	2.65028	-1.5382	-0.2832	0.41625	-0.0535	5.23378	-0.5526	-0.5416	0.66839	-0.1832	-1.3974	1.53042	-0.5805	0.11908	-0.9589	-0.3974	-0.6123	0.84933	-0.669	-0.0439
GSM4750	-0.3625	-0.905	0.94753	-0.372	2.04747	-1.1028	0.38281	-0.4735	-0.5526	-0.5416	0.25109	-0.2692	-0.0294	2.10319	-0.1372	0.14069	-0.9589	-0.3974	0.34098	0.42359	0.42914	0.31935
GSM4750	0.10449	-0.905	-1.0809	1.23207	-0.2832	-1.1028	-0.5439	0.05223	-0.5526	2.53305	-0.2781	-0.2692	0.09812	-0.3932	0.43001	-0.5036	0.83626	-0.3974	-0.2003	-0.5924	0.19558	0.78362
GSM4750	-0.3625	-0.6779	-0.7426	-0.2403	-0.2832	-1.0446	0.44322	-0.2068	-0.5526	-0.5416	1.83064	3.09647	-0.7799	1.85599	1.16559	-0.5036	-0.485	-0.3974	0.11437	-0.5924	2.7025	0.39375
GSM4750	2.43668	-0.905	-1.0809	0.12556	2.26961	-1.101	-0.4506	-0.4735	-0.5526	0.02324	-0.2193	-0.2692	-0.2764	-0.954	0.01719	0.33005	-0.9589	-0.3974	-0.6123	-0.5924	2.33497	2.57909
GSM4750	-0.3625	-0.2317	-1.0809	0.20149	-0.2832	-1.1028	0.28212	-0.4735	-0.5526	0.21799	-0.064	0.88138	0.32406	-0.341	0.71252	1.12226	-0.9589	0.00915	-0.1019	-0.5924	0.67021	1.05389
GSM4750	-0.3625	-0.8427	-0.6497	1.1554	-0.2832	-0.6708	-0.144	0.39726	-0.5526	-0.5416	-0.0044	-0.2692	-0.0768	0.38702	-0.3212	1.30241	-0.9589	-0.3974	-0.1724	-0.4003	-0.3985	1.48227
GSM4750	-0.1843	-0.8868	-0.6385	-0.2148	2.01166	-1.1028	0.87275	-0.4735	-0.5526	-0.5416	-0.4537	-0.2692	0.86467	1.12298	1.14045	-0.5036	-0.2411	-0.3974	-0.6123	-0.2523	-0.2123	0.53124
GSM4750	0.18681	-0.8039	-0.852	-0.5495	-0.2832	-0.7853	3.77484	-0.4735	-0.5526	-0.3582	-0.2743	-0.2692	-0.1694	0.09921	1.31783	-0.3388	-0.651	-0.3974	-0.1618	0.3569	-0.5033	1.45704
GSM4750	-0.3625	-0.905	0.02626	0.12384	1.39791	-1.1028	-0.1989	0.72993	-0.5526	-0.5416	-0.6695	-0.2692	0.6662	0.31918	-0.2767	-0.5036	-0.391	-0.3974	-0.6123	-0.5661	0.75087	1.8746
GSM4750	0.76182	-0.905	0.07828	-0.1965	-0.2832	-0.9633	0.10242	0.69738	-0.5526	-0.2641	-0.2557	-0.2692	1.67976	-0.483	0.66226	-0.2132	-0.5277	-0.3974	0.42234	-0.5924	-0.669	-0.4018
GSM4750	0.05387	-0.905	0.43046	0.25322	-0.2832	-0.0456	0.85549	0.20614	-0.5526	0.84903	0.77699	-0.2692	-1.5147	0.83288	2.95234	0.19792	-0.9589	-0.3974	-0.061	-0.4129	0.17618	-0.8464
GSM4750	-0.0497	-0.905	0.15344	0.6162	-0.2832	-1.1028	-0.9835	2.70056	-0.5526	0.643	-0.5477	2.10592	-0.2788	1.60336	-0.206	-0.12	0.23053	-0.3974	-0.2909	-0.3772	-0.669	0.42139
GSM4750	-0.3625	-0.6235	1.09238	-0.1222	-0.2832	-0.8585	-0.4933	2.33845	-0.3984	-0.5416	-0.8413	0.31717	-0.069	0.4591	-0.152	1.19116	-0.9589	-0.3974	0.10925	-0.5924	-0.5464	0.98126
GSM4750	0.17261	-0.905	-1.0809	0.19783	-0.2832	-1.1028	-0.0516	-0.4735	-0.5526	0.16957	1.74521	0.05979	0.34545	-1.2358	0.17022	4.28748	-0.9589	-0.3936	0.21342	-0.5924	1.01525	-0.253
GSM4750	1.12942	-0.905	-1.0809	0.48935	-0.2832	-0.6674	1.91554	-0.4735	0.24749	-0.2941	0.61882	-0.2692	-0.6371	0.97154	1.04684	0.05982	-0.7452	-0.3974	-0.263	0.25999	0.47707	0.22709
GSM4750	-0.3625	-0.6329	0.25817	0.34391	-0.2832	-1.1028	-0.4335	0.66792	-0.5526	-0.5416	-0.8413	-0.2692	0.046	0.01055	0.25578	2.20031	-0.9589	-0.3974	-0.0512	-0.5924	-0.0542	1.225
GSM4750	-0.3625	-0.6433	1.43721	-1.5382	2.31347	-0.7101	0.64893	3.06669	-0.5526	-0.5416	-0.5103	-0.2692	-0.1478	1.49026	0.23809	-0.5036	-0.5343	-0.3974	0.02805	-0.2816	-0.6419	0.47219
GSM4750	0.19292	-0.905	-0.6052	0.49086	-0.2832	-0.8116	-0.0888	-0.0941	-0.5526	0.88301	0.64507	-0.2672	0.18681	-0.6482	1.13236	-0.1106	0.03576	-0.3974	0.09405	-0.5924	0.48979	0.38277
GSM4750	-0.3625	-0.7619	0.31085	0.54694	-0.2832	-1.0769	-1.1003	3.40416	-0.5526	-0.5416	1.08877	1.47061	0.14567	-1.0883	0.62659	-0.5036	0.49392	-0.3974	-0.3132	0.18611	-0.669	0.39403
GSM4750	-0.3625	-0.892	0.88314	0.31976	-0.2832	-1.0129	-1.1257	1.97408	-0.5526	-0.5416	0.97806	1.23908	-0.0955	-0.6353	0.78458	-0.5036	0.4651	-0.3974	-0.1664	-0.2303	-0.669	0.90002
GSM4750	-0.3625	-0.905	1.97475	-1.5382	2.59621	-0.1404	0.51975	3.71122	-0.5526	-0.4341	-0.2829	-0.2692	-0.5977	1.51689	0.26767	-0.3659	-0.7991	-0.3974	0.06462	-0.1856	-0.669	0.01843
GSM4750	-0.2588	-0.8919	0.15596	0.71115	-0.2832	-1.0022	0.34843	-0.4051	-0.5526	-0.0097	1.54212	-0.129	-0.2785	-0.9901	1.58958	-0.5036	0.86539	-0.3974	0.3061	-0.15	-0.669	0.47315
GSM4750	0.82744	-0.905	-1.0809	0.5859	0.29166	-1.0891	-0.6992	-0.4735	-0.5526	-0.3468	1.08631	-0.2692	0.15003	-0.3728	0.57146	-0.1922	0.63093	-0.3974	-0.6123	-0.5924	2.3866	0.97077
GSM4750	-0.3625	-0.2521	0.73334	0.38092	0.51982	-1.1028	0.30291	3.45847	-0.5526	-0.5416	-0.1122	0.2826	-0.8495	2.13928	-0.7098	-0.5036	-0.3273	-0.3974	-0.1875	-0.5924	-0.669	0.8164
GSM4750	-0.3625	-0.014	-0.0508	0.13957	-0.2832	-1.1028	-0.1106	0.80951	-0.5526	-0.2969	-0.8413	-0.2692	-0.1664	0.1199	-0.762	2.18638	-0.9589	0.00164	-0.5242	-0.5924	-0.5802	2.11725
GSM4750	0.44401	-0.905	-0.0425	0.08291	-0.2832	-0.3129	0.73341	2.36072	-0.5526	-0.5416	-0.3973	-0.2692	0.83298	0.9829	0.42469	-0.2662	-0.9589	-0.3974	-0.6123	-0.5924	-0.669	-0.6227
GSM4750	-0.3625	-0.4686	-0.444	0.15153	0.4142	-1.1028	0.73485	-0.1529	-0.5526	-0.3749	-0.6004	0.99974	0.43354	-0.6594	-0.2709	1.06013	-0.9589	-0.0372	0.47988	-0.5924	0.71247	1.62777
GSM4750	-0.3625	-0.3	1.10636	0.99906	0.56248	-1.1028	1.87852	-0.4735	-0.5526	0.02479	-0.1656	-0.2692	-0.5587	0.39349	-0.6985	-0.2583	0.23179	1.57022	-0.0721	-0.5924	0.35883	0.52933
GSM4750	-0.3625	-0.7993	0.65134	-0.3205	-0.2832	-0.7041	-0.8955	3.47588	-0.5526	0.37362	0.4402	-0.2692	-0.8863	1.4149	0.30405	-0.3497	-0.9545	-0.3974	-0.1123	-0.2123	-0.669	1.28145
GSM4750	-0.3625	-0.563	-0.3372	-1.0669	2.95266	-1.1028	0.85562	0.77465	-0.5526	0.59778	-0.7392	-0.2692	-0.5371	0.86253	1.00096	0.11999	-0.9589	-0.3974	0.89627	-0.1316	-0.4885	1.64523
GSM4750	-0.3625	-0.8476	-0.3516	0.11723	-0.2832	0.07632	1.48485	0.81902	-0.5526	0.60833	-0.3029	-0.2692	-1.0823	0.83024	0.94645	0.93149	-0.9589	-0.3974	0.37491	-0.366	-0.669	0.61075
GSM4750	1.21761	-0.905	-0.0043	-0.2509	-0.2832	-0.1819	0.30659	3.23723	-0.5526	-0.5416	0.26865	-0.2692	0.06175	1.37795	-1.251	0.11297	-0.9589	-0.3974	-0.6123	-0.5924	0.77236	0.18181
GSM4750	-0.3625	-0.0918	-0.7943	0.5785	-0.2832	-1.1028	-0.5188	-0.2081	-0.5526	-0.5416	2.74742	-0.2692	-0.9285	-0.3882	1.17594	-0.5036	0.26015	-0.3974	-0.1245	0.20682	-0.2481	1.62249
GSM4750	0.24672	-0.905	-0.4532	0.54293	-0.2832	-1.0978	-0.5736	0.33461	-0.5526	1.082	-0.8413	-0.2692	0.55091	1.00789	0.70292	1.56029	-0.9589	-0.3974	-0.3606	-0.5924	1.07595	-0.6905
GSM4750	-0.3625	-0.7712	-0.2383	-0.0122	-0.0398	-1.1028	0.58381	-0.4149	-0.5526	-0.4059	-0.4465	-0.2692	0.63338	0.04408	0.3853	1.00688	-0.9589	0.82338	0.84815	-0.5924	0.6633	0.70346
GSM4750	0.95079	-0.905	-1.0809	0.17317	2.20498	-1.1028	2.3057	-0.4735	-0.5526	0.21326	0.19095	-0.2692	0.01829	-1.0343	-0.893	1.87795	-0.9589	-0.3974	-0.4135	-0.5924	2.79494	1.18088
GSM4750	1.50324	-0.905	0.06389	-1.5235	-0.2832	-0.9197	3.34092	2.10935	-0.5526	0.08085	0.60622	-0.2692	-0.4457	0.06354	0.43918	0.76584	-0.9589	-0.3974	0.39888	-0.5924	-0.669	0.64514
GSM4750	-0.3625	-0.8308	-0.5966	-0.221	3.80685	-1.1028	0.87331	1.29329	-0.5526	-0.5416	-0.8413	-0.2692	-0.0197	1.78173	0.49788	-0.5036	-0.2554	-0.3974	-0.2416	-0.5924	1.56853	0.57881
GSM4750	-0.3625	0.72995	-0.5213	1.5466	-0.2832	-1.1028	-0.0689	2.90567	-0.5526	0.15375	-0.2198	4.44682	-1.1648	-0.9871	0.48654	0.21908	-0.9589	-0.3974	0.09195	-0.5924	-0.3605	0.9055
GSM4750	1.72146	-0.905	-0.5621	-1.4268	2.58776	-1.1028	0.72774	0.69226	-0.354	-0.5416	-0.3584	-0.2692	2.5842	-0.4955	-0.4026	0.3728	-0.9589	-0.3974	-0.0314	-0.5924	0.05364	-0.4762
GSM4750	-0.3625	-0.7574	3.11852	-1.2363	-0.2832	-0.4595	-0.2673	1.89371	-0.5526	-0.3107	0.15995	0.14585	-1.0896	2.47021	-0.147	-0.2465	-0.9589	-0.3974	-0.373	-0.5924	1.00762	0.49027
GSM4750	-0.0862	-0.905	-1.0809	-1.5382	-0.0285	0.02727	3.2727	1.45959	0.61314	-0.5416	-0.0181	0.71031	1.28636	-0.4767	0.89914	1.05613	-0.9589	0.00853	-0.6123	-0.5924	-0.669	-0.6161
GSM4750	-0.3625	-0.5294	1.48879	-1.2423	-0.2832	-0.1897	-0.0122	2.99892	-0.5526	-0.5416	0.89247	-0.2692	-0.5443	0.64255	-0.9564	-0.5036	-0.241	-0.3974	-0.3099	-0.5924	-0.669	1.81167
GSM4750	-0.3625	-0.4236	0.32572	-0.0486	-0.2832	-0.4944	0.36011	1.37638	-0.5526	-0.523	0.1551	-0.2692	-0.1306	0.87301	-0.3912	-0.5036	0.91722	-0.3974	-0.5624	-0.5924	-0.669	0.97352
GSM4750	5.53075	-0.905	-0.1018	-0.7969	9.88756	-1.1028	0.35246	-0.4735	1.45737	0.68601	0.14868	1.65951	-1.3384	-0.8417	-1.7338	0.13882	-0.9589	0.41882	-0.6123	-0.5924	3.38536	1.18104
GSM4750	-0.2148	-0.3792	-0.1847	0.53731	7.1362	-1.1028	0.98405	-0.4597	-0.5526	-0.4166	-0.0329	-0.2692	-0.2185	2.8778	-0.7446	-0.5036	-0.3567	-0.3974	-0.6123	-0.5924	2.99187	-0.9399
GSM4750	0.54214	-0.905	0.24954	0.43217	-0.2832	0.11699	0.83487	1.4896	-0.5526	0.59394	-0.8413	-0.2692	-1.2347	2.59956	0.46776	0.54145	-0.9589	-0.3974	0.65237	-0.5924	-0.5526	-0.6359
GSM4750	-0.3625	-0.6478	-1.0809	-0.8749	0.33997	-1.1028	1.46483	0.34774	0.44014	0.53539	0.04904	-0.2692	-0.4276	0.84609	0.37012	-0.5036	-0.0956	-0.3974	-0.6123	-0.2929	2.06632	2.05721
GSM4750	-0.3625	-0.6543	0.29789	0.14033	-0.2832	-0.8039	0.36651	2.25766	-0.5526	0.51682	-0.8413	0.5669	0.5736	0.76039	-0.2938	-0.4046	-0.3641	-0.3974	-0.4143	-0.5924	-0.669	0.36091
GSM4750	1.97631	-0.905	0.07175	0.15543	0.88877	-0.6732	-0.3351	-0.0776	-0.5526	0.81334	0.03404	-0.2692	-0.4932	0.53962	-0.9553	3.09816	-0.9589	-0.3974	0.15071	-0.1417	0.05635	-0.1257
GSM4750	-0.3625	-0.8805	1.37197	-1.0014	2.88607	-0.5794	-0.2494	0.36931	-0.5526	0.77277	-0.024	-0.2692	-1.1416	1.02282	0.88709	-0.5036	0.9827	-0.3974	0.01634	-0.5924	0.29236	0.9481
GSM4750	-0.3625	0.05187	0.72486	1.53298	5.08914	-1.1028	-1.1257	0.08879	-0.5526	0.02944	-0.4302	-0.2692	-0.936	-0.2158	-0.3419	0.10592	-0.9589	-0.3974	0.10817	-0.5924	1.60442	1.09378
GSM4750	0.29803	-0.905	-1.0809	-0.6045	5.4034	-1.1028	-0.1363	-0.4735	-0.5526	0.86065	-0.8413	-0.2692	0.53362	-1.1765	-0.1135	4.45309	-0.9589	-0.3974	0.37532	-0.5924	2.48254	-0.2513
GSM4750	-0.3625	-0.5918	-1.0809	0.98965	-0.2832	-1.1028	0.8816	1.57955	-0.5526	-0.0187	-0.3976	-0.2692	0.12075	0.70118	-0.1656	-0.5036	0.10198	-0.3974	-0.6123	-0.3944	-0.5597	1.39812
GSM4750	0.75983	-0.905	-1.0809	-0.5671	-0.2832	-1.1028	1.19227	0.45579	-0.5526	-0.5416	-0.2709	-0.2692	1.0946	-0.6515	2.53863	-0.5036	-0.4357	-0.3974	-0.3563	-0.4075	-0.6226	0.49616
GSM4750	-0.225	-0.905	-0.2363	0.2108	-0.2832	-0.7223	1.33236	0.13157	-0.5526	-0.0056	0.30413	-0.2692	0.06143	0.99069	-0.312	-0.2077	-0.291	-0.3974	0.33168	-0.5924	1.22724	0.55037
GSM4750	-0.3625	-0.4121	0.28241	1.29076	-0.2832	-1.1028	-0.0855	3.53904	-0.5526	-0.2047	0.69348	1.58365	-0.9826	0.61956	-0.2975	-0.5036	0.13292	-0.3974	0.40207	-0.5924	-0.669	0.51636
GSM4750	-0.3625	-0.5726	3.62154	-1.4934	0.83679	-0.7731	-0.4307	2.94719	-0.5526	-0.4413	-0.1743	-0.2692	-0.5025	1.78602	0.06779	-0.5036	-0.7599	-0.3974	-0.1336	-0.5924	-0.669	0.15779
GSM4750	-0.3625	-0.905	-1.0809	0.82421	6.97827	-0.0639	-1.1257	-0.4735	-0.5526	0.59358	0.1466	-0.2692	0.92332	-1.0299	-0.1237	0.56449	-0.9589	-0.3974	0.2933	-0.5924	1.04861	-0.3885
GSM4750	-0.3625	-0.5479	0.88895	-0.1125	-0.2832	-0.4846	1.3385	1.39175	-0.5526	-0.4352	-0.1407	-0.2692	-0.8892	2.47183	-0.3097	-0.5036	0.21875	-0.3974	-0.3059	0.15533	-0.669	0.41289
GSM4750	0.08802	-0.905	0.31492	1.54724	-0.2832	-1.1028	-0.4866	0.85293	-0.5526	-0.5416	-0.3451	-0.2692	-0.8069	0.77091	2.96408	-0.0663	-0.4619	0.67323	0.13396	0.96178	-0.669	-0.9891
GSM4750	2.57959	-0.905	-1.0809	0.10147	1.78919	-1.0516	-0.7586	-0.4735	-0.5526	0.01152	0.73653	-0.2692	-0.4625	-0.1306	1.26365	0.49659	-0.9589	-0.3974	-0.5442	-0.5924	2.14085	0.88995
GSM4750	-0.3625	-0.4217	-0.409	-0.2274	1.65318	-1.1028	0.23211	1.04562	-0.5526	-0.5416	-0.215	-0.2692	0.11663	0.78333	0.00103	0.2697	-0.9589	-0.3974	0.05256	0.31401	-0.669	1.46233
GSM4750	0.37457	-0.905	-0.6798	0.76916	1.65814	-1.1028	-0.6317	2.14626	-0.5526	-0.5013	-0.5993	3.77802	0.23908	-1.1572	0.05334	2.97147	-0.9589	-0.3974	0.53058	-0.5924	-0.669	0.21301
GSM4750	-0.3625	-0.6533	-0.6562	-0.5337	-0.2832	-1.1028	0.59652	0.77879	-0.5526	-0.5172	0.39868	-0.2692	-0.7798	1.38431	1.01345	-0.5036	0.38549	-0.3974	0.39932	1.42083	-0.669	1.50049
GSM4750	-0.064	-0.905	-0.1399	-1.3189	-0.2832	-0.5001	0.16003	0.90651	0.51137	-0.5416	-0.6718	-0.2692	1.11988	0.57622	1.81228	-0.2699	-0.8672	-0.3974	-0.4548	-0.5924	0.94258	-0.095
GSM4750	-0.3625	-0.8432	-0.1381	-1.5382	-0.2832	-0.2263	2.3473	1.17903	-0.5526	-0.5416	0.00983	-0.2692	0.35578	1.25026	0.09895	-0.5036	0.01646	-0.3974	-0.131	0.53911	-0.669	0.59131
GSM4750	-0.3625	0.07462	-0.2324	0.3159	-0.2832	-1.1028	1.69828	0.16773	-0.5526	-0.3736	-0.8413	-0.2692	-0.3296	1.31196	-0.0131	-0.5036	0.43288	-0.3974	0.08186	-0.5924	-0.0276	1.05599
GSM4750	-0.3625	-0.6696	0.17631	-0.5636	5.0803	-1.1028	-0.113	0.52251	-0.5526	1.93716	-0.67	-0.2692	-0.6798	0.92734	-0.7979	0.42224	-0.9589	-0.3974	0.54415	-0.5924	0.49937	1.80183
GSM4750	0.55564	-0.286	1.47596	0.09582	1.9168	-1.1028	-0.0403	5.33365	-0.5526	-0.3445	-0.2499	1.18839	-0.8103	0.18556	-0.4663	-0.5036	-0.3368	-0.102	0.01532	-0.5924	0.35889	-0.108
GSM4750	-0.3625	-0.5987	-0.2814	-0.2959	-0.2832	-0.9787	0.62423	1.80139	-0.5526	-0.5416	0.28591	0.479	-0.1444	1.37277	1.40339	-0.5019	-0.6439	-0.3974	-0.0429	1.35067	-0.669	-0.0974
GSM4750	-0.3625	-0.7215	0.20347	0.20415	-0.2832	-1.0015	-0.4725	0.49308	-0.5526	0.32615	0.65477	6.43004	-1.1278	1.32348	1.63375	-0.5036	-0.3377	-0.3974	0.14273	-0.5924	0.01479	0.51516
GSM4750	-0.3625	-0.905	-1.0036	-0.1183	-0.2832	-1.1028	0.12632	-0.1278	-0.5526	1.82193	-0.8413	0.91505	0.49991	-1.1557	-0.4219	5.27994	-0.9589	-0.3974	0.81531	-0.5924	0.09558	0.1286
GSM4750	-0.3625	-0.1167	1.10295	-0.7952	3.49274	-1.1028	-0.0414	1.87505	-0.5526	-0.4812	-0.8413	-0.2692	-0.6872	2.35716	-0.1699	-0.5036	1.07048	-0.3974	-0.2501	-0.5924	0.26637	0.34798
GSM4750	-0.3625	-0.3886	0.59635	0.80808	-0.2832	-1.1028	-0.5184	1.35597	-0.5526	-0.5416	-0.395	-0.001	-0.0689	1.02963	0.44107	-0.5036	0.06061	-0.3974	0.13885	-0.5924	-0.0734	0.27539
GSM4750	-0.3625	-0.905	-0.4528	-0.2006	-0.2832	0.23721	2.29345	-0.4735	0.50035	-0.3492	0.39555	-0.2692	-1.2063	2.50756	1.86322	0.08688	-0.9589	-0.3974	-0.173	1.52615	-0.5932	-0.7538
GSM4750	-0.3625	-0.905	0.23982	-0.7765	-0.2832	-0.2278	-0.2789	-0.4735	-0.5526	-0.4053	-0.8413	-0.2692	1.06398	0.44891	-0.6665	1.3927	-0.9589	-0.3974	0.44368	-0.5924	1.10348	0.7721
GSM4750	0.38738	-0.905	-0.3916	0.40131	0.96905	-1.1028	1.10053	1.1733	-0.5526	-0.5416	0.15755	-0.2692	-0.3868	2.60588	0.77194	-0.5036	0.25148	-0.3974	-0.3704	-0.5924	-0.669	-0.1527
GSM4750	-0.3625	-0.2188	0.25642	0.55685	-0.2832	-1.1028	1.46527	3.50667	-0.5526	-0.5416	1.93064	2.91618	-1.1078	0.46702	0.02316	0.67344	-0.9589	-0.3974	-0.0932	-0.5924	-0.669	-0.1257
GSM4750	-0.3625	-0.4692	1.27036	-0.351	1.09134	-1.1028	-0.7333	2.5618	-0.5526	-0.5416	0.895	-0.2692	-1.0472	0.70361	0.10938	-0.5036	0.26541	-0.3974	0.24015	-0.5924	-0.3487	1.52418
GSM4750	2.38671	-0.905	-1.0809	1.0823	-0.2832	-0.7839	-1.1257	-0.4735	-0.5526	-0.2015	-0.817	-0.2692	1.44372	-1.3957	0.70248	0.26367	-0.9589	-0.3974	-0.6123	-0.5924	2.64646	-0.2454
GSM4750	-0.3377	-0.8419	-0.1462	0.35073	0.21107	-1.1028	0.66168	0.71911	-0.5526	-0.5416	0.65868	-0.2692	0.07765	1.20711	0.82054	0.18936	-0.5748	-0.0128	-0.0852	-0.5924	-0.3886	0.16494
GSM4750	-0.3625	-0.905	1.8447	-0.9518	0.7925	-0.2405	0.64237	1.22888	-0.5526	1.06192	-0.4216	-0.2692	-1.1253	1.31966	-0.1679	-0.5036	1.57142	-0.3974	-0.0315	1.04862	-0.669	0.13375
GSM4750	-0.3625	-0.4114	0.08193	0.20487	-0.2832	-1.1028	-0.1501	1.03983	-0.5526	-0.3865	0.11316	-0.2692	0.55389	0.06538	0.87189	-0.3622	-0.6511	-0.3974	0.3432	-0.3687	-0.6139	0.49488

Table-S3: Relative Immune Scores of 792 LUSC patients

Tag	StromalScore	ImmuneScore	ESTIMATEScore
TCGA-34-5234	1.025465691	1.076242958	3828.502618
TCGA-96-7544	0.598534772	-0.003268653	2252.850872
TCGA-85-A513	0.495060209	1.222116877	3386.359923
TCGA-85-8584	-0.380446726	0.547142659	1723.311156
TCGA-85-A511	0.327116712	-0.029414645	1923.88306
TCGA-66-2789	0.244808076	-0.358094693	1497.251174
TCGA-22-0940	0.111828422	0.812043072	2541.593667
TCGA-96-A4JL	0.1076451	1.162917858	2894.503436
TCGA-77-8136	0.428599883	0.730273962	2811.104918
TCGA-63-5131	-0.883969511	-0.806407856	-216.921083
TCGA-90-7964	-0.227792951	-0.309654644	1020.201919
TCGA-85-A5B5	-0.891543263	-1.250761623	-678.1891858
TCGA-37-3789	-0.416986635	0.21692942	1346.096987
TCGA-O2-A5IB	-1.570660881	-2.08621223	-2286.025109
TCGA-58-8390	-0.023348336	-0.596680061	955.4237101
TCGA-66-2777	-0.126807561	0.243140757	1696.028618
TCGA-77-8140	-1.323314124	-1.199890821	-1107.282037
TCGA-98-A538	-0.489002918	-0.554821891	479.4043935
TCGA-66-2785	-0.132727824	0.911940642	2370.994732
TCGA-43-5670	-1.272947118	-1.714581908	-1575.691329
TCGA-22-5472	-0.434718161	-0.196264599	905.2687715
TCGA-60-2704	1.105030081	0.851411568	3688.005433
TCGA-77-6843	-1.438329101	-1.744074934	-1789.960206
TCGA-63-A5MG	-0.304797375	-0.015659168	1234.034284
TCGA-66-2734	0.162883304	0.247694386	2023.345517
TCGA-43-2581	0.473038655	0.868824681	3001.797937
TCGA-52-7811	-0.1761049	-0.288678156	1099.152123
TCGA-85-8287	0.712496688	0.688580024	3084.838054
TCGA-85-8355	0.115381596	1.435190236	3180.588139
TCGA-94-7033	-1.614055326	-0.99227335	-1219.549575
TCGA-60-2697	0.641146165	1.445288343	3776.509435
TCGA-77-8145	0.24544599	-0.902183456	943.4918304
TCGA-85-7844	0.195454911	-0.395851406	1403.801328
TCGA-39-5040	-0.931924102	-1.059487971	-528.244847
TCGA-66-2795	0.374061953	0.096178496	2104.163139
TCGA-33-4586	-0.760956839	-1.162556012	-442.8450363
TCGA-NC-A5HH	-1.278844409	-1.232179538	-1090.653576
TCGA-22-1002	0.270774667	0.091735135	1984.586952
TCGA-60-2695	-0.516915499	-0.357723574	649.172482
TCGA-77-A5G8	-0.562302211	-0.522301639	430.8996416
TCGA-63-A5MP	-0.443857534	0.382288318	1484.680366
TCGA-22-0944	-1.131422137	-1.285530883	-980.8144141
TCGA-21-1081	-0.306832079	-1.275843898	-52.46096322
TCGA-O2-A52V	-0.437840791	-0.274951442	821.6024072
TCGA-33-AASB	-1.995017502	-1.724641281	-2390.23124
TCGA-63-5128	-0.760629087	-1.419406739	-704.2313646
TCGA-96-8170	-0.367921953	-0.194050845	981.926691
TCGA-63-A5MW	-0.030886012	-1.303295241	226.93047
TCGA-37-3792	0.346164296	1.422398774	3424.613287
TCGA-56-A4BX	-1.9824549	-2.156521287	-2816.358414
TCGA-56-8083	-1.043036121	-2.128409309	-1741.324782
TCGA-98-A53H	0.768494334	0.983950962	3448.218418
TCGA-94-A4VJ	-0.52197128	-0.567111322	430.1581923
TCGA-43-7658	-1.392097238	-0.120770574	-84.1874108
TCGA-NC-A5HN	-1.475708927	-1.365560636	-1445.859974
TCGA-63-7021	-0.073979595	0.147910483	1657.824564

TCGA-22-5471	-0.379255479	0.366317616	1540.362886
TCGA-77-8133	-0.567055656	0.439610823	1405.870555
TCGA-37-A5EM	0.450198965	0.175942689	2270.25554
TCGA-85-8071	-1.227054346	-1.10876815	-907.2004027
TCGA-NC-A5HP	-0.136495767	-1.277707251	135.3717127
TCGA-85-A4PA	-0.106647877	2.089261427	3599.828148
TCGA-33-4582	-1.262863972	-1.349883231	-1192.802996
TCGA-43-2578	-0.648921564	-0.496443377	360.7690491
TCGA-34-A5IX	-1.093832782	-0.598315952	-238.6180761
TCGA-56-8504	-0.288577094	-0.276462679	986.3218783
TCGA-18-5595	-1.244101872	-1.663562586	-1491.568868
TCGA-21-5784	-1.32E-01	0.377518799	1826.966417
TCGA-90-A4ED	-0.332468985	-0.68571269	520.3738181
TCGA-MF-A522	-1.485677393	-1.373119074	-1464.666148
TCGA-46-3768	-0.795944832	-0.823229675	-136.0162898
TCGA-77-7335	1.391190939	1.170692668	4332.122806
TCGA-56-A4BY	-0.88547875	-0.863088595	-276.3643698
TCGA-85-8070	-1.901898515	-1.943437906	-2509.480532
TCGA-56-7579	-0.551940557	-0.804123671	155.2419676
TCGA-NC-A5HG	-1.581851315	-1.472608101	-1673.178133
TCGA-56-8305	-0.531533546	-1.037616156	-59.97487197
TCGA-60-2703	-0.01706485	-1.053622711	496.7616169
TCGA-39-5036	-1.673381602	-0.829138379	-1119.383449
TCGA-33-6737	-0.192945031	-0.19990947	1170.856878
TCGA-34-2608	0.854106886	0.647973673	3201.191521
TCGA-39-5024	-1.070244369	-0.429152103	-39.9523087
TCGA-77-7142	-0.43205067	-0.665429758	430.1232356
TCGA-85-8350	-0.139633451	-0.296440629	1131.865797
TCGA-O2-A52Q	-0.017249573	0.936456627	2524.60561
TCGA-68-8250	-0.453836373	-0.082930525	999.4701437
TCGA-70-6723	-1.590652849	-1.674294129	-1888.516017
TCGA-68-8251	0.257681447	-0.013211733	1863.05364
TCGA-85-7697	-0.412143576	-0.337053792	786.9383709
TCGA-66-2756	-0.723149357	-0.932961337	-166.7573158
TCGA-85-6561	0.980539883	0.662260801	3356.58048
TCGA-94-7943	-0.866914366	-0.653297489	-41.89227222
TCGA-22-1011	-0.065339455	0.623979876	2152.601235
TCGA-85-8664	-1.820663966	-1.480683824	-1947.412887
TCGA-63-A5MH	-0.906164862	-1.34464821	-790.1535952
TCGA-94-8490	-1.431298492	-1.677315784	-1714.096155
TCGA-85-8481	0.183232769	-0.150626776	1640.090996
TCGA-60-2716	-1.554604552	-1.815371246	-1992.131944
TCGA-O2-A52S	-1.083650533	-0.317709547	58.68390309
TCGA-51-4080	-1.242493257	-1.297735261	-1116.969833
TCGA-22-5485	-0.768178252	-0.844256454	-126.5160465
TCGA-85-6175	1.249568546	0.623461846	3616.702739
TCGA-85-8276	0.560406752	0.473797987	2696.55042
TCGA-77-8008	1.135210501	0.3714328	3232.485643
TCGA-39-5028	0.13923723	0.052031595	1797.610986
TCGA-34-5928	0.739778024	-0.280374895	2127.783336
TCGA-43-6773	0.838944505	1.033994407	3577.688632
TCGA-63-A5MI	-1.17441083	-1.545608611	-1293.737967
TCGA-63-A5ML	-0.551041168	-0.707303248	254.911507
TCGA-39-5030	0.705882363	1.352794155	3754.357827
TCGA-51-4079	-0.323237184	-0.346255096	876.5911865
TCGA-22-5491	-1.410210588	-0.895683633	-894.0615115
TCGA-66-2744	0.339818815	1.242565493	3234.280809
TCGA-33-4547	-0.236255275	-0.408877137	909.6603933

TCGA-52-7810	-0.860790244	-1.364342798	-759.6827748
TCGA-77-7139	-0.769211515	-0.923878984	-208.8086784
TCGA-66-2800	-0.09478775	0.145304896	1631.991796
TCGA-33-4566	-0.345401202	0.416629499	1629.343791
TCGA-90-7766	-0.984018553	0.212506831	709.9932593
TCGA-60-2707	0.224475642	-0.184582114	1651.426769
TCGA-56-8628	0.330686389	0.57522069	2544.030881
TCGA-85-8666	0.19535043	-0.081605873	1723.92624
TCGA-60-2721	0.045795669	0.499879914	2149.922939
TCGA-22-4596	0.896317535	1.407394838	4022.119274
TCGA-33-A5GW	-1.663732618	-1.677496318	-1973.180268
TCGA-60-2715	1.007280345	0.927548246	3656.714821
TCGA-85-8351	-0.681416568	-0.705960136	111.0597088
TCGA-58-A46M	-0.840818964	-0.134830896	515.5333289
TCGA-37-4129	-1.239574923	-2.055291471	-1885.729473
TCGA-21-1075	-1.343705296	-1.844400925	-1786.802298
TCGA-43-7656	-0.090858776	0.538176408	2036.735583
TCGA-58-8388	0.089264734	0.112345983	1803.413571
TCGA-98-A53J	-1.239306093	-1.204506216	-1018.411924
TCGA-85-7696	-0.529269532	-0.720546741	265.665985
TCGA-34-5231	-0.75985464	-0.523580758	209.5494687
TCGA-34-5929	-0.612780044	-0.544849088	351.6965223
TCGA-22-5492	-0.090780824	0.640593598	2141.193706
TCGA-98-A53C	1.003498559	1.560443192	4297.47291
TCGA-94-8491	-0.814272196	0.067727137	751.5256427
TCGA-90-7767	0.343819475	0.284381201	2262.270698
TCGA-96-8169	-0.216924423	-0.163677923	1181.069921
TCGA-34-2596	-0.440841875	-0.296373409	796.428912
TCGA-56-A62T	-0.450835192	-0.652174585	422.707903
TCGA-21-1071	-0.188850443	-0.373258574	998.7611144
TCGA-22-5480	-0.249783306	0.526066723	1847.374446
TCGA-77-A5GH	0.237583551	0.925908807	2797.706125
TCGA-L3-A4E7	-0.579201473	-0.198880471	741.6681489
TCGA-60-2723	-0.027081028	0.295027945	1859.987657
TCGA-37-3783	0.123468053	0.755313173	2496.746342
TCGA-66-2763	-0.623775803	-0.431092818	455.3754583
TCGA-63-6202	0.426532737	1.116402916	3202.298632
TCGA-L3-A524	-0.759368915	-1.2870164	-567.9113781
TCGA-98-8021	-0.296556857	-0.820011347	423.5140096
TCGA-77-A5GF	-0.577623262	-0.665158168	268.2518568
TCGA-66-2787	-0.101242217	0.201973846	1682.55258
TCGA-77-8138	0.023594205	-0.05752245	1557.155999
TCGA-63-A5MR	-1.057478832	-0.786482338	-389.8812712
TCGA-66-2768	-0.665640917	0.127360075	977.851482
TCGA-77-8146	-0.515778001	-0.564540204	439.6768405
TCGA-43-A56V	-0.848762092	-0.867840202	-240.3092521
TCGA-22-1005	0.500729345	1.53453317	3711.05172
TCGA-66-2780	0.063313958	-0.225556691	1430.158116
TCGA-77-8156	0.227028796	0.763493481	2620.435371
TCGA-66-2790	-0.089698054	-0.1199771	1367.317492
TCGA-98-8022	-0.231399048	0.122452117	1456.536508
TCGA-58-A46L	-0.324387174	-0.235944092	987.7259763
TCGA-O2-A52N	-0.746037753	-0.360205128	391.4324219
TCGA-63-7022	1.18948229	1.029190815	3963.245004
TCGA-37-4135	-0.648182328	-0.281967532	580.1604697
TCGA-66-2727	-0.489387005	-0.321037618	717.2214142
TCGA-34-7107	0.243194448	-0.65217267	1195.764871
TCGA-NC-A5HD	-1.375497688	-1.801573286	-1778.569933

TCGA-56-7582	-0.509233472	-0.589630584	421.3974598
TCGA-18-3415	0.000594676	0.120508611	1712.965507
TCGA-56-7580	-0.761493562	-0.792771793	-66.60322727
TCGA-33-AASL	-1.442896861	-0.556796864	-585.1169161
TCGA-43-2576	0.583600147	0.631943986	2883.548121
TCGA-77-A5FZ	1.132066428	2.03195983	4921.193206
TCGA-33-4583	-1.270133153	-0.561827384	-397.8080458
TCGA-46-6026	-1.547557067	-1.460578977	-1622.720348
TCGA-18-3414	-0.431176163	-0.270558195	833.5029711
TCGA-63-A5MY	-2.013117514	-1.998423609	-2689.398255
TCGA-56-8082	-1.142365034	-1.396746892	-1106.341313
TCGA-60-2712	0.559858756	1.173126388	3408.611507
TCGA-60-2698	-0.074610437	0.613986711	2132.090783
TCGA-52-7809	-0.951099625	-1.073871886	-564.2621402
TCGA-34-5232	-0.675741414	0.807023032	1659.231719
TCGA-18-4083	-0.225715623	-0.615814199	710.5147473
TCGA-37-A5EN	-1.047951346	-0.745504679	-337.5094363
TCGA-85-A510	-0.601216364	-0.028558129	890.718608
TCGA-60-2720	-0.206349421	0.365389178	1732.010662
TCGA-60-2710	-0.07012645	0.016629333	1528.330471
TCGA-94-8035	-1.240324448	-1.104517664	-917.6499144
TCGA-22-4593	0.166088882	-1.079838331	674.0542573
TCGA-18-3419	-0.976995543	0.041951235	544.0061711
TCGA-66-2793	-1.736304752	-1.91913874	-2300.268719
TCGA-21-1079	0.088224725	-0.724802118	949.1343818
TCGA-92-8064	0.390439822	0.94973276	2992.245816
TCGA-63-7023	-0.907736685	-0.82370019	-261.0167647
TCGA-60-2706	-0.290515537	0.359117018	1631.869177
TCGA-98-7454	1.210013697	1.187161064	4147.098508
TCGA-37-4141	-0.413420664	-0.193339431	931.9722782
TCGA-22-5477	-1.009830072	-0.881568355	-433.7072504
TCGA-33-4533	-0.255965975	-1.239315306	41.42253928
TCGA-77-8128	-0.347806922	-1.842208071	-675.2717043
TCGA-56-8309	0.405294493	1.458363863	3527.127616
TCGA-56-A5DR	-0.42083098	-0.683931589	423.7656072
TCGA-37-4130	-0.264922177	1.378773673	2699.488263
TCGA-58-A46J	-0.568734235	-0.189778445	762.6029249
TCGA-43-6143	-0.454766396	-1.214256769	-154.4775534
TCGA-77-A5G6	-1.270056515	-1.280201101	-1129.802861
TCGA-39-5031	-0.092376174	-0.034523902	1451.418062
TCGA-90-A59Q	0.108483961	1.139325003	2871.394811
TCGA-68-7755	-0.392078166	-1.340891973	-213.7027399
TCGA-66-2767	0.305379212	1.241848909	3195.189499
TCGA-66-2771	-0.006861092	1.02561288	2627.034312
TCGA-85-6560	0.327738037	0.08722643	2043.441701
TCGA-56-8304	-1.244057418	-0.461441911	-266.4623961
TCGA-56-7221	-1.418467126	-1.447760035	-1465.868113
TCGA-18-3421	-0.553400749	0.030926595	1004.598411
TCGA-51-6867	-0.508272571	-0.586144045	426.0208341
TCGA-NC-A5HL	-0.857783769	0.241291729	879.935822
TCGA-77-A5G1	-0.26467833	0.058919243	1354.722823
TCGA-96-7545	0.179857026	0.328405408	2124.50292
TCGA-NC-A5HM	-1.327556108	-0.627393722	-528.586774
TCGA-33-4589	-0.426096758	0.401897996	1524.447293
TCGA-85-A4QR	-2.240102839	-2.00716949	-2951.141914
TCGA-60-2711	-1.117592389	-0.636719359	-304.2191986
TCGA-33-4532	-1.520646959	-1.459354342	-1591.498133
TCGA-NC-A5HJ	-0.07531946	1.071201586	2597.239494

TCGA-21-5787	-0.076986682	0.659947707	2176.281894
TCGA-85-8479	-1.222625147	-1.514106294	-1315.338809
TCGA-34-5240	-0.094957645	-0.633517072	838.1207834
TCGA-77-8148	-0.319480509	-0.830576227	387.2137095
TCGA-56-8626	-0.447024901	-0.621228663	458.4884154
TCGA-33-A4WN	-1.103013923	-0.909978161	-566.4532997
TCGA-39-5027	-1.875687504	-1.669740129	-2201.365128
TCGA-56-8503	0.948596275	0.597748003	3255.255934
TCGA-56-8623	0.80265673	1.566976848	4080.420631
TCGA-77-6845	-1.141928717	-1.808257073	-1525.217047
TCGA-85-A50Z	-0.971546486	-0.113623078	391.5330352
TCGA-77-8144	-1.108975132	-0.850646854	-512.6299402
TCGA-85-A53L	-0.876871465	-0.971289483	-377.0423642
TCGA-XC-AA0X	-0.279532459	0.316572654	1600.746734
TCGA-66-2782	0.113764197	0.44834864	2173.116191
TCGA-68-A59I	0.114978828	0.118895133	1838.729696
TCGA-77-A5G7	-1.517395005	-1.329890131	-1455.941527
TCGA-90-A4EE	0.387485817	1.424555057	3472.837289
TCGA-98-8023	0.386182896	0.55795417	2588.250512
TCGA-34-8456	-0.659931382	-0.745519003	94.67765098
TCGA-56-5897	0.969634468	0.617896064	3299.222115
TCGA-66-2786	-0.282955158	0.155438561	1432.725805
TCGA-39-5037	-2.077191141	-1.805462474	-2564.124792
TCGA-56-6545	0.95940524	0.567335519	3236.302908
TCGA-NK-A5CT	-1.365318377	-0.685914235	-630.2859023
TCGA-22-1016	0.471527978	0.370213839	2491.990986
TCGA-39-5016	-0.925226359	0.264208168	828.1675918
TCGA-63-A5MM	0.444132406	0.753976437	2852.56076
TCGA-33-4538	-1.000256873	-0.859714626	-400.7733083
TCGA-63-A5MT	-0.02846392	0.019670133	1577.835705
TCGA-18-3416	-0.607403701	0.862869933	1792.263127
TCGA-66-2753	-0.214352986	-0.397576477	945.5728518
TCGA-63-7020	-0.530223751	0.048425844	1048.247579
TCGA-56-8308	-0.310705676	-0.483705012	750.4771498
TCGA-18-3408	-0.174404787	-0.150088682	1242.279558
TCGA-NK-A5D1	-1.346620595	-1.469319656	-1407.811784
TCGA-85-6798	-0.337000031	-1.408308882	-221.0563712
TCGA-22-4599	-0.084203178	0.828658941	2340.173913
TCGA-56-A4ZK	-0.47934305	0.182347235	1241.39838
TCGA-56-7731	0.343915835	0.340684384	2319.75547
TCGA-92-7341	-0.080345075	-0.808446778	676.1298696
TCGA-85-8353	0.463346061	0.150547669	2259.020071
TCGA-85-A4QQ	-1.341983268	-1.047909865	-973.1962033
TCGA-77-6842	0.039704245	0.942373853	2594.074575
TCGA-22-4595	0.146415288	0.066059315	1819.901737
TCGA-33-4587	-1.060061171	-1.410309454	-1028.48729
TCGA-56-A4BW	-0.346473056	-0.430062228	765.3034133
TCGA-21-1076	0.750871087	1.227073541	3676.349829
TCGA-6A-AB49	-2.048981267	-1.464918508	-2185.661321
TCGA-63-A5M9	-0.547210752	-0.554031657	415.3740621
TCGA-39-5022	0.59798522	1.254188986	3533.688484
TCGA-43-6771	-0.05715588	-0.276588335	1243.965712
TCGA-98-8020	0.086069597	-0.062706645	1621.462017
TCGA-77-7141	-0.95797164	-1.143952196	-643.3340653
TCGA-92-7340	0.844858624	0.134916627	2668.044114
TCGA-56-8622	-0.060328059	-0.553515337	958.2216085
TCGA-70-6722	0.355044922	0.104134397	2091.088439
TCGA-43-A56U	0.095712963	1.356456174	3078.443649

TCGA-66-2770	0.091997607	0.047699752	1740.57795
TCGA-85-7710	-0.850051088	-0.408637193	226.2195139
TCGA-58-8387	-0.674486809	0.538278411	1386.756951
TCGA-85-8352	-1.884326039	-1.781035167	-2324.405816
TCGA-21-5786	-0.436896551	-0.794468062	293.2252445
TCGA-33-AASI	-1.152409121	-0.155938391	146.9538731
TCGA-56-8625	0.874268388	1.349265386	3938.320956
TCGA-34-2600	-1.461514403	-1.220972782	-1282.702629
TCGA-60-2726	-0.958908155	-0.189225381	328.5656404
TCGA-77-8153	-1.335829735	-1.795557688	-1728.254831
TCGA-66-2791	0.280444326	0.728545159	2644.317905
TCGA-68-7756	-0.342205911	-0.680198694	515.1474039
TCGA-94-7557	-0.446308058	-0.59726559	483.7071664
TCGA-56-A5DS	-0.609167233	-0.011812958	898.9270843
TCGA-22-4613	-0.459989988	0.284405364	1366.960452
TCGA-77-8130	0.094454728	-0.959350204	717.0505348
TCGA-21-1080	-0.890470368	-0.520366904	67.33641259
TCGA-98-A53I	-0.354883206	0.361757119	1562.862786
TCGA-34-5236	0.535618774	-0.210101449	1971.991835
TCGA-21-5782	-0.16755779	-0.458604792	935.5036354
TCGA-60-2708	0.444166739	-0.878562927	1188.911034
TCGA-96-A4JK	-0.59144869	-0.367834569	555.8486904
TCGA-66-2788	0.556567708	0.378877677	2595.54287
TCGA-56-8624	-0.158541367	-0.565226603	836.8905707
TCGA-22-5473	-0.524521857	-0.734736401	256.4938548
TCGA-21-A5DI	-0.952575038	-1.20472115	-699.2513986
TCGA-85-8049	0.711913616	0.919527959	3319.542978
TCGA-21-5783	-0.353115913	-1.360969952	-190.7651316
TCGA-46-3765	-0.295376222	-0.180927557	1076.10649
TCGA-39-5021	-0.223779956	0.035648358	1376.563182
TCGA-90-6837	0.549140382	0.432135804	2641.544118
TCGA-39-5011	-0.226466693	1.091249317	2449.312186
TCGA-39-5019	-0.143053645	-0.167336511	1259.62357
TCGA-98-A539	-0.504645455	-0.537217503	479.9210024
TCGA-66-2781	-0.346751607	-0.433604375	761.3834151
TCGA-37-5819	-0.664221371	-0.638180927	199.2852556
TCGA-56-8201	0.735357503	1.259351128	3691.963235
TCGA-63-A5MB	-0.970366228	-0.780165409	-286.4121744
TCGA-60-2722	-1.067344925	-1.296775304	-920.9000416
TCGA-85-8048	0.500910362	1.047549956	3214.978569
TCGA-56-7223	-1.431409623	-2.418594296	-2469.641933
TCGA-43-8115	0.967909063	0.795600031	3478.394779
TCGA-18-5592	-0.877907725	-0.973701125	-380.6542734
TCGA-77-7337	0.400101294	-0.407404867	1619.975781
TCGA-60-2719	-0.894520498	-0.560302422	22.12763501
TCGA-22-5478	-0.09160751	-0.485053829	993.148279
TCGA-66-2757	-0.395520946	-0.21103022	933.881812
TCGA-18-3406	0.37104932	0.533500824	2546.473846
TCGA-77-A5GB	0.007621304	0.123355939	1723.693873
TCGA-21-1070	-0.178106506	0.791922883	2198.141347
TCGA-43-A475	-0.233990391	1.248261097	2600.939346
TCGA-85-7698	0.162544696	-0.099562644	1669.085712
TCGA-85-7843	-0.029724614	-0.435637103	1112.437034
TCGA-NC-A5HT	-1.307129411	-2.099370059	-2005.895623
TCGA-58-8393	-0.105833558	0.799548098	2286.414325
TCGA-66-2737	0.810442651	0.082770956	2576.568778
TCGA-LA-A7SW	-0.84434729	-0.888495483	-256.4411417
TCGA-77-8007	0.432450577	0.780921015	2867.007459

TCGA-85-8354	-1.436824566	-1.233222335	-1267.684769
TCGA-22-5474	-0.14247723	0.752144531	2197.290218
TCGA-77-7140	-0.547643649	-1.25428381	-298.7209849
TCGA-22-4601	-0.899676746	-0.964471102	-395.4958837
TCGA-66-2794	-0.68263032	0.652852243	1494.446067
TCGA-22-1017	-0.723302679	-0.031159973	752.0795019
TCGA-77-A5GA	-1.027964478	-0.884798586	-457.1983864
TCGA-56-7222	-1.297761112	-1.284463155	-1165.005403
TCGA-85-7699	0.286918249	0.718654618	2641.449728
TCGA-66-2758	-0.418199026	0.517585936	1651.139545
TCGA-85-7950	0.25904642	0.287874883	2171.405339
TCGA-39-5034	0.163117991	-0.378025284	1385.94862
TCGA-85-8052	-0.730537637	-0.760737291	0.523196319
TCGA-43-3394	-0.609123316	-0.969954687	-77.44692763
TCGA-NK-A5CX	-0.178087783	-0.037807729	1352.600456
TCGA-39-5035	-0.293094669	0.543751551	1817.153705
TCGA-43-6647	0.953774135	0.435364938	3095.54206
TCGA-18-4086	-0.000870125	-0.1140821	1472.267251
TCGA-NC-A5HF	-1.556843529	-1.749143096	-1927.134088
TCGA-39-5029	-0.338858167	0.017000911	1229.378404
TCGA-43-5668	0.034934413	0.892968587	2538.413716
TCGA-22-1012	-0.610828267	-0.426367073	474.6131717
TCGA-34-8454	0.186166713	0.720872876	2531.486745
TCGA-60-2709	0.924807945	1.042163078	3681.653404
TCGA-85-A512	-0.704786112	-0.98450126	-198.8264443
TCGA-NC-A5HI	-1.520259697	-0.984846338	-1107.50523
TCGA-18-3409	1.022772841	-0.021924487	2706.382835
TCGA-56-8307	-0.842067447	0.165571623	820.2768417
TCGA-85-8288	0.177079882	-0.289585106	1491.62788
TCGA-18-3410	-0.458029832	0.604510046	1695.356031
TCGA-22-1000	1.12781969	1.025356885	3890.65414
TCGA-66-2778	-0.613888581	0.125814925	1033.921943
TCGA-34-5239	0.72003301	0.593578981	2996.418848
TCGA-34-8455	-0.279039456	-0.690539919	574.9677448
TCGA-66-2765	-0.137345702	0.269638069	1711.293433
TCGA-85-8277	-0.879359863	-0.587116624	11.68871671
TCGA-92-8065	-0.290643286	0.292336328	1563.672028
TCGA-46-3766	1.362669416	1.188862453	4318.870154
TCGA-77-8131	0.570468128	1.375199186	3626.357227
TCGA-94-A5I4	-1.087248156	-1.047640499	-689.1812854
TCGA-18-3407	0.044318423	-0.426703783	1204.014715
TCGA-77-8139	-0.279462899	-0.669031913	596.4144617
TCGA-21-1078	-1.713981941	-1.540793979	-1889.840609
TCGA-18-3411	-0.195189465	-0.060683223	1310.239544
TCGA-66-2742	-0.293609479	-0.210162276	1048.281888
TCGA-56-1622	-0.973575014	-1.50670242	-1030.385555
TCGA-77-A5G3	-0.847283807	-0.939817834	-312.0135973
TCGA-22-5482	-0.52859754	-1.170124519	-191.7411611
TCGA-NK-A7XE	-1.548175091	-0.889428095	-1041.360389
TCGA-22-5489	0.774256734	0.791581641	3258.597253
TCGA-O2-A52W	-1.339418631	-0.909326034	-829.1115562
TCGA-52-7812	-0.567838359	0.409647616	1374.463829
TCGA-46-6025	-0.770390994	-1.029286895	-317.5415344
TCGA-58-8391	-1.202456474	-1.24288108	-1016.473489
TCGA-85-A50M	-0.303810775	-0.761354943	475.2096866
TCGA-18-3417	0.528508619	-0.309385127	1862.89409
TCGA-60-2724	0.195653036	-0.719036164	1074.670937
TCGA-94-A5I6	-0.714292417	-1.135628816	-363.4262269

TCGA-43-8116	-0.722492292	-0.242004581	538.1146724
TCGA-34-5241	0.521762584	0.565212339	2746.664575
TCGA-77-7338	0.185654039	-0.401234393	1387.39879
TCGA-56-7822	-0.63215466	-0.661987541	210.7423875
TCGA-85-A4JC	-0.058145288	-0.060314698	1463.263752
TCGA-66-2792	-0.381951511	-0.849172771	298.678112
TCGA-98-A53A	-1.321486615	-1.105670906	-1009.228821
TCGA-22-4594	0.253211861	0.584433791	2467.123638
TCGA-58-8386	0.060511021	0.208444019	1869.317402
TCGA-22-5481	-0.817917378	0.005295832	683.8429197
TCGA-66-2754	-1.257420784	-1.544179908	-1384.743851
TCGA-21-1082	-0.169184746	0.044479902	1446.3749
TCGA-58-8392	-1.459491657	-1.42529793	-1488.673205
TCGA-60-2713	0.642922066	0.553812392	2870.002372
TCGA-22-5479	-0.492718965	-0.479193679	552.3364072
TCGA-37-4133	-0.471803672	-0.08592387	976.4065488
TCGA-63-A5MU	-1.613156853	-1.572173571	-1809.513428
TCGA-66-2769	0.530188033	0.71096421	2904.582178
TCGA-LA-A446	-1.351920522	-1.013842412	-949.5475033
TCGA-66-2759	-0.217409374	0.077468864	1426.277582
TCGA-22-4609	0.427248531	0.757017069	2836.853056
TCGA-66-2773	-0.249806185	0.292210254	1609.030545
TCGA-66-2755	0.236081597	1.049058855	2921.532883
TCGA-77-8150	-0.635349478	-0.155280217	723.5590159
TCGA-37-A5EL	-1.722289315	-1.304676956	-1658.471798
TCGA-77-8009	-0.609876842	-1.058286614	-168.3035391
TCGA-33-AAS8	-1.750076503	-1.319973291	-1705.011149
TCGA-58-A46N	-1.49263495	-1.556757728	-1659.558377
TCGA-33-AASD	-0.983475829	-1.310981758	-841.958739
TCGA-63-A5MS	0.272861211	0.47019233	2372.58918
TCGA-43-A474	-0.776503153	-1.925638605	-1237.803618
TCGA-43-6770	-0.179774465	-0.678121653	698.1907052
TCGA-22-4607	-0.115660115	-0.274408955	1181.020882
TCGA-58-A46K	-1.04259884	-0.93466464	-524.3165733
TCGA-77-7465	-0.287447474	0.572268939	1852.505411
TCGA-18-4721	0.333071851	0.557928809	2529.066156
TCGA-85-A4CL	-0.449301659	0.262003273	1356.036283
TCGA-22-4604	0.672633412	0.461045952	2808.560377
TCGA-56-7730	-1.81482604	-1.902211169	-2370.480251
TCGA-39-5039	0.293116303	-0.226722404	1684.938849
TCGA-J1-A4AH	-1.354981998	-1.201593073	-1144.290489
TCGA-92-8063	-0.344979948	-0.65398471	538.771644
TCGA-46-3769	0.080099549	0.396555399	2082.836885
TCGA-43-8118	-0.270388245	-0.118521384	1167.536584
TCGA-NC-A5HO	-1.127438841	-0.669834549	-348.9338357
TCGA-22-4605	0.317007444	0.042416614	1985.824493
TCGA-60-2714	-0.321273022	0.172797633	1407.735174
TCGA-33-6738	-1.231833104	-0.748213974	-545.089797
TCGA-63-A5MV	0.063441802	0.144111316	1807.021733
TCGA-85-A4CN	-0.022299168	-0.645016847	907.3332975
TCGA-NC-A5HK	-1.456708816	-0.920225026	-970.8638791
TCGA-43-3920	0.40925344	0.084651563	2131.614824
TCGA-21-1072	-0.652784691	-1.130733725	-289.9264633
TCGA-98-A53B	-0.602255855	-0.266299572	647.2832426
TCGA-85-8072	-0.815309559	-0.353721643	320.8802019
TCGA-68-A59J	-0.555992509	-0.009216724	960.8022924
TCGA-66-2766	-0.601195979	-0.760675203	144.6555575
TCGA-NC-A5HR	-1.446959325	-1.147627343	-1191.745385

TCGA-NC-A5HE	0.032863758	1.438265135	3091.808011
TCGA-98-A53D	0.952636923	0.937286161	3605.773164
TCGA-66-2783	-0.317045328	-0.272811	958.3334738
TCGA-77-7138	-1.021233695	-0.930925319	-496.708029
TCGA-56-8629	-0.764008941	-0.907752517	-186.5795571
TCGA-79-5596	-1.129967349	-1.423886058	-1120.188948
TCGA-63-A5MJ	0.011287149	-0.752403768	835.3080579
TCGA-56-6546	0.047152891	0.715716423	2371.38934
TCGA-51-4081	-0.401094077	-1.696680237	-586.3218945
TCGA-34-5927	-1.011719183	-1.454157815	-1019.325844
TCGA-85-8582	0.616655654	-0.247105454	2024.545908
TCGA-77-8154	-1.087158282	-1.705362221	-1359.352131
TCGA-77-6844	-0.946326496	-0.673060225	-150.4864347
TCGA-85-8580	-1.297986173	-0.642936497	-511.4892015
TCGA-56-A4ZJ	0.736152164	0.51848883	2937.850543
TCGA-21-1083	-0.310036527	-0.123423868	1118.377745
TCGA-46-3767	1.160302847	0.721594391	3617.277739
TCGA-56-5898	-0.203583208	-0.31809394	1038.567976
TCGA-68-7757	0.124753777	-0.387080174	1333.988426
TCGA-56-A49D	-0.515793898	0.381656528	1403.909155
TCGA-18-3412	-0.627526255	-0.557116129	322.770147
TCGA-37-4132	-0.351186715	0.229154767	1431.847791
TCGA-22-5483	0.069624243	-0.431017688	1227.805757
TCGA-85-A4JB	-0.600699134	-0.978431616	-76.70219773
TCGA-60-2725	0.454113112	0.943070421	3056.379782
TCGA-77-7463	-1.050554803	-0.725744451	-320.2720846
TCGA-33-AASJ	-1.758934854	-1.36138548	-1757.080479
TCGA-43-7657	-0.388129964	-1.402233757	-271.8171543
TCGA-22-A5C4	-1.080066514	-0.961714049	-593.6159953
TCGA-90-7769	-1.233006016	-1.537592138	-1350.835641
TCGA-21-1077	-0.297388818	-0.343285109	908.4094192
TCGA-NC-A5HQ	-1.116877175	-0.094776588	248.8604099
TCGA-60-2696	-0.242803192	0.164990747	1487.184096
TCGA-22-4591	-1.146020082	-1.012053724	-718.3794855
TCGA-77-8143	-1.378625915	-0.973879931	-938.5687352
TCGA-NK-A5CR	-1.425994048	-1.590847477	-1620.069614
TCGA-56-7823	-1.260794526	-1.227677378	-1065.960391
TCGA-63-A5MN	0.70102641	1.384218798	3780.973175
TCGA-52-7622	-0.011660087	0.560756603	2147.963216
GSM442070	2.697738748	2.371455702	7011.113512
GSM442071	2.772890515	2.05678316	6774.145956
GSM442072	2.941422207	2.988864666	7911.732913
GSM442073	2.9397058	2.001323702	6903.437974
GSM442074	2.837232427	2.257069868	7049.922279
GSM442075	1.98441281	1.581888308	5411.932814
GSM442076	2.687737564	1.534477871	6147.026306
GSM442077	2.018620732	1.320083694	5183.235999
GSM442078	1.356742853	0.82170737	3938.108419
GSM442079	2.281471036	2.029122586	6198.582882
GSM442080	1.810517517	2.036788135	5681.816255
GSM442081	1.705631912	0.774590137	4278.707353
GSM442082	2.929478689	2.484583065	7384.526235
GSM442083	2.399748643	2.28623977	6592.351057
GSM442084	3.351391216	2.646245806	8019.226886
GSM442085	2.72195235	1.446150386	6095.124184
GSM442086	3.129381995	2.936697632	8067.932286
GSM442087	2.750087082	1.725862087	6411.511023
GSM442088	1.219063373	0.726215189	3687.437969

GSM442089	1.597461131	1.6156016	5015.277588
GSM442090	2.891759378	1.360508033	6196.98982
GSM442091	2.197847873	2.23601327	6316.276131
GSM442092	2.259137777	1.420436974	5553.407415
GSM442093	2.370331124	0.835543945	5081.209045
GSM442094	2.475479546	1.146757619	5515.481729
GSM442095	2.182327377	2.233268524	6296.191286
GSM442096	3.117629813	3.068250606	8188.904931
GSM442097	1.444176352	1.901447425	5135.838825
GSM442098	0.447125659	-0.981458832	1087.347727
GSM442099	2.434642981	1.819695544	6155.772805
GSM442100	2.730365022	2.853036507	7538.224057
GSM442101	3.017478354	2.048162707	7037.798751
GSM442102	2.667876374	2.011835005	6611.368668
GSM442103	1.490229907	0.830261119	4095.511871
GSM442104	3.138556496	2.029518841	7153.663814
GSM442105	2.171029725	1.910094437	5954.267032
GSM442106	2.725305133	2.882274263	7562.383633
GSM442107	2.169842164	1.621972883	5659.32538
GSM442108	2.12294333	1.434980021	5416.525774
GSM442109	1.83650668	0.767596257	4417.356796
GSM442110	2.266863597	1.373133609	5513.807026
GSM442111	2.482409163	1.760883642	6149.043837
GSM442112	2.978553452	2.105678609	7053.054979
GSM442113	2.947800297	2.297346586	7214.125102
GSM442114	1.818787816	0.990734136	4625.015698
GSM442115	3.081451171	2.422407859	7490.441498
GSM442116	3.101002552	3.028544866	8129.921096
GSM442117	2.421400239	2.239411275	6568.746027
GSM442118	2.509039217	0.663722304	5060.6111
GSM442119	2.88453334	2.012739002	6853.616428
GSM442120	2.755171065	1.96915701	6665.110846
GSM442121	2.779030932	2.28600139	7014.577228
GSM442122	2.053854189	2.377662573	6300.23862
GSM442123	2.133495395	1.047158158	5033.0579
GSM442124	0.871402206	-0.616180591	1932.182028
GSM442125	1.802260307	0.694847549	4305.074234
GSM4750621	0.041022961	0.253772785	1893.803974
GSM4750622	0.334130171	0.594278526	2567.288245
GSM4750623	-0.152747057	-0.943573256	457.7791963
GSM4750624	0.025077654	-0.296217167	1315.559385
GSM4750625	0.388209126	-0.133379941	1885.982796
GSM4750626	-0.226927792	0.377723775	1721.659083
GSM4750627	-0.026344383	-0.070026254	1488.788803
GSM4750628	-0.268265613	-0.170348452	1117.084988
GSM4750629	0.359421742	-1.343777685	620.4256001
GSM4750630	0.269695678	-0.158852488	1728.016308
GSM4750631	-0.282877385	-0.692600727	568.592683
GSM4750632	1.355308796	1.222960033	4345.419577
GSM4750633	-0.714930036	-1.150553882	-379.3462828
GSM4750634	0.835987339	0.87402773	3411.375934
GSM4750635	0.214645716	-0.498824414	1320.239568
GSM4750636	-0.411849616	-0.064630993	1064.886388
GSM4750637	-0.25408038	-0.516162555	780.4732322
GSM4750638	0.460089734	1.149248397	3273.14875
GSM4750639	-0.077566779	0.298320784	1807.109033
GSM4750640	0.641628239	0.736354226	3054.585928
GSM4750641	-0.433705195	-0.476312948	621.0054491

GSM4750642	0.750537733	0.980603642	3424.806011
GSM4750643	0.406895201	0.549862648	2603.075308
GSM4750644	-0.962137284	-0.350487895	160.6294506
GSM4750645	0.177041145	-0.532796523	1243.732878
GSM4750646	0.820204286	0.381038028	2891.399881
GSM4750647	-1.218054847	-0.642720121	-422.2360252
GSM4750648	-0.563571734	-1.412323509	-477.5177854
GSM4750649	1.379600156	1.54198632	4697.590121
GSM4750650	0.774903699	1.337743146	3815.900068
GSM4750651	0.249407826	-0.926871552	922.7456385
GSM4750652	0.18230298	-1.337900185	429.1289308
GSM4750653	-0.525163686	-1.067756128	-83.5947486
GSM4750654	0.518275543	1.301387722	3493.00198
GSM4750655	-0.255734749	-0.862255752	425.9338871
GSM4750656	0.766209417	0.590424128	3044.638064
GSM4750657	1.144106496	0.494871335	3368.188301
GSM4750658	0.663304488	-0.621992212	1694.466852
GSM4750659	-0.207522292	0.200991977	1563.170369
GSM4750660	0.007618057	0.581611125	2190.688882
GSM4750661	-1.139424998	-1.148992962	-850.5854734
GSM4750662	0.531002803	0.211077097	2396.064838
GSM4750663	0.162302543	0.011524399	1782.022553
GSM4750664	1.492859245	1.090121141	4363.25871
GSM4750665	0.595703246	0.310255517	2569.203098
GSM4750666	1.744369238	1.30627834	4863.688221
GSM4750667	1.271956673	0.800993773	3822.559289
GSM4750668	0.709391577	0.688025453	3080.814229
GSM4750669	-0.460676614	-0.488111189	578.9395911
GSM4750670	-0.223220507	-0.901219989	422.4426805
GSM4750671	0.715041191	0.529767377	2925.829518
GSM4750672	-1.119967153	-0.891314654	-566.3172985
GSM4750673	0.274194476	0.358271165	2260.017653
GSM4750674	0.356053335	0.821404352	2823.167032
GSM4750675	0.301090547	0.421866157	2354.784608
GSM4750676	1.174807505	1.230692942	4152.246038
GSM4750677	1.13749346	0.735401833	3605.942057
GSM4750678	-0.281430056	-0.325387259	944.4246574
GSM4750679	-0.212799286	-0.989727923	343.8538564
GSM4750680	-0.166011235	0.150240776	1557.688537
GSM4750681	-0.543364562	-0.146681711	834.7803172
GSM4750682	0.515444946	1.085689795	3270.035669
GSM4750683	-0.881213563	-0.679031974	-84.045135
GSM4750684	1.044560444	1.717928354	4503.700243
GSM4750685	0.286058868	-0.524445569	1373.674144
GSM4750686	-0.367988873	0.017581534	1197.522444
GSM4750687	-1.291109861	-0.741384792	-604.1565946
GSM4750688	0.770648717	0.416930918	2872.77941
GSM4750689	-0.287729863	-0.488058022	771.6330298
GSM4750690	-0.773362565	-1.316619847	-613.6666843
GSM4750691	-0.494598423	0.045866396	1085.321087
GSM4750692	0.664722958	0.950268951	3298.306502
GSM4750693	-0.071379325	-0.342685006	1160.764936
GSM4750694	-0.804034798	-0.876649598	-199.4665832
GSM4750695	0.72560859	1.304800591	3727.420899
GSM4750696	-0.451746345	-0.435014397	642.9965723
GSM4750697	1.556588761	0.422742384	3754.132516
GSM4750698	0.701831069	0.212199925	2587.48864
GSM4750699	0.837363967	0.978805925	3519.686659

GSM4750700	0.254039885	0.062290557	1935.940296
GSM4750701	0.56490966	1.084478226	3323.897974
GSM4750702	-0.554689575	-0.394244557	569.8794799
GSM4750703	0.191878734	1.142323613	2967.341115
GSM4750704	-0.774960327	-0.647323641	66.61990098
GSM4750705	0.239408255	0.579952759	2447.181761
GSM4750706	-0.155591105	-0.597400718	807.3887703
GSM4750707	-0.348863147	-0.611143607	578.1047911
GSM4750708	-0.041352719	0.061535969	1606.143958
GSM4750709	0.496556478	1.155848936	3320.49424
GSM4750710	0.090534814	-0.466428473	1215.010883
GSM4750711	0.547621039	0.778382575	2992.704882
GSM4750712	0.649985304	0.805242499	3134.097217
GSM4750713	1.381852756	0.750954904	3893.975038
GSM4750714	0.201267243	-0.474514695	1330.111306
GSM4750715	0.881769321	1.129130902	3722.341362
GSM4750716	-1.428868046	-1.518094374	-1549.129636
GSM4750717	0.947812393	1.349585893	4020.56564
GSM4750718	-1.102398338	-0.715730428	-367.8136718
GSM4750719	1.144222472	1.646418842	4541.836341
GSM4750720	-0.408143215	0.373002615	1514.998406
GSM4750721	0.908200738	0.461129095	3071.0352
GSM4750722	0.21790271	0.958429433	2808.925447
GSM4750723	0.159767769	0.197054042	1968.268679
GSM4750724	0.667959903	1.09882248	3453.299928
GSM4750725	0.215685264	0.587061885	2428.002329
GSM4750726	0.20282005	-0.059714166	1754.555783
GSM4750727	1.240556604	1.929487033	4937.608606
GSM4750728	0.646789807	0.419978545	2737.923135
GSM4750729	-0.61249995	-0.038840944	867.671213
GSM4750730	0.340957815	0.197176021	2170.214156
GSM4750731	0.401316931	-0.615884204	1408.872745
GSM4750732	-1.183736299	-0.772648072	-516.4167683
GSM4750733	0.932092025	0.637888213	3277.778514
GSM4750734	-0.546030816	-0.373352279	600.815064
GSM4750735	0.662464195	0.408351416	2743.533306
GSM4750736	0.766843559	-0.035431631	2407.547421
GSM4750737	0.582009003	0.336329709	2580.52125
GSM4750738	0.751158441	0.725159567	3165.179485
GSM4750739	-0.310252317	-0.069887588	1172.695128
GSM4750740	0.728886297	0.374767632	2783.293975
GSM4750741	0.128406646	0.376152326	2115.852087
GSM4750742	0.736579198	1.160176788	3592.257468
GSM4750743	1.359711789	1.740107613	4877.338592
GSM4750744	1.081809799	1.259200896	4077.711071
GSM4750745	-0.613430535	-1.155160017	-270.9836053
GSM4750746	0.77070045	0.876045636	3340.71159
GSM4750747	0.233199625	-0.313177478	1530.095161
GSM4750748	1.253142332	1.16792179	4175.53161
GSM4750749	-0.54135378	0.460678607	1455.968717
GSM4750750	0.333657358	0.232001837	2197.572723
GSM4750751	0.655972206	0.531868529	2862.175911
GSM4750752	-0.793745184	-1.166630685	-483.5192288
GSM4750753	1.26232953	1.506485684	4530.788545
GSM4750754	1.125342503	0.229140836	3076.487157
GSM4750755	-1.515606808	-1.551968445	-1680.265252
GSM4750756	0.903049162	1.489415605	4113.203104
GSM4750757	1.444079044	1.073619792	4292.10798

GSM4750758	0.660582237	0.6281501	2965.42947
GSM4750759	0.119337564	0.861470977	2600.328816
GSM4750760	0.31957302	1.177176039	3145.092659
GSM4750761	0.842704539	-0.383457901	2137.379718
GSM4750762	-1.066003092	-1.702353333	-1332.721818
GSM4750763	0.687910046	1.337521245	3718.774786
GSM4750764	-0.115123031	-0.131372146	1327.38505
GSM4750765	0.018327715	0.168147562	1781.265605
GSM4750766	-0.007738996	0.897014876	2495.004811
GSM4750767	-0.416627741	-1.225521417	-123.4758342
GSM4750768	0.697689212	0.86010796	3243.145195
GSM4750769	0.090781288	-0.254630747	1431.124216
GSM4750770	-0.851483668	-1.457060585	-843.8033384
GSM4750771	-0.322897066	0.821646623	2067.155139
GSM4750772	0.787963314	0.741922392	3223.257787
GSM4750773	-0.233973199	-0.118321195	1208.302021
GSM4750774	0.207015742	-0.042386813	1776.887172
GSM4750775	0.006475376	-1.197482074	376.3780621
GSM4750776	-0.123960688	0.059680374	1512.23887
GSM4750777	0.319104499	0.315824708	2266.785007
GSM4750778	0.444377852	-0.603138265	1469.825908
GSM4750779	-0.682425837	-1.226063793	-420.0916356
GSM4750780	0.329097087	0.484093403	2449.394634
GSM4750781	0.470945219	0.552720135	2677.330359
GSM4750782	-0.621907872	-0.479522287	408.1025854
GSM4750783	0.811157612	0.300076081	2798.816422
GSM4750784	0.850173019	-0.084684463	2450.172584
GSM4750785	0.845209069	0.05786708	2589.914821
GSM4750786	0.237006012	-1.050040851	783.4123456
GSM4750787	0.676956316	0.225450612	2573.285044
GSM4750788	0.500164529	1.19483513	3364.243165
GSM4750789	0.023341012	-0.626948093	976.5837783
GSM4750790	0.97007695	0.82599372	3511.783107
GSM4750791	0.708082669	0.443477447	2830.142338
GSM4750792	0.438070902	1.207263246	3307.744517
GSM4750793	1.410953298	0.23620484	3401.817681
GSM4750794	0.370029183	-1.155431368	824.1807898
GSM4750795	0.065576452	0.767514196	2444.696756
GSM4750796	0.813421389	0.853104106	3364.917678
GSM4750797	-0.392844658	-0.154199798	994.7775719
GSM4750798	0.91612023	0.444324294	3062.731008
GSM4750799	-0.14864335	-0.257809499	1161.198195
GSM4750800	1.059260945	1.621054085	4421.351995
GSM4750801	0.823422588	0.564322458	3081.766102
GSM4750802	0.141544804	-0.764806898	967.7576627
GSM4750803	-0.669260227	-0.261865813	577.1678017
GSM4750804	0.267295316	0.193350357	2084.265452
GSM4750805	0.462029383	0.552183843	2666.852801
GSM4750806	0.28281251	-0.045114589	1858.534683
GSM4750807	-0.391817263	-1.47988438	-355.056491
GSM4750808	-0.49165203	-0.585748656	444.9367991
GSM4750809	0.135645924	0.327674908	2074.513302
GSM4750810	0.858170964	0.803529329	3364.241988
GSM4750811	-0.152249431	0.177467155	1600.763177
GSM4750812	0.822205746	1.44898623	3981.953726
GSM4750813	0.147848913	0.260269569	2019.414357
GSM4750814	0.219797996	1.294185608	3153.198891
GSM4750815	0.631917797	0.263538335	2561.932663

GSM4750816	-1.279279802	-1.413570042	-1275.989952
GSM4750817	-1.296266049	-0.488311833	-351.9983358
GSM4750818	0.269805159	0.31453641	2210.559341
GSM4750819	-0.3471109	-0.201380421	997.6379633
GSM4750820	0.608163164	0.272272406	2544.373935
GSM4750821	-0.481741878	-0.612476381	428.737679
GSM4750822	0.699627937	0.949558579	3336.461995
GSM4750823	0.421080992	0.341842502	2406.887218
GSM4750824	0.527885506	-0.386372558	1783.743689
GSM4750825	-0.062030986	-0.291620713	1223.216312
GSM4750826	0.517696276	-0.655664398	1497.964379
GSM4750827	-0.575598787	-0.787991435	145.32994
GSM4750828	0.840258803	1.197407346	3745.683416
GSM4750829	0.507492374	1.093340051	3268.973802
GSM4750830	-0.789041332	-0.968578178	-276.4484554
GSM4750831	0.826735184	0.763134984	3288.061724
GSM4750832	0.890233368	0.991170338	3591.176334
GSM4750833	-0.264343963	-0.863289784	415.290628
GSM4750834	0.281733495	0.667304094	2583.344331
GSM4750835	1.057638402	0.702476755	3483.441056
GSM4750836	1.141084475	1.090077825	3971.385118
GSM4750837	0.261982581	-0.017197456	1863.782751
GSM4750838	0.126866562	0.484849731	2224.907978
GSM4750839	0.656230749	0.647089339	2979.883098
GSM4750840	0.702059034	0.524350878	2905.849308
GSM4750841	0.485517389	0.941258106	3089.512999
GSM4750842	1.166975531	1.09653143	4006.800987
GSM4750843	0.784056256	0.89334444	3373.217012
GSM4750844	0.458488861	-0.047369539	2051.91637
GSM4750845	0.621153774	1.007216972	3307.810913
GSM4750846	0.332766672	1.024965652	3004.674064
GSM4750847	-0.532478612	-0.610713815	374.0200255
GSM4750848	-0.017337519	0.318431023	1894.690211
GSM4750849	0.81255074	0.181477682	2679.506939
GSM4750850	1.895052121	1.714392665	5447.42963
GSM4750851	0.448695099	0.954305752	3061.79455
GSM4750852	0.457725952	-1.322346884	751.7628729
GSM4750853	1.389435957	1.209126043	4369.334671
GSM4750854	0.881708805	1.084386238	3676.675571
GSM4750855	0.226586695	0.357412563	2206.114049

Table-S4: Differentially expressed genes of ICI-clusters in the TCGA cohort

ICI cluster-C1 VS ICI cluster-C2

Tag	logFC	AveExpr	t	P.Value	adj.P.Val	B
SELPLG	2.06299	2.79675	22.1845	1.20E-70	6.53E-66	150.205
LAPTM5	2.1324	5.5131	21.843	3.33E-69	9.03E-65	146.91
LCP2	1.95743	1.58273	21.7139	1.17E-68	2.11E-64	145.663
GIMAP4	2.01607	2.63862	21.6742	1.72E-68	2.34E-64	145.279
SLC7A7	2.08252	1.59984	21.6057	3.35E-68	3.03E-64	144.617
CD4	2.11106	3.48861	21.4677	1.29E-67	9.97E-64	143.283
WIPF1	1.57823	2.30684	21.3799	3.03E-67	2.05E-63	142.433
LAT2	1.63336	1.01571	21.2668	9.12E-67	5.50E-63	141.338
EVI2A	2.3194	1.47352	21.2423	1.16E-66	6.29E-63	141.1
TNFRSF1I	1.87478	3.16716	21.172	2.30E-66	1.13E-62	140.42
CD86	1.87343	1.68981	21.0926	4.99E-66	2.14E-62	139.65
FLI1	1.84544	0.4217	21.0899	5.12E-66	2.14E-62	139.624
CD53	2.19526	4.00532	21.0722	6.09E-66	2.21E-62	139.452
ZEB2	1.82543	0.14289	21.0734	6.02E-66	2.21E-62	139.465
SLA	2.13954	0.99526	21.0362	8.66E-66	2.94E-62	139.104
PLXNC1	1.97861	0.87785	20.9403	2.21E-65	7.06E-62	138.174
GMFG	1.76521	2.87038	20.8742	4.21E-65	1.27E-61	137.533
PILRA	1.96998	1.56366	20.851	5.28E-65	1.51E-61	137.308
TNFSF13I	2.29196	1.27907	20.8131	7.65E-65	2.08E-61	136.941
RCSD1	2.04848	0.56499	20.796	9.05E-65	2.34E-61	136.774
PTPRC	2.39971	2.00541	20.7072	2.16E-64	5.32E-61	135.913
SLAMF8	2.15072	2.34505	20.6923	2.49E-64	5.64E-61	135.768
SAMSN1	2.07237	1.29502	20.6965	2.39E-64	5.64E-61	135.809
HAVCR2	2.10545	1.68603	20.6551	3.59E-64	7.79E-61	135.407
IL10RA	2.16853	1.62172	20.6288	4.64E-64	9.68E-61	135.152
FCGR2A	2.06379	2.47155	20.5349	1.16E-63	2.33E-60	134.241
ARHGAP1	1.99499	-0.9038	20.5263	1.26E-63	2.45E-60	134.157
CYBB	2.51923	3.06418	20.4196	3.59E-63	6.72E-60	133.121
CTSS	2.08092	4.39041	20.3455	7.41E-63	1.34E-59	132.401
GPR65	2.18654	-0.7744	20.3364	8.10E-63	1.42E-59	132.312
FPR3	2.37315	2.47642	20.2812	1.39E-62	2.36E-59	131.776
NCKAP1L	2.23837	0.98216	20.2635	1.65E-62	2.72E-59	131.605
C3AR1	2.33151	2.20272	20.2395	2.09E-62	3.34E-59	131.371
EVI2B	2.2662	2.35829	20.2263	2.38E-62	3.69E-59	131.243
SASH3	2.07588	2.24757	20.2217	2.49E-62	3.75E-59	131.198
HLA-DRA	2.37644	8.47013	20.2067	2.88E-62	4.21E-59	131.053
LAIR1	2.1689	1.11796	20.2043	2.95E-62	4.21E-59	131.029
TNFAIP8L	1.87173	1.3176	20.1343	5.85E-62	8.15E-59	130.349
TRPV2	1.74891	2.00747	20.0946	8.64E-62	1.17E-58	129.962
HLA-DRB	2.25532	7.55642	20.0782	1.01E-61	1.34E-58	129.804
CSF1R	2.18341	2.84192	20.0633	1.17E-61	1.48E-58	129.659
DOCK8	1.99085	0.43815	20.0646	1.16E-61	1.48E-58	129.672
FCER1G	1.9143	4.82963	20.0321	1.59E-61	1.92E-58	129.355
BTK	2.07069	0.40044	20.0278	1.66E-61	1.96E-58	129.313
CD74	2.13488	8.53132	19.9851	2.52E-61	2.91E-58	128.899
DOCK2	2.20056	0.20581	19.9754	2.77E-61	3.14E-58	128.804
RFTN1	1.76473	2.20017	19.9595	3.24E-61	3.59E-58	128.65
CYTIP	2.19479	1.60593	19.9536	3.43E-61	3.73E-58	128.592
CYTH4	1.87067	1.12221	19.9372	4.03E-61	4.29E-58	128.433
CD14	1.91413	4.90776	19.9206	4.74E-61	4.86E-58	128.271
WAS	1.79546	1.93492	19.9217	4.69E-61	4.86E-58	128.282
CXorf21	2.19535	-0.3191	19.9087	5.33E-61	5.36E-58	128.156
CD28	2.28976	-0.9905	19.8585	8.71E-61	8.60E-58	127.667
RNASE6	2.04782	2.78932	19.8514	9.34E-61	8.89E-58	127.599
AIF1	1.84483	3.5414	19.8531	9.19E-61	8.89E-58	127.615
SNX20	2.09198	-0.1665	19.8281	1.17E-60	1.10E-57	127.372
GIMAP6	2.04318	1.28209	19.8075	1.43E-60	1.32E-57	127.172
LILRB1	2.10121	0.13624	19.7995	1.55E-60	1.40E-57	127.094
NCF4	1.81716	2.29076	19.7851	1.79E-60	1.59E-57	126.954
PTPN22	2.0253	-0.3388	19.7833	1.82E-60	1.59E-57	126.937
SRGN	2.18074	5.75873	19.7754	1.97E-60	1.69E-57	126.86
HLA-DMI	2.24356	2.69919	19.7617	2.25E-60	1.91E-57	126.727
TMEM27:	1.94739	0.14345	19.759	2.31E-60	1.93E-57	126.7
GPR183	2.20997	2.35038	19.7336	2.96E-60	2.43E-57	126.453
THEMIS2	1.85972	2.41868	19.6954	4.30E-60	3.48E-57	126.082
HLA-DPA	2.31029	4.71769	19.6876	4.64E-60	3.71E-57	126.006
TYROBP	1.98381	5.28513	19.6826	4.88E-60	3.84E-57	125.957
IL12RB1	2.10978	-0.4307	19.6775	5.12E-60	3.97E-57	125.908
HLA-DPB	2.09788	5.59031	19.666	5.74E-60	4.39E-57	125.796
LILRB2	2.02442	0.69647	19.5343	2.08E-59	1.57E-56	124.515
ITGB2	2.12887	3.48743	19.5025	2.85E-59	2.12E-56	124.206
SERPING1	1.76904	5.64148	19.4584	4.38E-59	3.21E-56	123.777
MNDA	2.29931	1.75117	19.4516	4.69E-59	3.39E-56	123.711
CSF2RB	2.33927	1.41144	19.4181	6.51E-59	4.59E-56	123.385
MYO1F	1.75222	1.07589	19.4186	6.47E-59	4.59E-56	123.39
MEF2C	1.72142	0.14614	19.3843	9.06E-59	6.31E-56	123.056
IL16	1.93683	-0.0593	19.377	9.73E-59	6.69E-56	122.985
SLCO2B1	2.46108	1.6721	19.3558	1.20E-58	8.12E-56	122.78
SPI1	1.87901	3.26956	19.2864	2.36E-58	1.58E-55	122.105
MILR1	1.96923	0.40655	19.2844	2.41E-58	1.60E-55	122.085
FERMT3	1.77295	2.81666	19.2779	2.57E-58	1.68E-55	122.022
HLA-DOA	2.50583	3.58274	19.2677	2.84E-58	1.83E-55	121.923
IL2RA	2.41488	0.79356	19.2086	5.06E-58	3.23E-55	121.348
GPSM3	1.67632	3.27221	19.1807	6.66E-58	4.20E-55	121.076
TAGAP	2.13722	0.69447	19.136	1.03E-57	6.43E-55	120.642
GIMAP1	1.87914	-0.6543	19.108	1.36E-57	8.36E-55	120.37
IL2RG	2.12878	3.01779	19.0495	2.40E-57	1.47E-54	119.801
CCR1	2.18349	1.55805	19.0448	2.52E-57	1.52E-54	119.755
CST7	1.98831	2.34275	19.0208	3.18E-57	1.90E-54	119.522
ABI3	1.67751	1.78333	19.0053	3.71E-57	2.19E-54	119.371
SPARC	1.88965	7.63135	18.9928	4.19E-57	2.44E-54	119.25
NLRP3	2.13541	-1.1377	18.9837	4.58E-57	2.64E-54	119.161
CCR5	2.26887	0.56924	18.9735	5.06E-57	2.89E-54	119.062
MPEG1	2.21915	2.18539	18.9446	6.72E-57	3.80E-54	118.781
SIGLEC10	2.19223	1.02375	18.9373	7.21E-57	4.04E-54	118.71
FCGR3A	2.27685	3.95997	18.9261	8.05E-57	4.46E-54	118.601
ANXA6	1.7243	3.57487	18.9093	9.49E-57	5.20E-54	118.438
FGL2	2.27226	1.9037	18.8555	1.61E-56	8.72E-54	117.914
LPXN	1.66417	2.40472	18.8076	2.57E-56	1.38E-53	117.449
CD84	2.4021	0.22782	18.7896	3.06E-56	1.61E-53	117.274
GIMAP7	1.94822	2.09077	18.7899	3.05E-56	1.61E-53	117.277
C1QC	2.19458	6.14125	18.7883	3.10E-56	1.62E-53	117.261
PCED1B-.	2.00505	0.56918	18.784	3.23E-56	1.67E-53	117.22
SIGLEC9	2.02018	-0.0646	18.7656	3.87E-56	1.98E-53	117.041
CCR4	2.86025	-1.1984	18.7608	4.06E-56	2.06E-53	116.994
IL21R	2.27636	-0.6568	18.7237	5.84E-56	2.94E-53	116.633
MS4A6A	2.17955	2.10188	18.7115	6.58E-56	3.28E-53	116.514
NFAM1	2.1078	0.48188	18.67	9.88E-56	4.87E-53	116.112
HCST	1.81798	2.1791	18.6667	1.02E-55	4.99E-53	116.08
OLFML1	1.98135	0.72934	18.6511	1.19E-55	5.76E-53	115.928
CMKLR1	2.27605	0.6666	18.6474	1.23E-55	5.92E-53	115.892
TLR8	2.72701	-0.836	18.6164	1.67E-55	7.94E-53	115.591
C1orf162	1.76208	1.59534	18.6008	1.94E-55	9.18E-53	115.439
GNGT2	1.72384	-0.4898	18.5814	2.35E-55	1.10E-52	115.25
FCGR1A	2.14953	0.13899	18.5664	2.72E-55	1.26E-52	115.105
CD37	1.91133	1.92044	18.5529	3.10E-55	1.43E-52	114.974
HLA-DMA	1.87419	4.20223	18.5462	3.32E-55	1.51E-52	114.909
LRRC25	1.87736	1.21185	18.5068	4.88E-55	2.21E-52	114.526
CD300LF	2.12285	0.59542	18.4896	5.77E-55	2.59E-52	114.359
GYPC	1.8909	1.68893	18.4864	5.96E-55	2.65E-52	114.327
CCR2	2.66233	-0.7514	18.4587	7.80E-55	3.44E-52	114.059
BIN2	1.92974	0.76067	18.4383	9.53E-55	4.17E-52	113.861
MS4A4A	2.29287	1.97435	18.4357	9.77E-55	4.24E-52	113.836
ITK	2.44017	-1.2272	18.4239	1.10E-54	4.73E-52	113.721
PLEK	2.14419	2.7276	18.4049	1.32E-54	5.64E-52	113.537
TNFSF8	2.48371	#NAME?	18.3667	2.09E-54	8.85E-52	113.083
CD5	2.15388	0.44053	18.3567	2.12E-54	8.90E-52	113.068
TM6SF1	1.91092	-0.6664	18.3539	2.18E-54	9.09E-52	113.041
CORO1A	1.66251	2.98947	18.3444	2.39E-54	9.89E-52	112.949
LST1	1.81693	1.62712	18.3289	2.78E-54	1.14E-51	112.799
ARHGEF6	1.76887	0.93571	18.3093	3.37E-54	1.37E-51	112.608
DOK2	1.89805	1.73178	18.2992	3.71E-54	1.50E-51	112.51
IGSF6	1.98029	1.48339	18.2903	4.05E-54	1.63E-51	112.424
CD80	2.11416	-1.5464	18.275	4.71E-54	1.88E-51	112.275
ICOS	2.21385	#NAME?	18.2329	7.70E-54	3.05E-51	111.786
C1QA	2.07956	6.10547	18.1841	1.14E-53	4.50E-51	111.392
PMP22	1.62024	3.99977	18.1807	1.18E-53	4.62E-51	111.36
FCGR2B	2.16694	-0.0583	18.1771	1.22E-53	4.75E-51	111.325
STARD8	1.71138	-0.0823	18.1708	1.30E-53	5.02E-51	111.263
ECM2	1.95688	0.26092	18.1639	1.39E-53	5.33E-51	111.196
TLR7	2.50016	-1.284	18.0964	2.69E-53	1.02E-50	110.542
OLFML2B	1.96485	2.74931	18.0806	3.15E-53	1.19E-50	110.388
SIRPB2	2.14589	-0.8253	18.074	3.36E-53	1.26E-50	110.324
CSF2RA	2.06065	0.44538	18.0537	4.09E-53	1.52E-50	110.127
SCIMP	2.2367	-0.5078	18.0477	4.34E-53	1.60E-50	110.07
FAM78A	1.65675	0.1744	18.0382	4.76E-53	1.75E-50	109.977
PIK3CG	2.46712	-0.8824	18.0362	4.85E-53	1.76E-50	109.958
CD3E	2.11578	1.99802	18.0368	4.82E-53	1.76E-50	109.964
CD163	2.53697	2.6493	18.0347	4.93E-53	1.77E-50	109.943
APBB1IP	1.98711	0.90566	17.9963	7.17E-53	2.56E-50	109.571
VIM	1.71632	6.26827	17.9942	7.31E-53	2.60E-50	109.55
KMO	1.93071	-1.8553	17.9923	7.45E-53	2.63E-50	109.532
FOXP3	1.77085	0.64285	17.9913	7.52E-53	2.64E-50	109.522
DOCK10	1.92417	-0.0753	17.9852	7.99E-53	2.78E-50	109.463
CASS4	2.08644	-0.8614	17.965	9.73E-53	3.36E-50	109.267
P2RY10	2.62386	-0.6801	17.9539	1.08E-52	3.72E-50	109.16
LILRB4	2.34155	1.43958	17.9464	1.17E-52	3.98E-50	109.087
ICAM3	2.33502	-0.7063	17.9185	1.53E-52	5.20E-50	108.816
CD48	2.19729	1.63707	17.8855	2.11E-52	7.09E-50	108.496
VCAM1	2.43128	1.88652	17.8833	2.16E-52	7.19E-50	108.475
IRF8	2.202	1.09418	17.8697	2.47E-52	8.16E-50	108.344
SIGLEC7	2.16	-0.4413	17.847	3.08E-52	1.01E-49	108.124
C1QB	2.21887	6.04042	17.8361	3.42E-52	1.12E-49	108.018
GVINP1	2.19226	-1.7221	17.8212	3.96E-52	1.28E-49	107.874
MS4A7	2.1511	1.65727	17.8214	3.95E-52	1.28E-49	107.875
AC09055!	2.11234	-1.4864	17.8104	4.40E-52	1.41E-49	107.769
ETS1	1.7532	3.04999	17.8083	4.49E-52	1.43E-49	107.749
ALOX5AP	2.05222	2.99887	17.7422	8.56E-52	2.70E-49	107.108
CRISPLD2	1.82389	2.35181	17.7375	8.96E-52	2.81E-49	107.062
WDFY4	2.25416	-0.8504	17.7322	9.43E-52	2.94E-49	107.011
TSPAN4	1.52456	1.85302	17.721	1.05E-51	3.27E-49	106.902
GPRIN3	1.80971	-0.6914	17.7112	1.16E-51	3.57E-49	106.808
CNRIP1	1.53619	0.30518	17.7041	1.24E-51	3.80E-49	106.739
TRAC	2.11435	2.95106	17.6993	1.30E-51	3.97E-49	106.692
LY86	2.07891	1.45532	17.6983	1.31E-51	3.98E-49	106.683
OSCAR	1.86025	1.23014	17.6829	1.53E-51	4.60E-49	106.533
ITGAL	2.10371	1.30873	17.6736	1.67E-51	5.01E-49	106.444
CD300C	2.08084	-0.0034	17.6549	2.01E-51	5.98E-49	106.262
PECAM1	1.58224	3.62639	17.6339	2.46E-51	7.30E-49	106.06
CCRL2	1.83562	-0.14	17.6303	2.55E-51	7.52E-49	106.025
FMOD	1.96539	3.74418	17.6092	3.13E-51	9.19E-49	105.82
C5AR1	1.80479	2.46522	17.5945	3.61E-51	1.05E-48	105.678
RUBCNL	1.9939	-1.0447	17.5914	3.72E-51	1.08E-48	105.648
LILRB3	1.7863	-0.5903	17.5825	4.06E-51	1.17E-48	105.562
CD2	2.16861	2.36085	17.5802	4.15E-51	1.19E-48	105.54
FBN1	2.1868	2.11561	17.5363	6.37E-51	1.80E-48	105.115
FPR1	2.34387	1.41558	17.5354	6.43E-51	1.81E-48	105.106
RGS18	2.12045	-1.2008	17.5315	6.68E-51	1.87E-48	105.068
SLAMF6	2.41255	0.45768	17.5235	7.22E-51	2.01E-48	104.991
IKZF1	2.05273	0.19438	17.5057	8.59E-51	2.38E-48	104.818
GASK1B	1.93027	1.3994	17.4828	1.07E-50	2.96E-48	104.597
TFEC	2.18307	-0.9889	17.4506	1.47E-50	4.01E-48	104.286
FGD2	1.83896	-0.5012	17.4497	1.48E-50	4.02E-48	104.277
GNG2	1.72872	0.45474	17.4372	1.67E-50	4.49E-48	104.156
TBXAS1	1.66166	0.92146	17.4158	2.06E-50	5.51E-48	103.949
GIMAP8	1.76584	0.73888	17.4119	2.14E-50	5.70E-48	103.911
SPN	2.10278	0.07475	17.4076	2.23E-50	5.91E-48	103.87
HLA-DOA	2.26569	2.38092	17.4054	2.28E-50	6.01E-48	103.849
RHOH	2.0206	-0.3717	17.4002	2.40E-50	6.29E-48	103.798
CCR8	2.47424	#NAME?	17.4201	2.45E-50	6.40E-48	103.778
DOK3	1.5734	0.85738	17.3956	2.51E-50	6.51E-48	103.755
TESPA1	1.97877	-1.5529	17.3911	2.62E-50	6.77E-48	103.711
RASSF2	1.9326	1.47685	17.3813	2.88E-50	7.41E-48	103.616
TMEM150	2.19413	-0.4407	17.3708	3.19E-50	8.17E-48	103.515
MSR1	2.38182	1.42988	17.3461	4.06E-50	1.03E-47	103.276
ACTA2	1.94574	5.22849	17.3383	4.38E-50	1.11E-47	103.2
EPSTI1	1.92442	1.56868	17.3188	5.30E-50	1.34E-47	103.012
TMEM176	1.9784	4.65361	17.2905	6.98E-50	1.75E-47	102.738
TRBV20-1	2.43587	#NAME?	17.3148	7.86E-50	1.97E-47	102.623
C16orf54	2.22573	-0.0855	17.2641	9.01E-50	2.24E-47	102.484
CD300A	1.70328	1.92365	17.2258	1.31E-49	3.21E-47	102.114
RASGRP4	1.74983	-1.4532	17.2221	1.36E-49	3.32E-47	102.078
ITGAM	2.10006	1.21795	17.2166	1.43E-49	3.48E-47	102.025
TIMP2	1.7323	4.98801	17.2031	1.63E-49	3.95E-47	101.894
CLEC4A	1.86004	1.08553	17.1974	1.72E-49	4.16E-47	101.84
GPR34	2.2077	0.6428	17.193	1.80E-49	4.33E-47	101.797
JAML	1.9359	0.03948	17.1782	2.08E-49	4.97E-47	101.654
THY1	1.75982	3.64645	17.1752	2.14E-49	5.08E-47	101.625
CTSK	2.07807	4.9417	17.1217	3.60E-49	8.46E-47	101.108
CD33	1.8926	-1.3299	17.1164	3.79E-49	8.87E-47	101.058
CXCR6	2.10816	0.11199	17.1077	4.12E-49	9.61E-47	100.974
CD52	1.99839	3.91383	17.0932	4.75E-49	1.10E-46	100.834
PIK3R5	1.88837	0.01511	17.0905	4.87E-49	1.13E-46	100.808
KCNMB1	1.97496	-1.2673	17.0196	9.70E-49	2.23E-46	100.124
SLA2	1.95949	-0.3167	17.0111	1.05E-48	2.41E-46	100.042
CYP1B1	2.41758	2.38674	17.0005	1.17E-48	2.65E-46	99.9398
RASSF4	1.61115	1.50146	17.0006	1.17E-48	2.65E-46	99.9407
CEACAM:	1.94743	-0.9446	16.9879	1.32E-48	2.99E-46	99.8187
P2RY8	2.23355	-0.2506	16.9817	1.40E-48	3.16E-46	99.759
NCF1	1.88203	-0.2754	16.964	1.67E-48	3.74E-46	99.5878
P2RY13	2.24755	-0.2927	16.9437	2.03E-48	4.53E-46	99.392
VSIG4	2.50068	2.82711	16.9266	2.39E-48	5.30E-46	99.2275
GPR84	1.76193	-0.3296	16.9269	2.39E-48	5.30E-46	99.2302
IL7R	2.3234	2.28608	16.9011	3.07E-48	6.77E-46	98.9818
FCGR1B	1.98915	-2.6341	16.8887	3.46E-48	7.60E-46	98.8622
TMEM119	2.30606	2.17544	16.8695	4.16E-48	9.11E-46	98.678
BICC1	2.10858	0.49162	16.8593	4.60E-48	1.00E-45	98.5793
GIMAP2	1.80787	1.28193	16.854	4.84E-48	1.05E-45	98.5286
LILRA6	1.9938	-1.0926	16.8429	5.39E-48	1.17E-45	98.4218
CD69	2.14643	0.54227	16.8242	6.46E-48	1.39E-45	98.242
NNMT	1.89594	4.19049	16.8067	7.66E-48	1.63E-45	98.0728
GZMK	2.7011	#NAME?	16.8102	8.43E-48	1.79E-45	97.9783
TRAF3IP3	1.79526	-0.528	16.7939	8.67E-48	1.83E-45	97.9497
SLAMF1	2.19959	-0.9199	16.7862	9.34E-48	1.97E-45	97.8756
CXCR3	2.26949	0.12241	16.7684	1.11E-47	2.33E-45	97.7049
CD247	1.77793	0.19	16.7445	1.40E-47	2.92E-45	97.4744
TRBC1	2.32551	#NAME?	16.7069	2.60E-47	5.42E-45	96.8604
CD180	2.18136	-0.3832	16.6691	2.90E-47	6.02E-45	96.7496
GIMAP5	2.07615	-2.6375	16.6665	2.98E-47	6.15E-45	96.7247
CD96	1.71785	0.15061	16.6619	3.11E-47	6.40E-45	96.6801
C19orf38	1.62876	-0.083	16.6443	3.69E-47	7.57E-45	96.5108
TRBV6-5	2.40374	#NAME?	16.8232	3.85E-47	7.86E-45	96.4866
PLA2G7	2.0327	1.94861	16.6311	4.20E-47	8.53E-45	96.3839
NEXN	1.73795	0.41257	16.6307	4.21E-47	8.53E-45	96.3806
TAGLN	1.8235	4.97002	16.6285	4.30E-47	8.68E-45	96.3594
RGS1	1.98546	3.62967	16.6265	4.39E-47	8.82E-45	96.3402
GPR132	1.7801	-0.0852	16.619	4.72E-47	9.45E-45	96.2677
LINC0109	1.79561	-0.2102	16.6138	4.96E-47	9.90E-45	96.2177
SP140	1.94288	-0.7051	16.6122	5.04E-47	1.00E-44	96.2029
GGTA1P	1.90118	-0.3973	16.5982	5.76E-47	1.14E-44	96.0686
CD3G	2.19469	-0.479	16.5613	8.24E-47	1.63E-44	95.7138
PYHIN1	2.13727	#NAME?	16.5635	8.59E-47	1.69E-44	95.6733
PIK3R6	1.67318	-0.9369	16.5486	9.31E-47	1.83E-44	95.5922
TRAV9-2	2.29168	#NAME?	16.8124	9.54E-47	1.86E-44	95.5936
CTLA4	2.02561	#NAME?	16.5179	1.33E-46	2.59E-44	95.2367
TRBV2	2.25204	#NAME?	16.744	1.34E-46	2.60E-44	95.2534
TRBC2	2.0741	2.64795	16.508	1.38E-46	2.67E-44	95.2018
FOLR2	2.26044	2.44438	16.4916	1.62E-46	3.11E-44	95.0444
AL590764	1.90672	#NAME?	16.5022	1.87E-46	3.59E-44	94.903
SH2D1A	2.33748	-0.4785	16.4602	2.19E-46	4.18E-44	94.7438
ITGB7	1.6529	-0.1296	16.453	2.35E-46	4.47E-44	94.6743
PPP1R16B	1.98913	0.07676	16.4514	2.38E-46	4.53E-44	94.6588
TRAV13-1	2.20682	#NAME?	16.8471	3.05E-46	5.76E-44	94.4526
HLA-DRB	2.27787	5.74226	16.4186	3.27E-46	6.15E-44	94.3439

ICI cluster-C1 VS ICI cluster-C3

Tag	logFC	AveExpr	t	P.Value	adj.P.Val	B
[illegible]	[illegible]	[illegible]	[illegible]	[illegible]	[illegible]	[illegible]

ICI cluster-C1 VS ICI cluster-C4

Tag	logFC	AveExpr	t	P.Value	adj.P.Val	B
[illegible]	[illegible]	[illegible]	[illegible]	[illegible]	[illegible]	[illegible]

ICI cluster-C2 VS ICI cluster-C3

Tag	logFC	AveExpr	t	P.Value	adj.P.Val	B
[illegible]	[illegible]	[illegible]	[illegible]	[illegible]	[illegible]	[illegible]

ICI cluster-C2 VS ICI cluster-C4

Tag	logFC	AveExpr	t	P.Value	adj.P.Val	B
[illegible]	[illegible]	[illegible]	[illegible]	[illegible]	[illegible]	[illegible]

ICI cluster-C3 VS ICI cluster-C4

Tag	logFC	AveExpr	t	P.Value	adj.P.Val	B
[illegible]	[illegible]	[illegible]	[illegible]	[illegible]	[illegible]	[illegible]

P2RX7	1.75954	-0.1923	16.4181	3.29E-46	6.16E-44	94.3394
RASGRF2	1.71142	-1	16.3722	5.12E-46	9.55E-44	93.8999
PLA2G5	2.10964	-1.3836	16.3493	6.39E-46	1.19E-43	93.6804
LYZ	2.70954	6.0279	16.3335	7.44E-46	1.38E-43	93.5288
GAB3	1.54319	-0.5539	16.3226	8.27E-46	1.53E-43	93.4242
FGR	1.65084	1.81536	16.3213	8.37E-46	1.54E-43	93.4119
CCR5AS	1.94646	#NAME?	16.3329	8.44E-46	1.55E-43	93.4049
INPP5D	1.74847	0.93616	16.3189	8.57E-46	1.57E-43	93.3889
CCDC69	1.66183	1.96086	16.3134	9.03E-46	1.65E-43	93.3364
TNFRSF9	2.05138	-0.8586	16.3115	9.20E-46	1.67E-43	93.318
TRBV28	2.09303	1.59486	16.2951	1.08E-45	1.94E-43	93.1605
BCL2A1	2.06279	2.45099	16.2914	1.12E-45	2.01E-43	93.125
MRVI1	1.66355	0.3965	16.2899	1.13E-45	2.03E-43	93.111
HK3	1.97093	1.0331	16.2867	1.17E-45	2.09E-43	93.0808
PDGFRB	1.61591	3.62924	16.2864	1.17E-45	2.09E-43	93.0775
CLEC4D	2.30111	#NAME?	16.3426	1.18E-45	2.09E-43	93.0772
C2	1.6435	2.1907	16.2844	1.20E-45	2.11E-43	93.0584
PTGER2	2.21286	-0.6778	16.2768	1.29E-45	2.27E-43	92.9853
ABCD2	2.23067	#NAME?	16.2609	1.59E-45	2.78E-43	92.7755
HNRNPA	1.87554	-0.2023	16.2279	2.06E-45	3.60E-43	92.5173
PARVG	1.67544	0.19029	16.2224	2.17E-45	3.78E-43	92.465
MMP19	1.92378	1.31955	16.1877	3.04E-45	5.26E-43	92.133
CXCR4	1.84897	4.62669	16.1818	3.21E-45	5.54E-43	92.0766
SIGLEC1	2.12784	0.63644	16.1747	3.44E-45	5.91E-43	92.0083
CD93	1.63506	2.64436	16.1604	3.95E-45	6.76E-43	91.872
DPEP2	1.82826	-0.6825	16.1595	3.98E-45	6.78E-43	91.8633
TRAT1	2.53746	#NAME?	16.1576	4.83E-45	8.20E-43	91.6732
HSD11B1	2.25525	1.17048	16.1386	4.87E-45	8.24E-43	91.6635
CIITA	2.01976	0.24518	16.1328	5.15E-45	8.68E-43	91.6082
CCL4	1.82557	1.71377	16.1319	5.20E-45	8.73E-43	91.5994
MRC1	2.51992	1.71671	16.1185	5.91E-45	9.85E-43	91.471
TRBV19	2.47291	#NAME?	16.1801	5.92E-45	9.85E-43	91.4774
AXL	1.63313	2.70601	16.116	6.06E-45	1.01E-42	91.4471
CD3D	2.0669	2.18371	16.0969	7.28E-45	1.20E-42	91.2648
TMIGD3	1.91838	-0.4706	16.08	8.57E-45	1.41E-42	91.1027
LILRA2	2.03346	-2.0804	16.0737	9.10E-45	1.48E-42	91.043
AC00603	2.08633	#NAME?	16.0794	1.15E-44	1.87E-42	90.8145
BHLHE22	1.97562	-1.3821	16.0471	1.18E-44	1.91E-42	90.7887
ARHGAP	1.60025	0.82045	16.0312	1.37E-44	2.21E-42	90.6371
LCP1	1.86964	4.55927	16.0219	1.50E-44	2.40E-42	90.5486
ICAM2	1.71589	0.50426	16.0102	1.68E-44	2.68E-42	90.4367
NAIP	1.90264	#NAME?	16.0112	1.86E-44	2.97E-42	90.3347
TRAV8-2	2.10082	#NAME?	16.2407	1.89E-44	3.01E-42	90.3425
MYO1G	1.75069	0.63533	15.9702	2.46E-44	3.91E-42	90.0548
LILRA1	1.84	-2.2655	15.9566	2.81E-44	4.44E-42	89.925
LINC0194	1.58479	-1.1414	15.956	2.82E-44	4.45E-42	89.919
ANGPTL2	1.7395	3.39476	15.9534	2.89E-44	4.55E-42	89.8942
PRKCB	1.89686	-0.3796	15.9496	3.00E-44	4.70E-42	89.8578
TRAV13-	2.32561	#NAME?	16.1134	3.63E-44	5.66E-42	89.6902
TLR4	2.09004	0.77616	15.9242	3.83E-44	5.96E-42	89.6156
TRG-AS1	1.84826	-1.9007	15.921	3.95E-44	6.12E-42	89.5858
GPR171	2.17932	-0.6177	15.92	3.99E-44	6.17E-42	89.5755
IL2RB	1.86864	1.66967	15.9192	4.02E-44	6.20E-42	89.5685
KLRB1	1.96732	0.28644	15.9091	4.43E-44	6.81E-42	89.4716
COL3A1	2.31745	8.02113	15.8953	5.06E-44	7.75E-42	89.3404
STARD13	1.5245	-0.1243	15.8816	5.77E-44	8.82E-42	89.21
COL8A1	2.17739	2.45955	15.8756	6.11E-44	9.31E-42	89.1528
FILIP1L	1.5712	2.11911	15.8634	6.87E-44	1.04E-41	89.0359
CLEC4E	2.34919	-0.4373	15.8534	7.56E-44	1.15E-41	88.9412
CD27	2.15185	1.74847	15.8506	7.76E-44	1.17E-41	88.9147
LAMA4	1.51992	1.86946	15.8501	7.80E-44	1.18E-41	88.9099
ABCA6	1.86458	-2.3622	15.8423	8.41E-44	1.26E-41	88.8355
PODN	2.14236	1.79755	15.8243	9.99E-44	1.50E-41	88.6641
RSPO3	2.4252	#NAME?	15.8237	1.06E-43	1.59E-41	88.6041
CLMP	2.15266	1.14438	15.7963	1.31E-43	1.94E-41	88.3971
AOC3	2.02483	2.03254	15.7943	1.33E-43	1.97E-41	88.3777
CD226	1.83103	-2.679	15.7943	1.33E-43	1.97E-41	88.3782
GPR174	2.44983	#NAME?	15.8254	1.46E-43	2.14E-41	88.2947
TMEM176	1.89673	3.02418	15.7824	1.49E-43	2.19E-41	88.2652
UBASH3A	2.12972	-1.3706	15.7728	1.64E-43	2.39E-41	88.1733
BGN	1.77593	7.29283	15.7696	1.69E-43	2.46E-41	88.1434
NCF1B	1.88979	-1.2128	15.769	1.70E-43	2.47E-41	88.1369
CILP	3.10654	0.11885	15.7546	1.95E-43	2.82E-41	87.9999
SPOCK2	2.01926	2.24035	15.754	1.96E-43	2.83E-41	87.995
COLEC12	2.12683	0.85761	15.7507	2.02E-43	2.91E-41	87.9635
LCK	1.80748	1.26416	15.7495	2.05E-43	2.94E-41	87.9513
AC14765	1.67103	-0.1137	15.7404	2.23E-43	3.20E-41	87.8656
S1PR4	1.77881	0.434	15.7399	2.25E-43	3.21E-41	87.86
TRAV12-	2.27309	#NAME?	15.9752	2.50E-43	3.56E-41	87.7838
FAP	2.10625	0.98151	15.7246	2.60E-43	3.68E-41	87.7151
IL4I1	1.93485	1.90683	15.7116	2.95E-43	4.16E-41	87.5909
LRRC32	1.53133	2.28815	15.7093	3.01E-43	4.23E-41	87.5697
TRAV8-6	2.19339	#NAME?	15.8895	3.31E-43	4.64E-41	87.4993
HLA-DOB	2.11157	4.06825	15.6899	3.63E-43	5.07E-41	87.3848
CRTAM	2.15753	-1.6245	15.6831	3.87E-43	5.40E-41	87.3206
CCN2	1.91839	5.43801	15.6771	4.10E-43	5.70E-41	87.2636
CD40LG	2.47343	#NAME?	15.6781	4.28E-43	5.94E-41	87.2208
SMIM25	1.77974	0.87643	15.6619	4.74E-43	6.56E-41	87.119
NCF1C	1.73683	0.12303	15.6612	4.77E-43	6.57E-41	87.1125
VGLL3	2.17452	-0.3804	15.6515	5.23E-43	7.17E-41	87.0203
DDR2	1.83481	0.78495	15.6367	6.03E-43	8.23E-41	86.8793
NFATC2	1.80245	0.0147	15.6319	6.31E-43	8.59E-41	86.8341
CD72	1.60578	0.18718	15.6242	6.80E-43	9.23E-41	86.7602
RASAL3	1.64541	0.7538	15.6154	7.39E-43	1.00E-40	86.677
SPATA20	1.90254	#NAME?	15.6423	8.32E-43	1.12E-40	86.5653
SYNE1	1.74458	-1.1804	15.5964	8.87E-43	1.19E-40	86.4965
TRAV8-4	2.13044	#NAME?	15.8065	1.04E-42	1.39E-40	86.3702
CCDC80	1.98548	2.88538	15.5717	1.12E-42	1.51E-40	86.2621
LINC0042	1.92087	#NAME?	15.539	1.62E-42	2.15E-40	85.9004
CD6	1.65015	0.40215	15.5229	1.79E-42	2.37E-40	85.7988
THBS1	1.81811	4.46666	15.5117	1.99E-42	2.63E-40	85.6932
STAB1	1.50781	2.00155	15.5018	2.19E-42	2.89E-40	85.599
FCMR	1.86065	1.1759	15.4938	2.37E-42	3.11E-40	85.5228
TNS3	1.58245	2.62153	15.4932	2.38E-42	3.12E-40	85.5168
ZNF521	1.96356	-0.3421	15.4889	2.48E-42	3.24E-40	85.4766
ALOX5	1.90199	2.29535	15.4853	2.56E-42	3.35E-40	85.4426
LINC0261	1.86744	-2.7833	15.4796	2.71E-42	3.53E-40	85.3882
LINC0135	1.97962	#NAME?	15.4844	3.35E-42	4.35E-40	85.1799
SIT1	2.05214	0.39789	15.4357	4.12E-42	5.33E-40	84.9726
FBLN5	1.69251	1.75698	15.4278	4.44E-42	5.73E-40	84.8971
ADGRE2	1.57345	0.05131	15.4183	4.86E-42	6.26E-40	84.8072
KCNA3	2.45823	#NAME?	15.4347	5.10E-42	6.55E-40	84.7626
TRBV5-1	2.33113	#NAME?	15.4933	5.26E-42	6.73E-40	84.7407
DNAJC5B	2.38623	#NAME?	15.4012	6.02E-42	7.69E-40	84.5962
TRAV12-	2.17209	#NAME?	15.7312	6.20E-42	7.90E-40	84.609
ATP10A	1.93771	-0.5511	15.392	6.25E-42	7.94E-40	84.5585
RP11-65	1.74685	#NAME?	15.5481	6.93E-42	8.77E-40	84.4769
TRBV18	2.13766	#NAME?	15.6093	7.18E-42	9.06E-40	84.4508
FTH1P22	2.24291	#NAME?	15.4541	8.02E-42	1.01E-39	84.3219
FN1	2.15213	6.2959	15.3594	8.53E-42	1.07E-39	84.2499
TRAV12-	2.2094	#NAME?	15.4533	1.06E-41	1.33E-39	84.0464
TRAV21	2.19616	#NAME?	15.5488	1.11E-41	1.38E-39	84.0195
ATP8B4	1.53659	-2.2092	15.3265	1.17E-41	1.45E-39	83.9385
CDH11	1.93163	2.12009	15.3167	1.28E-41	1.59E-39	83.8458
SPON1	2.17431	2.21573	15.3162	1.29E-41	1.59E-39	83.8415
TREM2	1.96711	2.97082	15.3151	1.30E-41	1.60E-39	83.8308
ICAM1	1.92234	4.3504	15.2829	1.77E-41	2.17E-39	83.5267
SLC15A3	1.62738	2.1288	15.2815	1.79E-41	2.20E-39	83.5129
FUT7	1.9328	#NAME?	15.2822	1.87E-41	2.28E-39	83.4717
OLR1	2.34831	1.63917	15.2726	1.95E-41	2.38E-39	83.429
FCGR1CP	2.00802	#NAME?	15.341	1.98E-41	2.41E-39	83.4242
UNC5C	1.96029	-1.5928	15.2645	2.11E-41	2.55E-39	83.3527
SNED1	1.60857	-0.304	15.2644	2.11E-41	2.55E-39	83.352
SLAMF7	2.22633	2.11114	15.2598	2.20E-41	2.66E-39	83.3081
AP00089	1.68713	-1.0952	15.2576	2.25E-41	2.71E-39	83.287
TRBV9	2.32703	#NAME?	15.4029	2.38E-41	2.86E-39	83.253
RARRES2	1.91291	4.52297	15.2482	2.46E-41	2.95E-39	83.1989
DPT	2.59018	2.41148	15.2171	3.31E-41	3.96E-39	82.9045
IL32	1.75995	3.50753	15.2081	3.60E-41	4.31E-39	82.8199
TRBV29-	2.30015	#NAME?	15.3016	4.15E-41	4.94E-39	82.6949
CCR6	2.19416	#NAME?	15.4561	4.45E-41	5.29E-39	82.6449
MMP2	1.97766	5.83253	15.1787	4.76E-41	5.63E-39	82.5425
ITGAX	1.64519	1.86251	15.1696	5.19E-41	6.13E-39	82.4566
FCN1	2.27414	-0.6358	15.152	6.14E-41	7.21E-39	82.2904
TRBV3-1	2.1563	#NAME?	15.2927	6.96E-41	8.15E-39	82.1869
OLFML3	1.72346	3.00484	15.1302	7.55E-41	8.80E-39	82.0849
PDLIM3	1.78401	1.16904	15.1135	8.85E-41	1.03E-38	81.9274
LAX1	2.28333	-0.1235	15.1048	9.61E-41	1.11E-38	81.8457
CCR7	2.01241	0.41009	15.1033	9.75E-41	1.13E-38	81.8312
GRAP	1.61535	-2.4223	15.0963	1.04E-40	1.20E-38	81.7654
TDRD6	1.84772	-2.9447	15.096	1.05E-40	1.20E-38	81.7621
SCML4	2.21662	#NAME?	15.0926	1.13E-40	1.30E-38	81.6838
CCL5	2.03853	4.25686	15.0813	1.20E-40	1.38E-38	81.6243
ITGBL1	2.48469	#NAME?	15.0776	1.31E-40	1.49E-38	81.5422
TOMM20	1.91143	#NAME?	15.2301	1.31E-40	1.50E-38	81.5585
CYSLTR1	1.93638	-1.3898	15.0708	1.33E-40	1.51E-38	81.5251
STAT4	1.52741	-0.3339	15.0516	1.59E-40	1.80E-38	81.3441
AC01189	1.94752	-2.3233	15.0473	1.66E-40	1.88E-38	81.3034
TBC1D10	1.64967	0.34696	15.0452	1.69E-40	1.91E-38	81.2835
GFPT2	1.92954	1.3331	15.0162	2.23E-40	2.51E-38	81.0111
THBS2	2.07665	4.49269	15.0059	2.46E-40	2.76E-38	80.9139
C1QTNF1	1.92824	2.2843	15.0054	2.47E-40	2.77E-38	80.9097
WNT2	2.42367	0.49923	15.0005	2.58E-40	2.89E-38	80.8634
TRAV8-3	2.05072	#NAME?	15.1661	2.64E-40	2.95E-38	80.8661
GEM	1.75523	2.69049	14.9816	3.09E-40	3.45E-38	80.6852
GLT8D2	1.76422	1.01675	14.9783	3.19E-40	3.55E-38	80.6547
PLCL2	1.57712	-0.2209	14.9584	3.85E-40	4.27E-38	80.4673
COL1A2	2.0897	6.97267	14.9558	3.95E-40	4.37E-38	80.4426
TIGIT	1.87593	-0.2436	14.9416	4.52E-40	4.97E-38	80.309
NKG7	1.88336	2.68759	14.9388	4.64E-40	5.10E-38	80.2826
LINC0096	2.20168	#NAME?	14.9913	4.77E-40	5.23E-38	80.2641
CD8A	1.88822	1.33443	14.9341	4.85E-40	5.30E-38	80.239
LTA	1.82831	-1.599	14.9223	5.42E-40	5.91E-38	80.1282
NID2	1.78948	0.65424	14.9128	5.93E-40	6.43E-38	80.0389
TRAV26-	1.91759	#NAME?	15.1315	7.49E-40	8.09E-38	79.8417
CHST11	1.6076	2.22007	14.8809	8.02E-40	8.64E-38	79.7389
CR1	2.35012	-2.5188	14.8701	8.89E-40	9.55E-38	79.6377
MRGPRF	1.77036	0.60661	14.8694	8.94E-40	9.59E-38	79.6313
FCRL6	2.00939	-2.0424	14.8656	9.27E-40	9.91E-38	79.5955
LILRA5	1.85893	0.24242	14.8628	9.52E-40	1.02E-37	79.5691
ZC3H12D	1.62644	-1.8205	14.8558	1.02E-39	1.08E-37	79.5031
GBP4	1.77426	2.33926	14.8429	1.15E-39	1.22E-37	79.3819
COL6A3	1.94418	4.61347	14.8312	1.28E-39	1.36E-37	79.2725
SELL	2.07181	1.68098	14.8208	1.42E-39	1.50E-37	79.1749
ENPP2	1.77316	1.96917	14.8168	1.47E-39	1.55E-37	79.1375
TRAV41	2.13597	#NAME?	15.0912	1.53E-39	1.61E-37	79.1409
ARHGAP	1.54991	1.04271	14.8116	1.54E-39	1.63E-37	79.0889
FGD5	1.63867	0.47155	14.8064	1.62E-39	1.70E-37	79.0402
AL365361	2.3105	-1.8407	14.8057	1.63E-39	1.71E-37	79.0338
TNFSF4	1.70172	-0.1159	14.7996	1.73E-39	1.81E-37	78.9766
GPC6	2.28476	0.34241	14.7949	1.81E-39	1.89E-37	78.9324
EBI3	1.7745	0.41404	14.7913	1.87E-39	1.95E-37	78.8983
CELF2	1.74087	1.04686	14.7899	1.90E-39	1.97E-37	78.8855
AL133371	1.83057	#NAME?	14.8987	2.04E-39	2.12E-37	78.829
JAK3	1.57895	1.46883	14.7801	2.08E-39	2.15E-37	78.7938
TRAV16	1.8952	#NAME?	15.0879	2.11E-39	2.18E-37	78.824
DOCK11	1.50504	0.83304	14.7783	2.11E-39	2.18E-37	78.777
AF127936	1.96337	#NAME?	14.8694	2.68E-39	2.75E-37	78.5605
PTGDR	1.89205	#NAME?	14.7605	2.74E-39	2.81E-37	78.5221
TRBV6-1	2.18458	#NAME?	14.9513	3.62E-39	3.71E-37	78.2777
ABI3BP	2.18539	-0.2199	14.7032	4.30E-39	4.39E-37	78.0735
TNFAIP6	1.9176	2.08029	14.6906	4.84E-39	4.94E-37	77.9558
LMOD1	1.87629	0.89413	14.6848	5.11E-39	5.20E-37	77.9014
APOL3	1.66971	1.61428	14.6771	5.50E-39	5.58E-37	77.8288
TRAV4	2.02718	#NAME?	14.8051	5.63E-39	5.70E-37	77.8255
AQP9	2.30444	0.73612	14.6697	5.89E-39	5.96E-37	77.7597
LINC0115	1.68716	#NAME?	14.9214	5.92E-39	5.97E-37	77.7937
RP11-556	1.84256	#NAME?	14.7684	6.18E-39	6.22E-37	77.7297
EMILIN1	1.88675	3.91527	14.659	6.52E-39	6.55E-37	77.6598
ANGPTL1	2.18585	#NAME?	14.6667	6.62E-39	6.64E-37	77.6465
ITGA11	2.26462	0.8559	14.6568	6.65E-39	6.66E-37	77.6394
AEBP1	1.85258	5.52018	14.6546	6.79E-39	6.79E-37	77.6188
PDCD1LG	1.83827	0.78862	14.6319	8.42E-39	8.38E-37	77.4062
POSTN	2.22426	5.18307	14.6101	1.03E-38	1.03E-36	77.203
RNASE2	1.93765	-0.3963	14.61	1.03E-38	1.03E-36	77.2018
ROR1	2.14374	#NAME?	14.6114	1.07E-38	1.06E-36	77.1723
TRBV7-9	2.24439	#NAME?	14.6184	1.42E-38	1.41E-36	76.8931
CLEC5A	1.85034	0.04703	14.5737	1.46E-38	1.44E-36	76.8623
GREM1	2.48137	1.28664	14.5717	1.48E-38	1.46E-36	76.8439
MXRA8	1.61998	3.58751	14.5604	1.65E-38	1.62E-36	76.7383
MEI1	1.95733	-0.4893	14.5361	2.07E-38	2.03E-36	76.512
PDGFRA	1.88947	1.58844	14.5345	2.10E-38	2.06E-36	76.4965
LILRB5	1.97694	-1.2408	14.5288	2.22E-38	2.17E-36	76.4438
PCDHGA	1.82526	#NAME?	14.5111	2.73E-38	2.66E-36	76.2376
AC00574	1.82912	0.97151	14.488	3.26E-38	3.17E-36	76.0634
LINC0161	2.57043	#NAME?	14.5008	3.42E-38	3.33E-36	76.0166
CCN1	1.64092	5.16421	14.4815	3.46E-38	3.36E-36	76.0023
AP00143	1.82091	#NAME?	14.5654	3.50E-38	3.39E-36	76.0057
BTLA	2.12292	#NAME?	14.4802	3.66E-38	3.53E-36	75.9494
TRAV17	2.06628	#NAME?	14.604	3.75E-38	3.61E-36	75.9446
CD300E	2.02116	-0.6351	14.4728	3.75E-38	3.61E-36	75.922
HEPH	1.78911	0.69309	14.4648	4.05E-38	3.89E-36	75.8466
PRF1	1.79205	1.74517	14.4527	4.54E-38	4.35E-36	75.7339
ADAMTS	1.94786	2.07017	14.4501	4.65E-38	4.45E-36	75.7102
ZNF831	2.33928	-3.3126	14.4439	4.93E-38	4.70E-36	75.6528
ADAMTS	1.94015	1.17524	14.438	5.21E-38	4.95E-36	75.5979
GJA5	1.95948	1.86052	14.4147	6.48E-38	6.14E-36	75.3806
CLEC10A	2.11181	0.10183	14.4117	6.66E-38	6.30E-36	75.353
COL5A2	1.81006	4.70605	14.4029	7.24E-38	6.83E-36	75.2711
CD209	2.04015	0.18431	14.4018	7.32E-38	6.89E-36	75.2601
P2RX1	1.97083	-0.9762	14.4016	7.32E-38	6.89E-36	75.259
LINC0168	1.91457	#NAME?	14.5305	7.70E-38	7.23E-36	75.2316
PABPC5	1.89618	#NAME?	14.4082	7.81E-38	7.32E-36	75.198
TRGV3	1.86794	#NAME?	14.6168	8.22E-38	7.70E-36	75.1812
ISLR	1.90587	4.10226	14.3868	8.41E-38	7.86E-36	75.1213
MSRB3	1.66227	1.50263	14.3799	8.98E-38	8.38E-36	75.0564
ADAMDE	2.56507	1.40126	14.3776	9.18E-38	8.55E-36	75.0351
SIRPG	2.06578	-0.3368	14.374	9.49E-38	8.82E-36	75.0022
CLEC6A	2.38168	#NAME?	14.5547	1.01E-37	9.34E-36	74.9734
CCL2	1.86825	3.63881	14.3483	1.21E-37	1.12E-35	74.763
TRBV5-4	2.16344	#NAME?	14.5325	1.23E-37	1.14E-35	74.775
TRAV29D	2.09982	#NAME?	14.528	1.35E-37	1.24E-35	74.6863
TGFB3	1.50206	2.27945	14.3324	1.40E-37	1.29E-35	74.6156
PDGFRL	1.75051	1.39253	14.3319	1.41E-37	1.29E-35	74.6109
USP30-A	1.82637	#NAME?	14.3318	1.47E-37	1.34E-35	74.5699
PEAK3	1.69005	#NAME?	14.3224	1.60E-37	1.46E-35	74.4828
GXYLT2	1.81527	0.81447	14.2738	2.42E-37	2.20E-35	74.0714
RCAN2	1.69582	0.84516	14.2728	2.45E-37	2.22E-35	74.0614
MOXD1	2.33997	2.0845	14.2608	2.74E-37	2.48E-35	73.9507
A2M	1.77685	5.68061	14.2452	3.17E-37	2.86E-35	73.8059
ST6GALN	1.61272	-0.0989	14.2344	3.50E-37	3.15E-35	73.7057
FNDC1	2.48668	1.46356	14.2341	3.51E-37	3.15E-35	73.703
TRAV19	2.14352	#NAME?	14.3404	3.81E-37	3.41E-35	73.642
IQGAP2	1.74081	0.18969	14.1954	5.04E-37	4.50E-35	73.3442
DAB2	1.54101	2.44007	14.1898	5.31E-37	4.73E-35	73.2923
PTAFR	1.6173	2.09067	14.1822	5.70E-37	5.07E-35	73.222
REM1	1.65349	-1.3188	14.1786	5.90E-37	5.24E-35	73.1881
MEOX2	2.26193	#NAME?	14.1751	6.34E-37	5.62E-35	73.1179
SAMD3	2.00947	-3.458	14.1333	9.01E-37	7.92E-35	72.769
MAGI2-A	1.62546	-0.9782	14.1227	9.94E-37	8.73E-35	72.6707
AC01591	1.9141	#NAME?	14.1617	1.03E-36	9.05E-35	72.6411
PTGDS	2.29784	3.20081	14.0958	1.28E-36	1.11E-34	72.4224
PCOLCE	1.54085	3.86522	14.0891	1.36E-36	1.18E-34	72.3604
VCAN	1.85665	3.3319	14.0863	1.40E-36	1.21E-34	72.3346
IL9R	1.83477	#NAME?	14.0916	1.44E-36	1.25E-34	72.3075
DLC1	1.60867	0.62959	14.0828	1.44E-36	1.25E-34	72.3025
NAPSB	1.84247	1.50246	14.0754	1.54E-36	1.34E-34	72.2335

AL031595	1.58124	#NAME?	8.1729	1.02E-13	9.99E-12	20.6969
CCL21	1.87059	5.40629	8.15154	1.03E-13	1.00E-11	20.6744
TRAV2	1.69415	#NAME?	8.1678	1.05E-13	1.02E-11	20.6707
CD22	1.86949	-0.145	8.13664	1.12E-13	1.08E-11	20.5895
CD27	1.57505	2.82339	8.13706	1.12E-13	1.08E-11	20.5919
FLT3	1.68551	-2.0965	8.12821	1.18E-13	1.12E-11	20.5415
AC00636	1.68478	#NAME?	8.15368	1.25E-13	1.19E-11	20.5105
IL12B	1.84147	#NAME?	8.11172	1.37E-13	1.30E-11	20.4013
LINC0268	1.50205	#NAME?	8.18209	1.52E-13	1.43E-11	20.3452
CCL5	1.54912	5.25955	8.05445	1.81E-13	1.68E-11	20.1222
TRAV4	1.633	#NAME?	8.07345	1.81E-13	1.68E-11	20.1382
CLEC4D	1.62681	-1.9556	8.0085	2.36E-13	2.17E-11	19.8619
LINC0148	1.62085	-1.3616	7.99704	2.52E-13	2.31E-11	19.797
TRAV29D	1.60789	#NAME?	7.98157	3.07E-13	2.80E-11	19.6162
IL27	1.50424	#NAME?	7.9104	4.26E-13	3.83E-11	19.2874
XCR1	1.80113	-1.4796	7.90146	4.37E-13	3.91E-11	19.2577
SPOCK2	1.69103	3.19069	7.89498	4.54E-13	4.05E-11	19.2213
CLIC5	2.16718	-0.4097	7.88839	4.71E-13	4.20E-11	19.1842
KLRC1	1.83441	#NAME?	7.88817	4.84E-13	4.30E-11	19.1625
AL02365	1.51938	#NAME?	7.88817	5.68E-13	5.04E-11	19.0285
TRGC1	1.65446	-2.7599	7.8497	5.89E-13	5.20E-11	18.9669
TRDC	1.6673	0.22119	7.83973	6.23E-13	5.48E-11	18.9109
TRGV10	1.76161	#NAME?	7.85667	6.61E-13	5.78E-11	18.8819
MS4A1	2.58029	0.08037	7.82663	6.72E-13	5.87E-11	18.8374
GBP5	1.50116	2.45296	7.82623	6.74E-13	5.87E-11	18.8352
ADGRF4F	1.71735	-2.1213	7.82323	6.85E-13	5.95E-11	18.8184
TRAV20	1.71481	#NAME?	7.85384	7.29E-13	6.30E-11	18.7943
NUGGC	1.88037	-1.0104	7.77803	8.88E-13	7.59E-11	18.5654
CXorf65	1.75873	#NAME?	7.74238	1.11E-12	9.47E-11	18.3475
TCL1A	2.71294	#NAME?	7.74001	1.16E-12	9.79E-11	18.3153
LINC0040	1.9118	#NAME?	7.72543	1.29E-12	1.08E-10	18.2089
TLR10	1.94788	-0.7778	7.70942	1.31E-12	1.10E-10	18.1827
C9orf139	1.74332	#NAME?	7.6691	1.69E-12	1.41E-10	17.9404
ANKRD55	1.70569	#NAME?	7.65708	1.94E-12	1.60E-10	17.8122
CD79B	1.55807	1.68748	7.63346	2.02E-12	1.67E-10	17.7607
TRBV11-	1.71265	#NAME?	7.641	2.03E-12	1.67E-10	17.7668
P2RX1	1.59064	-0.0323	7.62844	2.08E-12	1.71E-10	17.7329
ACOXL	1.82023	-2.2369	7.61578	2.24E-12	1.82E-10	17.6628
KCNIP1	1.5178	#NAME?	7.59948	2.57E-12	2.08E-10	17.5344
AC00449	1.78115	#NAME?	7.57603	2.93E-12	2.36E-10	17.4086
DCSTAMP	1.74506	-1.3143	7.53176	3.59E-12	2.88E-10	17.1987
IRF4	1.59797	0.97188	7.51207	4.02E-12	3.21E-10	17.0903
PKD2L1	1.84706	-2.0788	7.48932	4.56E-12	3.63E-10	16.9652
CHIT1	2.67717	1.3669	7.48437	4.69E-12	3.72E-10	16.938
TRBV30	1.77022	#NAME?	7.52972	5.07E-12	4.01E-10	16.9074
KLRC4-KI	1.68266	#NAME?	7.47768	5.32E-12	4.20E-10	16.8325
TRGV4	1.63883	#NAME?	7.48765	5.65E-12	4.45E-10	16.7928
AC02636	2.22547	#NAME?	7.48103	5.73E-12	4.50E-10	16.7821
STAP1	1.62886	-0.3974	7.43771	6.10E-12	4.78E-10	16.6821
AC01591	1.5398	#NAME?	7.4385	6.20E-12	4.85E-10	16.6708
TRAV12-	1.65886	#NAME?	7.43797	6.36E-12	4.95E-10	16.6521
FCN1	1.58129	0.52296	7.41416	6.96E-12	5.40E-10	16.5533
AC00810	1.62876	-0.6355	7.38851	8.03E-12	6.17E-10	16.4131
OLR1	1.54679	2.85935	7.38038	8.41E-12	6.43E-10	16.3687
AL60680	2.06187	-3.4474	7.364	9.21E-12	7.02E-10	16.2794
AC00756	1.75626	-1.9196	7.35312	9.79E-12	7.45E-10	16.2201
TRGC2	1.80941	-0.5937	7.34122	1.05E-11	7.94E-10	16.1553
CCL23	1.85391	-0.5107	7.32891	1.12E-11	8.48E-10	16.0884
TNFRSF1	2.16845	-2.3818	7.31999	1.18E-11	8.88E-10	16.0399
LINC0244	2.10164	#NAME?	7.31926	1.21E-11	9.07E-10	16.0215
KLRK1	1.52295	-2.5679	7.31333	1.22E-11	9.17E-10	16.0037
TRBV10-	1.57666	#NAME?	7.32411	1.30E-11	9.71E-10	15.969
MARCO	2.11655	3.46938	7.29264	1.37E-11	1.02E-09	15.8914
TIMD4	1.86508	#NAME?	7.29411	1.47E-11	1.09E-09	15.8416
CXCR5	1.95557	#NAME?	7.30424	1.52E-11	1.12E-09	15.8198
NCR1	1.89754	#NAME?	7.26352	1.68E-11	1.23E-09	15.7056
KCNJ10	2.10803	#NAME?	7.25386	1.73E-11	1.27E-09	15.6675
FFAR4	1.83633	-2.3067	7.23573	1.88E-11	1.37E-09	15.5833
BIRC3	1.52168	2.76459	7.22216	2.03E-11	1.47E-09	15.51
ART4	2.0798	#NAME?	7.2081	2.28E-11	1.65E-09	15.4035
CR1L	1.54268	#NAME?	7.20676	2.29E-11	1.66E-09	15.4
LINC0238	2.00757	-1.1603	7.17877	2.57E-11	1.86E-09	15.2761
TRBV12-	1.55492	#NAME?	7.22005	2.74E-11	1.97E-09	15.2737
FCRL1	2.41104	#NAME?	7.16629	2.86E-11	2.05E-09	15.1829
CNR2	2.05885	-3.5394	7.14657	3.07E-11	2.20E-09	15.103
LINC0121	1.88467	-1.7543	7.13732	3.23E-11	2.31E-09	15.0533
TRBV4-2	1.68182	#NAME?	7.14084	3.29E-11	2.34E-09	15.0466
SSTR3	1.60687	#NAME?	7.13561	3.39E-11	2.40E-09	15.0186
FAM30A	1.96363	#NAME?	7.12622	3.50E-11	2.48E-09	14.9812
THEMIS	1.6681	-0.4003	7.07635	4.52E-11	3.15E-09	14.7269
AC09358	1.57229	#NAME?	7.10011	4.71E-11	3.28E-09	14.725
AC00727	1.79376	#NAME?	7.08691	5.27E-11	3.66E-09	14.6195
PLD4	1.50542	-1.2329	7.03497	5.67E-11	3.92E-09	14.5061
FBP1	1.63478	4.11989	7.00881	6.54E-11	4.50E-09	14.3669
TRAV5	1.66986	#NAME?	7.03079	6.99E-11	4.78E-09	14.3519
ATP8A1	1.52352	-0.0214	6.99292	7.13E-11	4.86E-09	14.2825
AF165138	1.51235	#NAME?	7.01152	7.75E-11	5.26E-09	14.2512
AC13364	1.66286	#NAME?	6.94159	1.01E-10	6.79E-09	13.9618
RTP5	1.87322	#NAME?	6.96592	1.09E-10	7.32E-09	13.9397
TRAV41	1.56866	#NAME?	6.92096	1.13E-10	7.56E-09	13.8577
PTGDS	1.54564	4.38597	6.90374	1.16E-10	7.72E-09	13.8105
AC24678	2.08502	#NAME?	6.92353	1.29E-10	8.54E-09	13.7464
JCHAIN	2.08759	7.624	6.87904	1.32E-10	8.75E-09	13.6803
ZNF683	1.80536	-0.3112	6.85873	1.48E-10	9.70E-09	13.5735
LINC0227	1.50653	#NAME?	6.83358	1.77E-10	1.15E-08	13.4111
XXYLT1-A	1.52061	#NAME?	6.83615	1.87E-10	1.22E-08	13.3694
TSHR	1.70953	-3.1181	6.80388	1.98E-10	1.28E-08	13.2858
APOC2	1.51409	#NAME?	6.80147	2.18E-10	1.41E-08	13.2237
ITGB2-AS	1.51804	-0.2256	6.77213	2.35E-10	1.51E-08	13.1198
COL4A4	1.91923	-0.5514	6.76359	2.46E-10	1.57E-08	13.0753
TENT5C	1.50551	2.81244	6.75494	2.58E-10	1.64E-08	13.0302
BIRC7	1.79064	#NAME?	6.73994	2.84E-10	1.79E-08	12.9428
TTC24	1.62748	#NAME?	6.7087	3.35E-10	2.09E-08	12.7765
ACSL6	1.71628	-3.8652	6.69904	3.47E-10	2.17E-08	12.7394
B3GAT1	1.59066	-4.1354	6.65644	4.36E-10	2.69E-08	12.5188
TRAV10	1.60759	#NAME?	6.70886	4.54E-10	2.80E-08	12.5717
IDO2	1.86188	-3.978	6.64455	4.64E-10	2.85E-08	12.4573
HLA-DOB	1.59371	3.5555	6.62543	5.14E-10	3.15E-08	12.3586
TMEM163	1.51188	-0.1455	6.61512	5.43E-10	3.31E-08	12.3055
OR2I1P	1.72353	2.68356	6.59067	6.18E-10	3.73E-08	12.1796
MAL	1.73745	1.08521	6.58596	6.33E-10	3.81E-08	12.1554
CCL19	2.01712	4.55244	6.55309	7.53E-10	4.50E-08	11.9867
AC03423	1.58839	#NAME?	6.58473	7.77E-10	4.64E-08	12.0144
SPIB	2.56506	-0.2436	6.53735	8.18E-10	4.86E-08	11.9061
CLEC9A	1.58353	#NAME?	6.54519	8.31E-10	4.93E-08	11.9154
CD19	1.81025	0.23629	6.53077	8.47E-10	5.00E-08	11.8724
ADGRE1	1.62757	-0.8698	6.53096	8.46E-10	5.00E-08	11.8734
NIBAN3	1.85846	-3.107	6.51281	9.31E-10	5.49E-08	11.7806
IFNG	1.97231	#NAME?	6.48774	1.09E-09	6.39E-08	11.6383
MMP2-AS	1.89554	#NAME?	6.49047	1.09E-09	6.39E-08	11.6449
UBD	1.77223	3.27914	6.47351	1.14E-09	6.68E-08	11.5802
CD1C	1.50927	0.33926	6.46778	1.18E-09	6.87E-08	11.5511
MIR3945H	1.53721	#NAME?	6.46366	1.22E-09	7.09E-08	11.5231
GPR25	1.84739	#NAME?	6.4378	1.42E-09	8.16E-08	11.3851
CLEC4C	1.84061	#NAME?	6.47888	1.43E-09	8.23E-08	11.4391
SELP	1.59682	0.76745	6.40331	1.65E-09	9.39E-08	11.2241
AC00468	1.77568	#NAME?	6.36691	2.04E-09	1.15E-07	11.0277
HLA-DOA	2.17496	3.9703	6.35777	2.09E-09	1.17E-07	10.9942
FCRL5	1.99739	0.1673	6.34295	2.26E-09	1.26E-07	10.9196
M1AP	1.68447	-2.2312	6.31763	2.57E-09	1.42E-07	10.7924
CXCL10	1.66072	5.27311	6.26961	3.29E-09	1.80E-07	10.552
SIGLEC11	1.56181	-2.7426	6.23619	3.91E-09	2.11E-07	10.3854
TMEM156	1.63756	0.43872	6.21759	4.30E-09	2.31E-07	10.2928
GPA33	1.7109	#NAME?	6.21472	4.52E-09	2.42E-07	10.263
HLA-DRB	1.80231	4.19895	6.20032	4.70E-09	2.50E-07	10.207
TRBV4-1	1.57226	#NAME?	6.18945	5.47E-09	2.89E-07	10.102
LRRK2	1.8611	0.75322	6.16376	5.66E-09	2.97E-07	10.026
SIRPG-AS	1.5357	#NAME?	6.19444	5.85E-09	3.07E-07	10.0647
LINC0239	1.59849	#NAME?	6.14642	6.40E-09	3.32E-07	9.92132
LINC0257	1.55395	#NAME?	6.12801	6.87E-09	3.52E-07	9.84496
BLK	1.84822	-1.4996	6.12509	6.89E-09	3.53E-07	9.83518
SIGLEC14	1.72268	#NAME?	6.12478	7.14E-09	3.65E-07	9.81025
TRBV12-	1.53237	#NAME?	6.08228	9.16E-09	4.58E-07	9.58363
RUFY4	1.76381	-2.354	6.06305	9.44E-09	4.72E-07	9.53055
TNFRSF1	1.85788	1.37661	6.06169	9.50E-09	4.74E-07	9.52392
AC01223	1.51363	#NAME?	6.0555	9.91E-09	4.92E-07	9.48954
CXCL11	1.76606	1.94518	6.05094	1.00E-08	4.98E-07	9.47131
DNAH8	1.51061	#NAME?	6.06786	1.01E-08	5.00E-07	9.51535
AC00913	1.59437	#NAME?	6.0373	1.15E-08	5.65E-07	9.38113
HLA-DPB	1.62857	-0.2925	6.01419	1.21E-08	5.90E-07	9.29205
LINC0236	1.55989	-0.1963	5.96878	1.52E-08	7.28E-07	9.07142
CCL14	1.79813	-2.6174	5.95573	1.62E-08	7.70E-07	9.0082
RP3-4921	1.65492	#NAME?	6.01363	1.74E-08	8.24E-07	9.09213
AC05574	1.55174	#NAME?	5.98015	1.80E-08	8.53E-07	9.0079
HLA-DPA	1.5131	#NAME?	5.92956	2.03E-08	9.51E-07	8.83572
ITLN1	1.75401	#NAME?	5.9303	2.04E-08	9.57E-07	8.83008
COL4A3	1.94746	-1.639	5.89076	2.24E-08	1.04E-06	8.69486
CD1B	1.68161	#NAME?	5.89113	2.25E-08	1.05E-06	8.6936
PNOC	1.62977	-0.4036	5.86424	2.55E-08	1.17E-06	8.56756
SCN7A	1.80051	-0.9496	5.8619	2.58E-08	1.19E-06	8.55636
BCORP1	1.61751	#NAME?	5.91819	3.00E-08	1.37E-06	8.50575
FCRL4	1.87055	#NAME?	5.83864	3.07E-08	1.40E-06	8.42374
CD1E	1.58543	-0.9727	5.82399	3.11E-08	1.41E-06	8.37506
ACOD1	1.56523	#NAME?	5.84359	3.34E-08	1.51E-06	8.38837
SYT15	1.56996	-4.562	5.80461	3.42E-08	1.54E-06	8.2827
AC10688	1.55209	#NAME?	5.79671	3.62E-08	1.63E-06	8.23521
AL12212	1.69223	#NAME?	5.77373	4.34E-08	1.94E-06	8.11061
FCRL2	1.68175	#NAME?	5.75407	4.51E-08	2.01E-06	8.03635
IGLL5	1.94825	5.85769	5.744	4.61E-08	2.05E-06	7.99509
IGHV3-5	2.04006	4.62059	5.73385	4.84E-08	2.14E-06	7.94714
MIR4537	1.76598	#NAME?	5.80578	4.95E-08	2.19E-06	8.08227
BCL2L14	1.53385	-1.7958	5.72846	4.97E-08	2.20E-06	7.92166
LMO3	2.41953	-0.7211	5.72332	5.09E-08	2.25E-06	7.89741
SLC46A2	1.90147	-1.9541	5.7211	5.15E-08	2.27E-06	7.88694
IGLV8-61	2.71161	#NAME?	5.69929	5.78E-08	2.52E-06	7.77732
AL51230	1.58312	#NAME?	5.70308	5.78E-08	2.52E-06	7.79657
TBC1D27	1.97217	#NAME?	5.70206	5.86E-08	2.55E-06	7.78457
CD79A	1.69617	4.05062	5.68981	6.00E-08	2.60E-06	7.7396
IL22RA2	1.7064	-1.5831	5.66859	6.65E-08	2.87E-06	7.64001
CHRDL1	1.9601	0.65166	5.6591	6.96E-08	2.99E-06	7.59553
MZB1	1.72878	4.23396	5.65186	7.21E-08	3.09E-06	7.56164
IGLV6-57	2.03668	6.34483	5.64928	7.30E-08	3.12E-06	7.54956
GDF10	1.87653	#NAME?	5.63195	8.14E-08	3.46E-06	7.459
IGHM	2.0783	7.69815	5.62006	8.41E-08	3.57E-06	7.4131
IGLV2-23	1.95177	7.67533	5.61479	8.62E-08	3.65E-06	7.38857
SULT1C2	1.6254	-2.0978	5.6128	8.71E-08	3.68E-06	7.37927
PTHLH	-2.1182	4.16448	-5.5559	1.14E-07	4.75E-06	7.11533
NRIR	1.57322	#NAME?	5.53879	1.30E-07	5.36E-06	7.01413
CAPN8	2.43679	#NAME?	5.52215	1.36E-07	5.55E-06	6.95853
ANKRD36	1.62471	#NAME?	5.51655	1.39E-07	5.69E-06	6.93275
AC24678	1.59017	#NAME?	5.54577	1.44E-07	5.86E-06	6.98588
ANO5	1.54487	#NAME?	5.49069	1.57E-07	6.39E-06	6.81401
IGLV2-14	1.90843	8.39984	5.479	1.65E-07	6.67E-06	6.76106
LINC0242	1.51129	#NAME?	5.4789	1.73E-07	6.97E-06	6.75805
EPHA10	1.78912	#NAME?	5.44956	1.91E-07	7.64E-06	6.62073
IGKV1-27	2.13363	5.66015	5.44199	1.97E-07	7.85E-06	6.59184
AGER	2.09777	2.43754	5.43869	2.00E-07	7.95E-06	6.57678
IGHV3-30	1.87558	7.0958	5.41708	2.21E-07	8.76E-06	6.47836
IGKV1D-	1.85706	#NAME?	5.41436	2.26E-07	8.90E-06	6.46575
IGLV1-51	1.92625	7.70346	5.40753	2.32E-07	9.10E-06	6.43499
TNR	1.62522	#NAME?	5.39922	2.54E-07	9.92E-06	6.37936
DTNB-AS	1.51728	#NAME?	5.35477	3.03E-07	1.17E-05	6.18547
KLRC2	1.65763	#NAME?	5.36	3.07E-07	1.18E-05	6.1974
HS3ST2	1.58171	1.00012	5.34582	3.09E-07	1.19E-05	6.1558
AL16241	1.76772	#NAME?	5.38173	3.39E-07	1.29E-05	6.28388
ZBTB16	1.88071	#NAME?	5.32291	3.49E-07	1.33E-05	6.05319
EXOC3L4	1.95049	-1.6034	5.31944	3.50E-07	1.33E-05	6.03712
IGLV1-40	1.87638	8.42345	5.30361	3.77E-07	1.42E-05	5.96609
IGKV3-20	1.80997	9.07107	5.2988	3.85E-07	1.45E-05	5.94454
ADH1B	2.80069	#NAME?	5.28433	4.15E-07	1.54E-05	5.88021
IGHG2	1.85088	9.50148	5.27625	4.28E-07	1.59E-05	5.84369
LRRK2-D	1.84722	-1.2191	5.24729	4.89E-07	1.80E-05	5.71459
LINC0178	1.56684	#NAME?	5.24151	5.27E-07	1.93E-05	5.68872
IGLC2	1.68965	9.68822	5.22269	5.48E-07	1.99E-05	5.60533
IGHV3-2	1.76603	8.00988	5.2072	5.88E-07	2.13E-05	5.53674
IGLC3	1.7816	8.88366	5.1993	6.10E-07	2.21E-05	5.5018
DNASE2B	1.79162	#NAME?	5.20372	6.31E-07	2.27E-05	5.5173
SLC5A9	1.52287	#NAME?	5.19136	6.37E-07	2.29E-05	5.46216
IGKJ5	1.59932	#NAME?	5.19778	6.48E-07	2.32E-05	5.49131
IGKV3-11	1.80751	8.45097	5.17911	6.69E-07	2.39E-05	5.41271
IGKV3-15	1.69227	7.33593	5.17818	6.72E-07	2.40E-05	5.40861
AC02065	1.54648	#NAME?	5.17057	7.09E-07	2.52E-05	5.37247
C7	2.15915	2.56529	5.16481	7.15E-07	2.53E-05	5.34971
FCER2	1.74355	#NAME?	5.16154	7.39E-07	2.61E-05	5.33864
IGKC	1.77796	10.7336	5.14151	7.95E-07	2.78E-05	5.24739
IGHV4-61	1.89222	#NAME?	5.12914	8.46E-07	2.94E-05	5.19435
TMEM100	1.61907	-0.5042	5.11835	8.83E-07	3.05E-05	5.146
AC00803	1.67624	#NAME?	5.13463	8.93E-07	3.08E-05	5.21769
IGLV2-8	1.93249	5.97827	5.11294	9.05E-07	3.12E-05	5.12236
IGHV3-74	1.80017	5.74612	5.10801	9.26E-07	3.18E-05	5.10083
GPM6A	1.85737	#NAME?	5.10314	9.58E-07	3.28E-05	5.08223
IGHGP	1.72308	6.28165	5.09837	9.67E-07	3.31E-05	5.0588
RORC	1.78517	-0.8507	5.09478	9.83E-07	3.35E-05	5.04314
KEL	1.79642	-2.5117	5.09095	1.00E-06	3.41E-05	5.02646
TMEM132	2.09918	#NAME?	5.13933	1.00E-06	3.41E-05	5.13643
IGHV3-66	1.83255	3.63952	5.08645	1.02E-06	3.46E-05	5.00687
IGLV4-69	1.76728	#NAME?	5.04	1.27E-06	4.22E-05	4.80698
TNNC1	1.76761	-0.2757	5.03359	1.30E-06	4.32E-05	4.7777
TMPRSS2	1.62594	1.40748	5.02904	1.32E-06	4.40E-05	4.75809
IGLV7-43	1.90054	#NAME?	5.02459	1.36E-06	4.51E-05	4.74052
CTSE	2.825	2.77034	5.01309	1.42E-06	4.70E-05	4.68932
IGLV3-19	1.9079	7.9999	4.99778	1.52E-06	5.00E-05	4.62346
IGHV3-11	1.79538	6.40964	4.97473	1.69E-06	5.48E-05	4.52458
IGKV4-1	1.75035	8.85862	4.95765	1.82E-06	5.88E-05	4.45153
IGLV1-44	1.66037	7.45924	4.95475	1.84E-06	5.94E-05	4.43915
IGLV1-50	1.50011	#NAME?	4.94934	1.90E-06	6.12E-05	4.41802
IGLV3-27	2.01552	#NAME?	4.94524	1.93E-06	6.19E-05	4.40052
SFTPB	3.10008	6.48992	4.94184	1.95E-06	6.24E-05	4.38406
IGHV5-51	1.87089	8.05782	4.93477	2.01E-06	6.42E-05	4.35395
IGHG3	1.72405	8.5953	4.92572	2.10E-06	6.67E-05	4.31547
IGHV1-46	1.80381	6.0391	4.91833	2.17E-06	6.86E-05	4.28405
IGHV3-21	1.84658	6.7796	4.91246	2.22E-06	7.01E-05	4.25913
IGHV2-70	1.88736	#NAME?	4.91147	2.26E-06	7.10E-05	4.25931
IGKV2-24	1.99064	5.1657	4.89997	2.35E-06	7.36E-05	4.20618
AC13642	1.69412	#NAME?	4.90413	2.37E-06	7.41E-05	4.22323
COL6A5	1.52324	-1.8667	4.87062	2.68E-06	8.26E-05	4.08214
IGHV3-15	1.72249	6.94307	4.86729	2.71E-06	8.37E-05	4.06811
COPDA1	1.52663	#NAME?	4.86775	2.81E-06	8.62E-05	4.06903
IGHV4-31	1.84094	5.55957	4.85895	2.82E-06	8.63E-05	4.03298
IGHV4-55	1.51718	#NAME?	4.85704	2.85E-06	8.72E-05	4.02732
ALPP	2.02661	#NAME?	4.87503	2.89E-06	8.81E-05	4.09231
ARHGEF3	1.53345	-2.3855	4.84575	2.98E-06	9.09E-05	3.97747
IGHV1-2	1.9954	6.4267	4.84238	3.03E-06	9.21E-05	3.96333
CRB2	1.61436	#NAME?	4.84065	3.11E-06	9.45E-05	3.95422
IGHJ3P	1.62415	#NAME?	4.8288	3.28E-06	9.90E-05	3.917
IGKV1OR	1.7795	3.46063	4.82365	3.29E-06	9.91E-05	3.88482
IGKV1-9	1.84504	6.58023	4.81929	3.35E-06	0.0001	3.86655
ACKR1	1.73349	1.55112	4.8037	3.59E-06	0.00011	3.80142
IGHV3-3	1.63823	6.29592	4.79616	3.71E-06	0.00011	3.76996
IGHG1	1.65167	10.3092	4.79006	3.81E-06	0.00011	3.74455
CDH8	-1.6114	#NAME?	-4.7893	3.84E-06	0.00011	3.74388
IGKV1-6	1.782	5.69087	4.78227	3.94E-06	0.00012	3.71213
KRT7-AS	1.93836	#NAME?	4.7817	3.99E-06	0.00012	3.7093
AC00801	-1.6888	#NAME?	-4.7833	4.06E-06	0.00012	3.69988
IGHV3-1	1.9191	#NAME?	4.77493	4.08E-06	0.00012	3.68429
ANKRD44	1.60382	#NAME?	4.77814	4.11E-06	0.00012	3.7031
SFTPD	2.57228	3.80113	4.77236	4.11E-06	0.00012	3.67092

HEPHL1	-1.529	#NAME?	-3.9642	9.15E-05	0.0016	0.92056
SOX11	-1.9505	#NAME?	-3.9185	0.00011	0.00183	0.74966
IL1A	-1.5978	1.01253	-3.9126	0.00011	0.00186	0.73017
NMRAL2F	2.08635	2.98358	3.8574	0.00014	0.00219	0.5287
RNU6-57	-2.2636	#NAME?	-4.5999	0.00016	0.00245	-0.199
IGLV2-11	-1.5452	#NAME?	-3.7962	0.00018	0.00265	0.30806
IGFL3	-1.6141	#NAME?	-3.7747	0.0002	0.00288	0.222
CYP4F29F	1.51893	#NAME?	3.76167	0.0002	0.00296	0.19781
AL13332	1.74321	#NAME?	3.76247	0.00021	0.00303	0.29554
RNU6-56	-1.7441	#NAME?	-3.987	0.00022	0.00309	-0.1829
GSTA1	2.16017	#NAME?	3.71517	0.00024	0.00339	0.02119
IGHV1-3	-1.5773	#NAME?	-3.7167	0.00024	0.00341	0.05098
CASP14	-2.3374	#NAME?	-3.71	0.00025	0.00349	0.02482
AC01099	-4.3108	#NAME?	-4.3262	0.00026	0.00355	-1.3065
CAPN8	1.54053	#NAME?	3.69343	0.00026	0.0036	-0.0474
IGKV2D-	-1.7235	#NAME?	-3.6752	0.00028	0.00381	-0.0972
AL58998	2.47637	#NAME?	3.69757	0.00031	0.00407	0.15025
LINC0093	1.53678	#NAME?	3.64224	0.00033	0.00428	-0.1733
SOX2-OT	1.50238	-0.8237	3.61142	0.00035	0.00455	-0.3377
AC01189	-2.0572	#NAME?	-3.684	0.00039	0.0049	-0.5533
XKR7	1.56121	#NAME?	3.5925	0.00039	0.00493	-0.3297
LINC0113	1.60163	#NAME?	3.54596	0.00045	0.0055	-0.5597
RNA5SP2	-2.152	#NAME?	-3.6705	0.00047	0.00562	-0.2826
VENTXP5	1.80888	#NAME?	3.66474	0.00047	0.00566	-0.1732
AL35979	-4.5373	#NAME?	-3.6107	0.00068	0.00748	-0.5138
CACNA1E	1.56197	#NAME?	3.4295	0.00069	0.00753	-0.9371
AL39142	1.70041	#NAME?	3.43738	0.00071	0.00772	-0.7631
LINC0124	-5.9669	#NAME?	-3.9897	0.00074	0.00797	-1.8334
NTS	2.36779	#NAME?	3.33796	0.00095	0.00961	-1.2392
KCNA4	1.68985	#NAME?	3.29875	0.00114	0.01106	-1.2149
TENM2	-1.5957	0.59382	-3.2755	0.00117	0.01134	-1.4358
LHX8	1.74566	#NAME?	3.26305	0.00129	0.01217	-1.3684
AC00554	3.87958	#NAME?	3.44027	0.0015	0.01364	-1.2878
AC08728	-2.2314	#NAME?	-3.4275	0.00152	0.01378	-2.0712
AC09349	1.62134	#NAME?	3.21402	0.00159	0.01429	-1.3478
LGR5	-1.5321	#NAME?	-3.1767	0.00164	0.0146	-1.7396
PRAME	1.54347	#NAME?	3.17643	0.00164	0.0146	-1.7406
MRPS10P	-3.8607	#NAME?	-3.5173	0.00197	0.01684	-1.7202
AC00905	1.76782	#NAME?	3.14592	0.00236	0.01927	-1.54
CYP4F3	1.78848	1.62687	3.02668	0.00268	0.0212	-2.1847
MIR4314	-2.0456	#NAME?	-3.355	0.00277	0.02175	-2.4261
AC10591	-1.5511	#NAME?	-3.195	0.00316	0.02405	-1.7081
AL59042	1.60658	#NAME?	3.2674	0.00373	0.02728	-1.8124
AC09373	-2.0861	#NAME?	-3.0067	0.00391	0.02828	-1.9288
GSTA9P	1.76737	#NAME?	2.8963	0.00408	0.02914	-2.4901
ZIC3	1.87916	#NAME?	2.91424	0.00411	0.02932	-2.2288
LINC0206	-2.6032	#NAME?	-3.0291	0.0043	0.03037	-1.9783
AC00851	2.0003	#NAME?	2.87227	0.00486	0.03321	-2.2699
AC00246	-2.2335	#NAME?	-2.8581	0.00527	0.03517	-2.2313
AC00533	1.60942	#NAME?	2.80762	0.00533	0.03537	-2.779
UGT1A7	1.74986	#NAME?	2.76141	0.00612	0.03917	-2.894
IFNWP5	4.68215	#NAME?	4.53411	0.00716	0.04405	-3.4157
PRAC2	1.58481	#NAME?	2.68918	0.0078	0.04689	-2.9232

TOMM20	-1.7444	#NAME?	-12.214	2.25E-28	3.97E-26	53.7146
LINC0089	-2.087	#NAME?	-12.184	2.62E-28	4.62E-26	53.5619
CCR1	-1.6589	1.27914	-12.089	2.66E-28	4.68E-26	53.5315
TRBV6-1	-2.1332	#NAME?	-12.234	3.09E-28	5.42E-26	53.4071
APOL3	-1.6414	1.48555	-12.069	3.16E-28	5.51E-26	53.3631
BATF	-1.7061	1.96249	-12.068	3.19E-28	5.55E-26	53.3534
AL031846	-1.9158	#NAME?	-12.196	3.20E-28	5.55E-26	53.3687
MS4A4A	-1.7887	1.69207	-12.059	3.44E-28	5.95E-26	53.2777
CCR7	-1.8929	0.23558	-12.038	4.11E-28	7.06E-26	53.1022
TRAV2	-2.0066	#NAME?	-12.222	4.26E-28	7.30E-26	53.095
ALOX5AP	-1.6085	2.74793	-12.019	4.85E-28	8.25E-26	52.9384
GPR132	-1.5489	-0.2681	-12.015	4.99E-28	8.47E-26	52.9097
P2RY8	-1.9183	-0.4857	-12.002	5.61E-28	9.48E-26	52.7957
FASLG	-2.2834	#NAME?	-11.995	6.29E-28	1.06E-25	52.6832
HLA-DRB	-1.9176	5.49371	-11.969	7.43E-28	1.25E-25	52.5171
EPSTI1	-1.5667	1.34658	-11.926	1.07E-27	1.78E-25	52.1601
CLECL1	-1.8546	-2.3071	-11.905	1.28E-27	2.13E-25	51.9799
KLRD1	-1.8356	-2.848	-11.897	1.37E-27	2.26E-25	51.9147
CXCL9	-2.8123	3.45047	-11.875	1.65E-27	2.72E-25	51.7289
SPOCK2	-1.6744	2.01422	-11.867	1.76E-27	2.90E-25	51.6649
TRAV8-2	-1.856	#NAME?	-11.978	2.07E-27	3.38E-25	51.5294
TRAV13-	-2.0585	#NAME?	-11.923	2.53E-27	4.12E-25	51.3257
ZAP70	-1.9147	-0.1327	-11.81	2.87E-27	4.66E-25	51.1841
LINC019	-1.976	#NAME?	-11.902	2.93E-27	4.74E-25	51.1806
PRKCB	-1.6871	-0.5661	-11.787	3.46E-27	5.57E-25	50.9991
TRAV12-	-2.0519	#NAME?	-11.92	3.68E-27	5.90E-25	50.9625
SLCO2B1	-1.7706	1.33525	-11.769	4.06E-27	6.48E-25	50.8426
HLA-DOF	-1.8319	3.85015	-11.767	4.12E-27	6.57E-25	50.8265
NAPSB	-1.768	1.35061	-11.761	4.34E-27	6.88E-25	50.7747
CD40LG	-2.2255	#NAME?	-11.755	4.78E-27	7.55E-25	50.6827
SUSD3	-1.5458	1.17465	-11.748	4.85E-27	7.63E-25	50.6668
APOE	-1.8559	5.91805	-11.728	5.71E-27	8.95E-25	50.5041
LINC012	-1.9757	#NAME?	-11.851	6.25E-27	9.76E-25	50.4384
DPEP2	-1.5576	-0.8778	-11.708	6.78E-27	1.05E-24	50.3361
INPP5D	-1.5361	0.75992	-11.707	6.85E-27	1.06E-24	50.3258
FCGR3A	-1.7138	3.6655	-11.704	7.01E-27	1.09E-24	50.302
LINC023	-2.4294	#NAME?	-11.666	1.02E-26	1.57E-24	49.938
TMEM176	-1.5869	4.41991	-11.656	1.05E-26	1.61E-24	49.9072
LINC0244	-2.5262	#NAME?	-11.632	1.42E-26	2.18E-24	49.6076
UBD	-2.5371	2.11909	-11.61	1.55E-26	2.36E-24	49.5222
WDFY4	-1.8026	-1.1179	-11.593	1.78E-26	2.71E-24	49.3826
GGTA1P	-1.5455	-0.6172	-11.552	2.51E-26	3.80E-24	49.0421
GBP5	-2.1587	1.39366	-11.545	2.67E-26	4.03E-24	48.9827
DOCK10	-1.5318	-0.3052	-11.541	2.77E-26	4.16E-24	48.9459
TRBV5-4	-2.0794	#NAME?	-11.656	2.99E-26	4.45E-24	48.8949
SOCAR	-1.583	#NAME?	-11.534	3.31E-26	4.92E-24	48.7737
TRBV11-	-2.092	#NAME?	-11.673	3.34E-26	4.95E-24	48.7899
CD79B	-2.1243	0.44425	-11.517	3.39E-26	5.02E-24	48.7459
TRGC2	-2.1272	#NAME?	-11.508	3.74E-26	5.52E-24	48.6509
IL32	-1.6353	3.35034	-11.504	3.77E-26	5.54E-24	48.6425
CLEC4D	-1.9053	#NAME?	-11.525	3.95E-26	5.80E-24	48.6004
MS4A7	-1.6393	1.38364	-11.49	4.24E-26	6.20E-24	48.5263
ZC3H12D	-1.5644	-1.9537	-11.486	4.39E-26	6.41E-24	48.491
PSMB9	-1.6497	3.58401	-11.471	4.99E-26	7.26E-24	48.3657
RP11-65	-1.5508	#NAME?	-11.549	5.53E-26	7.98E-24	48.2805
HK3	-1.5997	0.80454	-11.436	6.65E-26	9.58E-24	48.0815
TLR7	-1.8845	-1.6068	-11.41	8.26E-26	1.19E-23	47.8683
TRAV21	-1.9663	#NAME?	-11.526	8.38E-26	1.20E-23	47.8766
TLR10	-2.1513	-1.8138	-11.387	1.00E-25	1.42E-23	47.6799
TRGC1	-1.993	#NAME?	-11.382	1.15E-25	1.64E-23	47.5423
AC00458	-1.9358	-0.9526	-11.359	1.27E-25	1.80E-23	47.4437
FMI4-AS	-1.8107	#NAME?	-11.412	1.33E-25	1.87E-23	47.4136
NFATC2	-1.5669	-0.1708	-11.354	1.32E-25	1.87E-23	47.4039
TRBV12-	-2.0532	#NAME?	-11.476	1.49E-25	2.10E-23	47.3106
TRAV41	-1.9655	#NAME?	-11.486	1.81E-25	2.52E-23	47.1301
TRBV5-6	-1.9317	#NAME?	-11.471	1.85E-25	2.58E-23	47.1024
TRAV17	-1.9171	#NAME?	-11.393	1.86E-25	2.59E-23	47.0828
FPR1	-1.796	1.11965	-11.312	1.87E-25	2.60E-23	47.0596
TRAV3	-2.1085	#NAME?	-11.424	1.91E-25	2.64E-23	47.0647
MEI1	-1.7344	-0.6832	-11.257	2.95E-25	4.06E-23	46.6122
SPIB	-2.6575	-1.408	-11.244	3.31E-25	4.54E-23	46.4993
CD300C	-1.5441	-0.2776	-11.234	3.58E-25	4.90E-23	46.4224
GRAP2	-1.5409	-1.5154	-11.224	3.88E-25	5.30E-23	46.3413
PTPN7	-1.6921	0.54328	-11.216	4.17E-25	5.67E-23	46.2705
TRAV22	-1.7994	#NAME?	-11.461	4.55E-25	6.16E-23	46.2349
CCL3	-1.5624	1.55325	-11.191	5.09E-25	6.87E-23	46.0734
PDE6G	-1.6027	-1.195	-11.163	6.44E-25	8.67E-23	45.8415
SIGLEC14	-1.8965	#NAME?	-11.146	9.95E-25	1.33E-22	45.4228
KLRC4-KI	-1.9203	#NAME?	-11.192	1.06E-24	1.41E-22	45.3731
CXCR4	-1.5363	4.42035	-11.101	1.08E-24	1.43E-22	45.3324
LTB	-1.8827	1.93905	-11.099	1.10E-24	1.45E-22	45.3169
PLA2G2D	-3.037	0.11699	-11.097	1.11E-24	1.46E-22	45.3066
SAMD3	-1.8937	-3.6314	-11.09	1.18E-24	1.56E-22	45.2437
GZMM	-1.8245	#NAME?	-11.088	1.23E-24	1.61E-22	45.2074
TRAV8-4	-1.7417	#NAME?	-11.163	1.56E-24	2.04E-22	44.9922
PLAAT4	-1.6885	3.96728	-11.056	1.56E-24	2.04E-22	44.9677
CXCR2P1	-2.8099	#NAME?	-11.065	1.61E-24	2.10E-22	44.9424
SPNS3	-1.5819	#NAME?	-11.052	1.71E-24	2.23E-22	44.8797
AC00492	-1.5476	#NAME?	-11.055	1.87E-24	2.42E-22	44.7972
VNN2	-2.2141	-0.3014	-11.012	2.23E-24	2.87E-22	44.6162
CXCL10	-2.4289	3.92827	-11.011	2.26E-24	2.89E-22	44.6055
TRAV26-	-1.7189	#NAME?	-11.124	2.64E-24	3.38E-22	44.4793
GBP1	-1.5817	3.36643	-10.988	2.72E-24	3.47E-22	44.4213
AC00772	-1.5788	#NAME?	-10.991	2.94E-24	3.74E-22	44.3457
LCP1	-1.5396	4.34748	-10.964	3.31E-24	4.18E-22	44.2279
LILRA5	-1.6044	0.04853	-10.964	3.33E-24	4.20E-22	44.2209
TIMD4	-2.2863	#NAME?	-10.983	3.43E-24	4.31E-22	44.1962
LY9	-1.8229	-1.9624	-10.949	3.75E-24	4.70E-22	44.1039
IFNG	-2.4502	#NAME?	-10.977	4.21E-24	5.25E-22	43.9999
OR2I1P	-2.328	1.51169	-10.896	5.81E-24	7.23E-22	43.6721
IL9R	-1.7002	#NAME?	-10.894	6.13E-24	7.60E-22	43.62
LINC0099	-1.8247	-2.6023	-10.875	6.85E-24	8.45E-22	43.5096
ZBP1	-1.9142	-1.1803	-10.871	7.11E-24	8.75E-22	43.473
LINC022	-1.5873	#NAME?	-10.908	7.34E-24	9.01E-22	43.451
BIRC3	-1.6729	1.71213	-10.867	7.36E-24	9.01E-22	43.4387
ANTXRLP	-1.7452	#NAME?	-11.135	7.88E-24	9.58E-22	43.4315
LINC0114	-1.7828	#NAME?	-10.928	8.42E-24	1.02E-21	43.324
CD180	-1.6921	-0.654	-10.839	9.19E-24	1.11E-21	43.2201
ZNF831	-2.1307	-3.5313	-10.836	9.46E-24	1.14E-21	43.192
ADGRG5	-1.9598	-1.6019	-10.825	1.03E-23	1.24E-21	43.1053
VSIG4	-1.9018	2.50813	-10.824	1.04E-23	1.25E-21	43.0957
AC02212	-2.0045	#NAME?	-10.968	1.09E-23	1.30E-21	43.0922
TRGV5	-2.0505	#NAME?	-10.824	1.25E-23	1.49E-21	42.9214
TRAV27	-1.7327	#NAME?	-11.019	1.30E-23	1.55E-21	42.9265
FCRL3	-2.3124	#NAME?	-10.772	1.63E-23	1.91E-21	42.6586
TRBV14	-1.7997	#NAME?	-11.066	1.80E-23	2.11E-21	42.6246
TREM2	-1.5836	2.73976	-10.749	1.91E-23	2.22E-21	42.4977
KIR3DX1	-1.587	#NAME?	-10.866	2.30E-23	2.66E-21	42.3505
GPR34	-1.6265	0.34928	-10.719	2.45E-23	2.82E-21	42.2536
LINC021	-1.9785	#NAME?	-10.729	2.81E-23	3.22E-21	42.1262
CCR8	-1.8423	#NAME?	-10.705	2.90E-23	3.31E-21	42.0879
RP11-556	-1.5167	#NAME?	-10.753	2.91E-23	3.31E-21	42.099
AC00603	-1.6996	#NAME?	-10.706	3.01E-23	3.41E-21	42.0545
LILRA2	-1.6305	-2.3208	-10.687	3.17E-23	3.59E-21	41.9985
TRAV14D	-1.8184	#NAME?	-10.835	4.04E-23	4.55E-21	41.8027
FLT3	-1.9675	#NAME?	-10.646	4.51E-23	5.05E-21	41.6526
ACSL5	-1.778	1.67285	-10.638	4.72E-23	5.26E-21	41.6073
FYB1	-1.85	1.42067	-10.635	4.84E-23	5.38E-21	41.5814
PDCD1LG	-1.6499	0.61124	-10.63	5.05E-23	5.61E-21	41.5394
TRBV27	-1.9033	#NAME?	-10.888	5.62E-23	6.21E-21	41.497
TDRD6	-1.5709	-3.1428	-10.599	6.46E-23	7.10E-21	41.2969
HLA-DOF	-1.5101	1.20737	-10.593	6.79E-23	7.44E-21	41.2488
ZNF683	-2.1031	-1.0689	-10.592	6.82E-23	7.46E-21	41.2436
STAP1	-1.9888	-1.4293	-10.592	6.85E-23	7.47E-21	41.2405
KCNJ10	-2.0677	#NAME?	-10.582	7.55E-23	8.20E-21	41.145
LILRA4	-2.1382	#NAME?	-10.571	8.72E-23	9.42E-21	41.0038
JAKMIP1	-1.8893	#NAME?	-10.45	2.19E-22	2.34E-20	40.0961
AC24382	-1.7873	#NAME?	-10.458	2.59E-22	2.77E-20	39.9357
CXCL13	-2.5542	3.20553	-10.409	2.97E-22	3.15E-20	39.795
CD163	-1.747	2.28433	-10.409	2.97E-22	3.15E-20	39.7954
RP11-367	-1.73	#NAME?	-10.432	2.96E-22	3.15E-20	39.803
HSD11B1	-1.7784	0.89714	-10.391	3.45E-22	3.63E-20	39.6472
AC01189	-1.5718	-2.5512	-10.384	3.64E-22	3.82E-20	39.5937
CLEC9A	-1.7928	#NAME?	-10.417	3.87E-22	4.06E-20	39.5439
GNLY	-1.7915	1.2329	-10.352	4.70E-22	4.92E-20	39.3406
LGALS2	-1.6249	0.45842	-10.349	4.82E-22	5.03E-20	39.3171
CXCL11	-2.4918	0.44869	-10.343	5.03E-22	5.24E-20	39.2752
TRBV4-2	-1.9194	#NAME?	-10.431	5.29E-22	5.48E-20	39.2486
KLRC1	-1.9501	#NAME?	-10.334	5.69E-22	5.87E-20	39.1547
TTC24	-1.9225	#NAME?	-10.319	6.88E-22	7.07E-20	38.9718
EOMES	-1.7061	-1.4196	-10.292	7.60E-22	7.79E-20	38.8683
AC01591	-1.6978	#NAME?	-10.31	7.95E-22	8.14E-20	38.8309
AC01591	-1.6907	-0.9395	-10.272	8.87E-22	9.06E-20	38.7157
HSH2D	-1.544	0.89802	-10.265	9.34E-22	9.53E-20	38.6642
GAPT	-1.8325	-2.2885	-10.252	1.04E-21	1.05E-19	38.5589
ZNF80	-1.8744	#NAME?	-10.241	1.60E-21	1.61E-19	38.1465
AL365361	-1.8832	-2.1068	-10.196	1.61E-21	1.61E-19	38.1257
HLA-DOF	-2.0017	2.28405	-10.186	1.76E-21	1.75E-19	38.0414
SIGLEC8	-2.1646	#NAME?	-10.161	2.17E-21	2.15E-19	37.8323
JCHAIN	-2.6369	6.04791	-10.152	2.29E-21	2.26E-19	37.7823
AC01875	-1.6196	#NAME?	-10.19	2.51E-21	2.48E-19	37.7076
CYSLTR1	-1.5178	-1.6266	-10.134	2.64E-21	2.59E-19	37.6424
LINC0086	-2.0015	-2.8229	-10.097	3.53E-21	3.47E-19	37.3535
CLEC12A	-2.0073	-0.9907	-10.077	4.13E-21	4.04E-19	37.1991
IKZF3	-1.5609	0.33335	-10.068	4.45E-21	4.34E-19	37.1263
NCR1	-1.9266	#NAME?	-10.096	4.49E-21	4.37E-19	37.1265
AC02636	-1.9393	#NAME?	-10.145	4.62E-21	4.49E-19	37.1132
TRAV5	-1.6907	#NAME?	-10.244	5.27E-21	5.10E-19	37.0179
TRGV3	-1.657	#NAME?	-10.141	5.48E-21	5.30E-19	36.9492
CR1	-1.902	-2.7925	-10.02	6.48E-21	6.22E-19	36.7567
TRDC	-2.0177	#NAME?	-10.012	7.12E-21	6.82E-19	36.6649
IL12B	-1.6005	#NAME?	-10.008	8.57E-21	8.16E-19	36.4896
ADGRE1	-1.8857	#NAME?	-9.9647	1.01E-20	9.62E-19	36.3157
AL09681	-1.8996	#NAME?	-9.9859	1.14E-20	1.08E-18	36.2079
AL60680	-2.067	#NAME?	-9.9872	1.15E-20	1.09E-18	36.2023
PTCRA	-1.5467	#NAME?	-9.979	1.36E-20	1.28E-18	36.0432
LINC0168	-1.5751	#NAME?	-9.9789	1.38E-20	1.30E-18	36.0267
TRBV6-6	-1.7075	#NAME?	-10.051	1.45E-20	1.36E-18	36.0034
AL16241	-1.8528	#NAME?	-10.337	1.49E-20	1.39E-18	36.0518
LAX1	-1.7612	-0.4091	-9.8835	1.88E-20	1.75E-18	35.7058
IRF4	-1.9342	-0.3885	-9.8749	2.01E-20	1.87E-18	35.6399
LINC0148	-1.6723	-2.1189	-9.8298	2.86E-20	2.63E-18	35.2944
ITM2A	-1.523	1.87691	-9.8272	2.92E-20	2.68E-18	35.2746
NUGGC	-2.0435	#NAME?	-9.8187	3.16E-20	2.89E-18	35.1963
C11orf21	-1.725	-2.7592	-9.8167	3.16E-20	2.89E-18	35.195
TLR4	-1.594	0.51058	-9.816	3.18E-20	2.90E-18	35.1892
TRAV6	-1.6322	#NAME?	-9.9965	3.88E-20	3.52E-18	35.059
LINC0040	-1.894	#NAME?	-9.9149	4.00E-20	3.62E-18	35.0045
MSR1	-1.6078	1.07989	-9.7819	4.14E-20	3.74E-18	34.9292
CLNK	-1.6126	#NAME?	-9.7711	4.57E-20	4.11E-18	34.8337
CCL19	-2.451	2.95621	-9.7398	5.74E-20	5.13E-18	34.6086
IL10	-1.5014	-1.8903	-9.7119	7.11E-20	6.35E-18	34.3964
CLEC17A	-1.9676	#NAME?	-9.7191	7.21E-20	6.42E-18	34.3859
FCRLA	-2.1936	-1.2415	-9.6802	9.08E-20	8.06E-18	34.1565
TRBV4-1	-1.8275	#NAME?	-9.7856	1.13E-19	9.97E-18	33.9866
PTGDS	-1.8287	2.92612	-9.6332	1.30E-19	1.14E-17	33.8005
CD8B	-2.0337	0.18683	-9.6187	1.46E-19	1.27E-17	33.691
AC00738	-1.6897	#NAME?	-9.6206	1.51E-19	1.32E-17	33.655
TRAV20	-1.6013	#NAME?	-9.8234	1.91E-19	1.65E-17	33.5026
CRIP1	-1.5171	-0.4829	-9.5734	2.06E-19	1.78E-17	33.3496
FCGR1CP	-1.5604	#NAME?	-9.5743	2.44E-19	2.09E-17	33.1914
TRBV7-6	-1.6233	#NAME?	-9.6932	2.56E-19	2.20E-17	33.1847
BFSP2	-1.9454	#NAME?	-9.5862	2.69E-19	2.31E-17	33.102
LINC0252	-1.7171	#NAME?	-9.7229	3.02E-19	2.57E-17	33.0411
TRBV7-3	-1.603	#NAME?	-9.6002	3.02E-19	2.57E-17	32.9992
HTRA4	-1.7626	-1.5322	-9.4725	4.45E-19	3.74E-17	32.5924
CNR2	-2.0392	#NAME?	-9.4945	4.52E-19	3.80E-17	32.5859
CXCR5	-1.8379	#NAME?	-9.5277	4.86E-19	4.06E-17	32.5289
TRAV8-1	-1.706	#NAME?	-9.6063	4.92E-19	4.11E-17	32.5441
SSTR3	-1.7178	#NAME?	-9.4829	5.15E-19	4.29E-17	32.4607
TNFRSF1	-2.2632	#NAME?	-9.4537	5.26E-19	4.37E-17	32.4283
CXorf65	-1.6702	#NAME?	-9.4304	6.95E-19	5.73E-17	32.16
FOLR2	-1.5352	2.11435	-9.4046	7.44E-19	6.12E-17	32.0858
MS4A1	-2.7125	#NAME?	-9.4051	7.79E-19	6.40E-17	32.0428
P2RY12	-2.0356	#NAME?	-9.3989	8.93E-19	7.31E-17	31.9128
ARRDC5	-1.5544	#NAME?	-9.3987	9.06E-19	7.41E-17	31.903
KLRC4	-1.5922	#NAME?	-9.504	9.26E-19	7.56E-17	31.9196
CCL18	-2.0315	4.61612	-9.3613	1.03E-18	8.39E-17	31.7633
VCAM1	-1.5207	1.50194	-9.3469	1.15E-18	9.33E-17	31.6566
IL21-AS1	-1.6106	#NAME?	-9.4382	1.37E-18	1.11E-16	31.5314
SIRPB1	-1.597	-1.8894	-9.3179	1.43E-18	1.16E-16	31.4414
CD79A	-2.3256	2.62314	-9.3029	1.60E-18	1.29E-16	31.3301
IGHM	-2.7583	6.26884	-9.2924	1.73E-18	1.39E-16	31.2529
TRBV30	-1.7176	#NAME?	-9.3716	2.01E-18	1.60E-16	31.1449
CCL4L2	-1.6824	#NAME?	-9.2699	2.08E-18	1.65E-16	31.077
KCNA3	-1.7768	#NAME?	-9.2438	2.62E-18	2.06E-16	30.8497
CD19	-2.1172	-0.7601	-9.1946	3.61E-18	2.82E-16	30.5312
LINC0121	-2.0438	#NAME?	-9.1948	3.78E-18	2.94E-16	30.4901
ITGB2-AS	-1.5571	-0.8287	-9.1848	3.88E-18	3.02E-16	30.4598
XXYLT1-A	-1.5827	#NAME?	-9.217	4.15E-18	3.23E-16	30.4097
LINC0087	-1.5298	#NAME?	-9.1882	4.16E-18	3.23E-16	30.398
TRBV15	-1.7232	#NAME?	-9.3423	4.38E-18	3.39E-16	30.412
TRAV36D	-1.514	#NAME?	-9.3541	4.54E-18	3.51E-16	30.3805
TRGV7	-1.5211	#NAME?	-9.2146	4.97E-18	3.84E-16	30.2421
APOC1P1	-1.5963	#NAME?	-9.1923	5.18E-18	3.98E-16	30.1977
SH2D1B	-1.6788	#NAME?	-9.1104	6.92E-18	5.26E-16	29.8932
FCN1	-1.5809	-0.9596	-9.1054	7.02E-18	5.32E-16	29.8778
TRAV10	-1.5377	#NAME?	-9.2551	9.10E-18	6.86E-16	29.699
LAIR2	-1.6465	#NAME?	-9.0804	9.68E-18	7.29E-16	29.5694
CCR6	-1.5365	#NAME?	-9.0769	1.51E-17	1.13E-15	29.1538
CD22	-1.826	-1.1025	-8.9961	1.58E-17	1.18E-15	29.0817
AC01223	-1.732	-0.5116	-8.9359	2.46E-17	1.81E-15	28.6463
CR1L	-1.6334	#NAME?	-8.9574	2.67E-17	1.96E-15	28.5779
DTNB-AS	-1.805	#NAME?	-8.9214	2.97E-17	2.17E-15	28.4636
LINC0227	-1.6419	#NAME?	-8.9534	3.03E-17	2.21E-15	28.4604
BATF2	-1.5163	0.76291	-8.9011	3.17E-17	2.30E-15	28.395
TRAV1-2	-1.6881	#NAME?	-9.0091	3.28E-17	2.38E-15	28.4111
AC09952	-1.573	#NAME?	-8.893	3.51E-17	2.53E-15	28.2983
CHRM3-A	-1.5875	#NAME?	-8.8666	4.17E-17	2.99E-15	28.128
PARP15	-1.5455	-1.2674	-8.8591	4.31E-17	3.08E-15	28.093
GPR141	-1.6672	#NAME?	-8.8543	4.60E-17	3.27E-15	28.0306
S100B	-1.5731	0.7578	-8.8237	5.58E-17	3.95E-15	27.839
FPR2	-1.8706	#NAME?	-8.8231	5.67E-17	4.00E-15	27.8253
HAMP	-1.5972	-2.0528	-8.7627	8.70E-17	6.07E-15	27.4029
CHI3L2	-1.8091	-0.2266	-8.7108	1.27E-16	8.73E-15	27.0332
CCL23	-1.6412	#NAME?	-8.6654	1.99E-16	1.36E-14	26.5958
LINC0178	-1.8435	#NAME?	-8.7032	2.09E-16	1.41E-14	26.5758
ACSM5	-1.7291	#NAME?	-8.6297	2.65E-16	1.78E-14	26.3176
SUCNR1	-1.7884	-1.181	-8.5929	2.96E-16	1.98E-14	26.199
TENT5C	-1.5449	1.5914	-8.5888	3.05E-16	2.04E-14	26.1696
MRC1	-1.5683	1.31634	-8.5788	3.28E-16	2.19E-14	26.0992
LINC005	-1.6375	#NAME?	-8.5966	3.79E-16	2.52E-14	25.9783
HLA-DRB	-1.7769	3.2594	-8.5389	4.37E-16	2.88E-14	25.8187
ACOD1	-1.7059	#NAME?	-8.6735	4.72E-16	3.10E-14	25.8202
MZB1	-2.1309	2.819	-8.5116	5.30E-16	3.47E-14	25.6275
KIR2DL4	-1.7884	#NAME?	-8.4909	6.60E-16	4.29E-14	25.418
IGLV3-1	-2.471	#NAME?	-8.4356	9.18E-16	5.94E-14	25.0891
SLC12A3	-1.7169	#NAME?	-8.4397	1.01E-15	6.49E-14	25.004
GPR25	-1.633	#NAME?	-8.4558	1.03E-15	6.62E-14	24.9923
TRBV13	-1.5749	#NAME?	-8.5619	1.10E-15	7.05E-14	24.989
FCRL1	-2.2129	#NAME?	-8.4207	1.11E-15	7.12E-14	24.9083
FCRL4	-1.9413	#NAME?	-8.4414	1.20E-15	7.65E-14	24.848
BLK	-2.0999	#NAME?	-8.3919	1.27E-15	8.09E-14	24.773
TCL1A	-2.3145	#NAME?	-8.3983	1.33E-15	8.42E-14	24.7349
AC00913	-1.5079	#NAME?	-8.3947	1.58E-15	9.97E-14	24.5791
FCER2	-2.1067	#NAME?	-8.3673	1.69E-15	1.07E-13	24.4969
LINC0257	-1.5606	#NAME?	-8.335	1.93E-15	1.21E-13	24.3617

TRGC1	-1.8372	-2.7769	-7.6249	5.88E-12	6.30E-10	16.8155
CHRM3-A	-2.1986	-3.1084	-7.6235	5.93E-12	6.32E-10	16.8082
AL60680	-2.3914	-3.403	-7.5979	6.78E-12	7.20E-10	16.6772
AC02636	-2.3814	#NAME?	-7.625	7.58E-12	7.95E-10	16.5959
CSMD2	1.54477	-2.8249	7.5687	7.90E-12	8.21E-10	16.5281
KIR3DX1	-1.6953	#NAME?	-7.6318	7.91E-12	8.21E-10	16.5592
TRAV14D	-1.7199	#NAME?	-7.579	9.26E-12	9.53E-10	16.3929
AL02365	-1.7068	#NAME?	-7.6151	9.33E-12	9.58E-10	16.401
TNN	2.00061	#NAME?	7.54276	9.36E-12	9.60E-10	16.3653
HSH2D	-1.6189	1.51658	-7.5016	1.12E-11	1.14E-09	16.1859
KLRC1	-2.0674	#NAME?	-7.4914	1.22E-11	1.24E-09	16.1061
TRAV8-2	-1.6503	#NAME?	-7.4514	1.56E-11	1.57E-09	15.8756
ZNF683	-2.1794	-0.2171	-7.4354	1.58E-11	1.59E-09	15.8499
ACSM5	-1.8342	#NAME?	-7.4375	1.62E-11	1.62E-09	15.833
C11orf21	-2.0235	#NAME?	-7.4355	1.64E-11	1.63E-09	15.8232
LINC0040	-1.9843	#NAME?	-7.4204	1.83E-11	1.80E-09	15.7175
TRAV19	-1.7755	#NAME?	-7.4079	1.95E-11	1.90E-09	15.6562
CXorf65	-1.92	#NAME?	-7.3993	2.04E-11	1.98E-09	15.611
AC13306	-1.7916	0.04536	-7.3771	2.15E-11	2.07E-09	15.5544
GALNT15	1.66946	-1.0032	7.36685	2.26E-11	2.18E-09	15.5028
LINC009	-1.5309	-3.5035	-7.36	2.34E-11	2.25E-09	15.4683
AC01591	-1.6699	-0.2256	-7.3465	2.51E-11	2.40E-09	15.4001
AL12198	-1.705	#NAME?	-7.3557	2.55E-11	2.43E-09	15.3935
CLNK	-1.5591	-3.1224	-7.3407	2.59E-11	2.46E-09	15.3707
PCAT29	-1.9027	#NAME?	-7.3988	2.59E-11	2.46E-09	15.403
PATL2	-1.6864	-1.5385	-7.3273	2.78E-11	2.63E-09	15.303
PARP15	-1.7858	-0.707	-7.2476	4.19E-11	3.85E-09	14.9022
ARMH1	-1.5337	-1.2153	-7.241	4.34E-11	3.96E-09	14.869
ISM1	1.9022	0.61256	7.23613	4.45E-11	4.05E-09	14.8444
HAMP	-1.5955	-1.3847	-7.2363	4.45E-11	4.05E-09	14.8453
PTGES3P	-1.6665	#NAME?	-7.2482	4.58E-11	4.16E-09	14.8259
TRAV8-4	-1.5955	#NAME?	-7.2294	4.75E-11	4.30E-09	14.7844
C9orf139	-1.7043	#NAME?	-7.2146	5.28E-11	4.74E-09	14.6856
AC11334	1.9773	#NAME?	7.26968	5.34E-11	4.79E-09	14.6965
NPFFR1	-1.7132	-3.7794	-7.1966	5.46E-11	4.88E-09	14.646
TIMP3	1.86183	0.48557	7.18698	5.73E-11	5.11E-09	14.5979
TRAV17	-1.5346	#NAME?	-7.1932	5.89E-11	5.24E-09	14.5808
LINC0171	1.94113	#NAME?	7.21038	6.10E-11	5.39E-09	14.5531
TRBV3-1	-1.6016	#NAME?	-7.1918	6.30E-11	5.54E-09	14.5222
MS4A1	-2.7988	0.011	-7.1604	6.57E-11	5.75E-09	14.4649
LINC0086	-1.9176	-1.9566	-7.1593	6.61E-11	5.77E-09	14.4593
TRBV9	-1.784	#NAME?	-7.1751	6.65E-11	5.80E-09	14.4666
LINC020	-1.5141	#NAME?	-7.1608	6.75E-11	5.87E-09	14.4435
LINC0092	1.67214	#NAME?	7.16858	6.88E-11	5.97E-09	14.4299
ANTXRLP	-1.7436	#NAME?	-7.1878	7.06E-11	6.10E-09	14.4229
TRBV5-6	-1.7546	#NAME?	-7.1695	7.06E-11	6.10E-09	14.412
VNN2	-1.7943	0.77303	-7.1201	8.08E-11	6.91E-09	14.2637
TWIST1	1.79985	1.6191	7.1042	8.77E-11	7.47E-09	14.1845
LINC014	-1.7127	-1.4344	-7.0945	9.22E-11	7.79E-09	14.136
MMP13	4.07973	2.27921	7.08361	9.74E-11	8.19E-09	14.0819
HTR1B	1.6653	#NAME?	7.11644	1.19E-10	9.88E-09	13.9241
PTGER3	1.76066	-2.7113	7.03952	1.22E-10	1.01E-08	13.8628
EOMES	-1.5106	-0.6371	-7.0242	1.32E-10	1.10E-08	13.7867
SKAP1	-1.5642	1.41885	-7.01	1.42E-10	1.18E-08	13.7162
TRAV8-1	-1.9022	#NAME?	-6.9948	1.86E-10	1.53E-08	13.4801
TRBV11-	-1.8974	#NAME?	-6.9511	2.02E-10	1.65E-08	13.3829
TCL1A	-2.9104	#NAME?	-6.9317	2.23E-10	1.79E-08	13.2876
TLR10	-2.0419	-0.8759	-6.9186	2.26E-10	1.81E-08	13.2645
SYNDIG1	1.62337	0.21885	6.90578	2.41E-10	1.92E-08	13.2015
UBD	-2.0886	3.33867	-6.8925	2.58E-10	2.04E-08	13.1361
LINC021	-1.7975	#NAME?	-6.8652	3.03E-10	2.38E-08	12.9823
AC13364	-1.6765	#NAME?	-6.8691	3.13E-10	2.45E-08	12.9607
TAFA5	1.53038	0.31621	6.85184	3.17E-10	2.47E-08	12.9363
THEMIS	-1.7344	-0.4934	-6.8488	3.21E-10	2.51E-08	12.9213
CHIT1	-2.6548	1.1343	-6.827	3.59E-10	2.78E-08	12.8147
AC12752	-1.5837	#NAME?	-6.8234	3.75E-10	2.89E-08	12.7774
TRAV3	-1.7532	#NAME?	-6.8265	3.78E-10	2.91E-08	12.7732
FCRL3	-1.9252	-1.4764	-6.807	3.97E-10	3.04E-08	12.7166
TRAV8-3	-1.6728	#NAME?	-6.8262	4.09E-10	3.13E-08	12.7091
IDO2	-2.0117	#NAME?	-6.8061	4.09E-10	3.13E-08	12.6907
STAP1	-1.6887	-0.4915	-6.7856	4.42E-10	3.36E-08	12.6118
TRBV6-6	-1.606	#NAME?	-6.7803	4.77E-10	3.60E-08	12.5482
KLRC4	-1.8884	#NAME?	-6.85	4.99E-10	3.76E-08	12.5676
SEMA3C	1.57897	3.24853	6.75667	5.11E-10	3.84E-08	12.4706
C1QTNF3	1.75659	0.43096	6.7485	5.33E-10	3.97E-08	12.4308
TRBV5-4	-1.6753	#NAME?	-6.7589	5.44E-10	4.04E-08	12.4231
AC01591	-1.5172	#NAME?	-6.742	5.63E-10	4.17E-08	12.3786
TRAV21	-1.6841	#NAME?	-6.7517	5.78E-10	4.26E-08	12.3713
TIMD4	-1.896	#NAME?	-6.7383	6.18E-10	4.52E-08	12.3065
CCL18	-1.7378	5.56939	-6.7128	6.37E-10	4.63E-08	12.2571
PTPRN	2.44669	-2.8446	6.70552	6.60E-10	4.79E-08	12.2216
KLRC2	-1.9396	#NAME?	-6.7185	6.81E-10	4.93E-08	12.2115
TRBV10-	-1.6235	#NAME?	-6.7208	7.08E-10	5.11E-08	12.1814
KLRK1	-1.7578	-2.5406	-6.6796	7.52E-10	5.40E-08	12.0956
AC00468	-2.0569	#NAME?	-6.6752	8.24E-10	5.87E-08	12.0189
IGLON5	1.92141	-1.8946	6.65477	8.51E-10	6.05E-08	11.9753
RP3-4921	-1.7659	#NAME?	-6.8068	9.20E-10	6.52E-08	12.0169
CNR2	-2.1541	#NAME?	-6.6411	9.32E-10	6.60E-08	11.8895
SPIB	-2.753	-0.3315	-6.6165	1.03E-09	7.27E-08	11.7901
TSPAN32	-1.5423	-1.7741	-6.6082	1.07E-09	7.53E-08	11.75
AL15787	-1.7152	#NAME?	-6.6165	1.08E-09	7.55E-08	11.7514
AC00416	1.54977	#NAME?	6.60564	1.14E-09	7.96E-08	11.6992
LINC017	1.78156	#NAME?	6.60348	1.15E-09	8.03E-08	11.6888
TRBV14	-1.6257	#NAME?	-6.6346	1.17E-09	8.14E-08	11.7071
BCL2L14	-1.8589	-1.7113	-6.5823	1.22E-09	8.46E-08	11.6253
LINC023	-2.0424	-1.3014	-6.5676	1.31E-09	9.04E-08	11.5543
FAM180A	1.53756	-2.0074	6.56633	1.32E-09	9.08E-08	11.5482
AC00756	-1.6576	-2.1263	-6.5648	1.33E-09	9.13E-08	11.541
RUFY4	-1.9814	-2.3574	-6.5574	1.38E-09	9.44E-08	11.5052
LINC015	2.05213	#NAME?	6.603	1.43E-09	9.78E-08	11.5001
TRAV12-	-1.5761	#NAME?	-6.5542	1.50E-09	1.02E-07	11.4382
KIR2DL4	-1.9521	#NAME?	-6.5372	1.63E-09	1.11E-07	11.3601
AL03159	-1.5277	#NAME?	-6.5379	1.66E-09	1.12E-07	11.3438
PTHLH	2.68799	3.96977	6.5144	1.71E-09	1.15E-07	11.2986
ITGB2-AS	-1.7033	-0.2299	-6.5082	1.76E-09	1.18E-07	11.2689
SH2D1B	-1.6405	#NAME?	-6.4968	1.90E-09	1.27E-07	11.199
TXK	-1.5295	-2.1655	-6.4834	1.99E-09	1.32E-07	11.1499
AC00551	-2.6511	#NAME?	-6.4993	2.05E-09	1.36E-07	11.144
LINC002	-1.5556	#NAME?	-6.479	2.22E-09	1.46E-07	11.0642
LILRA4	-1.8564	#NAME?	-6.4634	2.24E-09	1.48E-07	11.0393
SIGLEC8	-1.7295	#NAME?	-6.4362	2.57E-09	1.68E-07	10.9098
RTP5	-2.0716	#NAME?	-6.4881	2.62E-09	1.71E-07	10.9485
NCR1	-1.9784	#NAME?	-6.4268	2.74E-09	1.78E-07	10.8506
TRAV20	-1.5509	#NAME?	-6.4508	2.78E-09	1.81E-07	10.8659
KCND2	1.60468	-1.2755	6.41389	2.80E-09	1.81E-07	10.8183
APCDD1L	2.08348	-0.9119	6.41265	2.82E-09	1.82E-07	10.8125
CD8B	-1.8253	1.11076	-6.4092	2.87E-09	1.85E-07	10.7959
MFAP5	2.20998	0.68194	6.40381	2.95E-09	1.89E-07	10.7704
NEGR1	1.54606	-2.8404	6.39876	3.02E-09	1.94E-07	10.7463
MMP3	3.00231	#NAME?	6.39933	3.07E-09	1.96E-07	10.7321
CD19	-1.9552	0.1822	-6.3857	3.22E-09	2.05E-07	10.6842
PKD2L1	-1.7405	-2.298	-6.3602	3.65E-09	2.31E-07	10.5633
CCL11	1.59189	1.38384	6.35857	3.68E-09	2.33E-07	10.5555
TRAV5	-1.7632	#NAME?	-6.3974	3.86E-09	2.42E-07	10.561
TNFRSF1	-2.1038	-2.6002	-6.3404	4.02E-09	2.52E-07	10.4694
CD79B	-1.5308	1.54291	-6.3355	4.12E-09	2.58E-07	10.4462
CARMIL2	-1.5718	-0.7145	-6.3221	4.39E-09	2.74E-07	10.3828
AL35675	-1.5739	#NAME?	-6.3266	4.85E-09	3.01E-07	10.311
AC00727	-1.6978	#NAME?	-6.3562	5.04E-09	3.11E-07	10.3045
P2RX5	-1.7045	-1.244	-6.2892	5.16E-09	3.16E-07	10.2275
CR1L	-1.6962	#NAME?	-6.2926	5.37E-09	3.28E-07	10.2007
AC05574	-1.7095	#NAME?	-6.3875	5.49E-09	3.33E-07	10.2669
SCN2B	1.80772	#NAME?	6.27399	5.66E-09	3.43E-07	10.1402
OR2I1P	-1.8798	2.64383	-6.268	5.72E-09	3.46E-07	10.1277
PCDHGA	1.51108	-2.2921	6.25136	6.20E-09	3.70E-07	10.0495
IGF2	2.27438	1.61727	6.24026	6.54E-09	3.89E-07	9.99736
AL13924	-1.5251	#NAME?	-6.2239	7.21E-09	4.24E-07	9.90826
AL15807	-1.5799	#NAME?	-6.2097	8.50E-09	4.92E-07	9.77424
GUCA1A	1.5808	-2.6592	6.17443	8.99E-09	5.18E-07	9.68908
SUCNR1	-1.5491	-0.3486	-6.1674	9.30E-09	5.34E-07	9.65627
FCRL1	-2.4933	#NAME?	-6.1048	1.32E-08	7.39E-07	9.3276
NUGGC	-1.7343	-1.2578	-6.0913	1.34E-08	7.46E-07	9.30213
FCRL4	-2.0246	#NAME?	-6.1093	1.34E-08	7.48E-07	9.32761
IL11	1.85287	-0.226	6.0688	1.49E-08	8.26E-07	9.19799
AC09012	1.60924	#NAME?	6.09466	1.72E-08	9.41E-07	9.10866
LILRP2	-1.6356	#NAME?	-6.0723	1.73E-08	9.43E-07	9.0994
CXCR5	-1.9139	#NAME?	-6.0339	1.92E-08	1.04E-06	8.97916
TRBV4-2	-1.5474	#NAME?	-6.0219	1.93E-08	1.04E-06	8.96013
GRIA3	1.86325	#NAME?	6.01881	1.96E-08	1.06E-06	8.94018
CDH13	1.76188	0.86547	6.00979	1.98E-08	1.07E-06	8.92555
TRBV7-4	-1.5709	#NAME?	-6.0975	1.98E-08	1.07E-06	9.04028
AGER	-2.0176	2.21494	-5.9946	2.13E-08	1.14E-06	8.85557
TRBV12-	-1.6998	#NAME?	-5.981	2.42E-08	1.29E-06	8.74866
ITGAD	-1.629	#NAME?	-5.9691	2.52E-08	1.34E-06	8.70844
LINC0121	-1.9419	-1.871	-5.9581	2.53E-08	1.34E-06	8.68814
TRBJ2-7	-1.5518	#NAME?	-6.0096	2.66E-08	1.40E-06	8.72397
GRP	2.34476	#NAME?	5.95897	2.69E-08	1.41E-06	8.6396
AC01264	-1.5423	#NAME?	-5.9511	2.70E-08	1.42E-06	8.63304
JCHAIN	-1.9788	7.38374	-5.9387	2.77E-08	1.45E-06	8.59928
B3GAT1	-1.5803	-4.2717	-5.9339	2.84E-08	1.48E-06	8.57718
TRBV4-1	-1.6302	#NAME?	-5.9571	2.96E-08	1.54E-06	8.57893
AC00810	-1.6646	-0.7451	-5.915	3.10E-08	1.61E-06	8.49115
GLIS1	1.69329	-2.2742	5.89779	3.36E-08	1.73E-06	8.41252
TMEM156	-1.5321	0.23728	-5.8963	3.39E-08	1.74E-06	8.40584
AC03423	-1.6704	#NAME?	-5.9306	3.47E-08	1.78E-06	8.43789
CXCL10	-1.7392	5.18838	-5.883	3.61E-08	1.85E-06	8.34512
TRBV15	-1.718	#NAME?	-5.9397	3.66E-08	1.88E-06	8.41056
CSPG4	1.83811	1.59655	5.87719	3.71E-08	1.89E-06	8.31871
CD22	-1.8119	-0.3345	-5.8661	3.90E-08	1.99E-06	8.2682
SIGLEC14	-1.5198	#NAME?	-5.8691	4.10E-08	2.08E-06	8.23523
TENM3	1.6174	-0.3956	5.85299	4.15E-08	2.10E-06	8.20875
AC02212	-1.8381	#NAME?	-5.9105	4.44E-08	2.23E-06	8.23619
AL12212	-1.8414	#NAME?	-5.8803	4.62E-08	2.32E-06	8.16869
ACOD1	-1.8515	#NAME?	-5.8624	5.31E-08	2.64E-06	8.05101
APOC1P1	-1.6031	#NAME?	-5.8265	5.42E-08	2.69E-06	7.99907
IGFL2	1.95289	0.31888	5.79329	5.49E-08	2.72E-06	7.93862
MIR3142	-1.6029	#NAME?	-5.7799	5.93E-08	2.91E-06	7.86997
AC00801	2.06941	#NAME?	5.78672	6.10E-08	2.98E-06	7.85319
AC00449	-1.6816	#NAME?	-5.7776	6.27E-08	3.06E-06	7.82782
AC02065	-1.8781	#NAME?	-5.7579	6.76E-08	3.28E-06	7.75156
PMEPA1	1.59544	3.86174	5.7459	6.85E-08	3.32E-06	7.72529
NIBAN3	-1.9915	#NAME?	-5.7489	6.85E-08	3.32E-06	7.72774
NKX3-2	1.84539	#NAME?	5.74969	6.92E-08	3.35E-06	7.71991
AL16241	-2.067	#NAME?	-5.8251	7.73E-08	3.69E-06	7.77707
PIANP	1.79271	-1.8479	5.71531	7.89E-08	3.75E-06	7.5881
TNC	1.69992	4.939	5.702	8.40E-08	3.96E-06	7.52854
ANKRD44	-1.8586	#NAME?	-5.6898	9.54E-08	4.44E-06	7.43211
TMEM158	1.50688	2.65255	5.67395	9.56E-08	4.44E-06	7.40329
DBH-AS1	-1.5742	-1.0038	-5.6693	9.77E-08	4.52E-06	7.38271
BLK	-1.8774	#NAME?	-5.6636	1.02E-07	4.67E-06	7.34663
ACSL6	-1.6673	-4.0366	-5.6438	1.10E-07	5.03E-06	7.26888
GPA33	-1.6926	#NAME?	-5.6359	1.19E-07	5.40E-06	7.2127
EXOC3L4	-2.1048	-1.6629	-5.6213	1.22E-07	5.53E-06	7.16917
AP00110	1.50762	#NAME?	5.61243	1.30E-07	5.88E-06	7.10958
TRAV10	-1.5671	#NAME?	-5.6666	1.31E-07	5.88E-06	7.20094
TRBV27	-1.5065	#NAME?	-5.6367	1.33E-07	5.98E-06	7.14731
DBH	-1.5003	#NAME?	-5.5911	1.44E-07	6.42E-06	7.01535
BIRC7	-1.5054	#NAME?	-5.5755	1.52E-07	6.78E-06	6.95995
SOX11	2.87784	#NAME?	5.5827	1.55E-07	6.91E-06	6.9478
LINC0161	1.6973	-0.7412	5.56722	1.56E-07	6.93E-06	6.92995
AC00913	-1.5413	#NAME?	-5.5815	1.63E-07	7.22E-06	6.93074
TBC1D27	-2.0847	#NAME?	-5.5689	1.68E-07	7.39E-06	6.88635
IL21	-1.6218	#NAME?	-5.6519	1.80E-07	7.85E-06	6.97995
MIR4537	-1.8109	#NAME?	-5.6487	1.82E-07	7.95E-06	6.93483
GDNF	2.14357	#NAME?	5.52649	1.90E-07	8.27E-06	6.74131
AL51230	-2.0011	#NAME?	-5.4774	2.47E-07	1.04E-05	6.50872
TRBV23-	-1.9892	#NAME?	-5.5557	2.56E-07	1.07E-05	6.59704
LINC0015	-1.6822	#NAME?	-5.5425	2.65E-07	1.11E-05	6.58662
CD1B	-1.642	#NAME?	-5.4489	2.71E-07	1.13E-05	6.40579
KEL	-1.976	-2.5423	-5.4356	2.84E-07	1.18E-05	6.35364
TRDV1	-1.7556	#NAME?	-5.4544	3.23E-07	1.33E-05	6.33062
LINC023	-1.6829	#NAME?	-5.418	3.26E-07	1.34E-05	6.23947
AL13914	1.60401	#NAME?	5.46899	3.28E-07	1.34E-05	6.28267
AC24678	-1.9245	#NAME?	-5.4136	3.50E-07	1.42E-05	6.19283
FAM30A	-1.7052	#NAME?	-5.3774	3.73E-07	1.51E-05	6.09659
FGF5	2.12525	#NAME?	5.41971	4.13E-07	1.65E-05	6.06914
IGHM	-2.2127	7.61542	-5.3485	4.20E-07	1.68E-05	5.97667
TNNC1	-1.8025	#NAME?	-5.3407	4.40E-07	1.74E-05	5.9352
LAMC2	1.8079	5.91427	5.32839	4.60E-07	1.82E-05	5.89031
ART4	-1.5905	#NAME?	-5.3296	4.62E-07	1.82E-05	5.89073
MMP10	3.26131	3.46369	5.30668	5.06E-07	1.98E-05	5.7972
CXCL11	-1.7127	1.76687	-5.2702	5.95E-07	2.29E-05	5.6414
MAL	-1.5111	0.7977	-5.2695	5.97E-07	2.29E-05	5.63826
MMP1	2.23492	5.89098	5.2452	6.65E-07	2.53E-05	5.53475
GJB2	1.97209	5.54196	5.24174	6.75E-07	2.56E-05	5.52006
CLEC12B	-1.573	#NAME?	-5.2558	6.86E-07	2.60E-05	5.53866
SPOCK1	1.53905	1.44235	5.19723	8.22E-07	3.07E-05	5.33132
AC09214	-1.5986	#NAME?	-5.273	8.38E-07	3.12E-05	5.44128
LINC0252	-1.525	#NAME?	-5.2226	8.85E-07	3.28E-05	5.35968
AC09673	-1.7141	#NAME?	-5.2529	8.98E-07	3.32E-05	5.40062
CA4	-1.8683	#NAME?	-5.1704	9.64E-07	3.54E-05	5.19288
EPHA10	-1.8713	-3.4838	-5.1593	9.71E-07	3.56E-05	5.17126
HLA-DRB	-1.7431	4.01392	-5.1537	9.95E-07	3.64E-05	5.14791
VEGFD	-1.8017	#NAME?	-5.153	1.01E-06	3.68E-05	5.13793
CLSTN2	1.77929	-1.2775	5.14851	1.02E-06	3.71E-05	5.12592
AC02425	-1.6118	#NAME?	-5.1544	1.03E-06	3.76E-05	5.13329
CCL19	-1.7637	4.22471	-5.1434	1.04E-06	3.78E-05	5.10454
HLA-DOA	-1.9934	3.67604	-5.1004	1.26E-06	4.46E-05	4.92428
AC13949	-1.8712	#NAME?	-5.1347	1.27E-06	4.52E-05	4.9884
HEATR9	-1.5086	#NAME?	-5.1221	1.28E-06	4.55E-05	4.96652
CA12	1.64581	3.44364	5.08423	1.35E-06	4.76E-05	4.85685
HLA-DPB	-1.5276	-0.4903	-5.0732	1.41E-06	4.97E-05	4.81082
CXCL13	-1.5277	4.63768	-5.0703	1.43E-06	5.01E-05	4.79877
ST8SIA2	2.17029	#NAME?	5.07134	1.47E-06	5.12E-05	4.78381
LINC0242	-1.6169	#NAME?	-5.0569	1.64E-06	5.66E-05	4.71288
MMP2-AS	-1.7084	#NAME?	-5.0253	1.81E-06	6.16E-05	4.59829
SLC10A6	1.765	#NAME?	5.01816	1.83E-06	6.22E-05	4.57038
PLAC8	-1.7955	1.35873	-5.01	1.86E-06	6.32E-05	4.54899
CDH2	1.60712	-0.7347	4.97639	2.15E-06	7.16E-05	4.41029
CCDC8	1.63507	1.68518	4.96737	2.23E-06	7.39E-05	4.37326
CCL25	-1.605	#NAME?	-4.9588	2.42E-06	7.93E-05	4.32822
MIR4539	-1.626	#NAME?	-5.052	2.46E-06	8.04E-05	4.46289
KIAA154	2.06903	-0.825	4.91925	2.74E-06	8.82E-05	4.17625
BCORP1	-1.7968	#NAME?	-5.0038	2.76E-06	8.86E-05	4.29239
ADH1B	-2.3885	#NAME?	-4.9077	2.93E-06	9.34E-05	4.12203
SH2D5	1.91226	#NAME?	4.89945	3.01E-06	9.58E-05	4.09393
AC24678	-1.6065	#NAME?	-4.9209	3.13E-06	9.92E-05	4.13086
IGLV6-57	-1.7871	6.01796	-4.8832	3.20E-06	0.0001	4.02935
ACKR1	-1.532	1.27998	-4.8701	3.38E-06	0.00011	3.97655
ZFHX4	1.61597	-2.5757	4.86361	3.47E-06	0.00011	3.95009
TNFRSF1	-1.6271	1.07642	-4.8517	3.65E-06	0.00011	3.90187
FCER2	-1.9743	#NAME?	-4.8571	3.66E-06	0.00011	3.91986
AL13341	-1.6096	#NAME?	-4.8719	3.69E-06	0.00011	3.94415
TENM2	2.79983	#NAME?	4.82657	4.09E-06	0.00013	3.79553
GKN2	-2.3741	#NAME?	-4.8101	5.22E-06	0.00016	3.65307
KIR2DS4	-1.7628	#NAME?	-4.8218	5.39E-06	0.00016	3.6583
CD79A	-1.6024	3.85195	-4.7564	5.45E-06	0.00016	3.51938
AC00803	-1.7596	#NAME?	-4.7803	5.71E-06	0.00017	3.56309
COL4A3	-1.6055	-2.0182	-4.728	6.13E-06	0.00018	3.40618
COMP	1.95447	1.45718	4.7177	6.40E-06	0.00019	3.36524
ZYG11A	-1.594	-1.4128	-4.7098	6.62E-06	0.00019	3.33396
FCRL5	-1.6547	-0.2164	-4.7063	6.71E-06	0.00019	3.3202
RP11-401	1.50221	-1.5948	4.70023	6.88E-06	0.0002	3.29605
LINC017	-1.5503	#NAME?	-4.7102	7.08E-06	0.0002	3.31766
EPYC	1.86188	#NAME?	4.69879	7.13E-06	0.0002	3.27285
CYP4F35P	-1.5535	#NAME?	-4.6825	7.99E-06	0.00023	3.20458
IL13	-1.556	#NAME?	-4.66	1.14E-05	0.00031	3.06408

GPR18	1.62629	-1.9848	14.0747	1.56E-36	1.34E-34	72.2269
LINC0185	2.20308	#NAME?	14.1074	1.64E-36	1.41E-34	72.1829
PLN	2.5582	#NAME?	14.0721	1.66E-36	1.42E-34	72.1658
GZMH	2.03791	1.24884	14.0672	1.67E-36	1.43E-34	72.1585
SIRPB1	2.06517	-1.632	14.0667	1.67E-36	1.43E-34	72.1535
LILRA4	2.4185	#NAME?	14.0781	1.76E-36	1.50E-34	72.1076
MICAL2	1.56162	1.70923	14.055	1.87E-36	1.59E-34	72.0454
S1PR1	1.54294	1.81515	14.0528	1.91E-36	1.62E-34	72.0251
GAPT	2.12691	-2.0659	14.047	2.01E-36	1.71E-34	71.9712
FPR2	2.50183	#NAME?	14.0467	2.10E-36	1.78E-34	71.9317
FGF7	2.14959	-0.4449	14.0421	2.11E-36	1.78E-34	71.9263
KLRG1	1.64314	-1.7926	14.0385	2.18E-36	1.84E-34	71.8933
ADGRE1	2.26348	#NAME?	14.0402	2.23E-36	1.88E-34	71.8712
TRBV5-6	2.03056	#NAME?	14.2651	2.22E-36	1.88E-34	71.9116
FLT3	2.19157	#NAME?	14.0374	2.29E-36	1.92E-34	71.8456
DACT3	1.51785	-0.4109	14.0306	2.34E-36	1.97E-34	71.8198
CD79B	2.15164	0.60806	14.0187	2.62E-36	2.19E-34	71.7102
AC14509	1.5778	#NAME?	14.0343	2.64E-36	2.21E-34	71.7045
PRRX1	1.89428	2.32444	14.0036	3.01E-36	2.51E-34	71.5712
HECW2-A	1.83053	#NAME?	14.0028	3.15E-36	2.62E-34	71.5266
PRELP	2.17945	2.37298	13.9797	3.76E-36	3.12E-34	71.3506
TRAV2	2.0321	#NAME?	14.1998	3.98E-36	3.29E-34	71.3345
RFLNA	2.17588	0.27586	13.9657	4.28E-36	3.54E-34	71.2219
TRBV10-	1.99657	#NAME?	14.1607	4.66E-36	3.84E-34	71.1759
BIRC3	1.84855	1.88737	13.9565	4.66E-36	3.84E-34	71.1369
MFAP4	2.24381	3.89505	13.9528	4.83E-36	3.97E-34	71.1023
LY9	1.9491	-1.791	13.9361	5.64E-36	4.62E-34	70.9486
DACT1	1.9763	0.58698	13.9303	5.95E-36	4.87E-34	70.8948
FYB1	1.98955	1.59806	13.9224	6.41E-36	5.22E-34	70.8221
COL8A2	1.89356	1.88078	13.9225	6.40E-36	5.22E-34	70.823
AC243960	2.0197	-1.4142	13.9148	6.88E-36	5.60E-34	70.7521
INHBA	1.97859	2.31278	13.9062	7.44E-36	6.05E-34	70.6735
CD7	1.67415	1.26896	13.9011	7.80E-36	6.32E-34	70.6265
EDNRA	1.57624	1.74525	13.8904	8.62E-36	6.97E-34	70.5279
KIAA1755	1.72887	-1.6648	13.8767	9.78E-36	7.89E-34	70.4023
ADA2	1.81895	3.0273	13.8647	1.09E-35	8.81E-34	70.2918
FIBIN	1.97581	1.50546	13.8584	1.16E-35	9.32E-34	70.234
AC00492	1.67283	#NAME?	13.8734	1.31E-35	1.05E-33	70.1163
CFP	1.51106	-1.4661	13.8369	1.42E-35	1.13E-33	70.036
CCL3	1.65794	1.69637	13.8361	1.43E-35	1.14E-33	70.0286
CD244	1.66126	-1.5079	13.8075	1.86E-35	1.48E-33	69.7661
RP11-366	1.80796	#NAME?	13.8086	1.98E-35	1.57E-33	69.7054
CRYBB1	1.68178	#NAME?	13.802	2.10E-35	1.67E-33	69.6449
CNN1	1.72859	1.51545	13.784	2.31E-35	1.82E-33	69.5506
APOE	1.90336	6.06823	13.7781	2.44E-35	1.92E-33	69.4959
KLHL6	1.79842	-0.0004	13.7646	2.76E-35	2.18E-33	69.3724
LINC0086	2.18613	-2.6209	13.761	2.86E-35	2.25E-33	69.3389
CXCL12	2.06963	1.67263	13.76	2.88E-35	2.27E-33	69.3296
CORIN	1.92906	#NAME?	13.763	2.91E-35	2.28E-33	69.3227
AC11904	1.58252	#NAME?	13.7606	3.20E-35	2.50E-33	69.2298
DOK5	1.97237	#NAME?	13.7505	3.26E-35	2.55E-33	69.2073
MAN1A1	1.51095	2.76586	13.7427	3.38E-35	2.64E-33	69.1711
TBX21	1.79537	-1.666	13.7399	3.47E-35	2.71E-33	69.146
SGIP1	1.50001	-1.7343	13.7373	3.56E-35	2.77E-33	69.1215
F13A1	2.20521	2.11546	13.7349	3.63E-35	2.83E-33	69.1
P2RY14	1.51922	-0.986	13.7249	3.99E-35	3.09E-33	69.0083
SAMD9L	1.60235	1.44383	13.7229	4.06E-35	3.14E-33	68.9899
LY96	1.52698	3.20909	13.7228	4.06E-35	3.14E-33	68.989
CD248	1.54406	3.53348	13.7183	4.24E-35	3.27E-33	68.9478
GRAP2	1.58267	-1.3899	13.7117	4.50E-35	3.47E-33	68.8871
LINC0193	1.94884	#NAME?	13.8181	4.98E-35	3.82E-33	68.8107
SFRP4	2.63774	#NAME?	13.6994	5.23E-35	4.01E-33	68.7398
EML4-AS	1.86504	#NAME?	13.753	6.26E-35	4.80E-33	68.5751
COL5A1	1.89113	4.52666	13.6716	6.52E-35	4.99E-33	68.5197
LINC0114	1.8742	#NAME?	13.7915	6.59E-35	5.03E-33	68.5327
CTHRC1	1.58575	4.92737	13.6693	6.66E-35	5.08E-33	68.4992
GPR141	2.12115	#NAME?	13.6802	6.70E-35	5.10E-33	68.4948
APOC1	1.97456	4.86656	13.666	6.87E-35	5.22E-33	68.4688
LRRC15	2.79224	#NAME?	13.6411	8.95E-35	6.78E-33	68.2067
BNC2	1.94094	-1.6668	13.6209	1.04E-34	7.86E-33	68.0565
TRAV3	2.08286	#NAME?	13.7733	1.08E-34	8.16E-33	68.0479
TGM2	1.83912	4.24718	13.6053	1.20E-34	9.05E-33	67.9139
COL1A1	2.05888	7.78208	13.595	1.32E-34	9.94E-33	67.819
MEDAG	1.82484	1.0117	13.5891	1.40E-34	1.05E-32	67.7657
CAMK1G	1.65145	-1.9176	13.5888	1.40E-34	1.05E-32	67.763
PDCD1	2.00681	-0.1915	13.5768	1.56E-34	1.17E-32	67.6531
OMD	2.79747	#NAME?	13.584	1.68E-34	1.26E-32	67.5834
ZBP1	2.04626	-1.0004	13.5666	1.72E-34	1.28E-32	67.5597
SIGLEC8	2.4237	#NAME?	13.5573	1.94E-34	1.44E-32	67.4416
LINC0228	1.66961	#NAME?	13.6156	1.95E-34	1.45E-32	67.4477
COL15A1	1.89206	2.52317	13.5439	2.12E-34	1.57E-32	67.3529
L3MBTL4	1.66634	#NAME?	13.5828	2.26E-34	1.68E-32	67.2944
TIFAB	2.19662	#NAME?	13.5463	2.38E-34	1.76E-32	67.2409
TRGV7	1.84587	#NAME?	13.6788	2.52E-34	1.86E-32	67.2118
IRF4	2.23658	-0.1561	13.5183	2.68E-34	1.97E-32	67.1191
PDE4B	1.76841	0.69717	13.5114	2.85E-34	2.10E-32	67.0561
CATSPER	1.6865	-2.7436	13.5055	3.01E-34	2.21E-32	67.0031
CD300LB	1.71849	#NAME?	13.5114	3.05E-34	2.24E-32	66.9905
FBLN2	1.84279	3.55739	13.4987	3.20E-34	2.35E-32	66.9406
ACSL5	1.88227	1.83439	13.4946	3.33E-34	2.43E-32	66.9032
MEFV	1.686	-2.2504	13.4935	3.36E-34	2.45E-32	66.8938
C3	1.92806	5.03183	13.4862	3.60E-34	2.62E-32	66.8265
TBX5-AS	1.82048	-0.9525	13.4676	4.26E-34	3.10E-32	66.6579
TCF21	2.12806	#NAME?	13.4635	4.58E-34	3.32E-32	66.5871
CCL23	2.24234	#NAME?	13.5081	4.63E-34	3.36E-32	66.5856
LINC0238	2.3946	#NAME?	13.4633	4.74E-34	3.43E-32	66.553
AC007728	1.60763	#NAME?	13.4592	5.46E-34	3.93E-32	66.4163
SFRP2	2.34699	5.79915	13.437	5.64E-34	4.06E-32	66.3792
ASPN	2.17077	2.53301	13.4359	5.70E-34	4.09E-32	66.3691
PIM2	1.50916	4.17568	13.4282	6.12E-34	4.38E-32	66.2992
LINC0087	1.80893	#NAME?	13.4292	7.96E-34	5.69E-32	66.0445
SENCR	1.57034	-1.8595	13.3982	8.06E-34	5.76E-32	66.0257
FAM20A	1.61662	1.56928	13.3785	9.65E-34	6.86E-32	65.8476
FCAR	2.21836	#NAME?	13.3838	1.12E-33	7.96E-32	65.7009
CALHM6	1.74705	2.05952	13.3547	1.20E-33	8.48E-32	65.6311
CEACAM	1.85967	#NAME?	13.3542	1.25E-33	8.80E-32	65.5944
CPED1	1.85351	-0.9066	13.3474	1.28E-33	9.04E-32	65.5648
C1S	1.56434	5.60607	13.3387	1.39E-33	9.76E-32	65.4862
GPR82	1.51501	-2.6561	13.3285	1.53E-33	1.07E-31	65.393
DEPP1	1.5423	4.19554	13.3162	1.71E-33	1.19E-31	65.2817
AC01591	1.82206	-0.7762	13.3098	1.81E-33	1.26E-31	65.2237
ADAMTS	1.62078	1.00547	13.2956	2.06E-33	1.44E-31	65.0951
IL10	1.73327	-1.7108	13.2895	2.18E-33	1.52E-31	65.0394
SIGLEC5	1.91294	#NAME?	13.3145	2.19E-33	1.52E-31	65.042
HNMT	1.50827	1.64935	13.2689	2.63E-33	1.82E-31	64.8527
DCN	2.04597	4.42395	13.2319	3.68E-33	2.54E-31	64.5182
SIGLEC14	2.09935	#NAME?	13.2733	3.76E-33	2.59E-31	64.5105
GALNT15	2.11056	-1.5363	13.2271	3.85E-33	2.65E-31	64.4746
COL6A2	1.63461	6.08201	13.2252	3.92E-33	2.70E-31	64.4572
PGR	1.91693	-4.133	13.212	4.42E-33	3.04E-31	64.3377
ADAMTS	2.68824	-1.5863	13.2095	4.52E-33	3.10E-31	64.3155
CPXM1	1.80959	2.63868	13.1932	5.25E-33	3.60E-31	64.1678
AL096816	2.00817	#NAME?	13.2414	6.36E-33	4.36E-31	63.9915
AP00118	1.83629	#NAME?	13.1948	6.91E-33	4.72E-31	63.9029
AC03419	1.66342	#NAME?	13.2452	7.08E-33	4.83E-31	63.8903
MIR155H	1.55413	-0.7689	13.1574	7.27E-33	4.95E-31	63.8444
CLEC1A	1.60508	-0.6592	13.1503	7.75E-33	5.27E-31	63.7807
POU2F2	1.54525	-0.147	13.1474	7.96E-33	5.40E-31	63.754
MARCO	2.6422	2.19953	13.1448	8.15E-33	5.52E-31	63.7308
AC00738	1.97879	#NAME?	13.1562	8.33E-33	5.64E-31	63.7118
AC00636	1.72487	#NAME?	13.3877	8.93E-33	6.03E-31	63.6953
NLRP12	1.99097	#NAME?	13.1388	1.01E-32	6.80E-31	63.5237
PTCRA	1.77899	#NAME?	13.2156	1.03E-32	6.91E-31	63.5246
LINC0209	1.62343	#NAME?	13.1635	1.08E-32	7.27E-31	63.4619
CLECL1	1.74925	-2.2029	13.0992	1.23E-32	8.27E-31	63.32
PDE6G	1.57388	-1.0862	13.0941	1.29E-32	8.64E-31	63.2737
FCRL3	2.29401	#NAME?	13.0766	1.56E-32	1.04E-30	63.0861
VNN2	2.11736	-0.1683	13.0667	1.66E-32	1.11E-30	63.0271
TRBV6-6	1.931	#NAME?	13.2789	2.10E-32	1.40E-30	62.8451
TIMP3	2.17899	0.06538	13.0099	2.77E-32	1.84E-30	62.516
TRBV30	2.06004	#NAME?	13.2471	2.88E-32	1.91E-30	62.5379
PTPN7	1.65202	0.6552	12.9998	3.04E-32	2.01E-30	62.4252
HGF	1.9129	-1.2185	12.9906	3.31E-32	2.18E-30	62.3423
SOD3	1.56294	2.46981	12.9853	3.47E-32	2.28E-30	62.295
TLR10	2.05728	-1.6844	12.9848	3.48E-32	2.29E-30	62.2899
FCGR2C	1.78334	-0.8129	12.9808	3.61E-32	2.37E-30	62.2545
SSC5D	1.87602	0.45355	12.9714	3.94E-32	2.58E-30	62.1694
CALHM5	1.68272	-0.8826	12.9626	4.26E-32	2.79E-30	62.0908
AC00498	1.86834	#NAME?	12.9996	4.28E-32	2.80E-30	62.0947
ANTXRLP	1.67849	#NAME?	13.3242	4.36E-32	2.85E-30	62.1475
TRAV39	1.72319	#NAME?	13.2034	4.56E-32	2.97E-30	62.0798
RUNX1T1	1.79466	-2.6272	12.9431	5.08E-32	3.30E-30	61.9158
COL10A1	2.32445	2.50759	12.9368	5.38E-32	3.49E-30	61.8597
KLRD1	1.66649	-2.7643	12.9368	5.38E-32	3.49E-30	61.8597
SULF1	1.6851	3.89072	12.9358	5.43E-32	3.52E-30	61.8502
CCL18	2.36152	4.86396	12.9195	6.29E-32	4.07E-30	61.704
CHI3L2	2.35274	0.06898	12.9133	6.66E-32	4.29E-30	61.6482
TRAV22	1.80823	#NAME?	13.219	6.69E-32	4.30E-30	61.7128
TRGV5	2.05248	#NAME?	12.9262	7.77E-32	4.99E-30	61.5016
SOCAR	1.53529	#NAME?	12.9063	8.23E-32	5.27E-30	61.4417
AKAP2	1.96811	#NAME?	12.9177	8.39E-32	5.37E-30	61.4274
VSTM4	1.54491	0.10818	12.886	8.52E-32	5.44E-30	61.404
IL18RAP	1.70392	-1.6652	12.8824	8.80E-32	5.61E-30	61.3714
MIR100H	1.73658	-1.3613	12.8798	9.01E-32	5.74E-30	61.3484
CLEC17A	2.14661	#NAME?	12.8927	9.30E-32	5.92E-30	61.3205
F5	2.23812	-1.3843	12.8734	9.54E-32	6.06E-30	61.2912
LIPC	1.90605	#NAME?	12.8774	1.01E-31	6.37E-30	61.2417
LINC0227	1.93827	#NAME?	12.9643	1.06E-31	6.69E-30	61.2132
PDE1A	1.74491	-1.2294	12.8587	1.09E-31	6.89E-30	61.1594
SLFN12L	1.51166	-2.152	12.8481	1.20E-31	7.56E-30	61.0648
ASGR2	1.5338	#NAME?	12.8492	1.22E-31	7.70E-30	61.0463
TRBV4-2	2.05379	#NAME?	12.9818	1.28E-31	8.08E-30	61.0299
LINC0099	1.80608	-2.4743	12.839	1.30E-31	8.18E-30	60.9831
TRAV14D	1.88649	#NAME?	13.0643	1.33E-31	8.37E-30	61.0114
TENT5C	1.98368	1.83621	12.8272	1.45E-31	9.07E-30	60.8779
SGCD	1.89471	-1.2253	12.8245	1.48E-31	9.29E-30	60.8535
CREB3L1	1.73113	2.10625	12.8181	1.57E-31	9.83E-30	60.7966
APLNR	1.6993	0.91633	12.8141	1.63E-31	1.02E-29	60.7608
FAM180A	1.89857	#NAME?	12.8156	1.70E-31	1.06E-29	60.7179
PDZRN3	1.73635	0.32442	12.803	1.80E-31	1.12E-29	60.6609
CYP27A1	1.71625	3.06701	12.7987	1.87E-31	1.16E-29	60.6233
PLA1A	1.81871	-0.3718	12.7936	1.96E-31	1.22E-29	60.5777
DAAM2	1.50738	-0.0625	12.7879	2.06E-31	1.28E-29	60.5262
SPNS3	1.57261	#NAME?	12.7881	2.25E-31	1.39E-29	60.4446
AC01875	1.76655	#NAME?	12.8565	2.32E-31	1.44E-29	60.4316
TRBV7-6	1.82008	#NAME?	13.0348	2.58E-31	1.59E-29	60.3698
LINC0163	1.79504	#NAME?	12.8792	2.65E-31	1.64E-29	60.3077
TRBV11-	1.9073	#NAME?	12.9078	3.15E-31	1.94E-29	60.1477
ADAM19	1.559	1.69114	12.7268	3.58E-31	2.20E-29	59.9809
CASP5	1.64517	#NAME?	12.7172	4.13E-31	2.53E-29	59.8408
TIMD4	2.25536	#NAME?	12.737	4.23E-31	2.59E-29	59.8219
CCL13	2.25208	1.49465	12.7011	4.50E-31	2.76E-29	59.7522
LINC0193	2.15972	-2.0304	12.6992	4.58E-31	2.80E-29	59.7358
ADGRG5	1.93312	-1.4663	12.6984	4.62E-31	2.82E-29	59.7282
CPZ	1.86371	#NAME?	12.7003	4.80E-31	2.93E-29	59.6908
RNASE1	1.59335	5.75128	12.692	4.89E-31	2.98E-29	59.6717
TRIM61	1.85689	#NAME?	12.7574	4.92E-31	3.00E-29	59.6831
P2RY12	2.34383	#NAME?	12.727	5.06E-31	3.07E-29	59.6481
AC00416	1.78272	#NAME?	12.7069	5.22E-31	3.16E-29	59.6115
KCNJ5	2.10092	-0.7322	12.6753	5.68E-31	3.43E-29	59.5226
CCL19	2.70445	3.21179	12.6582	6.62E-31	4.00E-29	59.3709
CCN4	1.77187	1.63258	12.6528	6.95E-31	4.19E-29	59.3225
COMP	2.86365	1.07311	12.6418	7.67E-31	4.61E-29	59.2248
AC24382	1.86168	#NAME?	12.6715	8.78E-31	5.27E-29	59.1012
AC04104	1.55958	#NAME?	12.7182	9.23E-31	5.52E-29	59.0682
NUGGC	2.1896	#NAME?	12.6165	9.89E-31	5.90E-29	58.9736
ISM1	2.02389	0.48284	12.609	1.03E-30	6.14E-29	58.9329
NCR3	1.87363	#NAME?	12.6157	1.05E-30	6.27E-29	58.9127
ITM2A	1.6836	2.03666	12.5988	1.13E-30	6.70E-29	58.8431
OSM	1.71786	0.47558	12.598	1.14E-30	6.75E-29	58.8353
LTB	1.83089	2.06143	12.5931	1.19E-30	7.03E-29	58.7925
FAT4	1.72377	-1.4254	12.5859	1.27E-30	7.48E-29	58.7281
C5AR2	1.59635	-1.9318	12.5762	1.38E-30	8.14E-29	58.642
LINC0089	1.8684	#NAME?	12.6578	1.43E-30	8.41E-29	58.6304
CXCL10	2.35043	4.08266	12.5685	1.48E-30	8.71E-29	58.5737
GBP1	1.51631	3.46267	12.5568	1.64E-30	9.65E-29	58.4703
CPNE5	1.66321	0.4766	12.5505	1.74E-30	1.02E-28	58.4148
HLA-DOB	2.16181	2.47872	12.5504	1.74E-30	1.02E-28	58.4136
TRGV10	1.88562	#NAME?	12.6079	1.79E-30	1.05E-28	58.4006
CTSW	1.84	1.04207	12.5432	1.85E-30	1.08E-28	58.3496
LINC0128	1.81905	#NAME?	12.6812	1.89E-30	1.10E-28	58.369
AC24529	1.67893	-0.0452	12.522	2.24E-30	1.31E-28	58.1618
TMEM200	1.95481	-0.1345	12.505	2.61E-30	1.51E-28	58.0112
TRAV8-1	1.86211	#NAME?	12.761	2.62E-30	1.52E-28	58.0716
PIK3AP1	1.6167	1.7569	12.5018	2.68E-30	1.55E-28	57.9833
LPAR4	1.75767	#NAME?	12.5673	3.15E-30	1.82E-28	57.8453
IRAK3	1.51529	0.34683	12.4796	3.27E-30	1.88E-28	57.787
AC10688	1.7784	#NAME?	12.4908	3.48E-30	2.00E-28	57.7298
LSP1	1.50193	3.0484	12.4682	3.62E-30	2.07E-28	57.6864
ZAP70	1.68531	-0.0613	12.4664	3.68E-30	2.11E-28	57.6699
RP11-367	1.75418	#NAME?	12.4942	3.77E-30	2.16E-28	57.6536
AC24512	1.83838	#NAME?	12.488	3.98E-30	2.27E-28	57.6005
CXCL11	2.5452	0.64724	12.4482	4.33E-30	2.47E-28	57.5091
JCHAIN	2.74577	6.27373	12.4326	4.97E-30	2.82E-28	57.3712
TRBV27	1.87261	#NAME?	12.7401	6.76E-30	3.82E-28	57.1543
GBP5	1.95992	1.49216	12.3828	7.75E-30	4.37E-28	56.9319
DNM3OS	1.71972	-1.656	12.3679	8.85E-30	4.98E-28	56.8005
CSMD2	1.70876	-2.9892	12.365	9.08E-30	5.11E-28	56.775
SLCO2A1	1.83363	1.91203	12.3624	9.29E-30	5.21E-28	56.7524
PLXNA4	1.98227	-2.7705	12.3579	9.66E-30	5.42E-28	56.713
RP11-345	1.7001	#NAME?	12.3557	1.12E-29	6.27E-28	56.5687
GPR68	1.56524	1.60401	12.3389	1.14E-29	6.39E-28	56.5452
TMEM229	1.55132	-0.8501	12.3352	1.18E-29	6.59E-28	56.513
PNMA2	1.79249	-1.1645	12.3317	1.22E-29	6.79E-28	56.4824
NAIPP1	1.52502	#NAME?	12.429	1.34E-29	7.45E-28	56.4157
ZNF366	1.73965	#NAME?	12.3157	1.44E-29	7.99E-28	56.317
ACSM5	2.07028	#NAME?	12.3549	1.52E-29	8.40E-28	56.277
AOX1	1.79876	#NAME?	12.294	1.75E-29	9.63E-28	56.1263
XIRP1	2.25393	#NAME?	12.2975	1.78E-29	9.82E-28	56.1078
EPYC	2.77985	#NAME?	12.3815	1.80E-29	9.88E-28	56.1239
ACTA2-A	1.7253	-2.1161	12.2688	2.13E-29	1.17E-27	55.9297
TRGC1	1.81017	#NAME?	12.2741	2.25E-29	1.23E-27	55.8785
TREML1	1.59488	#NAME?	12.2628	2.49E-29	1.36E-27	55.7802
HPGDS	1.64334	-0.8161	12.2325	2.94E-29	1.60E-27	55.6113
AC00458	1.76206	-0.8629	12.2297	3.01E-29	1.64E-27	55.5865
MIR3945H	1.83875	#NAME?	12.2954	3.02E-29	1.64E-27	55.6016
ARRDC5	1.66809	#NAME?	12.2444	3.41E-29	1.85E-27	55.4703
AL357033	1.54726	#NAME?	12.2335	3.57E-29	1.93E-27	55.4248
DERL3	1.96099	2.46699	12.2023	3.84E-29	2.07E-27	55.346
STAP1	1.929	-1.3016	12.1934	4.15E-29	2.24E-27	55.2684
LINC0240	1.62459	#NAME?	12.2136	4.84E-29	2.61E-27	55.1267
ADAM8	1.56133	2.2209	12.1712	5.05E-29	2.72E-27	55.0739
TNFRSF1	2.49404	#NAME?	12.1715	5.29E-29	2.84E-27	55.0294
CLEC9A	1.90178	#NAME?	12.2057	5.91E-29	3.17E-27	54.9318
TRBV12-	1.88581	#NAME?	12.2926	6.08E-29	3.25E-27	54.9322
LYVE1	1.72078	-0.2019	12.1458	6.32E-29	3.38E-27	54.8519
AC09013	1.72539	#NAME?	12.2513	6.60E-29	3.53E-27	54.8389
LUM	1.9321	6.98173	12.1278	7.40E-29	3.95E-27	54.6946
MS4A14	1.60293	-2.0837	12.1223	7.77E-29	4.14E-27	54.6468
TRAV20	1.76525	#NAME?	12.4947	8.04E-29	4.28E-27	54.7137
IGLV2-11	3.12857	#NAME?	12.092	1.04E-28	5.51E-27	54.3592
GZMA	1.81409	2.69075	12.0787	1.14E-28	6.04E-27	54.2659
AC01223	2.01107	-0.3009	12.0774	1.15E-28	6.10E-27	54.2549
OR2I1P	2.17179	1.63536	12.0759	1.17E-28	6.17E-27	54.2415
AC00895	1.653	#NAME?	12.0964	1.22E-28	6.40E-27	54.2095
SLC16A4	1.51097	0.24417	12.0613	1.33E-28	6.98E-27	54.1148
AC09952	1.78317	#NAME?	12.0717	1.34E-28	7.01E-27	54.1125

IFNG-AS1	1.50687	#NAME?	4.76303	4.36E-06	0.00013	3.63761
AC00826	2.64607	#NAME?	4.88272	4.37E-06	0.00013	3.89876
CLDN18	2.42683	-0.0892	4.75398	4.45E-06	0.00013	3.59472
IGKV1-16	1.79313	5.94081	4.73202	4.89E-06	0.00014	3.50391
SLC34A2	2.35231	4.54783	4.71505	5.27E-06	0.00015	3.43399
IGHV4-28	1.54161	#NAME?	4.71357	5.32E-06	0.00015	3.4308
KHDRBS2	1.59448	#NAME?	4.69897	5.72E-06	0.00016	3.36514
IGKV1-17	1.7102	5.96346	4.68873	5.90E-06	0.00017	3.32585
NAPSA	2.63539	3.56069	4.68455	6.00E-06	0.00017	3.30875
IGLV3-21	1.87089	8.10429	4.67568	6.24E-06	0.00017	3.27243
AQP4	2.75145	#NAME?	4.66989	6.48E-06	0.00018	3.25225
IGKV1-5	1.64935	8.44999	4.66598	6.50E-06	0.00018	3.23278
IGHA1	1.60091	11.2492	4.66039	6.66E-06	0.00019	3.20999
IGHV4-59	1.73819	6.84058	4.65366	6.85E-06	0.00019	3.18253
MSLN	1.9231	1.68092	4.65105	6.93E-06	0.00019	3.17192
C4BPA	2.78421	#NAME?	4.65137	6.98E-06	0.00019	3.17369
IGKV1OR	1.72328	#NAME?	4.65197	7.37E-06	0.0002	3.17839
KBTBD12	1.83095	#NAME?	4.6435	7.38E-06	0.0002	3.13979
SFTPA1	3.21777	5.32464	4.62893	7.61E-06	0.00021	3.08197
SFTPA2	3.22155	5.71712	4.62269	7.82E-06	0.00021	3.05662
PRG4	1.6829	-1.8853	4.61371	8.12E-06	0.00022	3.02026
IGLV3-1	1.68659	7.08742	4.60743	8.34E-06	0.00023	2.99482
BMP5	1.76566	-0.5929	4.60467	8.44E-06	0.00023	2.98367
IGLL1	1.76717	#NAME?	4.60475	8.58E-06	0.00023	2.99816
PLA2G1B	1.94105	#NAME?	4.60496	8.81E-06	0.00024	2.97593
IGLL3P	1.82391	#NAME?	4.60149	8.94E-06	0.00024	2.98144
VEGFD	1.83738	-1.4328	4.57815	9.44E-06	0.00025	2.87669
IGLV3-10	1.88696	6.27394	4.56959	9.78E-06	0.00026	2.84224
LINC0055	1.52448	#NAME?	4.57989	9.87E-06	0.00026	2.89621
CA4	1.92083	#NAME?	4.5651	1.01E-05	0.00027	2.82034
KRT7	1.73347	4.13075	4.55804	1.03E-05	0.00027	2.79589
IGHV2-26	1.67545	#NAME?	4.54166	1.10E-05	0.00029	2.73383
SFTA3	2.57835	#NAME?	4.54503	1.12E-05	0.0003	2.75109
IGHV3-73	1.78607	4.74693	4.5356	1.13E-05	0.0003	2.70602
LINC0087	-1.8626	#NAME?	-4.5798	1.14E-05	0.0003	2.81
SSTR1	1.63425	#NAME?	4.51588	1.24E-05	0.00032	2.62872
IGKV1OR	1.73251	#NAME?	4.49216	1.36E-05	0.00035	2.53458
IGHV1-18	1.67831	7.79565	4.48797	1.38E-05	0.00035	2.51644
CD300LG	1.93825	#NAME?	4.4874	1.47E-05	0.00038	2.54492
CHRNA6	1.80036	#NAME?	4.46981	1.50E-05	0.00038	2.45232
CYP4B1	2.0838	#NAME?	4.45378	1.59E-05	0.0004	2.3852
SH2D5	-1.5685	#NAME?	-4.4469	1.64E-05	0.00042	2.35619
CFAP221	1.75029	-2.537	4.43325	1.73E-05	0.00043	2.30054
IGKV3D-	1.51391	#NAME?	4.43355	1.73E-05	0.00043	2.30566
FOLR1	2.13194	1.83856	4.42587	1.78E-05	0.00045	2.2716
IGLV3-25	1.62638	7.9636	4.42416	1.79E-05	0.00045	2.26489
AC13487	1.59863	#NAME?	4.41644	1.87E-05	0.00047	2.24708
IGHA2	1.60417	7.80024	4.40799	1.92E-05	0.00048	2.20157
LRRN4	1.58807	#NAME?	4.40103	1.98E-05	0.00049	2.17251
PLCXD3	1.59003	#NAME?	4.39839	2.02E-05	0.0005	2.17668
IGLV8OR	1.51422	#NAME?	4.44341	2.07E-05	0.00051	2.34762
ADAMTS	1.54294	-2.0204	4.37206	2.22E-05	0.00054	2.06156
OR2C3	-2.5077	#NAME?	-4.822	2.27E-05	0.00055	2.60364
CR2	1.83851	-0.8234	4.3575	2.36E-05	0.00057	2.00508
IL13	1.54952	#NAME?	4.37779	2.62E-05	0.00062	2.19355
IGLV7-46	1.65555	4.75938	4.3269	2.67E-05	0.00064	1.88687
IGLV2-11	1.58337	7.49276	4.32329	2.71E-05	0.00064	1.87296
IGKV1-8	1.58503	#NAME?	4.31076	2.86E-05	0.00068	1.82913
MYBPC2	1.7093	-1.6069	4.29565	3.03E-05	0.00071	1.7668
IGHV1-69	1.67155	#NAME?	4.28851	3.13E-05	0.00073	1.74388
CXCL13	1.53432	4.76695	4.27467	3.30E-05	0.00076	1.68661
SCN1A	1.849	#NAME?	4.28352	3.30E-05	0.00076	1.73261
IGLV1-36	1.61802	#NAME?	4.26013	3.52E-05	0.00081	1.64031
IGKV1-39	1.74943	#NAME?	4.25767	3.56E-05	0.00082	1.63577
IGHV1-24	1.59746	#NAME?	4.25277	3.61E-05	0.00082	1.6077
IGLV1-47	1.57299	#NAME?	4.2307	3.94E-05	0.00089	1.52405
C16orf89	2.02849	1.51195	4.22407	4.04E-05	0.00091	1.49441
PEBP4	2.16798	0.34031	4.21103	4.25E-05	0.00095	1.44517
ANKRD1	1.63283	#NAME?	4.18999	4.65E-05	0.00103	1.37555
IGHV1-69	1.77806	4.91978	4.18492	4.72E-05	0.00104	1.34696
GKN2	2.35812	#NAME?	4.19768	4.78E-05	0.00106	1.42388
MMP13	-2.0773	3.40074	-4.179	4.83E-05	0.00107	1.32469
SLC22A3	1.88956	0.60126	4.17257	4.95E-05	0.00109	1.30069
IGHV4-39	1.51562	7.51083	4.157	5.27E-05	0.00115	1.24248
MUCL3	2.00471	#NAME?	4.15138	5.57E-05	0.00121	1.23784
RBMY3AF	-5.9373	#NAME?	-9.5428	5.57E-05	0.00121	-1.0584
ABCA3	1.6314	2.70731	4.13506	5.74E-05	0.00123	1.16075
PLA2G2A	1.73497	0.39708	4.13064	5.84E-05	0.00125	1.14433
SLC13A5	-1.7143	#NAME?	-4.1242	6.01E-05	0.00128	1.11934
CYP2B7P	1.91314	0.00711	4.10758	6.39E-05	0.00135	1.05893
SCGB3A1	2.31679	#NAME?	4.1076	6.41E-05	0.00136	1.0578
AC00551	1.52409	#NAME?	4.10614	6.52E-05	0.00137	1.07301
CNTN6	1.59084	#NAME?	4.09203	6.97E-05	0.00145	1.0225
LINC0132	-2.1275	#NAME?	-4.3738	7.08E-05	0.00147	1.56252
LINC0199	1.83611	#NAME?	4.12292	7.82E-05	0.00161	1.16762
BAAT	1.58885	#NAME?	4.05464	7.94E-05	0.00163	0.87223
HNF1B	1.81754	#NAME?	4.0507	8.00E-05	0.00164	0.85492
IGKV3D-	1.57534	#NAME?	4.03013	8.66E-05	0.00176	0.77994
IGKV2D-	1.92214	#NAME?	4.0211	9.27E-05	0.00187	0.7665
NF1P8	-2.9879	#NAME?	-4.1772	9.50E-05	0.0019	1.16202
HP	2.11321	#NAME?	4.00006	9.72E-05	0.00193	0.67088
LINC0269	-2.5446	#NAME?	-5.4184	0.00011	0.00211	0.73733
IGKV3D-	1.63079	#NAME?	3.96597	0.00012	0.00225	0.56407
IGLV5-45	1.78347	#NAME?	3.95289	0.00012	0.00226	0.5067
IGKV1D-	1.7738	#NAME?	3.91303	0.00014	0.00259	0.36867
AC09371	-3.095	#NAME?	-5.2425	0.00015	0.00276	-0.2098
SCTR	1.70592	#NAME?	3.8855	0.00015	0.0028	0.26157
LINC0192	-2.1212	#NAME?	-4.2398	0.00015	0.00285	0.91657
CLC	1.69242	#NAME?	3.87785	0.00016	0.00302	0.29349
SFTPC	3.20432	#NAME?	3.85381	0.00017	0.0031	0.15009
IGLC7	1.6645	#NAME?	3.80063	0.00021	0.00366	-0.0354
CHIA	1.95072	#NAME?	3.79183	0.00022	0.00392	-0.016
IGLV4-3	1.61152	#NAME?	3.77189	0.00024	0.0041	-0.0928
SCGB3A2	2.10712	#NAME?	3.76153	0.00024	0.00413	-0.1703
IGKV1-33	1.54656	#NAME?	3.74327	0.00026	0.00439	-0.2212
GGTLC1	2.06182	#NAME?	3.74869	0.00026	0.00451	-0.1399
ORM1	1.64067	#NAME?	3.71151	0.00029	0.00485	-0.3299
SERTM2	1.69057	#NAME?	3.7115	0.00029	0.00491	-0.3127
LINC0201	1.98142	#NAME?	3.80031	0.00032	0.00525	0.09652
AC09752	-1.8396	#NAME?	-5.061	0.00034	0.00551	0.21472
AC23370	-2.7177	#NAME?	-4.3447	0.00038	0.00605	-0.6005
NKX2-1	1.83248	#NAME?	3.63402	0.00038	0.00607	-0.596
WFDC5	-1.6191	#NAME?	-3.6312	0.00039	0.00616	-0.6077
XAGE2	1.58721	#NAME?	3.6434	0.00039	0.00628	-0.5094
LINC0123	-1.9621	#NAME?	-3.6181	0.0004	0.00635	-0.6624
LINC0277	5.66354	#NAME?	7.82738	0.00041	0.00651	-1.8269
AC11114	-1.9964	#NAME?	-3.6449	0.00042	0.00656	-0.5084
ROS1	1.65514	0.57364	3.60237	0.00042	0.00661	-0.7138
IGKV1D-	1.60187	#NAME?	3.59432	0.00044	0.00692	-0.6958
AC24508	-3.2675	#NAME?	-4.4938	0.00048	0.00735	-0.2713
SFTA2	2.1614	#NAME?	3.56745	0.00048	0.00737	-0.8101
ORM2	1.53174	#NAME?	3.53358	0.00054	0.00816	-0.9139
LINC0122	-1.8303	#NAME?	-3.6014	0.00054	0.00818	-0.5901
MLLT10P	-4.2287	#NAME?	-4.6121	0.00056	0.00836	-0.9751
SLC6A2	-1.5778	#NAME?	-3.5035	0.0006	0.00891	-1.0384
LINC0005	-1.753	#NAME?	-3.5797	0.00061	0.00899	-0.7107
MS4A15	1.54535	#NAME?	3.49444	0.00062	0.00908	-1.0673
CAPN6	1.60375	#NAME?	3.48724	0.00063	0.00924	-1.084
IGKV1D-	1.54799	#NAME?	3.48601	0.00064	0.00929	-1.0882
IGKV1D-	1.59052	#NAME?	3.48549	0.00064	0.00937	-1.0551
AC00246	-2.7626	#NAME?	-3.6849	0.00069	0.00991	-0.4769
PGC	2.22116	#NAME?	3.46311	0.00069	0.00991	-1.1616
DMBT1	2.14327	1.07194	3.46189	0.00069	0.00991	-1.1719
LINC0206	-2.8186	#NAME?	-4.0183	0.00071	0.01018	-0.3304
IGLV1-41	1.69587	#NAME?	3.44721	0.00073	0.01037	-1.1847
IGHV2-70	1.80986	#NAME?	3.44542	0.00073	0.01038	-1.2046
AC01099	-4.6076	#NAME?	-5.5274	0.00076	0.01069	-1.5071
IGFL2-AS	-1.5685	#NAME?	-3.4292	0.00078	0.01088	-1.276
RNU6-56	-1.967	#NAME?	-4.3087	0.00081	0.01123	-0.6598
TRPC7-A	-1.5706	#NAME?	-4.2103	0.00083	0.01156	-0.5224
SPAG6	1.57912	#NAME?	3.39856	0.00086	0.01185	-1.36
LINC0124	-6.2208	#NAME?	-6.6886	0.00089	0.01218	-1.9971
AC02639	-3.7589	#NAME?	-4.5987	0.00091	0.01235	-1.322
AC12617	4.42886	#NAME?	6.64193	0.00092	0.01249	-1.6538
ATP13A4	2.05337	#NAME?	3.40768	0.00096	0.01286	-1.1609
AC08728	-2.4926	#NAME?	-4.7108	0.00101	0.0134	-1.4323
EMX2	-1.667	#NAME?	-3.3756	0.00102	0.01356	-1.3471
IGKV2D-	1.53034	#NAME?	3.31913	0.00115	0.01487	-1.5626
MMP10	-1.9291	4.16525	-3.3066	0.00117	0.01502	-1.66
MRGPRG	-2.7547	#NAME?	-3.9622	0.00121	0.01547	-1.3571
AC09653	1.5147	#NAME?	3.31294	0.00121	0.01553	-1.5102
AP00455	-2.935	#NAME?	-4.1724	0.00123	0.01565	-1.4177
RP11-445	-1.6321	#NAME?	-3.4052	0.00125	0.0159	-1.1441
IGHD	1.70328	4.03459	3.24456	0.00144	0.01776	-1.8497
AC10591	-1.8523	#NAME?	-3.8157	0.00148	0.01816	-0.93
PANX3	-1.9515	#NAME?	-3.3719	0.0016	0.01936	-1.3152
KRT23	-1.5587	1.0919	-3.2022	0.00165	0.0198	-1.9773
KLK8	-1.8756	#NAME?	-3.1971	0.00171	0.02036	-1.9543
AKR1C2	-1.5148	3.33059	-3.1793	0.00178	0.021	-2.0456
RNU6-57	-2.276	#NAME?	-4.1601	0.00182	0.0214	-1.2854
AC24285	-4.4852	#NAME?	-5.7019	0.0019	0.02209	-2.2058
PLA2G12	1.51684	#NAME?	3.16926	0.00198	0.02284	-1.9166
LINC0185	-3.7226	#NAME?	-5.0617	0.00199	0.02289	-2.0312
FGG	2.17689	#NAME?	3.13363	0.00207	0.02371	-2.165
PTF1A	-1.8601	#NAME?	-3.3911	0.00214	0.02427	-1.3958
AKR1B10	-1.8922	3.92801	-3.1086	0.00223	0.02508	-2.2543
AC07342	-3.3169	#NAME?	-3.309	0.00242	0.0267	-1.5132
LINC0062	-1.5053	#NAME?	-3.086	0.00243	0.02674	-2.2802
AC11756	-3.2766	#NAME?	-4.2845	0.00245	0.02689	-1.9403
SPATA8-A	-3.9669	#NAME?	-4.4875	0.00255	0.0277	-2.0275
AC01910	-2.1608	#NAME?	-4.0446	0.00271	0.02905	-1.9509
AC01876	-1.7115	#NAME?	-3.2106	0.00282	0.02996	-1.7629
PIGR	1.54777	2.78806	3.02464	0.00291	0.03065	-2.4963
LTF	1.58751	4.01797	3.02405	0.00291	0.03068	-2.498
NEFL	-1.8166	#NAME?	-3.003	0.00311	0.0323	-2.5505
AL079307	-1.7021	#NAME?	-3.2933	0.00314	0.03258	-1.6684
SST	-1.6533	#NAME?	-3.0542	0.00316	0.03272	-2.1809
AC01917	-1.5849	#NAME?	-3.6444	0.00323	0.03328	-1.6814
GJB6	-1.6012	3.80314	-2.9891	0.00324	0.03339	-2.5969
OTOP3	-2.0092	#NAME?	-2.9992	0.00337	0.03438	-2.4628
LINC0144	-1.9848	#NAME?	-3.002	0.00348	0.03529	-2.3762
AC09373	-2.1196	#NAME?	-3.1537	0.00355	0.03578	-1.8566
HCFC2P1	-3.4166	#NAME?	-3.8643	0.00359	0.03602	-2.1053
OTOP1	-1.6092	#NAME?	-3.0228	0.00374	0.03719	-2.1886
RARRES2	-1.645	#NAME?	-3.5719	0.0042	0.04043	-1.8745
AC02382	-1.5171	#NAME?	-3.2374	0.00427	0.0409	-1.9022
IRS4	-1.7643	#NAME?	-2.907	0.00437	0.04157	-2.7109
AL031713	-3.0102	#NAME?	-3.7352	0.00439	0.04174	-1.9148
AC11750	-2.4719	#NAME?	-3.3768	0.00481	0.04458	-1.9232
AL138826	-2.2387	#NAME?	-3.1121	0.00502	0.04607	-2.0489
FGA	1.69133	#NAME?	2.8424	0.0051	0.04655	-2.9821
HMGA1P	-2.6047	#NAME?	-3.4456	0.00526	0.04758	-2.2986
AP00165	-2.1586	#NAME?	-3.5215	0.00527	0.04767	-2.0412
DMRTA2	-1.5945	#NAME?	-2.8199	0.00546	0.04885	-3.0197
SMARCE1	1.56489	#NAME?	3.7102	0.00557	0.04949	-2.0216

LINC0236	-1.6007	-1.2214	-8.3098	2.21E-15	1.38E-13	24.2254
AQP9	-1.5908	0.4055	-8.3058	2.28E-15	1.42E-13	24.1978
TNFRSF1	-1.9754	#NAME?	-8.3165	2.39E-15	1.49E-13	24.1571
KLRC2	-1.7159	#NAME?	-8.3456	2.50E-15	1.55E-13	24.1315
IGKV1-5	-2.4932	6.70955	-8.2873	2.59E-15	1.60E-13	24.0707
MIR3142H	-1.6107	#NAME?	-8.2827	2.72E-15	1.67E-13	24.0241
DNAH8	-1.5782	#NAME?	-8.3173	2.73E-15	1.68E-13	24.043
AC13364	-1.6048	#NAME?	-8.2911	3.18E-15	1.94E-13	23.8858
HLA-DOB	-1.5431	#NAME?	-8.2507	3.37E-15	2.06E-13	23.8117
IGLV6-57	-2.5491	4.68194	-8.2196	4.16E-15	2.53E-13	23.6057
AC00803	-1.7701	#NAME?	-8.3216	4.48E-15	2.71E-13	23.6035
TMEM156	-1.6786	-0.5162	-8.2036	4.65E-15	2.81E-13	23.4962
IGKV1-8	-2.3243	#NAME?	-8.1955	5.15E-15	3.10E-13	23.3988
CD1B	-1.7191	#NAME?	-8.2126	5.28E-15	3.17E-13	23.3864
IGLV2-11	-2.4484	#NAME?	-8.1859	5.30E-15	3.18E-13	23.3683
IDO1	-1.9666	2.3582	-8.1778	5.56E-15	3.32E-13	23.3204
IGKJ5	-1.8615	#NAME?	-8.2216	5.63E-15	3.36E-13	23.3363
IFNG-AS1	-1.908	#NAME?	-8.2194	6.24E-15	3.70E-13	23.2424
FCRL2	-2.0606	#NAME?	-8.1461	7.20E-15	4.25E-13	23.0698
IGKV4-1	-2.4711	6.99324	-8.1137	8.68E-15	5.10E-13	22.8843
IGKC	-2.2613	9.16985	-8.107	9.09E-15	5.33E-13	22.839
IGLC2	-2.1366	8.19729	-8.088	1.04E-14	6.03E-13	22.7103
VNN1	-1.9503	-0.904	-8.0782	1.11E-14	6.44E-13	22.6435
IGLV2-14	-2.3832	6.51348	-8.0615	1.24E-14	7.19E-13	22.5307
DERL3	-1.5043	2.21979	-8.0609	1.25E-14	7.20E-13	22.5272
MMP2-AS	-1.7707	#NAME?	-8.0696	1.29E-14	7.41E-13	22.5073
AL133467	-1.5408	#NAME?	-8.0783	1.39E-14	8.00E-13	22.4379
IGKV3-20	-2.2549	7.48543	-8.0309	1.54E-14	8.77E-13	22.3246
IGLV9-49	-2.6177	#NAME?	-8.0141	1.84E-14	1.04E-12	22.1529
CD1E	-1.5992	#NAME?	-8.005	1.90E-14	1.07E-12	22.121
IGKV3-11	-2.2911	6.7638	-7.9973	1.93E-14	1.09E-12	22.0988
FCRL5	-2.1171	#NAME?	-7.9909	2.03E-14	1.15E-12	22.0494
IGKV2-30	-2.2467	#NAME?	-7.9981	2.10E-14	1.18E-12	22.0251
CCL25	-1.5848	#NAME?	-7.997	2.30E-14	1.29E-12	21.9414
IGLV2-23	-2.3796	5.93636	-7.9508	2.66E-14	1.48E-12	21.7865
TRDV1	-1.6199	#NAME?	-8.014	2.97E-14	1.64E-12	21.726
IGKV1-9	-2.4285	4.68535	-7.9107	3.49E-14	1.93E-12	21.5188
FAM30A	-1.9747	#NAME?	-7.8806	4.31E-14	2.36E-12	21.3127
IGHV3-2	-2.1974	#NAME?	-7.8502	5.30E-14	2.89E-12	21.1111
IGLV1-40	-2.3795	6.54858	-7.8413	5.59E-14	3.05E-12	21.0575
IGHV3-72	-2.3711	#NAME?	-7.8203	6.70E-14	3.62E-12	20.8833
GPA33	-1.5639	#NAME?	-7.805	7.79E-14	4.20E-12	20.7398
LINC0094	-1.8108	#NAME?	-7.7816	9.18E-14	4.93E-12	20.5802
C15orf48	-1.5423	3.45582	-7.7653	9.33E-14	5.01E-12	20.5559
MAP1LC3	-1.5512	#NAME?	-7.7886	9.40E-14	5.04E-12	20.5656
IGKV1-27	-2.3844	#NAME?	-7.7654	9.45E-14	5.06E-12	20.5449
CCDC141	-1.6146	#NAME?	-7.7572	1.02E-13	5.44E-12	20.4768
IGKV3-15	-2.1937	5.62444	-7.7301	1.18E-13	6.28E-12	20.3244
IGLV3-9	-2.583	#NAME?	-7.7284	1.21E-13	6.40E-12	20.3053
IGHV3-15	-2.2848	#NAME?	-7.724	1.24E-13	6.55E-12	20.2787
IGHV5-51	-2.3768	6.23714	-7.7194	1.27E-13	6.70E-12	20.2545
IGLV1-47	-2.23	5.41852	-7.7089	1.36E-13	7.17E-12	20.1853
IGLL5	-2.2067	4.1185	-7.6663	1.81E-13	9.46E-12	19.907
IGLV3-25	-2.2938	6.11731	-7.6488	2.03E-13	1.06E-11	19.7929
IGKV1-6	-2.2507	#NAME?	-7.6297	2.32E-13	1.20E-11	19.6635
RHEX	-1.6019	#NAME?	-7.6248	2.40E-13	1.24E-11	19.6314
IGHV4-55	-1.9905	#NAME?	-7.6266	2.46E-13	1.27E-11	19.611
IGLV5-45	-2.539	#NAME?	-7.6231	2.52E-13	1.30E-11	19.5885
IL22RA2	-1.5997	#NAME?	-7.6012	2.86E-13	1.47E-11	19.4625
IGHV3-3	-2.3546	#NAME?	-7.5658	3.54E-13	1.81E-11	19.2496
IGKV1-39	-2.3952	#NAME?	-7.5746	3.81E-13	1.94E-11	19.1921
LINC0239	-1.553	#NAME?	-7.5708	4.12E-13	2.09E-11	19.1289
COPDA1	-1.7388	#NAME?	-7.5858	4.16E-13	2.11E-11	19.1317
F5	-1.5438	-1.7057	-7.54	4.18E-13	2.11E-11	19.0881
IGKV3D-	-2.1689	#NAME?	-7.5447	4.29E-13	2.17E-11	19.0667
IGKV2-24	-2.3563	#NAME?	-7.5302	4.51E-13	2.27E-11	19.0148
IGLV4-69	-2.343	#NAME?	-7.5255	4.65E-13	2.33E-11	18.985
NIBAN3	-1.5408	#NAME?	-7.5182	4.85E-13	2.43E-11	18.9455
IGHV4-3	-2.2354	#NAME?	-7.5044	5.31E-13	2.65E-11	18.8542
IGKV1-17	-2.354	#NAME?	-7.484	6.10E-13	3.03E-11	18.7185
IGLC6	-1.8292	#NAME?	-7.4917	6.26E-13	3.10E-11	18.7009
MARCO	-1.75	1.80367	-7.4612	7.00E-13	3.46E-11	18.5822
CD70	-1.7856	#NAME?	-7.4409	8.04E-13	3.95E-11	18.4477
IGHV3-2	-2.2918	5.03395	-7.4279	8.70E-13	4.27E-11	18.3694
PNOC	-1.834	#NAME?	-7.4208	9.16E-13	4.47E-11	18.3198
IGLV1-44	-2.2068	#NAME?	-7.4186	9.30E-13	4.53E-11	18.3052
FCAR	-1.5441	#NAME?	-7.4157	9.74E-13	4.74E-11	18.2629
IGLV1-51	-2.1138	5.99339	-7.3947	1.08E-12	5.24E-11	18.1579
AICDA	-1.66	#NAME?	-7.4341	1.17E-12	5.64E-11	18.1266
IGHV1-18	-2.2088	5.91854	-7.3753	1.23E-12	5.91E-11	18.0347
TSHR	-1.5102	#NAME?	-7.3778	1.23E-12	5.91E-11	18.0366
AMPD1	-1.5999	#NAME?	-7.384	1.26E-12	6.05E-11	18.0235
HLA-DOA	-2.0724	2.78192	-7.3532	1.41E-12	6.79E-11	17.8947
AL122127	-1.5894	#NAME?	-7.3978	1.45E-12	6.95E-11	17.9194
IGKV1OR	-2.1279	#NAME?	-7.3347	1.60E-12	7.63E-11	17.7734
IGLV3-21	-2.329	6.44687	-7.328	1.66E-12	7.92E-11	17.7355
IGHV4-59	-2.209	#NAME?	-7.3237	1.72E-12	8.17E-11	17.7037
CHIT1	-2.0769	0.47176	-7.2851	2.19E-12	1.03E-10	17.4651
IGHG1	-2.0466	8.75352	-7.2788	2.28E-12	1.08E-10	17.4259
IGKV1D-	-2.0482	#NAME?	-7.2804	2.36E-12	1.11E-10	17.4002
CLIC5	-1.5013	-1.201	-7.2741	2.35E-12	1.11E-10	17.3963
IGKV3OR	-1.8591	#NAME?	-7.2823	2.44E-12	1.14E-10	17.3728
IGKV1OR	-1.8387	#NAME?	-7.3175	2.60E-12	1.21E-10	17.352
FXYD2	-1.5264	#NAME?	-7.2615	2.64E-12	1.23E-10	17.2863
IGHV3-30	-2.1094	#NAME?	-7.2532	2.71E-12	1.26E-10	17.2607
TRBV23-	-1.5355	#NAME?	-7.3831	2.79E-12	1.30E-10	17.3503
IGHV3-1	-2.3329	4.6227	-7.2331	3.06E-12	1.42E-10	17.1391
HLA-DPB	-1.7071	#NAME?	-7.2246	3.27E-12	1.51E-10	17.0772
EXOC3L4	-1.6549	-2.1946	-7.214	3.46E-12	1.59E-10	17.0197
CCL21	-1.6431	4.13842	-7.2084	3.59E-12	1.64E-10	16.9847
AIM2	-1.7197	1.08507	-7.1809	4.27E-12	1.95E-10	16.8135
IGHV3-48	-2.208	#NAME?	-7.1809	4.31E-12	1.97E-10	16.8051
IGHV3-7	-2.2832	#NAME?	-7.1766	4.43E-12	2.02E-10	16.7787
DCSTAMP	-1.5823	#NAME?	-7.1678	4.76E-12	2.16E-10	16.7114
IGLV7-43	-2.2805	#NAME?	-7.1601	4.97E-12	2.25E-10	16.668
IGKV3D-	-2.21	#NAME?	-7.1568	5.05E-12	2.29E-10	16.6511
IGKV1-16	-2.2952	#NAME?	-7.154	5.09E-12	2.30E-10	16.6421
IGLV3-19	-2.1655	#NAME?	-7.1274	6.03E-12	2.71E-10	16.4773
IGKV1-33	-2.0868	#NAME?	-7.1564	6.13E-12	2.76E-10	16.4869
IGHV3-53	-2.2172	#NAME?	-7.1235	6.30E-12	2.83E-10	16.4371
IGHV3OR	-1.7379	#NAME?	-7.1064	8.24E-12	3.67E-10	16.2
IGLL1	-1.6899	#NAME?	-7.0822	9.69E-12	4.29E-10	16.0429
IGHV4-28	-1.9313	#NAME?	-7.0348	1.09E-11	4.79E-10	15.9037
IGHA1	-2.0147	9.52573	-7.0095	1.26E-11	5.53E-10	15.7567
AC00551	-1.8555	#NAME?	-7.0126	1.40E-11	6.08E-10	15.6736
IGLV2-8	-2.1157	#NAME?	-6.9723	1.60E-11	6.90E-10	15.5261
IGLV3-10	-2.4405	#NAME?	-6.9706	1.61E-11	6.96E-10	15.5159
IGHV2-70	-2.2574	#NAME?	-6.9653	1.74E-11	7.50E-10	15.4491
IGKV3D-	-1.9677	#NAME?	-6.9624	1.75E-11	7.53E-10	15.4397
AC00801	1.98852	#NAME?	6.96102	1.79E-11	7.69E-10	15.4325
IGHV1-2	-2.3235	4.59084	-6.9435	1.90E-11	8.15E-10	15.3551
IGHV3OR	-1.6939	#NAME?	-6.9695	1.96E-11	8.39E-10	15.3579
IGHV4-34	-2.0333	5.13075	-6.9356	2.00E-11	8.54E-10	15.3069
IGHV1-24	-2.2216	#NAME?	-6.9362	2.01E-11	8.57E-10	15.3034
IGHV3-74	-2.0655	#NAME?	-6.9317	2.06E-11	8.81E-10	15.2763
IGKV1D-	-1.8801	#NAME?	-6.9385	2.39E-11	1.01E-09	15.1648
IGLC3	-1.9495	7.45061	-6.8957	2.56E-11	1.08E-09	15.0658
ANKRD36	-1.713	-1.9549	-6.8939	2.59E-11	1.09E-09	15.0547
TBC1D27	-1.6952	#NAME?	-6.9053	2.60E-11	1.09E-09	15.07
IGHV1-58	-2.2278	#NAME?	-6.8946	2.71E-11	1.14E-09	15.0157
FAM92B	-1.8474	#NAME?	-6.8721	3.05E-11	1.27E-09	14.8986
IGHV5-78	-1.7792	#NAME?	-6.8836	3.05E-11	1.27E-09	14.9136
MIR8071-	-1.569	#NAME?	-6.9071	3.05E-11	1.27E-09	14.947
IGKV3-7	-1.9254	#NAME?	-6.8629	3.31E-11	1.38E-09	14.8214
CR2	-2.1307	#NAME?	-6.8539	3.38E-11	1.41E-09	14.7999
IGHV4-61	-2.0411	#NAME?	-6.8417	3.69E-11	1.53E-09	14.7127
IGHV1-67	-1.8183	#NAME?	-6.8439	3.84E-11	1.59E-09	14.685
IGLV8-61	-2.2539	#NAME?	-6.8338	3.86E-11	1.60E-09	14.6692
IGHG3	-1.9172	7.24256	-6.8161	4.18E-11	1.72E-09	14.5874
IGKV1D-	-2.1365	#NAME?	-6.8116	5.40E-11	2.21E-09	14.377
IGKV1D-	-2.0331	#NAME?	-6.7826	5.45E-11	2.23E-09	14.3362
IGHV1-69	-2.1253	#NAME?	-6.7465	6.44E-11	2.61E-09	14.1663
IGHV3-7	-1.8427	#NAME?	-6.748	6.59E-11	2.67E-09	14.1487
GPR15	-1.6929	#NAME?	-6.7413	7.00E-11	2.83E-09	14.0916
IGKV1OR	-1.6139	#NAME?	-6.7811	7.75E-11	3.11E-09	14.0611
IGHV1OR	-1.8242	#NAME?	-6.7263	8.11E-11	3.24E-09	13.9631
IGHGP	-1.8989	4.89973	-6.6933	8.82E-11	3.52E-09	13.8578
AC14238	-1.6805	#NAME?	-6.699	9.68E-11	3.85E-09	13.7955
IGHA2	-2.0176	6.10444	-6.6659	1.04E-10	4.13E-09	13.6964
IGLV2-18	-2.0212	#NAME?	-6.6491	1.20E-10	4.72E-09	13.5668
IGHJ3	-1.8024	#NAME?	-6.644	1.26E-10	4.95E-09	13.5206
IGLV3-27	-2.327	#NAME?	-6.6357	1.26E-10	4.97E-09	13.51
IGHV3-35	-1.6878	#NAME?	-6.6329	1.43E-10	5.59E-09	13.4114
IGHV1OR	-1.7508	#NAME?	-6.6369	1.46E-10	5.70E-09	13.3986
IGHG2	-1.9793	8.067	-6.6077	1.48E-10	5.77E-09	13.3553
ART4	-1.5159	#NAME?	-6.6113	1.51E-10	5.89E-09	13.34
IGKV1OR	-1.7966	#NAME?	-6.6267	1.53E-10	5.95E-09	13.3506
IGLV10-5	-2.1449	#NAME?	-6.5679	2.00E-10	7.70E-09	13.0723
IGHV3-43	-2.0008	#NAME?	-6.5457	2.19E-10	8.39E-09	12.9769
IGHV1-46	-1.9844	#NAME?	-6.5155	2.57E-10	9.79E-09	12.8169
IGKV1-12	-1.7336	#NAME?	-6.4923	3.06E-10	1.16E-08	12.6519
IGHV3-11	-2.0703	#NAME?	-6.4568	3.70E-10	1.39E-08	12.4651
IGLV4-60	-2.2842	#NAME?	-6.4488	3.98E-10	1.49E-08	12.3975
AC03410	-1.6585	#NAME?	-6.4405	5.01E-10	1.86E-08	12.2166
IGKV2D-	-2.1787	#NAME?	-6.4043	5.05E-10	1.87E-08	12.163
IGKV2D-	-2.0701	#NAME?	-6.4072	5.15E-10	1.91E-08	12.1492
IGHV3-66	-1.9759	#NAME?	-6.3764	5.85E-10	2.15E-08	12.0173
KEL	-1.5216	#NAME?	-6.3696	6.08E-10	2.23E-08	11.979
ACKR1	-1.5987	#NAME?	-6.3496	6.81E-10	2.49E-08	11.8679
IGKV1D-	-1.9092	#NAME?	-6.3709	6.98E-10	2.55E-08	11.8759
IGKV1D-	-2.1738	#NAME?	-6.3626	7.41E-10	2.69E-08	11.8261
IGHV1OR	-1.8048	#NAME?	-6.3197	8.55E-10	3.09E-08	11.656
AC10356	-1.5245	#NAME?	-6.3499	8.93E-10	3.22E-08	11.6705
IGKV2-28	-1.8503	#NAME?	-6.3149	9.58E-10	3.43E-08	11.5669
AC13506	-1.5734	#NAME?	-6.308	9.71E-10	3.47E-08	11.5465
IGKV2OR	-1.7708	#NAME?	-6.2922	1.09E-09	3.86E-08	11.4376
IGHV6-1	-1.7233	#NAME?	-6.2367	1.36E-09	4.77E-08	11.2028
IGLV1-36	-2.1057	#NAME?	-6.2284	1.38E-09	4.85E-08	11.1801
THEMIS	-1.5532	-1.078	-6.2164	1.47E-09	5.13E-08	11.1225
IGKV2D-	-1.8394	#NAME?	-6.2226	1.60E-09	5.56E-08	11.0619
IGHV3-49	-1.9929	#NAME?	-6.2	1.61E-09	5.61E-08	11.0294
KLHDC7B	-1.5259	0.7426	-6.1515	2.12E-09	7.28E-08	10.7628
IGLC7	-2.0704	#NAME?	-6.1405	2.33E-09	7.95E-08	10.6778
IGLV3-16	-1.7236	#NAME?	-6.1467	2.35E-09	8.00E-08	10.6792
AC13642	-1.6076	#NAME?	-6.1421	2.51E-09	8.51E-08	10.621
IGLV2-28	-1.5111	#NAME?	-6.1252	2.83E-09	9.52E-08	10.52
IGLV1-50	-1.6225	#NAME?	-6.0694	3.49E-09	1.16E-07	10.29
IGKV1-13	-1.7162	#NAME?	-6.0623	4.45E-09	1.47E-07	10.107
IGKV1D-	-2.0123	#NAME?	-6.0311	4.47E-09	1.47E-07	10.0529
IGHV4-39	-1.9649	5.58969	-6.0174	4.52E-09	1.49E-07	10.029
PTHLH	1.87464	4.46411	5.99169	5.22E-09	1.71E-07	9.88955
LINC0089	1.7384	#NAME?	6.00068	5.52E-09	1.80E-07	9.9044
IGLV3-12	-1.5993	#NAME?	-5.9927	5.68E-09	1.85E-07	9.83264
IGHV2-5	-1.8545	#NAME?	-5.9616	6.36E-09	2.06E-07	9.70778
IGHV1-45	-1.9442	#NAME?	-5.9395	7.20E-09	2.32E-07	9.58754
AC13487	-1.6117	#NAME?	-5.9432	7.54E-09	2.43E-07	9.55656
IGKV6-21	-2.0248	#NAME?	-5.929	7.69E-09	2.47E-07	9.52193
IGHV2-26	-1.9166	#NAME?	-5.8995	8.84E-09	2.82E-07	9.38187
USH1G	1.65713	#NAME?	5.89297	9.07E-09	2.89E-07	9.35769
IGHV3-19	-1.5117	#NAME?	-5.9022	9.49E-09	3.02E-07	9.3386
IGLV7-46	-1.8075	3.21076	-5.871	1.02E-08	3.22E-07	9.24255
FDCSP	-2.4102	#NAME?	-5.8518	1.17E-08	3.67E-07	9.11584
IGHV1-69	-2.0205	#NAME?	-5.8052	1.47E-08	4.57E-07	8.88779
IGKV1D-	-1.701	#NAME?	-5.8417	1.51E-08	4.67E-07	8.93121
IGKV5-2	-1.8094	#NAME?	-5.7762	1.79E-08	5.52E-07	8.70987
IGKV1D-	-1.5467	#NAME?	-5.7743	1.87E-08	5.76E-07	8.6771
IGHV1-12	-1.6236	#NAME?	-5.7706	1.89E-08	5.80E-07	8.66427
IGHV3-20	-1.8237	#NAME?	-5.7575	1.95E-08	5.97E-07	8.62097
IGHJ2	-1.6116	#NAME?	-5.7021	2.69E-08	8.11E-07	8.31514
IGHV2-70	-2.0639	#NAME?	-5.6544	3.47E-08	1.03E-06	8.07491
IGKV2D-	-1.6528	#NAME?	-5.6796	3.66E-08	1.09E-06	8.08302
AC13506	-1.5566	#NAME?	-5.5973	6.48E-08	1.87E-06	7.59073
IGHV4-4	-1.7228	#NAME?	-5.4953	7.85E-08	2.24E-06	7.27781
IGHV1-3	-1.7576	#NAME?	-5.4339	1.09E-07	3.07E-06	6.96954
IGKV6D-	-1.9152	#NAME?	-5.381	1.48E-07	4.08E-06	6.67767
PLA2G2A	-1.6452	#NAME?	-5.3628	1.53E-07	4.21E-06	6.62754
SAA2-SA	-1.5919	#NAME?	-5.3277	1.89E-07	5.14E-06	6.45163
IGLV5-37	-1.8602	#NAME?	-5.3295	1.96E-07	5.33E-06	6.42518
IGHV3-64	-1.6741	#NAME?	-5.2897	2.22E-07	6.00E-06	6.26651
IGLV1-41	-1.6923	#NAME?	-5.2536	2.79E-07	7.42E-06	6.06703
BCORP1	-1.5315	#NAME?	-5.1075	6.48E-07	1.64E-05	5.40075
ADH1B	-1.6366	#NAME?	-4.8027	2.35E-06	5.39E-05	4.00024
EMILIN3	1.5689	#NAME?	4.5975	6.02E-06	0.00013	3.09997
TSPY2	-4.9523	#NAME?	-5.1705	6.21E-06	0.00013	3.78337
FAM216B	-1.5862	#NAME?	-4.4568	1.15E-05	0.00023	2.50068
FREM2	1.61392	#NAME?	4.27198	2.51E-05	0.00045	1.73954
IGHD	-1.6018	2.29871	-4.1572	4.07E-05	0.00069	1.27796
AP00160	-3.8584	#NAME?	-4.853	6.22E-05	0.001	1.40833
NTS	2.11127	#NAME?	3.72135	0.00023	0.00308	-0.3545
SLC18A3	1.61092	#NAME?	3.71255	0.00025	0.00322	-0.2989
AL031767	-4.9879	#NAME?	-4.4196	0.00027	0.00351	-0.0127
TMEM27	1.51575	#NAME?	3.71327	0.00029	0.00371	-0.0517
TMPRSS1	1.71464	#NAME?	3.64434	0.00031	0.00393	-0.6303
AL161431	1.63904	#NAME?	3.62822	0.00033	0.00414	-0.6823
UGT1A7	1.72916	#NAME?	3.56289	0.00042	0.00509	-0.8936
DEFA11P	-5.2733	#NAME?	-4.7606	0.0005	0.00582	-0.6006
CCAT1	1.5695	#NAME?	3.51688	0.0005	0.00583	-1.0591
GSTA9P	1.61096	#NAME?	3.48878	0.00056	0.0064	-1.1238
AC09286	-1.8722	#NAME?	-4.1892	0.00111	0.01131	-0.776
PROKR2	1.76243	#NAME?	3.37154	0.00112	0.01141	-1.2383
MPPED1	1.50416	#NAME?	3.23679	0.00134	0.01314	-1.9304
SFTPC	-1.7268	#NAME?	-3.196	0.00152	0.01463	-2.1036
AC00715	-2.4619	#NAME?	-3.3016	0.00158	0.01507	-1.2474
TNR-IT1	5.08159	#NAME?	5.57739	0.00166	0.01569	-2.3972
SPANXA1	-6.746	#NAME?	-3.8721	0.00175	0.01642	-1.8343
RBMY1A1	2.12548	#NAME?	3.68232	0.00207	0.01888	-1.2521
IFNA12P	3.74394	#NAME?	4.75664	0.00232	0.02069	-2.3253
AC10886	-2.0004	#NAME?	-3.1358	0.00259	0.02275	-1.8613
RN7SL61	-3.2886	#NAME?	-3.3119	0.00266	0.02324	-1.6579
RNU6-18	-3.4507	#NAME?	-3.786	0.0027	0.02352	-2.1065
ADH7	1.50443	#NAME?	2.96487	0.00324	0.02721	-2.7929
ZNF734P	-2.8572	#NAME?	-3.3652	0.0033	0.02763	-1.7064
AL845311	-2.147	#NAME?	-3.1265	0.00335	0.02791	-1.8179
XIST	-1.9887	#NAME?	-2.9289	0.00368	0.03011	-2.7948
AC11474	4.08225	#NAME?	4.66134	0.00393	0.03162	-2.6528
APOA4	-1.9337	#NAME?	-3.0081	0.00402	0.03225	-2.112
AC10546	1.64152	#NAME?	2.86907	0.00448	0.03511	-2.9168
AC11828	2.40525	#NAME?	2.87965	0.00494	0.03797	-2.2863
DEFA7P	-6.3862	#NAME?	-4.3629	0.00533	0.04025	-2.7563
KRTAP19	-1.6668	#NAME?	-2.9058	0.0061	0.04476	-2.3101
AC09194	2.49679	#NAME?	3.38616	0.0063	0.04589	-2.15

FCRL2	-1.5327	#NAME?	-4.5793	1.15E-05	0.00031	2.82256
FOXC2	1.68729	-0.993	4.55293	1.26E-05	0.00033	2.71962
DLX2	1.85176	#NAME?	4.53199	1.44E-05	0.00037	2.6207
TENM3-A	1.51903	#NAME?	4.49513	1.60E-05	0.00041	2.49366
ALPP	-2.255	#NAME?	-4.5087	1.62E-05	0.00042	2.54863
OR7E22P	-1.7546	#NAME?	-4.514	1.74E-05	0.00044	2.50473
CPA4	2.22552	-1.0211	4.46586	1.79E-05	0.00045	2.38501
AP00160	-3.7317	#NAME?	-8.0754	1.83E-05	0.00046	1.3841
MME	1.51377	0.91476	4.41729	2.17E-05	0.00054	2.20037
AC00799	-1.6573	#NAME?	-4.4437	2.31E-05	0.00056	2.27041
IGLV8-61	-2.0892	4.45954	-4.3491	2.85E-05	0.00068	1.9437
SHISA2	1.96987	1.73303	4.32914	3.08E-05	0.00072	1.86895
CR2	-1.9358	#NAME?	-4.299	3.49E-05	0.0008	1.75414
AK5	1.53867	#NAME?	4.26643	3.98E-05	0.0009	1.63146
LAMA1	1.84028	-0.5121	4.21627	4.79E-05	0.00104	1.45178
SLC26A4	-1.7187	#NAME?	-4.1946	5.37E-05	0.00115	1.36789
KRT17	2.03287	8.62451	4.12337	6.84E-05	0.00141	1.11456
PPP1R9A	-1.6486	#NAME?	-4.1229	6.88E-05	0.00141	1.11114
LINC0205	-1.6086	#NAME?	-4.1341	6.94E-05	0.00142	1.143
AC06922	-2.0831	#NAME?	-4.9406	7.69E-05	0.00155	1.62695
CRB2	-1.5728	#NAME?	-4.0728	8.44E-05	0.00166	0.93497
CDH8	1.74583	#NAME?	4.06891	8.52E-05	0.00168	0.91871
AC24508	3.48846	#NAME?	5.41707	8.69E-05	0.0017	0.77978
AC01189	2.31754	#NAME?	5.18569	8.71E-05	0.0017	0.85313
SFTPC	-3.2442	#NAME?	-4.0531	9.01E-05	0.00175	0.86432
APCDD1L	1.56797	#NAME?	4.04326	9.30E-05	0.0018	0.82681
AICDA	-1.5642	#NAME?	-4.0276	0.0001	0.00196	0.7804
CAPN8	-2.0669	#NAME?	-3.9997	0.00011	0.00208	0.67594
AMTN	2.7139	#NAME?	3.99303	0.00012	0.0022	0.64291
PNMA3	-1.574	-2.0451	-3.9757	0.00012	0.00223	0.59013
IGLV2-23	-1.5183	7.23687	-3.9729	0.00012	0.00225	0.58051
LINC0160	1.59108	#NAME?	3.94236	0.00014	0.00247	0.47273
GOLT1A	-1.5036	#NAME?	-3.9442	0.00014	0.00247	0.48099
PNMA8A	1.59063	0.26663	3.9156	0.00015	0.00267	0.38105
RORC	-1.5688	-1.1357	-3.902	0.00016	0.00278	0.33398
HSPB3	1.76885	#NAME?	3.89972	0.00016	0.00282	0.32308
C4BPB	-1.6603	#NAME?	-3.9	0.00016	0.00282	0.33608
OR2C3	2.49506	#NAME?	4.29113	0.00019	0.00323	0.74521
AC00826	-2.0871	#NAME?	-3.9417	0.0002	0.00334	0.42283
FAM3B	-1.8512	0.18831	-3.8351	0.0002	0.00337	0.10476
KRT16P4	1.658	#NAME?	3.86939	0.00021	0.00345	0.21295
COL17A1	1.85762	4.41264	3.82329	0.00021	0.00348	0.06455
LGR5	1.9367	#NAME?	3.82051	0.00021	0.00352	0.05435
SPATA8-A	4.12123	#NAME?	5.87961	0.00022	0.00361	-0.9307
IL17A	-2.3704	#NAME?	-3.916	0.00023	0.00381	0.3937
IL1A	1.71063	1.2853	3.79086	0.00023	0.00384	-0.0452
KRT75	2.24589	#NAME?	3.76674	0.00026	0.00415	-0.1289
FIBCD1	1.59928	#NAME?	3.76216	0.00026	0.00417	-0.1424
SLC13A5	1.88265	#NAME?	3.75824	0.00026	0.00422	-0.1555
AL04964	1.73039	#NAME?	3.79025	0.00028	0.00441	-0.0584
CYP4F29P	-1.6745	#NAME?	-3.7441	0.00028	0.00443	-0.1959
KCNA1	1.55148	#NAME?	3.75489	0.00028	0.00444	-0.1681
DLX1	1.78362	#NAME?	3.74177	0.00028	0.00447	-0.2043
CLDN18	-1.9225	-0.612	-3.7354	0.00029	0.00449	-0.2311
AC13949	3.78758	#NAME?	5.08287	0.00034	0.00513	-0.9252
COL2A1	1.65526	-3.2287	3.6683	0.00036	0.00547	-0.4534
SLC6A2	1.80915	#NAME?	3.6656	0.00037	0.0056	-0.4643
LMO3	-1.6993	-1.3825	-3.6597	0.00037	0.00561	-0.4816
IGKV1-27	-1.5191	5.09013	-3.6366	0.00041	0.00599	-0.5574
MS4A15	-1.6123	#NAME?	-3.6207	0.00043	0.00633	-0.6101
MSLN	-1.9418	1.53628	-3.6165	0.00044	0.00633	-0.6227
LINC0062	1.76906	#NAME?	3.62311	0.00044	0.00637	-0.5816
PPP1R1A	1.6584	#NAME?	4.55107	0.00044	0.00637	-0.3052
ADCY2	1.64963	#NAME?	3.59304	0.00047	0.00678	-0.6991
PTF1A	1.99509	#NAME?	4.11148	0.00049	0.00696	-0.0194
S100A2	1.76643	7.38801	3.56948	0.00051	0.00719	-0.7747
AC02639	3.37445	#NAME?	5.21468	0.00052	0.00727	-1.2828
SCGB3A1	-2.0078	2.42552	-3.5151	0.00062	0.00833	-0.9487
AL589987	-2.0397	#NAME?	-3.6539	0.00063	0.00843	-0.4884
IGLV3-21	-1.502	7.71403	-3.508	0.00063	0.00848	-0.971
AL356277	2.86001	#NAME?	4.16663	0.00063	0.00849	-0.2135
RNASE7	1.88937	#NAME?	3.46467	0.00074	0.00965	-1.1027
EMX2OS	1.67066	#NAME?	3.45485	0.00082	0.01049	-1.1141
KIR3DL3	-1.5395	#NAME?	-3.6115	0.00085	0.01079	-0.6904
SFTPD	-1.8277	3.11157	-3.4124	0.00087	0.01099	-1.2708
AC10591	1.9095	#NAME?	4.34662	0.00092	0.01143	-0.5643
IL36B	1.55988	#NAME?	3.40604	0.00096	0.01185	-1.2587
LINC0124	7.13778	#NAME?	8.27321	0.00096	0.01185	-2.5918
KBTBD12	-1.556	#NAME?	-3.3788	0.001	0.01219	-1.3582
FAT2	1.58324	2.28186	3.37043	0.00101	0.01223	-1.4003
C1orf61	-1.5269	#NAME?	-3.3717	0.00101	0.01223	-1.3863
HEPHL1	1.90491	-1.5898	3.35368	0.00106	0.01278	-1.4515
LINC0199	-1.5971	#NAME?	-3.397	0.00109	0.013	-1.1822
RP11-706	-1.7056	#NAME?	-3.3425	0.00113	0.01342	-1.4727
IGLV3-10	-1.5214	5.88455	-3.3014	0.00126	0.0146	-1.6103
TMEM212	-1.5042	#NAME?	-3.3208	0.0013	0.01488	-1.5031
CNN2P11	-1.7672	#NAME?	-4.5569	0.00131	0.01493	-0.9811
MAGEA6	2.77282	#NAME?	3.29712	0.00134	0.01518	-1.5763
KRT16P6	1.93149	#NAME?	3.28691	0.00134	0.01523	-1.6364
RNU6-56	1.73726	#NAME?	3.81664	0.00136	0.01535	-1.0905
AL391358	2.11169	#NAME?	4.22137	0.00138	0.01558	-1.6346
AC10808	4.07035	#NAME?	4.50712	0.0014	0.01574	-1.7328
SFTPA2	-2.5359	#NAME?	-3.2677	0.00141	0.01581	-1.7109
KRT14	2.49813	5.17192	3.25903	0.00145	0.01611	-1.7373
AC09752	1.68691	#NAME?	4.28449	0.00154	0.01688	-1.0369
CYP2B7P	-1.5372	-0.3911	-3.2226	0.00163	0.01763	-1.8456
KRT7-AS	-1.5475	#NAME?	-3.2195	0.00165	0.01783	-1.8482
C4BPA	-2.0753	#NAME?	-3.1862	0.00183	0.01939	-1.9474
AL031713	2.58466	#NAME?	4.15303	0.0019	0.01986	-1.3901
IL36G	1.98474	#NAME?	3.16575	0.00196	0.02033	-2.0115
LINC0188	1.94119	#NAME?	3.34345	0.00197	0.02045	-1.4517
AC24285	3.66126	#NAME?	4.46222	0.00199	0.02061	-1.9629
OR6C66P	-2.2384	#NAME?	-4.7265	0.002	0.02067	-1.6043
SLC7A10	-1.5559	#NAME?	-3.1642	0.00204	0.02095	-1.9698
LINC0123	2.89845	#NAME?	4.70533	0.00205	0.02106	-2.0602
SFTPA1	-2.4511	4.56822	-3.1218	0.00224	0.02258	-2.1396
MLLT10P	3.81678	#NAME?	4.04707	0.00225	0.02263	-1.9257
LINC0193	-1.9869	#NAME?	-3.7069	0.0023	0.023	-1.2969
GJB6	1.80461	3.80248	3.11311	0.00231	0.02304	-2.1645
TSPY2	-4.6435	#NAME?	-3.6088	0.00232	0.02312	-1.3225
IL36RN	1.63246	#NAME?	3.10848	0.00235	0.02337	-2.1751
SFTPA3P	2.69863	#NAME?	3.75202	0.00237	0.02346	-1.888
XIST	-3.1404	#NAME?	-3.109	0.00248	0.02441	-2.1036
AC11756	3.53355	#NAME?	4.89139	0.00252	0.02464	-2.2607
AC12134	1.65499	#NAME?	3.63743	0.00264	0.02549	-1.4189
GGTLC1	-1.6175	#NAME?	-3.0783	0.00269	0.0259	-2.1715
PEBP4	-1.6415	#NAME?	-3.0629	0.0027	0.02596	-2.3067
NAPSA	-1.8242	2.823	-3.0588	0.00273	0.02617	-2.3193
IGFL3	1.68936	#NAME?	3.06794	0.00277	0.02643	-2.2646
AC23722	-2.2844	#NAME?	-3.3194	0.00285	0.02701	-1.603
FTH1P14	2.90133	#NAME?	3.54851	0.00287	0.02715	-1.4988
TRIML2	1.5364	#NAME?	3.06636	0.00295	0.02773	-2.1826
DIPK1C	1.55445	#NAME?	3.03398	0.00302	0.02818	-2.3737
GOLGA8F	1.51581	#NAME?	3.35867	0.0031	0.02874	-1.6325
CYP4B1	-1.6633	#NAME?	-3.0183	0.0031	0.02875	-2.4274
AQP4	-1.9333	#NAME?	-3.0179	0.00311	0.02882	-2.4216
BX088651	2.50567	#NAME?	3.96637	0.00315	0.02906	-1.7519
SFTA3	-1.8708	#NAME?	-3.0132	0.00319	0.02937	-2.419
LINC0258	-1.5406	#NAME?	-3.0353	0.00329	0.02999	-2.2579
CHEK2P5	-3.7847	#NAME?	-5.0995	0.00341	0.03081	-2.5324
RN7SKP2	-1.8965	#NAME?	-3.5976	0.00358	0.03197	-1.656
AC01876	1.75385	#NAME?	3.11884	0.00367	0.03263	-1.9582
AL359792	4.27871	#NAME?	3.36113	0.00392	0.03422	-1.7738
DEFA7P	-8.3936	#NAME?	-7.5319	0.00396	0.03449	-3.306
AL592309	-1.9107	#NAME?	-3.1777	0.00403	0.0349	-1.9333
MRPL50P	-3.6311	#NAME?	-4.9014	0.00407	0.03509	-2.3311
RBMY3AF	4.61496	#NAME?	4.8143	0.0044	0.03703	-2.6145
SBSN	1.93227	1.4064	2.90025	0.00442	0.03718	-2.7575
SPANXA1	-8.0853	#NAME?	-7.2552	0.00444	0.03728	-3.3171
RPL31P41	-1.7483	#NAME?	-3.681	0.0049	0.03991	-1.9302
AC01045	4.70526	#NAME?	5.37751	0.0051	0.04105	-2.8905
LINC0177	1.63076	#NAME?	2.94617	0.00539	0.04275	-2.4136
AL353093	2.37712	#NAME?	3.91036	0.00554	0.04356	-2.5058
SFTA2	-1.7745	#NAME?	-2.8203	0.00564	0.04412	-2.9497
KRT5	1.63668	8.79876	2.81201	0.00574	0.04464	-2.9927
LINC0103	-2.493	#NAME?	-3.8669	0.00587	0.04544	-2.5333
KRT23	1.6115	#NAME?	2.79802	0.00598	0.04612	-3.028

LGI2	1.99511	-0.5947	12.0589	1.36E-28	7.11E-27	54.0937
SSTR3	1.84331	#NAME?	12.1006	1.40E-28	7.32E-27	54.0748
XPNPEP2	1.97293	#NAME?	12.0615	1.50E-28	7.82E-27	54.0007
HTR2A	1.92913	#NAME?	12.0727	1.54E-28	8.01E-27	53.9797
ADAMTS:	1.66521	#NAME?	12.0406	1.84E-28	9.57E-27	53.7965
GZMB	1.68713	2.28254	12.0224	1.87E-28	9.71E-27	53.7761
JPH2	1.72309	-2.1573	12.018	1.94E-28	1.01E-26	53.7381
EOMES	1.68053	-1.3023	12.014	2.01E-28	1.04E-26	53.7031
ARHGAP(	1.54044	-2.2066	12.0057	2.17E-28	1.12E-26	53.6306
ITGB3	1.69744	#NAME?	11.9997	2.34E-28	1.20E-26	53.5556
TNFSF14	1.51602	-1.7066	11.9902	2.48E-28	1.27E-26	53.4961
HLA-DOF	1.86781	#NAME?	11.9846	2.67E-28	1.36E-26	53.4246
COL14A1	2.04912	1.17546	11.9699	2.97E-28	1.51E-26	53.3195
FCRLA	2.30532	-1.0473	11.9664	3.06E-28	1.55E-26	53.2889
TNFRSF8	1.76128	-1.4365	11.9648	3.10E-28	1.57E-26	53.2757
MGP	1.69711	4.49211	11.9547	3.39E-28	1.71E-26	53.1881
MAP1LC3	2.10372	#NAME?	12.0059	3.82E-28	1.93E-26	53.0885
UBD	2.2207	2.21003	11.9324	4.12E-28	2.07E-26	52.9942
GZMM	1.72089	#NAME?	11.9321	4.23E-28	2.12E-26	52.9693
CPM	1.71016	1.70016	11.9211	4.55E-28	2.28E-26	52.8962
ADRA2A	2.06038	-0.7767	11.9188	4.64E-28	2.33E-26	52.8766
NR4A3	1.84602	-0.3376	11.9055	5.21E-28	2.61E-26	52.7607
DCSTAMI	2.1742	#NAME?	11.9131	5.47E-28	2.74E-26	52.7162
CCN5	1.97015	-0.6146	11.8989	5.52E-28	2.76E-26	52.7038
TRAV27	1.61059	#NAME?	12.1298	5.53E-28	2.76E-26	52.7688
ALPK2	2.08843	-2.5189	11.8946	5.73E-28	2.85E-26	52.6667
TMIGD2	1.79102	#NAME?	11.8862	6.92E-28	3.43E-26	52.4841
ZNF385D	1.66837	#NAME?	11.8544	8.52E-28	4.21E-26	52.2756
EMB	1.51278	1.86772	11.8288	1.02E-27	5.01E-26	52.0975
FASLG	1.92005	#NAME?	11.83	1.05E-27	5.18E-26	52.0648
TRAV23D	1.73473	#NAME?	12.0994	1.08E-27	5.29E-26	52.1237
HTRA3	1.51902	3.96769	11.8219	1.08E-27	5.31E-26	52.0379
CXCL9	2.37073	3.52403	11.8084	1.22E-27	5.96E-26	51.9217
TRAV6	1.66637	#NAME?	12.0993	1.29E-27	6.32E-26	51.9544
IL27	1.5534	#NAME?	11.8574	1.34E-27	6.53E-26	51.8468
ANKRD55	1.77513	#NAME?	11.8766	1.42E-27	6.93E-26	51.7933
CX3CR1	1.82786	-1.6754	11.7843	1.50E-27	7.29E-26	51.7139
DLG2	1.56854	-2.7485	11.783	1.52E-27	7.37E-26	51.7024
SPIB	2.46949	-1.2697	11.777	1.60E-27	7.74E-26	51.6509
SIGLEC22	1.59476	#NAME?	11.9619	1.67E-27	8.09E-26	51.6634
LINC0070	1.77151	#NAME?	11.7617	1.87E-27	9.03E-26	51.4982
MYL3	1.63808	#NAME?	11.8012	2.11E-27	1.02E-25	51.3924
TRBV12-:	1.64687	#NAME?	12.0129	2.60E-27	1.25E-25	51.2582
KCNN3	1.60359	-1.3792	11.7166	2.70E-27	1.30E-25	51.1307
AP003071	1.64161	#NAME?	11.7324	2.81E-27	1.35E-25	51.0985
Z95114.3	1.58916	#NAME?	11.7257	3.05E-27	1.46E-25	51.0181
IGLV2-14	2.94943	6.85793	11.6948	3.26E-27	1.56E-25	50.9434
AC004847	1.55354	-0.9206	11.6917	3.35E-27	1.60E-25	50.9165
AGTR1	2.00998	#NAME?	11.6924	3.63E-27	1.73E-25	50.8397
CCL8	1.97087	0.42482	11.6809	3.68E-27	1.75E-25	50.8238
AC243960	1.52692	#NAME?	11.7295	3.90E-27	1.85E-25	50.7844
IKZF3	1.50385	0.43057	11.6567	4.54E-27	2.15E-25	50.6162
CD22	1.88359	-0.9515	11.6463	4.97E-27	2.35E-25	50.5268
HRH2	1.91086	-1.267	11.6361	5.43E-27	2.56E-25	50.4395
ITGA8	1.69654	-0.6982	11.623	6.07E-27	2.86E-25	50.3278
CCDC170	1.96749	-1.9821	11.617	6.40E-27	3.01E-25	50.276
HMCN1	1.67803	-0.0023	11.6168	6.41E-27	3.01E-25	50.2742
LINC0234	1.89234	#NAME?	11.6201	6.64E-27	3.11E-25	50.2419
NAV3	1.65137	-2.3835	11.6026	7.25E-27	3.39E-25	50.1529
ADAMTS5	1.59164	-1.1805	11.6019	7.29E-27	3.41E-25	50.1472
IGKV1-9	2.95085	5.01997	11.5863	8.34E-27	3.89E-25	50.0135
HTRA4	1.79897	-1.3922	11.5859	8.37E-27	3.90E-25	50.0103
ACTG2	1.75539	1.37059	11.5736	9.31E-27	4.31E-25	49.9052
PLD4	1.55889	-1.9118	11.5735	9.32E-27	4.31E-25	49.9043
GAS1	1.80468	1.62168	11.5731	9.35E-27	4.33E-25	49.9009
LGALS2	1.55335	0.55599	11.5691	9.68E-27	4.48E-25	49.866
IGKV1-3:	2.97335	#NAME?	11.6421	1.27E-26	5.87E-25	49.6291
NCR1	1.84572	#NAME?	11.5657	1.35E-26	6.19E-25	49.5515
LAMA2	1.56456	0.76421	11.5272	1.39E-26	6.38E-25	49.5089
HSPB7	1.73299	-1.4178	11.5254	1.41E-26	6.48E-25	49.4929
CXCR5	1.87956	#NAME?	11.6002	1.81E-26	8.27E-25	49.284
C11orf96	1.5365	2.334	11.4792	2.10E-26	9.54E-25	49.0997
CCL21	2.15276	4.41169	11.4762	2.15E-26	9.79E-25	49.0737
LSAMP	1.77583	-0.1601	11.4741	2.19E-26	9.94E-25	49.0564
IGHV4-5!	2.54704	#NAME?	11.4905	2.20E-26	9.97E-25	49.0581
TMEM23(	1.5075	-3.6207	11.4696	2.28E-26	1.03E-24	49.0174
CD79A	2.41935	2.82163	11.4592	2.49E-26	1.13E-24	48.9292
TRGV4	1.57103	#NAME?	11.5895	2.62E-26	1.18E-24	48.9254
CDK15	1.52529	#NAME?	11.4429	2.99E-26	1.35E-24	48.7521
IGLV1-40	2.92832	6.88753	11.418	3.55E-26	1.59E-24	48.5786
IGHV3-30	2.8458	#NAME?	11.414	3.75E-26	1.68E-24	48.5257
IGHV3-5:	3.03542	#NAME?	11.4157	4.00E-26	1.78E-24	48.4638
COL11A1	2.4352	1.86167	11.404	4.00E-26	1.78E-24	48.4598
SLIT3	1.89158	-0.1329	11.3953	4.31E-26	1.92E-24	48.3866
APOC2	1.58582	#NAME?	11.4324	4.67E-26	2.07E-24	48.3238
C1QTNF7	1.89323	#NAME?	11.3877	4.79E-26	2.12E-24	48.2843
RIPOR2	1.56293	-0.4037	11.3745	5.16E-26	2.28E-24	48.2098
C11orf21	1.64504	-2.6568	11.3729	5.22E-26	2.31E-24	48.1967
IGLV3-1	2.88024	#NAME?	11.3711	5.41E-26	2.38E-24	48.1626
S100B	1.77969	0.93501	11.3677	5.46E-26	2.40E-24	48.1527
IGHV3-2:	2.71124	#NAME?	11.3638	5.76E-26	2.53E-24	48.1007
IGLV4-69	2.97518	#NAME?	11.3492	6.65E-26	2.92E-24	47.9585
IGLL5	2.77313	4.4501	11.3308	7.49E-26	3.28E-24	47.8399
TREM1	1.79751	0.88223	11.3267	7.76E-26	3.39E-24	47.805
RGS4	1.72815	-0.315	11.325	7.87E-26	3.44E-24	47.7907
IGLV1-44	2.82172	#NAME?	11.3259	7.96E-26	3.48E-24	47.7801
IGKV1-27	2.99896	#NAME?	11.3254	8.16E-26	3.56E-24	47.7569
CLEC12A	1.88573	-0.8801	11.3105	8.91E-26	3.88E-24	47.6679
LINC0244	2.10111	#NAME?	11.3135	9.39E-26	4.08E-24	47.619
TRAV5	1.59738	#NAME?	11.5141	1.00E-25	4.35E-24	47.6241
C5orf46	1.92604	#NAME?	11.3049	1.01E-25	4.37E-24	47.5468
LINC0212	1.61242	#NAME?	11.4811	1.10E-25	4.76E-24	47.5252
LINC0060	1.61631	#NAME?	11.288	1.17E-25	5.02E-24	47.4043
PLA2G2D	2.56269	0.19716	11.267	1.29E-25	5.54E-24	47.3007
OGN	2.31529	#NAME?	11.2693	1.32E-25	5.64E-24	47.2838
MMRN1	1.99898	-1.1335	11.2579	1.40E-25	5.98E-24	47.2239
JAKMIP1	1.71326	#NAME?	11.2582	1.42E-25	6.07E-24	47.2083
ADGRE4P	1.71627	#NAME?	11.2554	1.48E-25	6.33E-24	47.1665
PARP15	1.60404	-1.1366	11.2344	1.70E-25	7.27E-24	47.0261
CTC-251I	1.6099	0.85514	11.2297	1.77E-25	7.56E-24	46.9864
CXCL13	2.5608	3.39462	11.2276	1.81E-25	7.69E-24	46.9684
HAMP	1.75728	-1.8877	11.2263	1.83E-25	7.77E-24	46.9576
RTN1	1.73908	-0.9156	11.2209	1.91E-25	8.13E-24	46.9126
IGHV4-3:	2.8243	#NAME?	11.2191	1.98E-25	8.40E-24	46.8796
AC112721	1.71149	#NAME?	11.3141	2.06E-25	8.77E-24	46.8737
IGLV2-8	2.94718	#NAME?	11.2129	2.09E-25	8.85E-24	46.8268
IGHV4-28	2.60319	#NAME?	11.2124	2.13E-25	9.05E-24	46.8052
IGHV4-6:	2.81054	#NAME?	11.2259	2.14E-25	9.05E-24	46.8086
IGKV4-1	2.90508	7.30446	11.1925	2.43E-25	1.03E-23	46.673
COL12A1	1.85903	3.28367	11.187	2.55E-25	1.07E-23	46.627
TRAV24	1.56683	#NAME?	11.5206	2.66E-25	1.12E-23	46.704
CLNK	1.53749	#NAME?	11.1834	2.68E-25	1.13E-23	46.5792
LINC0148	1.5804	-2.0239	11.1766	2.78E-25	1.17E-23	46.5395
IGHV3-3:	2.94113	#NAME?	11.178	2.80E-25	1.18E-23	46.5333
IGKV3-15	2.67239	5.92874	11.1729	2.87E-25	1.20E-23	46.5089
DTNB-AS	2.01638	#NAME?	11.1952	2.88E-25	1.21E-23	46.5174
KCNE4	1.59906	0.28868	11.1718	2.90E-25	1.21E-23	46.4995
KCNIP1	1.69818	#NAME?	11.2362	2.95E-25	1.23E-23	46.5069
AC037198	1.64785	#NAME?	11.1819	3.04E-25	1.27E-23	46.459
SUCNR1	1.88883	-1.0199	11.1396	3.81E-25	1.59E-23	46.2289
TSPAN11	1.51657	-0.1279	11.1382	3.86E-25	1.61E-23	46.217
IGLV1-51	2.71692	6.32918	11.1282	4.20E-25	1.75E-23	46.1338
ADORA1	1.60138	-1.1357	11.1144	4.72E-25	1.96E-23	46.018
IGKV1D-I	2.65526	#NAME?	11.127	4.92E-25	2.04E-23	45.9818
FBP1	1.70503	3.37411	11.1056	5.08E-25	2.10E-23	45.9442
IGKV2-24	3.01152	#NAME?	11.1077	5.18E-25	2.14E-23	45.9267
TSHR	1.96559	#NAME?	11.1078	5.27E-25	2.18E-23	45.9103
CHRDL2	2.09609	-1.7646	11.0991	5.37E-25	2.21E-23	45.8898
ITIH3	1.59622	-3.2661	11.0893	5.83E-25	2.40E-23	45.8076
PIEZO2	1.79622	-1.584	11.0784	6.40E-25	2.63E-23	45.716
LPL	1.81463	0.87392	11.0688	6.94E-25	2.85E-23	45.6357
IGLV2-23	2.8131	6.24072	11.0609	7.42E-25	3.04E-23	45.5697
OR7E47P	1.85138	#NAME?	11.0501	8.27E-25	3.38E-23	45.4625
MZB1	2.37163	3.04731	11.0463	8.39E-25	3.43E-23	45.4478
AC010247	1.64864	#NAME?	11.0652	8.44E-25	3.45E-23	45.4505
AL442635	1.59722	#NAME?	11.2082	8.60E-25	3.51E-23	45.4893
TRBV7-3	1.66797	#NAME?	11.1449	8.73E-25	3.56E-23	45.4486
AL031846	1.55727	#NAME?	11.1203	8.97E-25	3.66E-23	45.4117
CSDC2	1.69874	-1.022	11.0382	8.98E-25	3.66E-23	45.3802
IGKV3-11	2.70171	7.05483	11.0322	9.45E-25	3.84E-23	45.3304
IGHV1-18	2.83759	6.269	11.0319	9.47E-25	3.84E-23	45.3278
TRBV24-:	1.67008	#NAME?	11.2545	9.87E-25	4.00E-23	45.3698
IGHV3-15	2.77918	#NAME?	11.0248	1.02E-24	4.15E-23	45.2513
LINC0191	1.79307	#NAME?	11.0517	1.12E-24	4.53E-23	45.1765
PLPP7	1.50917	-1.631	11.0116	1.12E-24	4.54E-23	45.1583
TRAV10	1.57824	#NAME?	11.2703	1.13E-24	4.57E-23	45.2478
RHEX	1.90893	#NAME?	11.0032	1.23E-24	4.94E-23	45.0714
IGHV5-5:	2.87202	6.55982	10.989	1.36E-24	5.46E-23	44.9699
TRGC2	1.74158	#NAME?	10.9897	1.38E-24	5.52E-23	44.959
ADAMTS9	1.72471	#NAME?	10.9931	1.49E-24	5.97E-23	44.8874
PPIAP39	1.69844	#NAME?	10.982	1.72E-24	6.88E-23	44.7423
AC087280	1.51206	#NAME?	11.0293	1.79E-24	7.12E-23	44.7268
FCRL5	2.45983	#NAME?	10.9569	1.81E-24	7.22E-23	44.6866
DPP4	1.85375	0.75818	10.949	1.90E-24	7.58E-23	44.6372
TRAV25	1.62339	#NAME?	11.1906	2.18E-24	8.67E-23	44.5945
KLRC1	1.71703	#NAME?	10.9308	2.34E-24	9.25E-23	44.4367
IGHV3-1:	3.00903	#NAME?	10.9365	2.35E-24	9.30E-23	44.4334
IGHV4-59	2.80433	#NAME?	10.9166	2.54E-24	1.00E-22	44.3515
IGLV3-25	2.81183	6.44075	10.9067	2.71E-24	1.07E-22	44.2854
IGHV3-66	2.86177	#NAME?	10.9087	2.76E-24	1.09E-22	44.2695
NALCN	1.84577	-2.766	10.9038	2.78E-24	1.10E-22	44.2617
PTGIS	1.89837	1.1248	10.8976	2.93E-24	1.15E-22	44.2103
IGHV6-1	2.67929	#NAME?	10.9254	2.98E-24	1.17E-22	44.2052
LINC0236	1.81518	-1.0397	10.8955	2.98E-24	1.17E-22	44.1931
LINC0121	1.98652	#NAME?	10.8841	3.51E-24	1.38E-22	44.0332
ZNF80	1.67511	#NAME?	10.9151	3.56E-24	1.39E-22	44.0349
DNAH8	1.7645	#NAME?	10.9236	4.02E-24	1.57E-22	43.9226
IGHA1	2.61879	9.85454	10.8432	4.62E-24	1.80E-22	43.7597
TRBV4-1	1.76963	#NAME?	10.9892	4.68E-24	1.82E-22	43.8037
MEF2C-A	1.57517	#NAME?	10.8811	4.71E-24	1.84E-22	43.7549
PPFIA2	1.67551	#NAME?	10.8574	4.79E-24	1.86E-22	43.7308
SELP	1.82236	-0.0709	10.8387	4.79E-24	1.87E-22	43.7223
LINC0040	1.82151	#NAME?	10.98	4.81E-24	1.87E-22	43.7743
IGLC2	2.43818	8.44427	10.8371	4.86E-24	1.89E-22	43.7089
AC142381	2.33659	#NAME?	10.8962	4.94E-24	1.92E-22	43.7168
IGHV2-70	3.00016	#NAME?	10.8502	5.17E-24	2.01E-22	43.655
IGKV1OR	2.70438	#NAME?	10.8197	5.72E-24	2.21E-22	43.5492
IGKV1-5	2.7575	6.97147	10.8165	5.77E-24	2.23E-22	43.5385
IGLV1-47	2.6974	5.72209	10.8048	6.36E-24	2.46E-22	43.442
IGLV1-50	2.3864	#NAME?	10.8249	6.50E-24	2.51E-22	43.4303
AC093583	1.53931	#NAME?	10.9484	6.97E-24	2.69E-22	43.4134
SSTR2	1.82878	-2.0975	10.7929	7.03E-24	2.71E-22	43.344
IGKV1-39	3.02253	#NAME?	10.8384	7.09E-24	2.73E-22	43.3535
IGKV1-6	2.68606	#NAME?	10.7896	7.34E-24	2.82E-22	43.301
IGKV3-20	2.59266	7.75194	10.7856	7.47E-24	2.87E-22	43.2835
SYNPO2	1.81333	-0.3384	10.7829	7.64E-24	2.93E-22	43.2616
IGKC	2.56349	9.42615	10.7777	7.98E-24	3.06E-22	43.2186
TENM3	1.84371	-0.867	10.7776	7.98E-24	3.06E-22	43.2176
CYTL1	1.89995	#NAME?	10.7825	8.06E-24	3.08E-22	43.2111
IGHV3-7:	2.78784	#NAME?	10.7838	8.38E-24	3.20E-22	43.1741
BHLHE41	1.59185	1.91655	10.7635	8.98E-24	3.43E-22	43.1015
PGM5	1.83134	-2.0925	10.7387	1.10E-23	4.19E-22	42.8976
ZNF804A	1.88478	#NAME?	10.7504	1.11E-23	4.19E-22	42.8997
IGHV3-74	2.71609	#NAME?	10.7305	1.22E-23	4.62E-22	42.7993
TRAV35	1.5201	#NAME?	11.0776	1.60E-23	6.01E-22	42.6785
VENTX	1.53065	-2.2289	10.6634	2.06E-23	7.71E-22	42.2785
AL139125	1.5073	#NAME?	10.6984	2.12E-23	7.93E-22	42.2651
KLRC4-KI	1.58866	#NAME?	10.7242	2.14E-23	7.97E-22	42.2709
IGKJ5	2.09986	#NAME?	10.7364	2.17E-23	8.08E-22	42.2634
MMP2-AS	1.95785	#NAME?	10.6725	2.29E-23	8.52E-22	42.184
CNR2	1.9439	#NAME?	10.6708	2.40E-23	8.92E-22	42.1387
FAM30A	2.23314	#NAME?	10.6347	2.66E-23	9.86E-22	42.0287
PTGER3	1.7371	-3.1498	10.6314	2.69E-23	9.96E-22	42.0168
IGHV1-46	2.75034	#NAME?	10.6088	3.29E-23	1.22E-21	41.8171
IGLV3-19	2.83057	#NAME?	10.6062	3.36E-23	1.24E-21	41.7955
MXRA5	1.57827	3.69064	10.5984	3.53E-23	1.30E-21	41.7462
TSPAN2	1.65015	-0.0915	10.5858	3.92E-23	1.44E-21	41.6436
AL356417	1.59742	#NAME?	10.6105	4.14E-23	1.52E-21	41.6014
RSPO1	1.73908	#NAME?	10.6164	4.22E-23	1.54E-21	41.5856
LINC0191	1.63365	#NAME?	10.6925	4.33E-23	1.58E-21	41.5915
NCAM2	1.92706	-1.7873	10.5633	4.72E-23	1.72E-21	41.4597
MMP9	1.82737	4.65174	10.5504	5.25E-23	1.91E-21	41.355
TRAV26-:	1.60479	#NAME?	10.7868	5.54E-23	2.01E-21	41.4034
BST1	1.50822	0.25655	10.5383	5.80E-23	2.10E-21	41.2558
DPEP1	1.98787	#NAME?	10.5383	5.89E-23	2.13E-21	41.2418
AC022164	1.68938	#NAME?	10.5861	6.10E-23	2.21E-21	41.2314
IL21-AS1	1.55815	#NAME?	10.6563	6.16E-23	2.23E-21	41.2482
CLEC7A	1.50226	1.99306	10.5292	6.25E-23	2.26E-21	41.1817
COL6A6	2.0755	-2.0217	10.5214	6.66E-23	2.40E-21	41.1186
IGHV3OR	2.23836	#NAME?	10.5929	7.04E-23	2.54E-21	41.0966
IGHV1OR	2.48192	#NAME?	10.5621	7.42E-23	2.67E-21	41.0358
LINC0187	1.5762	#NAME?	10.4997	8.34E-23	2.99E-21	40.8991
LINC0058	1.76405	#NAME?	10.5435	8.47E-23	3.04E-21	40.9019
HLA-DRB	1.83608	3.40732	10.491	8.56E-23	3.07E-21	40.871
GP5	1.50927	#NAME?	10.5254	8.72E-23	3.12E-21	40.8677
AC079325	1.61111	#NAME?	10.5244	9.09E-23	3.25E-21	40.8282
IGHV1-24	2.90385	#NAME?	10.4862	9.17E-23	3.28E-21	40.8039
AC026369	1.78341	#NAME?	10.5538	9.65E-23	3.45E-21	40.7848
RSAD2	1.66459	1.14853	10.4736	9.86E-23	3.52E-21	40.7302
AL691447	1.51378	#NAME?	10.5176	9.93E-23	3.54E-21	40.7418
IFNG-AS:	2.1037	#NAME?	10.5578	1.07E-22	3.79E-21	40.6943
AMPD1	1.93684	#NAME?	10.4781	1.16E-22	4.13E-21	40.5777
RANBP3L	1.71695	#NAME?	10.4592	1.18E-22	4.19E-21	40.5563
CSF3R	1.62472	0.82847	10.4326	1.38E-22	4.87E-21	40.3973
IGLV6-57	2.7987	4.94356	10.428	1.43E-22	5.05E-21	40.36
IGHV3-2:	2.77106	5.34561	10.4256	1.46E-22	5.14E-21	40.3405
SCN7A	2.19866	#NAME?	10.428	1.57E-22	5.51E-21	40.2757
IGHM	2.62396	6.4306	10.4152	1.59E-22	5.58E-21	40.2566
IGKV3OR	2.28742	#NAME?	10.4439	1.61E-22	5.64E-21	40.2582
MS4A2	1.81599	#NAME?	10.4151	1.62E-22	5.66E-21	40.2416
IGLL1	2.24842	#NAME?	10.4738	1.77E-22	6.17E-21	40.1817
CHI3L1	1.72132	4.20704	10.4011	1.79E-22	6.23E-21	40.1422
SIGLEC11	1.58019	#NAME?	10.406	1.88E-22	6.53E-21	40.0987
RP1-309I	1.79352	-4.4674	10.3934	1.90E-22	6.63E-21	40.0798
FCRL2	2.20962	#NAME?	10.4016	1.92E-22	6.67E-21	40.0768
AC009093	1.52163	#NAME?	10.3934	1.93E-22	6.71E-21	40.0664
LAIR2	1.66229	#NAME?	10.4145	1.95E-22	6.77E-21	40.0679
TRDC	1.75454	#NAME?	10.3934	1.96E-22	6.80E-21	40.053
BFSP2	1.88499	#NAME?	10.4392	1.97E-22	6.83E-21	40.0678
CCL7	1.90938	#NAME?	10.406	2.03E-22	7.02E-21	40.0273
MS4A1	2.49392	#NAME?	10.3921	2.04E-22	7.07E-21	40.0142
FAM198B	1.54989	#NAME?	10.3826	2.11E-22	7.30E-21	39.9789
IGHV1-69	2.82624	#NAME?	10.3816	2.16E-22	7.47E-21	39.9573
IGKV3D-:	2.51753	#NAME?	10.3821	2.35E-22	8.10E-21	39.8776
IL22RA2	1.98129	#NAME?	10.3669	2.51E-22	8.63E-21	39.8111
IGHG1	2.47208	9.03108	10.3581	2.54E-22	8.73E-21	39.7947
AF127936	1.81146	#NAME?	10.4076	2.76E-22	9.45E-21	39.7379
IGHV1-67	2.40215	#NAME?	10.3602	3.32E-22	1.14E-20	39.5436
RGS13	1.61105	#NAME?	10.3334	3.44E-22	1.17E-20	39.5002
IGKV1D-:	2.89283	#NAME?	10.4012	3.99E-22	1.36E-20	39.392
MCHR1	1.55423	#NAME?	10.2987	4.18E-22	1.42E-20	39.3031
IGKV1-8	2.56186	#NAME?	10.2988	4.49E-22	1.52E-20	39.2369
IGKV1OR	2.318	#NAME?	10.418	4.49E-22	1.52E-20	39.294
LINC0257	1.66503	#NAME?	10.2907	4.72E-22	1.60E-20	39.1852
AL133467	1.74437	#NAME?	10.331	4.94E-22	1.67E-20	39.1616
ESR1	1.79499	-2.7383	10.2619	5.55E-22	1.87E-20	39.021
IGLV7-43	2.80825	#NAME?	10.2456	6.71E-22	2.25E-20	38.8377

IGKV3D-	2.71771	#NAME?	10.2427	6.77E-22	2.27E-20	38.8273
IGHA2	2.59262	6.42476	10.2367	6.82E-22	2.29E-20	38.8183
LRRC55	2.15735	#NAME?	10.2376	6.95E-22	2.33E-20	38.8001
ATP6V0D	1.67722	-0.993	10.226	7.43E-22	2.49E-20	38.7328
LINC0178	1.86006	#NAME?	10.2974	7.75E-22	2.59E-20	38.7266
KCND2	1.62108	-1.4599	10.2202	7.79E-22	2.60E-20	38.6865
ZNF683	1.72903	-1.027	10.2112	8.38E-22	2.80E-20	38.6141
IGKV1-16	2.78185	#NAME?	10.2123	8.42E-22	2.81E-20	38.6101
IGHV3-1	2.80764	4.93602	10.1981	9.31E-22	3.10E-20	38.5098
HS3ST2	2.03785	#NAME?	10.197	9.53E-22	3.17E-20	38.4875
GPR88	1.63885	#NAME?	10.2068	1.07E-21	3.57E-20	38.3802
IGLV2-18	2.72677	#NAME?	10.1967	1.08E-21	3.60E-20	38.3674
CHRNA6	2.18789	#NAME?	10.2123	1.11E-21	3.67E-20	38.354
SIGLEC12	2.11404	-1.3337	10.1733	1.14E-21	3.77E-20	38.311
PTGFR	1.76659	#NAME?	10.1765	1.17E-21	3.87E-20	38.2858
PLPP4	1.69314	0.17272	10.1624	1.24E-21	4.10E-20	38.2239
FHL5	1.58883	#NAME?	10.1637	1.25E-21	4.11E-20	38.2223
AC13431	1.57032	#NAME?	10.1577	1.35E-21	4.43E-20	38.1484
IGHV1-58	2.79869	#NAME?	10.166	1.43E-21	4.69E-20	38.0974
DDO	1.59393	-1.2192	10.1392	1.50E-21	4.92E-20	38.0394
CXorf65	1.50895	#NAME?	10.1304	1.82E-21	5.97E-20	37.8543
PDZRN4	2.09411	#NAME?	10.1521	1.90E-21	6.23E-20	37.8228
IGKV3D-	2.49833	#NAME?	10.1186	2.03E-21	6.63E-20	37.7482
IGHV3-60	2.08755	#NAME?	10.1712	2.14E-21	6.98E-20	37.7247
AC135068	2.18173	#NAME?	10.1579	2.19E-21	7.14E-20	37.698
AC110995	1.57724	#NAME?	10.1595	2.20E-21	7.16E-20	37.6952
HAS2	1.6277	0.7423	10.0916	2.20E-21	7.17E-20	37.6601
CXCR2P1	2.1922	#NAME?	10.0992	2.21E-21	7.20E-20	37.6583
TNFRSF1	2.04009	#NAME?	10.1078	2.37E-21	7.71E-20	37.5968
CCL3L3	1.53671	#NAME?	10.0881	2.39E-21	7.74E-20	37.5878
CD19	1.97224	-0.6485	10.0731	2.55E-21	8.27E-20	37.5134
SLC1A7	1.70941	#NAME?	10.0744	2.63E-21	8.51E-20	37.4863
AC136428	2.25565	#NAME?	10.1336	2.83E-21	9.12E-20	37.449
NEGR1	1.66751	-3.1758	10.0599	2.84E-21	9.16E-20	37.408
MCEMP1	1.88316	-0.3892	10.0508	3.05E-21	9.82E-20	37.3362
IGHV1-2	2.90921	4.93676	10.048	3.12E-21	1.00E-19	37.3136
LAMP5	1.73473	0.70444	10.027	3.69E-21	1.18E-19	37.1475
PNOC	2.09391	#NAME?	10.0271	3.74E-21	1.20E-19	37.1358
TMEM130	1.7119	-1.1379	10.0232	3.81E-21	1.22E-19	37.1171
IGHV4-39	2.78177	5.9787	10.0224	3.83E-21	1.22E-19	37.1113
IGKV1-17	2.70347	#NAME?	10.0251	3.85E-21	1.23E-19	37.1081
AC064805	1.50088	#NAME?	10.0865	3.90E-21	1.24E-19	37.1257
ART4	2.00524	#NAME?	10.0383	4.02E-21	1.28E-19	37.0769
CD1E	1.78045	#NAME?	10.0219	4.05E-21	1.29E-19	37.0589
PAK3	1.84524	#NAME?	10.0156	4.16E-21	1.32E-19	37.0329
IGHV2-26	2.78086	#NAME?	10.0212	4.24E-21	1.35E-19	37.0158
AC007569	1.50191	#NAME?	10.0288	4.39E-21	1.39E-19	36.988
ADAM12	1.53774	1.726	10.0053	4.40E-21	1.39E-19	36.9757
IGHV1OR	2.4865	#NAME?	10.0343	4.44E-21	1.41E-19	36.9794
AC011511	1.59894	#NAME?	9.98076	5.42E-21	1.71E-19	36.7697
IGHV3-20	2.66955	#NAME?	10.0025	5.48E-21	1.73E-19	36.7703
CCL22	1.54737	0.71827	9.96973	5.84E-21	1.84E-19	36.6945
ANK2	1.52073	-2.047	9.96547	6.04E-21	1.90E-19	36.6609
SIGLEC6	1.72272	#NAME?	9.97589	6.33E-21	1.99E-19	36.624
IGKV3-7	2.37381	#NAME?	9.97643	6.57E-21	2.06E-19	36.5893
LINC0092	1.52375	#NAME?	9.96773	6.76E-21	2.12E-19	36.5583
GALNT17	1.87632	#NAME?	9.95272	6.78E-21	2.12E-19	36.5485
IGHV3-48	2.59646	#NAME?	9.95332	6.83E-21	2.14E-19	36.5415
G0S2	1.51059	3.28686	9.94847	6.92E-21	2.17E-19	36.5268
AC00913	1.56097	#NAME?	9.98433	7.50E-21	2.34E-19	36.4696
CCL14	2.02394	#NAME?	9.96436	7.63E-21	2.38E-19	36.4443
IGLV2-28	2.11403	#NAME?	9.99761	8.60E-21	2.68E-19	36.3502
XCR1	1.72534	#NAME?	9.91791	8.94E-21	2.78E-19	36.2744
TMEM156	1.78409	-0.3616	9.91072	9.35E-21	2.90E-19	36.2294
IGKV2-30	2.42686	#NAME?	9.92903	9.43E-21	2.93E-19	36.2304
KCNT2	1.5868	-3.2474	9.90965	9.43E-21	2.93E-19	36.221
AC034105	2.25203	#NAME?	10.0182	9.70E-21	3.00E-19	36.2537
IGHJ3	2.30848	#NAME?	9.92681	9.99E-21	3.09E-19	36.1761
IGHV3-7	2.34344	#NAME?	9.91752	1.01E-20	3.11E-19	36.1647
IGHV3OR	2.1607	#NAME?	9.94126	1.18E-20	3.64E-19	36.0287
MATN3	1.50495	0.241	9.87296	1.26E-20	3.89E-19	35.9326
IGLC3	2.36305	7.71748	9.86919	1.30E-20	4.00E-19	35.9031
IGLV3-21	2.69791	6.72816	9.86867	1.31E-20	4.02E-19	35.899
ITIH5	1.76384	-1.5384	9.85719	1.43E-20	4.39E-19	35.8089
IGHV3OR	2.21237	#NAME?	9.91679	1.45E-20	4.45E-19	35.8249
IGHV3OR	1.89731	#NAME?	10	1.46E-20	4.46E-19	35.867
IGKV1D-	2.59934	#NAME?	9.87382	1.52E-20	4.64E-19	35.7628
GPR55	1.55144	#NAME?	9.85055	1.53E-20	4.67E-19	35.7455
IGLJ2	1.87726	#NAME?	9.90695	1.57E-20	4.79E-19	35.749
FCRL1	2.13062	#NAME?	9.85698	1.62E-20	4.95E-19	35.6917
LINC0219	1.58146	#NAME?	9.85561	1.68E-20	5.13E-19	35.6588
CD1C	1.64594	-0.3995	9.81719	1.97E-20	5.97E-19	35.4956
AL606807	1.73747	#NAME?	9.84216	2.08E-20	6.30E-19	35.4557
LINC0254	1.60274	#NAME?	9.8249	2.15E-20	6.50E-19	35.4182
LINC0252	1.60575	#NAME?	9.98306	2.17E-20	6.57E-19	35.4903
IGHV3-7	2.1485	#NAME?	9.84385	2.23E-20	6.73E-19	35.3938
AC008760	1.6941	#NAME?	9.80662	2.24E-20	6.78E-19	35.3681
IGHV2-5	2.59284	#NAME?	9.81106	2.36E-20	7.12E-19	35.3223
IGLV5-45	2.92205	#NAME?	9.79833	2.48E-20	7.48E-19	35.2695
ARTN	-1.8094	2.27842	-9.7865	2.50E-20	7.54E-19	35.2559
IGF1	1.83467	-2.6304	9.78136	2.61E-20	7.84E-19	35.2156
IGLV8-61	2.87631	#NAME?	9.79336	2.62E-20	7.85E-19	35.2209
BLK	2.07081	#NAME?	9.75713	3.23E-20	9.68E-19	35.0047
TMEM52B	1.97956	-2.1096	9.75247	3.28E-20	9.80E-19	34.9903
KERA	1.75222	#NAME?	9.82567	3.36E-20	1.01E-18	35.0056
IGLV1-36	2.77669	#NAME?	9.74448	3.66E-20	1.09E-18	34.8842
IGKV5-2	2.70151	#NAME?	9.75675	4.06E-20	1.21E-18	34.7956
DKK2	1.80381	#NAME?	9.72616	4.08E-20	1.21E-18	34.7746
IGKV1-12	2.25005	#NAME?	9.7421	4.11E-20	1.22E-18	34.7759
IGHV3-7	2.61131	#NAME?	9.72652	4.12E-20	1.22E-18	34.7665
IGLV3-27	2.8723	#NAME?	9.72599	4.18E-20	1.24E-18	34.7515
IQSEC3	1.54055	-3.0034	9.69778	5.04E-20	1.49E-18	34.5648
SERPINA1	1.87217	4.60481	9.68592	5.53E-20	1.63E-18	34.4727
IGHV4-34	2.45391	5.40588	9.67618	5.97E-20	1.76E-18	34.3971
IGLC6	2.03253	#NAME?	9.69744	6.05E-20	1.78E-18	34.3968
CPA3	1.8565	2.43148	9.67186	6.18E-20	1.82E-18	34.3636
BIRC7	1.64041	#NAME?	9.65425	7.34E-20	2.15E-18	34.1955
CCDC141	1.71782	#NAME?	9.65329	7.49E-20	2.19E-18	34.1774
SLC12A3	1.66419	#NAME?	9.66045	8.07E-20	2.35E-18	34.1122
SELE	2.23103	-1.2234	9.63343	8.35E-20	2.43E-18	34.066
IGSF21	1.66861	#NAME?	9.63453	8.67E-20	2.52E-18	34.0326
AC134879	2.2734	#NAME?	9.66577	1.01E-19	2.94E-18	33.9043
AC106883	1.54034	#NAME?	9.62495	1.08E-19	3.11E-18	33.8301
CD1B	1.75873	#NAME?	9.63296	1.10E-19	3.18E-18	33.8103
IGLV10-5	2.64967	#NAME?	9.61804	1.16E-19	3.35E-18	33.7548
SLCO5A1	1.67934	-3.3959	9.58767	1.19E-19	3.44E-18	33.7126
MAGEL2	1.69697	#NAME?	9.58955	1.23E-19	3.54E-18	33.6858
IGHV3OR	1.96369	#NAME?	9.69741	1.24E-19	3.56E-18	33.7431
IGHV3OR	2.02048	#NAME?	9.6676	1.27E-19	3.65E-18	33.6998
IGLV3-9	2.80515	#NAME?	9.57396	1.34E-19	3.86E-18	33.5966
UNC5A	1.61718	#NAME?	9.57151	1.39E-19	3.97E-18	33.5675
IL5RA	1.90668	#NAME?	9.54973	1.66E-19	4.74E-18	33.3899
IGHV3-49	2.62522	#NAME?	9.54543	1.68E-19	4.80E-18	33.3771
CCL11	1.6939	#NAME?	9.52923	1.95E-19	5.53E-18	33.2325
ADGRE3	1.71452	#NAME?	9.52833	2.00E-19	5.69E-18	33.2069
ANKRD36	1.98476	-1.7479	9.49429	2.47E-19	6.99E-18	32.9945
HLA-DOA	2.25399	2.9882	9.4929	2.50E-19	7.05E-18	32.9838
TRAV1-2	1.5103	#NAME?	9.58868	2.68E-19	7.56E-18	32.9703
IGHGP	2.28442	5.15449	9.4819	2.72E-19	7.67E-18	32.8995
IGHJ2	2.30457	#NAME?	9.51884	2.76E-19	7.76E-18	32.9097
AL162414	1.55354	#NAME?	9.77511	2.82E-19	7.94E-18	33.0248
IGLV3-10	2.806	#NAME?	9.46053	3.25E-19	9.09E-18	32.7261
INHBA-A	1.58186	#NAME?	9.47367	3.38E-19	9.46E-18	32.6968
IGHG2	2.39019	8.33527	9.45008	3.48E-19	9.72E-18	32.656
IGHV1-69	2.74856	#NAME?	9.42985	4.25E-19	1.18E-17	32.4627
CD8B	1.64123	0.21806	9.42326	4.29E-19	1.19E-17	32.4512
VNN1	1.8126	-0.8025	9.4216	4.34E-19	1.21E-17	32.4385
CCL4L2	1.55659	#NAME?	9.3989	5.23E-19	1.44E-17	32.2576
IGKV1D-	2.48173	#NAME?	9.52797	5.60E-19	1.54E-17	32.2711
IGHV3-4	2.46161	#NAME?	9.39356	5.74E-19	1.58E-17	32.1671
AC13364	1.59117	#NAME?	9.41017	6.47E-19	1.78E-17	32.068
AC246787	1.6976	#NAME?	9.51386	7.22E-19	1.97E-17	32.0293
IGKV2D-	2.65208	#NAME?	9.36396	8.20E-19	2.23E-17	31.8239
IGHG3	2.25964	7.48574	9.33787	8.28E-19	2.25E-17	31.8013
PKD2L1	1.57631	#NAME?	9.33782	8.62E-19	2.34E-17	31.7636
COPDA1	1.84928	#NAME?	9.38137	9.80E-19	2.65E-17	31.676
NTRK3	1.99083	#NAME?	9.30156	1.12E-18	3.01E-17	31.5078
CLIC5	1.72527	-1.0238	9.29038	1.19E-18	3.21E-17	31.4416
FFAR4	1.68797	#NAME?	9.29638	1.20E-18	3.22E-17	31.4409
IGLV3-16	2.21765	#NAME?	9.30464	1.39E-18	3.72E-17	31.3073
IGKV2D-	2.67092	#NAME?	9.27806	1.41E-18	3.76E-17	31.2841
LY6H	1.84904	#NAME?	9.27862	1.43E-18	3.81E-17	31.2696
AC00803	1.68671	#NAME?	9.38416	1.45E-18	3.87E-17	31.3186
IGHV3-64	2.45232	#NAME?	9.27987	1.46E-18	3.89E-17	31.2505
IFI44L	1.62504	0.3937	9.25861	1.52E-18	4.05E-17	31.2016
TCL1A	2.11706	#NAME?	9.25524	1.78E-18	4.72E-17	31.0561
AC022509	1.56501	#NAME?	9.2437	1.83E-18	4.84E-17	31.0256
COL4A4	1.561	-1.1184	9.23337	1.84E-18	4.88E-17	31.0112
GCSAML	1.51095	#NAME?	9.22658	2.10E-18	5.54E-17	30.8879
SUSD5	1.52722	-1.8869	9.20504	2.29E-18	6.03E-17	30.7979
IGHV3-6	1.89584	#NAME?	9.21578	2.82E-18	7.38E-17	30.6151
GPA33	1.58217	#NAME?	9.18095	3.12E-18	8.16E-17	30.5005
NETO1	1.9108	#NAME?	9.16972	3.20E-18	8.35E-17	30.4711
INMT	1.55007	0.48518	9.16076	3.20E-18	8.35E-17	30.4655
C7	2.47905	1.42132	9.12884	4.08E-18	1.06E-16	30.2265
LRRC2	1.57342	#NAME?	9.11819	4.51E-18	1.17E-16	30.1298
IGLV3-12	2.21073	#NAME?	9.14745	4.86E-18	1.25E-16	30.0799
IGHG4	2.49468	7.23472	9.1021	5.00E-18	1.29E-16	30.0266
TNN	1.60417	#NAME?	9.101	5.23E-18	1.35E-16	29.9845
CHRNA1	1.70003	#NAME?	9.08022	6.18E-18	1.58E-16	29.8212
GFRA1	1.85034	#NAME?	9.04137	8.05E-18	2.05E-16	29.5595
HLA-DPB	1.80802	#NAME?	9.03499	8.45E-18	2.14E-16	29.5103
IGLV7-46	2.41189	3.52449	9.02772	8.76E-18	2.22E-16	29.4728
MAMDC2	1.74557	#NAME?	9.01327	9.86E-18	2.49E-16	29.3574
IGHV3-35	2.01518	#NAME?	9.04223	1.04E-17	2.62E-16	29.3274
IGHV3-4	1.9101	#NAME?	9.04858	1.06E-17	2.66E-16	29.318
IGHV3-19	2.02722	#NAME?	9.05128	1.06E-17	2.67E-16	29.3151
C4B	1.57036	-0.7481	8.99202	1.15E-17	2.88E-16	29.208
PEG3	1.66964	-3.1816	8.99098	1.15E-17	2.90E-16	29.2003
ELN	1.76972	2.18395	8.99066	1.16E-17	2.91E-16	29.1979
FCER2	1.87603	#NAME?	8.99107	1.34E-17	3.35E-16	29.0676
IGLV9-49	2.60069	#NAME?	8.98218	1.34E-17	3.35E-16	29.0616
INSYN2B	1.50715	#NAME?	9.00362	1.39E-17	3.47E-16	29.0432
IGHV3-6	1.81795	#NAME?	9.02044	1.54E-17	3.84E-16	28.9613
IGKV1D-	2.26159	#NAME?	8.99965	1.59E-17	3.96E-16	28.9195
CKMT1B	-1.7229	0.74932	-8.9481	1.59E-17	3.97E-16	28.8834
CLEC4G	1.59143	#NAME?	8.96147	1.64E-17	4.08E-16	28.8724
IGHV1OR	2.08573	#NAME?	8.97204	1.92E-17	4.76E-16	28.7286
AL033397	-1.6775	#NAME?	-8.8722	2.91E-17	7.11E-16	28.2988
AC10356	1.99599	#NAME?	8.96714	2.92E-17	7.14E-16	28.3571
IGLC7	2.59588	#NAME?	8.86599	3.23E-17	7.89E-16	28.1927
ABCA8	1.70594	#NAME?	8.8465	3.46E-17	8.41E-16	28.1206
MT1M	1.53526	#NAME?	8.83907	3.62E-17	8.80E-16	28.0738
CLEC4C	1.51492	#NAME?	8.91793	4.60E-17	1.11E-15	27.9195
AL928768	1.70917	#NAME?	8.82969	4.85E-17	1.17E-15	27.8054
FCRL4	1.78719	#NAME?	8.83575	5.06E-17	1.22E-15	27.7727
DTHD1	1.77998	#NAME?	8.79391	5.15E-17	1.24E-15	27.7288
MMP12	1.65494	4.64138	8.7702	5.98E-17	1.43E-15	27.5781
MMP8	1.83263	#NAME?	8.77444	6.15E-17	1.47E-15	27.557
LRRC17	1.50574	0.31867	8.76548	6.20E-17	1.48E-15	27.5438
FAM107A	1.64219	-0.7731	8.74033	7.46E-17	1.77E-15	27.3607
CNNM1	-1.9631	-0.8292	-8.7234	8.45E-17	1.99E-15	27.2377
PTX3	1.85987	#NAME?	8.71699	8.93E-17	2.10E-15	27.184
MMP7	1.7875	3.67484	8.71548	8.96E-17	2.11E-15	27.1802
IGHV3-38	1.83579	#NAME?	8.7096	1.24E-16	2.90E-15	26.8837
FCGR3B	2.08344	#NAME?	8.67437	1.25E-16	2.92E-15	26.854
LINC0192	1.81684	#NAME?	8.66986	1.34E-16	3.11E-15	26.7927
IGKV2OR	2.13844	#NAME?	8.70681	1.37E-16	3.19E-15	26.7959
IGKV1OR	2.10618	#NAME?	8.68536	1.54E-16	3.56E-15	26.6807
IGHV4-4	2.32895	#NAME?	8.65699	1.58E-16	3.66E-15	26.6338
CADM3	1.82732	-1.8348	8.63002	1.68E-16	3.87E-15	26.562
IGKV3D-	2.08142	#NAME?	8.76354	1.94E-16	4.47E-15	26.5356
JSRP1	1.52266	#NAME?	8.60846	1.98E-16	4.54E-15	26.3999
CNTN3	1.55008	#NAME?	8.60608	2.01E-16	4.62E-15	26.3828
VSTM1	1.63507	#NAME?	8.71694	2.04E-16	4.68E-15	26.4579
CHIT1	2.0993	0.63062	8.6005	2.08E-16	4.77E-15	26.3494
LINC0052	1.59902	#NAME?	8.62368	2.19E-16	5.02E-15	26.3262
IGKV1OR	2.0769	#NAME?	8.6445	2.29E-16	5.24E-15	26.2955
IGKV2-28	2.26013	#NAME?	8.62521	2.46E-16	5.61E-15	26.219
CCBE1	1.64073	-2.0667	8.57608	2.49E-16	5.66E-15	26.174
FGF10	1.90296	#NAME?	8.62954	2.58E-16	5.86E-15	26.1828
GPX2	-2.6871	6.08243	-8.5691	2.62E-16	5.95E-15	26.124
IGHV3-4	1.71513	#NAME?	8.5956	2.78E-16	6.31E-15	26.0903
SYNDIG1	1.67304	#NAME?	8.55938	2.83E-16	6.42E-15	26.0475
COL6A5	1.7889	#NAME?	8.5489	3.10E-16	7.02E-15	25.9591
GPR15	1.80318	#NAME?	8.55964	3.15E-16	7.12E-15	25.9527
IGHV1-3	2.39628	#NAME?	8.5547	3.46E-16	7.80E-15	25.8669
IGHV5-78	1.90135	#NAME?	8.5529	3.50E-16	7.89E-15	25.8559
PADI2	1.54529	0.14675	8.52217	3.68E-16	8.28E-15	25.7878
NDNF	1.98654	0.54139	8.51656	3.83E-16	8.61E-15	25.7477
IGKV6-21	2.54016	#NAME?	8.52764	4.00E-16	8.97E-15	25.7181
IGLV2-34	1.95007	#NAME?	8.54153	4.73E-16	1.06E-14	25.5804
FAM155A	1.59612	-3.5038	8.48398	4.85E-16	1.08E-14	25.5153
IGHJ1	1.90017	#NAME?	8.53157	5.18E-16	1.15E-14	25.4957
IL1RL1	1.59442	#NAME?	8.46924	5.44E-16	1.21E-14	25.4038
GPIHBP1	1.66438	#NAME?	8.46227	5.72E-16	1.27E-14	25.3543
AC244205	1.66041	#NAME?	8.47786	5.95E-16	1.32E-14	25.332
LINC0123	1.63106	#NAME?	8.45612	6.29E-16	1.39E-14	25.2653
IGKV2D-	2.20768	#NAME?	8.48628	6.58E-16	1.45E-14	25.2517
IGHV1-45	2.28516	#NAME?	8.44375	7.09E-16	1.56E-14	25.1512
IGKV1D-	1.98917	#NAME?	8.46291	7.37E-16	1.62E-14	25.1323
IGHV3-5	1.82781	#NAME?	8.46149	7.77E-16	1.71E-14	25.0828
SHISAL1	1.65931	-2.5187	8.40364	8.66E-16	1.89E-14	24.9445
FCER1A	1.76287	#NAME?	8.4061	8.76E-16	1.91E-14	24.937
AC10356	1.98044	#NAME?	8.44647	8.80E-16	1.92E-14	24.9626
CXCL5	2.01325	-0.13	8.4014	8.80E-16	1.92E-14	24.9287
IGKV1OR	1.90595	#NAME?	8.51617	9.32E-16	2.03E-14	24.9722
IDO1	1.66564	2.41198	8.38319	1.00E-15	2.18E-14	24.7999
CKMT1A	-1.5804	#NAME?	-8.3747	1.07E-15	2.33E-14	24.7359
SGCA	1.62231	-1.0665	8.35134	1.26E-15	2.71E-14	24.575
CYP4F26F	-1.7931	#NAME?	-8.3672	1.30E-15	2.79E-14	24.5918
SORCS2	1.56351	-0.3473	8.34096	1.36E-15	2.91E-14	24.5019
PDK4	1.63501	0.55729	8.33268	1.44E-15	3.08E-14	24.4436
PLA2G2A	2.23362	#NAME?	8.33903	1.47E-15	3.13E-14	24.4332
RETN	1.66493	#NAME?	8.34078	1.52E-15	3.25E-14	24.4026
IGHV3-76	1.72356	#NAME?	8.38812	1.56E-15	3.32E-14	24.4199
PKHD1L1	1.63952	#NAME?	8.32509	1.60E-15	3.39E-14	24.3478
FOXH1	-1.5193	#NAME?	-8.3213	1.63E-15	3.46E-14	24.3369
AC246787	1.56453	#NAME?	8.35939	1.92E-15	4.06E-14	24.2208
IGHV3OR	1.87739	#NAME?	8.31804	2.19E-15	4.61E-14	24.0659
LINC0094	1.66325	#NAME?	8.26466	2.57E-15	5.38E-14	23.8823
IGHV3-2	1.67785	#NAME?	8.31462	2.65E-15	5.53E-14	23.9015
IGHV2-70	2.58616	#NAME?	8.26758	2.71E-15	5.65E-14	23.8392
IGHJ3P	1.69867	#NAME?	8.25584	3.18E-15	6.57E-14	23.6916
IGKV1OR	1.8099	#NAME?	8.30346	3.34E-15	6.90E-14	23.6899
IGHV7-56	1.8393	#NAME?	8.25093	3.81E-15	7.82E-14	23.5317
ANKRD18	-1.7379	#NAME?	-8.1966	3.94E-15	8.08E-14	23.4671
IFNG	1.61216	#NAME?	8.20155	4.07E-15	8.35E-14	23.4323
IGHV7-8	1.91978	#NAME?	8.21547	4.42E-15	9.02E-14	23.375
FAM92B	1.91527	#NAME?	8.17796	4.53E-15	9.24E-14	23.322
IGLV2-33	1.85427	#NAME?	8.24182	4.68E-15	9.53E-14	23.3488
IGLL3P	1.67764	#NAME?	8.22229	4.85E-15	9.86E-14	23.3039
ADRA1B	1.57325	#NAME?	8.16883	5.20E-15	1.05E-13	23.1943
IGHV1-1	1.79321	#NAME?	8.20317	6.33E-15	1.27E-13	23.0529
CD70	1.6765	#NAME?	8.09407	7.85E-15	1.56E-13	22.7755
PRG4	1.57449	-2.5265	8.09004	8.03E-15	1.60E-13	22.753
DNASE2B	1.55991	#NAME?	8.10585	8.77E-15	1.74E-13	22.6927

CASKIN1	-1.5297	#NAME?	-8.0665	9.52E-15	1.88E-13	22.5874
CSF2	1.51516	#NAME?	8.07191	9.58E-15	1.89E-13	22.5852
IGKV2D-	2.11714	#NAME?	8.15648	1.09E-14	2.14E-13	22.5581
CDO1	1.66487	#NAME?	8.04471	1.11E-14	2.18E-13	22.4359
LRRC4C	1.5162	#NAME?	8.04498	1.13E-14	2.22E-13	22.4166
IGLV4-3	1.98812	#NAME?	8.08916	1.37E-14	2.68E-13	22.295
AC13506	1.95307	#NAME?	8.17606	1.41E-14	2.74E-13	22.3538
SCN2B	1.52258	#NAME?	7.99249	1.65E-14	3.20E-13	22.0472
TNIP3	1.57552	-0.8365	7.9822	1.70E-14	3.29E-13	22.013
IGKV7-3	1.80391	#NAME?	8.05565	1.77E-14	3.42E-13	22.049
IGKV2D-	2.25838	#NAME?	8.04496	2.11E-14	4.04E-13	21.8941
IGLV1-41	2.29725	#NAME?	7.98384	2.13E-14	4.09E-13	21.8218
L1TD1	1.58156	#NAME?	7.96511	2.15E-14	4.13E-13	21.8007
MFAP5	1.80038	#NAME?	7.94052	2.29E-14	4.37E-13	21.7239
PRND	1.57535	#NAME?	7.93707	2.73E-14	5.19E-13	21.5727
IGHV1-1	1.98845	#NAME?	7.94067	2.73E-14	5.19E-13	21.5725
DIO3	1.57711	#NAME?	7.89012	3.64E-14	6.85E-13	21.2861
IGHV1-1	1.83302	#NAME?	7.91984	4.13E-14	7.73E-13	21.2088
APCDD1L	1.79016	#NAME?	7.82819	4.95E-14	9.23E-13	20.9637
KCNA5	1.56933	#NAME?	7.83711	5.23E-14	9.72E-13	20.9271
APELA	1.68101	#NAME?	7.8167	5.80E-14	1.07E-12	20.8229
LINC0156	-1.7281	#NAME?	-7.8003	6.30E-14	1.16E-12	20.7396
CCDC162	-1.5352	#NAME?	-7.79	6.43E-14	1.18E-12	20.707
IGHD	2.56559	2.70518	7.78252	6.73E-14	1.24E-12	20.6614
MYOCD	1.60325	#NAME?	7.79341	6.87E-14	1.26E-12	20.6536
ACKR1	1.80024	#NAME?	7.77878	6.94E-14	1.27E-12	20.6318
USH1G	-1.819	#NAME?	-7.7509	8.53E-14	1.55E-12	20.4356
IGKV1D-	2.02728	#NAME?	7.7679	9.59E-14	1.74E-12	20.3514
IGLV2-5	1.64619	#NAME?	7.7684	1.04E-13	1.88E-12	20.2766
VSTM2L	1.75347	0.74766	7.71259	1.08E-13	1.95E-12	20.194
GDF10	1.74799	#NAME?	7.71465	1.16E-13	2.08E-12	20.1414
AIM2	1.52111	1.15148	7.67373	1.41E-13	2.51E-12	19.9355
IGHV3OR	1.59915	#NAME?	7.74458	1.42E-13	2.53E-12	20.0039
IGKV1-1	1.85588	#NAME?	7.73127	1.49E-13	2.64E-12	19.9472
IGKV1D-	2.15888	#NAME?	7.67042	1.66E-13	2.92E-12	19.7988
IGKV1OR	1.5697	#NAME?	7.74621	1.77E-13	3.10E-12	19.8265
CR2	2.03345	#NAME?	7.57646	2.77E-13	4.76E-12	19.2765
SAA2	1.88146	#NAME?	7.56857	2.87E-13	4.94E-12	19.2369
AC11448	-1.5045	#NAME?	-7.5986	2.94E-13	5.04E-12	19.2908
KCNA2	1.56415	#NAME?	7.58653	3.28E-13	5.60E-12	19.1451
IGKV6D-	2.32949	#NAME?	7.56235	3.69E-13	6.26E-12	19.0222
TBC1D27	1.58272	#NAME?	7.52537	4.11E-13	6.94E-12	18.8982
IGHV1OR	1.59116	#NAME?	7.57422	4.41E-13	7.43E-12	18.8942
ZBTB16	1.82781	#NAME?	7.49333	4.83E-13	8.09E-12	18.7287
AC24425	2.22619	#NAME?	7.49229	5.56E-13	9.24E-12	18.6175
CBLN4	1.70043	#NAME?	7.46695	6.53E-13	1.07E-11	18.4569
ENPP6	1.65841	#NAME?	7.44394	6.70E-13	1.10E-11	18.4079
ZPLD1	1.58798	#NAME?	7.4591	6.73E-13	1.10E-11	18.4277
SAA1	1.80501	3.48232	7.43623	6.89E-13	1.13E-11	18.377
CHRDL1	1.8953	#NAME?	7.43823	6.90E-13	1.13E-11	18.3786
ASXL3	1.60018	#NAME?	7.432	7.53E-13	1.23E-11	18.2993
NMRAL2	-2.6455	2.48028	-7.4176	7.79E-13	1.27E-11	18.2563
DES	1.67002	#NAME?	7.3937	9.16E-13	1.49E-11	18.0981
ACTG1P2	1.59477	#NAME?	7.42442	9.36E-13	1.52E-11	18.1129
IGHV3-7	1.52732	#NAME?	7.4472	1.07E-12	1.73E-11	18.0296
AC09181	-2.095	#NAME?	-7.3903	1.24E-12	1.98E-11	17.9149
SOX2	-1.924	5.3687	-7.3442	1.26E-12	2.01E-11	17.7831
MMP28	1.51934	0.4732	7.32821	1.40E-12	2.22E-11	17.6802
IGHV4OR	1.53508	#NAME?	7.41258	1.45E-12	2.30E-11	17.7529
CD300LG	1.64732	#NAME?	7.36252	1.53E-12	2.42E-11	17.653
HAS1	1.55288	#NAME?	7.31082	1.63E-12	2.56E-11	17.5403
EXOC3L4	1.50064	-2.1197	7.30076	1.68E-12	2.64E-11	17.5044
AL391427	-1.7461	#NAME?	-7.3152	1.69E-12	2.65E-11	17.5427
IGFL2	1.58263	#NAME?	7.26696	2.11E-12	3.28E-11	17.2817
TRIM55	1.50976	#NAME?	7.28428	2.11E-12	3.28E-11	17.2999
TENM3-A	1.52626	#NAME?	7.25006	2.44E-12	3.77E-11	17.1428
PAX5	1.56821	#NAME?	7.23496	2.58E-12	3.98E-11	17.0815
FDCSP	2.67731	#NAME?	7.2361	2.71E-12	4.16E-11	17.0438
ADCYAP1	1.56963	#NAME?	7.20551	3.30E-12	5.02E-11	16.8506
BRINP3	1.604	#NAME?	7.2151	3.47E-12	5.25E-11	16.823
HAP1	-1.6454	0.6849	-7.0812	6.92E-12	1.02E-10	16.1157
PLCXD3	1.60674	#NAME?	7.03709	9.62E-12	1.39E-10	15.8012
IGKV1D-	1.90719	#NAME?	7.06395	1.03E-11	1.48E-10	15.7811
IGKV2OR	1.63628	#NAME?	7.05627	1.14E-11	1.64E-10	15.6979
RASGRF1	1.54886	#NAME?	6.99456	1.20E-11	1.72E-10	15.5741
IGLV4-60	2.30364	#NAME?	6.99788	1.24E-11	1.76E-10	15.5558
MYBPC2	1.5284	-2.2086	6.97216	1.38E-11	1.96E-10	15.4385
MMP1	1.66799	5.22488	6.96604	1.43E-11	2.03E-10	15.4007
IGLV5-48	1.54279	#NAME?	7.00881	1.46E-11	2.07E-10	15.4389
SLC22A3	1.53546	#NAME?	6.93611	1.74E-11	2.45E-10	15.2107
CYP4F3	-2.7038	1.06462	-6.913	2.00E-11	2.79E-10	15.0745
CXCR1	1.63536	#NAME?	6.87552	2.58E-11	3.55E-10	14.8306
IL13RA2	1.56345	#NAME?	6.87093	2.62E-11	3.60E-10	14.8114
KANK4	1.69002	#NAME?	6.85966	2.84E-11	3.88E-10	14.7347
IGHV1OR	1.72381	#NAME?	6.9256	3.20E-11	4.34E-10	14.7373
IGLV5-37	2.16395	#NAME?	6.83374	4.01E-11	5.39E-10	14.4458
DNAJB3	-1.513	#NAME?	-6.8295	4.30E-11	5.76E-10	14.4502
SCARA5	1.63838	#NAME?	6.78103	4.66E-11	6.21E-10	14.2529
AC11481	-1.5003	#NAME?	-6.7888	5.34E-11	7.07E-10	14.2283
AKR1C2	-2.224	4.47248	-6.7328	6.13E-11	8.05E-10	13.98
TLX2	-1.5547	#NAME?	-6.7367	6.14E-11	8.06E-10	13.992
OTOP2	-1.832	#NAME?	-6.7692	6.15E-11	8.07E-10	14.0912
ANKRD1	1.62184	#NAME?	6.67883	8.72E-11	1.13E-09	13.64
XKR4	1.70713	#NAME?	6.64806	1.17E-10	1.50E-09	13.3829
EREG	1.83418	#NAME?	6.62306	1.21E-10	1.55E-09	13.3157
IGLVI-70	1.59607	#NAME?	6.5996	1.56E-10	1.97E-09	13.0957
AC00533	-2.5797	#NAME?	-6.5799	1.68E-10	2.12E-09	13.0304
DLX6-AS	-1.5496	#NAME?	-6.5662	1.71E-10	2.15E-09	12.9862
PNCK	-1.5819	1.35889	-6.5563	1.79E-10	2.25E-09	12.9305
ADH1B	2.04883	#NAME?	6.54616	1.93E-10	2.42E-09	12.8599
OSGIN1	-1.5742	2.64615	-6.5293	2.11E-10	2.62E-09	12.7714
DMRT2	-1.9679	#NAME?	-6.5097	2.39E-10	2.95E-09	12.6549
ALDH3A1	-2.3552	4.06938	-6.4771	2.88E-10	3.53E-09	12.4666
SAA2-SA	1.66029	#NAME?	6.48628	2.94E-10	3.59E-09	12.4701
AC00801	-1.6079	#NAME?	-6.4606	3.30E-10	4.00E-09	12.3564
ISM2	-1.5259	#NAME?	-6.4292	3.86E-10	4.64E-09	12.1861
SLC47A2	-1.5804	-1.8808	-6.3971	4.64E-10	5.52E-09	12.0023
BMP5	1.56846	#NAME?	6.39267	4.80E-10	5.70E-09	11.9713
IGKV1D-	1.8632	#NAME?	6.38131	5.77E-10	6.79E-09	11.8238
IGHE	1.51979	#NAME?	6.36329	5.88E-10	6.91E-09	11.7811
PART1	-1.843	#NAME?	-6.2613	1.07E-09	1.22E-08	11.2197
GPM6A	1.51225	#NAME?	6.2496	1.15E-09	1.31E-08	11.1277
IL17REL	1.52348	#NAME?	6.23145	1.26E-09	1.42E-08	11.0404
SERTM2	1.55344	#NAME?	6.25233	1.26E-09	1.43E-08	11.0793
CYP4F11	-2.5578	#NAME?	-6.2108	1.38E-09	1.56E-08	10.9371
AKR1C1	-2.0407	4.02991	-6.2069	1.41E-09	1.58E-08	10.9181
CES1P2	-1.6141	#NAME?	-6.2555	1.82E-09	2.01E-08	10.9486
FADS6	-1.7814	#NAME?	-6.155	1.99E-09	2.20E-08	10.6139
FAM216B	1.87997	#NAME?	6.15298	2.05E-09	2.25E-08	10.5787
COL22A1	1.63427	-1.3151	6.13893	2.08E-09	2.29E-08	10.5373
MMP13	2.15925	1.32032	6.10929	2.47E-09	2.68E-08	10.3722
LINC0046	1.55728	#NAME?	6.11701	2.57E-09	2.79E-08	10.3615
NECAB2	-1.659	#NAME?	-6.0993	2.64E-09	2.85E-08	10.3128
AKR1B10	-2.4267	5.10845	-6.0788	2.94E-09	3.16E-08	10.2034
HHIP	1.59349	#NAME?	6.03633	3.75E-09	3.97E-08	9.96588
EMILIN3	-1.7462	#NAME?	-6.0264	3.96E-09	4.18E-08	9.91254
IGLICOR1	1.51108	#NAME?	6.10507	4.72E-09	4.93E-08	9.97942
NTNG1	1.51882	#NAME?	5.98274	5.29E-09	5.49E-08	9.64726
AC00572	-1.5226	#NAME?	-5.9627	5.81E-09	5.99E-08	9.5731
AL033397	-2.9784	#NAME?	-5.9459	6.57E-09	6.72E-08	9.45764
NLRP7	1.57075	#NAME?	5.92938	6.97E-09	7.10E-08	9.37001
AC24425	1.51613	#NAME?	5.97831	7.04E-09	7.17E-08	9.4711
MMP3	1.63595	#NAME?	5.91873	7.24E-09	7.37E-08	9.32553
LINC0113	-1.8661	#NAME?	-5.887	8.67E-09	8.73E-08	9.15409
ORM1	1.57948	#NAME?	5.88793	9.01E-09	9.06E-08	9.13321
CAPN6	1.61951	#NAME?	5.86	1.02E-08	1.01E-07	9.00032
CACNA1F	-1.8043	#NAME?	-5.8311	1.18E-08	1.16E-07	8.85522
AKR1B10	-2.1275	#NAME?	-5.8509	1.25E-08	1.23E-07	8.92915
SLC34A2	1.89954	#NAME?	5.81246	1.30E-08	1.28E-07	8.75459
ADH7	-2.5005	#NAME?	-5.7906	1.49E-08	1.46E-07	8.64154
FABP4	1.56645	#NAME?	5.77878	1.57E-08	1.52E-07	8.5743
IGKV3OR	1.58027	#NAME?	5.87407	1.80E-08	1.74E-07	8.70676
LINC0087	-1.6827	#NAME?	-5.7474	2.16E-08	2.06E-07	8.39139
PAEP	1.50659	#NAME?	5.71307	2.32E-08	2.21E-07	8.20889
CTSE	2.11513	#NAME?	5.67393	2.79E-08	2.63E-07	8.0183
MXRA5Y	1.60947	#NAME?	5.67862	2.82E-08	2.66E-07	8.02751
NTS	-2.7509	#NAME?	-5.6515	3.13E-08	2.93E-07	7.90647
SOX2-OT	-1.6005	-1.0948	-5.6352	3.40E-08	3.16E-07	7.82231
AKR1C3	-1.7725	4.43807	-5.6342	3.42E-08	3.18E-07	7.81731
LTF	1.86362	3.14823	5.62044	3.68E-08	3.41E-07	7.74594
CEL	-1.5422	1.12801	-5.6137	3.82E-08	3.53E-07	7.71099
AL02139	-2.2545	#NAME?	-5.6928	3.90E-08	3.60E-07	7.99393
C4BPA	2.03624	#NAME?	5.58446	4.51E-08	4.13E-07	7.55563
DMRT3	-1.6072	#NAME?	-5.5824	4.57E-08	4.18E-07	7.54651
AKR1C4	-1.5197	#NAME?	-5.585	4.84E-08	4.41E-07	7.56417
NKAIN2	-1.6321	#NAME?	-5.5045	6.82E-08	6.08E-07	7.15126
FEZF1	-1.6437	#NAME?	-5.4806	7.79E-08	6.88E-07	7.03049
GSTA1	-2.1171	#NAME?	-5.3947	1.21E-07	1.04E-06	6.59512
STXBP5L	-1.8022	#NAME?	-5.3614	1.47E-07	1.24E-06	6.43379
CLDN18	1.58019	#NAME?	5.34646	1.55E-07	1.31E-06	6.35437
CHIAP2	1.73254	#NAME?	5.44895	1.57E-07	1.32E-06	6.65466
FAR2P1	-1.9125	#NAME?	-5.3046	1.98E-07	1.65E-06	6.15939
ZFP57	1.50751	#NAME?	5.31559	1.98E-07	1.65E-06	6.17503
ODAM	1.71142	#NAME?	5.22527	3.12E-07	2.51E-06	5.73717
UGT1A6	-1.6193	#NAME?	-5.1938	3.38E-07	2.70E-06	5.61054
CHGA	-1.6549	#NAME?	-5.1408	4.44E-07	3.49E-06	5.35139
SFTPD	1.63031	3.36442	5.13546	4.50E-07	3.53E-06	5.32473
FOXL2	-1.6866	#NAME?	-5.1082	5.27E-07	4.09E-06	5.19836
NELL1	-2.0168	#NAME?	-5.0999	5.49E-07	4.24E-06	5.16129
CASC19	-1.5633	#NAME?	-5.0938	5.79E-07	4.46E-06	5.14211
CYP4B1	1.51696	0.632	5.03954	7.22E-07	5.45E-06	4.86824
SOX21	-1.5077	1.95265	-5.006	8.50E-07	6.36E-06	4.71065
IGKV2-2	1.59775	#NAME?	4.98164	1.01E-06	7.49E-06	4.5771
NAPSA	1.64058	3.13408	4.95172	1.11E-06	8.14E-06	4.45697
AC00506	-1.5463	#NAME?	-4.9482	1.26E-06	9.22E-06	4.49586
GOLGA6L	-1.7683	#NAME?	-4.9443	1.27E-06	9.31E-06	4.44608
GSTA9P	-2.0524	#NAME?	-4.9081	1.45E-06	1.05E-05	4.28913
DMRTA2	-1.6556	#NAME?	-4.8451	1.86E-06	1.32E-05	3.97478
NR0B1	-2.6129	#NAME?	-4.7764	2.87E-06	1.98E-05	3.71892
LINC016	-1.5814	#NAME?	-4.7065	3.67E-06	2.49E-05	3.38315
AL03542	-1.881	#NAME?	-4.6936	3.92E-06	2.64E-05	3.32738
UGT1A7	-2.0729	#NAME?	-4.6867	3.97E-06	2.68E-05	3.28575
AQP4	1.6485	#NAME?	4.66986	4.20E-06	2.82E-05	3.18149
IRS4	-1.8812	#NAME?	-4.6591	4.73E-06	3.15E-05	3.18959
AP00311	-1.5019	#NAME?	-4.549	7.77E-06	4.99E-05	2.73649
AC06875	-1.7688	#NAME?	-4.6608	7.81E-06	5.01E-05	3.06875
SFTPB	1.88057	6.02259	4.45683	1.09E-05	6.87E-05	2.2593
AC00862	1.51967	#NAME?	4.62276	1.18E-05	7.37E-05	2.82106
CHP2	-1.518	#NAME?	-4.4161	1.32E-05	8.17E-05	2.10654
SFTA2	1.54755	#NAME?	4.38959	1.49E-05	9.10E-05	1.97887
DMBT1	1.5993	#NAME?	4.33777	1.85E-05	0.00011	1.76158
HP	1.50641	#NAME?	4.32778	1.93E-05	0.00012	1.7204
RNA5-8S	7.29241	#NAME?	8.8265	2.62E-05	0.00015	-0.3358
CCAT1	-1.6669	#NAME?	-4.2005	3.36E-05	0.00019	1.23151
OTOP3	-1.8301	#NAME?	-4.1994	3.55E-05	0.0002	1.31713
CPLX2	-1.7592	#NAME?	-4.0888	5.45E-05	0.0003	0.80636
SHISA9	-1.5857	#NAME?	-4.0182	7.24E-05	0.00039	0.54204
FGG	1.63212	#NAME?	3.98063	8.30E-05	0.00044	0.35716
AC00554	-2.3209	#NAME?	-4.2123	0.00011	0.00056	1.07064
AC10677	-1.5047	#NAME?	-3.9571	0.00012	0.0006	0.51016
AC00715	2.16388	#NAME?	4.01546	0.00014	0.00071	0.68279
LINC011	-1.6774	#NAME?	-3.8474	0.00016	0.00081	0.05544
TMEM13	-1.7303	#NAME?	-3.7179	0.00026	0.00125	-0.3595
SFTPA1	1.66202	#NAME?	3.66945	0.00028	0.00133	-0.8025
AC10886	2.21012	#NAME?	3.79207	0.00031	0.00146	-0.1071
RNA5-8S	2.93389	#NAME?	5.08926	0.00051	0.00231	-0.4497
SFTPA2	1.56152	#NAME?	3.48844	0.00054	0.00244	-1.4285
GSTA8P	-1.5684	#NAME?	-3.4784	0.00058	0.00258	-1.3222
SAGE1	-1.733	#NAME?	-3.4844	0.00059	0.00262	-1.294
SFTPC	1.6869	#NAME?	3.45569	0.00061	0.00271	-1.5386
CTCFL	-1.7042	#NAME?	-3.4453	0.00065	0.00288	-1.481
BNIP3P6	2.20755	#NAME?	4.24746	0.00084	0.00363	-0.4399
AC11448	1.55863	#NAME?	3.42901	0.00092	0.0039	-1.2251
LINC002	-2.2979	#NAME?	-3.3743	0.00093	0.00396	-1.5173
RP4-669	2.81605	#NAME?	3.65408	0.00134	0.0055	-0.9404
TUSC7	-1.7866	#NAME?	-3.17	0.00195	0.00771	-1.9147
AC24550	-4.5653	#NAME?	-5.2639	0.00217	0.00846	-2.153
OR10AE1	-4.682	#NAME?	-5.1611	0.00239	0.00922	-2.1875
AC11474	-4.4768	#NAME?	-5.1539	0.0024	0.00927	-2.1899
LINC0211	-3.8804	#NAME?	-4.6219	0.00267	0.0102	-1.7363
AC01058	-4.3499	#NAME?	-4.8015	0.00337	0.01256	-2.318
LINC0252	-1.7408	#NAME?	-2.9715	0.00351	0.01299	-2.5838
RNU4ATA	-3.6938	#NAME?	-4.3523	0.00366	0.01345	-2.2289
AC10690	-4.5099	#NAME?	-4.676	0.00382	0.014	-2.3675
AC00632	1.5787	#NAME?	3.13851	0.0039	0.01426	-1.8814
PQLC1P1	-4.1787	#NAME?	-4.6247	0.00403	0.01467	-2.3884
AC01008	-2.1524	#NAME?	-3.8654	0.00403	0.01468	-1.8579
AC01871	-5.2296	#NAME?	-5.1001	0.00438	0.01583	-2.6241
AC11100	-5.0936	#NAME?	-5.0173	0.00468	0.0168	-2.6453
AC00851	-1.5574	#NAME?	-2.866	0.00489	0.01745	-2.7457
LINC0277	5.85785	#NAME?	3.61699	0.00492	0.01754	-2.258
AC01873	3.54374	#NAME?	3.86122	0.00511	0.01816	-2.3342
IFNA12P	-3.9613	#NAME?	-3.8436	0.00524	0.01856	-2.3473
AL13874	2.10204	#NAME?	3.09739	0.00551	0.01941	-2.3008
AC09951	-3.2653	#NAME?	-3.6395	0.00568	0.01993	-2.0956
TPTE	-1.5521	#NAME?	-2.8306	0.00579	0.02028	-2.8521
AL13592	-2.6646	#NAME?	-3.0315	0.0058	0.02032	-2.2853
LINC0232	-1.572	#NAME?	-2.7877	0.00609	0.0212	-3.1029
AC00520	2.7506	#NAME?	3.18342	0.00628	0.02178	-2.3838
LINC0162	-4.7116	#NAME?	-4.6636	0.0063	0.02184	-2.7437
AC09263	-2.3924	#NAME?	-2.9186	0.00652	0.02251	-2.467
AC00639	-3.7328	#NAME?	-4.1517	0.0066	0.02276	-2.599
AL35514	-4.5184	#NAME?	-4.4871	0.00735	0.025	-2.798
AC07405	-2.4347	#NAME?	-3.5706	0.00769	0.02604	-2.2797
AC23980	-2.9693	#NAME?	-3.9583	0.00816	0.02741	-2.5093
LINC0261	-4.5461	#NAME?	-4.3618	0.00822	0.02758	-2.8389
H2AB2	4.3578	#NAME?	4.35831	0.00824	0.02765	-2.8401
RN7SKP2	-3.4366	#NAME?	-3.8832	0.00887	0.02947	-2.7344
AP00538	-2.7271	#NAME?	-3.2396	0.00918	0.03037	-2.4393
PRAMEF1	3.02389	#NAME?	3.59674	0.00935	0.0309	-2.6992
OR4K13	-4.3701	#NAME?	-4.0857	0.0106	0.03446	-2.9364
SSU72P3	3.93474	#NAME?	2.8949	0.01069	0.03472	-2.7341
RN7SKP4	3.12871	#NAME?	2.95066	0.01147	0.03689	-2.7728
AC09243	2.00877	#NAME?	2.91463	0.01149	0.03696	-2.7767
AP00535	-3.9923	#NAME?	-3.9911	0.01159	0.03723	-2.9722
AC02120	-1.5202	#NAME?	-2.6539	0.01169	0.03753	-3.072
AL59029	-3.9173	#NAME?	-3.9156	0.01246	0.03968	-3.0018
RBMY1A1	-1.8844	#NAME?	-2.8006	0.01298	0.0411	-2.7971
RNU1-34	-3.8017	#NAME?	-3.3283	0.01333	0.04208	-2.8919
AC02754	-3.9231	#NAME?	-3.7702	0.01436	0.04487	-3.0613
LINC0242	-2.9657	#NAME?	-3.4523	0.01458	0.04548	-2.9785
AC00486	-3.2776	#NAME?	-3.4453	0.0147	0.0458	-2.9828
AC02725	-2.3543	#NAME?	-2.6229	0.01471	0.04581	-3.0542
AL15998	-1.9951	#NAME?	-2.6296	0.0154	0.04768	-3.0808
AL39081	-3.7142	#NAME?	-3.6979	0.01543	0.04776	-3.0921
OR4C14P	-5.4777	#NAME?	-4.2198	0.01551	0.04799	-3.3325
AC09007	-3.735	#NAME?	-3.6786	0.01573	0.04856	-3.1005
FABP3P2	-2.6859	#NAME?	-2.9021	0.01619	0.04976	-2.8612
CR38157	2.70741	#NAME?	2.97037	0.0162	0.04977	-2.9859

Table-S5: ICI signature genes A and B

Symbol	Group
A2M	ICI signature genes A
AADAC	ICI signature genes B
ABCA3	ICI signature genes A
ABCA6	ICI signature genes B
ABCA8	ICI signature genes B
ABCD2	ICI signature genes B
ABI3	ICI signature genes A
ABI3BP	ICI signature genes A
AC002463.1	ICI signature genes B
AC003006.1	ICI signature genes B
AC004160.1	ICI signature genes B
AC004494.1	ICI signature genes B
AC004585.1	ICI signature genes A
AC004687.1	ICI signature genes A
AC004847.1	ICI signature genes A
AC004866.2	ICI signature genes B
AC004921.1	ICI signature genes B
AC004988.1	ICI signature genes B
AC005064.1	ICI signature genes B
AC005204.1	ICI signature genes B
AC005336.1	ICI signature genes B
AC005515.1	ICI signature genes B
AC005549.1	ICI signature genes B
AC005722.3	ICI signature genes B
AC005747.1	ICI signature genes A
AC006033.2	ICI signature genes B
AC006328.1	ICI signature genes B
AC006369.1	ICI signature genes B
AC006390.1	ICI signature genes B
AC007159.1	ICI signature genes B
AC007278.1	ICI signature genes B
AC007384.1	ICI signature genes B
AC007569.1	ICI signature genes B
AC007728.2	ICI signature genes B
AC007991.2	ICI signature genes B
AC008011.2	ICI signature genes B
AC008033.3	ICI signature genes B
AC008105.3	ICI signature genes A
AC008268.1	ICI signature genes B
AC008517.1	ICI signature genes B
AC008620.2	ICI signature genes B
AC008760.2	ICI signature genes B
AC008957.1	ICI signature genes B
AC009055.2	ICI signature genes B
AC009093.1	ICI signature genes B
AC009133.3	ICI signature genes B
AC010086.2	ICI signature genes B
AC010247.2	ICI signature genes B
AC010451.3	ICI signature genes B
AC010587.2	ICI signature genes B
AC010991.1	ICI signature genes B
AC011511.5	ICI signature genes B
AC011897.1	ICI signature genes B
AC011899.2	ICI signature genes B
AC012236.1	ICI signature genes A
AC012645.3	ICI signature genes B

AC015911.3	ICI signature genes B
AC015911.7	ICI signature genes A
AC015911.8	ICI signature genes B
AC018717.1	ICI signature genes B
AC018730.4	ICI signature genes B
AC018755.4	ICI signature genes A
AC018767.2	ICI signature genes B
AC019103.1	ICI signature genes B
AC019176.1	ICI signature genes B
AC020659.1	ICI signature genes B
AC021205.2	ICI signature genes B
AC022126.1	ICI signature genes B
AC022164.1	ICI signature genes B
AC022509.2	ICI signature genes B
AC023301.1	ICI signature genes B
AC023824.4	ICI signature genes B
AC024257.3	ICI signature genes B
AC024560.2	ICI signature genes B
AC026369.3	ICI signature genes A
AC026396.1	ICI signature genes B
AC027250.1	ICI signature genes B
AC027541.1	ICI signature genes B
AC034105.1	ICI signature genes A
AC034199.1	ICI signature genes B
AC034238.2	ICI signature genes B
AC037198.1	ICI signature genes B
AC041040.1	ICI signature genes B
AC055748.1	ICI signature genes B
AC064805.1	ICI signature genes B
AC066614.1	ICI signature genes B
AC068756.1	ICI signature genes B
AC069228.1	ICI signature genes B
AC073429.2	ICI signature genes B
AC074052.2	ICI signature genes B
AC079298.3	ICI signature genes B
AC079325.6	ICI signature genes B
AC087283.2	ICI signature genes B
AC087286.2	ICI signature genes B
AC090079.2	ICI signature genes B
AC090125.1	ICI signature genes B
AC090136.3	ICI signature genes B
AC090559.1	ICI signature genes B
AC091817.1	ICI signature genes B
AC091820.2	ICI signature genes B
AC091941.1	ICI signature genes B
AC092145.1	ICI signature genes B
AC092439.1	ICI signature genes B
AC092634.2	ICI signature genes B
AC092865.2	ICI signature genes B
AC093496.1	ICI signature genes B
AC093583.1	ICI signature genes B
AC093716.1	ICI signature genes B
AC093730.1	ICI signature genes B
AC096531.2	ICI signature genes B
AC096734.1	ICI signature genes B
AC097521.1	ICI signature genes B
AC099066.2	ICI signature genes B
AC099511.1	ICI signature genes B

AC099524.1	ICI signature genes B
AC103563.1	ICI signature genes A
AC103563.3	ICI signature genes A
AC105460.1	ICI signature genes B
AC105914.1	ICI signature genes B
AC106771.1	ICI signature genes B
AC106881.1	ICI signature genes B
AC106882.1	ICI signature genes B
AC106901.1	ICI signature genes B
AC108082.1	ICI signature genes B
AC108868.2	ICI signature genes B
AC110285.2	ICI signature genes B
AC110995.1	ICI signature genes B
AC111000.3	ICI signature genes B
AC111149.2	ICI signature genes B
AC112721.1	ICI signature genes B
AC112721.2	ICI signature genes B
AC113346.1	ICI signature genes B
AC114485.1	ICI signature genes B
AC114489.2	ICI signature genes B
AC114741.1	ICI signature genes B
AC114812.2	ICI signature genes B
AC117500.5	ICI signature genes B
AC117569.1	ICI signature genes B
AC118282.4	ICI signature genes B
AC119044.1	ICI signature genes B
AC121342.1	ICI signature genes B
AC124944.1	ICI signature genes B
AC126177.1	ICI signature genes B
AC127521.1	ICI signature genes B
AC131055.2	ICI signature genes B
AC133065.3	ICI signature genes A
AC133644.2	ICI signature genes B
AC134312.5	ICI signature genes B
AC134879.2	ICI signature genes A
AC135068.2	ICI signature genes A
AC135068.8	ICI signature genes A
AC136428.1	ICI signature genes A
AC139491.2	ICI signature genes B
AC139491.3	ICI signature genes B
AC142381.1	ICI signature genes A
AC145098.1	ICI signature genes A
AC147651.4	ICI signature genes A
AC233280.2	ICI signature genes B
AC233702.5	ICI signature genes B
AC237221.2	ICI signature genes B
AC239802.1	ICI signature genes B
AC242852.2	ICI signature genes B
AC243829.4	ICI signature genes B
AC243960.1	ICI signature genes A
AC243960.3	ICI signature genes B
AC244205.1	ICI signature genes B
AC244250.2	ICI signature genes A
AC244250.4	ICI signature genes A
AC245088.1	ICI signature genes B
AC245128.3	ICI signature genes B
AC245297.1	ICI signature genes A
AC245505.3	ICI signature genes B

AC246787.2	ICI signature genes B
AC246787.3	ICI signature genes A
AC246787.4	ICI signature genes A
ACKR1	ICI signature genes A
ACOD1	ICI signature genes B
ACOXL	ICI signature genes B
ACSL5	ICI signature genes A
ACSL6	ICI signature genes B
ACSM5	ICI signature genes B
ACTA2	ICI signature genes A
ACTA2-AS1	ICI signature genes B
ACTG1P22	ICI signature genes B
ACTG2	ICI signature genes A
ADA2	ICI signature genes A
ADAM12	ICI signature genes A
ADAM19	ICI signature genes A
ADAM8	ICI signature genes A
ADAMDEC1	ICI signature genes A
ADAMTS12	ICI signature genes A
ADAMTS16	ICI signature genes A
ADAMTS2	ICI signature genes A
ADAMTS4	ICI signature genes A
ADAMTS5	ICI signature genes B
ADAMTS7P4	ICI signature genes B
ADAMTS8	ICI signature genes B
ADAMTS9-AS1	ICI signature genes B
ADCY2	ICI signature genes B
ADCYAP1	ICI signature genes B
ADGRE1	ICI signature genes B
ADGRE2	ICI signature genes A
ADGRE3	ICI signature genes B
ADGRE4P	ICI signature genes B
ADGRG5	ICI signature genes A
ADH1B	ICI signature genes A
ADH7	ICI signature genes B
ADORA1	ICI signature genes B
ADRA1B	ICI signature genes B
ADRA2A	ICI signature genes A
AEBP1	ICI signature genes A
AF127936.1	ICI signature genes B
AF127936.5	ICI signature genes B
AF165138.7	ICI signature genes B
AGER	ICI signature genes A
AGTR1	ICI signature genes B
AICDA	ICI signature genes B
AIF1	ICI signature genes A
AIM2	ICI signature genes A
AK5	ICI signature genes B
AKAP2	ICI signature genes B
AKR1B10	ICI signature genes B
AKR1B10P1	ICI signature genes B
AKR1C1	ICI signature genes B
AKR1C2	ICI signature genes B
AKR1C3	ICI signature genes B
AKR1C4	ICI signature genes B
AL021395.1	ICI signature genes B
AL023653.1	ICI signature genes B
AL031595.1	ICI signature genes B

AL031713.1	ICI signature genes B
AL031767.1	ICI signature genes B
AL031846.1	ICI signature genes B
AL033397.1	ICI signature genes B
AL033397.2	ICI signature genes B
AL035425.4	ICI signature genes B
AL049649.1	ICI signature genes B
AL079307.2	ICI signature genes B
AL096816.1	ICI signature genes B
AL109918.1	ICI signature genes B
AL121985.1	ICI signature genes B
AL122127.2	ICI signature genes A
AL133325.3	ICI signature genes B
AL133371.2	ICI signature genes A
AL133410.1	ICI signature genes B
AL133467.1	ICI signature genes A
AL135929.1	ICI signature genes B
AL137186.1	ICI signature genes B
AL138745.1	ICI signature genes B
AL138826.1	ICI signature genes B
AL139125.1	ICI signature genes B
AL139147.1	ICI signature genes B
AL139246.3	ICI signature genes B
AL157871.2	ICI signature genes B
AL158071.3	ICI signature genes B
AL159987.2	ICI signature genes B
AL161431.1	ICI signature genes B
AL162414.1	ICI signature genes B
AL353093.1	ICI signature genes B
AL355143.1	ICI signature genes B
AL356277.2	ICI signature genes B
AL356417.2	ICI signature genes B
AL356750.1	ICI signature genes B
AL357033.3	ICI signature genes B
AL359792.1	ICI signature genes B
AL365361.1	ICI signature genes A
AL390816.3	ICI signature genes B
AL391358.1	ICI signature genes B
AL391427.1	ICI signature genes B
AL391427.3	ICI signature genes B
AL442635.1	ICI signature genes B
AL512306.2	ICI signature genes B
AL589863.1	ICI signature genes B
AL589987.1	ICI signature genes B
AL590290.1	ICI signature genes B
AL590422.1	ICI signature genes B
AL590764.1	ICI signature genes B
AL592309.2	ICI signature genes B
AL606807.1	ICI signature genes B
AL691447.2	ICI signature genes B
AL845311.1	ICI signature genes B
AL928768.3	ICI signature genes A
ALDH3A1	ICI signature genes B
ALOX5	ICI signature genes A
ALOX5AP	ICI signature genes A
ALPK2	ICI signature genes B
ALPP	ICI signature genes B
AMPD1	ICI signature genes B

AMTN	ICI signature genes B
ANGPTL1	ICI signature genes B
ANGPTL2	ICI signature genes A
ANK2	ICI signature genes B
ANKRD1	ICI signature genes A
ANKRD18B	ICI signature genes B
ANKRD36BP2	ICI signature genes A
ANKRD44-AS1	ICI signature genes A
ANKRD55	ICI signature genes B
ANO5	ICI signature genes B
ANTXRLP1	ICI signature genes B
ANXA6	ICI signature genes A
AOAH	ICI signature genes A
AOC3	ICI signature genes A
AOX1	ICI signature genes A
AP000892.4	ICI signature genes B
AP001107.5	ICI signature genes B
AP001189.3	ICI signature genes B
AP001434.1	ICI signature genes B
AP001604.1	ICI signature genes B
AP001652.1	ICI signature genes B
AP003071.4	ICI signature genes B
AP003119.1	ICI signature genes B
AP004550.1	ICI signature genes B
AP005357.1	ICI signature genes B
AP005380.1	ICI signature genes B
APBB1IP	ICI signature genes A
APCDD1L	ICI signature genes B
APCDD1L-DT	ICI signature genes B
APELA	ICI signature genes B
APLNR	ICI signature genes A
APOA4	ICI signature genes B
APOC1	ICI signature genes A
APOC1P1	ICI signature genes B
APOC2	ICI signature genes B
APOE	ICI signature genes A
APOL3	ICI signature genes A
AQP4	ICI signature genes A
AQP9	ICI signature genes A
ARHGAP15	ICI signature genes A
ARHGAP31	ICI signature genes A
ARHGAP6	ICI signature genes B
ARHGAP9	ICI signature genes A
ARHGEF38	ICI signature genes B
ARHGEF6	ICI signature genes A
ARMH1	ICI signature genes B
ARRDC5	ICI signature genes B
ART4	ICI signature genes B
ARTN	ICI signature genes B
ASGR2	ICI signature genes B
ASPN	ICI signature genes A
ASXL3	ICI signature genes B
ATP10A	ICI signature genes A
ATP13A4-AS1	ICI signature genes B
ATP6V0D2	ICI signature genes A
ATP8A1	ICI signature genes A
ATP8B4	ICI signature genes B
AXL	ICI signature genes A

B3GAT1	ICI signature genes B
BAAT	ICI signature genes B
BATF	ICI signature genes A
BATF2	ICI signature genes A
BCL2A1	ICI signature genes A
BCL2L14	ICI signature genes B
BCORP1	ICI signature genes B
BFSP2	ICI signature genes B
BGN	ICI signature genes A
BHLHE22	ICI signature genes B
BHLHE41	ICI signature genes A
BICC1	ICI signature genes A
BIN2	ICI signature genes A
BIRC3	ICI signature genes A
BIRC7	ICI signature genes B
BLK	ICI signature genes A
BMP5	ICI signature genes B
BNC2	ICI signature genes B
BNIP3P6	ICI signature genes B
BRINP3	ICI signature genes B
BST1	ICI signature genes B
BTK	ICI signature genes A
BTLA	ICI signature genes B
BX088651.2	ICI signature genes B
C11orf21	ICI signature genes B
C11orf96	ICI signature genes A
C15orf48	ICI signature genes A
C16orf54	ICI signature genes A
C16orf89	ICI signature genes A
C19orf38	ICI signature genes A
C1orf162	ICI signature genes A
C1orf61	ICI signature genes B
C1QA	ICI signature genes A
C1QB	ICI signature genes A
C1QC	ICI signature genes A
C1QTNF1	ICI signature genes A
C1QTNF3	ICI signature genes A
C1QTNF7	ICI signature genes B
C1S	ICI signature genes A
C2	ICI signature genes A
C3	ICI signature genes A
C3AR1	ICI signature genes A
C3orf80	ICI signature genes B
C4B	ICI signature genes A
C4BPA	ICI signature genes A
C4BPB	ICI signature genes B
C5AR1	ICI signature genes A
C5AR2	ICI signature genes B
C5orf46	ICI signature genes B
C7	ICI signature genes A
C9orf139	ICI signature genes B
CA12	ICI signature genes B
CA4	ICI signature genes B
CACNA1B	ICI signature genes B
CADM3	ICI signature genes A
CALHM5	ICI signature genes B
CALHM6	ICI signature genes A
CAMK1G	ICI signature genes B

CAPN6	ICI signature genes A
CAPN8	ICI signature genes A
CARMIL2	ICI signature genes A
CASC19	ICI signature genes B
CASKIN1	ICI signature genes B
CASP14	ICI signature genes B
CASP5	ICI signature genes B
CASS4	ICI signature genes A
CATSPER1	ICI signature genes B
CBLN4	ICI signature genes B
CCAT1	ICI signature genes B
CCBE1	ICI signature genes B
CCDC141	ICI signature genes B
CCDC162P	ICI signature genes B
CCDC170	ICI signature genes A
CCDC69	ICI signature genes A
CCDC8	ICI signature genes B
CCDC80	ICI signature genes A
CCL11	ICI signature genes A
CCL13	ICI signature genes A
CCL14	ICI signature genes B
CCL18	ICI signature genes A
CCL19	ICI signature genes A
CCL2	ICI signature genes A
CCL21	ICI signature genes A
CCL22	ICI signature genes A
CCL23	ICI signature genes A
CCL25	ICI signature genes B
CCL3	ICI signature genes A
CCL3L3	ICI signature genes A
CCL4	ICI signature genes A
CCL4L2	ICI signature genes A
CCL5	ICI signature genes A
CCL7	ICI signature genes B
CCL8	ICI signature genes A
CCN1	ICI signature genes A
CCN2	ICI signature genes A
CCN4	ICI signature genes A
CCN5	ICI signature genes A
CCR1	ICI signature genes A
CCR2	ICI signature genes A
CCR4	ICI signature genes A
CCR5	ICI signature genes A
CCR5AS	ICI signature genes B
CCR6	ICI signature genes B
CCR7	ICI signature genes A
CCR8	ICI signature genes B
CCRL2	ICI signature genes A
CD14	ICI signature genes A
CD163	ICI signature genes A
CD180	ICI signature genes A
CD19	ICI signature genes A
CD1B	ICI signature genes B
CD1C	ICI signature genes A
CD1E	ICI signature genes A
CD2	ICI signature genes A
CD209	ICI signature genes A
CD22	ICI signature genes A

CD226	ICI signature genes B
CD244	ICI signature genes B
CD247	ICI signature genes A
CD248	ICI signature genes A
CD27	ICI signature genes A
CD28	ICI signature genes A
CD300A	ICI signature genes A
CD300C	ICI signature genes A
CD300E	ICI signature genes A
CD300LB	ICI signature genes B
CD300LF	ICI signature genes A
CD300LG	ICI signature genes B
CD33	ICI signature genes B
CD37	ICI signature genes A
CD3D	ICI signature genes A
CD3E	ICI signature genes A
CD3G	ICI signature genes A
CD4	ICI signature genes A
CD40LG	ICI signature genes A
CD48	ICI signature genes A
CD5	ICI signature genes A
CD52	ICI signature genes A
CD53	ICI signature genes A
CD6	ICI signature genes A
CD69	ICI signature genes A
CD7	ICI signature genes A
CD70	ICI signature genes A
CD72	ICI signature genes A
CD74	ICI signature genes A
CD79A	ICI signature genes A
CD79B	ICI signature genes A
CD80	ICI signature genes B
CD84	ICI signature genes A
CD86	ICI signature genes A
CD8A	ICI signature genes A
CD8B	ICI signature genes A
CD93	ICI signature genes A
CD96	ICI signature genes A
CDH11	ICI signature genes A
CDH13	ICI signature genes B
CDH2	ICI signature genes B
CDH8	ICI signature genes B
CDK15	ICI signature genes B
CDO1	ICI signature genes B
CEACAM21	ICI signature genes A
CEACAM4	ICI signature genes B
CEL	ICI signature genes B
CELF2	ICI signature genes A
CES1P2	ICI signature genes B
CFAP221	ICI signature genes B
CFP	ICI signature genes A
CHEK2P5	ICI signature genes B
CHGA	ICI signature genes B
CHI3L1	ICI signature genes A
CHI3L2	ICI signature genes A
CHIA	ICI signature genes B
CHIAP2	ICI signature genes B
CHIT1	ICI signature genes A

CHP2	ICI signature genes B
CHRDL1	ICI signature genes A
CHRDL2	ICI signature genes A
CHRM3-AS2	ICI signature genes B
CHRNA1	ICI signature genes B
CHRNA6	ICI signature genes B
CHST11	ICI signature genes A
CIITA	ICI signature genes A
CILP	ICI signature genes A
CILP2	ICI signature genes A
CKMT1A	ICI signature genes B
CKMT1B	ICI signature genes B
CLC	ICI signature genes B
CLDN18	ICI signature genes A
CLEC10A	ICI signature genes A
CLEC12A	ICI signature genes A
CLEC12B	ICI signature genes B
CLEC17A	ICI signature genes A
CLEC1A	ICI signature genes A
CLEC4A	ICI signature genes A
CLEC4C	ICI signature genes B
CLEC4D	ICI signature genes B
CLEC4E	ICI signature genes A
CLEC4G	ICI signature genes B
CLEC5A	ICI signature genes A
CLEC6A	ICI signature genes B
CLEC7A	ICI signature genes A
CLEC9A	ICI signature genes B
CLECL1	ICI signature genes B
CLIC5	ICI signature genes A
CLMP	ICI signature genes A
CLNK	ICI signature genes B
CLSTN2	ICI signature genes B
CMKLR1	ICI signature genes A
CNN1	ICI signature genes A
CNN2P11	ICI signature genes B
CNNM1	ICI signature genes B
CNR2	ICI signature genes B
CNRIP1	ICI signature genes A
CNTN3	ICI signature genes B
CNTN6	ICI signature genes B
COL10A1	ICI signature genes A
COL11A1	ICI signature genes A
COL12A1	ICI signature genes A
COL14A1	ICI signature genes A
COL15A1	ICI signature genes A
COL16A1	ICI signature genes A
COL17A1	ICI signature genes B
COL1A1	ICI signature genes A
COL1A2	ICI signature genes A
COL22A1	ICI signature genes B
COL2A1	ICI signature genes B
COL3A1	ICI signature genes A
COL4A3	ICI signature genes A
COL4A4	ICI signature genes A
COL5A1	ICI signature genes A
COL5A2	ICI signature genes A
COL5A3	ICI signature genes A

COL6A1	ICI signature genes A
COL6A2	ICI signature genes A
COL6A3	ICI signature genes A
COL6A5	ICI signature genes B
COL6A6	ICI signature genes A
COL8A1	ICI signature genes A
COL8A2	ICI signature genes A
COLEC12	ICI signature genes A
COMP	ICI signature genes A
COPDA1	ICI signature genes A
CORIN	ICI signature genes B
CORO1A	ICI signature genes A
CPA3	ICI signature genes A
CPA4	ICI signature genes B
CPED1	ICI signature genes B
CPLX2	ICI signature genes B
CPM	ICI signature genes A
CPNE5	ICI signature genes A
CPXM1	ICI signature genes A
CPZ	ICI signature genes B
CR1	ICI signature genes B
CR1L	ICI signature genes B
CR2	ICI signature genes A
CR381572.1	ICI signature genes B
CRB2	ICI signature genes B
CREB3L1	ICI signature genes A
CRHR2	ICI signature genes B
CRIP1	ICI signature genes A
CRISPLD2	ICI signature genes A
CRTAM	ICI signature genes A
CRYBB1	ICI signature genes B
CSDC2	ICI signature genes B
CSF1R	ICI signature genes A
CSF2	ICI signature genes B
CSF2RA	ICI signature genes A
CSF2RB	ICI signature genes A
CSF3R	ICI signature genes A
CSMD2	ICI signature genes B
CSPG4	ICI signature genes B
CST1	ICI signature genes A
CST7	ICI signature genes A
CTC-251I16.1	ICI signature genes A
CTCFL	ICI signature genes B
CTHRC1	ICI signature genes A
CTLA4	ICI signature genes A
CTSE	ICI signature genes A
CTSK	ICI signature genes A
CTSS	ICI signature genes A
CTSW	ICI signature genes A
CX3CR1	ICI signature genes B
CXCL10	ICI signature genes A
CXCL11	ICI signature genes A
CXCL12	ICI signature genes A
CXCL13	ICI signature genes A
CXCL5	ICI signature genes A
CXCL9	ICI signature genes A
CXCR1	ICI signature genes B
CXCR2P1	ICI signature genes A

CXCR3	ICI signature genes A
CXCR4	ICI signature genes A
CXCR5	ICI signature genes B
CXCR6	ICI signature genes A
CXorf21	ICI signature genes A
CXorf65	ICI signature genes B
CYBB	ICI signature genes A
CYP1B1	ICI signature genes A
CYP27A1	ICI signature genes A
CYP2B7P	ICI signature genes A
CYP4B1	ICI signature genes A
CYP4F11	ICI signature genes B
CYP4F26P	ICI signature genes B
CYP4F29P	ICI signature genes B
CYP4F3	ICI signature genes B
CYP4F35P	ICI signature genes B
CYS1	ICI signature genes B
CYSLTR1	ICI signature genes A
CYTH4	ICI signature genes A
CYTIP	ICI signature genes A
CYTL1	ICI signature genes A
DAAM2	ICI signature genes A
DAB2	ICI signature genes A
DACT1	ICI signature genes A
DACT3	ICI signature genes B
DBH	ICI signature genes B
DBH-AS1	ICI signature genes A
DCN	ICI signature genes A
DCSTAMP	ICI signature genes B
DDO	ICI signature genes B
DDR2	ICI signature genes A
DEFA11P	ICI signature genes B
DEFA7P	ICI signature genes B
DEPP1	ICI signature genes A
DERL3	ICI signature genes A
DES	ICI signature genes A
DIO2	ICI signature genes B
DIO3	ICI signature genes B
DIPK1C	ICI signature genes B
DKK2	ICI signature genes B
DLC1	ICI signature genes A
DLG2	ICI signature genes B
DLX1	ICI signature genes B
DLX2	ICI signature genes B
DLX6-AS1	ICI signature genes B
DMBT1	ICI signature genes A
DMRT2	ICI signature genes B
DMRT3	ICI signature genes B
DMRTA2	ICI signature genes B
DNAH8	ICI signature genes B
DNAJB3	ICI signature genes B
DNAJC5B	ICI signature genes B
DNASE2B	ICI signature genes B
DNM3OS	ICI signature genes B
DOCK10	ICI signature genes A
DOCK11	ICI signature genes A
DOCK2	ICI signature genes A
DOCK8	ICI signature genes A

DOK2	ICI signature genes A
DOK3	ICI signature genes A
DOK5	ICI signature genes B
DPEP1	ICI signature genes A
DPEP2	ICI signature genes A
DPP4	ICI signature genes A
DPT	ICI signature genes A
DTHD1	ICI signature genes B
DTNB-AS1	ICI signature genes A
EBI3	ICI signature genes A
ECM2	ICI signature genes A
EDNRA	ICI signature genes A
ELN	ICI signature genes A
EMB	ICI signature genes A
EMILIN1	ICI signature genes A
EMILIN3	ICI signature genes B
EML4-AS1	ICI signature genes B
EMX2	ICI signature genes B
EMX2OS	ICI signature genes B
ENPP2	ICI signature genes A
ENPP6	ICI signature genes B
EOMES	ICI signature genes A
EPHA10	ICI signature genes B
EPSTI1	ICI signature genes A
EPYC	ICI signature genes B
EREG	ICI signature genes B
ERVMER61-1	ICI signature genes B
ESR1	ICI signature genes B
ETS1	ICI signature genes A
EVA1A	ICI signature genes A
EVI2A	ICI signature genes A
EVI2B	ICI signature genes A
EVPLL	ICI signature genes B
EXOC3L4	ICI signature genes A
F13A1	ICI signature genes A
F5	ICI signature genes A
FABP3P2	ICI signature genes B
FABP4	ICI signature genes B
FADS6	ICI signature genes B
FAM107A	ICI signature genes A
FAM155A	ICI signature genes B
FAM180A	ICI signature genes B
FAM198B-AS1	ICI signature genes B
FAM20A	ICI signature genes A
FAM216B	ICI signature genes A
FAM30A	ICI signature genes A
FAM3B	ICI signature genes B
FAM78A	ICI signature genes A
FAM92B	ICI signature genes A
FAP	ICI signature genes A
FAR2P1	ICI signature genes B
FASLG	ICI signature genes A
FAT2	ICI signature genes B
FAT4	ICI signature genes B
FBLN2	ICI signature genes A
FBLN5	ICI signature genes A
FBN1	ICI signature genes A
FBP1	ICI signature genes A

FCAR	ICI signature genes B
FCER1A	ICI signature genes A
FCER1G	ICI signature genes A
FCER2	ICI signature genes A
FCGR1A	ICI signature genes A
FCGR1B	ICI signature genes B
FCGR1CP	ICI signature genes B
FCGR2A	ICI signature genes A
FCGR2B	ICI signature genes A
FCGR2C	ICI signature genes A
FCGR3A	ICI signature genes A
FCGR3B	ICI signature genes A
FCMR	ICI signature genes A
FCN1	ICI signature genes A
FCRL1	ICI signature genes A
FCRL2	ICI signature genes A
FCRL3	ICI signature genes A
FCRL4	ICI signature genes B
FCRL5	ICI signature genes A
FCRL6	ICI signature genes B
FCRLA	ICI signature genes A
FDCSP	ICI signature genes A
FERMT3	ICI signature genes A
FEZF1	ICI signature genes B
FFAR4	ICI signature genes B
FGA	ICI signature genes A
FGD2	ICI signature genes A
FGD5	ICI signature genes A
FGF10	ICI signature genes B
FGF5	ICI signature genes B
FGF7	ICI signature genes A
FGG	ICI signature genes A
FGL2	ICI signature genes A
FGR	ICI signature genes A
FHL5	ICI signature genes B
FIBCD1	ICI signature genes B
FIBIN	ICI signature genes A
FILIP1L	ICI signature genes A
FLI1	ICI signature genes A
FLT3	ICI signature genes B
FMOD	ICI signature genes A
FN1	ICI signature genes A
FNDC1	ICI signature genes A
FOLR1	ICI signature genes A
FOLR2	ICI signature genes A
FOXC2	ICI signature genes B
FOXH1	ICI signature genes B
FOXL2	ICI signature genes B
FOXP3	ICI signature genes A
FPR1	ICI signature genes A
FPR2	ICI signature genes B
FPR3	ICI signature genes A
FREM2	ICI signature genes B
FTH1P14	ICI signature genes B
FTH1P22	ICI signature genes A
FUT7	ICI signature genes B
FXYD2	ICI signature genes B
FYB1	ICI signature genes A

G0S2	ICI signature genes A
GAB3	ICI signature genes A
GACAT1	ICI signature genes B
GALNT15	ICI signature genes B
GALNT17	ICI signature genes B
GAPT	ICI signature genes A
GAS1	ICI signature genes A
GASK1B	ICI signature genes A
GBP1	ICI signature genes A
GBP4	ICI signature genes A
GBP5	ICI signature genes A
GCSAML	ICI signature genes B
GDF10	ICI signature genes B
GDNF	ICI signature genes B
GEM	ICI signature genes A
GFPT2	ICI signature genes A
GFRA1	ICI signature genes B
GGTA1P	ICI signature genes A
GGTLC1	ICI signature genes A
GIMAP1	ICI signature genes A
GIMAP2	ICI signature genes A
GIMAP4	ICI signature genes A
GIMAP5	ICI signature genes B
GIMAP6	ICI signature genes A
GIMAP7	ICI signature genes A
GIMAP8	ICI signature genes A
GJA5	ICI signature genes A
GJB2	ICI signature genes B
GJB6	ICI signature genes B
GKN2	ICI signature genes A
GLIS1	ICI signature genes B
GLT8D2	ICI signature genes A
GMFG	ICI signature genes A
GNG2	ICI signature genes A
GNGT2	ICI signature genes A
GNLY	ICI signature genes A
GOLGA6L7	ICI signature genes B
GOLGA8EP	ICI signature genes B
GOLT1A	ICI signature genes B
GP5	ICI signature genes B
GPA33	ICI signature genes B
GPC6	ICI signature genes A
GPIHBP1	ICI signature genes A
GPM6A	ICI signature genes B
GPR1	ICI signature genes B
GPR132	ICI signature genes A
GPR141	ICI signature genes B
GPR15	ICI signature genes A
GPR171	ICI signature genes A
GPR174	ICI signature genes A
GPR18	ICI signature genes A
GPR183	ICI signature genes A
GPR25	ICI signature genes B
GPR34	ICI signature genes A
GPR55	ICI signature genes B
GPR65	ICI signature genes A
GPR68	ICI signature genes A
GPR82	ICI signature genes B

GPR84	ICI signature genes A
GPR88	ICI signature genes B
GPRIN3	ICI signature genes A
GPSM3	ICI signature genes A
GPX2	ICI signature genes B
GRAP	ICI signature genes B
GRAP2	ICI signature genes B
GREM1	ICI signature genes A
GRIA3	ICI signature genes B
GRP	ICI signature genes B
GSTA1	ICI signature genes B
GSTA8P	ICI signature genes B
GSTA9P	ICI signature genes B
GUCA1A	ICI signature genes B
GVINP1	ICI signature genes A
GXYLT2	ICI signature genes A
GYPC	ICI signature genes A
GZMA	ICI signature genes A
GZMB	ICI signature genes A
GZMH	ICI signature genes A
GZMK	ICI signature genes A
GZMM	ICI signature genes A
H2AB2	ICI signature genes B
HAMP	ICI signature genes B
HAP1	ICI signature genes B
HAPLN1	ICI signature genes B
HAS1	ICI signature genes B
HAS2	ICI signature genes A
HAVCR2	ICI signature genes A
HCFC2P1	ICI signature genes B
HCST	ICI signature genes A
HEATR9	ICI signature genes B
HECW2-AS1	ICI signature genes B
HEPH	ICI signature genes A
HEPHL1	ICI signature genes B
HGF	ICI signature genes B
HHIP	ICI signature genes B
HK3	ICI signature genes A
HLA-DMA	ICI signature genes A
HLA-DMB	ICI signature genes A
HLA-DOA	ICI signature genes A
HLA-DOB	ICI signature genes A
HLA-DPA1	ICI signature genes A
HLA-DPA3	ICI signature genes B
HLA-DPB1	ICI signature genes A
HLA-DPB2	ICI signature genes A
HLA-DQA1	ICI signature genes A
HLA-DQA2	ICI signature genes A
HLA-DQB1	ICI signature genes A
HLA-DQB1-AS1	ICI signature genes A
HLA-DQB2	ICI signature genes A
HLA-DRA	ICI signature genes A
HLA-DRB1	ICI signature genes A
HLA-DRB5	ICI signature genes A
HLA-DRB6	ICI signature genes A
HMCN1	ICI signature genes A
HMGA1P7	ICI signature genes B
HNF1B	ICI signature genes B

HNMT	ICI signature genes A
HNRNPA1P21	ICI signature genes A
HP	ICI signature genes A
HPGDS	ICI signature genes A
HRH2	ICI signature genes B
HS3ST2	ICI signature genes A
HSD11B1	ICI signature genes A
HSH2D	ICI signature genes A
HSPB3	ICI signature genes B
HSPB7	ICI signature genes B
HTR1B	ICI signature genes B
HTR2A	ICI signature genes B
HTRA3	ICI signature genes A
HTRA4	ICI signature genes A
ICAM1	ICI signature genes A
ICAM2	ICI signature genes A
ICAM3	ICI signature genes A
ICOS	ICI signature genes A
IDO1	ICI signature genes A
IDO2	ICI signature genes B
IFI44L	ICI signature genes A
IFNA12P	ICI signature genes B
IFNG	ICI signature genes A
IFNG-AS1	ICI signature genes B
IFNWP5	ICI signature genes B
IGF1	ICI signature genes B
IGF2	ICI signature genes B
IGFL2	ICI signature genes A
IGFL2-AS1	ICI signature genes B
IGFL3	ICI signature genes B
IGHA1	ICI signature genes A
IGHA2	ICI signature genes A
IGHD	ICI signature genes A
IGHE	ICI signature genes A
IGHG1	ICI signature genes A
IGHG2	ICI signature genes A
IGHG3	ICI signature genes A
IGHG4	ICI signature genes A
IGHGP	ICI signature genes A
IGHJ1	ICI signature genes A
IGHJ2	ICI signature genes A
IGHJ3	ICI signature genes A
IGHJ3P	ICI signature genes A
IGHM	ICI signature genes A
IGHV1-12	ICI signature genes A
IGHV1-14	ICI signature genes A
IGHV1-17	ICI signature genes A
IGHV1-18	ICI signature genes A
IGHV1-2	ICI signature genes A
IGHV1-24	ICI signature genes A
IGHV1-3	ICI signature genes A
IGHV1-45	ICI signature genes A
IGHV1-46	ICI signature genes A
IGHV1-58	ICI signature genes A
IGHV1-67	ICI signature genes A
IGHV1-69	ICI signature genes A
IGHV1-69D	ICI signature genes A
IGHV1OR15-2	ICI signature genes A

IGHV1OR15-9	ICI signature genes A
IGHV1OR16-1	ICI signature genes A
IGHV1OR16-3	ICI signature genes A
IGHV1OR21-1	ICI signature genes A
IGHV2-26	ICI signature genes A
IGHV2-5	ICI signature genes A
IGHV2-70	ICI signature genes A
IGHV2-70D	ICI signature genes A
IGHV3-11	ICI signature genes A
IGHV3-13	ICI signature genes A
IGHV3-15	ICI signature genes A
IGHV3-19	ICI signature genes A
IGHV3-20	ICI signature genes A
IGHV3-21	ICI signature genes A
IGHV3-22	ICI signature genes A
IGHV3-23	ICI signature genes A
IGHV3-30	ICI signature genes A
IGHV3-33	ICI signature genes A
IGHV3-35	ICI signature genes A
IGHV3-38	ICI signature genes A
IGHV3-41	ICI signature genes A
IGHV3-43	ICI signature genes A
IGHV3-47	ICI signature genes A
IGHV3-48	ICI signature genes A
IGHV3-49	ICI signature genes A
IGHV3-52	ICI signature genes A
IGHV3-53	ICI signature genes A
IGHV3-60	ICI signature genes A
IGHV3-62	ICI signature genes A
IGHV3-63	ICI signature genes A
IGHV3-64	ICI signature genes A
IGHV3-66	ICI signature genes A
IGHV3-7	ICI signature genes A
IGHV3-71	ICI signature genes A
IGHV3-72	ICI signature genes A
IGHV3-73	ICI signature genes A
IGHV3-74	ICI signature genes A
IGHV3-75	ICI signature genes A
IGHV3-76	ICI signature genes A
IGHV3OR15-7	ICI signature genes A
IGHV3OR16-10	ICI signature genes A
IGHV3OR16-11	ICI signature genes A
IGHV3OR16-12	ICI signature genes A
IGHV3OR16-13	ICI signature genes A
IGHV3OR16-6	ICI signature genes A
IGHV3OR16-8	ICI signature genes A
IGHV3OR16-9	ICI signature genes A
IGHV4-28	ICI signature genes A
IGHV4-31	ICI signature genes A
IGHV4-34	ICI signature genes A
IGHV4-39	ICI signature genes A
IGHV4-4	ICI signature genes A
IGHV4-55	ICI signature genes A
IGHV4-59	ICI signature genes A
IGHV4-61	ICI signature genes A
IGHV4OR15-8	ICI signature genes A
IGHV5-51	ICI signature genes A
IGHV5-78	ICI signature genes A

IGHV6-1	ICI signature genes A
IGHV7-56	ICI signature genes A
IGHV7-81	ICI signature genes A
IGKC	ICI signature genes A
IGKJ5	ICI signature genes A
IGKV1-12	ICI signature genes A
IGKV1-13	ICI signature genes A
IGKV1-16	ICI signature genes A
IGKV1-17	ICI signature genes A
IGKV1-27	ICI signature genes A
IGKV1-33	ICI signature genes A
IGKV1-39	ICI signature genes A
IGKV1-5	ICI signature genes A
IGKV1-6	ICI signature genes A
IGKV1-8	ICI signature genes A
IGKV1-9	ICI signature genes A
IGKV1D-12	ICI signature genes A
IGKV1D-13	ICI signature genes A
IGKV1D-16	ICI signature genes A
IGKV1D-17	ICI signature genes A
IGKV1D-27	ICI signature genes A
IGKV1D-33	ICI signature genes A
IGKV1D-39	ICI signature genes A
IGKV1D-42	ICI signature genes A
IGKV1D-43	ICI signature genes A
IGKV1D-8	ICI signature genes A
IGKV1OR10-1	ICI signature genes A
IGKV1OR2-108	ICI signature genes A
IGKV1OR2-11	ICI signature genes A
IGKV1OR2-3	ICI signature genes A
IGKV1OR2-6	ICI signature genes A
IGKV1OR22-1	ICI signature genes A
IGKV1OR22-5	ICI signature genes A
IGKV2-24	ICI signature genes A
IGKV2-28	ICI signature genes A
IGKV2-29	ICI signature genes A
IGKV2-30	ICI signature genes A
IGKV2D-24	ICI signature genes A
IGKV2D-28	ICI signature genes A
IGKV2D-29	ICI signature genes A
IGKV2D-30	ICI signature genes A
IGKV2D-40	ICI signature genes A
IGKV2OR22-3	ICI signature genes A
IGKV2OR22-4	ICI signature genes A
IGKV3-11	ICI signature genes A
IGKV3-15	ICI signature genes A
IGKV3-20	ICI signature genes A
IGKV3-7	ICI signature genes A
IGKV3D-11	ICI signature genes A
IGKV3D-15	ICI signature genes A
IGKV3D-20	ICI signature genes A
IGKV3D-7	ICI signature genes A
IGKV3OR2-268	ICI signature genes A
IGKV3OR2-5	ICI signature genes A
IGKV4-1	ICI signature genes A
IGKV5-2	ICI signature genes A
IGKV6-21	ICI signature genes A
IGKV6D-21	ICI signature genes A

IGKV7-3	ICI signature genes A
IGLC2	ICI signature genes A
IGLC3	ICI signature genes A
IGLC6	ICI signature genes A
IGLC7	ICI signature genes A
IGLJ2	ICI signature genes A
IGLJCOR18	ICI signature genes A
IGLL1	ICI signature genes A
IGLL3P	ICI signature genes A
IGLL5	ICI signature genes A
IGLON5	ICI signature genes B
IGLV1-36	ICI signature genes A
IGLV1-40	ICI signature genes A
IGLV1-41	ICI signature genes A
IGLV1-44	ICI signature genes A
IGLV1-47	ICI signature genes A
IGLV1-50	ICI signature genes A
IGLV1-51	ICI signature genes A
IGLV10-54	ICI signature genes A
IGLV2-11	ICI signature genes A
IGLV2-14	ICI signature genes A
IGLV2-18	ICI signature genes A
IGLV2-23	ICI signature genes A
IGLV2-28	ICI signature genes A
IGLV2-33	ICI signature genes A
IGLV2-34	ICI signature genes A
IGLV2-5	ICI signature genes A
IGLV2-8	ICI signature genes A
IGLV3-1	ICI signature genes A
IGLV3-10	ICI signature genes A
IGLV3-12	ICI signature genes A
IGLV3-16	ICI signature genes A
IGLV3-19	ICI signature genes A
IGLV3-21	ICI signature genes A
IGLV3-25	ICI signature genes A
IGLV3-27	ICI signature genes A
IGLV3-9	ICI signature genes A
IGLV4-3	ICI signature genes A
IGLV4-60	ICI signature genes A
IGLV4-69	ICI signature genes A
IGLV5-37	ICI signature genes A
IGLV5-45	ICI signature genes A
IGLV5-48	ICI signature genes A
IGLV6-57	ICI signature genes A
IGLV7-43	ICI signature genes A
IGLV7-46	ICI signature genes A
IGLV8-61	ICI signature genes A
IGLV8OR8-1	ICI signature genes B
IGLV9-49	ICI signature genes A
IGLVI-70	ICI signature genes A
IGSF21	ICI signature genes B
IGSF6	ICI signature genes A
IKZF1	ICI signature genes A
IKZF3	ICI signature genes A
IL10	ICI signature genes B
IL10RA	ICI signature genes A
IL11	ICI signature genes B
IL12B	ICI signature genes B

IL12RB1	ICI signature genes A
IL13	ICI signature genes B
IL13RA2	ICI signature genes A
IL16	ICI signature genes A
IL17A	ICI signature genes B
IL17REL	ICI signature genes A
IL18RAP	ICI signature genes B
IL1A	ICI signature genes B
IL1RL1	ICI signature genes A
IL21	ICI signature genes B
IL21-AS1	ICI signature genes B
IL21R	ICI signature genes A
IL22RA2	ICI signature genes B
IL27	ICI signature genes B
IL2RA	ICI signature genes A
IL2RB	ICI signature genes A
IL2RG	ICI signature genes A
IL32	ICI signature genes A
IL36B	ICI signature genes B
IL36G	ICI signature genes B
IL36RN	ICI signature genes B
IL4I1	ICI signature genes A
IL5RA	ICI signature genes B
IL7R	ICI signature genes A
IL9R	ICI signature genes B
INHBA	ICI signature genes A
INHBA-AS1	ICI signature genes B
INMT	ICI signature genes A
INPP5D	ICI signature genes A
INSYN2B	ICI signature genes B
IQGAP2	ICI signature genes A
IQSEC3	ICI signature genes B
IRAK3	ICI signature genes A
IRF4	ICI signature genes A
IRF8	ICI signature genes A
IRS4	ICI signature genes B
ISLR	ICI signature genes A
ISM1	ICI signature genes A
ISM2	ICI signature genes B
ITGA11	ICI signature genes A
ITGA8	ICI signature genes A
ITGAD	ICI signature genes B
ITGAL	ICI signature genes A
ITGAM	ICI signature genes A
ITGAX	ICI signature genes A
ITGB2	ICI signature genes A
ITGB2-AS1	ICI signature genes A
ITGB3	ICI signature genes B
ITGB7	ICI signature genes A
ITGBL1	ICI signature genes A
ITIH3	ICI signature genes B
ITIH5	ICI signature genes A
ITK	ICI signature genes A
ITLN1	ICI signature genes B
ITM2A	ICI signature genes A
JAK3	ICI signature genes A
JAKMIP1	ICI signature genes B
JAML	ICI signature genes A

JCHAIN	ICI signature genes A
JPH2	ICI signature genes B
JSRP1	ICI signature genes A
KANK4	ICI signature genes B
KBTBD12	ICI signature genes B
KCNA1	ICI signature genes B
KCNA2	ICI signature genes B
KCNA3	ICI signature genes A
KCNA4	ICI signature genes B
KCNA5	ICI signature genes B
KCND2	ICI signature genes B
KCNE4	ICI signature genes A
KCNIP1	ICI signature genes B
KCNJ10	ICI signature genes B
KCNJ5	ICI signature genes A
KCNMB1	ICI signature genes B
KCNN3	ICI signature genes B
KCNT2	ICI signature genes B
KEL	ICI signature genes A
KERA	ICI signature genes B
KHDRBS2	ICI signature genes B
KIAA1549L	ICI signature genes B
KIAA1755	ICI signature genes B
KIF26B	ICI signature genes A
KIR2DL4	ICI signature genes B
KIR2DS4	ICI signature genes B
KIR3DL3	ICI signature genes B
KIR3DX1	ICI signature genes B
KLHDC7B	ICI signature genes A
KLHL6	ICI signature genes A
KLK8	ICI signature genes B
KLRB1	ICI signature genes A
KLRC1	ICI signature genes B
KLRC2	ICI signature genes B
KLRC4	ICI signature genes B
KLRC4-KLRK1	ICI signature genes B
KLRD1	ICI signature genes B
KLRG1	ICI signature genes B
KLRK1	ICI signature genes B
KMO	ICI signature genes B
KRT14	ICI signature genes B
KRT16P4	ICI signature genes B
KRT16P6	ICI signature genes B
KRT17	ICI signature genes B
KRT23	ICI signature genes B
KRT5	ICI signature genes B
KRT7	ICI signature genes A
KRT7-AS	ICI signature genes B
KRT75	ICI signature genes B
KRTAP19-8	ICI signature genes B
L1TD1	ICI signature genes B
L3MBTL4-AS1	ICI signature genes B
LAG3	ICI signature genes A
LAIR1	ICI signature genes A
LAIR2	ICI signature genes B
LAMA1	ICI signature genes B
LAMA2	ICI signature genes A
LAMA4	ICI signature genes A

LAMC2	ICI signature genes B
LAMP5	ICI signature genes A
LAPTM5	ICI signature genes A
LAT2	ICI signature genes A
LAX1	ICI signature genes A
LCK	ICI signature genes A
LCP1	ICI signature genes A
LCP2	ICI signature genes A
LGALS1	ICI signature genes A
LGALS2	ICI signature genes A
LGI2	ICI signature genes A
LGR5	ICI signature genes B
LHX8	ICI signature genes B
LILRA1	ICI signature genes B
LILRA2	ICI signature genes B
LILRA4	ICI signature genes B
LILRA5	ICI signature genes A
LILRA6	ICI signature genes A
LILRB1	ICI signature genes A
LILRB2	ICI signature genes A
LILRB3	ICI signature genes A
LILRB4	ICI signature genes A
LILRB5	ICI signature genes B
LILRP2	ICI signature genes B
LINC00051	ICI signature genes B
LINC00158	ICI signature genes B
LINC00221	ICI signature genes B
LINC002481	ICI signature genes B
LINC00299	ICI signature genes B
LINC00402	ICI signature genes B
LINC00426	ICI signature genes B
LINC00460	ICI signature genes B
LINC00525	ICI signature genes B
LINC00551	ICI signature genes B
LINC00582	ICI signature genes A
LINC00607	ICI signature genes B
LINC00626	ICI signature genes B
LINC00702	ICI signature genes B
LINC00861	ICI signature genes B
LINC00871	ICI signature genes B
LINC00877	ICI signature genes B
LINC00892	ICI signature genes B
LINC00898	ICI signature genes B
LINC00922	ICI signature genes B
LINC00937	ICI signature genes B
LINC00939	ICI signature genes B
LINC00944	ICI signature genes B
LINC00968	ICI signature genes B
LINC00996	ICI signature genes B
LINC01038	ICI signature genes B
LINC01094	ICI signature genes A
LINC01133	ICI signature genes B
LINC01146	ICI signature genes B
LINC01150	ICI signature genes B
LINC01194	ICI signature genes B
LINC01205	ICI signature genes B
LINC01215	ICI signature genes A
LINC01228	ICI signature genes B

LINC01231	ICI signature genes B
LINC01234	ICI signature genes B
LINC01235	ICI signature genes B
LINC01241	ICI signature genes B
LINC01281	ICI signature genes B
LINC01326	ICI signature genes B
LINC01358	ICI signature genes B
LINC01446	ICI signature genes B
LINC01480	ICI signature genes A
LINC01561	ICI signature genes B
LINC01564	ICI signature genes B
LINC01605	ICI signature genes B
LINC01614	ICI signature genes A
LINC01615	ICI signature genes B
LINC01628	ICI signature genes B
LINC01638	ICI signature genes B
LINC01665	ICI signature genes B
LINC01684	ICI signature genes B
LINC01697	ICI signature genes B
LINC01705	ICI signature genes B
LINC01711	ICI signature genes B
LINC01776	ICI signature genes B
LINC01781	ICI signature genes A
LINC01853	ICI signature genes B
LINC01857	ICI signature genes A
LINC01871	ICI signature genes A
LINC01886	ICI signature genes B
LINC01914	ICI signature genes B
LINC01915	ICI signature genes B
LINC01925	ICI signature genes B
LINC01929	ICI signature genes B
LINC01934	ICI signature genes B
LINC01936	ICI signature genes B
LINC01937	ICI signature genes B
LINC01943	ICI signature genes B
LINC01996	ICI signature genes B
LINC01997	ICI signature genes B
LINC02016	ICI signature genes B
LINC02057	ICI signature genes B
LINC02063	ICI signature genes B
LINC02084	ICI signature genes B
LINC02099	ICI signature genes B
LINC02114	ICI signature genes B
LINC02126	ICI signature genes B
LINC02195	ICI signature genes A
LINC02257	ICI signature genes B
LINC02273	ICI signature genes B
LINC02285	ICI signature genes B
LINC02303	ICI signature genes B
LINC02327	ICI signature genes B
LINC02345	ICI signature genes B
LINC02362	ICI signature genes A
LINC02384	ICI signature genes A
LINC02397	ICI signature genes B
LINC02408	ICI signature genes B
LINC02422	ICI signature genes B
LINC02423	ICI signature genes B
LINC02446	ICI signature genes A

LINC02500	ICI signature genes B
LINC02525	ICI signature genes B
LINC02528	ICI signature genes B
LINC02544	ICI signature genes B
LINC02576	ICI signature genes A
LINC02588	ICI signature genes B
LINC02611	ICI signature genes B
LINC02619	ICI signature genes B
LINC02694	ICI signature genes B
LINC02699	ICI signature genes B
LINC02778	ICI signature genes B
LIPC	ICI signature genes B
LMO3	ICI signature genes A
LMOD1	ICI signature genes A
LNCOG	ICI signature genes B
LOXL2	ICI signature genes A
LPAR4	ICI signature genes B
LPL	ICI signature genes A
LPXN	ICI signature genes A
LRRC15	ICI signature genes A
LRRC17	ICI signature genes A
LRRC2	ICI signature genes B
LRRC25	ICI signature genes A
LRRC32	ICI signature genes A
LRRC4C	ICI signature genes B
LRRC55	ICI signature genes B
LRRK2	ICI signature genes A
LRRK2-DT	ICI signature genes A
LRRN4	ICI signature genes A
LSAMP	ICI signature genes A
LSP1	ICI signature genes A
LST1	ICI signature genes A
LTA	ICI signature genes A
LTB	ICI signature genes A
LTF	ICI signature genes A
LUM	ICI signature genes A
LY6H	ICI signature genes B
LY86	ICI signature genes A
LY9	ICI signature genes A
LY96	ICI signature genes A
LYVE1	ICI signature genes A
LYZ	ICI signature genes A
M1AP	ICI signature genes B
MAGEA6	ICI signature genes B
MAGEL2	ICI signature genes B
MAGI2-AS3	ICI signature genes B
MAL	ICI signature genes A
MAMDC2	ICI signature genes A
MAN1A1	ICI signature genes A
MAP1LC3C	ICI signature genes B
MAP4K1	ICI signature genes A
MARCO	ICI signature genes A
MATN3	ICI signature genes A
MCCC1-AS1	ICI signature genes B
MCEMP1	ICI signature genes A
MCHR1	ICI signature genes B
MEDAG	ICI signature genes A
MEF2C	ICI signature genes A

MEF2C-AS1	ICI signature genes B
MEFV	ICI signature genes B
MEI1	ICI signature genes A
MEOX2	ICI signature genes A
MFAP4	ICI signature genes A
MFAP5	ICI signature genes B
MGP	ICI signature genes A
MICAL2	ICI signature genes A
MILR1	ICI signature genes A
MIR100HG	ICI signature genes B
MIR155HG	ICI signature genes A
MIR3142HG	ICI signature genes B
MIR3945HG	ICI signature genes B
MIR4314	ICI signature genes B
MIR4537	ICI signature genes A
MIR4539	ICI signature genes A
MIR670HG	ICI signature genes B
MIR8071-2	ICI signature genes A
MLLT10P2	ICI signature genes B
MME	ICI signature genes B
MMP1	ICI signature genes B
MMP10	ICI signature genes B
MMP11	ICI signature genes A
MMP12	ICI signature genes A
MMP13	ICI signature genes A
MMP16	ICI signature genes B
MMP19	ICI signature genes A
MMP2	ICI signature genes A
MMP2-AS1	ICI signature genes B
MMP28	ICI signature genes B
MMP3	ICI signature genes B
MMP7	ICI signature genes A
MMP8	ICI signature genes B
MMP9	ICI signature genes A
MMRN1	ICI signature genes A
MNDA	ICI signature genes A
MOXD1	ICI signature genes A
MPEG1	ICI signature genes A
MPPED1	ICI signature genes B
MRC1	ICI signature genes A
MRGPRF	ICI signature genes A
MRGPRG	ICI signature genes B
MRPL50P1	ICI signature genes B
MRPS10P1	ICI signature genes B
MRVI1	ICI signature genes A
MS4A1	ICI signature genes A
MS4A14	ICI signature genes B
MS4A15	ICI signature genes A
MS4A2	ICI signature genes B
MS4A4A	ICI signature genes A
MS4A6A	ICI signature genes A
MS4A7	ICI signature genes A
MSLN	ICI signature genes A
MSR1	ICI signature genes A
MSRB3	ICI signature genes A
MT1M	ICI signature genes A
MUCL3	ICI signature genes B
MXRA5	ICI signature genes A

MXRA5Y	ICI signature genes B
MXRA8	ICI signature genes A
MYBPC2	ICI signature genes B
MYL3	ICI signature genes B
MYO1F	ICI signature genes A
MYO1G	ICI signature genes A
MYOCD	ICI signature genes B
MZB1	ICI signature genes A
NAIP	ICI signature genes B
NAIPP1	ICI signature genes B
NALCN	ICI signature genes B
NAPSA	ICI signature genes A
NAPSB	ICI signature genes A
NAV3	ICI signature genes B
NCAM2	ICI signature genes B
NCF1	ICI signature genes A
NCF1B	ICI signature genes A
NCF1C	ICI signature genes A
NCF4	ICI signature genes A
NCKAP1L	ICI signature genes A
NCR1	ICI signature genes B
NCR3	ICI signature genes A
NDNF	ICI signature genes A
NECAB2	ICI signature genes B
NEFL	ICI signature genes B
NEGR1	ICI signature genes B
NELL1	ICI signature genes B
NETO1	ICI signature genes B
NEXN	ICI signature genes A
NF1P8	ICI signature genes B
NFAM1	ICI signature genes A
NFATC2	ICI signature genes A
NIBAN3	ICI signature genes A
NID2	ICI signature genes A
NKAIN2	ICI signature genes B
NKG7	ICI signature genes A
NKX2-1	ICI signature genes A
NKX3-2	ICI signature genes B
NLRC3	ICI signature genes A
NLRP12	ICI signature genes B
NLRP3	ICI signature genes A
NLRP7	ICI signature genes B
NMRAL2P	ICI signature genes B
NNMT	ICI signature genes A
NOX4	ICI signature genes B
NPFFR1	ICI signature genes B
NR0B1	ICI signature genes B
NR4A3	ICI signature genes A
NRIR	ICI signature genes B
NT5E	ICI signature genes B
NTM	ICI signature genes A
NTNG1	ICI signature genes B
NTRK3	ICI signature genes B
NTS	ICI signature genes B
NUGGC	ICI signature genes A
ODAM	ICI signature genes B
OGN	ICI signature genes A
OLFML1	ICI signature genes A

OLFML2B	ICI signature genes A
OLFML3	ICI signature genes A
OLR1	ICI signature genes A
OMD	ICI signature genes A
OR10AE1P	ICI signature genes B
OR1E3	ICI signature genes B
OR2C3	ICI signature genes B
OR2I1P	ICI signature genes A
OR4C14P	ICI signature genes B
OR4K13	ICI signature genes B
OR6C66P	ICI signature genes B
OR7E22P	ICI signature genes B
OR7E47P	ICI signature genes B
ORM1	ICI signature genes A
ORM2	ICI signature genes A
OSCAR	ICI signature genes A
OSGIN1	ICI signature genes B
OSM	ICI signature genes A
OTOP1	ICI signature genes B
OTOP2	ICI signature genes B
OTOP3	ICI signature genes B
P2RX1	ICI signature genes A
P2RX5	ICI signature genes A
P2RX7	ICI signature genes A
P2RY10	ICI signature genes A
P2RY12	ICI signature genes B
P2RY13	ICI signature genes A
P2RY14	ICI signature genes A
P2RY8	ICI signature genes A
P4HA3	ICI signature genes B
PABPC5	ICI signature genes B
PADI2	ICI signature genes A
PAEP	ICI signature genes B
PAK3	ICI signature genes B
PALM2AKAP2	ICI signature genes B
PANX3	ICI signature genes B
PAPPA	ICI signature genes B
PARP15	ICI signature genes A
PART1	ICI signature genes B
PARVG	ICI signature genes A
PATL2	ICI signature genes B
PAX5	ICI signature genes A
PCAT29	ICI signature genes B
PCDHGA12	ICI signature genes B
PCDHGA9	ICI signature genes B
PCED1B-AS1	ICI signature genes A
PCOLCE	ICI signature genes A
PDCD1	ICI signature genes A
PDCD1LG2	ICI signature genes A
PDE1A	ICI signature genes B
PDE4B	ICI signature genes A
PDE6G	ICI signature genes A
PDGFRA	ICI signature genes A
PDGFRB	ICI signature genes A
PDGFRL	ICI signature genes A
PDK4	ICI signature genes A
PDLIM3	ICI signature genes A
PDPN	ICI signature genes A

PDZRN3	ICI signature genes A
PDZRN4	ICI signature genes B
PEAK3	ICI signature genes B
PEBP4	ICI signature genes A
PECAM1	ICI signature genes A
PEG3	ICI signature genes B
PGC	ICI signature genes A
PGM5	ICI signature genes B
PGR	ICI signature genes B
PIANP	ICI signature genes B
PIEZO2	ICI signature genes B
PIGR	ICI signature genes A
PIK3AP1	ICI signature genes A
PIK3CG	ICI signature genes A
PIK3R5	ICI signature genes A
PIK3R6	ICI signature genes A
PILRA	ICI signature genes A
PIM2	ICI signature genes A
PKD2L1	ICI signature genes B
PKHD1L1	ICI signature genes B
PLA1A	ICI signature genes A
PLA2G12B	ICI signature genes B
PLA2G1B	ICI signature genes A
PLA2G2A	ICI signature genes A
PLA2G2D	ICI signature genes A
PLA2G5	ICI signature genes B
PLA2G7	ICI signature genes A
PLAAT4	ICI signature genes A
PLAC8	ICI signature genes A
PLCL2	ICI signature genes A
PLCXD3	ICI signature genes B
PLD4	ICI signature genes B
PLEK	ICI signature genes A
PLN	ICI signature genes A
PLPP4	ICI signature genes B
PLPP7	ICI signature genes B
PLXNA4	ICI signature genes B
PLXNC1	ICI signature genes A
PMEPA1	ICI signature genes A
PMP22	ICI signature genes A
PNCK	ICI signature genes B
PNMA2	ICI signature genes B
PNMA3	ICI signature genes B
PNMA8A	ICI signature genes B
PNOC	ICI signature genes A
PODN	ICI signature genes A
PODNL1	ICI signature genes A
POSTN	ICI signature genes A
POU2F2	ICI signature genes A
PPFIA2	ICI signature genes B
PPIAP39	ICI signature genes B
PPP1R16B	ICI signature genes A
PPP1R1AP1	ICI signature genes B
PPP1R9A	ICI signature genes B
PQLC1P1	ICI signature genes B
PRAC2	ICI signature genes B
PRAME	ICI signature genes B
PRAMEF13	ICI signature genes B

PRELP	ICI signature genes A
PRF1	ICI signature genes A
PRG4	ICI signature genes B
PRKCB	ICI signature genes A
PRND	ICI signature genes B
PROKR2	ICI signature genes B
PRRX1	ICI signature genes A
PSMB9	ICI signature genes A
PTAFR	ICI signature genes A
PTCRA	ICI signature genes B
PTF1A	ICI signature genes B
PTGDR	ICI signature genes B
PTGDS	ICI signature genes A
PTGER2	ICI signature genes A
PTGER3	ICI signature genes B
PTGES3P2	ICI signature genes B
PTGFR	ICI signature genes B
PTGIS	ICI signature genes A
PTHLH	ICI signature genes B
PTPN22	ICI signature genes A
PTPN7	ICI signature genes A
PTPRC	ICI signature genes A
PTPRN	ICI signature genes B
PTX3	ICI signature genes B
PXDN	ICI signature genes B
PYHIN1	ICI signature genes A
RAB37	ICI signature genes A
RANBP3L	ICI signature genes B
RARRES2	ICI signature genes A
RARRES2P8	ICI signature genes B
RASAL3	ICI signature genes A
RASGRF1	ICI signature genes B
RASGRF2	ICI signature genes B
RASGRP4	ICI signature genes B
RASSF2	ICI signature genes A
RASSF4	ICI signature genes A
RBMY1A1	ICI signature genes B
RBMY3AP	ICI signature genes B
RCAN2	ICI signature genes A
RCN3	ICI signature genes A
RCSD1	ICI signature genes A
REM1	ICI signature genes B
RETN	ICI signature genes A
RFLNA	ICI signature genes A
RFTN1	ICI signature genes A
RGS1	ICI signature genes A
RGS13	ICI signature genes B
RGS18	ICI signature genes A
RGS4	ICI signature genes B
RHEX	ICI signature genes B
RHOH	ICI signature genes A
RIPOR2	ICI signature genes A
RN7SKP229	ICI signature genes B
RN7SKP231	ICI signature genes B
RN7SKP48	ICI signature genes B
RN7SL132P	ICI signature genes B
RN7SL617P	ICI signature genes B
RNA5-8SN3	ICI signature genes B

RNA5-8SP4	ICI signature genes B
RNA5SP263	ICI signature genes B
RNASE1	ICI signature genes A
RNASE2	ICI signature genes A
RNASE6	ICI signature genes A
RNASE7	ICI signature genes B
RNU1-34P	ICI signature genes B
RNU4ATAC6P	ICI signature genes B
RNU6-189P	ICI signature genes B
RNU6-562P	ICI signature genes B
RNU6-579P	ICI signature genes B
ROR1	ICI signature genes B
RORC	ICI signature genes A
ROS1	ICI signature genes A
RP1-309I22.2	ICI signature genes B
RP11-345J18.2	ICI signature genes B
RP11-366M4.11	ICI signature genes B
RP11-367G6.3	ICI signature genes B
RP11-401P9.4	ICI signature genes B
RP11-445P19.3	ICI signature genes B
RP11-556E13.1	ICI signature genes B
RP11-652L8.4	ICI signature genes B
RP11-706O15.3	ICI signature genes B
RP3-492J12.2	ICI signature genes B
RP4-669B10.3	ICI signature genes B
RPL31P41	ICI signature genes B
RSAD2	ICI signature genes A
RSPO1	ICI signature genes B
RSPO3	ICI signature genes A
RTL3	ICI signature genes B
RTN1	ICI signature genes B
RTP5	ICI signature genes B
RUBCNL	ICI signature genes A
RUFY4	ICI signature genes B
RUNX1T1	ICI signature genes B
S100A2	ICI signature genes B
S100B	ICI signature genes A
S1PR1	ICI signature genes A
S1PR4	ICI signature genes A
SAA1	ICI signature genes A
SAA2	ICI signature genes A
SAA2-SAA4	ICI signature genes A
SAGE1	ICI signature genes B
SAMD3	ICI signature genes B
SAMD9L	ICI signature genes A
SAMSN1	ICI signature genes A
SAPCD1	ICI signature genes B
SASH3	ICI signature genes A
SBSN	ICI signature genes B
SCARA5	ICI signature genes B
SCARF2	ICI signature genes A
SCGB3A1	ICI signature genes A
SCGB3A2	ICI signature genes A
SCIMP	ICI signature genes A
SCML4	ICI signature genes B
SCN1A	ICI signature genes B
SCN2B	ICI signature genes B
SCN7A	ICI signature genes A

SCTR	ICI signature genes A
SELE	ICI signature genes A
SELL	ICI signature genes A
SELP	ICI signature genes A
SELPLG	ICI signature genes A
SEMA3C	ICI signature genes B
SEMA5A	ICI signature genes B
SENCR	ICI signature genes B
SEPTIN1	ICI signature genes A
SERPINA1	ICI signature genes A
SERPING1	ICI signature genes A
SERTM2	ICI signature genes B
SFRP2	ICI signature genes A
SFRP4	ICI signature genes A
SFTA2	ICI signature genes A
SFTA3	ICI signature genes A
SFTPA1	ICI signature genes A
SFTPA2	ICI signature genes A
SFTPA3P	ICI signature genes B
SFTPB	ICI signature genes A
SFTPC	ICI signature genes A
SFTPD	ICI signature genes A
SGCA	ICI signature genes A
SGCD	ICI signature genes B
SGIP1	ICI signature genes B
SH2D1A	ICI signature genes A
SH2D1B	ICI signature genes B
SH2D5	ICI signature genes B
SHANK1	ICI signature genes B
SHISA2	ICI signature genes B
SHISA9	ICI signature genes B
SHISAL1	ICI signature genes B
SIGLEC1	ICI signature genes A
SIGLEC10	ICI signature genes A
SIGLEC11	ICI signature genes B
SIGLEC12	ICI signature genes A
SIGLEC14	ICI signature genes A
SIGLEC22P	ICI signature genes B
SIGLEC5	ICI signature genes B
SIGLEC6	ICI signature genes B
SIGLEC7	ICI signature genes A
SIGLEC8	ICI signature genes A
SIGLEC9	ICI signature genes A
SIRPB1	ICI signature genes A
SIRPB2	ICI signature genes A
SIRPG	ICI signature genes A
SIRPG-AS1	ICI signature genes B
SIT1	ICI signature genes A
SKAP1	ICI signature genes A
SLA	ICI signature genes A
SLA2	ICI signature genes A
SLAMF1	ICI signature genes A
SLAMF6	ICI signature genes A
SLAMF7	ICI signature genes A
SLAMF8	ICI signature genes A
SLC10A6	ICI signature genes B
SLC12A3	ICI signature genes B
SLC13A5	ICI signature genes B

SLC15A3	ICI signature genes A
SLC16A4	ICI signature genes A
SLC18A3	ICI signature genes B
SLC1A7	ICI signature genes B
SLC22A3	ICI signature genes A
SLC22A31	ICI signature genes A
SLC24A2	ICI signature genes B
SLC26A4-AS1	ICI signature genes B
SLC34A2	ICI signature genes A
SLC46A2	ICI signature genes B
SLC47A2	ICI signature genes B
SLC5A9	ICI signature genes B
SLC6A2	ICI signature genes B
SLC7A10	ICI signature genes B
SLC7A7	ICI signature genes A
SLCO2A1	ICI signature genes A
SLCO2B1	ICI signature genes A
SLCO5A1	ICI signature genes B
SLFN12L	ICI signature genes B
SLIT3	ICI signature genes A
SMARCE1P7	ICI signature genes B
SMIM25	ICI signature genes A
SNED1	ICI signature genes A
SNX20	ICI signature genes A
SOCAR	ICI signature genes B
SOD3	ICI signature genes A
SORCS2	ICI signature genes B
SOX11	ICI signature genes B
SOX2	ICI signature genes B
SOX2-OT	ICI signature genes B
SOX21	ICI signature genes B
SP140	ICI signature genes A
SPAG6	ICI signature genes A
SPANXA1	ICI signature genes B
SPARC	ICI signature genes A
SPATA20P1	ICI signature genes A
SPATA31C1	ICI signature genes B
SPATA8-AS1	ICI signature genes B
SPI1	ICI signature genes A
SPIB	ICI signature genes A
SPN	ICI signature genes A
SPNS3	ICI signature genes B
SPOCK1	ICI signature genes B
SPOCK2	ICI signature genes A
SPON1	ICI signature genes A
SPON2	ICI signature genes A
SRGN	ICI signature genes A
SSC5D	ICI signature genes A
SSPN	ICI signature genes A
SST	ICI signature genes B
SSTR1	ICI signature genes B
SSTR2	ICI signature genes B
SSTR3	ICI signature genes B
SSU72P3	ICI signature genes B
ST6GALNAC5	ICI signature genes A
ST8SIA2	ICI signature genes B
STAB1	ICI signature genes A
STAP1	ICI signature genes A

STARD13	ICI signature genes A
STARD8	ICI signature genes A
STAT4	ICI signature genes A
STXBP5L	ICI signature genes B
SUCNR1	ICI signature genes A
SUGCT	ICI signature genes B
SULF1	ICI signature genes A
SULT1C2	ICI signature genes B
SUSD3	ICI signature genes A
SUSD5	ICI signature genes B
SYNDIG1	ICI signature genes B
SYNE1	ICI signature genes A
SYNPO2	ICI signature genes A
SYT15	ICI signature genes B
TAFA5	ICI signature genes B
TAGAP	ICI signature genes A
TAGLN	ICI signature genes A
TBC1D10C	ICI signature genes A
TBC1D27P	ICI signature genes B
TBX21	ICI signature genes A
TBX5-AS1	ICI signature genes B
TBXAS1	ICI signature genes A
TCF21	ICI signature genes A
TCL1A	ICI signature genes A
TDRD6	ICI signature genes B
TENM2	ICI signature genes B
TENM3	ICI signature genes B
TENM3-AS1	ICI signature genes B
TENT5C	ICI signature genes A
TESPA1	ICI signature genes A
TFAP2D	ICI signature genes B
TFEC	ICI signature genes A
TGFB3	ICI signature genes A
TGFBI	ICI signature genes A
TGM2	ICI signature genes A
THBS1	ICI signature genes A
THBS2	ICI signature genes A
THEMIS	ICI signature genes B
THEMIS2	ICI signature genes A
THY1	ICI signature genes A
TIFAB	ICI signature genes B
TIGIT	ICI signature genes A
TIMD4	ICI signature genes A
TIMP2	ICI signature genes A
TIMP3	ICI signature genes A
TLR10	ICI signature genes A
TLR4	ICI signature genes A
TLR7	ICI signature genes A
TLR8	ICI signature genes A
TLX2	ICI signature genes B
TM6SF1	ICI signature genes A
TMEM100	ICI signature genes A
TMEM119	ICI signature genes A
TMEM130	ICI signature genes A
TMEM132D	ICI signature genes B
TMEM132D-AS1	ICI signature genes B
TMEM150B	ICI signature genes A
TMEM156	ICI signature genes A

TMEM158	ICI signature genes B
TMEM163	ICI signature genes B
TMEM176A	ICI signature genes A
TMEM176B	ICI signature genes A
TMEM200A	ICI signature genes A
TMEM212	ICI signature genes B
TMEM229B	ICI signature genes B
TMEM236	ICI signature genes B
TMEM271	ICI signature genes B
TMEM273	ICI signature genes A
TMEM52B	ICI signature genes B
TMIGD2	ICI signature genes B
TMIGD3	ICI signature genes A
TMPRSS11D	ICI signature genes B
TMPRSS2	ICI signature genes A
TNC	ICI signature genes B
TNFAIP6	ICI signature genes A
TNFAIP8L2	ICI signature genes A
TNFRSF13B	ICI signature genes A
TNFRSF17	ICI signature genes A
TNFRSF1B	ICI signature genes A
TNFRSF8	ICI signature genes B
TNFRSF9	ICI signature genes A
TNFSF11	ICI signature genes B
TNFSF13B	ICI signature genes A
TNFSF14	ICI signature genes B
TNFSF4	ICI signature genes A
TNFSF8	ICI signature genes A
TNIP3	ICI signature genes A
TNN	ICI signature genes B
TNNC1	ICI signature genes A
TNR	ICI signature genes B
TNR-IT1	ICI signature genes B
TNS3	ICI signature genes A
TOMM20P2	ICI signature genes B
TPTE	ICI signature genes B
TRAC	ICI signature genes A
TRAF3IP3	ICI signature genes A
TRAT1	ICI signature genes A
TRAV1-2	ICI signature genes B
TRAV10	ICI signature genes B
TRAV12-1	ICI signature genes A
TRAV12-2	ICI signature genes A
TRAV12-3	ICI signature genes A
TRAV13-1	ICI signature genes A
TRAV13-2	ICI signature genes B
TRAV14DV4	ICI signature genes B
TRAV16	ICI signature genes B
TRAV17	ICI signature genes B
TRAV19	ICI signature genes A
TRAV2	ICI signature genes A
TRAV20	ICI signature genes B
TRAV21	ICI signature genes A
TRAV22	ICI signature genes B
TRAV23DV6	ICI signature genes B
TRAV24	ICI signature genes B
TRAV25	ICI signature genes B
TRAV26-1	ICI signature genes B

TRAV26-2	ICI signature genes B
TRAV27	ICI signature genes B
TRAV29DV5	ICI signature genes B
TRAV3	ICI signature genes B
TRAV35	ICI signature genes B
TRAV36DV7	ICI signature genes B
TRAV39	ICI signature genes B
TRAV4	ICI signature genes A
TRAV41	ICI signature genes B
TRAV5	ICI signature genes B
TRAV6	ICI signature genes B
TRAV8-1	ICI signature genes B
TRAV8-2	ICI signature genes B
TRAV8-3	ICI signature genes A
TRAV8-4	ICI signature genes B
TRAV8-6	ICI signature genes A
TRAV9-2	ICI signature genes A
TRBC1	ICI signature genes A
TRBC2	ICI signature genes A
TRBJ2-7	ICI signature genes A
TRBV10-3	ICI signature genes A
TRBV11-2	ICI signature genes A
TRBV12-3	ICI signature genes B
TRBV12-4	ICI signature genes A
TRBV13	ICI signature genes B
TRBV14	ICI signature genes B
TRBV15	ICI signature genes B
TRBV18	ICI signature genes A
TRBV19	ICI signature genes A
TRBV2	ICI signature genes A
TRBV20-1	ICI signature genes A
TRBV23-1	ICI signature genes B
TRBV24-1	ICI signature genes B
TRBV27	ICI signature genes B
TRBV28	ICI signature genes A
TRBV29-1	ICI signature genes A
TRBV3-1	ICI signature genes A
TRBV30	ICI signature genes A
TRBV4-1	ICI signature genes A
TRBV4-2	ICI signature genes A
TRBV5-1	ICI signature genes A
TRBV5-4	ICI signature genes A
TRBV5-6	ICI signature genes A
TRBV6-1	ICI signature genes A
TRBV6-5	ICI signature genes A
TRBV6-6	ICI signature genes A
TRBV7-3	ICI signature genes B
TRBV7-4	ICI signature genes B
TRBV7-6	ICI signature genes B
TRBV7-9	ICI signature genes A
TRBV9	ICI signature genes A
TRDC	ICI signature genes A
TRDV1	ICI signature genes B
TREM1	ICI signature genes A
TREM2	ICI signature genes A
TREML1	ICI signature genes B
TRG-AS1	ICI signature genes B
TRGC1	ICI signature genes B

TRGC2	ICI signature genes A
TRGV10	ICI signature genes A
TRGV3	ICI signature genes B
TRGV4	ICI signature genes B
TRGV5	ICI signature genes B
TRGV7	ICI signature genes B
TRIM55	ICI signature genes B
TRIM61	ICI signature genes B
TRIML2	ICI signature genes B
TRPC7-AS2	ICI signature genes B
TRPV2	ICI signature genes A
TSHR	ICI signature genes B
TSHZ3	ICI signature genes B
TSPAN11	ICI signature genes A
TSPAN2	ICI signature genes A
TSPAN32	ICI signature genes B
TSPAN4	ICI signature genes A
TSPY2	ICI signature genes B
TTC24	ICI signature genes B
TUSC7	ICI signature genes B
TVP23A	ICI signature genes B
TWIST1	ICI signature genes B
TXK	ICI signature genes B
TYROBP	ICI signature genes A
UBASH3A	ICI signature genes A
UBD	ICI signature genes A
UBE2QL1	ICI signature genes B
UGT1A6	ICI signature genes B
UGT1A7	ICI signature genes B
UNC5A	ICI signature genes B
UNC5C	ICI signature genes B
USH1G	ICI signature genes B
USP30-AS1	ICI signature genes A
VCAM1	ICI signature genes A
VCAN	ICI signature genes A
VEGFD	ICI signature genes A
VENTX	ICI signature genes B
VENTXP5	ICI signature genes B
VGLL3	ICI signature genes A
VIM	ICI signature genes A
VNN1	ICI signature genes A
VNN2	ICI signature genes A
VSIG4	ICI signature genes A
VSTM1	ICI signature genes B
VSTM2L	ICI signature genes A
VSTM4	ICI signature genes A
WAS	ICI signature genes A
WDFY4	ICI signature genes A
WFDC5	ICI signature genes B
WIPF1	ICI signature genes A
WNT2	ICI signature genes A
XAGE2	ICI signature genes B
XCL2	ICI signature genes A
XCR1	ICI signature genes B
XIRP1	ICI signature genes B
XIST	ICI signature genes A
XKR4	ICI signature genes B
XKR7	ICI signature genes B

XPNPEP2	ICI signature genes B
XXYLT1-AS2	ICI signature genes B
Z95114.3	ICI signature genes B
ZAP70	ICI signature genes A
ZBP1	ICI signature genes A
ZBTB16	ICI signature genes B
ZC3H12D	ICI signature genes A
ZEB2	ICI signature genes A
ZEB2-AS1	ICI signature genes B
ZFHX4	ICI signature genes B
ZFP57	ICI signature genes B
ZIC3	ICI signature genes B
ZNF366	ICI signature genes B
ZNF385D	ICI signature genes B
ZNF423	ICI signature genes B
ZNF469	ICI signature genes B
ZNF521	ICI signature genes A
ZNF683	ICI signature genes A
ZNF734P	ICI signature genes B
ZNF80	ICI signature genes B
ZNF804A	ICI signature genes B
ZNF831	ICI signature genes B
ZPLD1	ICI signature genes B
ZYG11A	ICI signature genes B

Table-S6-ICI score for each individual

Tag	ICI score	ICI score group accroding to X-tile
TCGA-34-5234	1.544043521	High
TCGA-96-7544	-0.931571569	Low
TCGA-85-A513	-1.137877033	Low
TCGA-85-8584	1.316153426	High
TCGA-85-A511	-0.079421555	High
TCGA-66-2789	0.485929559	High
TCGA-22-0940	0.484505843	High
TCGA-96-A4JL	1.627464558	High
TCGA-77-8136	-0.464589508	High
TCGA-63-5131	0.851938446	High
TCGA-90-7964	-0.051154649	High
TCGA-85-A5B5	0.04297421	High
TCGA-37-3789	0.465675467	High
TCGA-O2-A5IB	1.647481702	High
TCGA-58-8390	0.742565671	High
TCGA-66-2777	-0.819016086	Low
TCGA-77-8140	0.330324299	High
TCGA-98-A538	-0.723476552	High
TCGA-66-2785	-0.161925757	High
TCGA-43-5670	0.545836861	High
TCGA-22-5472	0.355308529	High
TCGA-60-2704	-1.581584553	Low
TCGA-77-6843	0.247138069	High
TCGA-63-A5MG	-2.181351953	Low
TCGA-66-2734	-0.331864926	High
TCGA-43-2581	-2.217745057	Low
TCGA-52-7811	-0.446056682	High
TCGA-85-8287	-1.835688604	Low
TCGA-85-8355	0.993076602	High
TCGA-94-7033	0.602852833	High
TCGA-60-2697	-0.715056912	High
TCGA-77-8145	0.010006072	High
TCGA-85-7844	0.039917284	High
TCGA-39-5040	1.952090452	High
TCGA-66-2795	-0.852783926	Low
TCGA-33-4586	-0.423311789	High
TCGA-NC-A5HH	1.395530474	High
TCGA-22-1002	1.393429497	High
TCGA-60-2695	0.631130868	High
TCGA-77-A5G8	-0.641667067	High
TCGA-63-A5MP	-0.163034333	High
TCGA-22-0944	-0.171721731	High
TCGA-21-1081	0.181061104	High
TCGA-O2-A52V	-0.156287712	High
TCGA-33-AASB	0.800106986	High
TCGA-63-5128	-0.476911396	High
TCGA-96-8170	0.345447462	High
TCGA-63-A5MW	2.865572858	High
TCGA-37-3792	-0.575116741	High
TCGA-56-A4BX	-0.643615875	High
TCGA-56-8083	3.774137741	High
TCGA-98-A53H	2.84831054	High
TCGA-94-A4VJ	0.395689758	High
TCGA-43-7658	2.494275908	High
TCGA-NC-A5HN	-0.749283297	High
TCGA-63-7021	-1.108660243	Low

TCGA-22-5471	0.51191592	High
TCGA-77-8133	0.412573179	High
TCGA-37-A5EM	0.488107881	High
TCGA-85-8071	0.893727947	High
TCGA-NC-A5HP	0.811178257	High
TCGA-85-A4PA	4.534305926	High
TCGA-33-4582	0.279693695	High
TCGA-43-2578	0.264936655	High
TCGA-34-A5IX	0.517666704	High
TCGA-56-8504	-0.061708362	High
TCGA-18-5595	-0.001255899	High
TCGA-21-5784	-1.001546002	Low
TCGA-90-A4ED	-0.604666084	High
TCGA-MF-A522	-0.295620001	High
TCGA-46-3768	-0.616179367	High
TCGA-77-7335	-1.569075169	Low
TCGA-56-A4BY	0.512212898	High
TCGA-85-8070	-0.526043795	High
TCGA-56-7579	-0.54224447	High
TCGA-NC-A5HG	0.184387639	High
TCGA-56-8305	-0.951684134	Low
TCGA-60-2703	0.320327172	High
TCGA-39-5036	-0.50071705	High
TCGA-33-6737	0.200446804	High
TCGA-34-2608	-1.758950121	Low
TCGA-39-5024	0.357406058	High
TCGA-77-7142	0.80456417	High
TCGA-85-8350	1.121818482	High
TCGA-O2-A52Q	-0.205737	High
TCGA-68-8250	-0.202623224	High
TCGA-70-6723	0.198139195	High
TCGA-68-8251	1.051746634	High
TCGA-85-7697	-0.271425114	High
TCGA-66-2756	1.456382858	High
TCGA-85-6561	-3.133967867	Low
TCGA-94-7943	0.88289153	High
TCGA-22-1011	-3.449207954	Low
TCGA-85-8664	0.69447279	High
TCGA-63-A5MH	1.547103905	High
TCGA-94-8490	-0.345915775	High
TCGA-85-8481	-0.28016173	High
TCGA-60-2716	0.534584735	High
TCGA-O2-A52S	-0.423819252	High
TCGA-51-4080	-0.223563203	High
TCGA-22-5485	-0.87820596	Low
TCGA-85-6175	-0.313420182	High
TCGA-85-8276	-2.208041711	Low
TCGA-77-8008	-1.180111757	Low
TCGA-39-5028	-0.870954656	Low
TCGA-34-5928	-1.49E-01	High
TCGA-43-6773	-7.06142189	Low
TCGA-63-A5MI	0.599783792	High
TCGA-63-A5ML	0.202789688	High
TCGA-39-5030	-4.451494771	Low
TCGA-51-4079	-1.707616381	Low
TCGA-22-5491	-0.057444171	High
TCGA-66-2744	-0.953021985	Low
TCGA-33-4547	0.069553625	High

TCGA-52-7810	0.165922	High
TCGA-77-7139	-0.743925967	High
TCGA-66-2800	-1.142194708	Low
TCGA-33-4566	-0.756860254	High
TCGA-90-7766	-1.200490539	Low
TCGA-60-2707	-0.465204079	High
TCGA-56-8628	0.893008271	High
TCGA-85-8666	-0.453727622	High
TCGA-60-2721	0.508355134	High
TCGA-22-4596	0.444849644	High
TCGA-33-A5GW	-0.04477713	High
TCGA-60-2715	2.095849313	High
TCGA-85-8351	0.591025805	High
TCGA-58-A46M	-0.363012296	High
TCGA-37-4129	5.225163183	High
TCGA-21-1075	-0.478891042	High
TCGA-43-7656	0.711014153	High
TCGA-58-8388	2.22083007	High
TCGA-98-A53J	0.095098362	High
TCGA-85-7696	0.584426133	High
TCGA-34-5231	0.293434624	High
TCGA-34-5929	0.253729223	High
TCGA-22-5492	-0.896474997	Low
TCGA-98-A53C	3.820896174	High
TCGA-94-8491	0.691662813	High
TCGA-90-7767	1.348767852	High
TCGA-96-8169	-0.560880405	High
TCGA-34-2596	0.168756683	High
TCGA-56-A62T	-0.123939082	High
TCGA-21-1071	0.146001229	High
TCGA-22-5480	1.071792622	High
TCGA-77-A5GH	0.49124625	High
TCGA-L3-A4E7	0.544159457	High
TCGA-60-2723	-1.373418694	Low
TCGA-37-3783	0.844180038	High
TCGA-66-2763	-0.206367254	High
TCGA-63-6202	-0.630445055	High
TCGA-L3-A524	-0.322239698	High
TCGA-98-8021	0.413482162	High
TCGA-77-A5GF	-0.80902599	Low
TCGA-66-2787	-0.243183868	High
TCGA-77-8138	-0.466481713	High
TCGA-63-A5MR	0.035700693	High
TCGA-66-2768	0.336268293	High
TCGA-77-8146	-0.185015007	High
TCGA-43-A56V	-0.098230377	High
TCGA-22-1005	-5.014943001	Low
TCGA-66-2780	-0.595776118	High
TCGA-77-8156	-0.87673882	Low
TCGA-66-2790	1.393602552	High
TCGA-98-8022	-0.163579362	High
TCGA-58-A46L	1.791685064	High
TCGA-O2-A52N	0.985934528	High
TCGA-63-7022	-5.056243744	Low
TCGA-37-4135	1.459361088	High
TCGA-66-2727	-0.572792075	High
TCGA-34-7107	-1.358513964	Low
TCGA-NC-A5HD	0.121475511	High

TCGA-56-7582	-0.200658779	High
TCGA-18-3415	-0.633425372	High
TCGA-56-7580	0.075171022	High
TCGA-33-AASL	0.580161353	High
TCGA-43-2576	-1.463899566	Low
TCGA-77-A5FZ	-1.15326389	Low
TCGA-33-4583	-0.012858756	High
TCGA-46-6026	0.655403452	High
TCGA-18-3414	0.773716296	High
TCGA-63-A5MY	-0.023822898	High
TCGA-56-8082	0.380418869	High
TCGA-60-2712	-3.28913149	Low
TCGA-60-2698	-0.005514435	High
TCGA-52-7809	1.659015977	High
TCGA-34-5232	0.473845503	High
TCGA-18-4083	-0.35878798	High
TCGA-37-A5EN	0.178765968	High
TCGA-85-A510	3.004454876	High
TCGA-60-2720	-0.751712899	High
TCGA-60-2710	-0.63860232	High
TCGA-94-8035	-0.061248869	High
TCGA-22-4593	0.384324747	High
TCGA-18-3419	-0.526773938	High
TCGA-66-2793	0.9474929	High
TCGA-21-1079	0.366398699	High
TCGA-92-8064	-1.17486966	Low
TCGA-63-7023	0.383348702	High
TCGA-60-2706	-0.071895742	High
TCGA-98-7454	-0.781164834	High
TCGA-37-4141	2.49E+00	High
TCGA-22-5477	-0.168502593	High
TCGA-33-4533	2.904224877	High
TCGA-77-8128	-0.507964396	High
TCGA-56-8309	-0.123888482	High
TCGA-56-A5DR	0.30198539	High
TCGA-37-4130	4.275100832	High
TCGA-58-A46J	0.320161014	High
TCGA-43-6143	0.528383627	High
TCGA-77-A5G6	0.353002839	High
TCGA-39-5031	-0.76817175	High
TCGA-90-A59Q	0.296029593	High
TCGA-68-7755	-0.556668553	High
TCGA-66-2767	0.521349127	High
TCGA-66-2771	-0.108819361	High
TCGA-85-6560	0.117177862	High
TCGA-56-8304	-0.686302219	High
TCGA-56-7221	0.948729169	High
TCGA-18-3421	0.643462466	High
TCGA-51-6867	0.349975925	High
TCGA-NC-A5HL	0.986366675	High
TCGA-77-A5G1	0.188469689	High
TCGA-96-7545	0.112013158	High
TCGA-NC-A5HM	0.47548564	High
TCGA-33-4589	-0.849673082	Low
TCGA-85-A4QR	0.033057402	High
TCGA-60-2711	-0.436530412	High
TCGA-33-4532	-0.234280262	High
TCGA-NC-A5HJ	3.228184979	High

TCGA-21-5787	-0.081358498	High
TCGA-85-8479	0.430249148	High
TCGA-34-5240	-0.30121038	High
TCGA-77-8148	-0.808205893	Low
TCGA-56-8626	1.418324291	High
TCGA-33-A4WN	-0.690361633	High
TCGA-39-5027	-0.071101713	High
TCGA-56-8503	-0.556750313	High
TCGA-56-8623	1.27485852	High
TCGA-77-6845	-0.159446964	High
TCGA-85-A50Z	0.83906381	High
TCGA-77-8144	0.152044212	High
TCGA-85-A53L	0.350370087	High
TCGA-XC-AA0X	-0.03547021	High
TCGA-66-2782	0.499395281	High
TCGA-68-A59I	0.721002747	High
TCGA-77-A5G7	0.915970897	High
TCGA-90-A4EE	2.123028655	High
TCGA-98-8023	0.163933194	High
TCGA-34-8456	-0.020229148	High
TCGA-56-5897	-2.373501108	Low
TCGA-66-2786	0.24772739	High
TCGA-39-5037	-0.377300872	High
TCGA-56-6545	-0.981304176	Low
TCGA-NK-A5CT	1.452318915	High
TCGA-22-1016	1.416221816	High
TCGA-39-5016	-0.04274036	High
TCGA-63-A5MM	0.243906619	High
TCGA-33-4538	-0.285251767	High
TCGA-63-A5MT	0.402812476	High
TCGA-18-3416	1.320989671	High
TCGA-66-2753	-0.425227048	High
TCGA-63-7020	0.383644313	High
TCGA-56-8308	-1.266085894	Low
TCGA-18-3408	-0.488964107	High
TCGA-NK-A5D1	1.660433826	High
TCGA-85-6798	-0.501471604	High
TCGA-22-4599	-1.632363124	Low
TCGA-56-A4ZK	-0.489884652	High
TCGA-56-7731	-1.483434914	Low
TCGA-92-7341	-0.821997993	Low
TCGA-85-8353	-1.555988253	Low
TCGA-85-A4QQ	0.537810933	High
TCGA-77-6842	0.436144916	High
TCGA-22-4595	-0.577176185	High
TCGA-33-4587	1.630998547	High
TCGA-56-A4BW	1.287954387	High
TCGA-21-1076	0.118648696	High
TCGA-6A-AB49	0.798429364	High
TCGA-63-A5M9	0.407437845	High
TCGA-39-5022	-3.319823689	Low
TCGA-43-6771	-0.336222453	High
TCGA-98-8020	-0.354109889	High
TCGA-77-7141	0.179120728	High
TCGA-92-7340	-0.252878417	High
TCGA-56-8622	-0.91696446	Low
TCGA-70-6722	-0.853544923	Low
TCGA-43-A56U	-3.719582018	Low

TCGA-66-2770	-0.339617018	High
TCGA-85-7710	0.721724158	High
TCGA-58-8387	-1.950737269	Low
TCGA-85-8352	1.305181047	High
TCGA-21-5786	-0.938294924	Low
TCGA-33-AASI	0.007573276	High
TCGA-56-8625	0.338789309	High
TCGA-34-2600	0.458948446	High
TCGA-60-2726	1.695953383	High
TCGA-77-8153	-0.510025035	High
TCGA-66-2791	-0.76825371	High
TCGA-68-7756	-0.557360047	High
TCGA-94-7557	0.02344664	High
TCGA-56-A5DS	0.787422884	High
TCGA-22-4613	-1.448145772	Low
TCGA-77-8130	-0.003300349	High
TCGA-21-1080	0.180126591	High
TCGA-98-A53I	-0.084829753	High
TCGA-34-5236	-1.075191853	Low
TCGA-21-5782	22.50137288	High
TCGA-60-2708	0.199110609	High
TCGA-96-A4JK	-0.801537105	Low
TCGA-66-2788	-1.513627096	Low
TCGA-56-8624	1.43423636	High
TCGA-22-5473	-0.54090439	High
TCGA-21-A5DI	0.682453109	High
TCGA-85-8049	-1.947190998	Low
TCGA-21-5783	0.955757743	High
TCGA-46-3765	-1.52604189	Low
TCGA-39-5021	-1.311001617	Low
TCGA-90-6837	-1.020501676	Low
TCGA-39-5011	-0.765711153	High
TCGA-39-5019	-0.318277333	High
TCGA-98-A539	-0.102826076	High
TCGA-66-2781	-0.883638644	Low
TCGA-37-5819	0.214424932	High
TCGA-56-8201	-1.613089284	Low
TCGA-63-A5MB	0.607594919	High
TCGA-60-2722	-0.168766658	High
TCGA-85-8048	0.705180142	High
TCGA-56-7223	1.346806433	High
TCGA-43-8115	-0.092588521	High
TCGA-18-5592	-0.687188012	High
TCGA-77-7337	-0.189118698	High
TCGA-60-2719	0.022622379	High
TCGA-22-5478	0.161478782	High
TCGA-66-2757	0.368397961	High
TCGA-18-3406	-0.391748172	High
TCGA-77-A5GB	-0.616676328	High
TCGA-21-1070	-0.195247208	High
TCGA-43-A475	2.118772727	High
TCGA-85-7698	-0.131589659	High
TCGA-85-7843	-0.890239798	Low
TCGA-NC-A5HT	0.234071859	High
TCGA-58-8393	1.086888444	High
TCGA-66-2737	-0.041789906	High
TCGA-LA-A7SW	1.10944217	High
TCGA-77-8007	-0.252172921	High

TCGA-85-8354	-0.325128237	High
TCGA-22-5474	-2.08724215	Low
TCGA-77-7140	-0.258660807	High
TCGA-22-4601	0.017606444	High
TCGA-66-2794	-0.299388587	High
TCGA-22-1017	0.631580381	High
TCGA-77-A5GA	-0.127556959	High
TCGA-56-7222	0.30311196	High
TCGA-85-7699	-0.690546996	High
TCGA-66-2758	-0.697650645	High
TCGA-85-7950	3.385592778	High
TCGA-39-5034	-0.751543727	High
TCGA-85-8052	-0.645718199	High
TCGA-43-3394	0.066608602	High
TCGA-NK-A5CX	-0.250560304	High
TCGA-39-5035	-0.922359429	Low
TCGA-43-6647	-0.570319214	High
TCGA-18-4086	-1.58882369	Low
TCGA-NC-A5HF	0.299596367	High
TCGA-39-5029	-0.860416419	Low
TCGA-43-5668	-0.591146659	High
TCGA-22-1012	-0.379488263	High
TCGA-34-8454	-0.131920205	High
TCGA-60-2709	-3.262863343	Low
TCGA-85-A512	0.508024451	High
TCGA-NC-A5HI	0.632002674	High
TCGA-18-3409	0.541584921	High
TCGA-56-8307	1.100831319	High
TCGA-85-8288	0.727302945	High
TCGA-18-3410	2.174945862	High
TCGA-22-1000	-2.302436982	Low
TCGA-66-2778	-0.278647221	High
TCGA-34-5239	0.623726687	High
TCGA-34-8455	-0.061863877	High
TCGA-66-2765	-0.523043999	High
TCGA-85-8277	-0.696683872	High
TCGA-92-8065	-0.499190912	High
TCGA-46-3766	-2.223422752	Low
TCGA-77-8131	-2.431585374	Low
TCGA-94-A5I4	-0.225532157	High
TCGA-18-3407	-1.428288952	Low
TCGA-77-8139	-1.622769703	Low
TCGA-21-1078	1.066734384	High
TCGA-18-3411	-1.621191252	Low
TCGA-66-2742	-0.13971879	High
TCGA-56-1622	0.344485884	High
TCGA-77-A5G3	0.243460705	High
TCGA-22-5482	-0.511890403	High
TCGA-NK-A7XE	0.273150604	High
TCGA-22-5489	-2.539552993	Low
TCGA-O2-A52W	0.566834374	High
TCGA-52-7812	0.041715824	High
TCGA-46-6025	-0.184047081	High
TCGA-58-8391	-0.101286049	High
TCGA-85-A50M	0.207946479	High
TCGA-18-3417	-0.737819802	High
TCGA-60-2724	0.061846859	High
TCGA-94-A5I6	-0.105154612	High

TCGA-43-8116	-0.127290432	High
TCGA-34-5241	0.637255246	High
TCGA-77-7338	-0.745502128	High
TCGA-56-7822	0.254744324	High
TCGA-85-A4JC	-0.218403789	High
TCGA-66-2792	-0.252122075	High
TCGA-98-A53A	0.353099318	High
TCGA-22-4594	-3.46680348	Low
TCGA-58-8386	-0.055725102	High
TCGA-22-5481	-0.590709777	High
TCGA-66-2754	2.053827103	High
TCGA-21-1082	0.017446049	High
TCGA-58-8392	-0.300726982	High
TCGA-60-2713	-0.478297837	High
TCGA-22-5479	-1.084526761	Low
TCGA-37-4133	-0.36019729	High
TCGA-63-A5MU	-0.448336605	High
TCGA-66-2769	-0.760793446	High
TCGA-LA-A446	-0.156334757	High
TCGA-66-2759	-0.622966807	High
TCGA-22-4609	-2.058630622	Low
TCGA-66-2773	-0.112976694	High
TCGA-66-2755	-0.216236465	High
TCGA-77-8150	-0.280818795	High
TCGA-37-A5EL	0.678401409	High
TCGA-77-8009	-0.103286445	High
TCGA-33-AAS8	-0.096661261	High
TCGA-58-A46N	-0.202447885	High
TCGA-33-AASD	-0.035832984	High
TCGA-63-A5MS	0.488887336	High
TCGA-43-A474	-0.328302871	High
TCGA-43-6770	-0.322126026	High
TCGA-22-4607	0.042314846	High
TCGA-58-A46K	0.048989723	High
TCGA-77-7465	1.032128693	High
TCGA-18-4721	-1.898887205	Low
TCGA-85-A4CL	0.075521681	High
TCGA-22-4604	-1.231774363	Low
TCGA-56-7730	-0.368859856	High
TCGA-39-5039	-0.223910727	High
TCGA-J1-A4AH	-1.599875492	Low
TCGA-92-8063	-0.223447574	High
TCGA-46-3769	-0.580609062	High
TCGA-43-8118	0.066804227	High
TCGA-NC-A5HO	0.917257171	High
TCGA-22-4605	-0.763374738	High
TCGA-60-2714	-1.245004315	Low
TCGA-33-6738	0.162559746	High
TCGA-63-A5MV	1.588337173	High
TCGA-85-A4CN	-0.694704651	High
TCGA-NC-A5HK	0.973535556	High
TCGA-43-3920	2.042073857	High
TCGA-21-1072	-0.238834592	High
TCGA-98-A53B	1.641978695	High
TCGA-85-8072	-0.112186576	High
TCGA-68-A59J	1.031847102	High
TCGA-66-2766	-0.278028993	High
TCGA-NC-A5HR	0.834839001	High

TCGA-NC-A5HE	2.082004084	High
TCGA-98-A53D	2.723903082	High
TCGA-66-2783	-1.651771476	Low
TCGA-77-7138	0.248015041	High
TCGA-56-8629	0.360514375	High
TCGA-79-5596	-0.408746278	High
TCGA-63-A5MJ	-0.161086724	High
TCGA-56-6546	-1.932282195	Low
TCGA-51-4081	0.216405939	High
TCGA-34-5927	1.516347051	High
TCGA-85-8582	0.157081415	High
TCGA-77-8154	0.268025454	High
TCGA-77-6844	0.764260374	High
TCGA-85-8580	-0.073071091	High
TCGA-56-A4ZJ	0.908445009	High
TCGA-21-1083	1.055673543	High
TCGA-46-3767	0.017362484	High
TCGA-56-5898	-0.415102646	High
TCGA-68-7757	0.045145307	High
TCGA-56-A49D	1.501345032	High
TCGA-18-3412	-0.302912675	High
TCGA-37-4132	-0.607726157	High
TCGA-22-5483	0.03492057	High
TCGA-85-A4JB	0.222102727	High
TCGA-60-2725	-0.076331197	High
TCGA-77-7463	-0.065785386	High
TCGA-33-AASJ	0.091794948	High
TCGA-43-7657	-0.392452659	High
TCGA-22-A5C4	1.190221611	High
TCGA-90-7769	-0.330379153	High
TCGA-21-1077	-1.482813217	Low
TCGA-NC-A5HQ	1.53060454	High
TCGA-60-2696	-0.687647973	High
TCGA-22-4591	0.033267325	High
TCGA-77-8143	0.282248978	High
TCGA-NK-A5CR	-0.551077235	High
TCGA-56-7823	0.244348064	High
TCGA-63-A5MN	-2.4731655	Low
TCGA-52-7622	-0.623573495	High

Table S7-IPS score for each individual

ID	ips_ctla4_neg_pd1_neg	ips_ctla4_neg_pd1_pos	ips_ctla4_pos_pd1_neg	ips_ctla4_pos_pd1_pos
TCGA-34-5234	8	7	8	7
TCGA-96-7544	6	6	6	6
TCGA-85-A513	8	8	9	8
TCGA-85-8584	10	9	10	9
TCGA-85-A511	8	7	7	7
TCGA-66-2789	8	6	7	6
TCGA-22-0940	8	8	8	8
TCGA-96-A4JL	8	8	8	9
TCGA-77-8136	7	7	6	7
TCGA-63-5131	7	6	7	5
TCGA-90-7964	6	5	6	4
TCGA-85-A5B5	6	6	6	5
TCGA-37-3789	8	8	8	8
TCGA-O2-A5IB	7	5	7	5
TCGA-58-8390	7	5	7	5
TCGA-66-2777	7	6	7	6
TCGA-77-8140	7	6	7	5
TCGA-98-A538	7	6	6	5
TCGA-66-2785	8	9	8	9
TCGA-43-5670	8	6	8	6
TCGA-22-5472	7	6	7	5
TCGA-60-2704	6	6	6	6
TCGA-77-6843	7	6	6	5
TCGA-63-A5MG	7	6	7	6
TCGA-66-2734	6	5	5	4
TCGA-43-2581	7	6	6	6
TCGA-52-7811	8	7	8	7
TCGA-85-8287	8	8	8	8
TCGA-85-8355	7	8	7	8
TCGA-94-7033	9	8	9	7
TCGA-60-2697	9	8	9	8
TCGA-77-8145	7	6	7	6
TCGA-85-7844	7	6	6	6
TCGA-39-5040	7	7	7	6
TCGA-66-2795	7	7	7	6
TCGA-33-4586	6	6	6	6
TCGA-NC-A5HH	6	4	6	4
TCGA-22-1002	8	7	7	6
TCGA-60-2695	7	7	7	6
TCGA-77-A5G8	6	6	6	5
TCGA-63-A5MP	7	8	7	8
TCGA-22-0944	9	7	8	7
TCGA-21-1081	6	4	5	4
TCGA-O2-A52V	7	6	6	6
TCGA-33-AASB	7	6	7	5
TCGA-63-5128	7	5	6	5
TCGA-96-8170	8	6	7	6
TCGA-63-A5MW	8	6	7	6
TCGA-37-3792	6	7	7	7
TCGA-56-A4BX	7	6	6	5
TCGA-56-8083	8	6	7	5
TCGA-98-A53H	7	7	7	7
TCGA-94-A4VJ	9	8	9	8
TCGA-43-7658	7	7	7	7
TCGA-NC-A5HN	9	8	9	8
TCGA-63-7021	7	7	7	7
TCGA-22-5471	9	9	8	8
TCGA-77-8133	8	8	8	8
TCGA-37-A5EM	7	6	7	6
TCGA-85-8071	8	7	8	7
TCGA-NC-A5HP	8	6	7	6
TCGA-85-A4PA	9	9	10	10
TCGA-33-4582	7	5	6	4
TCGA-43-2578	8	7	7	6
TCGA-34-A5IX	7	6	7	5
TCGA-56-8504	8	7	7	7
TCGA-18-5595	8	7	7	6
TCGA-21-5784	9	7	8	7
TCGA-90-A4ED	8	8	8	7
TCGA-MF-A522	7	6	7	5
TCGA-46-3768	7	6	7	6
TCGA-77-7335	7	7	7	7
TCGA-56-A4BY	8	8	8	7

TCGA-85-8070	8	8	7	7
TCGA-56-7579	7	5	7	5
TCGA-NC-A5HG	8	6	8	6
TCGA-56-8305	7	6	7	5
TCGA-60-2703	7	5	6	5
TCGA-39-5036	9	8	8	7
TCGA-33-6737	6	5	6	5
TCGA-34-2608	8	7	7	7
TCGA-39-5024	9	8	9	7
TCGA-77-7142	8	6	7	6
TCGA-85-8350	9	7	8	7
TCGA-O2-A52Q	7	7	7	8
TCGA-68-8250	7	7	7	6
TCGA-70-6723	9	7	8	7
TCGA-68-8251	8	7	7	7
TCGA-85-7697	9	7	8	7
TCGA-66-2756	7	5	6	5
TCGA-85-6561	7	7	7	7
TCGA-94-7943	8	7	8	6
TCGA-22-1011	8	7	7	7
TCGA-85-8664	6	4	6	4
TCGA-63-A5MH	6	6	6	5
TCGA-94-8490	7	6	6	5
TCGA-85-8481	8	7	8	7
TCGA-60-2716	7	6	7	5
TCGA-O2-A52S	7	6	7	6
TCGA-51-4080	7	7	7	6
TCGA-22-5485	7	6	6	5
TCGA-85-6175	7	7	7	7
TCGA-85-8276	8	7	8	7
TCGA-77-8008	6	6	6	5
TCGA-39-5028	7	7	7	6
TCGA-34-5928	7	6	7	6
TCGA-43-6773	8	9	8	8
TCGA-63-A5MI	7	6	7	5
TCGA-63-A5ML	7	8	7	7
TCGA-39-5030	7	8	8	8
TCGA-51-4079	7	7	7	7
TCGA-22-5491	7	6	7	6
TCGA-66-2744	7	8	7	8
TCGA-33-4547	7	5	7	5
TCGA-52-7810	7	5	6	5
TCGA-77-7139	7	7	7	7
TCGA-66-2800	7	6	6	6
TCGA-33-4566	8	8	8	8
TCGA-90-7766	7	7	7	7
TCGA-60-2707	7	6	6	5
TCGA-56-8628	7	6	7	6
TCGA-85-8666	6	5	6	5
TCGA-60-2721	8	8	8	8
TCGA-22-4596	7	7	7	7
TCGA-33-A5GW	7	6	6	5
TCGA-60-2715	8	7	8	7
TCGA-85-8351	7	6	7	6
TCGA-58-A46M	7	8	7	7
TCGA-37-4129	7	5	6	4
TCGA-21-1075	6	5	5	5
TCGA-43-7656	7	7	7	7
TCGA-58-8388	7	7	7	7
TCGA-98-A53J	7	6	6	5
TCGA-85-7696	9	7	8	7
TCGA-34-5231	9	8	9	8
TCGA-34-5929	8	7	8	6
TCGA-22-5492	7	7	7	7
TCGA-98-A53C	8	8	8	8
TCGA-94-8491	8	7	8	7
TCGA-90-7767	8	8	7	8
TCGA-96-8169	7	6	7	6
TCGA-34-2596	7	8	6	7
TCGA-56-A62T	7	6	7	6
TCGA-21-1071	6	6	6	6
TCGA-22-5480	9	9	9	9
TCGA-77-A5GH	7	8	7	8
TCGA-L3-A4E7	7	7	7	6
TCGA-60-2723	7	8	7	8
TCGA-37-3783	9	8	9	8

TCGA-66-2763	6	6	6	6
TCGA-63-6202	7	7	7	7
TCGA-L3-A524	7	5	6	5
TCGA-98-8021	8	6	7	6
TCGA-77-A5GF	8	8	7	8
TCGA-66-2787	7	7	6	7
TCGA-77-8138	7	6	7	6
TCGA-63-A5MR	8	7	7	6
TCGA-66-2768	9	8	8	8
TCGA-77-8146	7	6	7	6
TCGA-43-A56V	6	4	6	4
TCGA-22-1005	9	9	10	9
TCGA-66-2780	8	7	8	7
TCGA-77-8156	8	7	7	7
TCGA-66-2790	8	8	8	7
TCGA-98-8022	9	8	9	8
TCGA-58-A46L	7	7	7	7
TCGA-O2-A52N	9	8	9	8
TCGA-63-7022	8	7	8	7
TCGA-37-4135	8	7	7	6
TCGA-66-2727	7	7	7	7
TCGA-34-7107	7	7	7	7
TCGA-NC-A5HD	7	5	6	4
TCGA-56-7582	6	5	6	5
TCGA-18-3415	8	8	8	8
TCGA-56-7580	8	7	8	6
TCGA-33-AASL	8	7	8	7
TCGA-43-2576	7	6	7	6
TCGA-77-A5FZ	7	8	8	8
TCGA-33-4583	9	9	8	9
TCGA-46-6026	7	5	6	4
TCGA-18-3414	8	7	7	6
TCGA-63-A5MY	5	3	4	3
TCGA-56-8082	7	6	6	5
TCGA-60-2712	8	8	8	8
TCGA-60-2698	8	7	7	7
TCGA-52-7809	8	6	7	6
TCGA-34-5232	8	7	8	8
TCGA-18-4083	6	7	6	7
TCGA-37-A5EN	9	8	8	7
TCGA-85-A510	8	7	8	7
TCGA-60-2720	9	8	9	7
TCGA-60-2710	7	7	7	7
TCGA-94-8035	6	5	6	5
TCGA-22-4593	7	6	7	5
TCGA-18-3419	6	6	6	5
TCGA-66-2793	7	5	6	4
TCGA-21-1079	7	7	7	7
TCGA-92-8064	7	8	7	9
TCGA-63-7023	9	8	9	7
TCGA-60-2706	7	7	7	7
TCGA-98-7454	7	7	7	7
TCGA-37-4141	8	8	8	7
TCGA-22-5477	7	7	6	7
TCGA-33-4533	8	6	8	6
TCGA-77-8128	7	6	7	5
TCGA-56-8309	7	8	7	8
TCGA-56-A5DR	6	5	6	5
TCGA-37-4130	8	8	8	8
TCGA-58-A46J	7	6	7	5
TCGA-43-6143	8	7	7	6
TCGA-77-A5G6	7	5	6	5
TCGA-39-5031	8	7	7	6
TCGA-90-A59Q	8	8	8	8
TCGA-68-7755	7	7	7	7
TCGA-66-2767	8	8	8	9
TCGA-66-2771	7	7	7	7
TCGA-85-6560	6	6	6	6
TCGA-56-8304	7	7	7	6
TCGA-56-7221	8	6	7	5
TCGA-18-3421	7	6	6	6
TCGA-51-6867	7	8	7	7
TCGA-NC-A5HL	8	9	8	9
TCGA-77-A5G1	7	6	7	6
TCGA-96-7545	7	7	7	7
TCGA-NC-A5HM	9	8	9	8

TCGA-33-4589	8	8	8	8
TCGA-85-A4QR	7	6	6	5
TCGA-60-2711	8	7	8	7
TCGA-33-4532	7	5	6	5
TCGA-NC-A5HJ	8	9	8	8
TCGA-21-5787	8	8	8	8
TCGA-85-8479	7	5	7	5
TCGA-34-5240	6	6	6	5
TCGA-77-8148	8	7	7	6
TCGA-56-8626	8	6	7	5
TCGA-33-A4WN	7	6	7	6
TCGA-39-5027	8	7	7	6
TCGA-56-8503	7	7	7	7
TCGA-56-8623	8	8	8	8
TCGA-77-6845	6	5	5	4
TCGA-85-A50Z	7	6	6	5
TCGA-77-8144	8	7	7	7
TCGA-85-A53L	7	5	6	5
TCGA-XC-AA0X	7	6	7	6
TCGA-66-2782	7	6	7	6
TCGA-68-A59I	7	6	7	6
TCGA-77-A5G7	8	6	8	6
TCGA-90-A4EE	7	8	8	8
TCGA-98-8023	8	7	8	7
TCGA-34-8456	7	7	7	7
TCGA-56-5897	7	7	7	7
TCGA-66-2786	8	7	8	7
TCGA-39-5037	9	8	8	7
TCGA-56-6545	7	7	7	7
TCGA-NK-A5CT	7	7	7	6
TCGA-22-1016	7	6	6	5
TCGA-39-5016	8	9	8	8
TCGA-63-A5MM	8	8	8	9
TCGA-33-4538	8	8	8	8
TCGA-63-A5MT	7	7	7	6
TCGA-18-3416	8	9	8	9
TCGA-66-2753	7	6	6	5
TCGA-63-7020	8	7	8	7
TCGA-56-8308	8	8	7	7
TCGA-18-3408	7	7	6	6
TCGA-NK-A5D1	9	7	8	7
TCGA-85-6798	7	6	6	6
TCGA-22-4599	7	7	7	7
TCGA-56-A4ZK	6	6	6	6
TCGA-56-7731	7	6	7	6
TCGA-92-7341	7	6	6	6
TCGA-85-8353	8	7	8	7
TCGA-85-A4QQ	8	6	7	6
TCGA-77-6842	6	8	6	8
TCGA-22-4595	7	6	7	6
TCGA-33-4587	7	6	7	5
TCGA-56-A4BW	8	7	8	7
TCGA-21-1076	7	7	7	7
TCGA-6A-AB49	7	6	7	6
TCGA-63-A5M9	7	7	7	7
TCGA-39-5022	9	9	9	9
TCGA-43-6771	7	7	7	7
TCGA-98-8020	8	7	7	7
TCGA-77-7141	7	7	7	6
TCGA-92-7340	7	6	7	6
TCGA-56-8622	8	7	7	6
TCGA-70-6722	7	8	7	7
TCGA-43-A56U	8	8	8	8
TCGA-66-2770	7	7	7	7
TCGA-85-7710	8	8	8	7
TCGA-58-8387	8	9	8	9
TCGA-85-8352	7	5	6	5
TCGA-21-5786	8	6	7	6
TCGA-33-AASI	10	9	9	9
TCGA-56-8625	7	7	7	7
TCGA-34-2600	7	5	7	5
TCGA-60-2726	9	8	9	7
TCGA-77-8153	7	5	6	5
TCGA-66-2791	7	8	7	8
TCGA-68-7756	7	6	7	6
TCGA-94-7557	6	5	6	5

TCGA-56-A5DS	8	6	7	6
TCGA-22-4613	7	7	7	7
TCGA-77-8130	8	7	7	6
TCGA-21-1080	6	6	6	5
TCGA-98-A53I	8	8	8	7
TCGA-34-5236	7	5	6	5
TCGA-21-5782	8	7	8	6
TCGA-60-2708	6	6	6	6
TCGA-96-A4JK	8	7	8	7
TCGA-66-2788	7	7	7	7
TCGA-56-8624	8	6	7	6
TCGA-22-5473	7	7	6	6
TCGA-21-A5DI	7	6	7	5
TCGA-85-8049	7	7	7	7
TCGA-21-5783	8	6	7	6
TCGA-46-3765	8	7	7	6
TCGA-39-5021	8	7	7	7
TCGA-90-6837	7	6	6	6
TCGA-39-5011	7	8	7	7
TCGA-39-5019	8	7	7	7
TCGA-98-A539	7	6	6	5
TCGA-66-2781	8	7	8	6
TCGA-37-5819	8	6	7	6
TCGA-56-8201	6	7	7	7
TCGA-63-A5MB	7	7	7	6
TCGA-60-2722	7	7	6	6
TCGA-85-8048	8	8	8	8
TCGA-56-7223	6	4	5	4
TCGA-43-8115	7	7	7	7
TCGA-18-5592	8	7	8	7
TCGA-77-7337	7	6	7	6
TCGA-60-2719	7	6	7	6
TCGA-22-5478	7	5	6	5
TCGA-66-2757	8	7	7	6
TCGA-18-3406	9	8	9	8
TCGA-77-A5GB	8	7	8	7
TCGA-21-1070	7	8	7	8
TCGA-43-A475	7	9	7	9
TCGA-85-7698	7	8	7	8
TCGA-85-7843	7	6	6	5
TCGA-NC-A5HT	9	7	8	6
TCGA-58-8393	7	7	7	7
TCGA-66-2737	7	7	7	7
TCGA-LA-A7SW	6	5	6	5
TCGA-77-8007	6	7	6	7
TCGA-85-8354	7	6	7	6
TCGA-22-5474	8	9	8	9
TCGA-77-7140	6	5	5	5
TCGA-22-4601	8	7	8	7
TCGA-66-2794	8	9	8	9
TCGA-22-1017	7	7	7	7
TCGA-77-A5GA	7	7	7	6
TCGA-56-7222	8	6	8	6
TCGA-85-7699	7	7	7	7
TCGA-66-2758	7	7	7	7
TCGA-85-7950	8	8	8	8
TCGA-39-5034	8	7	7	7
TCGA-85-8052	7	6	7	6
TCGA-43-3394	7	7	7	6
TCGA-NK-A5CX	8	7	8	7
TCGA-39-5035	8	9	8	9
TCGA-43-6647	6	7	6	7
TCGA-18-4086	8	8	8	8
TCGA-NC-A5HF	6	5	6	4
TCGA-39-5029	8	6	7	6
TCGA-43-5668	7	8	7	8
TCGA-22-1012	7	6	7	6
TCGA-34-8454	8	7	8	7
TCGA-60-2709	7	8	7	8
TCGA-85-A512	7	6	7	6
TCGA-NC-A5HI	6	4	6	4
TCGA-18-3409	7	7	7	6
TCGA-56-8307	8	8	8	8
TCGA-85-8288	9	8	8	8
TCGA-18-3410	7	9	7	9
TCGA-22-1000	7	7	8	7

TCGA-66-2778	7	6	7	5
TCGA-34-5239	8	8	7	7
TCGA-34-8455	8	7	7	6
TCGA-66-2765	8	8	8	8
TCGA-85-8277	8	8	7	8
TCGA-92-8065	7	6	6	6
TCGA-46-3766	8	7	8	7
TCGA-77-8131	7	7	7	7
TCGA-94-A5I4	7	6	7	5
TCGA-18-3407	7	7	7	7
TCGA-77-8139	8	7	7	6
TCGA-21-1078	8	7	8	6
TCGA-18-3411	6	6	6	5
TCGA-66-2742	7	6	6	5
TCGA-56-1622	7	7	7	7
TCGA-77-A5G3	7	7	6	6
TCGA-22-5482	8	6	7	6
TCGA-NK-A7XE	8	7	8	6
TCGA-22-5489	8	8	8	8
TCGA-O2-A52W	7	5	6	5
TCGA-52-7812	8	9	8	8
TCGA-46-6025	7	6	6	6
TCGA-58-8391	7	6	6	5
TCGA-85-A50M	7	6	6	6
TCGA-18-3417	8	7	8	7
TCGA-60-2724	7	6	6	5
TCGA-94-A5I6	6	6	6	5
TCGA-43-8116	8	7	8	7
TCGA-34-5241	8	7	8	7
TCGA-77-7338	7	6	6	5
TCGA-56-7822	7	6	7	5
TCGA-85-A4JC	8	6	7	6
TCGA-66-2792	7	7	6	6
TCGA-98-A53A	7	6	7	6
TCGA-22-4594	8	7	7	7
TCGA-58-8386	7	7	6	6
TCGA-22-5481	8	8	8	8
TCGA-66-2754	7	5	6	5
TCGA-21-1082	8	7	8	6
TCGA-58-8392	8	7	7	6
TCGA-60-2713	7	7	7	7
TCGA-22-5479	7	7	7	7
TCGA-37-4133	8	8	7	8
TCGA-63-A5MU	7	6	7	6
TCGA-66-2769	7	7	7	7
TCGA-LA-A446	7	6	6	6
TCGA-66-2759	5	7	5	6
TCGA-22-4609	7	7	7	7
TCGA-66-2773	7	7	6	6
TCGA-66-2755	7	8	7	7
TCGA-77-8150	7	7	7	7
TCGA-37-A5EL	10	9	10	8
TCGA-77-8009	7	6	6	6
TCGA-33-AAS8	7	6	7	6
TCGA-58-A46N	6	6	6	6
TCGA-33-AASD	8	7	8	6
TCGA-63-A5MS	8	7	8	7
TCGA-43-A474	7	5	6	4
TCGA-43-6770	6	6	6	5
TCGA-22-4607	7	6	7	6
TCGA-58-A46K	8	9	8	8
TCGA-77-7465	7	7	7	7
TCGA-18-4721	7	8	7	8
TCGA-85-A4CL	7	6	7	6
TCGA-22-4604	7	7	7	7
TCGA-56-7730	7	6	7	6
TCGA-39-5039	8	7	8	6
TCGA-J1-A4AH	7	6	6	6
TCGA-92-8063	6	5	6	5
TCGA-46-3769	8	8	8	8
TCGA-43-8118	7	6	6	5
TCGA-NC-A5HO	7	5	6	5
TCGA-22-4605	6	5	6	5
TCGA-60-2714	7	8	7	8
TCGA-33-6738	8	6	7	6
TCGA-63-A5MV	6	6	6	6

TCGA-85-A4CN	8	7	7	7
TCGA-NC-A5HK	7	8	6	7
TCGA-43-3920	6	6	6	6
TCGA-21-1072	7	7	7	6
TCGA-98-A53B	7	6	7	6
TCGA-85-8072	8	8	8	8
TCGA-68-A59J	8	8	8	8
TCGA-66-2766	7	7	7	7
TCGA-NC-A5HR	8	7	7	7
TCGA-NC-A5HE	7	8	7	8
TCGA-98-A53D	7	7	7	7
TCGA-66-2783	7	7	7	7
TCGA-77-7138	8	6	7	6
TCGA-56-8629	7	6	7	6
TCGA-79-5596	7	6	7	6
TCGA-63-A5MJ	7	7	6	6
TCGA-56-6546	8	7	7	7
TCGA-51-4081	6	5	6	5
TCGA-34-5927	7	5	6	4
TCGA-85-8582	7	7	7	6
TCGA-77-8154	8	6	7	5
TCGA-77-6844	7	6	7	6
TCGA-85-8580	7	6	7	6
TCGA-56-A4ZJ	7	6	7	6
TCGA-21-1083	7	7	7	7
TCGA-46-3767	8	7	7	7
TCGA-56-5898	8	7	7	6
TCGA-68-7757	7	6	7	6
TCGA-56-A49D	8	7	7	7
TCGA-18-3412	8	7	7	6
TCGA-37-4132	9	8	9	9
TCGA-22-5483	7	6	7	6
TCGA-85-A4JB	7	5	6	5
TCGA-60-2725	7	7	7	7
TCGA-77-7463	6	6	6	5
TCGA-33-AASJ	8	8	8	8
TCGA-43-7657	9	7	8	7
TCGA-22-A5C4	6	4	5	4
TCGA-90-7769	6	6	6	5
TCGA-21-1077	7	7	7	6
TCGA-NC-A5HQ	7	7	7	7
TCGA-60-2696	7	6	7	6
TCGA-22-4591	8	6	7	6
TCGA-77-8143	6	6	6	5
TCGA-NK-A5CR	6	5	5	5
TCGA-56-7823	7	6	6	5
TCGA-63-A5MN	8	9	8	9
TCGA-52-7622	7	8	7	8
