# Supplementary figures and images for: Characterization of the Immune Microenvironmental Landscape of Lung Squamous Cell Carcinoma with Immune Cell Infiltration
Source: Dis Markers. 2022 Nov 11;2022:2361507. doi: 10.1155/2022/2361507 (PMC9674995; doi:10.1155/2022/2361507)

A

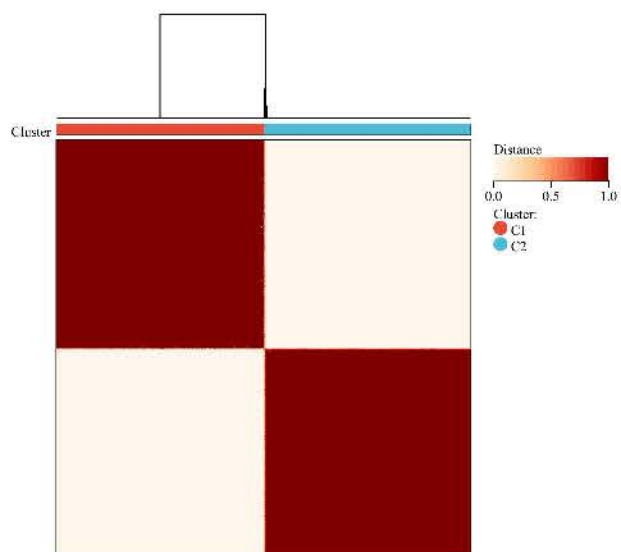

B

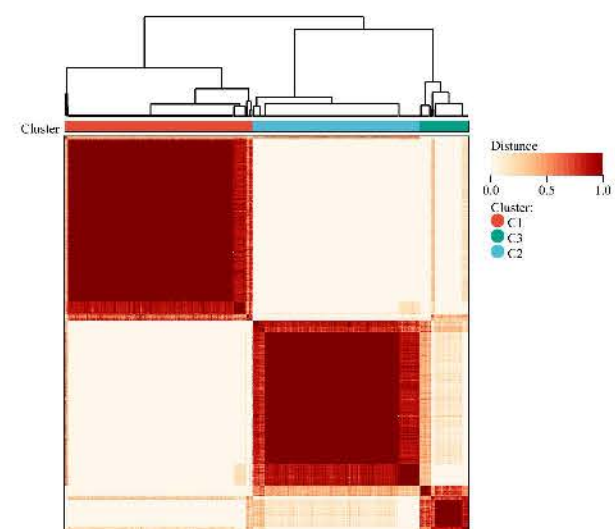

C

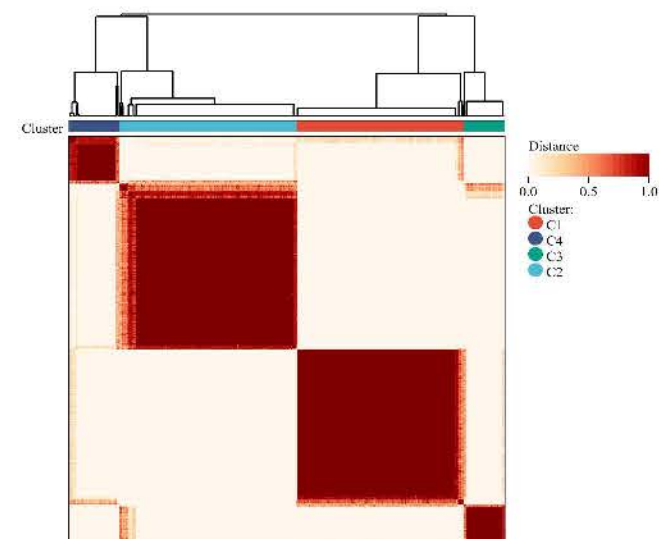

D

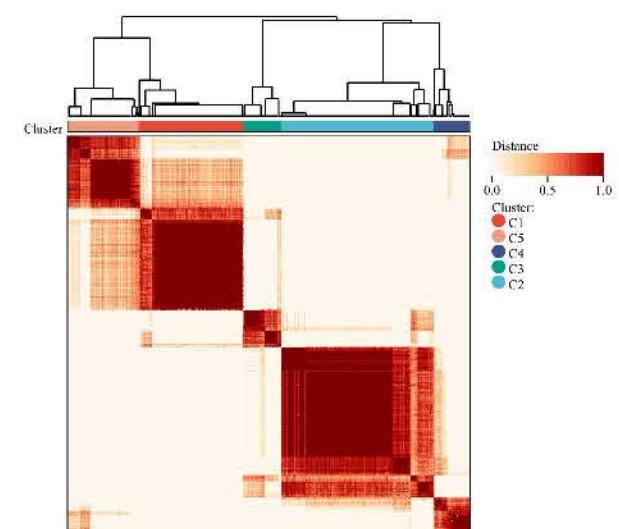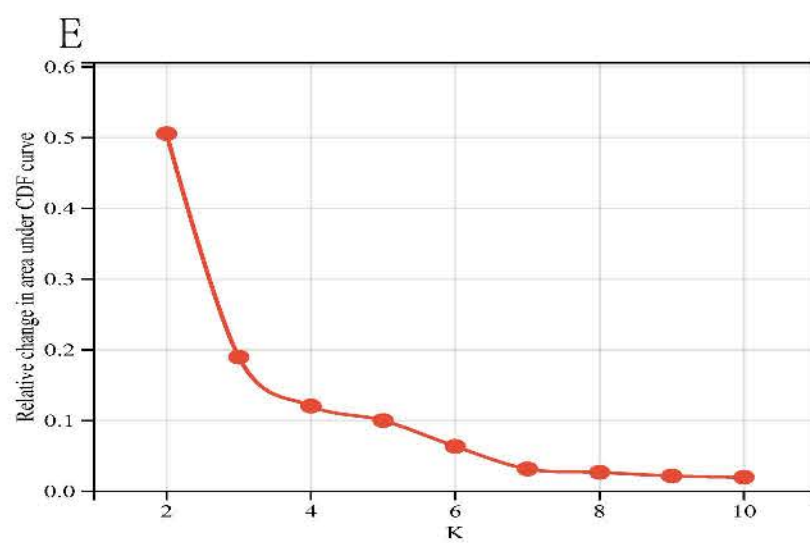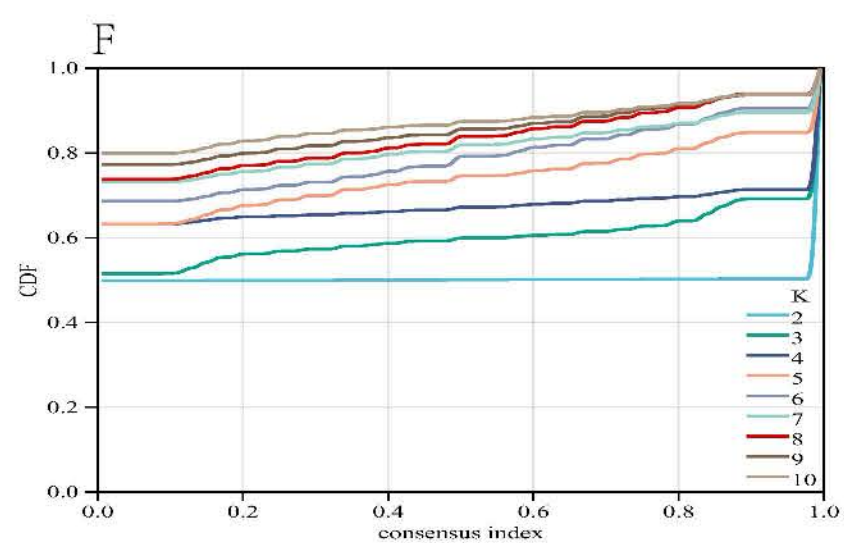

G

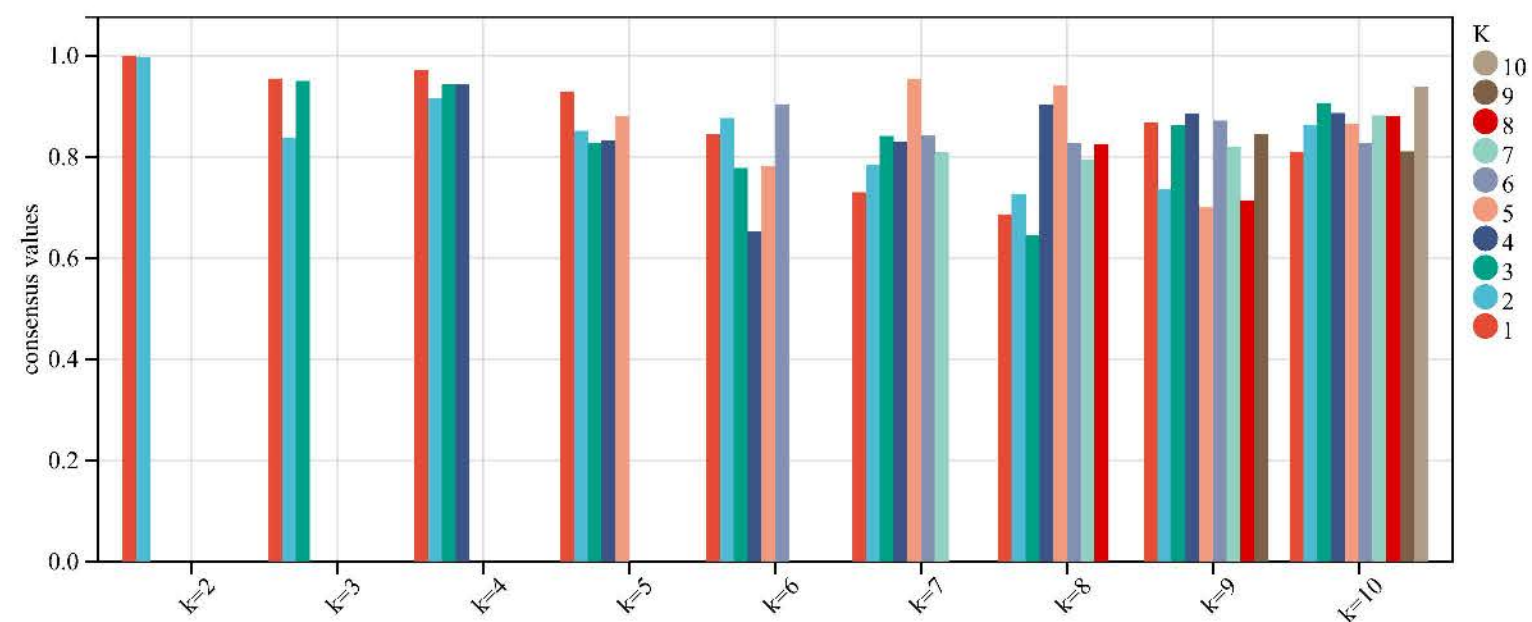

A

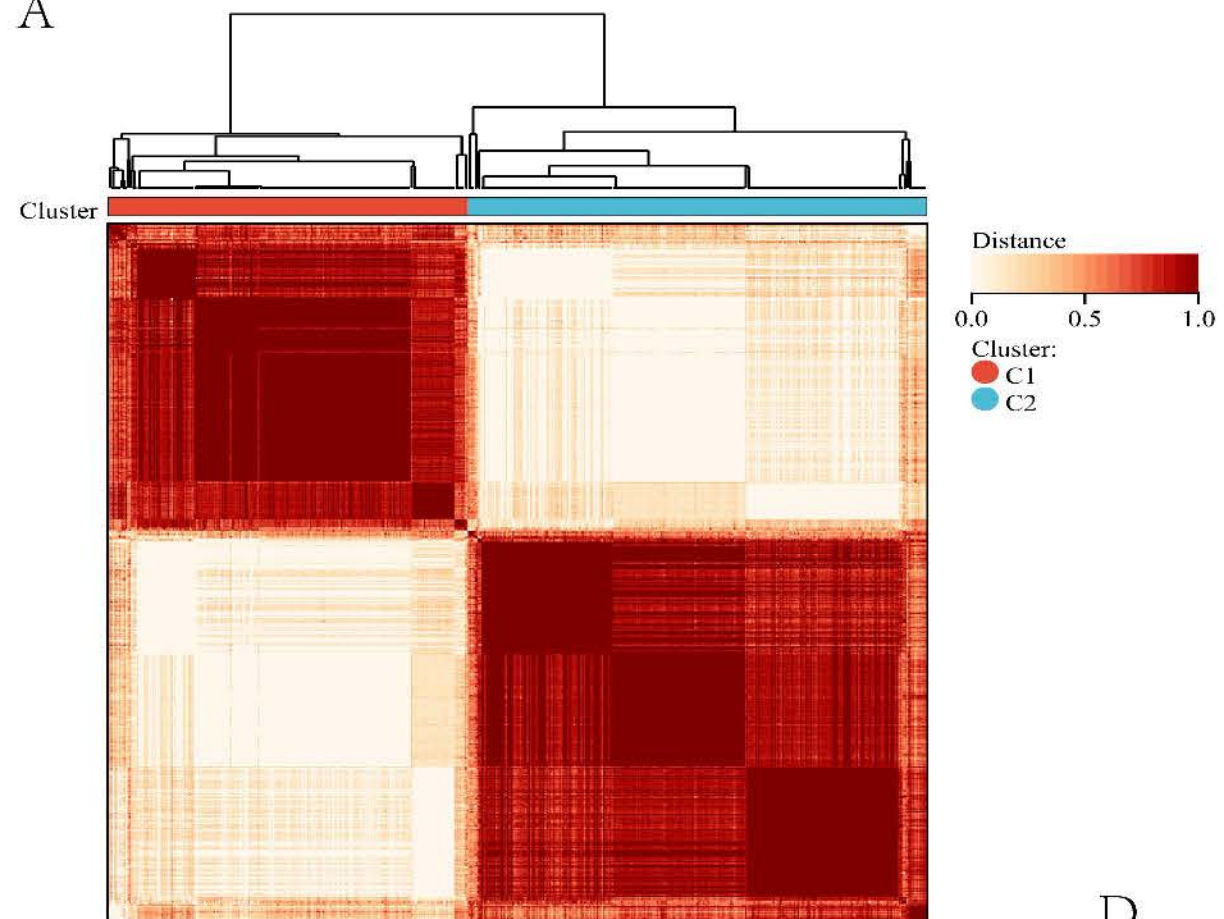

B

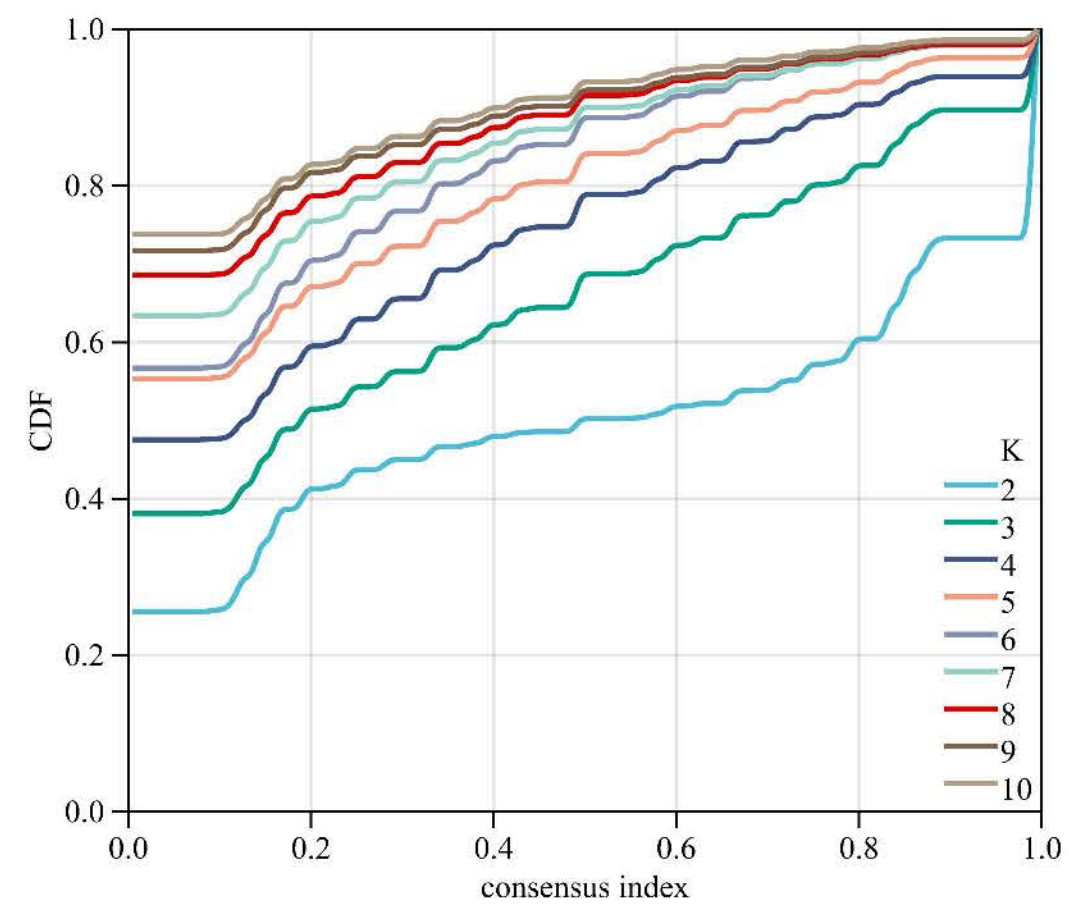

D

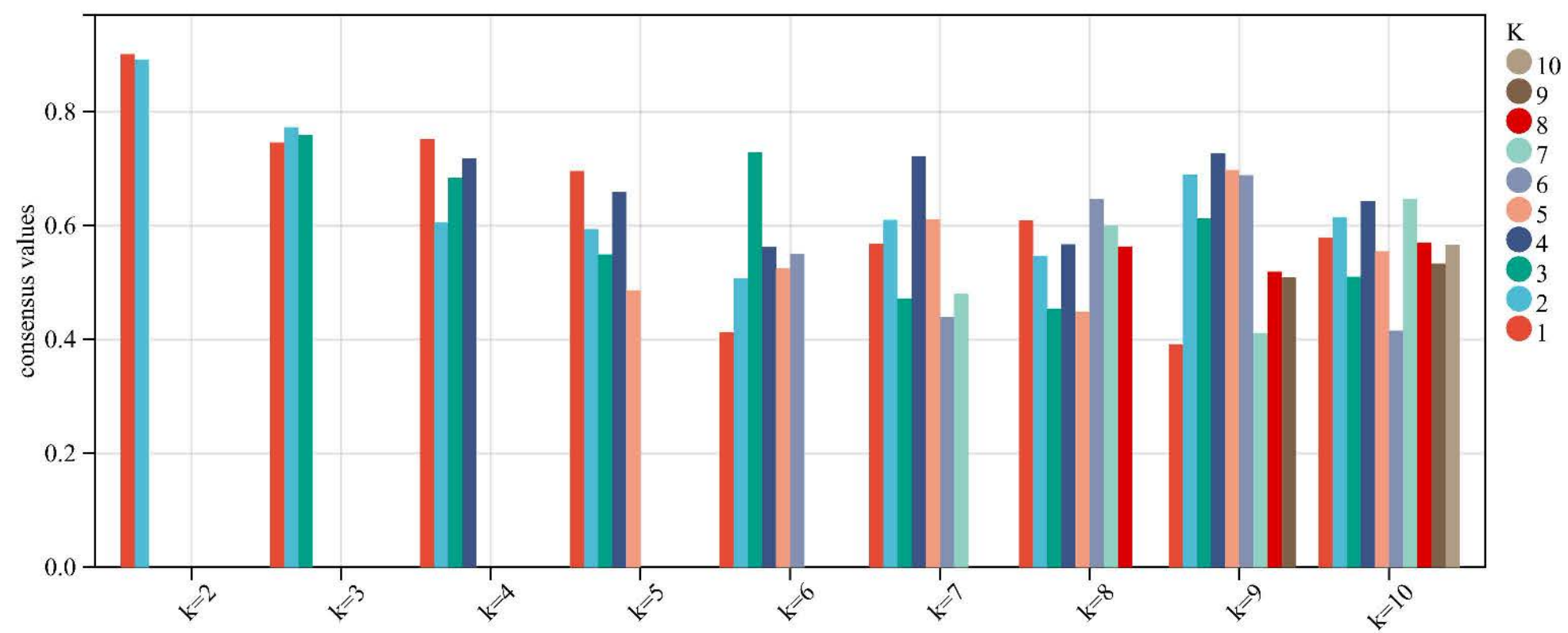

C

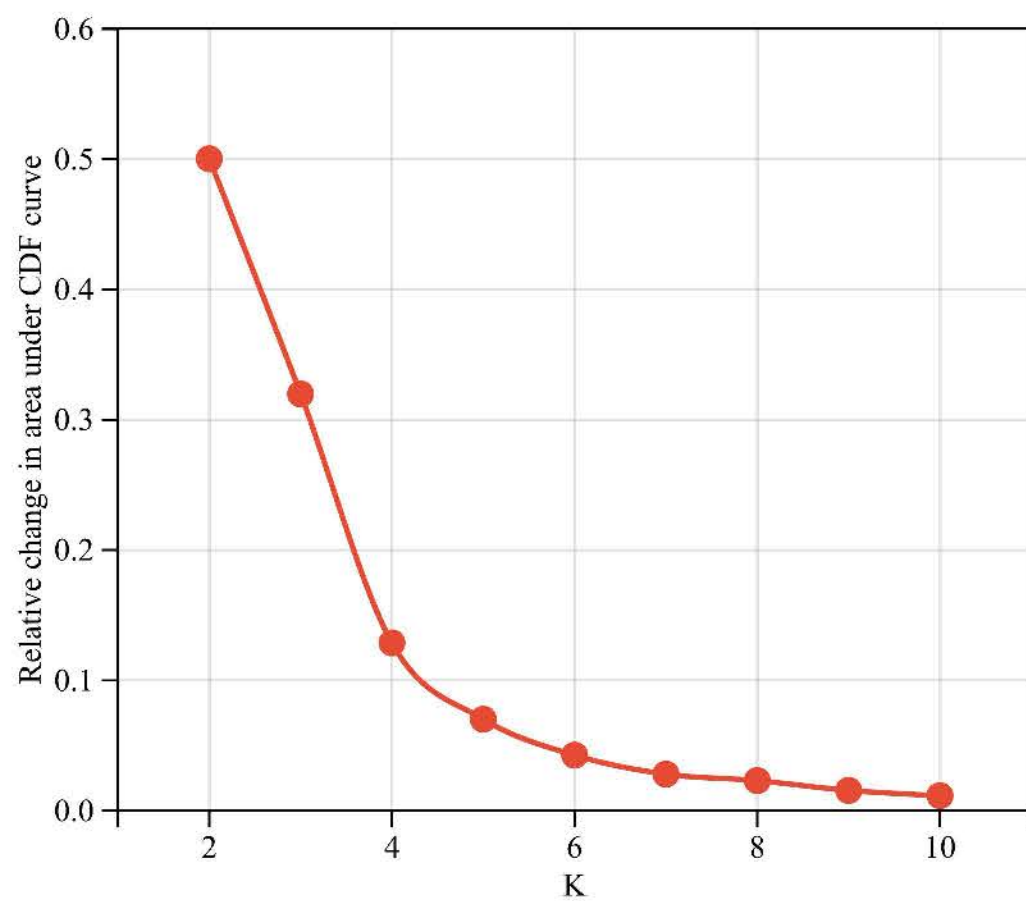

Supplement: Supplementary 2 — Supplementary Figure 1: consensus clustering based on the ICI patterns. (A–D) Consensus matrixes of BRCA samples for each k (k = 2–5), displaying the clustering stability using 1000 iterations of hierarchical clustering. (E) The relative change in area under the CDF curve for k = 2–9. (F) Area under cumulative distribution function (CDF) curve when index k ranges from 2 to 10. (G) Bar graph of consensus values when index k ranges from 2 to 10. Supplementary Figure 2: consensus clustering based on DEGs of different ICI clusters. (A) Consensus matrixes of BRCA cohorts for k = 2 displaying the clustering stability using 1000 iterations of hierarchical clustering. (B) Area under cumulative distribution function (CDF) curve when index k ranges from 2 to 10. (C) The relative change in area under the CDF curve for k = 2–10. (D) Bar graph of consensus values when index k ranges from 2 to 10. [file 2361507.f2.pdf]
